# Human T-cell leukemia virus type 1 infects multiple lineage hematopoietic cells *in vivo*

**DOI:** 10.1371/journal.ppat.1006722

**Published:** 2017-11-29

**Authors:** Rie Furuta, Jun-ichirou Yasunaga, Michi Miura, Kenji Sugata, Akatsuki Saito, Hirofumi Akari, Takaharu Ueno, Norihiro Takenouchi, Jun-ichi Fujisawa, Ki-Ryang Koh, Yusuke Higuchi, Mohamed Mahgoub, Masakazu Shimizu, Fumihiko Matsuda, Anat Melamed, Charles R. Bangham, Masao Matsuoka

**Affiliations:** 1 Laboratory of Virus Control, Institute for Frontier Life and Medical Sciences, Kyoto University, Kyoto, Japan; 2 Department of Hematology, Rheumatology, and Infectious Diseases, Graduate School of Medical Sciences, Faculty of Life Sciences, Kumamoto University, Kumamoto, Japan; 3 Center for Human Evolution Modeling Research, Primate Research Institute, Kyoto University, Inuyama, Aich, Japan; 4 Laboratory of Infectious Disease Model, Institute for Frontier Life and Medical Sciences, Kyoto University, Kyoto, Japan; 5 Department of Microbiology, Kansai Medical University, Hirakata, Osaka, Japan; 6 Department of Hematology, Osaka General Hospital of West Japan Railway Company, Osaka, Japan; 7 Center for Genomic Medicine, Kyoto University Graduate School of Medicine, Kyoto, Japan; 8 Section of Virology, Department of Medicine, Imperial College London, London, United Kingdom; University of Illinois at Chicago College of Medicine, UNITED STATES

## Abstract

Human T-cell leukemia virus type 1 (HTLV-1) infects mainly CD4^+^CCR4^+^ effector/memory T cells *in vivo*. However, it remains unknown whether HTLV-1 preferentially infects these T cells or this virus converts infected precursor cells to specialized T cells. Expression of viral genes *in vivo* is critical to study viral replication and proliferation of infected cells. Therefore, we first analyzed viral gene expression in non-human primates naturally infected with simian T-cell leukemia virus type 1 (STLV-1), whose virological attributes closely resemble those of HTLV-1. Although the *tax* transcript was detected only in certain tissues, Tax expression was much higher in the bone marrow, indicating the possibility of *de novo* infection. Furthermore, Tax expression of non-T cells was suspected in bone marrow. These data suggest that HTLV-1 infects hematopoietic cells in the bone marrow. To explore the possibility that HTLV-1 infects hematopoietic stem cells (HSCs), we analyzed integration sites of HTLV-1 provirus in various lineages of hematopoietic cells in patients with HTLV-1 associated myelopathy/tropical spastic paraparesis (HAM/TSP) and a HTLV-1 carrier using the high-throughput sequencing method. Identical integration sites were detected in neutrophils, monocytes, B cells, CD8^+^ T cells and CD4^+^ T cells, indicating that HTLV-1 infects HSCs *in vivo*. We also detected Tax protein in myeloperoxidase positive neutrophils. Furthermore, dendritic cells differentiated from HTLV-1 infected monocytes caused *de novo* infection to T cells, indicating that infected monocytes are implicated in viral spreading *in vivo*. Certain integration sites were re-detected in neutrophils from HAM/TSP patients at different time points, indicating that infected HSCs persist and differentiate *in vivo*. This study demonstrates that HTLV-1 infects HSCs, and infected stem cells differentiate into diverse cell lineages. These data indicate that infection of HSCs can contribute to the persistence and spread of HTLV-1 *in vivo*.

## Author summary

HTLV-1 primarily infects peripheral CD4^+^CCR4^+^CADM1^+^ T cells *in vivo*. In this study, we show that HTLV-1 infects HSCs, which differentiate into multiple lineages of hematopoietic cells, and likely act as viral reservoir, giving rise to infected neutrophils, monocytes and B cells. Infected monocytes are thought to spread virus *in vivo*. Infected T cells in the periphery are chimeric in origin: T cells newly infected in the periphery, and infected T cells differentiated from infected HSCs. This observation suggests that viral genes such as *HBZ* and *tax* are responsible for converging the molecular differentiation program into a single direction with the characteristic immunophenotype associated with the expression of CCR4 and CADM1. It has been believed that HTLV-1 infects target cells in the periphery. However, this study reveals a new strategy of HTLV-1 spreading *in vivo*. These findings have implications for understanding of HTLV-1 pathogenesis as well as treatment of HTLV-1 associated diseases.

## Introduction

Human T-cell leukemia virus type 1 (HTLV-1) is the causal agent of adult T-cell leukemia-lymphoma (ATL) and inflammatory diseases including HTLV-1 associated myelopathy/tropical spastic paraparesis (HAM/TSP) [1–4]. HTLV-1 is a unique retrovirus since this virus transmits only by cell-to-cell infection [5–7]. The infectivity of free HTLV-1 virions is very inefficient whereas this virus transmits efficiently through cell-to-cell contact [8, 9]. Therefore, HTLV-1 induces proliferation of infected cells to increase the chance of transmission [10–12]. There are two different ways to increase the number of HTLV-1-infected cells *in vivo*: proliferation of infected cells (mitotic division) and *de novo* infection [7]. It is thought that mitotic division is predominant in the chronic infection of this virus.

HTLV-1 is a member of the primate T-cell leukemia virus type 1 (PTLV-1) group, which contains simian T-cell leukemia virus type 1 (STLV-1) [13]. Based on phylogenetic analyses, HTLV-1 is thought to be derived from STLV-1 by interspecies transmission [14]. Old World monkeys are infected with STLV-1 while New World monkeys are not infected [15]. It was reported that the seroprevalence of STLV-1 in Japanese macaques (JMs) was high [16]. We have reported that STLV-1 induces clonal proliferation of CD4^+^ T cells *in vivo*, and development of T-cell lymphoma was observed in a STLV-1 infected JM [17]. STLV-1 encodes Tax in the plus strand and STLV-1 bZIP factor (SBZ) in the minus strand. STLV-1 Tax and SBZ possess similar functions to HTLV-1 Tax and HTLV-1 bZIP factor (HBZ). Therefore, the STLV-1 infected JM is a good model for HTLV-1 infection [17]. However, the frequency of expression of viral genes in various organs and tissues *in vivo* remains unknown.

The receptors for HTLV-1 are glucose transporter 1 (GLUT-1) and neuropilin 1, which are expressed on various types of cells [18]. Therefore, this virus can infect different types of cells *in vitro* [19–22]. However, the HTLV-1 provirus is mainly detected in CD4^+^ T cells, in particular, CADM1^+^CCR4^+^CD45RO^+^ T cells *in vivo* [23–26]. This suggests that HTLV-1 either modulates the immunophenotype of T cells, or preferentially infects this subpopulation. Since the cellular receptors of this virus do not absolutely define the specificity of target cells, it is possible that hematopoietic stem cells (HSCs) are also infected by HTLV-1. Although Tax expressing cells were found in the bone marrow [27], previous studies reported that HTLV-1 did not infect HSCs in ATL patients [28, 29].

In this study, we analyzed expression of *tax* and *SBZ* genes in various organs and tissues of STLV-1 infected JMs, and found that expression of *SBZ* were higher than those of the *tax* gene, while the *tax* gene was highly expressed in the peripheral blood and bone marrow, suggesting that infectious cycle replication of STLV-1 occurs in the bone marrow. To explore the possibility that HTLV-1 infects HSCs, we analyzed integration sites of HTLV-1 in the different hematopoietic cells. The same integration sites of HTLV-1 proviruses were detected in neutrophils, monocytes, B cells, CD8^+^ T cells and CD4^+^ T cells in HAM/TSP patients, suggesting that HTLV-1 infects HSCs. This study uncovers a new aspect of HTLV-1 infection and spread *in vivo*.

## Results

### Expression of viral genes in various organs and tissues

**Fig 1:**
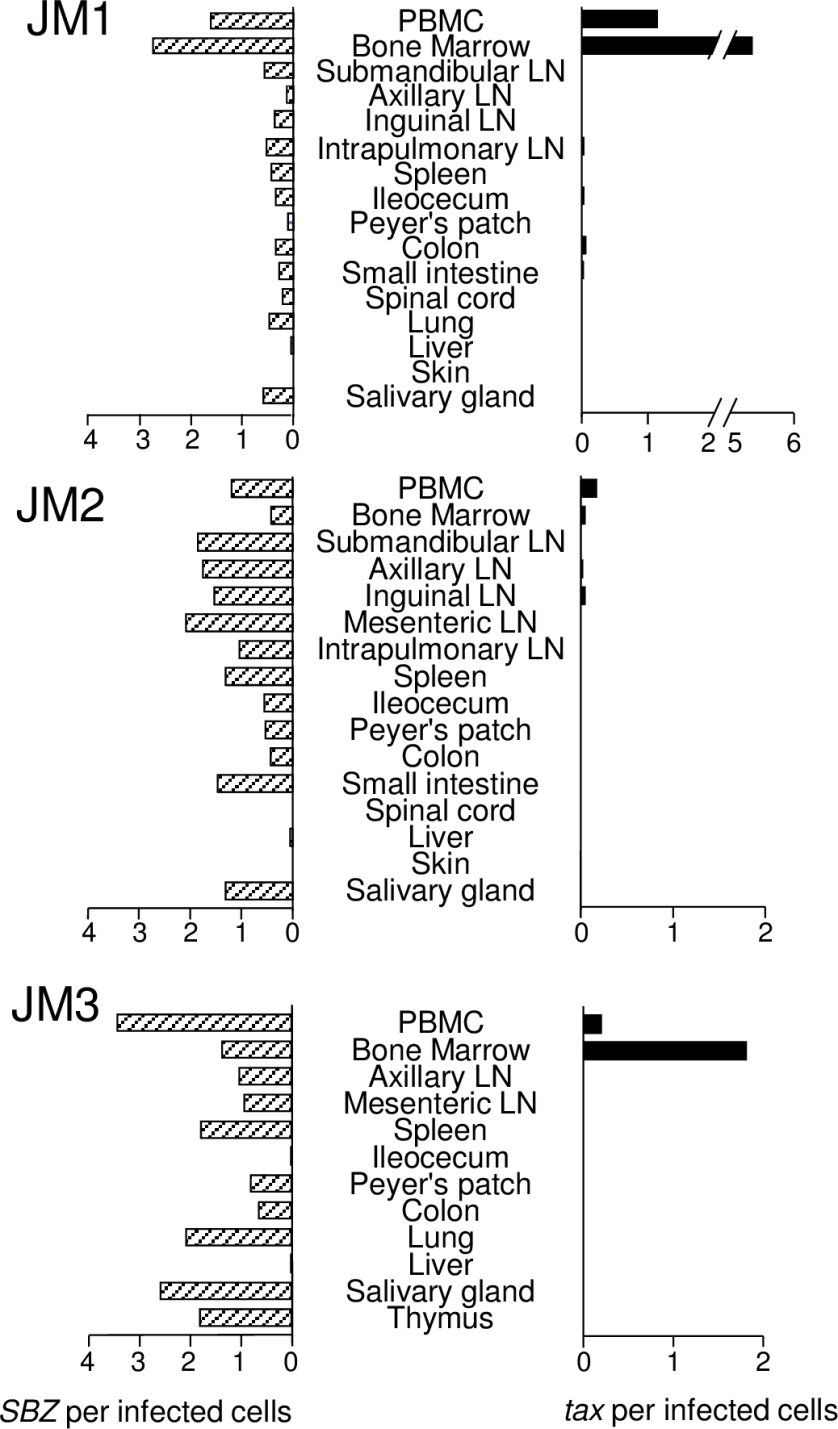
Tax and SBZ expression in STLV-1 naturally infected monkeys. *SBZ* and *tax* expression level in various tissues and organs of STLV-1 naturally infected Japanese macaques (JM1-3) was measured by quantitative PCR. The relative expression level per provirus was presented.

To explore *in vivo* viral gene expression of virus-infected cells, we analyzed the proviral loads (PVLs) and transcripts of the *tax* and *SBZ* genes in STLV-1-infected JMs. To reduce contamination of peripheral blood lymphocytes in organs and tissues, three monkeys were perfused (JM1, JM2 and JM3). PVL was presented as the percentage of infected cells in total cells. The limit of detection was 100 copies per sample as described in Materials and Methods. The PVL varied between tissues and organs although it was high in peripheral blood, lymph nodes and spleen (S1 Table), indicating that infected cells are abundant in lymphatic tissues and lymphocytes. Next, we quantified *tax* and *SBZ* transcripts per provirus of these tissues and organs. The expression levels of *tax* and *SBZ* were measured by real-time PCR with the ddCt algorithm using an STLV-1 infected cell line, Si-2, as a reference. In order to compare the expression of *tax* and *SBZ* in each sample, the absolute amount of them in Si-2 was determined by the standard curve method, and the expression values in all samples were normalized to the expression in Si-2 cells. Details of this calculation are described in Materials and Methods. In general, the level of *SBZ* expression was much higher than that of *tax*. *SBZ* was expressed in most tissues and organs, although the level of expression was variable (Fig 1). However, *tax* transcripts were detected in very limited tissues and organs. In particular, the *tax* transcript was highly expressed in peripheral blood and bone marrow.

### Multi-lineage hematopoietic cells are infected by HTLV-1

**Fig 2:**
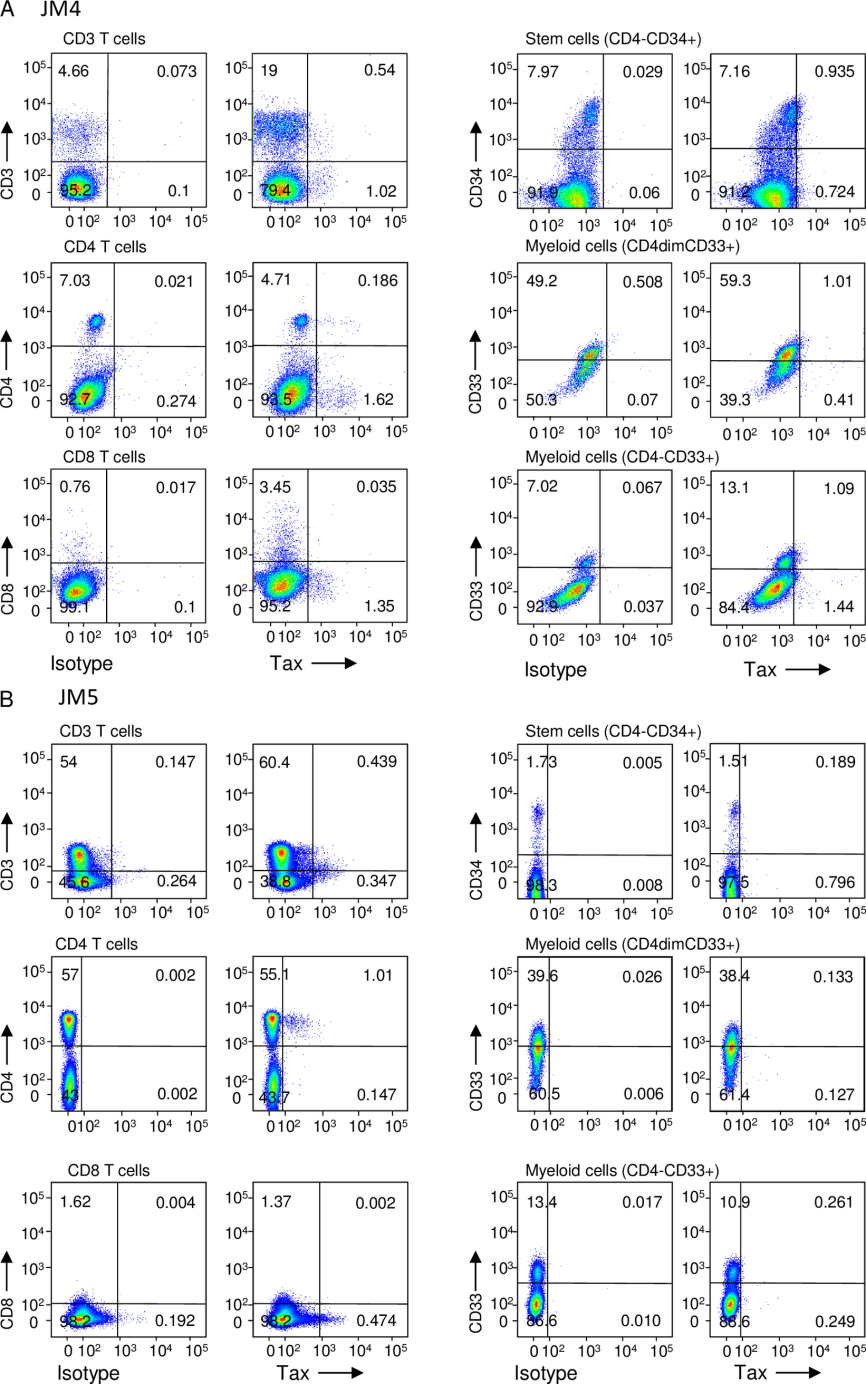
HTLV-1 infection in multiple lineage hematopoietic cells. Bone marrow cells from two STLV-1 infected JMs were cultured for 24 hours after removal of CD8^+^ T cells. (A, B) Tax expression in bone marrow cells of two STLV-1 infected JMs. Mononuclear cells from bone marrow were stained by various antibodies. Tax expression was shown in association with CD3, CD4, CD8, CD34 and CD33 expression.

It has been reported that Tax-expressing cells were abundant in the bone marrow of HAM/TSP patients [27]. Likewise, this study showed that higher Tax expression was found in the bone marrow cells of STLV-1-infected JMs (Fig 1). Since Tax is essential for viral replication and transmission, the presence of Tax-expressing cells suggests that *de novo* infection of HSCs with HTLV-1 occurs in the bone marrow. To address this question, we analyzed Tax expression in bone marrow cells of two STLV-1-infected JMs (JM4, 5) and an uninfected JM (JM6). Twenty-four hours after removal of CD8^+^ T cells from the bone marrow cells, Tax expression was measured by flow cytometry. As shown in Fig 2, both CD3^+^ and CD3^-^ bone marrow cells expressed Tax. Tax positive cells were also found in CD4^-^ or CD8^-^ cells. These data indicate that non-T cells are infected by STLV-1. On the other hand, Tax expression was not detected in a non-infected monkey (JM6: S2 Fig). Further analyses suggested that stem cells (CD4^-^CD34^+^), myeloid cells (CD4^dim^CD33^+^ or CD4^-^CD33^+^) and B cells (CD4^-^CD19^+^) express Tax *in vivo* (Fig 2 and S2 Fig). These data indicate the possibility that not only T cells but also non-T cells are infected by STLV-1 in bone marrow.

In view of the similarity between STLV-1 and HTLV-1, we speculated that HTLV-1 also infects hematopoietic precursor cells in bone marrow. To test this possibility, we analyzed the genomic integration sites of HTLV-1 in peripheral blood mononuclear cells (PBMCs) and neutrophils of a HAM/TSP patient (HAM/TSP#1) using high-throughput sequencing. Contamination of infected T cells is a serious problem to identify the integration sites of provirus in various hematopoietic cells. Since neutrophil is abundantly present in the peripheral blood, the level of T-cell contamination is low. Contamination of lymphocytes in isolated neutrophils was morphologically confirmed, and their percentages were 0.2–0.9% (0.92% for HAM/TSP#1, 0.20% for HAM/TSP#2, and 0.26% for HAM/TSP#3).

We observed certain integration sites in both PBMCs and neutrophils (HAM/TSP#1)(S2 Table), suggesting that HTLV-1 infects HSCs *in vivo*. However, since most proviruses are present in T cells *in vivo*, the risk of contamination of T cells cannot be excluded in this experiment. To examine further the possibility of HTLV-1 infection in HSCs, we isolated various lineages of hematopoietic cells (CD4^+^ T cells, CD8^+^ T cells, B cells, monocytes, and neutrophils) from two HAM/TSP patients (HAM/TSP#2 and #3) and a HTLV-1 carrier, and then analyzed integration sites of the HTLV-1 provirus in each lineage (S3 Table). The PVLs of HTLV-1 in each of these lineages are shown in S4 Table. To avoid the contamination of detected sequences, we analyzed each sample using the Ion PGM machine on a separate chip. The observation that a given proviral integration site is present at a higher abundance in non-T cell lineages than in CD4^+^ or CD8^+^ T cells, argues against the possibility of T-cell contamination. Further, the presence of such integration sites in different cell lineage suggests that HTLV-1 infects HSCs. Tables 1–3 show the 15 most abundant clones in each cell lineage from two HAM/TSP patients and a HTLV-1 carrier. We repeatedly identified identical integration sites in different cell types, suggesting that HTLV-1 infects HSCs in the bone marrow and subsequently differentiate *in vivo*. The purity of each cell type was not perfect, raising the question whether certain detected integration sites were derived from contaminating T cells. However, certain integration sites that were frequently observed in neutrophils, B cells or monocytes were rarely detected in CD4^+^ T cells. Conversely, certain integration sites observed in high-abundance CD4^+^ T cell clones were not detected in cells of other lineages (Tables 1, 2 and 3). These data suggest that in non-T cells and HSCs are infected with HTLV-1. All integration site data in all lineage cells are summarized in S5 Table.

**Table 1:** Top 15 clones of each cell lineage in HAM/TSP#2.

CD4 T cells^2^						B cells^2^						Neutrophils^2^					
ID^1^	CD4^2^	CD8^3^	B^3^	Mo^3^	Neu^3^	ID^1^	CD4^3^	CD8^3^	B^2^	Mo^3^	Neu^3^	ID^1^	CD4^3^	CD8^3^	B^3^	Mo^3^	Neu^2^
3492	141	23	19	7	6	3492	141	23	19	7	6	4708^4^	2	9	11	11	14
4497	47	1	0	0	5	5028^4^	3	0	15	0	0	5202	1	14	11	30	12
4025	46	0	0	0	2	4708	2	9	11	11	14	5686^4^	5	0	0	0	9
4799	41	2	0	0	0	5202	1	14	11	30	12	4460^4^	3	0	0	0	9
5133	40	1	0	0	0	5143^4^	4	0	8	1	0	5033^4^	3	0	0	0	7
5831	39	1	0	0	3	3306	16	0	7	0	5	3503^4^	0	4	4	6	7
5165	39	1	0	0	0	3432	12	0	7	0	0	4675	0	0	0	0	7
4375	36	0	0	0	0	3635	0	0	7	0	0	3492	141	23	19	7	6
3996	31	0	0	0	0	4861	25	1	6	0	0	4360	0	0	0	0	6
3113	30	0	0	0	0	5160	0	28	6	0	0	4497	47	1	0	0	5
3669	27	0	1	0	2	3267	0	0	6	0	0	3306	16	0	7	0	5
3147	27	0	0	0	0	5895	0	0	5	0	0	5786	10	0	0	0	5
5974	26	0	0	0	1	3503	0	4	4	6	7	4422^4^	4	1	0	0	5
5595	26	0	0	0	0	4570	0	0	4	0	0	3063	5	0	0	0	4
4861	25	1	6	0	0	3754	1	3	2	1	3	3946^4^	1	0	0	3	4
CD8 T cells^2^						Monocytes^2^					
ID^1^	CD4^3^	CD8^2^	B^3^	Mo^3^	Neu^3^	ID^1^	CD4^3^	CD8^3^	B^3^	Mo^2^	Neu^3^
5891	0	119	1	2	0	5328	0	0	0	47	0
5818	0	73	1	0	0	5202^4^	1	14	11	30	12
4433	2	70	0	0	0	4300	0	0	0	14	0
3169	0	50	0	0	0	5542^4^	10	0	0	13	1
4703	0	40	0	0	0	4708	2	9	11	11	14
5160	0	28	6	0	0	3210	0	0	0	8	0
5917	0	25	0	0	0	3492	141	23	19	7	6
4285	0	25	0	0	0	3503	0	4	4	6	7
3130	0	24	0	0	0	4336	0	0	0	5	0
3492	141	23	19	7	6	3680^4^	2	0	0	4	0
3037	0	20	0	0	0	3946	1	0	0	3	4
5923	0	20	0	0	0	3290	8	0	0	3	0
3598	0	19	0	0	0	3489	0	0	0	3	0
5675	0	17	0	0	0	3751	0	0	0	3	0
4147	0	17	0	0	0	4082	0	0	0	3	0

CD4: CD4 T cell; CD8: CD8 T cell; B: B cell; Mo: Monocyte; Neu: Neutrophil

^1^ = The ID of clones was allocated by integration sites

^2^ = Top 15 high abundance clones in each cell lineage were shown. Each number indicates the abundance of clones. Top 15 high abundance clones in each cell type was shown in color (CD4 T cells, sky blue: CD8 T cells, pink: B cells, green: monocytes, yellow: neutrophils, brown).

^3^ = To show whether the same integration sites are detected in other types of cells, the abundance of each clone in other cells was shown in the same rows.

^4^ = These clones were detected in other types of cells, but the abundance was lower in other cells, indicating that this is not due to contamination of infected T cells.

**Table 2:** Top 15 clones of each cell lineage in HAM/TSP#3.

CD4 T cells^2^						B cells^2^						Neutrophils^2^					
ID^1^	CD4^2^	CD8^3^	B^3^	Mo^3^	Neu^3^	ID^1^	CD4^3^	CD8^3^	B^2^	Mo^3^	Neu^3^	ID^1^	CD4^3^	CD8^3^	B^3^	Mo^3^	Neu^2^
8603	106	0	0	8	0	8196	24	0	11	0	0	8033	59	0	0	0	12
6533	83	4	0	0	0	7175^4^	6	0	9	0	0	7883	21	0	0	0	11
7370	78	0	0	1	1	8659	0	83	7	1	0	7693	55	7	0	18	6
7566	77	0	0	15	0	7968	51	0	7	0	0	7751	38	0	0	0	4
8392	69	0	0	6	0	7511	39	0	5	0	0	7855	5	0	0	0	3
7999	64	0	0	0	0	6349^4^	1	0	5	0	0	7160	26	0	0	6	2
8033	59	0	0	0	12	6330	22	0	4	0	0	6598	0	0	0	0	2
8139	57	0	0	0	0	7827	8	0	3	0	0	7370	78	0	0	1	1
7693	55	7	0	18	6	6883	0	0	3	0	0	6250	22	0	0	0	1
8488	53	0	0	0	0	7177	14	0	2	0	0	8148	40	0	0	0	1
6706	53	0	0	0	0	6228	19	0	1	0	0	6154	0	0	0	0	1
7968	51	0	7	0	0	8282	1	0	1	0	0	8090	0	0	0	0	1
7515	50	0	0	0	0	6395	42	0	1	0	0	6472	0	0	0	0	1
8125	49	0	0	0	0	7231	12	0	1	0	0	8668	0	0	0	0	1
8583	48	0	0	0	0	8626	17	0	1	0	0
CD8 T cells^2^						Monocytes^2^					
ID^1^	CD4^3^	CD8^2^	B^3^	Mo^3^	Neu^3^	ID^1^	CD4^3^	CD8^3^	B^3^	Mo^2^	Neu^3^
7266	9	143	0	0	0	7693	55	7	0	18	6
8659	0	83	7	1	0	7050	17	16	0	15	0
6500	0	83	0	0	0	7566	77	0	0	15	0
7820	0	78	0	0	0	6728	21	0	0	15	0
7451	0	63	0	0	0	7682	14	0	0	14	0
7715	0	54	0	0	0	8008^4^	6	0	0	14	0
8210	0	46	0	0	0	6052^4^	11	0	0	13	0
8105	0	44	0	0	0	6420	0	0	0	13	0
6999	0	43	0	0	0	6303^4^	7	0	0	12	0
6686	0	38	0	0	0	8536^4^	8	0	0	11	0
6409	0	36	0	0	0	7597	20	0	0	10	0
7010	0	35	0	0	0	7956	0	20	0	10	0
7260	0	34	0	0	0	7133	0	0	0	10	0
8610	0	32	0	0	0	8457	27	0	0	9	0
6162	0	28	0	0	0	6905^4^	5	0	0	9	0

CD4: CD4 T cell; CD8: CD8 T cell; B: B cell; Mo: Monocyte; Neu: Neutrophil

^1^ = The ID of clones was allocated by integration sites

^2^ = Top 15 high abundance clones in each cell lineage were shown. Each number indicates the abundance of clones. Top 15 high abundance clones in each cell type was shown in color (CD4 T cells, sky blue: CD8 T cells, pink: B cells, green: monocytes, yellow: neutrophils, brown).

^3^ = To show whether the same integration sites are detected in other types of cells, the abundance of each clone in other cells was shown in the same rows.

^4^ = These clones were detected in other types of cells, but the abundance was lower in other cells, indicating that this is not due to contamination of infected T cells.

**Table 3:** Top 15 clones of each cell lineage in a HTLV-1 carrier.

CD4 T cells^2^						B cells^2^						Neutrophils^2^					
ID^1^	CD4^2^	CD8^3^	B^3^	Mo^3^	Neu^3^	ID^1^	CD4^3^	CD8^3^	B^2^	Mo^3^	Neu^3^	ID^1^	CD4^3^	CD8^3^	B^3^	Mo^3^	Neu^2^
11921	159	0	0	5	0	11070^4^	0	26	57	0	0	10660	5	178	37	40	43
10549	140	3	0	24	6	10660	5	178	37	40	43	10359^4^	8	0	0	7	21
11565	101	4	0	6	3	9170	0	62	27	7	0	10427	65	0	0	0	13
10772	97	0	0	0	10	9295^4^	0	10	24	0	0	11461^4^	6	0	0	0	13
9042	88	0	0	0	0	9503	0	52	23	4	0	11959	14	0	0	11	10
11265	80	0	0	14	1	9441^4^	0	8	16	0	0	10772	97	0	0	0	10
12107	80	0	0	18	8	11833	0	9	8	0	0	11415	0	0	0	0	10
11026	72	2	0	6	7	11602	51	0	8	13	0	11692	0	0	0	0	10
9476	71	0	0	10	0	10914	0	40	7	0	0	11106	45	0	0	0	9
9274	69	0	0	11	0	9068	0	73	6	0	0	10201	26	0	0	0	9
9447	69	0	0	0	0	11854	1	6	5	0	0	11252^4^	3	0	0	0	9
9207	66	0	0	12	4	11253	0	0	5	0	0	12107	80	0	0	18	8
10427	65	0	0	0	13	8863	6	117	4	11	2	9908	9	0	0	0	8
10174	63	0	0	9	5	10375	0	8	4	0	0	12117	50	1	0	7	7
9206	54	0	0	5	0	10016	0	113	3	4	1	11026	72	2	0	6	7
CD8 T cells^2^						Monocytes^2^					
ID^1^	CD4^3^	CD8^2^	B^3^	Mo^3^	Neu^3^	ID^1^	CD4^3^	CD8^3^	B^3^	Mo^2^	Neu^3^
10660	5	178	37	40	43	10660	5	178	37	40	43
8863	6	117	4	11	2	10549	140	3	0	24	6
10016	0	113	3	4	1	11992^4^	11	5	0	20	0
8901	0	83	2	3	0	10488	0	0	0	20	0
9068	0	73	6	0	0	12107	80	0	0	18	8
9170	0	62	27	7	0	11042^4^	1	0	0	17	0
9435	0	61	3	1	0	11876	54	0	0	16	2
9503	0	52	23	4	0	11265	80	0	0	14	1
9366	1	51	0	0	0	9664	0	0	0	14	0
12127	0	47	3	0	0	11602	51	0	8	13	0
9555	0	41	1	0	0	11616	15	0	0	13	3
10741	0	40	0	0	0	9207	66	0	0	12	4
10914	0	40	7	0	0	11524	42	0	0	12	0
10205	0	31	0	0	0	11198	36	0	0	12	0
10376	0	29	0	0	0	10134^4^	11	0	0	12	0

CD4: CD4 T cell; CD8: CD8 T cell; B: B cell; Mo: Monocyte; Neu: Neutrophil

^1^ = The ID of clones was allocated by integration sites

^2^ = Top 15 high abundance clones in each cell lineage were shown. Each number indicates the abundance of clones. Top 15 high abundance clones in each cell type was shown in color (CD4 T cells, sky blue: CD8 T cells, pink: B cells, green: monocytes, yellow: neutrophils, brown).

^3^ = To show whether the same integration sites are detected in other types of cells, the abundance of each clone in other cells was shown in the same rows.

^4^ = These clones were detected in other types of cells, but the abundance was lower in other cells, indicating that this is not due to contamination of infected T cells.

**Fig 3:**
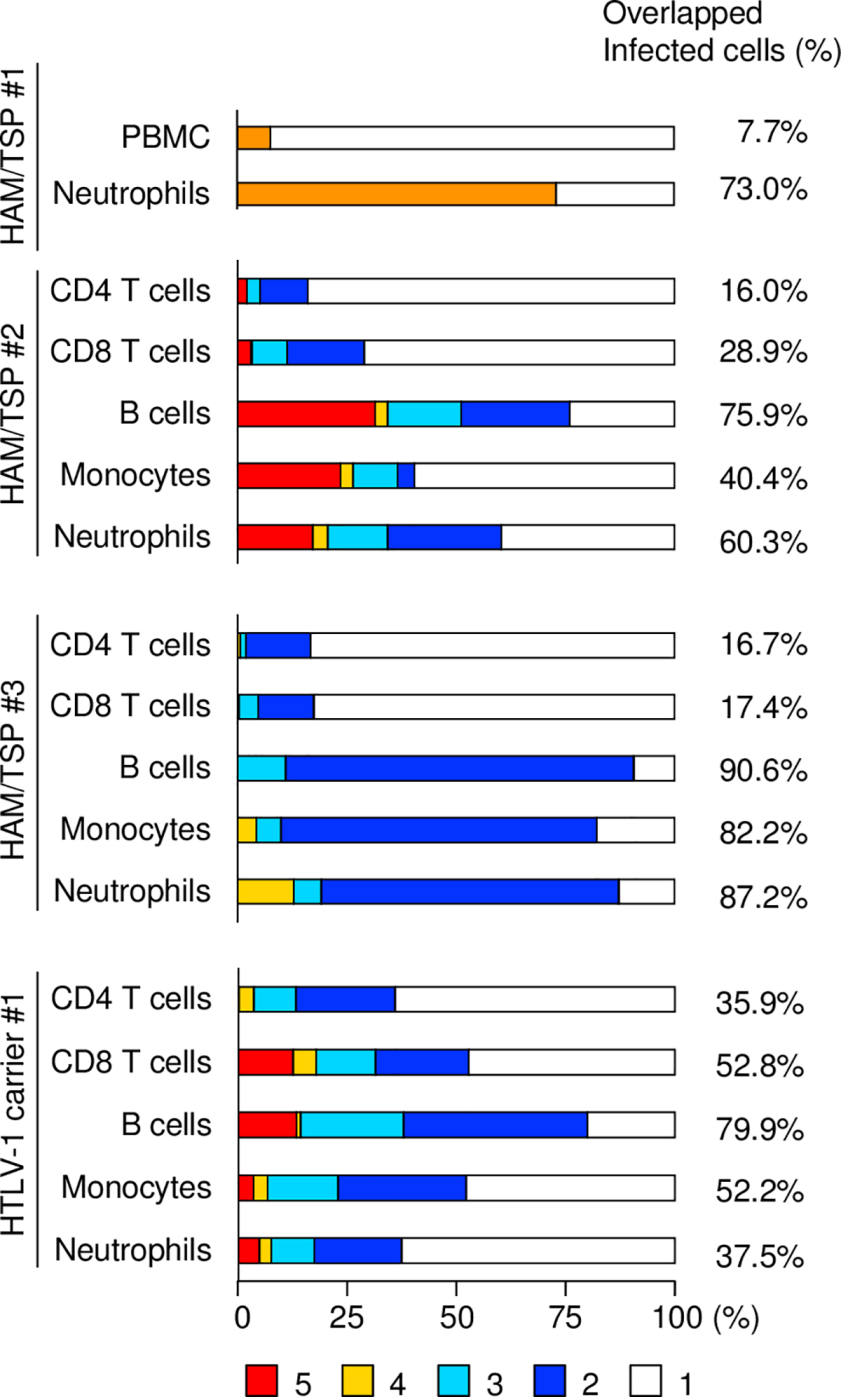
The proportions of clones with the same integration sites. The proportions of clones with the same integration sites are shown. The same integration sites are identified in 5 (red), 4 (yellow), 3 (light blue), and 2 (blue) different lineage cells. For HAM/TSP1, the proportion of clones with the same integration sites between PBMCs and neutrophils are shown in orange.

Next, we analyzed the proportion of HTLV-1 infected cells that share integration sites with other lineage cells and are derived from infected HSCs. The percentages of infected cells with the same integration sites with other lineage cells were generally high in neutrophils, monocytes and B cells (Fig 3), suggesting that these cells were infected as precursor cells in the bone marrow. A substantial number of CD4^+^ T cells (16.0% for HAM/TSP#2, 16.7% for HAM/TSP#3 and 35.9% for a HTLV-1 carrier) possessed integration sites observed in other hematopoietic cells. This indicates that some HTLV-1 infected CD4^+^ T cells are derived from infected HSCs.

### HTLV-1 infection in neutrophils and monocytes

**Fig 4:**
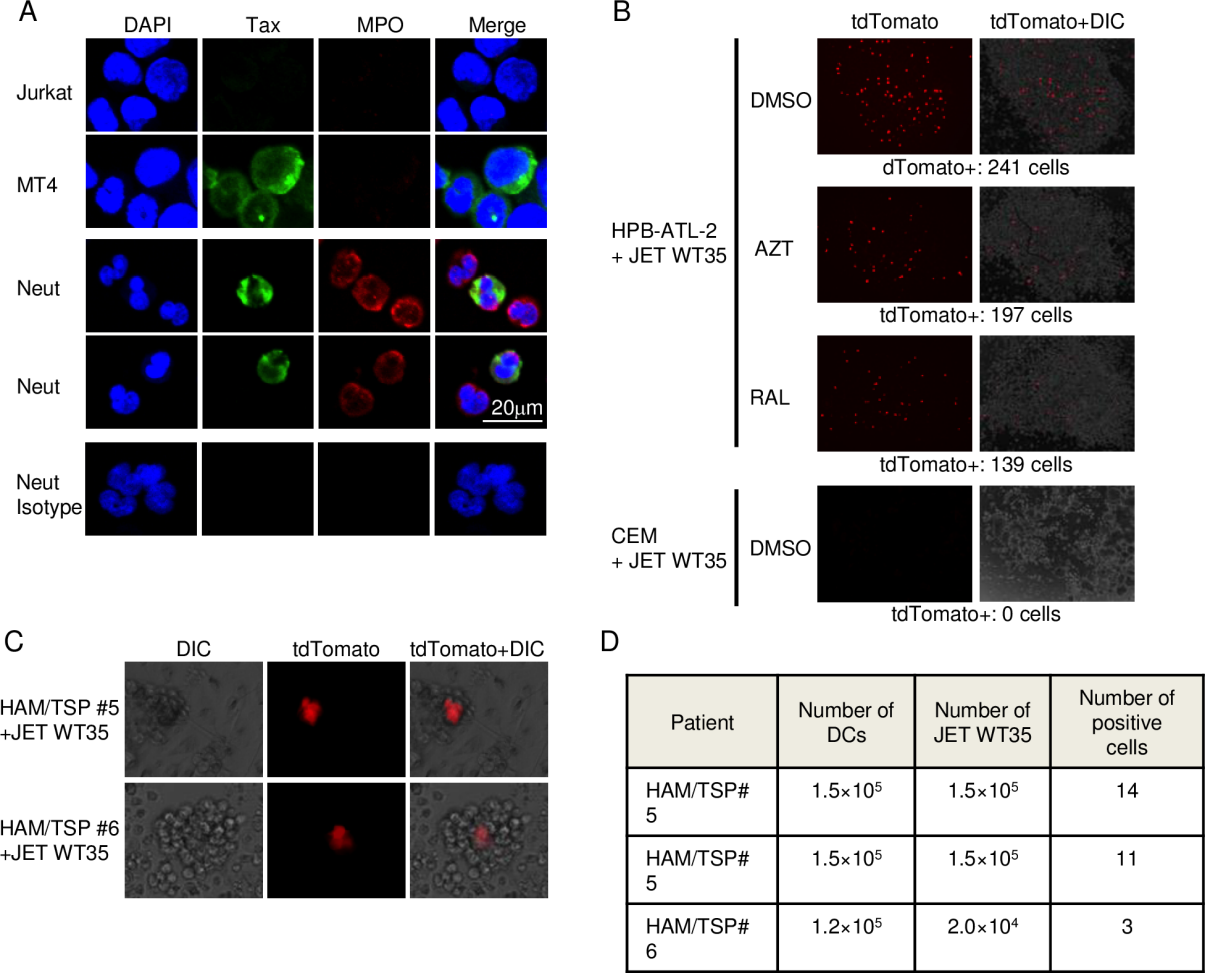
HTLV-1 infection in neutrophils and monocytes. (A) Tax expression is detected by confocal immunofluorescence microscopy in MT4 and neutrophils from HAM/TSP patients using anti-Tax antibody and secondary Alexa Fluor 488 antibody. Myeloperoxidase expression is also detected in neutrophils using anti-MPO antibody and secondary Alexa Fluor 568 antibody. Jurkat was used as a negative control. MT4, HTLV-1 infected cell line; Jurkat cells, HTLV-1 negative human T-cell line. DAPI (blue) was used for nuclei staining. (B) JET WT35 cells were co-cultured with HPB-ATL-2 cells in the presence and absence of azidothymidine (AZT) or raltegravir (RAL), or HTLV-1 uninfected T cell line, CCRF-CEM. The number of tdTomato-positive cells is presented. (C) Dendritic cells were induced from monocytes from HAM/TSP patients, and then differentiated DCs were co-cultured with JET WT35. After 48 hours, newly infected JET WT35 was shown to be red. (D) Quantitative data of co-culture between differentiated DCs and JET WT35 cells. The count of tdTomato positive cells and the number of cells used for this experiment are shown.

These data indicate that HTLV-1 infects HSCs *in vivo*. To confirm the presence of HTLV-1 infection in neutrophils, we tried to detect Tax protein in neutrophils using immunofluorescent staining. Tax protein was detected in the neutrophils from HAM/TSP patients along with myeloperoxidase (Fig 4A), which confirmed HTLV-1 infection of neutrophils.

It has been reported that HTLV-1 infected DCs spread virus to T cells via a virological synapse [30]. When the monocytes that are infected in the bone marrow differentiate *in vivo*, infected DCs may subsequently disseminate the virus. To check this possibility, we differentiated monocytes from HAM/TSP patients to DCs using GM-CSF and IL-4 in the presence of azidothymidine (AZT), and the differentiated DCs were then co-cultured with Jurkat cells stably transfected with plasmid that encodes the tandem dimer Tomato (tdTomato) under the control of the Tax responsive element (JET WT35). Differentiation to DCs was confirmed by expression of CD11c and CD209, and loss of CD14 expression (S3 Fig). Treatment by AZT or raltegravir partially reduced tdTomato positive cells of JET WT35 co-cultured with an ATL cell line, HPB-ATL-2, indicating that JET WT35 could detect both *de novo* infection and Tax expression (via soluble Tax or cell fusion) (Fig 4B). As shown in Fig 4C, tdTomato positive JET WT35 cells showed *de novo* infection or Tax production from the differentiated DCs. The quantitative data was shown in Fig 4D. These data suggest that DCs derived from HTLV-1-infected monocytes facilitate *in vivo* infection of this virus.

### Persistent HTLV-1 infection in hematopoietic stem cells

**Fig 5:**
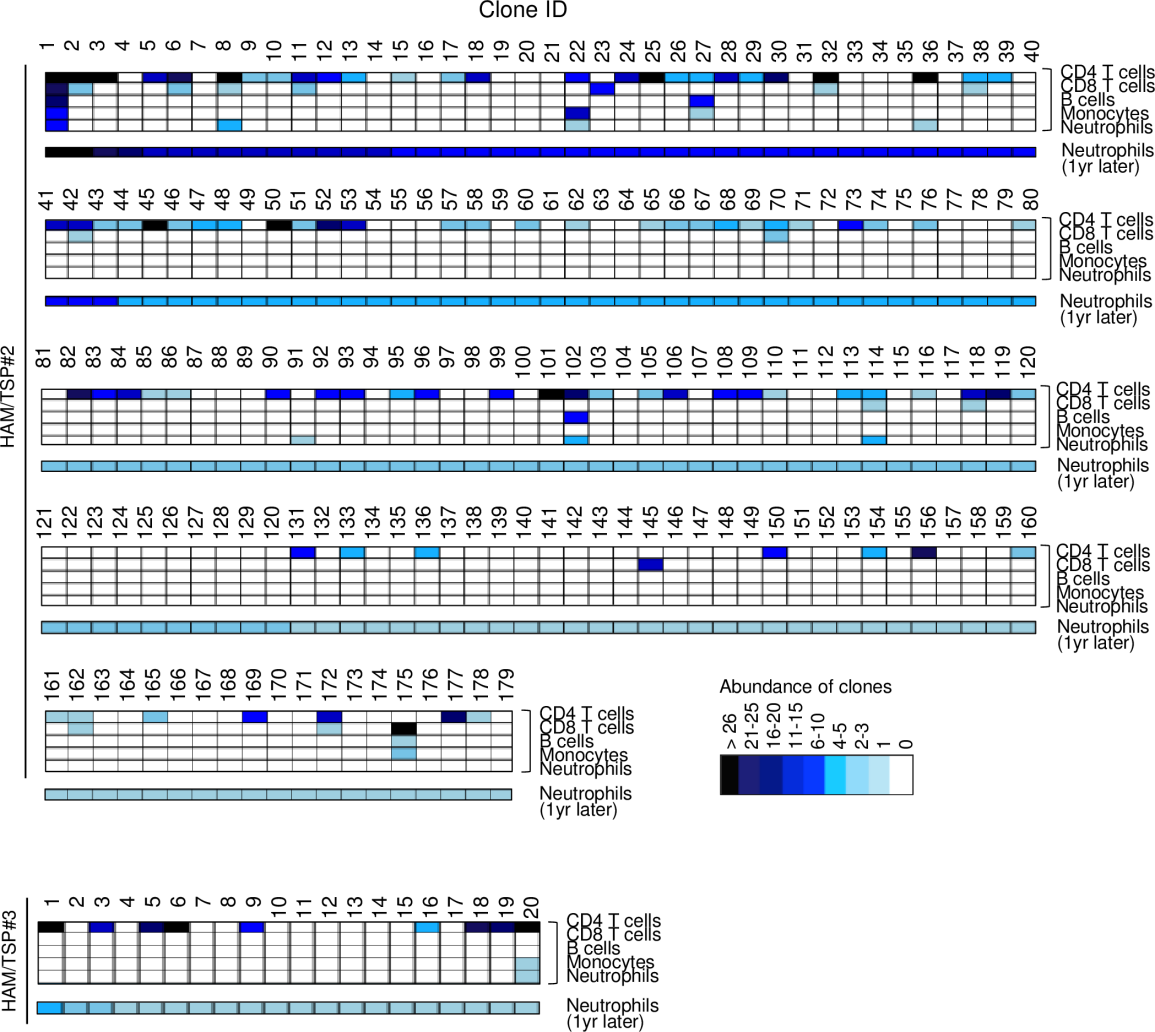
Persistence of HTLV-1 infected clones in HAM/TSP patients at different time point. HTLV-1 integration sites are determined using high throughput sequencing using genomic DNAs from neutrophils one year later in two HAM/TSP patients. The cell number of each clone is demonstrated by color. The longitudinal column represents the same integration site.

The next question is whether HTLV-1-infected HSCs persist *in vivo*. To answer this question, we analyzed HTLV-1 integration sites in neutrophils at different time points in the same HAM/TSP patients. Neutrophils with the same integration site as observed in other hematopoietic cells were detected after one year in two HAM/TSP patients (HAM/TSP#2 and HAM/TSP#3)(Fig 5). Since half-life of neutrophil in blood is about seven hours [31], this data suggests that HTLV-1-infected HSCs can survive and generate infected neutrophils *in vivo*. Data on the integration sites is summarized in S6 Table. Interestingly, approximately half of the clones detected in neutrophils were present at the first analysis (96 of 179 clones (54%) in HAM/TSP#2, and 9 of 20 clones (45%) in HAM/TSP#3), again suggesting that HTLV-1-infected clones can persist *in vivo*.

## Discussion

It has been reported that HTLV-1 can infect various types of cells *in vitro* [19–22]. Furthermore, the provirus was detected in various hematopoietic cells *in vivo* [32]. However, it remains uncertain whether HTLV-1 infects HSCs *in vivo*. It was thought that HTLV-1 infects mature lymphocytes, macrophages and dendritic cells in the periphery. Indeed, previous studies reported that HSCs were not infected by HTLV-1 [28, 29]. In these studies, HSCs were isolated from patients with ATL, in which most of the HTLV-1-infected cells were leukemic cells that frequently do not express Tax [3]. On the other hand, Tax was relatively highly expressed in peripheral blood of HAM/TSP patients, and Tax-expressing cells were also found in the bone marrow of HAM/TSP patients [27, 33]. These observations raise the possibility that bone marrow is a reservoir of HTLV-1 [34]. It has been reported that CD4^+^ memory T cells specific for cytomegalovirus, tetanus toxoid, measles, mumps and rubella are enriched in the bone marrow [35]. It is possible that such memory T cells are infected by HTLV-1 and express Tax in the bone marrow. Tax is an essential protein for HTLV-1 replication, and the observed Tax expression suggests that *de novo* infection occurs in the bone marrow in HAM/TSP patients. Indeed, we first presented the evidence that HTLV-1-infected hematopoietic cells of different lineages have the same integration sites *in vivo*, indicating that this virus infects HSCs.

It is critical to show that these commonly identified integration sites are not derived from contamination with HTLV-1-infected lymphocytes during separation of each cells. It is almost impossible to completely exclude contamination of lymphocytes from isolated cells in every case due to predominance of CD4^+^ T cells in infected cells. However, it is unlikely that all of the integration sites identified in different cell lineages were derived from contaminated cells, for the following reasons. First, approximately 90% of HTLV-1 provirus is detected in CD4^+^ T cells [23]. Therefore, the contaminating cells are likely to be CD4^+^ T cell clones with high abundance. However, the abundance of a given integration site in non-CD4^+^ T cells was frequently higher than that in CD4^+^ T cells (Tables 1–3). Second, some integration sites were identified only in non-T cells (in HAM/TSP#2, clone ID 5328, 4300, 3210, 4336, 3489, 3751, and 4082 in monocytes; ID 4675 and 4360 in neutrophils: in HAM/TSP#3, clone ID 6420 and 7133 in monocytes; 6598 in neutrophils: in a carrier, clone 11253 in B cells; ID 10488, 9664 in monocytes; ID 11415, and 11692 in neutrophils)(Tables 1–3). These data indicate that HTLV-1 infects HSCs *in vivo*. However, the percentage of infected cells derived from infected HSCs might be over or underestimated. As shown in S6 Table, identified integration sites in neutrophil were frequently found only in CD4^+^ T cells one year ago, suggesting that frequency of infected cells derived from infected HSCs is underestimated. At the same time, contamination of infected CD4^+^ T cells might cause overestimation of this frequency especially when only a cell with the integration site was found in non-T cell lineage.

Is HTLV-1 infection of HSCs beneficial for this virus? Viral transmission needs expression of viral antigens (Env, Gag, Pol, Tax, and Rex) to form viral particles. Therefore, cytotoxic T lymphocytes tend to attack infected cells during transmission. In this regard, HTLV-1-infected cells that differentiate from HSCs can reduce necessity to express viral antigens *in vivo*. It might be a strategy of HTLV-1 to decrease viral replication, in order to avoid immune attack by the host. After transmission via breast-feeding or sexual intercourse, it is speculated that HTLV-1 infects many T cells as shown in bovine leukemia virus infected cow [36]. During this stage, some infected T cells might migrate into the bone marrow. It is possible that hypoxic condition in the bone marrow enables infected T cells to express Tax, which causes *de novo* infection [37]. This scenario should be analyzed in the future studies.

It is intriguing that a fraction of infected CD4^+^ T cells appear to be derived from infected HSCs, suggesting that infected pre-T cells in the bone marrow migrate to the thymus and differentiate to CD4^+^ and CD8^+^ T cells. It has been well recognized that HTLV-1-infected cells and ATL cells possess specific surface markers including CD4, CD25, CCR4, and CADM1 [24–26, 38, 39]. There are two possible scenarios. First, HTLV-1 targets this specific subpopulation. Secondly, viral proteins modulate phenotypes of infected cells. Our finding that HTLV-1 infected HSCs can differentiate to mature CD4^+^ T cells *in vivo* suggests that viral proteins convert infected cells to cells with specific markers, which supports the second hypothesis. It has been reported that HBZ induces expression of Foxp3 while Tax suppresses its expression [40, 41]. Recently, we have reported that HBZ induces expression of CCR4, T cell immunoglobulin and ITIM domain (TIGIT), and PD-1, which are expressed on ATL cells and HTLV-1-infected cells [42]. Thus, HBZ is considered to control the immunophenotype of infected cells and ATL cells during differentiation from HSC.

Analyses of integration sites at different time points demonstrated that identical integration sites were frequently detected in neutrophils and other lineage cells (Fig 5), indicating that HTLV-1 infected HSCs can persist *in vivo* for at least one year. It is noteworthy that approximately half the observed integration sites in neutrophils were detected in other lineage cells one year earlier. These findings suggest that most of infected HSCs persist *in vivo*. It has been reported that the risk of cancer is influenced by the number of stem cell divisions [43]. If HTLV-1-infected HSCs survive for a long time, persistent HTLV-1 infection in HSCs might predispose to leukemogenesis by HTLV-1.

It has been shown that HTLV-1 infects DCs, which likely transmits viruses to T cells [21, 30, 44]. This study reveals that at least, some HTLV-1 infected monocytes are derived from infected HSCs *in vivo*. It is thought that DCs derived from infected monocytes efficiently transmit virus to T cells through virological synapses formed between DCs and T cells. *De novo* infection requires expression of Tax and other viral proteins. However, infected monocytes derived from HSCs do not need to express viral proteins until transmission occurs at the periphery. This strategy might therefore enable infected cells to evade the host immune responses *in vivo*.

In this study, we demonstrate that HTLV-1 infects HSCs, which then differentiate to multiple lineage hematopoietic cells *in vivo*. This study suggests that HTLV-1-infected HSCs form a persistent reservoir of HTLV-1 infection, which has implications for viral propagation and possibly leukemogenesis.

## Materials and methods

### Human samples and Japanese macaques samples

Neutrophils (> 99%) and PBMC were isolated using 3% dextran and density gradient media; Ficoll-Paque PLUS (GE Healthcare Bio-Science). CD4 T lymphocytes (>98%), CD8 T lymphocytes (>98%), monocytes (> 97%) and B lymphocyte (>97%) were subsequently isolated by positive selection with BD IMag (BD Bioscience). Purity of cells is shown in parenthesis.

To obtain whole blood, bone marrow aspirates and organs from Japanese macaques (*Macaca fuscata*), four animals were euthanized with Pentobarbital (50mg/kg) [17]. Appropriate procedures were utilized in order to reduce potential distress, pain and discomfort. We obtained whole blood, bone marrow aspirates and organs for this study. Before sampling of organs, monkeys were perfused with phosphate buffered saline (PBS) to get rid of the contamination of PBMC in solid organs.

### Ethics statement

Blood samples from adult patients with HAM/TSP and a HTLV-1 carrier were collected after the written informed consent was obtained in accordance with the Declaration of Helsinki. These experiments were approved by the Institutional Ethics Committee of Kyoto University (approval number G311).

Six Japanese monkeys (*Macaca fuscata*) were used for this study. All monkeys were supplied from colonies in the Primate Research Institute. The monkeys were reared in outdoor group cages with wooded toys provided as environmental enrichment. They were fed with apple, potato and commercial monkey diet. They were able to access to water ad libitum. They had own health record from birth with yearly health checkup. Blood samples were obtained from the macaques under ketamine anesthesia with medetomidine, followed by administration of its antagonist atipamezole at the end of the procedure. At euthanasia, ketamine anesthesia to the macaques was followed by injection of pentobarbital sodium at a dose of ≥25 mg/kg. Then they were perfused with phosphate buffered saline (PBS) to get rid of the contamination of blood cells in solid organs before necropsy for this study. The animal experiment was approved by the Animal Welfare and Animal Care Committees of Kyoto University (approval number R11-11, R12-01, R13-01, R14-01 and R15-01), and was carried out in accordance with the Guidelines for Care and Use of Nonhuman Primates (Version3) by the Animal Welfare and Animal Care Committee of KUPRI. This guideline was prepared based on the provisions of the Guidelines for Proper Conduct of Animal Experiments (June 1, 2006; Science Council of Japan) as well as Fundamental Guidelines for Proper Conduct of Animal Experiment and Related Activities in Academic Research Institutions [Notice No. 71 of the Ministry of Education, Culture, Sports, Science and Technology dated June 1, 2006], in accordance with the recommendations of the Weatherall report, “The use of non-human primates in research”: http://www.acmedsci.ac.uk/more/news/the-use-of-non-human-primates-in-research/.

### Cells

HTLV-1 transformed human T-cell line (MT4, HPB-ATL-2) and HTLV-1 negative human T-cell lines (Jurkat, CCRF-CEM) were cultured in RPMI 1640 medium supplemented with 10% fetal bovine serum (FBS)(Biowest) and antibiotics. MT-4 and HPB-ATL-2 cells were gifts from Dr. Isao Miyoshi (Kochi University) and Dr. Shigeru Morikawa (Shimane University) respectively. Jurkat cells were obtained from Dr. Shimon Sakaguchi (Osaka University). JET WT35 is a subline of Jurkat cell expressing tdTomato under the control of 5 times tandem repeat of Tax responsive element (TRE)[45]. They were cultured with RPMI 1640 medium supplemented with 10%FBS, antibiotics and G418 (250 μg/mL) for selection.

### Detection of cell-to-cell infection by JET WT35 cells

5x10^4^ JET WT35 cells were co-cultured with 1x10^4^ cells of either HPB-ATL-2 or CEM cell lines in 12 wells plate in presence of either DMSO, azidothymidine (AZT) (5 μM), or raltegravir (RAL) (5 μM). 1.5x10^5^ DCs that were differentiated from monocytes of HAM/TSP patients were co-cultured with 1.5x10^5^ JET WT35 cells. After 48 hours, total number of tdTomato positive cells was counted. Images represent overlay of differential interference contrast and tdTomato channels.

### Proviral load

Proviral load was measured by real-time PCR as previously described [17, 23]. Briefly the copy number of the pX region and *RAG1* gene in genomic DNA was quantified. HTLV-1 proviral load was calculated with relative quantification method by using TL-Om1 of which proviral load is 100%. STLV-1 proviral load was calculated with absolute quantification method using plasmid DNA that contains STLV-1 sequence. We used serially diluted plasmid DNAs (the limit of detection is 100 copies) for standard curve. Therefore, we defined proviral load lower than 100 copies as under detection level (UD) in S1 Table. The sequences of primers for *RAG1* and pX were reported before [17] and newly constructed ones were as follows; *tax* primer (human) 5’-GAAGACTGTTTGCCCACCACC-3’ (sense) and 5’-TGAGGGTTGAGTGGAACGGA-3’ (anti-sense); pX probe (Human) was 5’-CACCCGTCACGCTAACAGCCTGGCAA-3’. The reaction conditions were 50°C for 2 minutes, 95°C for 10 minutes and 45 cycles of 15 seconds at 95°C, followed by 60seconds at 60°C.

### Quantitative analysis of viral gene expression

Total RNA was extracted using Trizol reagent (Thermo Fisher Scientific). The tissues of JMs were treated with RNA later (Thermo Fisher Scientific) to prevent RNA degradation. Reverse transcription was performed using random primer and SuperScript III reverse transcriptase (Thermo Fisher Scientific). The transcripts of *SBZ* and those of STLV-1 *tax* were measured by real time PCR. *GAPDH* mRNA was measured as internal control. The primers and probes for *GAPDH* were previously described [46]. Others were as follows; *stax* primers; 5’-ATCCCGTGGAGGCTCCTC-3’ (sense) and 5′-CCAAATACGTAGACTGGGTATCCAT-3′(anti-sense); *stax* probe; 5′-ACCAACACCATGGCCCACTTCCC-3′; *SBZ* primers; 5'-AGAGCGCAACTCAACCGG-3' (sense) and 5'-GCAGGGAACAGGTAAACA TCG-3'(anti-sense); *SBZ* probe; 5'-TGGATGGCGGCCTCAGGGCC-3'.The sequence of *GAPDH* primers and probe for JMs were same as those for humans. The amplification condition was 50°C for 2 min, 95°C for 10 min, 45 cycles of 95°C for 15sec and 60°C for 1 min. For the comparison of the expression level of *tax* with that of *SBZ* in each sample, we normalized the values of *tax* and *SBZ* per infected cell. Briefly, the relative expression levels of *SBZ* and *tax* were quantified by ddCt method using Si-2, which is an STLV-1-infected cell line, as a reference sample. Next, we determined absolute copy number of *tax* and *SBZ* transcripts in Si-2, and found that the copy numbers of *tax* and *SBZ* transcripts were 24.7 and 1, respectively. To normalize the expression levels of *SBZ* and *tax* in primary JM tissues, the value of *tax* was multiplied by 24.7 (S1 Fig).

### Flow cytometric analysis

Bone marrow mononuclear cells from two STLV-1 infected JMs and an uninfected JM were prepared by density gradient centrifugation using Ficoll-Paque PLUS (GE Healthcare Bio-Science). CD8 T lymphocytes were removed by positive selection with BD IMag (BD Bioscience). CD8 T lymphocytes depleted cells were cultured for 24 hours with RPMI 1640 medium supplemented with 10% fetal bovine serum (FBS) and antibiotics. The following antibody was used for cell surface staining: anti-CD4 (OKT4), CD34 (561) (all from BioLegend), CD8 (RPA-T8), CD3 (SP34-2), CD14 (M5E2), (all from BD Bioscience), CD19 (J3-119) (from Beckman Coulter), CD33 (AC104.3E3) (from Miltenyi Biotec). After cell surface staining, cells were fixed and permeabilized with Fixation/Permeabilization working solution (eBioscience), and Tax was stained by anti-Tax monoclonal antibody (MI73)(39). Samples were analyzed on a FACSVerse with FACSuite software (BD Biosciences) and data was analyzed with Flow Jo software (FlowJo, LLC).

### Differentiation of monocyte to dendritic cells *in vitro*

Monocytes from HAM/TSP patients were isolated using positive selection with BD IMag systems (BD Bioscience). Then, monocytes were cultured in AIMV medium (Thermo Fisher Scientific) supplemented with 5% human AB serum, IL-4 (10 ng/ml) and GM-CSF (10 ng/ml). To avoid *de novo* infection of HTLV-1, raltegravir (10 μM) or azidothymidine (5 μM) was added. After culture with the antiviral drugs for 5 days, cells were washed by RPMI supplemented with 10% FBS. Cells were then co-cultured with JET WT35 in RPMI 1640 medium supplemented with 10% FBS and antibiotics without G418. The JET WT35 cells are indicator Jurkat cells stably transfected with a plasmid that encodes the tdTomato under the control of the Tax responsive element. After 48 hours, tdTomato expression in co-cultured cells was observed with the EVOS FL fluorescence microscope (Thermo Fisher Scientific, 20× objective lens), and images were acquired by a CCD camera with which microscopy is equipped and built-in software.

### Immunofluorescent staining

Cells were fixed using 2% paraformaldehyde for 15min and fixed on poly-D-lysine coated glass or FRONTIER-coated slides (Matsunami-glass) by centrifugation. Fixed cells were permeabilized with 0.2% Triton X-100 for 15 min, blocked by overnight incubation in blocking solution (10% Blocking One and 5% Normal Goat Serum in PBS, both from Nakalai tesque, Japan) at 4°C, and then incubated with anti-Tax antibody (clone: MI73) (1:1000) for 3 days at 4°C [46]. After incubation, cells were gently washed five times with PBS, treated with secondary antibody (1:500 dilution, Alexa Fluor 488-conjugated goat anti-mouse IgG, abcam) for 1 h at room temperature, and subsequently rinsed five times with PBS. After re-blocking as described above, myeloperoxidase was stained to identify neutrophils. Cells were incubated with anti-myeloperoxidase antibody (1:200 dilution, abcam) for 1h at room temperature, rinsed five times with PBS, incubated with secondary antibody (1:500 dilution, Goat Anti-Rabbit IgG H&L (Alexa Fluor 568), abcam) for 1 h at room temperature, and washed five times with PBS. Then they were mounted with ProLong Gold antifade reagent with DAPI (Thermo Fisher Scientific) for staining of the nuclei. Images were observed using a confocal microscopy (FV1000-D IX81, Olympus, 40× objective lens) at room temperature and obtained with FV10-ASW software. Brightness and contrast were adjusted by ImageJ software (National Institutes of Health, http://imagej.nih.gov/ij/index.html).

### High throughput sequencing of provirus integration sites

Integration sites were amplified with ligation mediated PCR and high-throughput sequencing was performed as previously reported with some modifications using Ion Torrent Personal Genome Machine (Ion PGM, Thermo Fisher Scientific) or Miseq (Illumina) [17, 47]. Genomic DNA was extracted with phenol/chloroform method and sheared by sonication with a Covaris M220 instrument (Covaris). After end-repair and linker ligation, nested PCR was performed to amplify the integration sites using the primers specific for viral and linker sequences. Amplicons were size-selected with E-Gel SizeSelect Agarose Gel (Thermo Fisher Scientific) to generate libraries for Ion PGM. Templates were prepared by Ion PGM Hi-Q OT2 Kit or Ion PGM Template OT2 400 Kit, and then sequencing was performed on Ion 318 Chip Kit v2 using Ion PGM Hi-Q Sequencing Kit or Ion PGM Sequencing 400 Kit (Thermo Fisher Scientific). For Miseq, additional steps were needed after nested PCR. TruSeq DNA PCR-Free Sample Prep Kit (Illumina) was used to ligate the adaptor specific for Miseq according to the manuscripts but without fragmentation because samples were already fragmented. PCR products after nested PCR were used as input DNA in that case. High throughput sequencing was performed according to the manufacturer’s instructions.

### Bioinformatics

For Illumina pair-end sequencing, the obtained reads were trimmed with a quality threshold of 20 on the Phred scale and minimum length of both pairs of 20 bp in order to remove low quality reads and short reads using Trim Galore! (http://www.bioinformatics.babraham.ac.uk/projects/trim_galore/). The end of viral sequence (“TTTAGTACACA” was used as a marker) and that of linker sequence (“TCGCTCTTCCGATCT” was used as a marker) were removed from Read1 and Read2. Trimmed reads were arranged as Read1 including sequence started from the beginning base of integration site and Read2 including sequence started from the end base of shear-site and aligned to human genome reference (UCSC hg38) using Burrows-Wheeler Aligner (BWA)[48]. The reads were filtered by mapping quality, removing supplementary reads and excluding un-paired reads with SAMtools software package [49]. Minus strand sequences were converted into complementary sequencing to count the number of clones and PCR duplicates. Sequence similarity for the longest sequence in each integration site was evaluated through the program ClustalW (version 2) in order to remove twin integration sites arising from mismapping of some duplicates [50]. With regard to the pair of clones with high homology score (>85), the clone which has the smaller number of shear sites was removed. When the number of shear sites was same, we used total read number including the number of PCR products in addition to the number of shear sites. Furthermore, both clones were excluded when the pair of clones has the same shear-sites number and the same number of reads.

For Ion PGM single end sequencing, data analysis was done as previously reported with some modifications [17]. When extracting the host genomic sequences, the viral 3’ LTR sequence (TAGTACACA) and the linker sequence (AGATCGGAA) were regarded as the tags and removed. After the reads were mapped to human genome reference (UCSC hg38) by BWA, they were filtered by the mapping quality. The length of host genomic sequences was calculated using the start position and cigar codes in the SAM files to count the number of clones and PCR duplicates. Sequence similarity was assessed as described earlier.

## Supporting information

S1 Fig Quantification and normalization of *tax* and *SBZ* expression by real-time PCR. (A) Transcripts of STLV-1 *tax* and *SBZ* in various tissues were quantified by real-time PCR. In order to compare the expression levels of *SBZ* and *tax* in each sample, we calculated their relative expression values as follows. To normalize the expression values of all samples, we decided to use cDNA of Si-2, which is an STLV-1-infected cell line, as a reference sample. First we determined the absolute copy numbers of *SBZ* and *tax* in Si-2 cDNA by the standard curve method. To draw the standard curves to quantify *SBZ* or *tax* transcripts, we generated the plasmids containing the fragments of the *SBZ* gene or the *tax* gene. The standard curves drawn with *SBZ*- and *tax*-encoding plasmids were quite similar as shown in S1 Fig A, indicating that PCR efficiency of *SBZ* was similar to that of *tax*. (B) Using these standard curves, we found that the copy numbers of *tax* and *SBZ* in Si-2 were 35.8 copies and 1.45 copies respectively, showing that the expression of *tax* was 24.7 times higher than that of *SBZ* in Si-2 (the ratio of *tax* to *SBZ* was 24.7:1 in Si-2). (C) The relative expression levels of *SBZ* and *tax* in all JM tissues were quantified by the ddCt method using the Si-2 cDNA as a reference sample (*SBZ* in Si-2 was assumed as 1, and tax in Si-2 was done as 24.7 for normalization). Since percentages of infected cells in each tissue were varied, the expression values of *SBZ* and *tax* were divided by the proviral load of each sample to reflect the expression levels of *SBZ* and *tax* per infected cell. An example of the normalized value is shown.
(PPTX)

S2 Fig Tax expression in STLV-1 uninfected JM and B cells of STLV-1 infected JMs. Bone marrow cells were stained with antibodies to Tax, CD3, CD4, CD8, CD34, CD33 and CD19, and analyzed using flow cytometry. (A) Bone marrow cells from a uninfected JM (JM6) were negative for Tax expression when compared with patterns by isotype antibody. (B) CD19 positive B cells of STLV-1 infected JMs (JM4, 5) showed weak positivity for Tax expression.
(PPTX)

S3 Fig Differentiation to DCs in a HAM/TSP patient and a healthy control. Monocyte derived dendritic cells (MDDC) from a healthy donor and a HAM/TSP patient were assessed by flow cytometry to confirm their differentiation into DCs. CD14 was negative, and CD11c and CD209 were positive for MDDC.
(PPTX)

S1 Table Proviral load in STLV-1 infected Japanese macaques. STLV-1 proviral loads were measured by quantitative PCR.
(DOCX)

S2 Table Integration sites of HTLV-1 in PBMC and neutrophil of HAM/TSP#1. Integration sites of HTLV-1 provirus were determined by high-throughput sequencing method in PBMC and neutrophils of HAM/TSP#1.
(DOCX)

S3 Table The number of sequence reads and identified HTLV-1 infected clones. The number of sequence reads and HTLV-1 infected clones were shown.
(DOCX)

S4 Table Proviral loads in different hematopoietic lineage cells of HAM/TSP patients. Proviral loads were measured by realtime PCR and shown.
(DOCX)

S5 Table Integration sites of HTLV-1 provirus in this study. This table presents all integration sites of HTLV-1 provirus in all HTLV-1 infected individuals of this study.
(DOCX)

S6 Table Clonality of HTLV-1 infected cells at different time point. Integration sites of HTLV-1 provirus in various hematopoietic cells and neutrophils at different time point were shown.
(DOCX)
