# Supplementary material for: Human T-cell leukemia virus type 1 infects multiple lineage hematopoietic cells in vivo
Source: PLoS Pathog. 2017 Nov 29;13(11):e1006722. doi: 10.1371/journal.ppat.1006722 (PMC5724899; doi:10.1371/journal.ppat.1006722)
Supplement: S1 Fig — (A) Transcripts of STLV-1 tax and SBZ in various tissues were quantified by real-time PCR. In order to compare the expression levels of SBZ and tax in each sample, we calculated their relative expression values as follows. To normalize the expression values of all samples, we decided to use cDNA of Si-2, which is an STLV-1-infected cell line, as a reference sample. First we determined the absolute copy numbers of SBZ and tax in Si-2 cDNA by the standard curve method. To draw the standard curves to quantify SBZ or tax transcripts, we generated the plasmids containing the fragments of the SBZ gene or the tax gene. The standard curves drawn with SBZ- and tax-encoding plasmids were quite similar as shown in S1 Fig A, indicating that PCR efficiency of SBZ was similar to that of tax. (B) Using these standard curves, we found that the copy numbers of tax and SBZ in Si-2 were 35.8 copies and 1.45 copies respectively, showing that the expression of tax was 24.7 times higher than that of SBZ in Si-2 (the ratio of tax to SBZ was 24.7:1 in Si-2). (C) The relative expression levels of SBZ and tax in all JM tissues were quantified by the ddCt method using the Si-2 cDNA as a reference sample (SBZ in Si-2 was assumed as 1, and tax in Si-2 was done as 24.7 for normalization). Since percentages of infected cells in each tissue were varied, the expression values of SBZ and tax were divided by the proviral load of each sample to reflect the expression levels of SBZ and tax per infected cell. An example of the normalized value is shown. (PPTX) [file ppat.1006722.s001.pptx]

## Slide 1
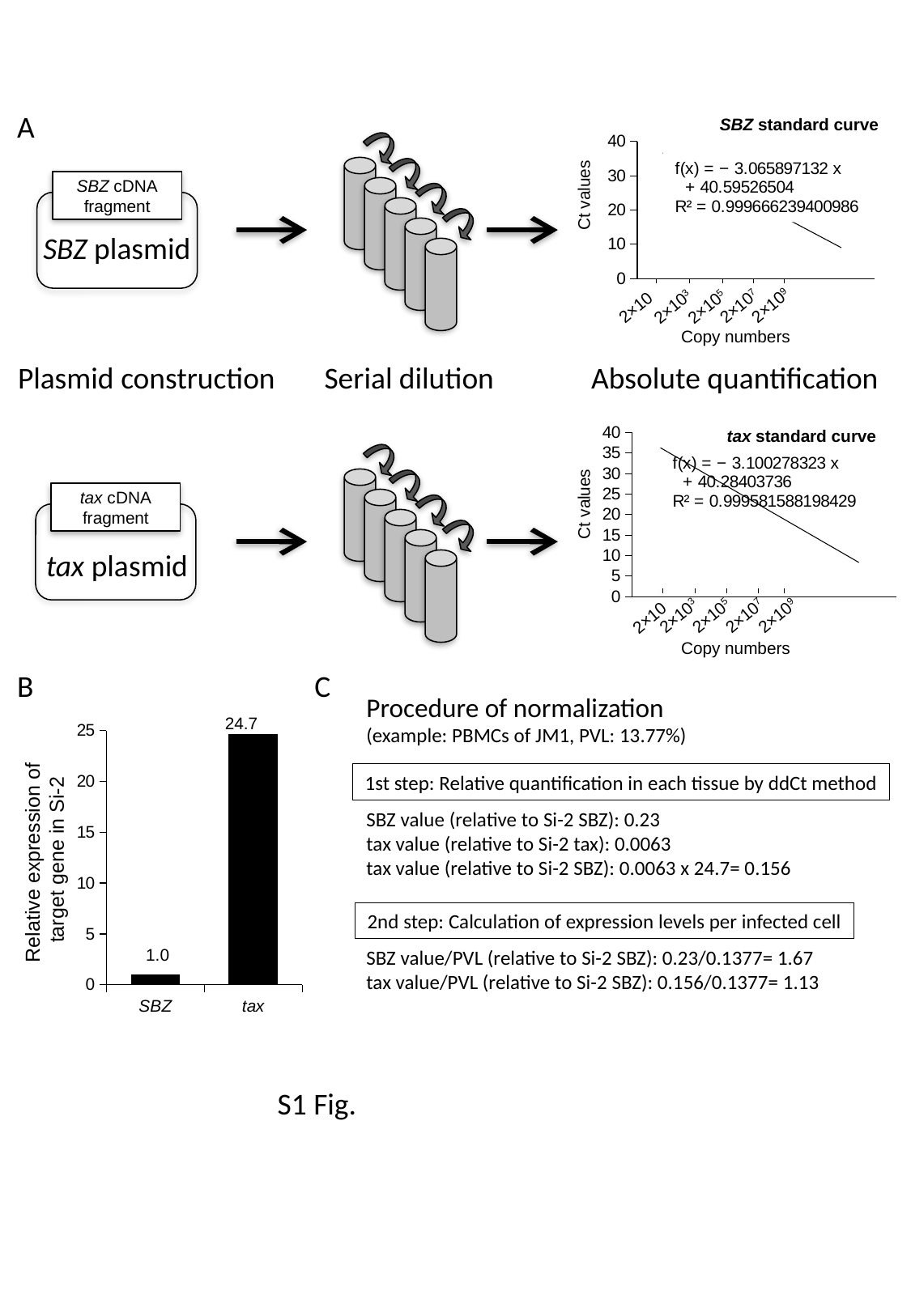

A
SBZ standard curve
### Chart
| Category | Y の値 1 |
|---|---|Ct values
2×10^9^
2×10
2×10^7^
2×10^3^
2×10^5^
Copy numbers
SBZ cDNA
fragment
SBZ plasmid
Plasmid construction
Serial dilution
Absolute quantification
### Chart
| Category | Y の値 1 |
|---|---|tax standard curve
Ct values
2×10^3^
2×10^5^
2×10^7^
2×10^9^
2×10
Copy numbers
tax cDNA
fragment
tax plasmid
B
C
Procedure of normalization
(example: PBMCs of JM1, PVL: 13.77%)
24.7
### Chart
| Category | |
|---|---|
| SBZ | 1.0 |
| tax | 24.6512841340674 |Relative expression of
target gene in Si-2
1.0
1st step: Relative quantification in each tissue by ddCt method
SBZ value (relative to Si-2 SBZ): 0.23
tax value (relative to Si-2 tax): 0.0063
tax value (relative to Si-2 SBZ): 0.0063 x 24.7= 0.156
2nd step: Calculation of expression levels per infected cell
SBZ value/PVL (relative to Si-2 SBZ): 0.23/0.1377= 1.67
tax value/PVL (relative to Si-2 SBZ): 0.156/0.1377= 1.13
S1 Fig.
