# Supplementary material for: Human T-cell leukemia virus type 1 infects multiple lineage hematopoietic cells in vivo
Source: PLoS Pathog. 2017 Nov 29;13(11):e1006722. doi: 10.1371/journal.ppat.1006722 (PMC5724899; doi:10.1371/journal.ppat.1006722)
Supplement: S2 Fig — Bone marrow cells were stained with antibodies to Tax, CD3, CD4, CD8, CD34, CD33 and CD19, and analyzed using flow cytometry. (A) Bone marrow cells from a uninfected JM (JM6) were negative for Tax expression when compared with patterns by isotype antibody. (B) CD19 positive B cells of STLV-1 infected JMs (JM4, 5) showed weak positivity for Tax expression. (PPTX) [file ppat.1006722.s002.pptx]

## Slide 1
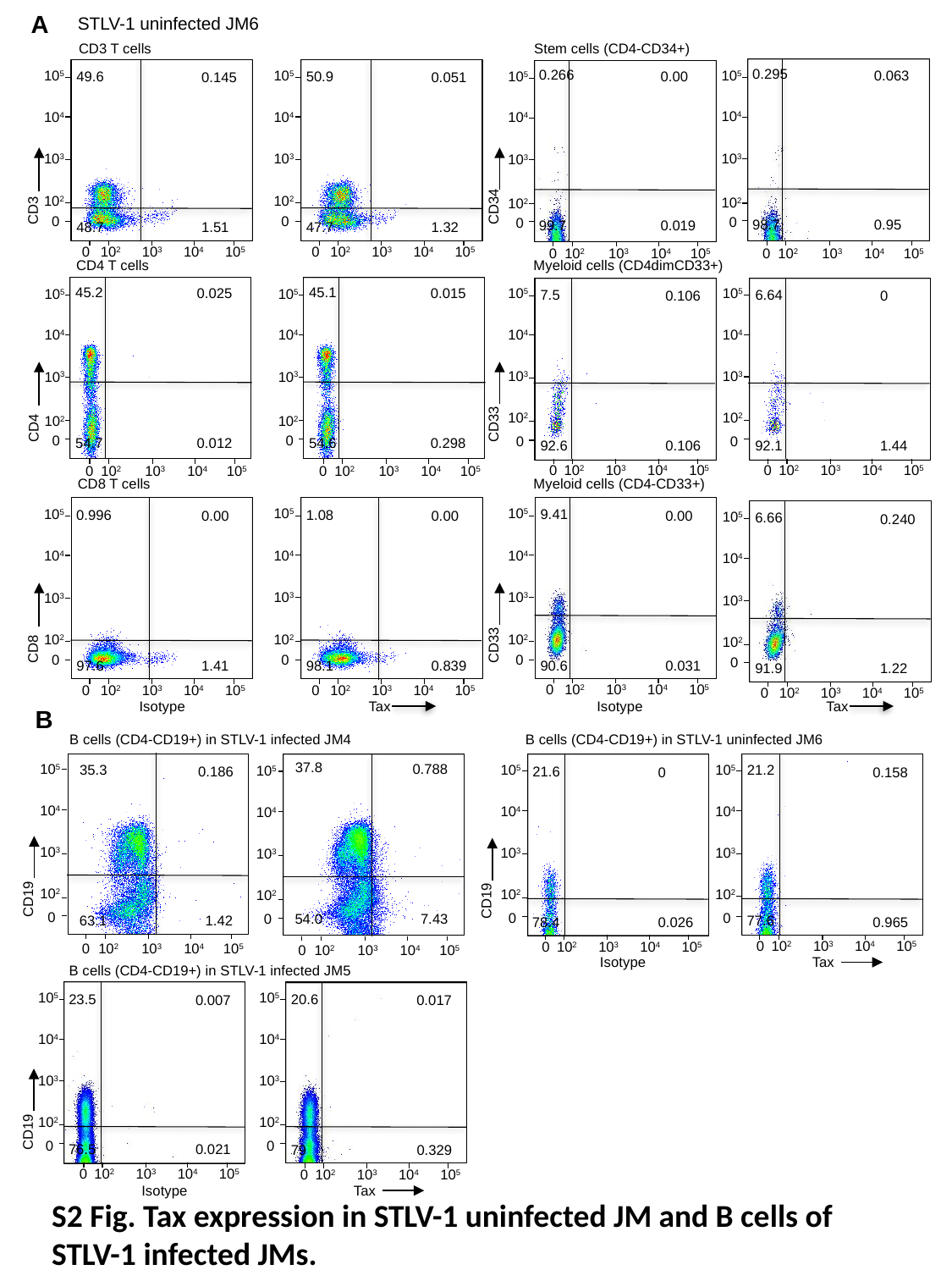

A
STLV-1 uninfected JM6
CD3 T cells
Stem cells (CD4-CD34+)
105
104
103
102
0
0
102
103
104
105
0.295
0.063
98.7
0.95
105
104
103
102
 0
0
102
103
104
105
49.6
0.145
48.7
1.51
105
104
103
102
 0
0
102
103
104
105
50.9
0.051
47.7
1.32
105
104
103
102
0
0
102
103
104
105
0.266
0.00
99.7
0.019
CD34
CD3
CD4 T cells
Myeloid cells (CD4dimCD33+)
105
104
103
102
0
0
102
103
104
105
45.2
0.025
54.7
0.012
105
104
103
102
0
0
102
103
104
105
45.1
0.015
54.6
0.298
105
104
103
102
0
0
102
103
104
105
7.5
0.106
92.6
0.106
105
104
103
102
0
0
102
103
104
105
6.64
0
92.1
1.44
CD33
CD4
CD8 T cells
Myeloid cells (CD4-CD33+)
105
104
103
102
 0
0
102
103
104
105
0.996
0.00
97.6
1.41
105
104
103
102
 0
0
102
103
104
105
1.08
0.00
98.1
0.839
105
104
103
102
 0
0
102
103
104
105
9.41
0.00
90.6
0.031
105
104
103
102
 0
0
102
103
104
105
6.66
0.240
91.9
1.22
CD33
CD8
Tax
Tax
Isotype
Isotype
B
B cells (CD4-CD19+) in STLV-1 infected JM4
B cells (CD4-CD19+) in STLV-1 uninfected JM6
105
104
103
102
0
0
102
103
104
105
35.3
0.186
63.1
1.42
37.8
0.788
105
104
103
102
0
0
102
103
104
105
54.0
7.43
105
104
103
102
0
0
102
103
104
105
21.6
0
78.4
0.026
105
104
103
102
0
0
102
103
104
105
21.2
0.158
77.6
0.965
CD19
CD19
Isotype
Tax
B cells (CD4-CD19+) in STLV-1 infected JM5
105
104
103
102
0
0
102
103
104
105
20.6
0.017
79
0.329
105
104
103
102
0
0
102
103
104
105
23.5
0.007
76.5
0.021
CD19
Isotype
Tax
S2 Fig. Tax expression in STLV-1 uninfected JM and B cells of STLV-1 infected JMs.
