# Supplementary material for: Human T-cell leukemia virus type 1 infects multiple lineage hematopoietic cells in vivo
Source: PLoS Pathog. 2017 Nov 29;13(11):e1006722. doi: 10.1371/journal.ppat.1006722 (PMC5724899; doi:10.1371/journal.ppat.1006722)
Supplement: S3 Fig — Monocyte derived dendritic cells (MDDC) from a healthy donor and a HAM/TSP patient were assessed by flow cytometry to confirm their differentiation into DCs. CD14 was negative, and CD11c and CD209 were positive for MDDC. (PPTX) [file ppat.1006722.s003.pptx]

## Slide 1
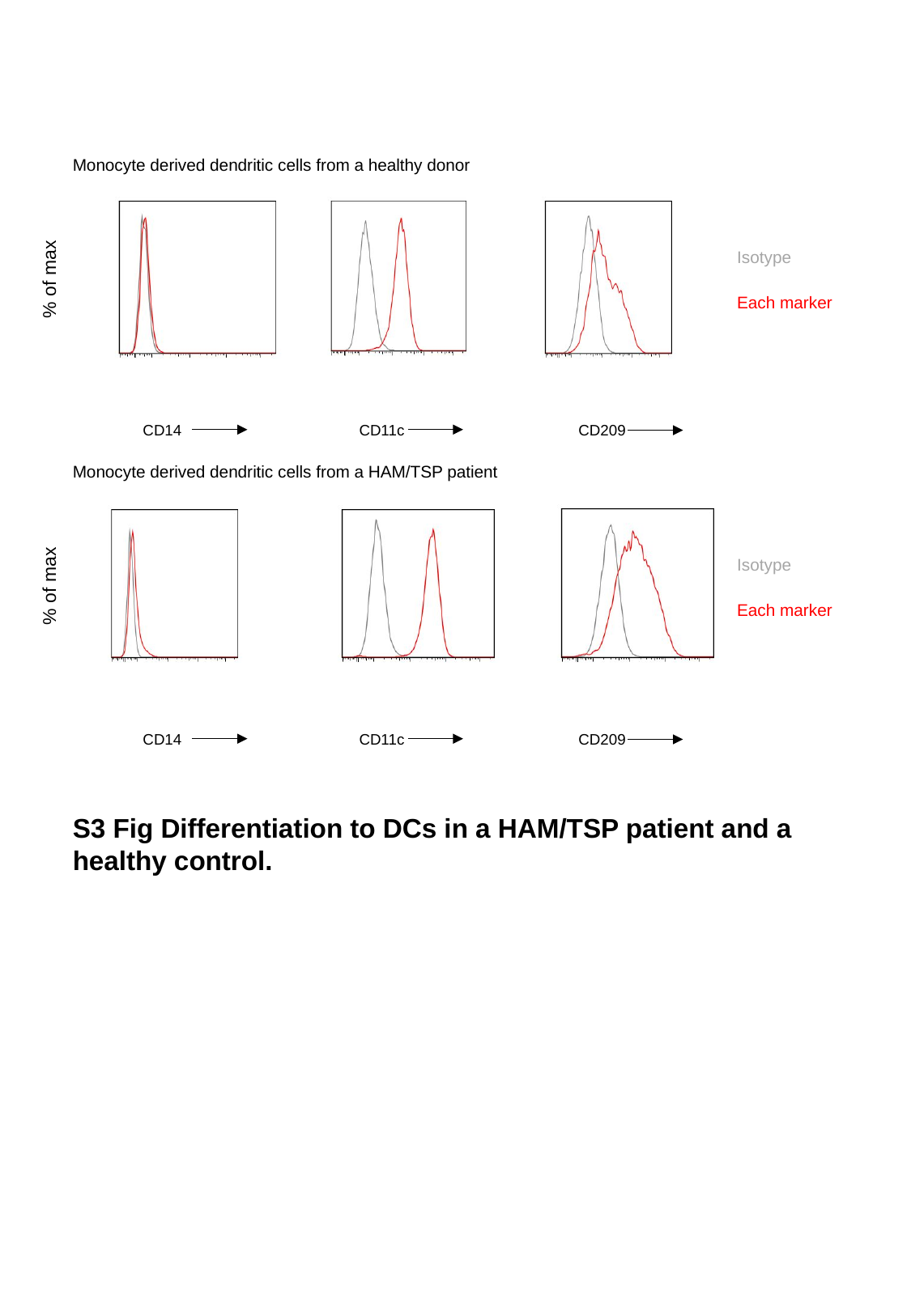

Monocyte derived dendritic cells from a healthy donor
% of max
Isotype
Each marker
　CD14
　CD11c
　CD209
Monocyte derived dendritic cells from a HAM/TSP patient
% of max
Isotype
Each marker
　CD14
　CD11c
　CD209
S3 Fig Differentiation to DCs in a HAM/TSP patient and a healthy control.
