# Supplementary material for: Human T-cell leukemia virus type 1 infects multiple lineage hematopoietic cells in vivo
Source: PLoS Pathog. 2017 Nov 29;13(11):e1006722. doi: 10.1371/journal.ppat.1006722 (PMC5724899; doi:10.1371/journal.ppat.1006722)
Supplement: S1 Table — STLV-1 proviral loads were measured by quantitative PCR. (DOCX) [file ppat.1006722.s004.docx]

**Table S1. Proviral load in STLV-1 infected Japanese macaques.**

| Proviral load (%) | JM1 | JM2 | JM3 |
| --- | --- | --- | --- |
| PBMC | 13.77 | 13.99 | 11.51 |
| Bone Marrow | 6.67 | 3.04 | 4.60 |
| Submandibular LN | 7.14 | 14.56 | ND |
| Axillary LN | 2.17 | 23.13 | 6.27 |
| Inguinal LN | 3.21 | 11.76 | ND |
| Mesenteric LN | UD | 11.35 | 3.00 |
| Pulmonary hilar LN | 5.81 | 25.98 | ND |
| Spleen | 4.92 | 7.99 | 7.87 |
| Ileocecum | 1.53 | 1.13 | 2.00 |
| Peyer's patch | 1.18 | 1.56 | 1.98 |
| Colon | 0.59 | 0.91 | 1.21 |
| Small intestine | 1.44 | 1.17 | ND |
| Brain | UD | UD | ND |
| Spinal cord | 0.55 | 1.68 | ND |
| Lung | 2.27 | UD | 3.06 |
| Liver | 0.86 | 2.28 | 0.64 |
| Skin | 0.36 | 4.60 | ND |
| Salivary gland | 0.85 | 7.54 | 2.29 |
| Thymus | ND | ND | 1.86 |

ND: not determined

UD: under detection level
