# Supplementary material for: Human T-cell leukemia virus type 1 infects multiple lineage hematopoietic cells in vivo
Source: PLoS Pathog. 2017 Nov 29;13(11):e1006722. doi: 10.1371/journal.ppat.1006722 (PMC5724899; doi:10.1371/journal.ppat.1006722)
Supplement: S2 Table — Integration sites of HTLV-1 provirus were determined by high-throughput sequencing method in PBMC and neutrophils of HAM/TSP#1. (DOCX) [file ppat.1006722.s005.docx]

**Table S2. Integration sites of HTLV-1 in PBMC and neutrophil of HAM/TSP#1.**

|  | Clone ID | PBMC | Neutrophils |
| --- | --- | --- | --- |
| 1 | 109 | 37 | 2 |
| 2 | 427 | 5 | 1 |
| 3 | 482 | 188 | 16 |
| 4 | 875 | 194 | 23 |
| 5 | 996 | 2 | 3 |
| 6 | 1002 | 30 | 1 |
| 7 | 1010 | 1 | 1 |
| 8 | 1106 | 8 | 1 |
| 9 | 1146 | 27 | 1 |
| 10 | 1732 | 8 | 2 |
| 11 | 1888 | 11 | 1 |
| 12 | 2154 | 48 | 3 |
| 13 | 2180 | 2 | 3 |
| 14 | 2293 | 7 | 2 |
| 15 | 2432 | 29 | 1 |
| 16 | 2462 | 1 | 2 |
| 17 | 2596 | 11 | 4 |
| 18 | 2831 | 5 | 6 |

PBMC: Peripheral blood mononuclear cells.
