# Supplementary material for: Human T-cell leukemia virus type 1 infects multiple lineage hematopoietic cells in vivo
Source: PLoS Pathog. 2017 Nov 29;13(11):e1006722. doi: 10.1371/journal.ppat.1006722 (PMC5724899; doi:10.1371/journal.ppat.1006722)
Supplement: S3 Table — The number of sequence reads and HTLV-1 infected clones were shown. (DOCX) [file ppat.1006722.s006.docx]

**Table S3. The number of sequence reads and identified clones.**

| Patient | Cell types | Sequence reads | Clones |
| --- | --- | --- | --- |
| HAM/TSP#1 | PBMC | 3244613 | 8018 |
|  | Neutrophils | 880749 | 100 |
| HAM/TSP#2 | CD4 T cells | 7645036 | 6856 |
|  | CD8 T cells | 5876193 | 1604 |
|  | B cells | 2922091 | 137 |
|  | Monocytes | 5734501 | 208 |
|  | Neutrophils | 1077181 | 204 |
|  | Neutrophils 1year later | 5145383 | 812 |
| HAM/TSP#3 | CD4 T cells | 8442702 | 9572 |
|  | CD8 T cells | 8147740 | 2376 |
|  | B cells | 8049807 | 64 |
|  | Monocytes | 4642506 | 422 |
|  | Neutrophils | 8190619 | 47 |
|  | Neutrophils 1year later | 619363 | 25 |
| HTLV-1 carrier#1 | CD4 T cells | 7457492 | 10642 |
|  | CD8 T cells | 6408343 | 2342 |
|  | B cells | 2744439 | 309 |
|  | Monocytes | 6609635 | 1473 |
|  | Neutrophils | 3643807 | 921 |
