# Supplementary material for: Human T-cell leukemia virus type 1 infects multiple lineage hematopoietic cells in vivo
Source: PLoS Pathog. 2017 Nov 29;13(11):e1006722. doi: 10.1371/journal.ppat.1006722 (PMC5724899; doi:10.1371/journal.ppat.1006722)
Supplement: S4 Table — Proviral loads were measured by realtime PCR and shown. (DOCX) [file ppat.1006722.s007.docx]

**Table S4. Proviral loads in different hematopoietic lineage cells of HAM/TSP patients.**

| PVL (%) | HAM/TSP#1 | HAM/TSP#2 | HAM/TSP#3 |
| --- | --- | --- | --- |
| PBMC | 10.8 | 18.9 | 21 |
| CD4 T cells | ND | 29 | 31.7 |
| CD8 T cells | ND | 11.5 | 2.7 |
| B cells | ND | 0.03 | 0.02 |
| Monocytes | ND | 0.06 | 0.12 |
| Neutrophils | 0.06 | 0.12 | 0.04 |

ND: not determined
