# Supplementary material for: Human T-cell leukemia virus type 1 infects multiple lineage hematopoietic cells in vivo
Source: PLoS Pathog. 2017 Nov 29;13(11):e1006722. doi: 10.1371/journal.ppat.1006722 (PMC5724899; doi:10.1371/journal.ppat.1006722)
Supplement: S5 Table — This table presents all integration sites of HTLV-1 provirus in all HTLV-1 infected individuals of this study. (DOCX) [file ppat.1006722.s008.docx]

**S5 Table. Integration sites in all patients.**

| Patient | CloneID | Integration sites | CD4 | CD8 | B | Mono | Neutro | PBMC |
| --- | --- | --- | --- | --- | --- | --- | --- | --- |
| HAM/TSP#1 | 1 | chr1_100419150_+ |  |  |  |  | 0 | 1 |
| HAM/TSP#1 | 2 | chr1_100651630_- |  |  |  |  | 0 | 1 |
| HAM/TSP#1 | 3 | chr1_102707850_+ |  |  |  |  | 0 | 1 |
| HAM/TSP#1 | 4 | chr1_103377946_+ |  |  |  |  | 0 | 1 |
| HAM/TSP#1 | 5 | chr1_106505786_- |  |  |  |  | 0 | 5 |
| HAM/TSP#1 | 6 | chr1_10735549_- |  |  |  |  | 0 | 1 |
| HAM/TSP#1 | 7 | chr1_107928293_- |  |  |  |  | 0 | 1 |
| HAM/TSP#1 | 8 | chr1_107957123_- |  |  |  |  | 0 | 2 |
| HAM/TSP#1 | 9 | chr1_107957124_- |  |  |  |  | 0 | 1 |
| HAM/TSP#1 | 10 | chr1_10815606_- |  |  |  |  | 0 | 1 |
| HAM/TSP#1 | 11 | chr1_110640793_+ |  |  |  |  | 0 | 4 |
| HAM/TSP#1 | 12 | chr1_110669843_- |  |  |  |  | 0 | 2 |
| HAM/TSP#1 | 13 | chr1_111350760_+ |  |  |  |  | 0 | 2 |
| HAM/TSP#1 | 14 | chr1_113073708_- |  |  |  |  | 0 | 1 |
| HAM/TSP#1 | 15 | chr1_11367783_+ |  |  |  |  | 0 | 7 |
| HAM/TSP#1 | 16 | chr1_113883278_+ |  |  |  |  | 0 | 1 |
| HAM/TSP#1 | 17 | chr1_115006451_- |  |  |  |  | 0 | 1 |
| HAM/TSP#1 | 18 | chr1_115641025_+ |  |  |  |  | 0 | 2 |
| HAM/TSP#1 | 19 | chr1_116089201_+ |  |  |  |  | 0 | 1 |
| HAM/TSP#1 | 20 | chr1_116178161_- |  |  |  |  | 0 | 1 |
| HAM/TSP#1 | 21 | chr1_117247326_- |  |  |  |  | 0 | 1 |
| HAM/TSP#1 | 22 | chr1_117625503_+ |  |  |  |  | 0 | 4 |
| HAM/TSP#1 | 23 | chr1_117660803_- |  |  |  |  | 0 | 3 |
| HAM/TSP#1 | 24 | chr1_118517392_+ |  |  |  |  | 0 | 1 |
| HAM/TSP#1 | 25 | chr1_119911099_- |  |  |  |  | 0 | 1 |
| HAM/TSP#1 | 26 | chr1_12218575_- |  |  |  |  | 0 | 1 |
| HAM/TSP#1 | 27 | chr1_133142_+ |  |  |  |  | 0 | 1 |
| HAM/TSP#1 | 28 | chr1_13424465_- |  |  |  |  | 0 | 1 |
| HAM/TSP#1 | 29 | chr1_14729426_- |  |  |  |  | 0 | 1 |
| HAM/TSP#1 | 30 | chr1_147571776_+ |  |  |  |  | 0 | 4 |
| HAM/TSP#1 | 31 | chr1_150113327_+ |  |  |  |  | 0 | 2 |
| HAM/TSP#1 | 32 | chr1_150349645_- |  |  |  |  | 0 | 1 |
| HAM/TSP#1 | 33 | chr1_151184437_- |  |  |  |  | 0 | 1 |
| HAM/TSP#1 | 34 | chr1_151305284_- |  |  |  |  | 0 | 1 |
| HAM/TSP#1 | 35 | chr1_153458971_- |  |  |  |  | 0 | 1 |
| HAM/TSP#1 | 36 | chr1_15372568_- |  |  |  |  | 0 | 1 |
| HAM/TSP#1 | 37 | chr1_157253173_+ |  |  |  |  | 0 | 3 |
| HAM/TSP#1 | 38 | chr1_15792142_+ |  |  |  |  | 0 | 1 |
| HAM/TSP#1 | 39 | chr1_158949941_- |  |  |  |  | 0 | 1 |
| HAM/TSP#1 | 40 | chr1_160290227_- |  |  |  |  | 0 | 1 |
| HAM/TSP#1 | 41 | chr1_160991105_- |  |  |  |  | 0 | 9 |
| HAM/TSP#1 | 42 | chr1_161547532_+ |  |  |  |  | 0 | 4 |
| HAM/TSP#1 | 43 | chr1_16270250_- |  |  |  |  | 0 | 2 |
| HAM/TSP#1 | 44 | chr1_163773777_- |  |  |  |  | 0 | 1 |
| HAM/TSP#1 | 45 | chr1_164049827_+ |  |  |  |  | 0 | 11 |
| HAM/TSP#1 | 46 | chr1_164253437_+ |  |  |  |  | 0 | 1 |
| HAM/TSP#1 | 47 | chr1_164742977_+ |  |  |  |  | 0 | 1 |
| HAM/TSP#1 | 48 | chr1_165136345_- |  |  |  |  | 0 | 1 |
| HAM/TSP#1 | 49 | chr1_166090824_- |  |  |  |  | 0 | 6 |
| HAM/TSP#1 | 50 | chr1_167135101_- |  |  |  |  | 0 | 1 |
| HAM/TSP#1 | 51 | chr1_171350885_- |  |  |  |  | 0 | 1 |
| HAM/TSP#1 | 52 | chr1_171981625_- |  |  |  |  | 0 | 1 |
| HAM/TSP#1 | 53 | chr1_172357014_- |  |  |  |  | 0 | 9 |
| HAM/TSP#1 | 54 | chr1_172357015_- |  |  |  |  | 0 | 1 |
| HAM/TSP#1 | 55 | chr1_174539812_+ |  |  |  |  | 0 | 1 |
| HAM/TSP#1 | 56 | chr1_175762995_- |  |  |  |  | 0 | 1 |
| HAM/TSP#1 | 57 | chr1_176137783_- |  |  |  |  | 0 | 8 |
| HAM/TSP#1 | 58 | chr1_177126217_+ |  |  |  |  | 0 | 1 |
| HAM/TSP#1 | 59 | chr1_177186912_+ |  |  |  |  | 0 | 1 |
| HAM/TSP#1 | 60 | chr1_177401313_+ |  |  |  |  | 0 | 1 |
| HAM/TSP#1 | 61 | chr1_17906972_+ |  |  |  |  | 0 | 1 |
| HAM/TSP#1 | 62 | chr1_179247083_- |  |  |  |  | 0 | 2 |
| HAM/TSP#1 | 63 | chr1_180549315_- |  |  |  |  | 0 | 1 |
| HAM/TSP#1 | 64 | chr1_181798079_- |  |  |  |  | 0 | 1 |
| HAM/TSP#1 | 65 | chr1_181849049_- |  |  |  |  | 0 | 1 |
| HAM/TSP#1 | 66 | chr1_182668665_- |  |  |  |  | 0 | 5 |
| HAM/TSP#1 | 67 | chr1_183185581_- |  |  |  |  | 0 | 1 |
| HAM/TSP#1 | 68 | chr1_184487016_- |  |  |  |  | 0 | 1 |
| HAM/TSP#1 | 69 | chr1_184773060_+ |  |  |  |  | 0 | 2 |
| HAM/TSP#1 | 70 | chr1_185054283_+ |  |  |  |  | 0 | 1 |
| HAM/TSP#1 | 71 | chr1_185460501_- |  |  |  |  | 0 | 1 |
| HAM/TSP#1 | 72 | chr1_185980581_+ |  |  |  |  | 0 | 30 |
| HAM/TSP#1 | 73 | chr1_18622434_- |  |  |  |  | 0 | 4 |
| HAM/TSP#1 | 74 | chr1_186622270_+ |  |  |  |  | 0 | 1 |
| HAM/TSP#1 | 75 | chr1_188047760_+ |  |  |  |  | 0 | 1 |
| HAM/TSP#1 | 76 | chr1_188732003_+ |  |  |  |  | 0 | 1 |
| HAM/TSP#1 | 77 | chr1_189248852_+ |  |  |  |  | 0 | 10 |
| HAM/TSP#1 | 78 | chr1_18976196_- |  |  |  |  | 0 | 1 |
| HAM/TSP#1 | 79 | chr1_191176168_- |  |  |  |  | 0 | 1 |
| HAM/TSP#1 | 80 | chr1_192243216_+ |  |  |  |  | 0 | 2 |
| HAM/TSP#1 | 81 | chr1_193323288_- |  |  |  |  | 0 | 2 |
| HAM/TSP#1 | 82 | chr1_196830233_- |  |  |  |  | 0 | 3 |
| HAM/TSP#1 | 83 | chr1_197529338_+ |  |  |  |  | 0 | 1 |
| HAM/TSP#1 | 84 | chr1_200402997_+ |  |  |  |  | 0 | 1 |
| HAM/TSP#1 | 85 | chr1_20085069_- |  |  |  |  | 0 | 1 |
| HAM/TSP#1 | 86 | chr1_201167364_+ |  |  |  |  | 0 | 3 |
| HAM/TSP#1 | 87 | chr1_20152159_- |  |  |  |  | 0 | 1 |
| HAM/TSP#1 | 88 | chr1_202578955_- |  |  |  |  | 0 | 5 |
| HAM/TSP#1 | 89 | chr1_203270285_- |  |  |  |  | 0 | 1 |
| HAM/TSP#1 | 90 | chr1_2037454_+ |  |  |  |  | 0 | 1 |
| HAM/TSP#1 | 91 | chr1_204638972_+ |  |  |  |  | 0 | 1 |
| HAM/TSP#1 | 92 | chr1_204756284_- |  |  |  |  | 0 | 2 |
| HAM/TSP#1 | 93 | chr1_205018462_- |  |  |  |  | 0 | 1 |
| HAM/TSP#1 | 94 | chr1_205157370_- |  |  |  |  | 0 | 1 |
| HAM/TSP#1 | 95 | chr1_20634970_+ |  |  |  |  | 0 | 1 |
| HAM/TSP#1 | 96 | chr1_206600789_+ |  |  |  |  | 0 | 1 |
| HAM/TSP#1 | 97 | chr1_209454002_- |  |  |  |  | 0 | 1 |
| HAM/TSP#1 | 98 | chr1_21022974_+ |  |  |  |  | 0 | 9 |
| HAM/TSP#1 | 99 | chr1_210485712_- |  |  |  |  | 0 | 1 |
| HAM/TSP#1 | 100 | chr1_211858466_+ |  |  |  |  | 0 | 1 |
| HAM/TSP#1 | 101 | chr1_212502311_- |  |  |  |  | 0 | 1 |
| HAM/TSP#1 | 102 | chr1_213483701_+ |  |  |  |  | 0 | 1 |
| HAM/TSP#1 | 103 | chr1_216202170_+ |  |  |  |  | 0 | 1 |
| HAM/TSP#1 | 104 | chr1_220915816_- |  |  |  |  | 0 | 1 |
| HAM/TSP#1 | 105 | chr1_221371554_+ |  |  |  |  | 0 | 1 |
| HAM/TSP#1 | 106 | chr1_223048949_+ |  |  |  |  | 0 | 1 |
| HAM/TSP#1 | 107 | chr1_22340517_- |  |  |  |  | 0 | 1 |
| HAM/TSP#1 | 108 | chr1_223756407_- |  |  |  |  | 0 | 3 |
| HAM/TSP#1 | 109 | chr1_2251855_+ |  |  |  |  | 2 | 37 |
| HAM/TSP#1 | 110 | chr1_226482935_+ |  |  |  |  | 0 | 1 |
| HAM/TSP#1 | 111 | chr1_227093261_- |  |  |  |  | 0 | 1 |
| HAM/TSP#1 | 112 | chr1_227285347_+ |  |  |  |  | 0 | 1 |
| HAM/TSP#1 | 113 | chr1_230275968_- |  |  |  |  | 0 | 1 |
| HAM/TSP#1 | 114 | chr1_231193125_+ |  |  |  |  | 0 | 1 |
| HAM/TSP#1 | 115 | chr1_23215753_+ |  |  |  |  | 0 | 1 |
| HAM/TSP#1 | 116 | chr1_232296643_+ |  |  |  |  | 0 | 1 |
| HAM/TSP#1 | 117 | chr1_232924289_+ |  |  |  |  | 0 | 5 |
| HAM/TSP#1 | 118 | chr1_233173267_+ |  |  |  |  | 0 | 1 |
| HAM/TSP#1 | 119 | chr1_235120346_+ |  |  |  |  | 0 | 1 |
| HAM/TSP#1 | 120 | chr1_235422849_- |  |  |  |  | 0 | 1 |
| HAM/TSP#1 | 121 | chr1_24120005_+ |  |  |  |  | 0 | 1 |
| HAM/TSP#1 | 122 | chr1_241652621_+ |  |  |  |  | 0 | 1 |
| HAM/TSP#1 | 123 | chr1_241934692_- |  |  |  |  | 0 | 1 |
| HAM/TSP#1 | 124 | chr1_243828213_- |  |  |  |  | 0 | 1 |
| HAM/TSP#1 | 125 | chr1_24496742_- |  |  |  |  | 0 | 2 |
| HAM/TSP#1 | 126 | chr1_246346601_+ |  |  |  |  | 0 | 1 |
| HAM/TSP#1 | 127 | chr1_247185347_- |  |  |  |  | 4 | 0 |
| HAM/TSP#1 | 128 | chr1_247822511_- |  |  |  |  | 0 | 1 |
| HAM/TSP#1 | 129 | chr1_247935419_- |  |  |  |  | 0 | 6 |
| HAM/TSP#1 | 130 | chr1_248861478_- |  |  |  |  | 0 | 7 |
| HAM/TSP#1 | 131 | chr1_25359055_+ |  |  |  |  | 0 | 1 |
| HAM/TSP#1 | 132 | chr1_25753335_+ |  |  |  |  | 0 | 1 |
| HAM/TSP#1 | 133 | chr1_28538307_+ |  |  |  |  | 0 | 1 |
| HAM/TSP#1 | 134 | chr1_28591786_- |  |  |  |  | 0 | 1 |
| HAM/TSP#1 | 135 | chr1_30323677_- |  |  |  |  | 0 | 1 |
| HAM/TSP#1 | 136 | chr1_32078586_- |  |  |  |  | 0 | 14 |
| HAM/TSP#1 | 137 | chr1_32424382_+ |  |  |  |  | 0 | 1 |
| HAM/TSP#1 | 138 | chr1_3285800_+ |  |  |  |  | 0 | 1 |
| HAM/TSP#1 | 139 | chr1_32924469_- |  |  |  |  | 0 | 1 |
| HAM/TSP#1 | 140 | chr1_33293725_+ |  |  |  |  | 0 | 1 |
| HAM/TSP#1 | 141 | chr1_33510089_- |  |  |  |  | 0 | 1 |
| HAM/TSP#1 | 142 | chr1_33662228_- |  |  |  |  | 0 | 1 |
| HAM/TSP#1 | 143 | chr1_33705441_- |  |  |  |  | 0 | 1 |
| HAM/TSP#1 | 144 | chr1_34518878_- |  |  |  |  | 0 | 1 |
| HAM/TSP#1 | 145 | chr1_3497733_+ |  |  |  |  | 0 | 1 |
| HAM/TSP#1 | 146 | chr1_3497897_+ |  |  |  |  | 0 | 1 |
| HAM/TSP#1 | 147 | chr1_34986488_+ |  |  |  |  | 0 | 1 |
| HAM/TSP#1 | 148 | chr1_36461171_- |  |  |  |  | 0 | 3 |
| HAM/TSP#1 | 149 | chr1_36497078_+ |  |  |  |  | 0 | 1 |
| HAM/TSP#1 | 150 | chr1_38679375_+ |  |  |  |  | 0 | 1 |
| HAM/TSP#1 | 151 | chr1_38781907_- |  |  |  |  | 0 | 1 |
| HAM/TSP#1 | 152 | chr1_43148750_+ |  |  |  |  | 0 | 1 |
| HAM/TSP#1 | 153 | chr1_43976191_- |  |  |  |  | 0 | 1 |
| HAM/TSP#1 | 154 | chr1_45673316_+ |  |  |  |  | 0 | 1 |
| HAM/TSP#1 | 155 | chr1_46135388_+ |  |  |  |  | 0 | 1 |
| HAM/TSP#1 | 156 | chr1_46189665_- |  |  |  |  | 0 | 7 |
| HAM/TSP#1 | 157 | chr1_47415252_- |  |  |  |  | 0 | 1 |
| HAM/TSP#1 | 158 | chr1_48646996_- |  |  |  |  | 0 | 1 |
| HAM/TSP#1 | 159 | chr1_48853812_+ |  |  |  |  | 0 | 1 |
| HAM/TSP#1 | 160 | chr1_48871246_- |  |  |  |  | 0 | 1 |
| HAM/TSP#1 | 161 | chr1_49441338_+ |  |  |  |  | 0 | 1 |
| HAM/TSP#1 | 162 | chr1_49520681_- |  |  |  |  | 0 | 1 |
| HAM/TSP#1 | 163 | chr1_4997894_- |  |  |  |  | 0 | 1 |
| HAM/TSP#1 | 164 | chr1_50181219_+ |  |  |  |  | 0 | 1 |
| HAM/TSP#1 | 165 | chr1_50945004_+ |  |  |  |  | 0 | 7 |
| HAM/TSP#1 | 166 | chr1_51726253_+ |  |  |  |  | 0 | 6 |
| HAM/TSP#1 | 167 | chr1_5194047_+ |  |  |  |  | 0 | 1 |
| HAM/TSP#1 | 168 | chr1_5236222_- |  |  |  |  | 0 | 3 |
| HAM/TSP#1 | 169 | chr1_52514850_+ |  |  |  |  | 0 | 1 |
| HAM/TSP#1 | 170 | chr1_5387379_- |  |  |  |  | 0 | 1 |
| HAM/TSP#1 | 171 | chr1_5502969_+ |  |  |  |  | 0 | 1 |
| HAM/TSP#1 | 172 | chr1_55488211_- |  |  |  |  | 0 | 1 |
| HAM/TSP#1 | 173 | chr1_5575913_- |  |  |  |  | 0 | 1 |
| HAM/TSP#1 | 174 | chr1_56048783_- |  |  |  |  | 0 | 3 |
| HAM/TSP#1 | 175 | chr1_57363630_+ |  |  |  |  | 0 | 2 |
| HAM/TSP#1 | 176 | chr1_59696717_- |  |  |  |  | 0 | 1 |
| HAM/TSP#1 | 177 | chr1_6167856_+ |  |  |  |  | 0 | 1 |
| HAM/TSP#1 | 178 | chr1_632132_- |  |  |  |  | 0 | 1 |
| HAM/TSP#1 | 179 | chr1_63619412_- |  |  |  |  | 0 | 1 |
| HAM/TSP#1 | 180 | chr1_63682682_- |  |  |  |  | 0 | 2 |
| HAM/TSP#1 | 181 | chr1_64778853_- |  |  |  |  | 0 | 1 |
| HAM/TSP#1 | 182 | chr1_65210279_- |  |  |  |  | 0 | 1 |
| HAM/TSP#1 | 183 | chr1_67261918_+ |  |  |  |  | 0 | 10 |
| HAM/TSP#1 | 184 | chr1_67997936_+ |  |  |  |  | 0 | 1 |
| HAM/TSP#1 | 185 | chr1_68784916_- |  |  |  |  | 0 | 1 |
| HAM/TSP#1 | 186 | chr1_69047351_+ |  |  |  |  | 0 | 1 |
| HAM/TSP#1 | 187 | chr1_69819797_- |  |  |  |  | 0 | 1 |
| HAM/TSP#1 | 188 | chr1_71105524_+ |  |  |  |  | 0 | 3 |
| HAM/TSP#1 | 189 | chr1_74654063_- |  |  |  |  | 0 | 1 |
| HAM/TSP#1 | 190 | chr1_7584743_- |  |  |  |  | 0 | 1 |
| HAM/TSP#1 | 191 | chr1_7595762_+ |  |  |  |  | 0 | 1 |
| HAM/TSP#1 | 192 | chr1_76903314_- |  |  |  |  | 0 | 6 |
| HAM/TSP#1 | 193 | chr1_77340338_- |  |  |  |  | 0 | 1 |
| HAM/TSP#1 | 194 | chr1_81352337_- |  |  |  |  | 0 | 1 |
| HAM/TSP#1 | 195 | chr1_87564542_- |  |  |  |  | 0 | 1 |
| HAM/TSP#1 | 196 | chr1_88425369_- |  |  |  |  | 0 | 1 |
| HAM/TSP#1 | 197 | chr1_90556742_- |  |  |  |  | 0 | 1 |
| HAM/TSP#1 | 198 | chr1_90593704_- |  |  |  |  | 0 | 18 |
| HAM/TSP#1 | 199 | chr1_91155836_- |  |  |  |  | 0 | 1 |
| HAM/TSP#1 | 200 | chr1_91320775_- |  |  |  |  | 0 | 7 |
| HAM/TSP#1 | 201 | chr1_91595148_- |  |  |  |  | 0 | 1 |
| HAM/TSP#1 | 202 | chr1_92464777_+ |  |  |  |  | 0 | 1 |
| HAM/TSP#1 | 203 | chr1_92981373_+ |  |  |  |  | 0 | 1 |
| HAM/TSP#1 | 204 | chr1_9375137_+ |  |  |  |  | 0 | 3 |
| HAM/TSP#1 | 205 | chr1_9389091_- |  |  |  |  | 0 | 1 |
| HAM/TSP#1 | 206 | chr1_94311188_+ |  |  |  |  | 0 | 1 |
| HAM/TSP#1 | 207 | chr1_94439837_+ |  |  |  |  | 0 | 1 |
| HAM/TSP#1 | 208 | chr1_97061442_+ |  |  |  |  | 0 | 1 |
| HAM/TSP#1 | 209 | chr1_97348569_+ |  |  |  |  | 0 | 1 |
| HAM/TSP#1 | 210 | chr1_98112994_- |  |  |  |  | 0 | 3 |
| HAM/TSP#1 | 211 | chr1_98904875_- |  |  |  |  | 0 | 2 |
| HAM/TSP#1 | 212 | chr1_99020958_+ |  |  |  |  | 0 | 1 |
| HAM/TSP#1 | 213 | chr1_995965_- |  |  |  |  | 0 | 3 |
| HAM/TSP#1 | 214 | chr1_99692937_- |  |  |  |  | 0 | 1 |
| HAM/TSP#1 | 215 | chr2_101121431_+ |  |  |  |  | 0 | 12 |
| HAM/TSP#1 | 216 | chr2_101378299_+ |  |  |  |  | 0 | 1 |
| HAM/TSP#1 | 217 | chr2_101860188_+ |  |  |  |  | 0 | 3 |
| HAM/TSP#1 | 218 | chr2_102048917_+ |  |  |  |  | 0 | 1 |
| HAM/TSP#1 | 219 | chr2_102775828_+ |  |  |  |  | 0 | 4 |
| HAM/TSP#1 | 220 | chr2_103106296_- |  |  |  |  | 0 | 3 |
| HAM/TSP#1 | 221 | chr2_10387305_- |  |  |  |  | 0 | 1 |
| HAM/TSP#1 | 222 | chr2_106090470_+ |  |  |  |  | 0 | 1 |
| HAM/TSP#1 | 223 | chr2_106104572_- |  |  |  |  | 0 | 1 |
| HAM/TSP#1 | 224 | chr2_107723188_- |  |  |  |  | 0 | 4 |
| HAM/TSP#1 | 225 | chr2_109199662_- |  |  |  |  | 0 | 11 |
| HAM/TSP#1 | 226 | chr2_109591716_- |  |  |  |  | 0 | 1 |
| HAM/TSP#1 | 227 | chr2_110177774_+ |  |  |  |  | 0 | 1 |
| HAM/TSP#1 | 228 | chr2_110210093_+ |  |  |  |  | 0 | 1 |
| HAM/TSP#1 | 229 | chr2_111665420_+ |  |  |  |  | 0 | 2 |
| HAM/TSP#1 | 230 | chr2_111687170_+ |  |  |  |  | 0 | 1 |
| HAM/TSP#1 | 231 | chr2_112512941_+ |  |  |  |  | 0 | 2 |
| HAM/TSP#1 | 232 | chr2_114328458_- |  |  |  |  | 0 | 2 |
| HAM/TSP#1 | 233 | chr2_115296229_+ |  |  |  |  | 0 | 1 |
| HAM/TSP#1 | 234 | chr2_116095500_- |  |  |  |  | 0 | 2 |
| HAM/TSP#1 | 235 | chr2_116287097_- |  |  |  |  | 0 | 1 |
| HAM/TSP#1 | 236 | chr2_116456804_+ |  |  |  |  | 0 | 1 |
| HAM/TSP#1 | 237 | chr2_116585540_- |  |  |  |  | 0 | 2 |
| HAM/TSP#1 | 238 | chr2_116649751_- |  |  |  |  | 0 | 1 |
| HAM/TSP#1 | 239 | chr2_116760045_+ |  |  |  |  | 0 | 1 |
| HAM/TSP#1 | 240 | chr2_117629035_+ |  |  |  |  | 0 | 5 |
| HAM/TSP#1 | 241 | chr2_118202532_- |  |  |  |  | 0 | 14 |
| HAM/TSP#1 | 242 | chr2_118320931_- |  |  |  |  | 0 | 1 |
| HAM/TSP#1 | 243 | chr2_119264844_- |  |  |  |  | 0 | 1 |
| HAM/TSP#1 | 244 | chr2_119574759_- |  |  |  |  | 0 | 1 |
| HAM/TSP#1 | 245 | chr2_122046616_+ |  |  |  |  | 0 | 1 |
| HAM/TSP#1 | 246 | chr2_123208124_- |  |  |  |  | 0 | 1 |
| HAM/TSP#1 | 247 | chr2_123576202_+ |  |  |  |  | 0 | 1 |
| HAM/TSP#1 | 248 | chr2_124778606_- |  |  |  |  | 0 | 1 |
| HAM/TSP#1 | 249 | chr2_125389657_+ |  |  |  |  | 0 | 1 |
| HAM/TSP#1 | 250 | chr2_12649706_+ |  |  |  |  | 0 | 1 |
| HAM/TSP#1 | 251 | chr2_128086571_+ |  |  |  |  | 0 | 4 |
| HAM/TSP#1 | 252 | chr2_128886951_- |  |  |  |  | 0 | 1 |
| HAM/TSP#1 | 253 | chr2_130760876_+ |  |  |  |  | 0 | 1 |
| HAM/TSP#1 | 254 | chr2_131037174_- |  |  |  |  | 0 | 1 |
| HAM/TSP#1 | 255 | chr2_133001473_- |  |  |  |  | 0 | 2 |
| HAM/TSP#1 | 256 | chr2_134367086_+ |  |  |  |  | 0 | 1 |
| HAM/TSP#1 | 257 | chr2_134460730_+ |  |  |  |  | 0 | 2 |
| HAM/TSP#1 | 258 | chr2_134893744_+ |  |  |  |  | 0 | 15 |
| HAM/TSP#1 | 259 | chr2_136259234_+ |  |  |  |  | 0 | 1 |
| HAM/TSP#1 | 260 | chr2_136314340_- |  |  |  |  | 0 | 1 |
| HAM/TSP#1 | 261 | chr2_136344661_- |  |  |  |  | 0 | 1 |
| HAM/TSP#1 | 262 | chr2_136728164_- |  |  |  |  | 0 | 2 |
| HAM/TSP#1 | 263 | chr2_138555383_+ |  |  |  |  | 0 | 2 |
| HAM/TSP#1 | 264 | chr2_1397777_+ |  |  |  |  | 0 | 1 |
| HAM/TSP#1 | 265 | chr2_140319281_+ |  |  |  |  | 0 | 2 |
| HAM/TSP#1 | 266 | chr2_141555048_+ |  |  |  |  | 0 | 1 |
| HAM/TSP#1 | 267 | chr2_143109542_+ |  |  |  |  | 0 | 1 |
| HAM/TSP#1 | 268 | chr2_147674975_+ |  |  |  |  | 0 | 4 |
| HAM/TSP#1 | 269 | chr2_147696127_- |  |  |  |  | 0 | 1 |
| HAM/TSP#1 | 270 | chr2_147698578_- |  |  |  |  | 0 | 1 |
| HAM/TSP#1 | 271 | chr2_150463100_+ |  |  |  |  | 0 | 3 |
| HAM/TSP#1 | 272 | chr2_151819222_+ |  |  |  |  | 0 | 1 |
| HAM/TSP#1 | 273 | chr2_151896004_- |  |  |  |  | 0 | 8 |
| HAM/TSP#1 | 274 | chr2_15265410_- |  |  |  |  | 0 | 1 |
| HAM/TSP#1 | 275 | chr2_152760010_+ |  |  |  |  | 0 | 1 |
| HAM/TSP#1 | 276 | chr2_153197634_- |  |  |  |  | 0 | 1 |
| HAM/TSP#1 | 277 | chr2_154531477_+ |  |  |  |  | 0 | 3 |
| HAM/TSP#1 | 278 | chr2_15759014_+ |  |  |  |  | 0 | 1 |
| HAM/TSP#1 | 279 | chr2_158924716_- |  |  |  |  | 0 | 1 |
| HAM/TSP#1 | 280 | chr2_160576703_+ |  |  |  |  | 0 | 1 |
| HAM/TSP#1 | 281 | chr2_160798882_- |  |  |  |  | 0 | 1 |
| HAM/TSP#1 | 282 | chr2_16216250_+ |  |  |  |  | 0 | 1 |
| HAM/TSP#1 | 283 | chr2_164106310_- |  |  |  |  | 0 | 1 |
| HAM/TSP#1 | 284 | chr2_168385425_+ |  |  |  |  | 0 | 11 |
| HAM/TSP#1 | 285 | chr2_168821725_+ |  |  |  |  | 0 | 1 |
| HAM/TSP#1 | 286 | chr2_171114481_- |  |  |  |  | 0 | 1 |
| HAM/TSP#1 | 287 | chr2_17187272_- |  |  |  |  | 0 | 1 |
| HAM/TSP#1 | 288 | chr2_173691191_- |  |  |  |  | 0 | 1 |
| HAM/TSP#1 | 289 | chr2_176279314_- |  |  |  |  | 0 | 1 |
| HAM/TSP#1 | 290 | chr2_176840417_- |  |  |  |  | 0 | 1 |
| HAM/TSP#1 | 291 | chr2_176847558_+ |  |  |  |  | 0 | 1 |
| HAM/TSP#1 | 292 | chr2_177410794_- |  |  |  |  | 0 | 1 |
| HAM/TSP#1 | 293 | chr2_179028265_+ |  |  |  |  | 0 | 13 |
| HAM/TSP#1 | 294 | chr2_180276526_- |  |  |  |  | 0 | 1 |
| HAM/TSP#1 | 295 | chr2_181512954_+ |  |  |  |  | 0 | 1 |
| HAM/TSP#1 | 296 | chr2_182741617_- |  |  |  |  | 0 | 1 |
| HAM/TSP#1 | 297 | chr2_185557363_- |  |  |  |  | 0 | 1 |
| HAM/TSP#1 | 298 | chr2_186436938_- |  |  |  |  | 0 | 1 |
| HAM/TSP#1 | 299 | chr2_189119248_- |  |  |  |  | 0 | 1 |
| HAM/TSP#1 | 300 | chr2_189227090_+ |  |  |  |  | 0 | 1 |
| HAM/TSP#1 | 301 | chr2_189474020_+ |  |  |  |  | 0 | 3 |
| HAM/TSP#1 | 302 | chr2_18960187_+ |  |  |  |  | 0 | 1 |
| HAM/TSP#1 | 303 | chr2_189923772_- |  |  |  |  | 0 | 1 |
| HAM/TSP#1 | 304 | chr2_190267099_+ |  |  |  |  | 0 | 1 |
| HAM/TSP#1 | 305 | chr2_190488386_+ |  |  |  |  | 0 | 4 |
| HAM/TSP#1 | 306 | chr2_192314635_- |  |  |  |  | 0 | 1 |
| HAM/TSP#1 | 307 | chr2_195144813_+ |  |  |  |  | 0 | 4 |
| HAM/TSP#1 | 308 | chr2_197484014_- |  |  |  |  | 0 | 4 |
| HAM/TSP#1 | 309 | chr2_197710725_- |  |  |  |  | 0 | 1 |
| HAM/TSP#1 | 310 | chr2_197724290_- |  |  |  |  | 0 | 1 |
| HAM/TSP#1 | 311 | chr2_199584452_- |  |  |  |  | 0 | 1 |
| HAM/TSP#1 | 312 | chr2_202832654_- |  |  |  |  | 0 | 4 |
| HAM/TSP#1 | 313 | chr2_203631988_- |  |  |  |  | 0 | 2 |
| HAM/TSP#1 | 314 | chr2_20433314_- |  |  |  |  | 0 | 1 |
| HAM/TSP#1 | 315 | chr2_204381220_- |  |  |  |  | 0 | 1 |
| HAM/TSP#1 | 316 | chr2_207081949_+ |  |  |  |  | 0 | 1 |
| HAM/TSP#1 | 317 | chr2_210056482_- |  |  |  |  | 0 | 1 |
| HAM/TSP#1 | 318 | chr2_212949944_- |  |  |  |  | 0 | 1 |
| HAM/TSP#1 | 319 | chr2_214143061_+ |  |  |  |  | 0 | 1 |
| HAM/TSP#1 | 320 | chr2_216687326_- |  |  |  |  | 0 | 1 |
| HAM/TSP#1 | 321 | chr2_217600633_- |  |  |  |  | 0 | 9 |
| HAM/TSP#1 | 322 | chr2_219464316_- |  |  |  |  | 0 | 1 |
| HAM/TSP#1 | 323 | chr2_219602104_- |  |  |  |  | 0 | 1 |
| HAM/TSP#1 | 324 | chr2_223590859_- |  |  |  |  | 0 | 1 |
| HAM/TSP#1 | 325 | chr2_224628380_+ |  |  |  |  | 0 | 1 |
| HAM/TSP#1 | 326 | chr2_225233547_- |  |  |  |  | 0 | 1 |
| HAM/TSP#1 | 327 | chr2_226320956_- |  |  |  |  | 0 | 1 |
| HAM/TSP#1 | 328 | chr2_227178240_+ |  |  |  |  | 0 | 1 |
| HAM/TSP#1 | 329 | chr2_227460661_+ |  |  |  |  | 0 | 12 |
| HAM/TSP#1 | 330 | chr2_227460662_+ |  |  |  |  | 0 | 1 |
| HAM/TSP#1 | 331 | chr2_231500696_+ |  |  |  |  | 0 | 1 |
| HAM/TSP#1 | 332 | chr2_232486618_+ |  |  |  |  | 0 | 4 |
| HAM/TSP#1 | 333 | chr2_232541787_- |  |  |  |  | 0 | 1 |
| HAM/TSP#1 | 334 | chr2_233626950_+ |  |  |  |  | 0 | 2 |
| HAM/TSP#1 | 335 | chr2_233922696_- |  |  |  |  | 0 | 1 |
| HAM/TSP#1 | 336 | chr2_234104717_- |  |  |  |  | 0 | 1 |
| HAM/TSP#1 | 337 | chr2_234904345_+ |  |  |  |  | 0 | 1 |
| HAM/TSP#1 | 338 | chr2_235882302_- |  |  |  |  | 0 | 1 |
| HAM/TSP#1 | 339 | chr2_236586459_+ |  |  |  |  | 0 | 1 |
| HAM/TSP#1 | 340 | chr2_236624519_+ |  |  |  |  | 0 | 1 |
| HAM/TSP#1 | 341 | chr2_238107073_+ |  |  |  |  | 0 | 1 |
| HAM/TSP#1 | 342 | chr2_23855635_+ |  |  |  |  | 0 | 1 |
| HAM/TSP#1 | 343 | chr2_239024379_+ |  |  |  |  | 0 | 1 |
| HAM/TSP#1 | 344 | chr2_239053085_- |  |  |  |  | 0 | 1 |
| HAM/TSP#1 | 345 | chr2_239109596_+ |  |  |  |  | 0 | 1 |
| HAM/TSP#1 | 346 | chr2_239994440_- |  |  |  |  | 0 | 1 |
| HAM/TSP#1 | 347 | chr2_240769222_- |  |  |  |  | 0 | 1 |
| HAM/TSP#1 | 348 | chr2_241746999_+ |  |  |  |  | 0 | 5 |
| HAM/TSP#1 | 349 | chr2_242160265_- |  |  |  |  | 0 | 1 |
| HAM/TSP#1 | 350 | chr2_25026417_- |  |  |  |  | 0 | 1 |
| HAM/TSP#1 | 351 | chr2_25211019_- |  |  |  |  | 0 | 13 |
| HAM/TSP#1 | 352 | chr2_2586262_- |  |  |  |  | 0 | 3 |
| HAM/TSP#1 | 353 | chr2_26640498_- |  |  |  |  | 0 | 1 |
| HAM/TSP#1 | 354 | chr2_27025926_- |  |  |  |  | 0 | 1 |
| HAM/TSP#1 | 355 | chr2_27100985_- |  |  |  |  | 0 | 1 |
| HAM/TSP#1 | 356 | chr2_27213526_- |  |  |  |  | 0 | 4 |
| HAM/TSP#1 | 357 | chr2_27242742_- |  |  |  |  | 0 | 1 |
| HAM/TSP#1 | 358 | chr2_28372143_+ |  |  |  |  | 0 | 1 |
| HAM/TSP#1 | 359 | chr2_29902101_+ |  |  |  |  | 0 | 1 |
| HAM/TSP#1 | 360 | chr2_30367486_- |  |  |  |  | 0 | 1 |
| HAM/TSP#1 | 361 | chr2_31556913_- |  |  |  |  | 0 | 1 |
| HAM/TSP#1 | 362 | chr2_32136064_- |  |  |  |  | 0 | 3 |
| HAM/TSP#1 | 363 | chr2_33957319_- |  |  |  |  | 0 | 1 |
| HAM/TSP#1 | 364 | chr2_36046009_- |  |  |  |  | 0 | 1 |
| HAM/TSP#1 | 365 | chr2_36123096_- |  |  |  |  | 0 | 1 |
| HAM/TSP#1 | 366 | chr2_36972889_+ |  |  |  |  | 0 | 1 |
| HAM/TSP#1 | 367 | chr2_38186228_+ |  |  |  |  | 0 | 3 |
| HAM/TSP#1 | 368 | chr2_39169979_- |  |  |  |  | 0 | 1 |
| HAM/TSP#1 | 369 | chr2_39665427_+ |  |  |  |  | 0 | 1 |
| HAM/TSP#1 | 370 | chr2_40178286_+ |  |  |  |  | 0 | 9 |
| HAM/TSP#1 | 371 | chr2_44163038_+ |  |  |  |  | 0 | 4 |
| HAM/TSP#1 | 372 | chr2_44361236_- |  |  |  |  | 0 | 1 |
| HAM/TSP#1 | 373 | chr2_44605242_+ |  |  |  |  | 0 | 1 |
| HAM/TSP#1 | 374 | chr2_44899536_- |  |  |  |  | 0 | 1 |
| HAM/TSP#1 | 375 | chr2_47092758_+ |  |  |  |  | 0 | 1 |
| HAM/TSP#1 | 376 | chr2_47731054_+ |  |  |  |  | 0 | 1 |
| HAM/TSP#1 | 377 | chr2_47915282_- |  |  |  |  | 0 | 15 |
| HAM/TSP#1 | 378 | chr2_484305_+ |  |  |  |  | 0 | 1 |
| HAM/TSP#1 | 379 | chr2_50072311_- |  |  |  |  | 0 | 1 |
| HAM/TSP#1 | 380 | chr2_50417324_- |  |  |  |  | 1 | 0 |
| HAM/TSP#1 | 381 | chr2_50666223_- |  |  |  |  | 0 | 1 |
| HAM/TSP#1 | 382 | chr2_50843049_- |  |  |  |  | 0 | 23 |
| HAM/TSP#1 | 383 | chr2_51485663_+ |  |  |  |  | 0 | 1 |
| HAM/TSP#1 | 384 | chr2_51953556_- |  |  |  |  | 0 | 1 |
| HAM/TSP#1 | 385 | chr2_52126809_+ |  |  |  |  | 0 | 3 |
| HAM/TSP#1 | 386 | chr2_53183910_- |  |  |  |  | 0 | 3 |
| HAM/TSP#1 | 387 | chr2_55521197_- |  |  |  |  | 0 | 1 |
| HAM/TSP#1 | 388 | chr2_5915378_- |  |  |  |  | 0 | 1 |
| HAM/TSP#1 | 389 | chr2_59948614_+ |  |  |  |  | 0 | 2 |
| HAM/TSP#1 | 390 | chr2_60057446_- |  |  |  |  | 0 | 1 |
| HAM/TSP#1 | 391 | chr2_6052098_+ |  |  |  |  | 0 | 1 |
| HAM/TSP#1 | 392 | chr2_60779721_- |  |  |  |  | 0 | 2 |
| HAM/TSP#1 | 393 | chr2_62102421_+ |  |  |  |  | 0 | 1 |
| HAM/TSP#1 | 394 | chr2_62605193_+ |  |  |  |  | 0 | 1 |
| HAM/TSP#1 | 395 | chr2_633736_- |  |  |  |  | 0 | 16 |
| HAM/TSP#1 | 396 | chr2_6337638_- |  |  |  |  | 0 | 1 |
| HAM/TSP#1 | 397 | chr2_640651_- |  |  |  |  | 0 | 1 |
| HAM/TSP#1 | 398 | chr2_64108047_- |  |  |  |  | 0 | 1 |
| HAM/TSP#1 | 399 | chr2_65296489_- |  |  |  |  | 0 | 1 |
| HAM/TSP#1 | 400 | chr2_65501949_- |  |  |  |  | 0 | 1 |
| HAM/TSP#1 | 401 | chr2_66109412_+ |  |  |  |  | 0 | 1 |
| HAM/TSP#1 | 402 | chr2_66312891_+ |  |  |  |  | 0 | 1 |
| HAM/TSP#1 | 403 | chr2_66898378_- |  |  |  |  | 0 | 3 |
| HAM/TSP#1 | 404 | chr2_67305764_- |  |  |  |  | 0 | 1 |
| HAM/TSP#1 | 405 | chr2_68721734_+ |  |  |  |  | 0 | 1 |
| HAM/TSP#1 | 406 | chr2_6895981_+ |  |  |  |  | 0 | 1 |
| HAM/TSP#1 | 407 | chr2_69439740_+ |  |  |  |  | 0 | 1 |
| HAM/TSP#1 | 408 | chr2_69804212_- |  |  |  |  | 0 | 3 |
| HAM/TSP#1 | 409 | chr2_71022869_- |  |  |  |  | 0 | 1 |
| HAM/TSP#1 | 410 | chr2_71162911_+ |  |  |  |  | 0 | 1 |
| HAM/TSP#1 | 411 | chr2_71648946_- |  |  |  |  | 0 | 1 |
| HAM/TSP#1 | 412 | chr2_71910790_- |  |  |  |  | 0 | 3 |
| HAM/TSP#1 | 413 | chr2_72023186_+ |  |  |  |  | 0 | 5 |
| HAM/TSP#1 | 414 | chr2_72124012_+ |  |  |  |  | 0 | 2 |
| HAM/TSP#1 | 415 | chr2_72534358_+ |  |  |  |  | 0 | 1 |
| HAM/TSP#1 | 416 | chr2_74445888_- |  |  |  |  | 0 | 1 |
| HAM/TSP#1 | 417 | chr2_75475128_- |  |  |  |  | 0 | 1 |
| HAM/TSP#1 | 418 | chr2_76555505_+ |  |  |  |  | 0 | 1 |
| HAM/TSP#1 | 419 | chr2_7740463_+ |  |  |  |  | 0 | 1 |
| HAM/TSP#1 | 420 | chr2_78152601_+ |  |  |  |  | 0 | 1 |
| HAM/TSP#1 | 421 | chr2_79733030_- |  |  |  |  | 0 | 1 |
| HAM/TSP#1 | 422 | chr2_80299490_- |  |  |  |  | 0 | 6 |
| HAM/TSP#1 | 423 | chr2_81095321_+ |  |  |  |  | 0 | 1 |
| HAM/TSP#1 | 424 | chr2_81197479_+ |  |  |  |  | 0 | 1 |
| HAM/TSP#1 | 425 | chr2_81756461_- |  |  |  |  | 0 | 1 |
| HAM/TSP#1 | 426 | chr2_8220732_- |  |  |  |  | 0 | 1 |
| HAM/TSP#1 | 427 | chr2_82887724_+ |  |  |  |  | 1 | 5 |
| HAM/TSP#1 | 428 | chr2_8342889_+ |  |  |  |  | 0 | 1 |
| HAM/TSP#1 | 429 | chr2_84128583_- |  |  |  |  | 0 | 1 |
| HAM/TSP#1 | 430 | chr2_85062119_+ |  |  |  |  | 0 | 1 |
| HAM/TSP#1 | 431 | chr2_85304870_+ |  |  |  |  | 0 | 1 |
| HAM/TSP#1 | 432 | chr2_86103663_- |  |  |  |  | 0 | 1 |
| HAM/TSP#1 | 433 | chr2_86608143_- |  |  |  |  | 0 | 1 |
| HAM/TSP#1 | 434 | chr2_88170283_- |  |  |  |  | 0 | 2 |
| HAM/TSP#1 | 435 | chr2_88205918_- |  |  |  |  | 0 | 1 |
| HAM/TSP#1 | 436 | chr2_89831074_- |  |  |  |  | 0 | 1 |
| HAM/TSP#1 | 437 | chr2_9124289_- |  |  |  |  | 0 | 1 |
| HAM/TSP#1 | 438 | chr2_9715062_+ |  |  |  |  | 0 | 3 |
| HAM/TSP#1 | 439 | chr2_97626863_+ |  |  |  |  | 0 | 1 |
| HAM/TSP#1 | 440 | chr2_98233547_+ |  |  |  |  | 0 | 1 |
| HAM/TSP#1 | 441 | chr2_99651314_+ |  |  |  |  | 0 | 1 |
| HAM/TSP#1 | 442 | chr3_100866225_+ |  |  |  |  | 0 | 1 |
| HAM/TSP#1 | 443 | chr3_100962065_+ |  |  |  |  | 0 | 1 |
| HAM/TSP#1 | 444 | chr3_100965025_+ |  |  |  |  | 0 | 1 |
| HAM/TSP#1 | 445 | chr3_101540210_+ |  |  |  |  | 0 | 2 |
| HAM/TSP#1 | 446 | chr3_101540211_+ |  |  |  |  | 0 | 2 |
| HAM/TSP#1 | 447 | chr3_101772500_+ |  |  |  |  | 0 | 4 |
| HAM/TSP#1 | 448 | chr3_101847000_+ |  |  |  |  | 0 | 6 |
| HAM/TSP#1 | 449 | chr3_101944675_- |  |  |  |  | 0 | 4 |
| HAM/TSP#1 | 450 | chr3_102393123_+ |  |  |  |  | 0 | 1 |
| HAM/TSP#1 | 451 | chr3_102455927_+ |  |  |  |  | 0 | 2 |
| HAM/TSP#1 | 452 | chr3_102663873_- |  |  |  |  | 0 | 1 |
| HAM/TSP#1 | 453 | chr3_10288683_- |  |  |  |  | 0 | 3 |
| HAM/TSP#1 | 454 | chr3_103093933_- |  |  |  |  | 0 | 1 |
| HAM/TSP#1 | 455 | chr3_10399306_- |  |  |  |  | 0 | 1 |
| HAM/TSP#1 | 456 | chr3_106638324_+ |  |  |  |  | 0 | 1 |
| HAM/TSP#1 | 457 | chr3_106683124_+ |  |  |  |  | 0 | 1 |
| HAM/TSP#1 | 458 | chr3_106683125_+ |  |  |  |  | 0 | 3 |
| HAM/TSP#1 | 459 | chr3_108243684_- |  |  |  |  | 0 | 13 |
| HAM/TSP#1 | 460 | chr3_108939253_- |  |  |  |  | 0 | 1 |
| HAM/TSP#1 | 461 | chr3_10949015_+ |  |  |  |  | 0 | 1 |
| HAM/TSP#1 | 462 | chr3_111760926_+ |  |  |  |  | 0 | 1 |
| HAM/TSP#1 | 463 | chr3_112198665_- |  |  |  |  | 0 | 2 |
| HAM/TSP#1 | 464 | chr3_112329780_- |  |  |  |  | 0 | 1 |
| HAM/TSP#1 | 465 | chr3_112756387_- |  |  |  |  | 0 | 1 |
| HAM/TSP#1 | 466 | chr3_114275407_- |  |  |  |  | 0 | 7 |
| HAM/TSP#1 | 467 | chr3_114698607_+ |  |  |  |  | 0 | 1 |
| HAM/TSP#1 | 468 | chr3_11882024_- |  |  |  |  | 0 | 2 |
| HAM/TSP#1 | 469 | chr3_119340545_- |  |  |  |  | 0 | 1 |
| HAM/TSP#1 | 470 | chr3_122943015_+ |  |  |  |  | 0 | 1 |
| HAM/TSP#1 | 471 | chr3_123956995_- |  |  |  |  | 0 | 1 |
| HAM/TSP#1 | 472 | chr3_123960536_- |  |  |  |  | 0 | 1 |
| HAM/TSP#1 | 473 | chr3_124635301_- |  |  |  |  | 0 | 2 |
| HAM/TSP#1 | 474 | chr3_124961438_- |  |  |  |  | 0 | 1 |
| HAM/TSP#1 | 475 | chr3_125006788_- |  |  |  |  | 0 | 4 |
| HAM/TSP#1 | 476 | chr3_125135020_+ |  |  |  |  | 0 | 1 |
| HAM/TSP#1 | 477 | chr3_12570236_+ |  |  |  |  | 0 | 3 |
| HAM/TSP#1 | 478 | chr3_126855046_+ |  |  |  |  | 0 | 1 |
| HAM/TSP#1 | 479 | chr3_128212882_- |  |  |  |  | 0 | 1 |
| HAM/TSP#1 | 480 | chr3_128338948_- |  |  |  |  | 0 | 1 |
| HAM/TSP#1 | 481 | chr3_128408370_+ |  |  |  |  | 0 | 1 |
| HAM/TSP#1 | 482 | chr3_128503302_+ |  |  |  |  | 16 | 188 |
| HAM/TSP#1 | 483 | chr3_128658288_- |  |  |  |  | 0 | 1 |
| HAM/TSP#1 | 484 | chr3_128670551_- |  |  |  |  | 0 | 1 |
| HAM/TSP#1 | 485 | chr3_129953707_+ |  |  |  |  | 0 | 1 |
| HAM/TSP#1 | 486 | chr3_129956538_- |  |  |  |  | 0 | 1 |
| HAM/TSP#1 | 487 | chr3_132288065_- |  |  |  |  | 0 | 1 |
| HAM/TSP#1 | 488 | chr3_132398794_+ |  |  |  |  | 0 | 5 |
| HAM/TSP#1 | 489 | chr3_133224393_- |  |  |  |  | 0 | 1 |
| HAM/TSP#1 | 490 | chr3_133387166_- |  |  |  |  | 0 | 1 |
| HAM/TSP#1 | 491 | chr3_133391675_- |  |  |  |  | 0 | 1 |
| HAM/TSP#1 | 492 | chr3_134228508_- |  |  |  |  | 0 | 1 |
| HAM/TSP#1 | 493 | chr3_134355483_- |  |  |  |  | 0 | 1 |
| HAM/TSP#1 | 494 | chr3_134643253_- |  |  |  |  | 0 | 4 |
| HAM/TSP#1 | 495 | chr3_134761314_+ |  |  |  |  | 0 | 1 |
| HAM/TSP#1 | 496 | chr3_135550693_- |  |  |  |  | 0 | 1 |
| HAM/TSP#1 | 497 | chr3_135986654_+ |  |  |  |  | 0 | 2 |
| HAM/TSP#1 | 498 | chr3_13707528_- |  |  |  |  | 0 | 1 |
| HAM/TSP#1 | 499 | chr3_138721612_- |  |  |  |  | 0 | 1 |
| HAM/TSP#1 | 500 | chr3_138820488_- |  |  |  |  | 0 | 1 |
| HAM/TSP#1 | 501 | chr3_140331258_+ |  |  |  |  | 0 | 1 |
| HAM/TSP#1 | 502 | chr3_14114875_- |  |  |  |  | 0 | 7 |
| HAM/TSP#1 | 503 | chr3_142440065_+ |  |  |  |  | 0 | 1 |
| HAM/TSP#1 | 504 | chr3_143083467_+ |  |  |  |  | 0 | 1 |
| HAM/TSP#1 | 505 | chr3_143325988_- |  |  |  |  | 0 | 2 |
| HAM/TSP#1 | 506 | chr3_143484938_- |  |  |  |  | 0 | 1 |
| HAM/TSP#1 | 507 | chr3_143517166_+ |  |  |  |  | 0 | 1 |
| HAM/TSP#1 | 508 | chr3_14540742_+ |  |  |  |  | 0 | 1 |
| HAM/TSP#1 | 509 | chr3_147870940_+ |  |  |  |  | 0 | 3 |
| HAM/TSP#1 | 510 | chr3_150858126_+ |  |  |  |  | 0 | 2 |
| HAM/TSP#1 | 511 | chr3_151486828_- |  |  |  |  | 0 | 1 |
| HAM/TSP#1 | 512 | chr3_152374104_- |  |  |  |  | 0 | 1 |
| HAM/TSP#1 | 513 | chr3_153024337_- |  |  |  |  | 0 | 1 |
| HAM/TSP#1 | 514 | chr3_15341255_- |  |  |  |  | 0 | 1 |
| HAM/TSP#1 | 515 | chr3_153747500_+ |  |  |  |  | 0 | 4 |
| HAM/TSP#1 | 516 | chr3_154137871_- |  |  |  |  | 0 | 8 |
| HAM/TSP#1 | 517 | chr3_154148467_- |  |  |  |  | 0 | 1 |
| HAM/TSP#1 | 518 | chr3_157625585_- |  |  |  |  | 0 | 2 |
| HAM/TSP#1 | 519 | chr3_158643364_+ |  |  |  |  | 0 | 1 |
| HAM/TSP#1 | 520 | chr3_16095713_- |  |  |  |  | 0 | 1 |
| HAM/TSP#1 | 521 | chr3_16376109_+ |  |  |  |  | 0 | 5 |
| HAM/TSP#1 | 522 | chr3_165123331_- |  |  |  |  | 0 | 1 |
| HAM/TSP#1 | 523 | chr3_1700506_+ |  |  |  |  | 0 | 1 |
| HAM/TSP#1 | 524 | chr3_170058135_+ |  |  |  |  | 0 | 1 |
| HAM/TSP#1 | 525 | chr3_170217221_+ |  |  |  |  | 0 | 1 |
| HAM/TSP#1 | 526 | chr3_171972415_+ |  |  |  |  | 0 | 1 |
| HAM/TSP#1 | 527 | chr3_172107870_- |  |  |  |  | 0 | 1 |
| HAM/TSP#1 | 528 | chr3_176219497_- |  |  |  |  | 0 | 1 |
| HAM/TSP#1 | 529 | chr3_176624151_- |  |  |  |  | 0 | 3 |
| HAM/TSP#1 | 530 | chr3_177415945_- |  |  |  |  | 0 | 6 |
| HAM/TSP#1 | 531 | chr3_177448173_+ |  |  |  |  | 0 | 1 |
| HAM/TSP#1 | 532 | chr3_177638675_+ |  |  |  |  | 0 | 1 |
| HAM/TSP#1 | 533 | chr3_177920701_+ |  |  |  |  | 0 | 1 |
| HAM/TSP#1 | 534 | chr3_178840771_- |  |  |  |  | 0 | 2 |
| HAM/TSP#1 | 535 | chr3_179055692_- |  |  |  |  | 0 | 6 |
| HAM/TSP#1 | 536 | chr3_179592910_- |  |  |  |  | 0 | 2 |
| HAM/TSP#1 | 537 | chr3_182165352_+ |  |  |  |  | 0 | 1 |
| HAM/TSP#1 | 538 | chr3_183304460_- |  |  |  |  | 0 | 15 |
| HAM/TSP#1 | 539 | chr3_183519804_- |  |  |  |  | 0 | 13 |
| HAM/TSP#1 | 540 | chr3_18439671_- |  |  |  |  | 0 | 1 |
| HAM/TSP#1 | 541 | chr3_185729177_+ |  |  |  |  | 0 | 1 |
| HAM/TSP#1 | 542 | chr3_185881420_- |  |  |  |  | 0 | 2 |
| HAM/TSP#1 | 543 | chr3_186253643_+ |  |  |  |  | 0 | 1 |
| HAM/TSP#1 | 544 | chr3_18667759_- |  |  |  |  | 0 | 2 |
| HAM/TSP#1 | 545 | chr3_187041142_- |  |  |  |  | 0 | 2 |
| HAM/TSP#1 | 546 | chr3_187395615_- |  |  |  |  | 0 | 1 |
| HAM/TSP#1 | 547 | chr3_187929453_- |  |  |  |  | 0 | 1 |
| HAM/TSP#1 | 548 | chr3_189944026_- |  |  |  |  | 0 | 6 |
| HAM/TSP#1 | 549 | chr3_191052239_- |  |  |  |  | 0 | 5 |
| HAM/TSP#1 | 550 | chr3_191662378_+ |  |  |  |  | 0 | 3 |
| HAM/TSP#1 | 551 | chr3_192727658_+ |  |  |  |  | 0 | 1 |
| HAM/TSP#1 | 552 | chr3_194983916_+ |  |  |  |  | 0 | 1 |
| HAM/TSP#1 | 553 | chr3_195030675_+ |  |  |  |  | 0 | 6 |
| HAM/TSP#1 | 554 | chr3_196341020_+ |  |  |  |  | 0 | 2 |
| HAM/TSP#1 | 555 | chr3_197311305_- |  |  |  |  | 0 | 1 |
| HAM/TSP#1 | 556 | chr3_20316800_- |  |  |  |  | 0 | 1 |
| HAM/TSP#1 | 557 | chr3_21696481_+ |  |  |  |  | 0 | 1 |
| HAM/TSP#1 | 558 | chr3_29256994_- |  |  |  |  | 0 | 3 |
| HAM/TSP#1 | 559 | chr3_31346217_+ |  |  |  |  | 0 | 2 |
| HAM/TSP#1 | 560 | chr3_31634801_- |  |  |  |  | 0 | 2 |
| HAM/TSP#1 | 561 | chr3_31648359_- |  |  |  |  | 0 | 56 |
| HAM/TSP#1 | 562 | chr3_3200816_+ |  |  |  |  | 0 | 1 |
| HAM/TSP#1 | 563 | chr3_33484902_- |  |  |  |  | 0 | 1 |
| HAM/TSP#1 | 564 | chr3_34894910_+ |  |  |  |  | 0 | 1 |
| HAM/TSP#1 | 565 | chr3_37173058_+ |  |  |  |  | 0 | 1 |
| HAM/TSP#1 | 566 | chr3_37383815_+ |  |  |  |  | 0 | 1 |
| HAM/TSP#1 | 567 | chr3_39695543_- |  |  |  |  | 0 | 1 |
| HAM/TSP#1 | 568 | chr3_40769844_- |  |  |  |  | 0 | 1 |
| HAM/TSP#1 | 569 | chr3_41660095_+ |  |  |  |  | 0 | 1 |
| HAM/TSP#1 | 570 | chr3_41962036_+ |  |  |  |  | 0 | 1 |
| HAM/TSP#1 | 571 | chr3_43775886_- |  |  |  |  | 0 | 4 |
| HAM/TSP#1 | 572 | chr3_4390241_- |  |  |  |  | 0 | 3 |
| HAM/TSP#1 | 573 | chr3_44557437_- |  |  |  |  | 0 | 13 |
| HAM/TSP#1 | 574 | chr3_4467221_+ |  |  |  |  | 0 | 1 |
| HAM/TSP#1 | 575 | chr3_44982345_+ |  |  |  |  | 0 | 6 |
| HAM/TSP#1 | 576 | chr3_46952921_+ |  |  |  |  | 0 | 1 |
| HAM/TSP#1 | 577 | chr3_47856813_+ |  |  |  |  | 0 | 1 |
| HAM/TSP#1 | 578 | chr3_48185862_- |  |  |  |  | 0 | 1 |
| HAM/TSP#1 | 579 | chr3_48329848_+ |  |  |  |  | 0 | 9 |
| HAM/TSP#1 | 580 | chr3_48604275_- |  |  |  |  | 0 | 3 |
| HAM/TSP#1 | 581 | chr3_4999808_+ |  |  |  |  | 0 | 1 |
| HAM/TSP#1 | 582 | chr3_5020801_- |  |  |  |  | 0 | 1 |
| HAM/TSP#1 | 583 | chr3_50752848_- |  |  |  |  | 0 | 1 |
| HAM/TSP#1 | 584 | chr3_50952477_+ |  |  |  |  | 0 | 1 |
| HAM/TSP#1 | 585 | chr3_52018151_- |  |  |  |  | 0 | 1 |
| HAM/TSP#1 | 586 | chr3_52252901_+ |  |  |  |  | 0 | 17 |
| HAM/TSP#1 | 587 | chr3_54660555_+ |  |  |  |  | 0 | 1 |
| HAM/TSP#1 | 588 | chr3_59761782_+ |  |  |  |  | 0 | 2 |
| HAM/TSP#1 | 589 | chr3_60980911_+ |  |  |  |  | 0 | 1 |
| HAM/TSP#1 | 590 | chr3_61485222_+ |  |  |  |  | 0 | 9 |
| HAM/TSP#1 | 591 | chr3_61554121_+ |  |  |  |  | 0 | 4 |
| HAM/TSP#1 | 592 | chr3_61929471_+ |  |  |  |  | 0 | 1 |
| HAM/TSP#1 | 593 | chr3_62479595_- |  |  |  |  | 0 | 1 |
| HAM/TSP#1 | 594 | chr3_62511876_+ |  |  |  |  | 0 | 2 |
| HAM/TSP#1 | 595 | chr3_62662921_- |  |  |  |  | 0 | 1 |
| HAM/TSP#1 | 596 | chr3_63049663_+ |  |  |  |  | 0 | 5 |
| HAM/TSP#1 | 597 | chr3_63793034_+ |  |  |  |  | 0 | 1 |
| HAM/TSP#1 | 598 | chr3_64209028_+ |  |  |  |  | 0 | 1 |
| HAM/TSP#1 | 599 | chr3_6520078_- |  |  |  |  | 0 | 1 |
| HAM/TSP#1 | 600 | chr3_65422544_- |  |  |  |  | 0 | 1 |
| HAM/TSP#1 | 601 | chr3_66507944_+ |  |  |  |  | 0 | 1 |
| HAM/TSP#1 | 602 | chr3_67882322_+ |  |  |  |  | 0 | 1 |
| HAM/TSP#1 | 603 | chr3_68175372_+ |  |  |  |  | 0 | 1 |
| HAM/TSP#1 | 604 | chr3_68338020_- |  |  |  |  | 0 | 5 |
| HAM/TSP#1 | 605 | chr3_69154656_+ |  |  |  |  | 0 | 1 |
| HAM/TSP#1 | 606 | chr3_69398419_- |  |  |  |  | 0 | 3 |
| HAM/TSP#1 | 607 | chr3_69480532_- |  |  |  |  | 0 | 1 |
| HAM/TSP#1 | 608 | chr3_71663741_- |  |  |  |  | 0 | 1 |
| HAM/TSP#1 | 609 | chr3_71701775_- |  |  |  |  | 0 | 1 |
| HAM/TSP#1 | 610 | chr3_72673235_+ |  |  |  |  | 0 | 1 |
| HAM/TSP#1 | 611 | chr3_73145961_- |  |  |  |  | 0 | 2 |
| HAM/TSP#1 | 612 | chr3_75158603_+ |  |  |  |  | 0 | 6 |
| HAM/TSP#1 | 613 | chr3_75902746_- |  |  |  |  | 0 | 1 |
| HAM/TSP#1 | 614 | chr3_76426529_+ |  |  |  |  | 0 | 1 |
| HAM/TSP#1 | 615 | chr3_77947994_- |  |  |  |  | 0 | 5 |
| HAM/TSP#1 | 616 | chr3_81990404_- |  |  |  |  | 0 | 1 |
| HAM/TSP#1 | 617 | chr3_83145512_- |  |  |  |  | 0 | 4 |
| HAM/TSP#1 | 618 | chr3_83225431_- |  |  |  |  | 0 | 3 |
| HAM/TSP#1 | 619 | chr3_83258617_- |  |  |  |  | 0 | 1 |
| HAM/TSP#1 | 620 | chr3_85065870_- |  |  |  |  | 0 | 1 |
| HAM/TSP#1 | 621 | chr3_86354252_+ |  |  |  |  | 0 | 2 |
| HAM/TSP#1 | 622 | chr3_86424847_+ |  |  |  |  | 0 | 1 |
| HAM/TSP#1 | 623 | chr3_86519461_+ |  |  |  |  | 0 | 4 |
| HAM/TSP#1 | 624 | chr3_88104480_- |  |  |  |  | 0 | 1 |
| HAM/TSP#1 | 625 | chr3_89584936_- |  |  |  |  | 0 | 1 |
| HAM/TSP#1 | 626 | chr3_89691900_+ |  |  |  |  | 0 | 2 |
| HAM/TSP#1 | 627 | chr3_93470361_+ |  |  |  |  | 0 | 2 |
| HAM/TSP#1 | 628 | chr3_93470588_+ |  |  |  |  | 0 | 6 |
| HAM/TSP#1 | 629 | chr3_93470671_- |  |  |  |  | 0 | 3 |
| HAM/TSP#1 | 630 | chr3_94844269_- |  |  |  |  | 0 | 1 |
| HAM/TSP#1 | 631 | chr3_95440625_+ |  |  |  |  | 0 | 5 |
| HAM/TSP#1 | 632 | chr3_9722371_- |  |  |  |  | 0 | 1 |
| HAM/TSP#1 | 633 | chr3_9775401_- |  |  |  |  | 0 | 8 |
| HAM/TSP#1 | 634 | chr3_99810188_- |  |  |  |  | 0 | 1 |
| HAM/TSP#1 | 635 | chr4_100770488_+ |  |  |  |  | 0 | 1 |
| HAM/TSP#1 | 636 | chr4_10117626_- |  |  |  |  | 0 | 2 |
| HAM/TSP#1 | 637 | chr4_104051053_- |  |  |  |  | 0 | 1 |
| HAM/TSP#1 | 638 | chr4_104090157_+ |  |  |  |  | 0 | 12 |
| HAM/TSP#1 | 639 | chr4_104905885_+ |  |  |  |  | 0 | 2 |
| HAM/TSP#1 | 640 | chr4_105810419_- |  |  |  |  | 0 | 1 |
| HAM/TSP#1 | 641 | chr4_106604599_+ |  |  |  |  | 0 | 1 |
| HAM/TSP#1 | 642 | chr4_1076360_- |  |  |  |  | 0 | 1 |
| HAM/TSP#1 | 643 | chr4_107953723_- |  |  |  |  | 0 | 1 |
| HAM/TSP#1 | 644 | chr4_109211282_- |  |  |  |  | 0 | 1 |
| HAM/TSP#1 | 645 | chr4_109635367_+ |  |  |  |  | 0 | 1 |
| HAM/TSP#1 | 646 | chr4_109635368_+ |  |  |  |  | 0 | 17 |
| HAM/TSP#1 | 647 | chr4_112178489_- |  |  |  |  | 0 | 1 |
| HAM/TSP#1 | 648 | chr4_113571793_- |  |  |  |  | 0 | 1 |
| HAM/TSP#1 | 649 | chr4_113642817_- |  |  |  |  | 0 | 1 |
| HAM/TSP#1 | 650 | chr4_113685413_+ |  |  |  |  | 0 | 2 |
| HAM/TSP#1 | 651 | chr4_113827855_- |  |  |  |  | 0 | 1 |
| HAM/TSP#1 | 652 | chr4_115355010_+ |  |  |  |  | 0 | 1 |
| HAM/TSP#1 | 653 | chr4_116208980_+ |  |  |  |  | 0 | 3 |
| HAM/TSP#1 | 654 | chr4_116994371_+ |  |  |  |  | 0 | 3 |
| HAM/TSP#1 | 655 | chr4_117752012_+ |  |  |  |  | 0 | 26 |
| HAM/TSP#1 | 656 | chr4_118264446_- |  |  |  |  | 0 | 18 |
| HAM/TSP#1 | 657 | chr4_118601163_- |  |  |  |  | 0 | 1 |
| HAM/TSP#1 | 658 | chr4_121261531_- |  |  |  |  | 0 | 1 |
| HAM/TSP#1 | 659 | chr4_121513392_+ |  |  |  |  | 0 | 1 |
| HAM/TSP#1 | 660 | chr4_125365440_+ |  |  |  |  | 0 | 8 |
| HAM/TSP#1 | 661 | chr4_127000359_- |  |  |  |  | 0 | 6 |
| HAM/TSP#1 | 662 | chr4_127246943_- |  |  |  |  | 0 | 1 |
| HAM/TSP#1 | 663 | chr4_127389508_+ |  |  |  |  | 0 | 1 |
| HAM/TSP#1 | 664 | chr4_128817189_- |  |  |  |  | 0 | 4 |
| HAM/TSP#1 | 665 | chr4_129434643_+ |  |  |  |  | 0 | 1 |
| HAM/TSP#1 | 666 | chr4_129571496_- |  |  |  |  | 0 | 1 |
| HAM/TSP#1 | 667 | chr4_13074550_- |  |  |  |  | 0 | 7 |
| HAM/TSP#1 | 668 | chr4_131732143_- |  |  |  |  | 0 | 1 |
| HAM/TSP#1 | 669 | chr4_132678762_- |  |  |  |  | 0 | 1 |
| HAM/TSP#1 | 670 | chr4_132812289_- |  |  |  |  | 0 | 1 |
| HAM/TSP#1 | 671 | chr4_133559979_+ |  |  |  |  | 0 | 1 |
| HAM/TSP#1 | 672 | chr4_133951376_+ |  |  |  |  | 0 | 6 |
| HAM/TSP#1 | 673 | chr4_13534827_+ |  |  |  |  | 0 | 1 |
| HAM/TSP#1 | 674 | chr4_135415281_- |  |  |  |  | 0 | 1 |
| HAM/TSP#1 | 675 | chr4_135898209_- |  |  |  |  | 0 | 1 |
| HAM/TSP#1 | 676 | chr4_137224542_+ |  |  |  |  | 0 | 1 |
| HAM/TSP#1 | 677 | chr4_139051662_+ |  |  |  |  | 0 | 1 |
| HAM/TSP#1 | 678 | chr4_139780091_- |  |  |  |  | 0 | 25 |
| HAM/TSP#1 | 679 | chr4_140588842_- |  |  |  |  | 0 | 1 |
| HAM/TSP#1 | 680 | chr4_141945442_- |  |  |  |  | 0 | 5 |
| HAM/TSP#1 | 681 | chr4_141981070_+ |  |  |  |  | 0 | 1 |
| HAM/TSP#1 | 682 | chr4_142595231_+ |  |  |  |  | 0 | 1 |
| HAM/TSP#1 | 683 | chr4_143535046_+ |  |  |  |  | 0 | 2 |
| HAM/TSP#1 | 684 | chr4_143949577_+ |  |  |  |  | 0 | 1 |
| HAM/TSP#1 | 685 | chr4_144101026_+ |  |  |  |  | 0 | 4 |
| HAM/TSP#1 | 686 | chr4_144622224_- |  |  |  |  | 0 | 1 |
| HAM/TSP#1 | 687 | chr4_14576132_- |  |  |  |  | 0 | 1 |
| HAM/TSP#1 | 688 | chr4_145861846_+ |  |  |  |  | 0 | 5 |
| HAM/TSP#1 | 689 | chr4_147679772_- |  |  |  |  | 0 | 1 |
| HAM/TSP#1 | 690 | chr4_147769349_+ |  |  |  |  | 0 | 3 |
| HAM/TSP#1 | 691 | chr4_147851681_+ |  |  |  |  | 0 | 35 |
| HAM/TSP#1 | 692 | chr4_149458039_+ |  |  |  |  | 0 | 1 |
| HAM/TSP#1 | 693 | chr4_15006746_+ |  |  |  |  | 0 | 3 |
| HAM/TSP#1 | 694 | chr4_15094684_+ |  |  |  |  | 0 | 1 |
| HAM/TSP#1 | 695 | chr4_151673449_+ |  |  |  |  | 0 | 4 |
| HAM/TSP#1 | 696 | chr4_152096166_+ |  |  |  |  | 0 | 1 |
| HAM/TSP#1 | 697 | chr4_152675694_+ |  |  |  |  | 0 | 2 |
| HAM/TSP#1 | 698 | chr4_152718973_+ |  |  |  |  | 0 | 4 |
| HAM/TSP#1 | 699 | chr4_15334078_+ |  |  |  |  | 0 | 1 |
| HAM/TSP#1 | 700 | chr4_153487287_+ |  |  |  |  | 0 | 4 |
| HAM/TSP#1 | 701 | chr4_154036488_- |  |  |  |  | 0 | 9 |
| HAM/TSP#1 | 702 | chr4_154062351_- |  |  |  |  | 0 | 1 |
| HAM/TSP#1 | 703 | chr4_155265086_+ |  |  |  |  | 0 | 4 |
| HAM/TSP#1 | 704 | chr4_155665165_+ |  |  |  |  | 0 | 3 |
| HAM/TSP#1 | 705 | chr4_155699530_+ |  |  |  |  | 0 | 1 |
| HAM/TSP#1 | 706 | chr4_160043778_+ |  |  |  |  | 0 | 1 |
| HAM/TSP#1 | 707 | chr4_161338070_+ |  |  |  |  | 0 | 3 |
| HAM/TSP#1 | 708 | chr4_161701491_+ |  |  |  |  | 0 | 1 |
| HAM/TSP#1 | 709 | chr4_161794609_+ |  |  |  |  | 0 | 1 |
| HAM/TSP#1 | 710 | chr4_16420957_- |  |  |  |  | 0 | 2 |
| HAM/TSP#1 | 711 | chr4_164487013_- |  |  |  |  | 0 | 1 |
| HAM/TSP#1 | 712 | chr4_164855318_+ |  |  |  |  | 0 | 2 |
| HAM/TSP#1 | 713 | chr4_165772010_+ |  |  |  |  | 0 | 1 |
| HAM/TSP#1 | 714 | chr4_168321551_- |  |  |  |  | 0 | 3 |
| HAM/TSP#1 | 715 | chr4_169654115_- |  |  |  |  | 0 | 2 |
| HAM/TSP#1 | 716 | chr4_170154753_+ |  |  |  |  | 0 | 1 |
| HAM/TSP#1 | 717 | chr4_171208457_- |  |  |  |  | 0 | 1 |
| HAM/TSP#1 | 718 | chr4_173777581_+ |  |  |  |  | 0 | 1 |
| HAM/TSP#1 | 719 | chr4_177747203_+ |  |  |  |  | 0 | 4 |
| HAM/TSP#1 | 720 | chr4_179657970_+ |  |  |  |  | 0 | 1 |
| HAM/TSP#1 | 721 | chr4_179975299_- |  |  |  |  | 0 | 1 |
| HAM/TSP#1 | 722 | chr4_184263634_+ |  |  |  |  | 0 | 1 |
| HAM/TSP#1 | 723 | chr4_184525327_- |  |  |  |  | 0 | 1 |
| HAM/TSP#1 | 724 | chr4_186454232_- |  |  |  |  | 0 | 1 |
| HAM/TSP#1 | 725 | chr4_186701690_- |  |  |  |  | 0 | 1 |
| HAM/TSP#1 | 726 | chr4_186762339_- |  |  |  |  | 0 | 1 |
| HAM/TSP#1 | 727 | chr4_188261492_- |  |  |  |  | 0 | 3 |
| HAM/TSP#1 | 728 | chr4_189301950_+ |  |  |  |  | 0 | 1 |
| HAM/TSP#1 | 729 | chr4_189412754_- |  |  |  |  | 0 | 3 |
| HAM/TSP#1 | 730 | chr4_19069548_+ |  |  |  |  | 0 | 2 |
| HAM/TSP#1 | 731 | chr4_19303633_+ |  |  |  |  | 0 | 15 |
| HAM/TSP#1 | 732 | chr4_19756967_- |  |  |  |  | 0 | 1 |
| HAM/TSP#1 | 733 | chr4_20351620_+ |  |  |  |  | 0 | 1 |
| HAM/TSP#1 | 734 | chr4_20477780_+ |  |  |  |  | 0 | 1 |
| HAM/TSP#1 | 735 | chr4_21519826_- |  |  |  |  | 0 | 1 |
| HAM/TSP#1 | 736 | chr4_22730865_+ |  |  |  |  | 0 | 1 |
| HAM/TSP#1 | 737 | chr4_23091449_- |  |  |  |  | 0 | 2 |
| HAM/TSP#1 | 738 | chr4_23737013_+ |  |  |  |  | 0 | 2 |
| HAM/TSP#1 | 739 | chr4_23910713_- |  |  |  |  | 0 | 1 |
| HAM/TSP#1 | 740 | chr4_24880756_+ |  |  |  |  | 0 | 1 |
| HAM/TSP#1 | 741 | chr4_26684566_- |  |  |  |  | 0 | 4 |
| HAM/TSP#1 | 742 | chr4_26866985_- |  |  |  |  | 0 | 5 |
| HAM/TSP#1 | 743 | chr4_28365663_+ |  |  |  |  | 0 | 2 |
| HAM/TSP#1 | 744 | chr4_30751057_- |  |  |  |  | 0 | 3 |
| HAM/TSP#1 | 745 | chr4_31194326_+ |  |  |  |  | 0 | 2 |
| HAM/TSP#1 | 746 | chr4_32473400_+ |  |  |  |  | 0 | 1 |
| HAM/TSP#1 | 747 | chr4_35058825_+ |  |  |  |  | 0 | 10 |
| HAM/TSP#1 | 748 | chr4_3618234_+ |  |  |  |  | 0 | 23 |
| HAM/TSP#1 | 749 | chr4_3644934_+ |  |  |  |  | 0 | 1 |
| HAM/TSP#1 | 750 | chr4_36530448_+ |  |  |  |  | 0 | 1 |
| HAM/TSP#1 | 751 | chr4_36535387_+ |  |  |  |  | 0 | 3 |
| HAM/TSP#1 | 752 | chr4_36554584_- |  |  |  |  | 0 | 1 |
| HAM/TSP#1 | 753 | chr4_38696986_- |  |  |  |  | 0 | 3 |
| HAM/TSP#1 | 754 | chr4_39736608_- |  |  |  |  | 0 | 2 |
| HAM/TSP#1 | 755 | chr4_41936264_- |  |  |  |  | 0 | 1 |
| HAM/TSP#1 | 756 | chr4_41970924_+ |  |  |  |  | 0 | 4 |
| HAM/TSP#1 | 757 | chr4_43298475_- |  |  |  |  | 0 | 1 |
| HAM/TSP#1 | 758 | chr4_43459954_+ |  |  |  |  | 0 | 1 |
| HAM/TSP#1 | 759 | chr4_44316776_- |  |  |  |  | 0 | 7 |
| HAM/TSP#1 | 760 | chr4_44640859_- |  |  |  |  | 0 | 6 |
| HAM/TSP#1 | 761 | chr4_44897011_- |  |  |  |  | 0 | 4 |
| HAM/TSP#1 | 762 | chr4_45143314_+ |  |  |  |  | 0 | 1 |
| HAM/TSP#1 | 763 | chr4_45376653_- |  |  |  |  | 0 | 1 |
| HAM/TSP#1 | 764 | chr4_4605613_+ |  |  |  |  | 0 | 1 |
| HAM/TSP#1 | 765 | chr4_46431822_- |  |  |  |  | 0 | 2 |
| HAM/TSP#1 | 766 | chr4_46876883_+ |  |  |  |  | 0 | 2 |
| HAM/TSP#1 | 767 | chr4_47843481_+ |  |  |  |  | 0 | 3 |
| HAM/TSP#1 | 768 | chr4_48033513_+ |  |  |  |  | 0 | 1 |
| HAM/TSP#1 | 769 | chr4_48048379_+ |  |  |  |  | 0 | 5 |
| HAM/TSP#1 | 770 | chr4_48048411_+ |  |  |  |  | 0 | 1 |
| HAM/TSP#1 | 771 | chr4_48452480_- |  |  |  |  | 0 | 8 |
| HAM/TSP#1 | 772 | chr4_48613670_- |  |  |  |  | 0 | 1 |
| HAM/TSP#1 | 773 | chr4_48766318_+ |  |  |  |  | 0 | 4 |
| HAM/TSP#1 | 774 | chr4_48835297_+ |  |  |  |  | 0 | 9 |
| HAM/TSP#1 | 775 | chr4_49076403_+ |  |  |  |  | 0 | 6 |
| HAM/TSP#1 | 776 | chr4_49154610_- |  |  |  |  | 0 | 3 |
| HAM/TSP#1 | 777 | chr4_49178091_- |  |  |  |  | 0 | 1 |
| HAM/TSP#1 | 778 | chr4_49514553_- |  |  |  |  | 0 | 1 |
| HAM/TSP#1 | 779 | chr4_52994070_- |  |  |  |  | 0 | 1 |
| HAM/TSP#1 | 780 | chr4_53092716_- |  |  |  |  | 0 | 5 |
| HAM/TSP#1 | 781 | chr4_53973460_+ |  |  |  |  | 0 | 1 |
| HAM/TSP#1 | 782 | chr4_56894474_- |  |  |  |  | 0 | 1 |
| HAM/TSP#1 | 783 | chr4_57078544_+ |  |  |  |  | 0 | 1 |
| HAM/TSP#1 | 784 | chr4_57435168_- |  |  |  |  | 0 | 1 |
| HAM/TSP#1 | 785 | chr4_57477268_+ |  |  |  |  | 0 | 7 |
| HAM/TSP#1 | 786 | chr4_57912667_+ |  |  |  |  | 0 | 2 |
| HAM/TSP#1 | 787 | chr4_59241263_- |  |  |  |  | 0 | 1 |
| HAM/TSP#1 | 788 | chr4_59551984_- |  |  |  |  | 0 | 2 |
| HAM/TSP#1 | 789 | chr4_6045053_+ |  |  |  |  | 0 | 1 |
| HAM/TSP#1 | 790 | chr4_61004757_- |  |  |  |  | 0 | 1 |
| HAM/TSP#1 | 791 | chr4_61320088_+ |  |  |  |  | 0 | 1 |
| HAM/TSP#1 | 792 | chr4_61529809_+ |  |  |  |  | 0 | 1 |
| HAM/TSP#1 | 793 | chr4_63251990_+ |  |  |  |  | 0 | 1 |
| HAM/TSP#1 | 794 | chr4_64166844_- |  |  |  |  | 0 | 1 |
| HAM/TSP#1 | 795 | chr4_64361490_+ |  |  |  |  | 0 | 1 |
| HAM/TSP#1 | 796 | chr4_65667693_- |  |  |  |  | 0 | 1 |
| HAM/TSP#1 | 797 | chr4_66263752_+ |  |  |  |  | 0 | 3 |
| HAM/TSP#1 | 798 | chr4_68369774_+ |  |  |  |  | 0 | 1 |
| HAM/TSP#1 | 799 | chr4_68855492_- |  |  |  |  | 0 | 1 |
| HAM/TSP#1 | 800 | chr4_68989548_+ |  |  |  |  | 0 | 1 |
| HAM/TSP#1 | 801 | chr4_6939032_- |  |  |  |  | 0 | 1 |
| HAM/TSP#1 | 802 | chr4_69501776_- |  |  |  |  | 0 | 1 |
| HAM/TSP#1 | 803 | chr4_7044260_- |  |  |  |  | 0 | 1 |
| HAM/TSP#1 | 804 | chr4_71269674_- |  |  |  |  | 0 | 2 |
| HAM/TSP#1 | 805 | chr4_7145914_- |  |  |  |  | 0 | 1 |
| HAM/TSP#1 | 806 | chr4_72929765_- |  |  |  |  | 0 | 2 |
| HAM/TSP#1 | 807 | chr4_73215333_+ |  |  |  |  | 0 | 26 |
| HAM/TSP#1 | 808 | chr4_7457915_+ |  |  |  |  | 0 | 1 |
| HAM/TSP#1 | 809 | chr4_75411438_+ |  |  |  |  | 0 | 2 |
| HAM/TSP#1 | 810 | chr4_75556393_+ |  |  |  |  | 0 | 1 |
| HAM/TSP#1 | 811 | chr4_7565691_- |  |  |  |  | 0 | 5 |
| HAM/TSP#1 | 812 | chr4_75986924_+ |  |  |  |  | 0 | 1 |
| HAM/TSP#1 | 813 | chr4_77662690_+ |  |  |  |  | 0 | 2 |
| HAM/TSP#1 | 814 | chr4_79143237_- |  |  |  |  | 0 | 1 |
| HAM/TSP#1 | 815 | chr4_79259322_- |  |  |  |  | 0 | 1 |
| HAM/TSP#1 | 816 | chr4_80593092_+ |  |  |  |  | 0 | 1 |
| HAM/TSP#1 | 817 | chr4_80750042_+ |  |  |  |  | 0 | 1 |
| HAM/TSP#1 | 818 | chr4_8218760_- |  |  |  |  | 0 | 1 |
| HAM/TSP#1 | 819 | chr4_8247849_- |  |  |  |  | 0 | 1 |
| HAM/TSP#1 | 820 | chr4_8390413_+ |  |  |  |  | 0 | 1 |
| HAM/TSP#1 | 821 | chr4_84550783_- |  |  |  |  | 0 | 1 |
| HAM/TSP#1 | 822 | chr4_8543847_+ |  |  |  |  | 0 | 1 |
| HAM/TSP#1 | 823 | chr4_85527173_- |  |  |  |  | 0 | 1 |
| HAM/TSP#1 | 824 | chr4_86083778_- |  |  |  |  | 0 | 1 |
| HAM/TSP#1 | 825 | chr4_86532334_+ |  |  |  |  | 0 | 4 |
| HAM/TSP#1 | 826 | chr4_8732787_+ |  |  |  |  | 0 | 1 |
| HAM/TSP#1 | 827 | chr4_87439644_+ |  |  |  |  | 0 | 1 |
| HAM/TSP#1 | 828 | chr4_87978618_+ |  |  |  |  | 0 | 1 |
| HAM/TSP#1 | 829 | chr4_88458601_- |  |  |  |  | 0 | 1 |
| HAM/TSP#1 | 830 | chr4_88565546_+ |  |  |  |  | 0 | 1 |
| HAM/TSP#1 | 831 | chr4_88934629_- |  |  |  |  | 0 | 2 |
| HAM/TSP#1 | 832 | chr4_91871589_- |  |  |  |  | 0 | 1 |
| HAM/TSP#1 | 833 | chr4_93090727_+ |  |  |  |  | 0 | 1 |
| HAM/TSP#1 | 834 | chr4_94308194_+ |  |  |  |  | 0 | 1 |
| HAM/TSP#1 | 835 | chr4_9698232_- |  |  |  |  | 0 | 1 |
| HAM/TSP#1 | 836 | chr4_9713115_- |  |  |  |  | 0 | 1 |
| HAM/TSP#1 | 837 | chr4_97280030_- |  |  |  |  | 0 | 18 |
| HAM/TSP#1 | 838 | chr4_977930_- |  |  |  |  | 0 | 7 |
| HAM/TSP#1 | 839 | chr4_99523410_+ |  |  |  |  | 0 | 1 |
| HAM/TSP#1 | 840 | chr5_100389773_+ |  |  |  |  | 0 | 1 |
| HAM/TSP#1 | 841 | chr5_100849314_+ |  |  |  |  | 0 | 3 |
| HAM/TSP#1 | 842 | chr5_100912655_- |  |  |  |  | 0 | 1 |
| HAM/TSP#1 | 843 | chr5_101223942_- |  |  |  |  | 0 | 1 |
| HAM/TSP#1 | 844 | chr5_10192674_+ |  |  |  |  | 0 | 1 |
| HAM/TSP#1 | 845 | chr5_102089804_- |  |  |  |  | 0 | 1 |
| HAM/TSP#1 | 846 | chr5_102451368_- |  |  |  |  | 0 | 1 |
| HAM/TSP#1 | 847 | chr5_102997384_- |  |  |  |  | 0 | 5 |
| HAM/TSP#1 | 848 | chr5_103637409_- |  |  |  |  | 0 | 17 |
| HAM/TSP#1 | 849 | chr5_105301431_- |  |  |  |  | 0 | 1 |
| HAM/TSP#1 | 850 | chr5_10768449_+ |  |  |  |  | 0 | 1 |
| HAM/TSP#1 | 851 | chr5_110075628_- |  |  |  |  | 0 | 5 |
| HAM/TSP#1 | 852 | chr5_11027382_+ |  |  |  |  | 0 | 4 |
| HAM/TSP#1 | 853 | chr5_112233427_- |  |  |  |  | 0 | 1 |
| HAM/TSP#1 | 854 | chr5_112910129_- |  |  |  |  | 0 | 2 |
| HAM/TSP#1 | 855 | chr5_113658431_+ |  |  |  |  | 0 | 1 |
| HAM/TSP#1 | 856 | chr5_114077388_+ |  |  |  |  | 0 | 1 |
| HAM/TSP#1 | 857 | chr5_114364018_- |  |  |  |  | 0 | 1 |
| HAM/TSP#1 | 858 | chr5_114413514_+ |  |  |  |  | 0 | 7 |
| HAM/TSP#1 | 859 | chr5_115734996_+ |  |  |  |  | 0 | 1 |
| HAM/TSP#1 | 860 | chr5_115855418_+ |  |  |  |  | 0 | 1 |
| HAM/TSP#1 | 861 | chr5_116381546_+ |  |  |  |  | 0 | 2 |
| HAM/TSP#1 | 862 | chr5_117958507_- |  |  |  |  | 0 | 2 |
| HAM/TSP#1 | 863 | chr5_120215387_+ |  |  |  |  | 0 | 1 |
| HAM/TSP#1 | 864 | chr5_124249583_+ |  |  |  |  | 0 | 1 |
| HAM/TSP#1 | 865 | chr5_124430908_- |  |  |  |  | 0 | 1 |
| HAM/TSP#1 | 866 | chr5_126906_- |  |  |  |  | 0 | 1 |
| HAM/TSP#1 | 867 | chr5_127874086_+ |  |  |  |  | 0 | 1 |
| HAM/TSP#1 | 868 | chr5_128973119_+ |  |  |  |  | 0 | 1 |
| HAM/TSP#1 | 869 | chr5_129120615_+ |  |  |  |  | 0 | 1 |
| HAM/TSP#1 | 870 | chr5_131337478_+ |  |  |  |  | 0 | 1 |
| HAM/TSP#1 | 871 | chr5_131609359_+ |  |  |  |  | 0 | 1 |
| HAM/TSP#1 | 872 | chr5_131956050_+ |  |  |  |  | 0 | 1 |
| HAM/TSP#1 | 873 | chr5_132430794_- |  |  |  |  | 0 | 1 |
| HAM/TSP#1 | 874 | chr5_13348504_- |  |  |  |  | 1 | 0 |
| HAM/TSP#1 | 875 | chr5_134594879_+ |  |  |  |  | 23 | 194 |
| HAM/TSP#1 | 876 | chr5_135582248_+ |  |  |  |  | 0 | 1 |
| HAM/TSP#1 | 877 | chr5_135725348_+ |  |  |  |  | 0 | 1 |
| HAM/TSP#1 | 878 | chr5_139343429_+ |  |  |  |  | 0 | 2 |
| HAM/TSP#1 | 879 | chr5_1394198_- |  |  |  |  | 0 | 1 |
| HAM/TSP#1 | 880 | chr5_139681172_+ |  |  |  |  | 0 | 1 |
| HAM/TSP#1 | 881 | chr5_139762248_- |  |  |  |  | 0 | 1 |
| HAM/TSP#1 | 882 | chr5_140875827_- |  |  |  |  | 0 | 1 |
| HAM/TSP#1 | 883 | chr5_141296780_- |  |  |  |  | 0 | 1 |
| HAM/TSP#1 | 884 | chr5_141577502_+ |  |  |  |  | 0 | 2 |
| HAM/TSP#1 | 885 | chr5_141620471_- |  |  |  |  | 0 | 2 |
| HAM/TSP#1 | 886 | chr5_142244837_+ |  |  |  |  | 0 | 1 |
| HAM/TSP#1 | 887 | chr5_142323678_- |  |  |  |  | 0 | 1 |
| HAM/TSP#1 | 888 | chr5_142750164_+ |  |  |  |  | 0 | 26 |
| HAM/TSP#1 | 889 | chr5_142757632_+ |  |  |  |  | 0 | 1 |
| HAM/TSP#1 | 890 | chr5_143630138_+ |  |  |  |  | 0 | 5 |
| HAM/TSP#1 | 891 | chr5_146523437_- |  |  |  |  | 0 | 1 |
| HAM/TSP#1 | 892 | chr5_149212004_+ |  |  |  |  | 0 | 1 |
| HAM/TSP#1 | 893 | chr5_149241873_+ |  |  |  |  | 0 | 1 |
| HAM/TSP#1 | 894 | chr5_149317941_- |  |  |  |  | 0 | 1 |
| HAM/TSP#1 | 895 | chr5_150293438_+ |  |  |  |  | 0 | 1 |
| HAM/TSP#1 | 896 | chr5_153955623_- |  |  |  |  | 0 | 1 |
| HAM/TSP#1 | 897 | chr5_154235759_- |  |  |  |  | 0 | 1 |
| HAM/TSP#1 | 898 | chr5_157163356_+ |  |  |  |  | 0 | 1 |
| HAM/TSP#1 | 899 | chr5_157291281_+ |  |  |  |  | 0 | 1 |
| HAM/TSP#1 | 900 | chr5_157351546_+ |  |  |  |  | 0 | 1 |
| HAM/TSP#1 | 901 | chr5_157931765_+ |  |  |  |  | 0 | 38 |
| HAM/TSP#1 | 902 | chr5_159930886_+ |  |  |  |  | 0 | 1 |
| HAM/TSP#1 | 903 | chr5_161350777_- |  |  |  |  | 0 | 1 |
| HAM/TSP#1 | 904 | chr5_163900854_- |  |  |  |  | 0 | 4 |
| HAM/TSP#1 | 905 | chr5_164660506_+ |  |  |  |  | 0 | 1 |
| HAM/TSP#1 | 906 | chr5_16470062_+ |  |  |  |  | 0 | 2 |
| HAM/TSP#1 | 907 | chr5_167944382_- |  |  |  |  | 0 | 8 |
| HAM/TSP#1 | 908 | chr5_168471614_+ |  |  |  |  | 0 | 1 |
| HAM/TSP#1 | 909 | chr5_170607810_+ |  |  |  |  | 0 | 1 |
| HAM/TSP#1 | 910 | chr5_172176479_+ |  |  |  |  | 0 | 1 |
| HAM/TSP#1 | 911 | chr5_172350495_- |  |  |  |  | 0 | 1 |
| HAM/TSP#1 | 912 | chr5_173561732_+ |  |  |  |  | 0 | 1 |
| HAM/TSP#1 | 913 | chr5_1745591_- |  |  |  |  | 0 | 1 |
| HAM/TSP#1 | 914 | chr5_174697325_- |  |  |  |  | 0 | 1 |
| HAM/TSP#1 | 915 | chr5_174815600_+ |  |  |  |  | 0 | 1 |
| HAM/TSP#1 | 916 | chr5_175169002_+ |  |  |  |  | 0 | 1 |
| HAM/TSP#1 | 917 | chr5_176496754_- |  |  |  |  | 0 | 1 |
| HAM/TSP#1 | 918 | chr5_176889796_+ |  |  |  |  | 0 | 2 |
| HAM/TSP#1 | 919 | chr5_177560561_+ |  |  |  |  | 0 | 1 |
| HAM/TSP#1 | 920 | chr5_178811911_+ |  |  |  |  | 0 | 1 |
| HAM/TSP#1 | 921 | chr5_179723850_+ |  |  |  |  | 0 | 5 |
| HAM/TSP#1 | 922 | chr5_179792136_+ |  |  |  |  | 0 | 1 |
| HAM/TSP#1 | 923 | chr5_180215802_+ |  |  |  |  | 0 | 1 |
| HAM/TSP#1 | 924 | chr5_180329129_+ |  |  |  |  | 0 | 1 |
| HAM/TSP#1 | 925 | chr5_18386992_- |  |  |  |  | 0 | 2 |
| HAM/TSP#1 | 926 | chr5_18589215_+ |  |  |  |  | 0 | 1 |
| HAM/TSP#1 | 927 | chr5_20333037_+ |  |  |  |  | 0 | 2 |
| HAM/TSP#1 | 928 | chr5_2178869_+ |  |  |  |  | 0 | 1 |
| HAM/TSP#1 | 929 | chr5_21887612_- |  |  |  |  | 0 | 1 |
| HAM/TSP#1 | 930 | chr5_21917196_+ |  |  |  |  | 0 | 1 |
| HAM/TSP#1 | 931 | chr5_24065025_- |  |  |  |  | 0 | 1 |
| HAM/TSP#1 | 932 | chr5_2538717_+ |  |  |  |  | 0 | 1 |
| HAM/TSP#1 | 933 | chr5_2645176_- |  |  |  |  | 0 | 1 |
| HAM/TSP#1 | 934 | chr5_26976683_- |  |  |  |  | 0 | 1 |
| HAM/TSP#1 | 935 | chr5_28185342_+ |  |  |  |  | 0 | 1 |
| HAM/TSP#1 | 936 | chr5_28953625_+ |  |  |  |  | 0 | 2 |
| HAM/TSP#1 | 937 | chr5_29121666_- |  |  |  |  | 0 | 1 |
| HAM/TSP#1 | 938 | chr5_29298281_- |  |  |  |  | 0 | 1 |
| HAM/TSP#1 | 939 | chr5_29771396_+ |  |  |  |  | 0 | 3 |
| HAM/TSP#1 | 940 | chr5_30184807_- |  |  |  |  | 0 | 1 |
| HAM/TSP#1 | 941 | chr5_32322732_- |  |  |  |  | 0 | 1 |
| HAM/TSP#1 | 942 | chr5_32448649_- |  |  |  |  | 0 | 2 |
| HAM/TSP#1 | 943 | chr5_33120728_+ |  |  |  |  | 0 | 1 |
| HAM/TSP#1 | 944 | chr5_33419431_+ |  |  |  |  | 0 | 4 |
| HAM/TSP#1 | 945 | chr5_33432215_- |  |  |  |  | 0 | 3 |
| HAM/TSP#1 | 946 | chr5_33740427_+ |  |  |  |  | 0 | 2 |
| HAM/TSP#1 | 947 | chr5_33911345_- |  |  |  |  | 0 | 1 |
| HAM/TSP#1 | 948 | chr5_34439339_+ |  |  |  |  | 0 | 1 |
| HAM/TSP#1 | 949 | chr5_34466745_+ |  |  |  |  | 0 | 1 |
| HAM/TSP#1 | 950 | chr5_34960144_- |  |  |  |  | 0 | 1 |
| HAM/TSP#1 | 951 | chr5_36541502_+ |  |  |  |  | 1 | 0 |
| HAM/TSP#1 | 952 | chr5_37376701_- |  |  |  |  | 0 | 2 |
| HAM/TSP#1 | 953 | chr5_374537_+ |  |  |  |  | 0 | 2 |
| HAM/TSP#1 | 954 | chr5_37651842_- |  |  |  |  | 0 | 1 |
| HAM/TSP#1 | 955 | chr5_38644377_- |  |  |  |  | 0 | 1 |
| HAM/TSP#1 | 956 | chr5_39058413_- |  |  |  |  | 0 | 1 |
| HAM/TSP#1 | 957 | chr5_39257553_+ |  |  |  |  | 0 | 1 |
| HAM/TSP#1 | 958 | chr5_40027284_- |  |  |  |  | 0 | 2 |
| HAM/TSP#1 | 959 | chr5_40359389_- |  |  |  |  | 0 | 1 |
| HAM/TSP#1 | 960 | chr5_41340067_+ |  |  |  |  | 0 | 1 |
| HAM/TSP#1 | 961 | chr5_43402469_+ |  |  |  |  | 0 | 1 |
| HAM/TSP#1 | 962 | chr5_44603787_- |  |  |  |  | 0 | 1 |
| HAM/TSP#1 | 963 | chr5_45770240_+ |  |  |  |  | 0 | 1 |
| HAM/TSP#1 | 964 | chr5_50161689_- |  |  |  |  | 0 | 41 |
| HAM/TSP#1 | 965 | chr5_50208623_+ |  |  |  |  | 0 | 2 |
| HAM/TSP#1 | 966 | chr5_50417441_- |  |  |  |  | 0 | 2 |
| HAM/TSP#1 | 967 | chr5_51052093_- |  |  |  |  | 0 | 1 |
| HAM/TSP#1 | 968 | chr5_52961666_- |  |  |  |  | 0 | 1 |
| HAM/TSP#1 | 969 | chr5_54742933_+ |  |  |  |  | 0 | 3 |
| HAM/TSP#1 | 970 | chr5_57993069_- |  |  |  |  | 0 | 15 |
| HAM/TSP#1 | 971 | chr5_602709_- |  |  |  |  | 0 | 1 |
| HAM/TSP#1 | 972 | chr5_60935332_- |  |  |  |  | 0 | 1 |
| HAM/TSP#1 | 973 | chr5_61553416_+ |  |  |  |  | 0 | 3 |
| HAM/TSP#1 | 974 | chr5_62458504_+ |  |  |  |  | 0 | 1 |
| HAM/TSP#1 | 975 | chr5_63553292_- |  |  |  |  | 0 | 2 |
| HAM/TSP#1 | 976 | chr5_63940433_- |  |  |  |  | 0 | 1 |
| HAM/TSP#1 | 977 | chr5_64244359_+ |  |  |  |  | 0 | 1 |
| HAM/TSP#1 | 978 | chr5_64690352_+ |  |  |  |  | 0 | 1 |
| HAM/TSP#1 | 979 | chr5_65161290_- |  |  |  |  | 0 | 1 |
| HAM/TSP#1 | 980 | chr5_65189460_+ |  |  |  |  | 0 | 1 |
| HAM/TSP#1 | 981 | chr5_65246134_+ |  |  |  |  | 0 | 1 |
| HAM/TSP#1 | 982 | chr5_65686337_+ |  |  |  |  | 0 | 1 |
| HAM/TSP#1 | 983 | chr5_66155646_- |  |  |  |  | 0 | 8 |
| HAM/TSP#1 | 984 | chr5_6831732_- |  |  |  |  | 0 | 7 |
| HAM/TSP#1 | 985 | chr5_69140717_- |  |  |  |  | 0 | 1 |
| HAM/TSP#1 | 986 | chr5_73450035_+ |  |  |  |  | 0 | 4 |
| HAM/TSP#1 | 987 | chr5_74462556_+ |  |  |  |  | 0 | 5 |
| HAM/TSP#1 | 988 | chr5_7527709_- |  |  |  |  | 0 | 1 |
| HAM/TSP#1 | 989 | chr5_77496946_+ |  |  |  |  | 0 | 3 |
| HAM/TSP#1 | 990 | chr5_78310025_- |  |  |  |  | 0 | 1 |
| HAM/TSP#1 | 991 | chr5_79112271_- |  |  |  |  | 0 | 1 |
| HAM/TSP#1 | 992 | chr5_7931657_+ |  |  |  |  | 0 | 1 |
| HAM/TSP#1 | 993 | chr5_80050556_- |  |  |  |  | 0 | 3 |
| HAM/TSP#1 | 994 | chr5_80177062_- |  |  |  |  | 0 | 1 |
| HAM/TSP#1 | 995 | chr5_80817286_- |  |  |  |  | 0 | 1 |
| HAM/TSP#1 | 996 | chr5_81526061_- |  |  |  |  | 3 | 2 |
| HAM/TSP#1 | 997 | chr5_82268840_+ |  |  |  |  | 0 | 8 |
| HAM/TSP#1 | 998 | chr5_85056496_- |  |  |  |  | 0 | 1 |
| HAM/TSP#1 | 999 | chr5_85312565_+ |  |  |  |  | 0 | 1 |
| HAM/TSP#1 | 1000 | chr5_87122346_- |  |  |  |  | 0 | 6 |
| HAM/TSP#1 | 1001 | chr5_88800571_+ |  |  |  |  | 0 | 1 |
| HAM/TSP#1 | 1002 | chr5_90012079_+ |  |  |  |  | 1 | 30 |
| HAM/TSP#1 | 1003 | chr5_91289287_- |  |  |  |  | 0 | 1 |
| HAM/TSP#1 | 1004 | chr5_91498912_+ |  |  |  |  | 0 | 1 |
| HAM/TSP#1 | 1005 | chr5_92226921_+ |  |  |  |  | 0 | 1 |
| HAM/TSP#1 | 1006 | chr5_92513027_- |  |  |  |  | 0 | 1 |
| HAM/TSP#1 | 1007 | chr5_93953585_- |  |  |  |  | 0 | 1 |
| HAM/TSP#1 | 1008 | chr5_9398458_+ |  |  |  |  | 0 | 1 |
| HAM/TSP#1 | 1009 | chr5_94456941_+ |  |  |  |  | 2 | 0 |
| HAM/TSP#1 | 1010 | chr5_95683541_+ |  |  |  |  | 1 | 1 |
| HAM/TSP#1 | 1011 | chr5_96410769_+ |  |  |  |  | 0 | 1 |
| HAM/TSP#1 | 1012 | chr5_980088_+ |  |  |  |  | 0 | 1 |
| HAM/TSP#1 | 1013 | chr5_99021061_+ |  |  |  |  | 0 | 1 |
| HAM/TSP#1 | 1014 | chr5_99813670_+ |  |  |  |  | 0 | 1 |
| HAM/TSP#1 | 1015 | chr6_10095709_+ |  |  |  |  | 0 | 1 |
| HAM/TSP#1 | 1016 | chr6_101231180_+ |  |  |  |  | 0 | 1 |
| HAM/TSP#1 | 1017 | chr6_102389308_+ |  |  |  |  | 0 | 1 |
| HAM/TSP#1 | 1018 | chr6_103086690_- |  |  |  |  | 0 | 1 |
| HAM/TSP#1 | 1019 | chr6_105628964_+ |  |  |  |  | 0 | 1 |
| HAM/TSP#1 | 1020 | chr6_105651033_- |  |  |  |  | 0 | 1 |
| HAM/TSP#1 | 1021 | chr6_106050097_+ |  |  |  |  | 0 | 1 |
| HAM/TSP#1 | 1022 | chr6_10617086_- |  |  |  |  | 0 | 1 |
| HAM/TSP#1 | 1023 | chr6_106485915_+ |  |  |  |  | 0 | 5 |
| HAM/TSP#1 | 1024 | chr6_106788900_- |  |  |  |  | 0 | 1 |
| HAM/TSP#1 | 1025 | chr6_107780291_+ |  |  |  |  | 0 | 2 |
| HAM/TSP#1 | 1026 | chr6_107847897_+ |  |  |  |  | 0 | 1 |
| HAM/TSP#1 | 1027 | chr6_108123810_- |  |  |  |  | 0 | 6 |
| HAM/TSP#1 | 1028 | chr6_108593385_- |  |  |  |  | 0 | 1 |
| HAM/TSP#1 | 1029 | chr6_109367916_- |  |  |  |  | 0 | 1 |
| HAM/TSP#1 | 1030 | chr6_110256414_- |  |  |  |  | 0 | 4 |
| HAM/TSP#1 | 1031 | chr6_111626687_- |  |  |  |  | 0 | 1 |
| HAM/TSP#1 | 1032 | chr6_114681554_+ |  |  |  |  | 0 | 1 |
| HAM/TSP#1 | 1033 | chr6_115073670_- |  |  |  |  | 0 | 1 |
| HAM/TSP#1 | 1034 | chr6_117024678_+ |  |  |  |  | 0 | 1 |
| HAM/TSP#1 | 1035 | chr6_117585282_+ |  |  |  |  | 0 | 1 |
| HAM/TSP#1 | 1036 | chr6_119095618_+ |  |  |  |  | 0 | 1 |
| HAM/TSP#1 | 1037 | chr6_12054686_+ |  |  |  |  | 0 | 1 |
| HAM/TSP#1 | 1038 | chr6_121609841_- |  |  |  |  | 0 | 5 |
| HAM/TSP#1 | 1039 | chr6_122779867_+ |  |  |  |  | 0 | 1 |
| HAM/TSP#1 | 1040 | chr6_12333304_- |  |  |  |  | 0 | 2 |
| HAM/TSP#1 | 1041 | chr6_125287389_- |  |  |  |  | 0 | 2 |
| HAM/TSP#1 | 1042 | chr6_126124685_+ |  |  |  |  | 0 | 1 |
| HAM/TSP#1 | 1043 | chr6_12691952_- |  |  |  |  | 0 | 1 |
| HAM/TSP#1 | 1044 | chr6_127276876_- |  |  |  |  | 0 | 1 |
| HAM/TSP#1 | 1045 | chr6_127875138_- |  |  |  |  | 0 | 4 |
| HAM/TSP#1 | 1046 | chr6_127986994_- |  |  |  |  | 0 | 3 |
| HAM/TSP#1 | 1047 | chr6_1280923_+ |  |  |  |  | 0 | 1 |
| HAM/TSP#1 | 1048 | chr6_13230153_- |  |  |  |  | 0 | 1 |
| HAM/TSP#1 | 1049 | chr6_132346259_- |  |  |  |  | 0 | 1 |
| HAM/TSP#1 | 1050 | chr6_132749502_- |  |  |  |  | 0 | 1 |
| HAM/TSP#1 | 1051 | chr6_132820994_+ |  |  |  |  | 0 | 1 |
| HAM/TSP#1 | 1052 | chr6_133955806_+ |  |  |  |  | 0 | 1 |
| HAM/TSP#1 | 1053 | chr6_134192320_+ |  |  |  |  | 0 | 1 |
| HAM/TSP#1 | 1054 | chr6_135668197_+ |  |  |  |  | 0 | 2 |
| HAM/TSP#1 | 1055 | chr6_136278263_- |  |  |  |  | 0 | 1 |
| HAM/TSP#1 | 1056 | chr6_136774481_- |  |  |  |  | 0 | 3 |
| HAM/TSP#1 | 1057 | chr6_138759701_- |  |  |  |  | 0 | 1 |
| HAM/TSP#1 | 1058 | chr6_144029687_+ |  |  |  |  | 0 | 1 |
| HAM/TSP#1 | 1059 | chr6_144649843_+ |  |  |  |  | 0 | 1 |
| HAM/TSP#1 | 1060 | chr6_144736657_+ |  |  |  |  | 0 | 1 |
| HAM/TSP#1 | 1061 | chr6_148181167_+ |  |  |  |  | 0 | 1 |
| HAM/TSP#1 | 1062 | chr6_148288019_+ |  |  |  |  | 0 | 1 |
| HAM/TSP#1 | 1063 | chr6_14877865_- |  |  |  |  | 0 | 1 |
| HAM/TSP#1 | 1064 | chr6_150533562_- |  |  |  |  | 0 | 1 |
| HAM/TSP#1 | 1065 | chr6_151959145_- |  |  |  |  | 0 | 1 |
| HAM/TSP#1 | 1066 | chr6_152180101_+ |  |  |  |  | 0 | 1 |
| HAM/TSP#1 | 1067 | chr6_153333675_+ |  |  |  |  | 0 | 1 |
| HAM/TSP#1 | 1068 | chr6_153737972_- |  |  |  |  | 0 | 1 |
| HAM/TSP#1 | 1069 | chr6_154428278_+ |  |  |  |  | 0 | 1 |
| HAM/TSP#1 | 1070 | chr6_154508176_- |  |  |  |  | 0 | 3 |
| HAM/TSP#1 | 1071 | chr6_154734884_- |  |  |  |  | 0 | 6 |
| HAM/TSP#1 | 1072 | chr6_154769299_+ |  |  |  |  | 0 | 13 |
| HAM/TSP#1 | 1073 | chr6_157437156_- |  |  |  |  | 0 | 1 |
| HAM/TSP#1 | 1074 | chr6_158515795_- |  |  |  |  | 0 | 1 |
| HAM/TSP#1 | 1075 | chr6_15992035_- |  |  |  |  | 0 | 2 |
| HAM/TSP#1 | 1076 | chr6_161058090_+ |  |  |  |  | 0 | 1 |
| HAM/TSP#1 | 1077 | chr6_161441992_- |  |  |  |  | 0 | 1 |
| HAM/TSP#1 | 1078 | chr6_161697920_+ |  |  |  |  | 0 | 1 |
| HAM/TSP#1 | 1079 | chr6_164388636_- |  |  |  |  | 0 | 24 |
| HAM/TSP#1 | 1080 | chr6_165492082_+ |  |  |  |  | 0 | 6 |
| HAM/TSP#1 | 1081 | chr6_166826775_+ |  |  |  |  | 0 | 2 |
| HAM/TSP#1 | 1082 | chr6_167053445_+ |  |  |  |  | 0 | 9 |
| HAM/TSP#1 | 1083 | chr6_169661675_- |  |  |  |  | 0 | 1 |
| HAM/TSP#1 | 1084 | chr6_169666720_- |  |  |  |  | 0 | 1 |
| HAM/TSP#1 | 1085 | chr6_169669183_- |  |  |  |  | 0 | 1 |
| HAM/TSP#1 | 1086 | chr6_169729105_+ |  |  |  |  | 0 | 5 |
| HAM/TSP#1 | 1087 | chr6_170570561_- |  |  |  |  | 0 | 1 |
| HAM/TSP#1 | 1088 | chr6_18158807_+ |  |  |  |  | 0 | 1 |
| HAM/TSP#1 | 1089 | chr6_19156900_- |  |  |  |  | 0 | 1 |
| HAM/TSP#1 | 1090 | chr6_21451828_+ |  |  |  |  | 0 | 2 |
| HAM/TSP#1 | 1091 | chr6_21895062_- |  |  |  |  | 0 | 1 |
| HAM/TSP#1 | 1092 | chr6_23601520_+ |  |  |  |  | 0 | 1 |
| HAM/TSP#1 | 1093 | chr6_25703168_- |  |  |  |  | 0 | 1 |
| HAM/TSP#1 | 1094 | chr6_2645848_+ |  |  |  |  | 0 | 1 |
| HAM/TSP#1 | 1095 | chr6_28272422_- |  |  |  |  | 0 | 1 |
| HAM/TSP#1 | 1096 | chr6_28524852_+ |  |  |  |  | 0 | 1 |
| HAM/TSP#1 | 1097 | chr6_28633490_- |  |  |  |  | 0 | 1 |
| HAM/TSP#1 | 1098 | chr6_29967489_- |  |  |  |  | 0 | 1 |
| HAM/TSP#1 | 1099 | chr6_30664027_- |  |  |  |  | 0 | 1 |
| HAM/TSP#1 | 1100 | chr6_31562243_- |  |  |  |  | 0 | 1 |
| HAM/TSP#1 | 1101 | chr6_32242142_- |  |  |  |  | 0 | 1 |
| HAM/TSP#1 | 1102 | chr6_32813604_+ |  |  |  |  | 0 | 1 |
| HAM/TSP#1 | 1103 | chr6_33971633_+ |  |  |  |  | 0 | 1 |
| HAM/TSP#1 | 1104 | chr6_34746330_- |  |  |  |  | 0 | 1 |
| HAM/TSP#1 | 1105 | chr6_35023641_- |  |  |  |  | 0 | 1 |
| HAM/TSP#1 | 1106 | chr6_35522431_+ |  |  |  |  | 1 | 8 |
| HAM/TSP#1 | 1107 | chr6_36651036_- |  |  |  |  | 0 | 1 |
| HAM/TSP#1 | 1108 | chr6_36744473_+ |  |  |  |  | 0 | 6 |
| HAM/TSP#1 | 1109 | chr6_37123461_- |  |  |  |  | 0 | 1 |
| HAM/TSP#1 | 1110 | chr6_372600_- |  |  |  |  | 0 | 1 |
| HAM/TSP#1 | 1111 | chr6_3836821_+ |  |  |  |  | 0 | 1 |
| HAM/TSP#1 | 1112 | chr6_3899115_+ |  |  |  |  | 0 | 1 |
| HAM/TSP#1 | 1113 | chr6_39273185_+ |  |  |  |  | 0 | 1 |
| HAM/TSP#1 | 1114 | chr6_40034328_- |  |  |  |  | 0 | 2 |
| HAM/TSP#1 | 1115 | chr6_40807346_- |  |  |  |  | 0 | 3 |
| HAM/TSP#1 | 1116 | chr6_41126070_+ |  |  |  |  | 0 | 1 |
| HAM/TSP#1 | 1117 | chr6_41170911_- |  |  |  |  | 0 | 1 |
| HAM/TSP#1 | 1118 | chr6_41618689_- |  |  |  |  | 0 | 1 |
| HAM/TSP#1 | 1119 | chr6_41757262_+ |  |  |  |  | 0 | 1 |
| HAM/TSP#1 | 1120 | chr6_42793872_- |  |  |  |  | 0 | 1 |
| HAM/TSP#1 | 1121 | chr6_42978019_+ |  |  |  |  | 0 | 1 |
| HAM/TSP#1 | 1122 | chr6_43455054_+ |  |  |  |  | 0 | 1 |
| HAM/TSP#1 | 1123 | chr6_43493592_- |  |  |  |  | 0 | 1 |
| HAM/TSP#1 | 1124 | chr6_44064083_- |  |  |  |  | 0 | 1 |
| HAM/TSP#1 | 1125 | chr6_44696529_+ |  |  |  |  | 0 | 1 |
| HAM/TSP#1 | 1126 | chr6_45328985_- |  |  |  |  | 0 | 2 |
| HAM/TSP#1 | 1127 | chr6_45853232_+ |  |  |  |  | 0 | 8 |
| HAM/TSP#1 | 1128 | chr6_45925341_+ |  |  |  |  | 0 | 1 |
| HAM/TSP#1 | 1129 | chr6_46733733_+ |  |  |  |  | 0 | 1 |
| HAM/TSP#1 | 1130 | chr6_46745747_+ |  |  |  |  | 0 | 1 |
| HAM/TSP#1 | 1131 | chr6_4677674_- |  |  |  |  | 0 | 1 |
| HAM/TSP#1 | 1132 | chr6_46942891_- |  |  |  |  | 0 | 1 |
| HAM/TSP#1 | 1133 | chr6_48694680_+ |  |  |  |  | 0 | 1 |
| HAM/TSP#1 | 1134 | chr6_49456131_+ |  |  |  |  | 0 | 1 |
| HAM/TSP#1 | 1135 | chr6_511587_- |  |  |  |  | 0 | 1 |
| HAM/TSP#1 | 1136 | chr6_52670719_+ |  |  |  |  | 0 | 6 |
| HAM/TSP#1 | 1137 | chr6_5412123_- |  |  |  |  | 0 | 1 |
| HAM/TSP#1 | 1138 | chr6_5682960_+ |  |  |  |  | 0 | 1 |
| HAM/TSP#1 | 1139 | chr6_57006873_- |  |  |  |  | 0 | 1 |
| HAM/TSP#1 | 1140 | chr6_58047113_+ |  |  |  |  | 0 | 4 |
| HAM/TSP#1 | 1141 | chr6_58239275_- |  |  |  |  | 0 | 3 |
| HAM/TSP#1 | 1142 | chr6_61099273_+ |  |  |  |  | 0 | 1 |
| HAM/TSP#1 | 1143 | chr6_63818588_- |  |  |  |  | 0 | 1 |
| HAM/TSP#1 | 1144 | chr6_63880840_+ |  |  |  |  | 0 | 1 |
| HAM/TSP#1 | 1145 | chr6_64247264_+ |  |  |  |  | 0 | 1 |
| HAM/TSP#1 | 1146 | chr6_64704893_- |  |  |  |  | 1 | 27 |
| HAM/TSP#1 | 1147 | chr6_6638864_+ |  |  |  |  | 0 | 1 |
| HAM/TSP#1 | 1148 | chr6_66389597_- |  |  |  |  | 0 | 1 |
| HAM/TSP#1 | 1149 | chr6_66481946_+ |  |  |  |  | 0 | 2 |
| HAM/TSP#1 | 1150 | chr6_66914452_+ |  |  |  |  | 0 | 2 |
| HAM/TSP#1 | 1151 | chr6_67798503_+ |  |  |  |  | 0 | 1 |
| HAM/TSP#1 | 1152 | chr6_67831912_+ |  |  |  |  | 0 | 1 |
| HAM/TSP#1 | 1153 | chr6_68353120_- |  |  |  |  | 0 | 1 |
| HAM/TSP#1 | 1154 | chr6_68677483_+ |  |  |  |  | 0 | 7 |
| HAM/TSP#1 | 1155 | chr6_70571337_+ |  |  |  |  | 0 | 5 |
| HAM/TSP#1 | 1156 | chr6_72236338_+ |  |  |  |  | 0 | 4 |
| HAM/TSP#1 | 1157 | chr6_72605206_- |  |  |  |  | 0 | 1 |
| HAM/TSP#1 | 1158 | chr6_74406795_- |  |  |  |  | 0 | 3 |
| HAM/TSP#1 | 1159 | chr6_75303634_+ |  |  |  |  | 0 | 1 |
| HAM/TSP#1 | 1160 | chr6_76950548_+ |  |  |  |  | 0 | 1 |
| HAM/TSP#1 | 1161 | chr6_77274575_- |  |  |  |  | 0 | 1 |
| HAM/TSP#1 | 1162 | chr6_77417651_- |  |  |  |  | 0 | 4 |
| HAM/TSP#1 | 1163 | chr6_79005893_+ |  |  |  |  | 0 | 1 |
| HAM/TSP#1 | 1164 | chr6_80866898_- |  |  |  |  | 0 | 2 |
| HAM/TSP#1 | 1165 | chr6_81785384_+ |  |  |  |  | 0 | 1 |
| HAM/TSP#1 | 1166 | chr6_86354974_+ |  |  |  |  | 0 | 15 |
| HAM/TSP#1 | 1167 | chr6_87472771_+ |  |  |  |  | 0 | 3 |
| HAM/TSP#1 | 1168 | chr6_88187207_+ |  |  |  |  | 0 | 1 |
| HAM/TSP#1 | 1169 | chr6_89729591_- |  |  |  |  | 0 | 1 |
| HAM/TSP#1 | 1170 | chr6_91919131_- |  |  |  |  | 0 | 2 |
| HAM/TSP#1 | 1171 | chr6_93560645_- |  |  |  |  | 0 | 1 |
| HAM/TSP#1 | 1172 | chr6_93740805_+ |  |  |  |  | 0 | 1 |
| HAM/TSP#1 | 1173 | chr6_94881581_+ |  |  |  |  | 0 | 1 |
| HAM/TSP#1 | 1174 | chr6_95083815_- |  |  |  |  | 0 | 1 |
| HAM/TSP#1 | 1175 | chr6_95607107_- |  |  |  |  | 0 | 4 |
| HAM/TSP#1 | 1176 | chr6_95779448_+ |  |  |  |  | 0 | 1 |
| HAM/TSP#1 | 1177 | chr6_99025756_- |  |  |  |  | 0 | 1 |
| HAM/TSP#1 | 1178 | chr6_99141578_- |  |  |  |  | 0 | 1 |
| HAM/TSP#1 | 1179 | chr6_99325991_+ |  |  |  |  | 0 | 1 |
| HAM/TSP#1 | 1180 | chr6_99350715_+ |  |  |  |  | 0 | 1 |
| HAM/TSP#1 | 1181 | chr6_99969045_+ |  |  |  |  | 0 | 2 |
| HAM/TSP#1 | 1182 | chr7_100142756_+ |  |  |  |  | 0 | 1 |
| HAM/TSP#1 | 1183 | chr7_102428010_+ |  |  |  |  | 0 | 1 |
| HAM/TSP#1 | 1184 | chr7_102460232_- |  |  |  |  | 0 | 4 |
| HAM/TSP#1 | 1185 | chr7_105240202_- |  |  |  |  | 0 | 1 |
| HAM/TSP#1 | 1186 | chr7_109694129_+ |  |  |  |  | 0 | 1 |
| HAM/TSP#1 | 1187 | chr7_110465730_+ |  |  |  |  | 0 | 1 |
| HAM/TSP#1 | 1188 | chr7_112998431_+ |  |  |  |  | 0 | 1 |
| HAM/TSP#1 | 1189 | chr7_116470_+ |  |  |  |  | 0 | 5 |
| HAM/TSP#1 | 1190 | chr7_116686215_- |  |  |  |  | 0 | 1 |
| HAM/TSP#1 | 1191 | chr7_119567617_+ |  |  |  |  | 0 | 1 |
| HAM/TSP#1 | 1192 | chr7_119578268_- |  |  |  |  | 0 | 1 |
| HAM/TSP#1 | 1193 | chr7_120058342_+ |  |  |  |  | 0 | 1 |
| HAM/TSP#1 | 1194 | chr7_121568777_+ |  |  |  |  | 0 | 2 |
| HAM/TSP#1 | 1195 | chr7_122885242_+ |  |  |  |  | 0 | 1 |
| HAM/TSP#1 | 1196 | chr7_123282358_+ |  |  |  |  | 0 | 2 |
| HAM/TSP#1 | 1197 | chr7_123751766_- |  |  |  |  | 0 | 4 |
| HAM/TSP#1 | 1198 | chr7_123918214_+ |  |  |  |  | 0 | 1 |
| HAM/TSP#1 | 1199 | chr7_124010797_+ |  |  |  |  | 0 | 1 |
| HAM/TSP#1 | 1200 | chr7_12418534_+ |  |  |  |  | 0 | 1 |
| HAM/TSP#1 | 1201 | chr7_125267318_- |  |  |  |  | 0 | 4 |
| HAM/TSP#1 | 1202 | chr7_125941544_+ |  |  |  |  | 0 | 3 |
| HAM/TSP#1 | 1203 | chr7_126253326_+ |  |  |  |  | 0 | 1 |
| HAM/TSP#1 | 1204 | chr7_126636699_- |  |  |  |  | 0 | 2 |
| HAM/TSP#1 | 1205 | chr7_128081589_+ |  |  |  |  | 0 | 1 |
| HAM/TSP#1 | 1206 | chr7_128844544_+ |  |  |  |  | 0 | 1 |
| HAM/TSP#1 | 1207 | chr7_131320738_+ |  |  |  |  | 0 | 1 |
| HAM/TSP#1 | 1208 | chr7_132387042_- |  |  |  |  | 0 | 3 |
| HAM/TSP#1 | 1209 | chr7_132424534_+ |  |  |  |  | 0 | 1 |
| HAM/TSP#1 | 1210 | chr7_133429156_- |  |  |  |  | 0 | 1 |
| HAM/TSP#1 | 1211 | chr7_133933823_- |  |  |  |  | 0 | 1 |
| HAM/TSP#1 | 1212 | chr7_134538391_+ |  |  |  |  | 0 | 2 |
| HAM/TSP#1 | 1213 | chr7_13532211_+ |  |  |  |  | 0 | 1 |
| HAM/TSP#1 | 1214 | chr7_136129201_+ |  |  |  |  | 0 | 1 |
| HAM/TSP#1 | 1215 | chr7_137814828_- |  |  |  |  | 0 | 2 |
| HAM/TSP#1 | 1216 | chr7_137915174_+ |  |  |  |  | 0 | 3 |
| HAM/TSP#1 | 1217 | chr7_139333781_+ |  |  |  |  | 0 | 1 |
| HAM/TSP#1 | 1218 | chr7_13997776_+ |  |  |  |  | 0 | 1 |
| HAM/TSP#1 | 1219 | chr7_140694864_+ |  |  |  |  | 0 | 20 |
| HAM/TSP#1 | 1220 | chr7_141235624_+ |  |  |  |  | 0 | 4 |
| HAM/TSP#1 | 1221 | chr7_142871397_+ |  |  |  |  | 0 | 1 |
| HAM/TSP#1 | 1222 | chr7_143359545_+ |  |  |  |  | 0 | 1 |
| HAM/TSP#1 | 1223 | chr7_145515375_- |  |  |  |  | 0 | 1 |
| HAM/TSP#1 | 1224 | chr7_147423430_- |  |  |  |  | 0 | 1 |
| HAM/TSP#1 | 1225 | chr7_148932369_+ |  |  |  |  | 0 | 2 |
| HAM/TSP#1 | 1226 | chr7_1499091_- |  |  |  |  | 0 | 2 |
| HAM/TSP#1 | 1227 | chr7_150594456_+ |  |  |  |  | 0 | 1 |
| HAM/TSP#1 | 1228 | chr7_1519005_- |  |  |  |  | 0 | 1 |
| HAM/TSP#1 | 1229 | chr7_153060691_- |  |  |  |  | 0 | 1 |
| HAM/TSP#1 | 1230 | chr7_154097704_+ |  |  |  |  | 0 | 1 |
| HAM/TSP#1 | 1231 | chr7_15466447_+ |  |  |  |  | 0 | 3 |
| HAM/TSP#1 | 1232 | chr7_15685667_+ |  |  |  |  | 0 | 1 |
| HAM/TSP#1 | 1233 | chr7_157158470_+ |  |  |  |  | 0 | 1 |
| HAM/TSP#1 | 1234 | chr7_157303329_- |  |  |  |  | 0 | 1 |
| HAM/TSP#1 | 1235 | chr7_157894292_- |  |  |  |  | 0 | 1 |
| HAM/TSP#1 | 1236 | chr7_158343615_- |  |  |  |  | 0 | 2 |
| HAM/TSP#1 | 1237 | chr7_158815712_- |  |  |  |  | 0 | 1 |
| HAM/TSP#1 | 1238 | chr7_159100996_+ |  |  |  |  | 0 | 1 |
| HAM/TSP#1 | 1239 | chr7_16619572_- |  |  |  |  | 0 | 4 |
| HAM/TSP#1 | 1240 | chr7_17675137_+ |  |  |  |  | 0 | 1 |
| HAM/TSP#1 | 1241 | chr7_17791863_- |  |  |  |  | 0 | 3 |
| HAM/TSP#1 | 1242 | chr7_19219352_- |  |  |  |  | 0 | 5 |
| HAM/TSP#1 | 1243 | chr7_1943067_- |  |  |  |  | 0 | 1 |
| HAM/TSP#1 | 1244 | chr7_21311468_- |  |  |  |  | 0 | 1 |
| HAM/TSP#1 | 1245 | chr7_21347804_- |  |  |  |  | 0 | 1 |
| HAM/TSP#1 | 1246 | chr7_21958908_- |  |  |  |  | 0 | 1 |
| HAM/TSP#1 | 1247 | chr7_22175495_+ |  |  |  |  | 0 | 1 |
| HAM/TSP#1 | 1248 | chr7_22269010_- |  |  |  |  | 0 | 4 |
| HAM/TSP#1 | 1249 | chr7_22387097_+ |  |  |  |  | 0 | 17 |
| HAM/TSP#1 | 1250 | chr7_23029065_- |  |  |  |  | 0 | 2 |
| HAM/TSP#1 | 1251 | chr7_23311794_+ |  |  |  |  | 0 | 1 |
| HAM/TSP#1 | 1252 | chr7_2488715_- |  |  |  |  | 0 | 4 |
| HAM/TSP#1 | 1253 | chr7_24899144_+ |  |  |  |  | 0 | 1 |
| HAM/TSP#1 | 1254 | chr7_26390433_- |  |  |  |  | 0 | 1 |
| HAM/TSP#1 | 1255 | chr7_26390434_- |  |  |  |  | 0 | 1 |
| HAM/TSP#1 | 1256 | chr7_27036409_+ |  |  |  |  | 0 | 6 |
| HAM/TSP#1 | 1257 | chr7_30318283_+ |  |  |  |  | 0 | 1 |
| HAM/TSP#1 | 1258 | chr7_31257453_+ |  |  |  |  | 0 | 1 |
| HAM/TSP#1 | 1259 | chr7_33122345_- |  |  |  |  | 0 | 1 |
| HAM/TSP#1 | 1260 | chr7_3322617_- |  |  |  |  | 0 | 5 |
| HAM/TSP#1 | 1261 | chr7_34622020_- |  |  |  |  | 0 | 19 |
| HAM/TSP#1 | 1262 | chr7_35508050_- |  |  |  |  | 0 | 4 |
| HAM/TSP#1 | 1263 | chr7_35682159_+ |  |  |  |  | 0 | 1 |
| HAM/TSP#1 | 1264 | chr7_36706808_- |  |  |  |  | 0 | 1 |
| HAM/TSP#1 | 1265 | chr7_36777479_+ |  |  |  |  | 0 | 7 |
| HAM/TSP#1 | 1266 | chr7_37717588_- |  |  |  |  | 2 | 0 |
| HAM/TSP#1 | 1267 | chr7_41224512_- |  |  |  |  | 0 | 1 |
| HAM/TSP#1 | 1268 | chr7_41848678_- |  |  |  |  | 0 | 1 |
| HAM/TSP#1 | 1269 | chr7_41995158_+ |  |  |  |  | 0 | 1 |
| HAM/TSP#1 | 1270 | chr7_42201252_+ |  |  |  |  | 0 | 3 |
| HAM/TSP#1 | 1271 | chr7_42365051_+ |  |  |  |  | 0 | 1 |
| HAM/TSP#1 | 1272 | chr7_42521496_- |  |  |  |  | 0 | 1 |
| HAM/TSP#1 | 1273 | chr7_43595811_+ |  |  |  |  | 0 | 17 |
| HAM/TSP#1 | 1274 | chr7_43694075_+ |  |  |  |  | 0 | 1 |
| HAM/TSP#1 | 1275 | chr7_43975158_+ |  |  |  |  | 0 | 1 |
| HAM/TSP#1 | 1276 | chr7_44217922_- |  |  |  |  | 0 | 2 |
| HAM/TSP#1 | 1277 | chr7_46630505_- |  |  |  |  | 0 | 1 |
| HAM/TSP#1 | 1278 | chr7_46662580_+ |  |  |  |  | 0 | 1 |
| HAM/TSP#1 | 1279 | chr7_471453_- |  |  |  |  | 0 | 1 |
| HAM/TSP#1 | 1280 | chr7_47415968_+ |  |  |  |  | 0 | 1 |
| HAM/TSP#1 | 1281 | chr7_47416633_+ |  |  |  |  | 0 | 1 |
| HAM/TSP#1 | 1282 | chr7_48044825_+ |  |  |  |  | 0 | 13 |
| HAM/TSP#1 | 1283 | chr7_49099326_+ |  |  |  |  | 0 | 1 |
| HAM/TSP#1 | 1284 | chr7_49380967_+ |  |  |  |  | 0 | 1 |
| HAM/TSP#1 | 1285 | chr7_50357469_+ |  |  |  |  | 0 | 1 |
| HAM/TSP#1 | 1286 | chr7_5192028_- |  |  |  |  | 0 | 2 |
| HAM/TSP#1 | 1287 | chr7_52010518_+ |  |  |  |  | 0 | 1 |
| HAM/TSP#1 | 1288 | chr7_52149299_- |  |  |  |  | 0 | 2 |
| HAM/TSP#1 | 1289 | chr7_54066114_- |  |  |  |  | 0 | 1 |
| HAM/TSP#1 | 1290 | chr7_54996061_+ |  |  |  |  | 0 | 2 |
| HAM/TSP#1 | 1291 | chr7_55050978_+ |  |  |  |  | 0 | 1 |
| HAM/TSP#1 | 1292 | chr7_55561216_- |  |  |  |  | 0 | 31 |
| HAM/TSP#1 | 1293 | chr7_56056283_+ |  |  |  |  | 0 | 7 |
| HAM/TSP#1 | 1294 | chr7_5687744_+ |  |  |  |  | 0 | 1 |
| HAM/TSP#1 | 1295 | chr7_57995618_- |  |  |  |  | 0 | 6 |
| HAM/TSP#1 | 1296 | chr7_58018680_- |  |  |  |  | 0 | 1 |
| HAM/TSP#1 | 1297 | chr7_63054200_+ |  |  |  |  | 0 | 1 |
| HAM/TSP#1 | 1298 | chr7_63580660_+ |  |  |  |  | 0 | 1 |
| HAM/TSP#1 | 1299 | chr7_64556499_+ |  |  |  |  | 0 | 2 |
| HAM/TSP#1 | 1300 | chr7_6569663_+ |  |  |  |  | 0 | 2 |
| HAM/TSP#1 | 1301 | chr7_65992011_- |  |  |  |  | 0 | 1 |
| HAM/TSP#1 | 1302 | chr7_69365431_+ |  |  |  |  | 0 | 1 |
| HAM/TSP#1 | 1303 | chr7_69558526_+ |  |  |  |  | 0 | 1 |
| HAM/TSP#1 | 1304 | chr7_69603264_+ |  |  |  |  | 0 | 1 |
| HAM/TSP#1 | 1305 | chr7_70692687_- |  |  |  |  | 0 | 1 |
| HAM/TSP#1 | 1306 | chr7_7169104_- |  |  |  |  | 0 | 8 |
| HAM/TSP#1 | 1307 | chr7_72027492_- |  |  |  |  | 0 | 1 |
| HAM/TSP#1 | 1308 | chr7_7205662_- |  |  |  |  | 0 | 3 |
| HAM/TSP#1 | 1309 | chr7_73919562_+ |  |  |  |  | 0 | 1 |
| HAM/TSP#1 | 1310 | chr7_74464256_- |  |  |  |  | 0 | 1 |
| HAM/TSP#1 | 1311 | chr7_7561827_+ |  |  |  |  | 0 | 1 |
| HAM/TSP#1 | 1312 | chr7_75841436_- |  |  |  |  | 0 | 1 |
| HAM/TSP#1 | 1313 | chr7_77174347_+ |  |  |  |  | 0 | 1 |
| HAM/TSP#1 | 1314 | chr7_7896328_- |  |  |  |  | 0 | 1 |
| HAM/TSP#1 | 1315 | chr7_79086215_- |  |  |  |  | 0 | 1 |
| HAM/TSP#1 | 1316 | chr7_80096947_+ |  |  |  |  | 0 | 1 |
| HAM/TSP#1 | 1317 | chr7_80270210_+ |  |  |  |  | 0 | 1 |
| HAM/TSP#1 | 1318 | chr7_8172693_- |  |  |  |  | 0 | 2 |
| HAM/TSP#1 | 1319 | chr7_81950233_- |  |  |  |  | 0 | 1 |
| HAM/TSP#1 | 1320 | chr7_82062640_- |  |  |  |  | 0 | 1 |
| HAM/TSP#1 | 1321 | chr7_83812108_+ |  |  |  |  | 0 | 1 |
| HAM/TSP#1 | 1322 | chr7_86089131_- |  |  |  |  | 0 | 1 |
| HAM/TSP#1 | 1323 | chr7_88876280_+ |  |  |  |  | 0 | 3 |
| HAM/TSP#1 | 1324 | chr7_904419_- |  |  |  |  | 0 | 1 |
| HAM/TSP#1 | 1325 | chr7_91921507_+ |  |  |  |  | 0 | 1 |
| HAM/TSP#1 | 1326 | chr7_93000402_- |  |  |  |  | 0 | 1 |
| HAM/TSP#1 | 1327 | chr7_93272356_+ |  |  |  |  | 0 | 1 |
| HAM/TSP#1 | 1328 | chr7_94158697_+ |  |  |  |  | 0 | 2 |
| HAM/TSP#1 | 1329 | chr7_94532840_- |  |  |  |  | 0 | 1 |
| HAM/TSP#1 | 1330 | chr7_9537412_+ |  |  |  |  | 0 | 6 |
| HAM/TSP#1 | 1331 | chr7_97966377_+ |  |  |  |  | 0 | 1 |
| HAM/TSP#1 | 1332 | chr7_9900112_+ |  |  |  |  | 0 | 4 |
| HAM/TSP#1 | 1333 | chr7_9935647_+ |  |  |  |  | 0 | 3 |
| HAM/TSP#1 | 1334 | chr8_10197414_- |  |  |  |  | 0 | 1 |
| HAM/TSP#1 | 1335 | chr8_101984000_- |  |  |  |  | 0 | 14 |
| HAM/TSP#1 | 1336 | chr8_102601168_- |  |  |  |  | 0 | 1 |
| HAM/TSP#1 | 1337 | chr8_102959990_- |  |  |  |  | 0 | 1 |
| HAM/TSP#1 | 1338 | chr8_103838228_+ |  |  |  |  | 0 | 1 |
| HAM/TSP#1 | 1339 | chr8_10421296_- |  |  |  |  | 0 | 1 |
| HAM/TSP#1 | 1340 | chr8_104794542_+ |  |  |  |  | 0 | 1 |
| HAM/TSP#1 | 1341 | chr8_105182943_+ |  |  |  |  | 0 | 1 |
| HAM/TSP#1 | 1342 | chr8_105553630_+ |  |  |  |  | 0 | 1 |
| HAM/TSP#1 | 1343 | chr8_10621473_- |  |  |  |  | 0 | 1 |
| HAM/TSP#1 | 1344 | chr8_107667257_- |  |  |  |  | 0 | 1 |
| HAM/TSP#1 | 1345 | chr8_107710781_- |  |  |  |  | 0 | 1 |
| HAM/TSP#1 | 1346 | chr8_108216815_+ |  |  |  |  | 0 | 1 |
| HAM/TSP#1 | 1347 | chr8_10929157_- |  |  |  |  | 0 | 1 |
| HAM/TSP#1 | 1348 | chr8_109342328_+ |  |  |  |  | 0 | 2 |
| HAM/TSP#1 | 1349 | chr8_109985119_+ |  |  |  |  | 0 | 2 |
| HAM/TSP#1 | 1350 | chr8_11355461_+ |  |  |  |  | 0 | 4 |
| HAM/TSP#1 | 1351 | chr8_1139794_- |  |  |  |  | 0 | 1 |
| HAM/TSP#1 | 1352 | chr8_117231810_+ |  |  |  |  | 0 | 4 |
| HAM/TSP#1 | 1353 | chr8_117728281_- |  |  |  |  | 0 | 2 |
| HAM/TSP#1 | 1354 | chr8_118304655_+ |  |  |  |  | 0 | 1 |
| HAM/TSP#1 | 1355 | chr8_118499364_- |  |  |  |  | 0 | 1 |
| HAM/TSP#1 | 1356 | chr8_119750047_- |  |  |  |  | 0 | 1 |
| HAM/TSP#1 | 1357 | chr8_120739452_+ |  |  |  |  | 0 | 1 |
| HAM/TSP#1 | 1358 | chr8_121679566_- |  |  |  |  | 0 | 1 |
| HAM/TSP#1 | 1359 | chr8_123441806_- |  |  |  |  | 0 | 1 |
| HAM/TSP#1 | 1360 | chr8_123960211_+ |  |  |  |  | 0 | 1 |
| HAM/TSP#1 | 1361 | chr8_125098367_+ |  |  |  |  | 0 | 1 |
| HAM/TSP#1 | 1362 | chr8_125163983_+ |  |  |  |  | 0 | 1 |
| HAM/TSP#1 | 1363 | chr8_125381041_- |  |  |  |  | 0 | 1 |
| HAM/TSP#1 | 1364 | chr8_125388117_- |  |  |  |  | 0 | 1 |
| HAM/TSP#1 | 1365 | chr8_12612271_- |  |  |  |  | 0 | 1 |
| HAM/TSP#1 | 1366 | chr8_128651747_- |  |  |  |  | 0 | 1 |
| HAM/TSP#1 | 1367 | chr8_130534024_+ |  |  |  |  | 0 | 1 |
| HAM/TSP#1 | 1368 | chr8_131963243_- |  |  |  |  | 0 | 1 |
| HAM/TSP#1 | 1369 | chr8_132417796_- |  |  |  |  | 0 | 2 |
| HAM/TSP#1 | 1370 | chr8_133072450_- |  |  |  |  | 0 | 5 |
| HAM/TSP#1 | 1371 | chr8_133619045_- |  |  |  |  | 0 | 1 |
| HAM/TSP#1 | 1372 | chr8_134812503_- |  |  |  |  | 0 | 1 |
| HAM/TSP#1 | 1373 | chr8_135041146_- |  |  |  |  | 0 | 1 |
| HAM/TSP#1 | 1374 | chr8_135054521_- |  |  |  |  | 0 | 1 |
| HAM/TSP#1 | 1375 | chr8_135685809_- |  |  |  |  | 0 | 1 |
| HAM/TSP#1 | 1376 | chr8_138646807_- |  |  |  |  | 0 | 1 |
| HAM/TSP#1 | 1377 | chr8_140311995_+ |  |  |  |  | 0 | 1 |
| HAM/TSP#1 | 1378 | chr8_140775870_- |  |  |  |  | 0 | 1 |
| HAM/TSP#1 | 1379 | chr8_141431821_- |  |  |  |  | 0 | 1 |
| HAM/TSP#1 | 1380 | chr8_142034760_- |  |  |  |  | 0 | 1 |
| HAM/TSP#1 | 1381 | chr8_142209740_- |  |  |  |  | 0 | 1 |
| HAM/TSP#1 | 1382 | chr8_142322630_+ |  |  |  |  | 0 | 1 |
| HAM/TSP#1 | 1383 | chr8_142489486_- |  |  |  |  | 0 | 1 |
| HAM/TSP#1 | 1384 | chr8_142633473_+ |  |  |  |  | 0 | 1 |
| HAM/TSP#1 | 1385 | chr8_142780044_+ |  |  |  |  | 0 | 1 |
| HAM/TSP#1 | 1386 | chr8_143200891_- |  |  |  |  | 0 | 1 |
| HAM/TSP#1 | 1387 | chr8_144320353_- |  |  |  |  | 0 | 1 |
| HAM/TSP#1 | 1388 | chr8_144483660_- |  |  |  |  | 0 | 1 |
| HAM/TSP#1 | 1389 | chr8_144512454_+ |  |  |  |  | 0 | 1 |
| HAM/TSP#1 | 1390 | chr8_15298413_+ |  |  |  |  | 0 | 1 |
| HAM/TSP#1 | 1391 | chr8_15620669_+ |  |  |  |  | 0 | 1 |
| HAM/TSP#1 | 1392 | chr8_15940472_- |  |  |  |  | 0 | 3 |
| HAM/TSP#1 | 1393 | chr8_16363828_- |  |  |  |  | 0 | 1 |
| HAM/TSP#1 | 1394 | chr8_17236179_+ |  |  |  |  | 0 | 1 |
| HAM/TSP#1 | 1395 | chr8_18174875_+ |  |  |  |  | 0 | 1 |
| HAM/TSP#1 | 1396 | chr8_18538807_- |  |  |  |  | 0 | 4 |
| HAM/TSP#1 | 1397 | chr8_18976571_- |  |  |  |  | 0 | 1 |
| HAM/TSP#1 | 1398 | chr8_19620198_- |  |  |  |  | 0 | 2 |
| HAM/TSP#1 | 1399 | chr8_21313955_+ |  |  |  |  | 0 | 1 |
| HAM/TSP#1 | 1400 | chr8_21729933_- |  |  |  |  | 0 | 1 |
| HAM/TSP#1 | 1401 | chr8_23135192_+ |  |  |  |  | 0 | 1 |
| HAM/TSP#1 | 1402 | chr8_23562991_+ |  |  |  |  | 0 | 1 |
| HAM/TSP#1 | 1403 | chr8_23673703_- |  |  |  |  | 0 | 1 |
| HAM/TSP#1 | 1404 | chr8_26217204_+ |  |  |  |  | 0 | 1 |
| HAM/TSP#1 | 1405 | chr8_26532922_+ |  |  |  |  | 0 | 1 |
| HAM/TSP#1 | 1406 | chr8_26638509_- |  |  |  |  | 0 | 1 |
| HAM/TSP#1 | 1407 | chr8_2777754_- |  |  |  |  | 0 | 1 |
| HAM/TSP#1 | 1408 | chr8_28152163_+ |  |  |  |  | 0 | 1 |
| HAM/TSP#1 | 1409 | chr8_28597589_+ |  |  |  |  | 0 | 1 |
| HAM/TSP#1 | 1410 | chr8_29521656_+ |  |  |  |  | 0 | 1 |
| HAM/TSP#1 | 1411 | chr8_2977987_- |  |  |  |  | 0 | 1 |
| HAM/TSP#1 | 1412 | chr8_3088261_- |  |  |  |  | 0 | 1 |
| HAM/TSP#1 | 1413 | chr8_3153418_+ |  |  |  |  | 0 | 1 |
| HAM/TSP#1 | 1414 | chr8_3162088_+ |  |  |  |  | 0 | 1 |
| HAM/TSP#1 | 1415 | chr8_32258035_- |  |  |  |  | 0 | 3 |
| HAM/TSP#1 | 1416 | chr8_33123956_+ |  |  |  |  | 0 | 1 |
| HAM/TSP#1 | 1417 | chr8_33128818_- |  |  |  |  | 0 | 1 |
| HAM/TSP#1 | 1418 | chr8_33388152_- |  |  |  |  | 0 | 1 |
| HAM/TSP#1 | 1419 | chr8_33777442_- |  |  |  |  | 0 | 1 |
| HAM/TSP#1 | 1420 | chr8_35138037_- |  |  |  |  | 0 | 1 |
| HAM/TSP#1 | 1421 | chr8_36500619_- |  |  |  |  | 0 | 1 |
| HAM/TSP#1 | 1422 | chr8_40683773_- |  |  |  |  | 0 | 1 |
| HAM/TSP#1 | 1423 | chr8_40804692_- |  |  |  |  | 0 | 1 |
| HAM/TSP#1 | 1424 | chr8_42186587_- |  |  |  |  | 0 | 1 |
| HAM/TSP#1 | 1425 | chr8_4236842_+ |  |  |  |  | 0 | 1 |
| HAM/TSP#1 | 1426 | chr8_43408458_+ |  |  |  |  | 0 | 1 |
| HAM/TSP#1 | 1427 | chr8_43609701_+ |  |  |  |  | 0 | 2 |
| HAM/TSP#1 | 1428 | chr8_43692451_- |  |  |  |  | 0 | 1 |
| HAM/TSP#1 | 1429 | chr8_46037681_- |  |  |  |  | 0 | 13 |
| HAM/TSP#1 | 1430 | chr8_46225198_+ |  |  |  |  | 0 | 1 |
| HAM/TSP#1 | 1431 | chr8_46225318_- |  |  |  |  | 0 | 1 |
| HAM/TSP#1 | 1432 | chr8_46541222_- |  |  |  |  | 0 | 2 |
| HAM/TSP#1 | 1433 | chr8_46923595_- |  |  |  |  | 0 | 1 |
| HAM/TSP#1 | 1434 | chr8_4713333_- |  |  |  |  | 0 | 1 |
| HAM/TSP#1 | 1435 | chr8_47923187_+ |  |  |  |  | 0 | 2 |
| HAM/TSP#1 | 1436 | chr8_48590121_+ |  |  |  |  | 0 | 1 |
| HAM/TSP#1 | 1437 | chr8_49219088_- |  |  |  |  | 0 | 11 |
| HAM/TSP#1 | 1438 | chr8_50686109_- |  |  |  |  | 0 | 1 |
| HAM/TSP#1 | 1439 | chr8_50906545_- |  |  |  |  | 0 | 1 |
| HAM/TSP#1 | 1440 | chr8_51456480_- |  |  |  |  | 0 | 1 |
| HAM/TSP#1 | 1441 | chr8_51494375_+ |  |  |  |  | 0 | 1 |
| HAM/TSP#1 | 1442 | chr8_52354119_+ |  |  |  |  | 0 | 1 |
| HAM/TSP#1 | 1443 | chr8_53707454_+ |  |  |  |  | 0 | 1 |
| HAM/TSP#1 | 1444 | chr8_54136781_+ |  |  |  |  | 0 | 5 |
| HAM/TSP#1 | 1445 | chr8_54822086_+ |  |  |  |  | 0 | 1 |
| HAM/TSP#1 | 1446 | chr8_55080454_- |  |  |  |  | 0 | 1 |
| HAM/TSP#1 | 1447 | chr8_55239708_- |  |  |  |  | 0 | 2 |
| HAM/TSP#1 | 1448 | chr8_55449537_- |  |  |  |  | 0 | 1 |
| HAM/TSP#1 | 1449 | chr8_58731440_- |  |  |  |  | 0 | 2 |
| HAM/TSP#1 | 1450 | chr8_59901937_- |  |  |  |  | 0 | 2 |
| HAM/TSP#1 | 1451 | chr8_6036582_+ |  |  |  |  | 0 | 17 |
| HAM/TSP#1 | 1452 | chr8_60910846_+ |  |  |  |  | 0 | 1 |
| HAM/TSP#1 | 1453 | chr8_62625780_+ |  |  |  |  | 0 | 2 |
| HAM/TSP#1 | 1454 | chr8_62662464_- |  |  |  |  | 0 | 1 |
| HAM/TSP#1 | 1455 | chr8_637915_- |  |  |  |  | 0 | 1 |
| HAM/TSP#1 | 1456 | chr8_63810793_- |  |  |  |  | 0 | 3 |
| HAM/TSP#1 | 1457 | chr8_65333374_- |  |  |  |  | 0 | 1 |
| HAM/TSP#1 | 1458 | chr8_6598285_+ |  |  |  |  | 0 | 1 |
| HAM/TSP#1 | 1459 | chr8_66167683_+ |  |  |  |  | 0 | 1 |
| HAM/TSP#1 | 1460 | chr8_6655823_- |  |  |  |  | 0 | 1 |
| HAM/TSP#1 | 1461 | chr8_67055919_+ |  |  |  |  | 0 | 5 |
| HAM/TSP#1 | 1462 | chr8_6903940_- |  |  |  |  | 0 | 1 |
| HAM/TSP#1 | 1463 | chr8_69871574_+ |  |  |  |  | 0 | 5 |
| HAM/TSP#1 | 1464 | chr8_70643360_+ |  |  |  |  | 0 | 1 |
| HAM/TSP#1 | 1465 | chr8_71843663_+ |  |  |  |  | 0 | 1 |
| HAM/TSP#1 | 1466 | chr8_73520142_- |  |  |  |  | 0 | 3 |
| HAM/TSP#1 | 1467 | chr8_73829220_- |  |  |  |  | 0 | 4 |
| HAM/TSP#1 | 1468 | chr8_73935649_- |  |  |  |  | 0 | 4 |
| HAM/TSP#1 | 1469 | chr8_74474344_+ |  |  |  |  | 0 | 1 |
| HAM/TSP#1 | 1470 | chr8_74693706_+ |  |  |  |  | 0 | 1 |
| HAM/TSP#1 | 1471 | chr8_75765398_- |  |  |  |  | 0 | 1 |
| HAM/TSP#1 | 1472 | chr8_75785183_+ |  |  |  |  | 0 | 1 |
| HAM/TSP#1 | 1473 | chr8_77001982_- |  |  |  |  | 0 | 7 |
| HAM/TSP#1 | 1474 | chr8_77010545_- |  |  |  |  | 0 | 11 |
| HAM/TSP#1 | 1475 | chr8_78345176_+ |  |  |  |  | 0 | 1 |
| HAM/TSP#1 | 1476 | chr8_80046887_+ |  |  |  |  | 0 | 7 |
| HAM/TSP#1 | 1477 | chr8_81154229_+ |  |  |  |  | 0 | 1 |
| HAM/TSP#1 | 1478 | chr8_81278570_+ |  |  |  |  | 0 | 1 |
| HAM/TSP#1 | 1479 | chr8_82468683_+ |  |  |  |  | 0 | 1 |
| HAM/TSP#1 | 1480 | chr8_82510256_+ |  |  |  |  | 0 | 2 |
| HAM/TSP#1 | 1481 | chr8_83723112_- |  |  |  |  | 0 | 1 |
| HAM/TSP#1 | 1482 | chr8_87668844_+ |  |  |  |  | 0 | 12 |
| HAM/TSP#1 | 1483 | chr8_88101486_+ |  |  |  |  | 0 | 1 |
| HAM/TSP#1 | 1484 | chr8_89355178_- |  |  |  |  | 0 | 7 |
| HAM/TSP#1 | 1485 | chr8_90356455_- |  |  |  |  | 0 | 1 |
| HAM/TSP#1 | 1486 | chr8_90412295_- |  |  |  |  | 0 | 1 |
| HAM/TSP#1 | 1487 | chr8_93991467_+ |  |  |  |  | 0 | 1 |
| HAM/TSP#1 | 1488 | chr8_94106064_+ |  |  |  |  | 0 | 1 |
| HAM/TSP#1 | 1489 | chr8_96130976_+ |  |  |  |  | 0 | 1 |
| HAM/TSP#1 | 1490 | chr8_96263134_- |  |  |  |  | 0 | 1 |
| HAM/TSP#1 | 1491 | chr8_98657298_- |  |  |  |  | 0 | 1 |
| HAM/TSP#1 | 1492 | chr8_99016889_- |  |  |  |  | 0 | 1 |
| HAM/TSP#1 | 1493 | chr8_99631372_- |  |  |  |  | 0 | 1 |
| HAM/TSP#1 | 1494 | chr9_101174809_+ |  |  |  |  | 0 | 1 |
| HAM/TSP#1 | 1495 | chr9_102450051_+ |  |  |  |  | 0 | 1 |
| HAM/TSP#1 | 1496 | chr9_103459976_+ |  |  |  |  | 0 | 1 |
| HAM/TSP#1 | 1497 | chr9_105516038_+ |  |  |  |  | 0 | 1 |
| HAM/TSP#1 | 1498 | chr9_105711536_+ |  |  |  |  | 0 | 4 |
| HAM/TSP#1 | 1499 | chr9_106575885_+ |  |  |  |  | 0 | 1 |
| HAM/TSP#1 | 1500 | chr9_106937324_- |  |  |  |  | 0 | 1 |
| HAM/TSP#1 | 1501 | chr9_107164859_+ |  |  |  |  | 0 | 2 |
| HAM/TSP#1 | 1502 | chr9_10887511_- |  |  |  |  | 0 | 1 |
| HAM/TSP#1 | 1503 | chr9_109163832_- |  |  |  |  | 0 | 1 |
| HAM/TSP#1 | 1504 | chr9_109306932_+ |  |  |  |  | 0 | 1 |
| HAM/TSP#1 | 1505 | chr9_109586710_- |  |  |  |  | 0 | 1 |
| HAM/TSP#1 | 1506 | chr9_109844694_+ |  |  |  |  | 0 | 4 |
| HAM/TSP#1 | 1507 | chr9_110573893_- |  |  |  |  | 1 | 0 |
| HAM/TSP#1 | 1508 | chr9_111702690_- |  |  |  |  | 0 | 12 |
| HAM/TSP#1 | 1509 | chr9_113384417_+ |  |  |  |  | 0 | 15 |
| HAM/TSP#1 | 1510 | chr9_113476773_- |  |  |  |  | 0 | 1 |
| HAM/TSP#1 | 1511 | chr9_113650499_+ |  |  |  |  | 0 | 1 |
| HAM/TSP#1 | 1512 | chr9_113778316_- |  |  |  |  | 0 | 1 |
| HAM/TSP#1 | 1513 | chr9_114088_+ |  |  |  |  | 0 | 7 |
| HAM/TSP#1 | 1514 | chr9_12029741_- |  |  |  |  | 0 | 1 |
| HAM/TSP#1 | 1515 | chr9_120387955_+ |  |  |  |  | 0 | 1 |
| HAM/TSP#1 | 1516 | chr9_122201470_+ |  |  |  |  | 0 | 1 |
| HAM/TSP#1 | 1517 | chr9_122594105_+ |  |  |  |  | 0 | 1 |
| HAM/TSP#1 | 1518 | chr9_123094609_+ |  |  |  |  | 0 | 3 |
| HAM/TSP#1 | 1519 | chr9_124271672_+ |  |  |  |  | 0 | 1 |
| HAM/TSP#1 | 1520 | chr9_124457512_+ |  |  |  |  | 0 | 1 |
| HAM/TSP#1 | 1521 | chr9_125306708_+ |  |  |  |  | 0 | 1 |
| HAM/TSP#1 | 1522 | chr9_126506872_- |  |  |  |  | 0 | 1 |
| HAM/TSP#1 | 1523 | chr9_128589988_+ |  |  |  |  | 0 | 1 |
| HAM/TSP#1 | 1524 | chr9_128923717_- |  |  |  |  | 0 | 1 |
| HAM/TSP#1 | 1525 | chr9_128993380_- |  |  |  |  | 0 | 1 |
| HAM/TSP#1 | 1526 | chr9_129185337_+ |  |  |  |  | 0 | 1 |
| HAM/TSP#1 | 1527 | chr9_131198537_+ |  |  |  |  | 0 | 7 |
| HAM/TSP#1 | 1528 | chr9_131198538_+ |  |  |  |  | 0 | 1 |
| HAM/TSP#1 | 1529 | chr9_132328000_- |  |  |  |  | 0 | 1 |
| HAM/TSP#1 | 1530 | chr9_132474362_+ |  |  |  |  | 0 | 1 |
| HAM/TSP#1 | 1531 | chr9_132656475_+ |  |  |  |  | 0 | 1 |
| HAM/TSP#1 | 1532 | chr9_1332535_+ |  |  |  |  | 0 | 46 |
| HAM/TSP#1 | 1533 | chr9_133438218_- |  |  |  |  | 0 | 1 |
| HAM/TSP#1 | 1534 | chr9_133866241_+ |  |  |  |  | 0 | 1 |
| HAM/TSP#1 | 1535 | chr9_133936756_+ |  |  |  |  | 0 | 1 |
| HAM/TSP#1 | 1536 | chr9_134954710_+ |  |  |  |  | 0 | 1 |
| HAM/TSP#1 | 1537 | chr9_135705501_- |  |  |  |  | 0 | 1 |
| HAM/TSP#1 | 1538 | chr9_135908087_+ |  |  |  |  | 0 | 1 |
| HAM/TSP#1 | 1539 | chr9_137465360_- |  |  |  |  | 0 | 1 |
| HAM/TSP#1 | 1540 | chr9_137744781_+ |  |  |  |  | 0 | 1 |
| HAM/TSP#1 | 1541 | chr9_14004895_+ |  |  |  |  | 0 | 1 |
| HAM/TSP#1 | 1542 | chr9_14346629_- |  |  |  |  | 0 | 1 |
| HAM/TSP#1 | 1543 | chr9_14453346_+ |  |  |  |  | 0 | 4 |
| HAM/TSP#1 | 1544 | chr9_15486198_- |  |  |  |  | 0 | 1 |
| HAM/TSP#1 | 1545 | chr9_18384686_- |  |  |  |  | 0 | 2 |
| HAM/TSP#1 | 1546 | chr9_1901054_+ |  |  |  |  | 0 | 3 |
| HAM/TSP#1 | 1547 | chr9_19318111_+ |  |  |  |  | 0 | 1 |
| HAM/TSP#1 | 1548 | chr9_19499954_+ |  |  |  |  | 0 | 2 |
| HAM/TSP#1 | 1549 | chr9_2026141_- |  |  |  |  | 0 | 2 |
| HAM/TSP#1 | 1550 | chr9_2095006_+ |  |  |  |  | 0 | 7 |
| HAM/TSP#1 | 1551 | chr9_21172766_- |  |  |  |  | 0 | 2 |
| HAM/TSP#1 | 1552 | chr9_21601733_+ |  |  |  |  | 0 | 1 |
| HAM/TSP#1 | 1553 | chr9_22579144_+ |  |  |  |  | 0 | 2 |
| HAM/TSP#1 | 1554 | chr9_24732108_+ |  |  |  |  | 0 | 2 |
| HAM/TSP#1 | 1555 | chr9_25669082_- |  |  |  |  | 0 | 1 |
| HAM/TSP#1 | 1556 | chr9_2689271_- |  |  |  |  | 0 | 1 |
| HAM/TSP#1 | 1557 | chr9_2742263_- |  |  |  |  | 0 | 1 |
| HAM/TSP#1 | 1558 | chr9_28856279_- |  |  |  |  | 0 | 1 |
| HAM/TSP#1 | 1559 | chr9_303764_- |  |  |  |  | 0 | 3 |
| HAM/TSP#1 | 1560 | chr9_31450334_+ |  |  |  |  | 0 | 1 |
| HAM/TSP#1 | 1561 | chr9_33156550_+ |  |  |  |  | 0 | 9 |
| HAM/TSP#1 | 1562 | chr9_33165479_+ |  |  |  |  | 0 | 1 |
| HAM/TSP#1 | 1563 | chr9_33445827_- |  |  |  |  | 0 | 28 |
| HAM/TSP#1 | 1564 | chr9_33460652_- |  |  |  |  | 0 | 2 |
| HAM/TSP#1 | 1565 | chr9_3386696_+ |  |  |  |  | 0 | 2 |
| HAM/TSP#1 | 1566 | chr9_36959056_- |  |  |  |  | 0 | 1 |
| HAM/TSP#1 | 1567 | chr9_37734723_+ |  |  |  |  | 0 | 1 |
| HAM/TSP#1 | 1568 | chr9_38351969_- |  |  |  |  | 0 | 2 |
| HAM/TSP#1 | 1569 | chr9_39609141_+ |  |  |  |  | 0 | 1 |
| HAM/TSP#1 | 1570 | chr9_4097458_+ |  |  |  |  | 0 | 1 |
| HAM/TSP#1 | 1571 | chr9_43177040_- |  |  |  |  | 0 | 1 |
| HAM/TSP#1 | 1572 | chr9_43206396_- |  |  |  |  | 0 | 2 |
| HAM/TSP#1 | 1573 | chr9_512199_+ |  |  |  |  | 0 | 1 |
| HAM/TSP#1 | 1574 | chr9_64084706_+ |  |  |  |  | 0 | 1 |
| HAM/TSP#1 | 1575 | chr9_64095599_+ |  |  |  |  | 0 | 1 |
| HAM/TSP#1 | 1576 | chr9_69723117_- |  |  |  |  | 0 | 4 |
| HAM/TSP#1 | 1577 | chr9_69730749_- |  |  |  |  | 0 | 6 |
| HAM/TSP#1 | 1578 | chr9_70299472_+ |  |  |  |  | 0 | 2 |
| HAM/TSP#1 | 1579 | chr9_7036574_- |  |  |  |  | 0 | 1 |
| HAM/TSP#1 | 1580 | chr9_70368286_+ |  |  |  |  | 0 | 3 |
| HAM/TSP#1 | 1581 | chr9_71274187_- |  |  |  |  | 0 | 2 |
| HAM/TSP#1 | 1582 | chr9_72022994_+ |  |  |  |  | 0 | 1 |
| HAM/TSP#1 | 1583 | chr9_72053906_+ |  |  |  |  | 0 | 1 |
| HAM/TSP#1 | 1584 | chr9_72594408_- |  |  |  |  | 0 | 1 |
| HAM/TSP#1 | 1585 | chr9_77816779_+ |  |  |  |  | 0 | 1 |
| HAM/TSP#1 | 1586 | chr9_78188359_+ |  |  |  |  | 0 | 1 |
| HAM/TSP#1 | 1587 | chr9_7898175_- |  |  |  |  | 0 | 2 |
| HAM/TSP#1 | 1588 | chr9_79443788_+ |  |  |  |  | 0 | 1 |
| HAM/TSP#1 | 1589 | chr9_82546019_+ |  |  |  |  | 0 | 1 |
| HAM/TSP#1 | 1590 | chr9_82677564_+ |  |  |  |  | 0 | 7 |
| HAM/TSP#1 | 1591 | chr9_83351868_- |  |  |  |  | 0 | 1 |
| HAM/TSP#1 | 1592 | chr9_8432662_+ |  |  |  |  | 0 | 3 |
| HAM/TSP#1 | 1593 | chr9_84870903_- |  |  |  |  | 0 | 2 |
| HAM/TSP#1 | 1594 | chr9_86026347_- |  |  |  |  | 0 | 2 |
| HAM/TSP#1 | 1595 | chr9_86600798_+ |  |  |  |  | 0 | 1 |
| HAM/TSP#1 | 1596 | chr9_8703988_- |  |  |  |  | 0 | 2 |
| HAM/TSP#1 | 1597 | chr9_87378198_- |  |  |  |  | 0 | 1 |
| HAM/TSP#1 | 1598 | chr9_87599919_+ |  |  |  |  | 0 | 1 |
| HAM/TSP#1 | 1599 | chr9_87604912_+ |  |  |  |  | 0 | 1 |
| HAM/TSP#1 | 1600 | chr9_87783052_+ |  |  |  |  | 0 | 3 |
| HAM/TSP#1 | 1601 | chr9_89116651_+ |  |  |  |  | 0 | 1 |
| HAM/TSP#1 | 1602 | chr9_8967606_- |  |  |  |  | 0 | 1 |
| HAM/TSP#1 | 1603 | chr9_89721653_- |  |  |  |  | 0 | 1 |
| HAM/TSP#1 | 1604 | chr9_9080619_- |  |  |  |  | 0 | 5 |
| HAM/TSP#1 | 1605 | chr9_91643374_+ |  |  |  |  | 0 | 1 |
| HAM/TSP#1 | 1606 | chr9_91878547_+ |  |  |  |  | 0 | 10 |
| HAM/TSP#1 | 1607 | chr9_93308743_- |  |  |  |  | 0 | 6 |
| HAM/TSP#1 | 1608 | chr9_949788_- |  |  |  |  | 0 | 1 |
| HAM/TSP#1 | 1609 | chr9_95105937_+ |  |  |  |  | 0 | 1 |
| HAM/TSP#1 | 1610 | chr9_95412055_- |  |  |  |  | 0 | 2 |
| HAM/TSP#1 | 1611 | chr9_96403869_- |  |  |  |  | 0 | 1 |
| HAM/TSP#1 | 1612 | chr9_96421531_- |  |  |  |  | 0 | 1 |
| HAM/TSP#1 | 1613 | chr9_96879395_- |  |  |  |  | 0 | 1 |
| HAM/TSP#1 | 1614 | chr9_96955142_- |  |  |  |  | 0 | 1 |
| HAM/TSP#1 | 1615 | chr9_97378964_+ |  |  |  |  | 0 | 1 |
| HAM/TSP#1 | 1616 | chr9_97518130_- |  |  |  |  | 0 | 17 |
| HAM/TSP#1 | 1617 | chr9_99170819_+ |  |  |  |  | 0 | 1 |
| HAM/TSP#1 | 1618 | chr10_101002205_- |  |  |  |  | 0 | 1 |
| HAM/TSP#1 | 1619 | chr10_102639828_+ |  |  |  |  | 0 | 1 |
| HAM/TSP#1 | 1620 | chr10_103577972_+ |  |  |  |  | 0 | 1 |
| HAM/TSP#1 | 1621 | chr10_103579106_+ |  |  |  |  | 0 | 1 |
| HAM/TSP#1 | 1622 | chr10_108354303_- |  |  |  |  | 0 | 1 |
| HAM/TSP#1 | 1623 | chr10_108766459_- |  |  |  |  | 0 | 1 |
| HAM/TSP#1 | 1624 | chr10_10960056_+ |  |  |  |  | 0 | 3 |
| HAM/TSP#1 | 1625 | chr10_110503608_- |  |  |  |  | 0 | 2 |
| HAM/TSP#1 | 1626 | chr10_110504598_- |  |  |  |  | 0 | 1 |
| HAM/TSP#1 | 1627 | chr10_111069213_- |  |  |  |  | 0 | 2 |
| HAM/TSP#1 | 1628 | chr10_11143104_+ |  |  |  |  | 0 | 29 |
| HAM/TSP#1 | 1629 | chr10_112542396_- |  |  |  |  | 0 | 1 |
| HAM/TSP#1 | 1630 | chr10_113757319_- |  |  |  |  | 0 | 4 |
| HAM/TSP#1 | 1631 | chr10_114657921_+ |  |  |  |  | 0 | 1 |
| HAM/TSP#1 | 1632 | chr10_119146936_- |  |  |  |  | 0 | 1 |
| HAM/TSP#1 | 1633 | chr10_12039261_- |  |  |  |  | 0 | 1 |
| HAM/TSP#1 | 1634 | chr10_121195186_- |  |  |  |  | 0 | 1 |
| HAM/TSP#1 | 1635 | chr10_121449289_+ |  |  |  |  | 0 | 2 |
| HAM/TSP#1 | 1636 | chr10_121457872_+ |  |  |  |  | 0 | 1 |
| HAM/TSP#1 | 1637 | chr10_122228594_- |  |  |  |  | 0 | 1 |
| HAM/TSP#1 | 1638 | chr10_122404097_+ |  |  |  |  | 0 | 1 |
| HAM/TSP#1 | 1639 | chr10_12275885_+ |  |  |  |  | 0 | 1 |
| HAM/TSP#1 | 1640 | chr10_124904682_- |  |  |  |  | 0 | 1 |
| HAM/TSP#1 | 1641 | chr10_125209855_- |  |  |  |  | 0 | 1 |
| HAM/TSP#1 | 1642 | chr10_125585565_+ |  |  |  |  | 0 | 1 |
| HAM/TSP#1 | 1643 | chr10_126689575_- |  |  |  |  | 0 | 1 |
| HAM/TSP#1 | 1644 | chr10_12901642_- |  |  |  |  | 0 | 19 |
| HAM/TSP#1 | 1645 | chr10_129440741_- |  |  |  |  | 0 | 4 |
| HAM/TSP#1 | 1646 | chr10_129556895_+ |  |  |  |  | 0 | 1 |
| HAM/TSP#1 | 1647 | chr10_129581704_- |  |  |  |  | 0 | 1 |
| HAM/TSP#1 | 1648 | chr10_130158649_- |  |  |  |  | 0 | 1 |
| HAM/TSP#1 | 1649 | chr10_132439377_- |  |  |  |  | 0 | 1 |
| HAM/TSP#1 | 1650 | chr10_132809392_+ |  |  |  |  | 0 | 1 |
| HAM/TSP#1 | 1651 | chr10_132962221_- |  |  |  |  | 0 | 1 |
| HAM/TSP#1 | 1652 | chr10_133370826_+ |  |  |  |  | 0 | 1 |
| HAM/TSP#1 | 1653 | chr10_13658875_- |  |  |  |  | 0 | 7 |
| HAM/TSP#1 | 1654 | chr10_13666819_- |  |  |  |  | 0 | 1 |
| HAM/TSP#1 | 1655 | chr10_14603316_+ |  |  |  |  | 0 | 1 |
| HAM/TSP#1 | 1656 | chr10_14695395_+ |  |  |  |  | 0 | 4 |
| HAM/TSP#1 | 1657 | chr10_16719442_- |  |  |  |  | 0 | 1 |
| HAM/TSP#1 | 1658 | chr10_16914653_- |  |  |  |  | 0 | 6 |
| HAM/TSP#1 | 1659 | chr10_17652580_+ |  |  |  |  | 0 | 2 |
| HAM/TSP#1 | 1660 | chr10_18650446_- |  |  |  |  | 0 | 1 |
| HAM/TSP#1 | 1661 | chr10_1961761_- |  |  |  |  | 0 | 1 |
| HAM/TSP#1 | 1662 | chr10_1968866_+ |  |  |  |  | 0 | 1 |
| HAM/TSP#1 | 1663 | chr10_2002363_- |  |  |  |  | 0 | 5 |
| HAM/TSP#1 | 1664 | chr10_20668072_+ |  |  |  |  | 0 | 1 |
| HAM/TSP#1 | 1665 | chr10_23533468_+ |  |  |  |  | 0 | 1 |
| HAM/TSP#1 | 1666 | chr10_24310713_+ |  |  |  |  | 0 | 1 |
| HAM/TSP#1 | 1667 | chr10_2903619_+ |  |  |  |  | 0 | 1 |
| HAM/TSP#1 | 1668 | chr10_29459100_+ |  |  |  |  | 0 | 11 |
| HAM/TSP#1 | 1669 | chr10_32133748_- |  |  |  |  | 0 | 1 |
| HAM/TSP#1 | 1670 | chr10_32704272_+ |  |  |  |  | 0 | 1 |
| HAM/TSP#1 | 1671 | chr10_33510086_+ |  |  |  |  | 0 | 1 |
| HAM/TSP#1 | 1672 | chr10_36354583_+ |  |  |  |  | 0 | 1 |
| HAM/TSP#1 | 1673 | chr10_36786752_- |  |  |  |  | 0 | 1 |
| HAM/TSP#1 | 1674 | chr10_37488788_+ |  |  |  |  | 0 | 1 |
| HAM/TSP#1 | 1675 | chr10_37730137_+ |  |  |  |  | 0 | 1 |
| HAM/TSP#1 | 1676 | chr10_3806541_- |  |  |  |  | 0 | 8 |
| HAM/TSP#1 | 1677 | chr10_38396406_+ |  |  |  |  | 0 | 1 |
| HAM/TSP#1 | 1678 | chr10_41879540_- |  |  |  |  | 0 | 1 |
| HAM/TSP#1 | 1679 | chr10_42551967_- |  |  |  |  | 0 | 1 |
| HAM/TSP#1 | 1680 | chr10_43193959_- |  |  |  |  | 0 | 48 |
| HAM/TSP#1 | 1681 | chr10_43407874_+ |  |  |  |  | 0 | 1 |
| HAM/TSP#1 | 1682 | chr10_43441966_+ |  |  |  |  | 0 | 4 |
| HAM/TSP#1 | 1683 | chr10_43816655_+ |  |  |  |  | 0 | 1 |
| HAM/TSP#1 | 1684 | chr10_43845215_+ |  |  |  |  | 0 | 1 |
| HAM/TSP#1 | 1685 | chr10_44348840_- |  |  |  |  | 0 | 1 |
| HAM/TSP#1 | 1686 | chr10_45612653_+ |  |  |  |  | 0 | 3 |
| HAM/TSP#1 | 1687 | chr10_46555652_- |  |  |  |  | 0 | 27 |
| HAM/TSP#1 | 1688 | chr10_4687468_+ |  |  |  |  | 0 | 1 |
| HAM/TSP#1 | 1689 | chr10_47366289_+ |  |  |  |  | 0 | 1 |
| HAM/TSP#1 | 1690 | chr10_48266926_- |  |  |  |  | 0 | 1 |
| HAM/TSP#1 | 1691 | chr10_48659142_+ |  |  |  |  | 0 | 1 |
| HAM/TSP#1 | 1692 | chr10_49395136_- |  |  |  |  | 0 | 1 |
| HAM/TSP#1 | 1693 | chr10_50703746_+ |  |  |  |  | 0 | 1 |
| HAM/TSP#1 | 1694 | chr10_52591616_+ |  |  |  |  | 0 | 1 |
| HAM/TSP#1 | 1695 | chr10_53691945_+ |  |  |  |  | 0 | 4 |
| HAM/TSP#1 | 1696 | chr10_53975457_- |  |  |  |  | 0 | 1 |
| HAM/TSP#1 | 1697 | chr10_55778774_- |  |  |  |  | 0 | 1 |
| HAM/TSP#1 | 1698 | chr10_55778788_- |  |  |  |  | 0 | 2 |
| HAM/TSP#1 | 1699 | chr10_56759362_- |  |  |  |  | 0 | 3 |
| HAM/TSP#1 | 1700 | chr10_58398251_+ |  |  |  |  | 0 | 5 |
| HAM/TSP#1 | 1701 | chr10_58640572_+ |  |  |  |  | 0 | 1 |
| HAM/TSP#1 | 1702 | chr10_59900424_+ |  |  |  |  | 0 | 1 |
| HAM/TSP#1 | 1703 | chr10_62664627_- |  |  |  |  | 0 | 1 |
| HAM/TSP#1 | 1704 | chr10_63573857_- |  |  |  |  | 0 | 4 |
| HAM/TSP#1 | 1705 | chr10_64021775_- |  |  |  |  | 0 | 1 |
| HAM/TSP#1 | 1706 | chr10_66030467_- |  |  |  |  | 0 | 3 |
| HAM/TSP#1 | 1707 | chr10_66127551_+ |  |  |  |  | 0 | 2 |
| HAM/TSP#1 | 1708 | chr10_68507877_- |  |  |  |  | 0 | 2 |
| HAM/TSP#1 | 1709 | chr10_69768983_+ |  |  |  |  | 0 | 1 |
| HAM/TSP#1 | 1710 | chr10_69843131_- |  |  |  |  | 0 | 1 |
| HAM/TSP#1 | 1711 | chr10_700313_- |  |  |  |  | 0 | 1 |
| HAM/TSP#1 | 1712 | chr10_717349_+ |  |  |  |  | 0 | 23 |
| HAM/TSP#1 | 1713 | chr10_723774_- |  |  |  |  | 0 | 1 |
| HAM/TSP#1 | 1714 | chr10_73545222_- |  |  |  |  | 0 | 1 |
| HAM/TSP#1 | 1715 | chr10_73607174_- |  |  |  |  | 0 | 1 |
| HAM/TSP#1 | 1716 | chr10_7363671_- |  |  |  |  | 0 | 3 |
| HAM/TSP#1 | 1717 | chr10_73776551_+ |  |  |  |  | 0 | 11 |
| HAM/TSP#1 | 1718 | chr10_74184356_- |  |  |  |  | 0 | 3 |
| HAM/TSP#1 | 1719 | chr10_74931974_+ |  |  |  |  | 0 | 1 |
| HAM/TSP#1 | 1720 | chr10_77038762_+ |  |  |  |  | 0 | 1 |
| HAM/TSP#1 | 1721 | chr10_78056310_+ |  |  |  |  | 0 | 1 |
| HAM/TSP#1 | 1722 | chr10_79288577_- |  |  |  |  | 0 | 1 |
| HAM/TSP#1 | 1723 | chr10_79547553_+ |  |  |  |  | 0 | 1 |
| HAM/TSP#1 | 1724 | chr10_79931173_+ |  |  |  |  | 0 | 1 |
| HAM/TSP#1 | 1725 | chr10_81683115_- |  |  |  |  | 0 | 1 |
| HAM/TSP#1 | 1726 | chr10_8186054_- |  |  |  |  | 0 | 2 |
| HAM/TSP#1 | 1727 | chr10_8377808_+ |  |  |  |  | 0 | 1 |
| HAM/TSP#1 | 1728 | chr10_84158303_+ |  |  |  |  | 0 | 26 |
| HAM/TSP#1 | 1729 | chr10_88075430_- |  |  |  |  | 0 | 1 |
| HAM/TSP#1 | 1730 | chr10_88193517_+ |  |  |  |  | 0 | 1 |
| HAM/TSP#1 | 1731 | chr10_88289883_- |  |  |  |  | 0 | 1 |
| HAM/TSP#1 | 1732 | chr10_89115939_+ |  |  |  |  | 2 | 8 |
| HAM/TSP#1 | 1733 | chr10_89129800_- |  |  |  |  | 0 | 1 |
| HAM/TSP#1 | 1734 | chr10_89275883_+ |  |  |  |  | 0 | 1 |
| HAM/TSP#1 | 1735 | chr10_89351971_- |  |  |  |  | 0 | 1 |
| HAM/TSP#1 | 1736 | chr10_92245671_- |  |  |  |  | 0 | 6 |
| HAM/TSP#1 | 1737 | chr10_93410786_- |  |  |  |  | 0 | 1 |
| HAM/TSP#1 | 1738 | chr10_95761381_+ |  |  |  |  | 0 | 2 |
| HAM/TSP#1 | 1739 | chr10_96230891_+ |  |  |  |  | 0 | 1 |
| HAM/TSP#1 | 1740 | chr10_97180069_+ |  |  |  |  | 0 | 1 |
| HAM/TSP#1 | 1741 | chr10_98152081_- |  |  |  |  | 0 | 1 |
| HAM/TSP#1 | 1742 | chr10_99629759_+ |  |  |  |  | 0 | 4 |
| HAM/TSP#1 | 1743 | chr10_99857846_- |  |  |  |  | 0 | 4 |
| HAM/TSP#1 | 1744 | chr11_100753498_- |  |  |  |  | 0 | 1 |
| HAM/TSP#1 | 1745 | chr11_100979868_- |  |  |  |  | 0 | 1 |
| HAM/TSP#1 | 1746 | chr11_101133317_- |  |  |  |  | 0 | 1 |
| HAM/TSP#1 | 1747 | chr11_101887691_- |  |  |  |  | 0 | 1 |
| HAM/TSP#1 | 1748 | chr11_102398944_+ |  |  |  |  | 0 | 1 |
| HAM/TSP#1 | 1749 | chr11_102808012_- |  |  |  |  | 0 | 1 |
| HAM/TSP#1 | 1750 | chr11_1032340_- |  |  |  |  | 0 | 1 |
| HAM/TSP#1 | 1751 | chr11_10633406_+ |  |  |  |  | 0 | 1 |
| HAM/TSP#1 | 1752 | chr11_106689858_- |  |  |  |  | 0 | 1 |
| HAM/TSP#1 | 1753 | chr11_106938980_- |  |  |  |  | 0 | 3 |
| HAM/TSP#1 | 1754 | chr11_111302234_+ |  |  |  |  | 0 | 5 |
| HAM/TSP#1 | 1755 | chr11_111561048_+ |  |  |  |  | 0 | 3 |
| HAM/TSP#1 | 1756 | chr11_112312346_- |  |  |  |  | 0 | 1 |
| HAM/TSP#1 | 1757 | chr11_11282609_+ |  |  |  |  | 0 | 1 |
| HAM/TSP#1 | 1758 | chr11_113630618_+ |  |  |  |  | 0 | 1 |
| HAM/TSP#1 | 1759 | chr11_114419851_- |  |  |  |  | 0 | 1 |
| HAM/TSP#1 | 1760 | chr11_114523953_+ |  |  |  |  | 0 | 9 |
| HAM/TSP#1 | 1761 | chr11_114832218_- |  |  |  |  | 0 | 1 |
| HAM/TSP#1 | 1762 | chr11_115809742_- |  |  |  |  | 0 | 1 |
| HAM/TSP#1 | 1763 | chr11_116167524_+ |  |  |  |  | 0 | 1 |
| HAM/TSP#1 | 1764 | chr11_116254630_+ |  |  |  |  | 0 | 1 |
| HAM/TSP#1 | 1765 | chr11_116296741_+ |  |  |  |  | 0 | 1 |
| HAM/TSP#1 | 1766 | chr11_11633172_+ |  |  |  |  | 0 | 1 |
| HAM/TSP#1 | 1767 | chr11_116752429_- |  |  |  |  | 0 | 1 |
| HAM/TSP#1 | 1768 | chr11_116867134_+ |  |  |  |  | 0 | 2 |
| HAM/TSP#1 | 1769 | chr11_117258483_- |  |  |  |  | 0 | 3 |
| HAM/TSP#1 | 1770 | chr11_117371045_- |  |  |  |  | 0 | 4 |
| HAM/TSP#1 | 1771 | chr11_117392562_+ |  |  |  |  | 0 | 1 |
| HAM/TSP#1 | 1772 | chr11_118163322_+ |  |  |  |  | 0 | 2 |
| HAM/TSP#1 | 1773 | chr11_118225855_- |  |  |  |  | 0 | 2 |
| HAM/TSP#1 | 1774 | chr11_118342330_- |  |  |  |  | 0 | 1 |
| HAM/TSP#1 | 1775 | chr11_118438687_+ |  |  |  |  | 0 | 1 |
| HAM/TSP#1 | 1776 | chr11_119382260_- |  |  |  |  | 0 | 3 |
| HAM/TSP#1 | 1777 | chr11_11973871_+ |  |  |  |  | 0 | 1 |
| HAM/TSP#1 | 1778 | chr11_12119987_- |  |  |  |  | 0 | 1 |
| HAM/TSP#1 | 1779 | chr11_122355501_- |  |  |  |  | 0 | 3 |
| HAM/TSP#1 | 1780 | chr11_122638710_+ |  |  |  |  | 0 | 2 |
| HAM/TSP#1 | 1781 | chr11_122679560_- |  |  |  |  | 0 | 5 |
| HAM/TSP#1 | 1782 | chr11_123383529_- |  |  |  |  | 0 | 1 |
| HAM/TSP#1 | 1783 | chr11_125576267_- |  |  |  |  | 0 | 3 |
| HAM/TSP#1 | 1784 | chr11_126451253_+ |  |  |  |  | 0 | 1 |
| HAM/TSP#1 | 1785 | chr11_126589638_+ |  |  |  |  | 0 | 1 |
| HAM/TSP#1 | 1786 | chr11_128713081_+ |  |  |  |  | 0 | 1 |
| HAM/TSP#1 | 1787 | chr11_128859808_- |  |  |  |  | 0 | 1 |
| HAM/TSP#1 | 1788 | chr11_128930262_+ |  |  |  |  | 0 | 1 |
| HAM/TSP#1 | 1789 | chr11_129899100_- |  |  |  |  | 0 | 2 |
| HAM/TSP#1 | 1790 | chr11_130532030_+ |  |  |  |  | 0 | 2 |
| HAM/TSP#1 | 1791 | chr11_132960826_- |  |  |  |  | 0 | 1 |
| HAM/TSP#1 | 1792 | chr11_133108256_- |  |  |  |  | 0 | 7 |
| HAM/TSP#1 | 1793 | chr11_133914801_- |  |  |  |  | 0 | 1 |
| HAM/TSP#1 | 1794 | chr11_134176466_+ |  |  |  |  | 0 | 1 |
| HAM/TSP#1 | 1795 | chr11_1391163_- |  |  |  |  | 0 | 1 |
| HAM/TSP#1 | 1796 | chr11_16582547_- |  |  |  |  | 0 | 1 |
| HAM/TSP#1 | 1797 | chr11_16799923_- |  |  |  |  | 0 | 1 |
| HAM/TSP#1 | 1798 | chr11_17701552_+ |  |  |  |  | 0 | 1 |
| HAM/TSP#1 | 1799 | chr11_1824750_+ |  |  |  |  | 0 | 1 |
| HAM/TSP#1 | 1800 | chr11_18413784_- |  |  |  |  | 0 | 1 |
| HAM/TSP#1 | 1801 | chr11_19788474_+ |  |  |  |  | 0 | 1 |
| HAM/TSP#1 | 1802 | chr11_20109733_- |  |  |  |  | 0 | 1 |
| HAM/TSP#1 | 1803 | chr11_20256470_- |  |  |  |  | 0 | 4 |
| HAM/TSP#1 | 1804 | chr11_2036173_+ |  |  |  |  | 0 | 3 |
| HAM/TSP#1 | 1805 | chr11_21079927_- |  |  |  |  | 0 | 2 |
| HAM/TSP#1 | 1806 | chr11_21883596_+ |  |  |  |  | 0 | 4 |
| HAM/TSP#1 | 1807 | chr11_2205232_- |  |  |  |  | 0 | 1 |
| HAM/TSP#1 | 1808 | chr11_23016700_+ |  |  |  |  | 0 | 1 |
| HAM/TSP#1 | 1809 | chr11_2423695_- |  |  |  |  | 0 | 1 |
| HAM/TSP#1 | 1810 | chr11_25585237_- |  |  |  |  | 0 | 5 |
| HAM/TSP#1 | 1811 | chr11_26002087_- |  |  |  |  | 0 | 6 |
| HAM/TSP#1 | 1812 | chr11_2628346_+ |  |  |  |  | 0 | 1 |
| HAM/TSP#1 | 1813 | chr11_28245678_+ |  |  |  |  | 0 | 2 |
| HAM/TSP#1 | 1814 | chr11_2844349_- |  |  |  |  | 0 | 1 |
| HAM/TSP#1 | 1815 | chr11_29065024_+ |  |  |  |  | 0 | 2 |
| HAM/TSP#1 | 1816 | chr11_29974922_- |  |  |  |  | 0 | 1 |
| HAM/TSP#1 | 1817 | chr11_3023488_- |  |  |  |  | 0 | 18 |
| HAM/TSP#1 | 1818 | chr11_30284055_+ |  |  |  |  | 0 | 2 |
| HAM/TSP#1 | 1819 | chr11_30992749_- |  |  |  |  | 0 | 2 |
| HAM/TSP#1 | 1820 | chr11_3149449_- |  |  |  |  | 0 | 1 |
| HAM/TSP#1 | 1821 | chr11_32366707_+ |  |  |  |  | 0 | 3 |
| HAM/TSP#1 | 1822 | chr11_32494177_- |  |  |  |  | 0 | 1 |
| HAM/TSP#1 | 1823 | chr11_34208777_- |  |  |  |  | 0 | 1 |
| HAM/TSP#1 | 1824 | chr11_34242051_+ |  |  |  |  | 0 | 1 |
| HAM/TSP#1 | 1825 | chr11_34879257_- |  |  |  |  | 0 | 1 |
| HAM/TSP#1 | 1826 | chr11_35876888_+ |  |  |  |  | 0 | 1 |
| HAM/TSP#1 | 1827 | chr11_35945422_- |  |  |  |  | 0 | 1 |
| HAM/TSP#1 | 1828 | chr11_35984785_- |  |  |  |  | 0 | 1 |
| HAM/TSP#1 | 1829 | chr11_36273281_- |  |  |  |  | 0 | 1 |
| HAM/TSP#1 | 1830 | chr11_36992832_- |  |  |  |  | 0 | 3 |
| HAM/TSP#1 | 1831 | chr11_37211953_- |  |  |  |  | 0 | 4 |
| HAM/TSP#1 | 1832 | chr11_38769442_+ |  |  |  |  | 0 | 1 |
| HAM/TSP#1 | 1833 | chr11_38895052_- |  |  |  |  | 0 | 3 |
| HAM/TSP#1 | 1834 | chr11_40664965_+ |  |  |  |  | 0 | 1 |
| HAM/TSP#1 | 1835 | chr11_41914451_- |  |  |  |  | 0 | 1 |
| HAM/TSP#1 | 1836 | chr11_42031544_- |  |  |  |  | 0 | 3 |
| HAM/TSP#1 | 1837 | chr11_42821953_- |  |  |  |  | 0 | 3 |
| HAM/TSP#1 | 1838 | chr11_43223947_- |  |  |  |  | 0 | 1 |
| HAM/TSP#1 | 1839 | chr11_43293295_- |  |  |  |  | 0 | 5 |
| HAM/TSP#1 | 1840 | chr11_43413199_- |  |  |  |  | 0 | 3 |
| HAM/TSP#1 | 1841 | chr11_4351924_+ |  |  |  |  | 0 | 1 |
| HAM/TSP#1 | 1842 | chr11_44345541_+ |  |  |  |  | 0 | 1 |
| HAM/TSP#1 | 1843 | chr11_44914924_+ |  |  |  |  | 0 | 1 |
| HAM/TSP#1 | 1844 | chr11_45437884_- |  |  |  |  | 0 | 1 |
| HAM/TSP#1 | 1845 | chr11_45805659_- |  |  |  |  | 0 | 3 |
| HAM/TSP#1 | 1846 | chr11_4613286_- |  |  |  |  | 0 | 1 |
| HAM/TSP#1 | 1847 | chr11_46372321_- |  |  |  |  | 0 | 2 |
| HAM/TSP#1 | 1848 | chr11_47148467_- |  |  |  |  | 0 | 12 |
| HAM/TSP#1 | 1849 | chr11_48344216_- |  |  |  |  | 0 | 1 |
| HAM/TSP#1 | 1850 | chr11_49791219_- |  |  |  |  | 0 | 1 |
| HAM/TSP#1 | 1851 | chr11_50757334_- |  |  |  |  | 0 | 1 |
| HAM/TSP#1 | 1852 | chr11_57481792_+ |  |  |  |  | 0 | 1 |
| HAM/TSP#1 | 1853 | chr11_57525636_+ |  |  |  |  | 0 | 1 |
| HAM/TSP#1 | 1854 | chr11_58276775_+ |  |  |  |  | 0 | 1 |
| HAM/TSP#1 | 1855 | chr11_59105376_- |  |  |  |  | 0 | 9 |
| HAM/TSP#1 | 1856 | chr11_60311353_+ |  |  |  |  | 0 | 2 |
| HAM/TSP#1 | 1857 | chr11_60928237_+ |  |  |  |  | 0 | 1 |
| HAM/TSP#1 | 1858 | chr11_61568930_- |  |  |  |  | 0 | 1 |
| HAM/TSP#1 | 1859 | chr11_62099858_- |  |  |  |  | 0 | 1 |
| HAM/TSP#1 | 1860 | chr11_6225804_- |  |  |  |  | 0 | 1 |
| HAM/TSP#1 | 1861 | chr11_62670481_- |  |  |  |  | 0 | 1 |
| HAM/TSP#1 | 1862 | chr11_6340878_+ |  |  |  |  | 0 | 1 |
| HAM/TSP#1 | 1863 | chr11_6346106_+ |  |  |  |  | 0 | 1 |
| HAM/TSP#1 | 1864 | chr11_63904865_- |  |  |  |  | 0 | 1 |
| HAM/TSP#1 | 1865 | chr11_6416563_- |  |  |  |  | 0 | 1 |
| HAM/TSP#1 | 1866 | chr11_64691722_- |  |  |  |  | 0 | 1 |
| HAM/TSP#1 | 1867 | chr11_64895434_+ |  |  |  |  | 0 | 1 |
| HAM/TSP#1 | 1868 | chr11_65030983_- |  |  |  |  | 0 | 1 |
| HAM/TSP#1 | 1869 | chr11_65109362_+ |  |  |  |  | 0 | 1 |
| HAM/TSP#1 | 1870 | chr11_65449274_+ |  |  |  |  | 0 | 1 |
| HAM/TSP#1 | 1871 | chr11_66359656_+ |  |  |  |  | 0 | 1 |
| HAM/TSP#1 | 1872 | chr11_66619018_+ |  |  |  |  | 0 | 3 |
| HAM/TSP#1 | 1873 | chr11_674704_+ |  |  |  |  | 0 | 1 |
| HAM/TSP#1 | 1874 | chr11_68006755_- |  |  |  |  | 0 | 4 |
| HAM/TSP#1 | 1875 | chr11_68515470_+ |  |  |  |  | 0 | 1 |
| HAM/TSP#1 | 1876 | chr11_68807719_- |  |  |  |  | 0 | 1 |
| HAM/TSP#1 | 1877 | chr11_69126600_+ |  |  |  |  | 0 | 1 |
| HAM/TSP#1 | 1878 | chr11_69466909_- |  |  |  |  | 0 | 1 |
| HAM/TSP#1 | 1879 | chr11_71489742_+ |  |  |  |  | 0 | 1 |
| HAM/TSP#1 | 1880 | chr11_72823906_+ |  |  |  |  | 0 | 1 |
| HAM/TSP#1 | 1881 | chr11_73758697_- |  |  |  |  | 0 | 1 |
| HAM/TSP#1 | 1882 | chr11_74138399_+ |  |  |  |  | 0 | 5 |
| HAM/TSP#1 | 1883 | chr11_75174945_- |  |  |  |  | 0 | 1 |
| HAM/TSP#1 | 1884 | chr11_75265357_- |  |  |  |  | 0 | 1 |
| HAM/TSP#1 | 1885 | chr11_75980120_+ |  |  |  |  | 0 | 8 |
| HAM/TSP#1 | 1886 | chr11_76601571_- |  |  |  |  | 0 | 1 |
| HAM/TSP#1 | 1887 | chr11_77732395_- |  |  |  |  | 0 | 1 |
| HAM/TSP#1 | 1888 | chr11_78772857_- |  |  |  |  | 1 | 11 |
| HAM/TSP#1 | 1889 | chr11_78809435_+ |  |  |  |  | 0 | 5 |
| HAM/TSP#1 | 1890 | chr11_78977669_- |  |  |  |  | 0 | 1 |
| HAM/TSP#1 | 1891 | chr11_79512167_- |  |  |  |  | 0 | 1 |
| HAM/TSP#1 | 1892 | chr11_79545611_- |  |  |  |  | 0 | 1 |
| HAM/TSP#1 | 1893 | chr11_8004559_+ |  |  |  |  | 0 | 12 |
| HAM/TSP#1 | 1894 | chr11_80251191_+ |  |  |  |  | 0 | 1 |
| HAM/TSP#1 | 1895 | chr11_80413226_+ |  |  |  |  | 0 | 1 |
| HAM/TSP#1 | 1896 | chr11_83096001_- |  |  |  |  | 0 | 1 |
| HAM/TSP#1 | 1897 | chr11_8359022_- |  |  |  |  | 0 | 1 |
| HAM/TSP#1 | 1898 | chr11_83853202_- |  |  |  |  | 0 | 1 |
| HAM/TSP#1 | 1899 | chr11_8426366_- |  |  |  |  | 0 | 1 |
| HAM/TSP#1 | 1900 | chr11_85737533_- |  |  |  |  | 0 | 1 |
| HAM/TSP#1 | 1901 | chr11_85766373_- |  |  |  |  | 0 | 1 |
| HAM/TSP#1 | 1902 | chr11_88110112_+ |  |  |  |  | 0 | 3 |
| HAM/TSP#1 | 1903 | chr11_8874385_+ |  |  |  |  | 0 | 2 |
| HAM/TSP#1 | 1904 | chr11_88967788_+ |  |  |  |  | 0 | 1 |
| HAM/TSP#1 | 1905 | chr11_92375082_- |  |  |  |  | 0 | 1 |
| HAM/TSP#1 | 1906 | chr11_92396234_- |  |  |  |  | 0 | 1 |
| HAM/TSP#1 | 1907 | chr11_92763066_+ |  |  |  |  | 0 | 1 |
| HAM/TSP#1 | 1908 | chr11_936499_- |  |  |  |  | 0 | 1 |
| HAM/TSP#1 | 1909 | chr11_9394064_+ |  |  |  |  | 0 | 1 |
| HAM/TSP#1 | 1910 | chr11_94088955_+ |  |  |  |  | 0 | 1 |
| HAM/TSP#1 | 1911 | chr11_94358358_- |  |  |  |  | 0 | 1 |
| HAM/TSP#1 | 1912 | chr11_94988768_+ |  |  |  |  | 0 | 1 |
| HAM/TSP#1 | 1913 | chr11_95394349_- |  |  |  |  | 0 | 2 |
| HAM/TSP#1 | 1914 | chr11_97814923_+ |  |  |  |  | 0 | 7 |
| HAM/TSP#1 | 1915 | chr11_97866251_+ |  |  |  |  | 0 | 1 |
| HAM/TSP#1 | 1916 | chr12_100010927_- |  |  |  |  | 0 | 1 |
| HAM/TSP#1 | 1917 | chr12_100637075_- |  |  |  |  | 0 | 1 |
| HAM/TSP#1 | 1918 | chr12_101228732_+ |  |  |  |  | 0 | 1 |
| HAM/TSP#1 | 1919 | chr12_101454396_+ |  |  |  |  | 0 | 1 |
| HAM/TSP#1 | 1920 | chr12_102263984_+ |  |  |  |  | 0 | 2 |
| HAM/TSP#1 | 1921 | chr12_102771884_- |  |  |  |  | 0 | 1 |
| HAM/TSP#1 | 1922 | chr12_10332973_+ |  |  |  |  | 0 | 1 |
| HAM/TSP#1 | 1923 | chr12_103510339_- |  |  |  |  | 0 | 2 |
| HAM/TSP#1 | 1924 | chr12_103525350_+ |  |  |  |  | 0 | 1 |
| HAM/TSP#1 | 1925 | chr12_105385818_- |  |  |  |  | 0 | 1 |
| HAM/TSP#1 | 1926 | chr12_106759857_+ |  |  |  |  | 0 | 3 |
| HAM/TSP#1 | 1927 | chr12_107725630_- |  |  |  |  | 0 | 1 |
| HAM/TSP#1 | 1928 | chr12_108603474_+ |  |  |  |  | 0 | 1 |
| HAM/TSP#1 | 1929 | chr12_108784241_+ |  |  |  |  | 0 | 1 |
| HAM/TSP#1 | 1930 | chr12_109102748_+ |  |  |  |  | 0 | 3 |
| HAM/TSP#1 | 1931 | chr12_109840866_- |  |  |  |  | 0 | 17 |
| HAM/TSP#1 | 1932 | chr12_111226767_- |  |  |  |  | 0 | 1 |
| HAM/TSP#1 | 1933 | chr12_111363488_+ |  |  |  |  | 0 | 1 |
| HAM/TSP#1 | 1934 | chr12_111695131_- |  |  |  |  | 0 | 1 |
| HAM/TSP#1 | 1935 | chr12_111736799_+ |  |  |  |  | 0 | 2 |
| HAM/TSP#1 | 1936 | chr12_112857934_+ |  |  |  |  | 0 | 1 |
| HAM/TSP#1 | 1937 | chr12_113021476_+ |  |  |  |  | 0 | 1 |
| HAM/TSP#1 | 1938 | chr12_113854799_+ |  |  |  |  | 0 | 1 |
| HAM/TSP#1 | 1939 | chr12_114335920_- |  |  |  |  | 0 | 1 |
| HAM/TSP#1 | 1940 | chr12_115272407_+ |  |  |  |  | 0 | 1 |
| HAM/TSP#1 | 1941 | chr12_115440543_- |  |  |  |  | 0 | 1 |
| HAM/TSP#1 | 1942 | chr12_117454244_+ |  |  |  |  | 0 | 1 |
| HAM/TSP#1 | 1943 | chr12_11835135_- |  |  |  |  | 0 | 1 |
| HAM/TSP#1 | 1944 | chr12_120591643_+ |  |  |  |  | 0 | 1 |
| HAM/TSP#1 | 1945 | chr12_122192743_+ |  |  |  |  | 0 | 1 |
| HAM/TSP#1 | 1946 | chr12_122737679_- |  |  |  |  | 0 | 2 |
| HAM/TSP#1 | 1947 | chr12_122949250_- |  |  |  |  | 0 | 1 |
| HAM/TSP#1 | 1948 | chr12_125158487_+ |  |  |  |  | 0 | 1 |
| HAM/TSP#1 | 1949 | chr12_128650940_+ |  |  |  |  | 0 | 1 |
| HAM/TSP#1 | 1950 | chr12_130064388_+ |  |  |  |  | 0 | 1 |
| HAM/TSP#1 | 1951 | chr12_132083647_+ |  |  |  |  | 0 | 12 |
| HAM/TSP#1 | 1952 | chr12_132325348_+ |  |  |  |  | 0 | 1 |
| HAM/TSP#1 | 1953 | chr12_132525459_- |  |  |  |  | 0 | 1 |
| HAM/TSP#1 | 1954 | chr12_132535797_- |  |  |  |  | 0 | 1 |
| HAM/TSP#1 | 1955 | chr12_132787193_- |  |  |  |  | 0 | 5 |
| HAM/TSP#1 | 1956 | chr12_132954239_- |  |  |  |  | 0 | 2 |
| HAM/TSP#1 | 1957 | chr12_15892463_- |  |  |  |  | 0 | 1 |
| HAM/TSP#1 | 1958 | chr12_16871976_- |  |  |  |  | 0 | 4 |
| HAM/TSP#1 | 1959 | chr12_17136843_- |  |  |  |  | 0 | 1 |
| HAM/TSP#1 | 1960 | chr12_19127122_+ |  |  |  |  | 0 | 2 |
| HAM/TSP#1 | 1961 | chr12_2029626_+ |  |  |  |  | 0 | 1 |
| HAM/TSP#1 | 1962 | chr12_21018037_- |  |  |  |  | 0 | 1 |
| HAM/TSP#1 | 1963 | chr12_21719029_- |  |  |  |  | 0 | 4 |
| HAM/TSP#1 | 1964 | chr12_24771167_+ |  |  |  |  | 0 | 7 |
| HAM/TSP#1 | 1965 | chr12_24953977_+ |  |  |  |  | 0 | 1 |
| HAM/TSP#1 | 1966 | chr12_2707175_- |  |  |  |  | 0 | 1 |
| HAM/TSP#1 | 1967 | chr12_28883327_+ |  |  |  |  | 0 | 1 |
| HAM/TSP#1 | 1968 | chr12_28948024_+ |  |  |  |  | 0 | 1 |
| HAM/TSP#1 | 1969 | chr12_29231901_- |  |  |  |  | 0 | 5 |
| HAM/TSP#1 | 1970 | chr12_29785033_- |  |  |  |  | 0 | 30 |
| HAM/TSP#1 | 1971 | chr12_30675838_- |  |  |  |  | 0 | 12 |
| HAM/TSP#1 | 1972 | chr12_31816783_- |  |  |  |  | 0 | 1 |
| HAM/TSP#1 | 1973 | chr12_32037330_- |  |  |  |  | 0 | 1 |
| HAM/TSP#1 | 1974 | chr12_3266852_- |  |  |  |  | 0 | 1 |
| HAM/TSP#1 | 1975 | chr12_3322094_+ |  |  |  |  | 0 | 1 |
| HAM/TSP#1 | 1976 | chr12_33974747_+ |  |  |  |  | 0 | 1 |
| HAM/TSP#1 | 1977 | chr12_34281503_- |  |  |  |  | 0 | 1 |
| HAM/TSP#1 | 1978 | chr12_34332404_+ |  |  |  |  | 0 | 1 |
| HAM/TSP#1 | 1979 | chr12_3695744_- |  |  |  |  | 0 | 1 |
| HAM/TSP#1 | 1980 | chr12_37335712_- |  |  |  |  | 0 | 1 |
| HAM/TSP#1 | 1981 | chr12_3816691_+ |  |  |  |  | 0 | 1 |
| HAM/TSP#1 | 1982 | chr12_40387772_+ |  |  |  |  | 0 | 1 |
| HAM/TSP#1 | 1983 | chr12_43832672_+ |  |  |  |  | 0 | 4 |
| HAM/TSP#1 | 1984 | chr12_4690871_+ |  |  |  |  | 0 | 1 |
| HAM/TSP#1 | 1985 | chr12_47282103_- |  |  |  |  | 0 | 1 |
| HAM/TSP#1 | 1986 | chr12_47928295_- |  |  |  |  | 0 | 1 |
| HAM/TSP#1 | 1987 | chr12_48050287_+ |  |  |  |  | 0 | 1 |
| HAM/TSP#1 | 1988 | chr12_48410439_- |  |  |  |  | 0 | 1 |
| HAM/TSP#1 | 1989 | chr12_48865551_- |  |  |  |  | 0 | 1 |
| HAM/TSP#1 | 1990 | chr12_48934589_+ |  |  |  |  | 0 | 1 |
| HAM/TSP#1 | 1991 | chr12_49185189_- |  |  |  |  | 0 | 1 |
| HAM/TSP#1 | 1992 | chr12_4980961_- |  |  |  |  | 0 | 1 |
| HAM/TSP#1 | 1993 | chr12_51320501_- |  |  |  |  | 0 | 1 |
| HAM/TSP#1 | 1994 | chr12_52789969_- |  |  |  |  | 0 | 1 |
| HAM/TSP#1 | 1995 | chr12_53721993_+ |  |  |  |  | 0 | 1 |
| HAM/TSP#1 | 1996 | chr12_54859303_- |  |  |  |  | 0 | 23 |
| HAM/TSP#1 | 1997 | chr12_56445716_- |  |  |  |  | 0 | 1 |
| HAM/TSP#1 | 1998 | chr12_56810265_- |  |  |  |  | 0 | 1 |
| HAM/TSP#1 | 1999 | chr12_57058533_- |  |  |  |  | 0 | 5 |
| HAM/TSP#1 | 2000 | chr12_57801322_- |  |  |  |  | 0 | 2 |
| HAM/TSP#1 | 2001 | chr12_5902288_+ |  |  |  |  | 0 | 1 |
| HAM/TSP#1 | 2002 | chr12_6077540_+ |  |  |  |  | 0 | 1 |
| HAM/TSP#1 | 2003 | chr12_61599693_- |  |  |  |  | 0 | 1 |
| HAM/TSP#1 | 2004 | chr12_61997521_+ |  |  |  |  | 0 | 3 |
| HAM/TSP#1 | 2005 | chr12_63336482_+ |  |  |  |  | 0 | 8 |
| HAM/TSP#1 | 2006 | chr12_64115817_+ |  |  |  |  | 0 | 1 |
| HAM/TSP#1 | 2007 | chr12_64123769_+ |  |  |  |  | 0 | 1 |
| HAM/TSP#1 | 2008 | chr12_64597170_+ |  |  |  |  | 0 | 1 |
| HAM/TSP#1 | 2009 | chr12_6526532_- |  |  |  |  | 0 | 1 |
| HAM/TSP#1 | 2010 | chr12_66188031_+ |  |  |  |  | 0 | 7 |
| HAM/TSP#1 | 2011 | chr12_67806536_- |  |  |  |  | 0 | 1 |
| HAM/TSP#1 | 2012 | chr12_68252906_- |  |  |  |  | 0 | 1 |
| HAM/TSP#1 | 2013 | chr12_69992441_- |  |  |  |  | 0 | 1 |
| HAM/TSP#1 | 2014 | chr12_7109570_+ |  |  |  |  | 0 | 1 |
| HAM/TSP#1 | 2015 | chr12_72092478_- |  |  |  |  | 0 | 1 |
| HAM/TSP#1 | 2016 | chr12_72585472_+ |  |  |  |  | 0 | 1 |
| HAM/TSP#1 | 2017 | chr12_72640327_- |  |  |  |  | 0 | 1 |
| HAM/TSP#1 | 2018 | chr12_72768592_+ |  |  |  |  | 0 | 1 |
| HAM/TSP#1 | 2019 | chr12_73185838_- |  |  |  |  | 0 | 5 |
| HAM/TSP#1 | 2020 | chr12_73730504_- |  |  |  |  | 0 | 1 |
| HAM/TSP#1 | 2021 | chr12_75639410_+ |  |  |  |  | 0 | 1 |
| HAM/TSP#1 | 2022 | chr12_76069812_- |  |  |  |  | 0 | 1 |
| HAM/TSP#1 | 2023 | chr12_76351377_- |  |  |  |  | 0 | 2 |
| HAM/TSP#1 | 2024 | chr12_76351378_- |  |  |  |  | 0 | 2 |
| HAM/TSP#1 | 2025 | chr12_76415719_+ |  |  |  |  | 0 | 3 |
| HAM/TSP#1 | 2026 | chr12_76737679_- |  |  |  |  | 0 | 1 |
| HAM/TSP#1 | 2027 | chr12_76863150_+ |  |  |  |  | 0 | 1 |
| HAM/TSP#1 | 2028 | chr12_77538588_- |  |  |  |  | 0 | 1 |
| HAM/TSP#1 | 2029 | chr12_80917907_- |  |  |  |  | 0 | 10 |
| HAM/TSP#1 | 2030 | chr12_83400691_+ |  |  |  |  | 0 | 3 |
| HAM/TSP#1 | 2031 | chr12_86120475_- |  |  |  |  | 0 | 1 |
| HAM/TSP#1 | 2032 | chr12_89386777_+ |  |  |  |  | 0 | 1 |
| HAM/TSP#1 | 2033 | chr12_92536170_- |  |  |  |  | 0 | 1 |
| HAM/TSP#1 | 2034 | chr12_94615313_+ |  |  |  |  | 0 | 1 |
| HAM/TSP#1 | 2035 | chr12_94928177_+ |  |  |  |  | 0 | 1 |
| HAM/TSP#1 | 2036 | chr12_95600605_+ |  |  |  |  | 0 | 1 |
| HAM/TSP#1 | 2037 | chr12_96003393_+ |  |  |  |  | 0 | 3 |
| HAM/TSP#1 | 2038 | chr12_99527700_+ |  |  |  |  | 0 | 2 |
| HAM/TSP#1 | 2039 | chr13_103537439_+ |  |  |  |  | 0 | 1 |
| HAM/TSP#1 | 2040 | chr13_104099435_+ |  |  |  |  | 0 | 1 |
| HAM/TSP#1 | 2041 | chr13_104739216_+ |  |  |  |  | 0 | 1 |
| HAM/TSP#1 | 2042 | chr13_105403287_- |  |  |  |  | 0 | 1 |
| HAM/TSP#1 | 2043 | chr13_106750665_- |  |  |  |  | 0 | 1 |
| HAM/TSP#1 | 2044 | chr13_106953719_+ |  |  |  |  | 0 | 1 |
| HAM/TSP#1 | 2045 | chr13_108922719_+ |  |  |  |  | 0 | 3 |
| HAM/TSP#1 | 2046 | chr13_109424258_- |  |  |  |  | 0 | 1 |
| HAM/TSP#1 | 2047 | chr13_109774540_- |  |  |  |  | 0 | 1 |
| HAM/TSP#1 | 2048 | chr13_110558549_- |  |  |  |  | 0 | 1 |
| HAM/TSP#1 | 2049 | chr13_110735120_- |  |  |  |  | 0 | 3 |
| HAM/TSP#1 | 2050 | chr13_112064316_+ |  |  |  |  | 0 | 1 |
| HAM/TSP#1 | 2051 | chr13_112958154_+ |  |  |  |  | 0 | 1 |
| HAM/TSP#1 | 2052 | chr13_113015150_- |  |  |  |  | 0 | 1 |
| HAM/TSP#1 | 2053 | chr13_113507020_+ |  |  |  |  | 0 | 2 |
| HAM/TSP#1 | 2054 | chr13_113805100_+ |  |  |  |  | 0 | 1 |
| HAM/TSP#1 | 2055 | chr13_113908887_- |  |  |  |  | 0 | 2 |
| HAM/TSP#1 | 2056 | chr13_114117976_- |  |  |  |  | 0 | 1 |
| HAM/TSP#1 | 2057 | chr13_19518570_- |  |  |  |  | 0 | 1 |
| HAM/TSP#1 | 2058 | chr13_19560455_+ |  |  |  |  | 0 | 1 |
| HAM/TSP#1 | 2059 | chr13_20009457_+ |  |  |  |  | 0 | 1 |
| HAM/TSP#1 | 2060 | chr13_20334284_+ |  |  |  |  | 0 | 1 |
| HAM/TSP#1 | 2061 | chr13_20425848_- |  |  |  |  | 0 | 1 |
| HAM/TSP#1 | 2062 | chr13_22010887_+ |  |  |  |  | 0 | 3 |
| HAM/TSP#1 | 2063 | chr13_22946614_- |  |  |  |  | 0 | 3 |
| HAM/TSP#1 | 2064 | chr13_24893089_+ |  |  |  |  | 0 | 1 |
| HAM/TSP#1 | 2065 | chr13_25912094_+ |  |  |  |  | 0 | 11 |
| HAM/TSP#1 | 2066 | chr13_26304429_+ |  |  |  |  | 0 | 2 |
| HAM/TSP#1 | 2067 | chr13_27086935_- |  |  |  |  | 0 | 108 |
| HAM/TSP#1 | 2068 | chr13_27870785_+ |  |  |  |  | 0 | 4 |
| HAM/TSP#1 | 2069 | chr13_28774213_+ |  |  |  |  | 0 | 1 |
| HAM/TSP#1 | 2070 | chr13_29042885_- |  |  |  |  | 0 | 4 |
| HAM/TSP#1 | 2071 | chr13_29623075_- |  |  |  |  | 0 | 1 |
| HAM/TSP#1 | 2072 | chr13_30557825_+ |  |  |  |  | 0 | 1 |
| HAM/TSP#1 | 2073 | chr13_31076659_- |  |  |  |  | 0 | 1 |
| HAM/TSP#1 | 2074 | chr13_31300817_+ |  |  |  |  | 0 | 1 |
| HAM/TSP#1 | 2075 | chr13_31822623_+ |  |  |  |  | 0 | 1 |
| HAM/TSP#1 | 2076 | chr13_32173041_+ |  |  |  |  | 0 | 3 |
| HAM/TSP#1 | 2077 | chr13_32771032_- |  |  |  |  | 0 | 1 |
| HAM/TSP#1 | 2078 | chr13_32874136_- |  |  |  |  | 0 | 1 |
| HAM/TSP#1 | 2079 | chr13_34180447_- |  |  |  |  | 0 | 1 |
| HAM/TSP#1 | 2080 | chr13_34692784_+ |  |  |  |  | 0 | 2 |
| HAM/TSP#1 | 2081 | chr13_35514820_- |  |  |  |  | 0 | 2 |
| HAM/TSP#1 | 2082 | chr13_36001335_+ |  |  |  |  | 0 | 1 |
| HAM/TSP#1 | 2083 | chr13_36043080_+ |  |  |  |  | 0 | 2 |
| HAM/TSP#1 | 2084 | chr13_36845773_- |  |  |  |  | 0 | 4 |
| HAM/TSP#1 | 2085 | chr13_37002075_+ |  |  |  |  | 0 | 4 |
| HAM/TSP#1 | 2086 | chr13_37721268_- |  |  |  |  | 0 | 1 |
| HAM/TSP#1 | 2087 | chr13_38969999_+ |  |  |  |  | 0 | 6 |
| HAM/TSP#1 | 2088 | chr13_39944671_- |  |  |  |  | 0 | 1 |
| HAM/TSP#1 | 2089 | chr13_41680694_- |  |  |  |  | 0 | 1 |
| HAM/TSP#1 | 2090 | chr13_42073656_+ |  |  |  |  | 0 | 7 |
| HAM/TSP#1 | 2091 | chr13_42262787_+ |  |  |  |  | 0 | 1 |
| HAM/TSP#1 | 2092 | chr13_43121082_+ |  |  |  |  | 0 | 1 |
| HAM/TSP#1 | 2093 | chr13_44085306_+ |  |  |  |  | 0 | 3 |
| HAM/TSP#1 | 2094 | chr13_45258520_- |  |  |  |  | 0 | 6 |
| HAM/TSP#1 | 2095 | chr13_45414475_- |  |  |  |  | 0 | 1 |
| HAM/TSP#1 | 2096 | chr13_46296218_+ |  |  |  |  | 0 | 2 |
| HAM/TSP#1 | 2097 | chr13_46474322_- |  |  |  |  | 0 | 1 |
| HAM/TSP#1 | 2098 | chr13_49155245_+ |  |  |  |  | 0 | 1 |
| HAM/TSP#1 | 2099 | chr13_49741789_- |  |  |  |  | 0 | 26 |
| HAM/TSP#1 | 2100 | chr13_50799639_+ |  |  |  |  | 0 | 1 |
| HAM/TSP#1 | 2101 | chr13_50947607_- |  |  |  |  | 0 | 1 |
| HAM/TSP#1 | 2102 | chr13_51259740_+ |  |  |  |  | 0 | 1 |
| HAM/TSP#1 | 2103 | chr13_51349328_+ |  |  |  |  | 0 | 5 |
| HAM/TSP#1 | 2104 | chr13_51463479_+ |  |  |  |  | 0 | 3 |
| HAM/TSP#1 | 2105 | chr13_54261668_- |  |  |  |  | 0 | 1 |
| HAM/TSP#1 | 2106 | chr13_55405447_- |  |  |  |  | 0 | 1 |
| HAM/TSP#1 | 2107 | chr13_55966972_- |  |  |  |  | 0 | 8 |
| HAM/TSP#1 | 2108 | chr13_56659568_- |  |  |  |  | 0 | 1 |
| HAM/TSP#1 | 2109 | chr13_56734594_- |  |  |  |  | 0 | 3 |
| HAM/TSP#1 | 2110 | chr13_57453797_+ |  |  |  |  | 0 | 1 |
| HAM/TSP#1 | 2111 | chr13_58333764_+ |  |  |  |  | 0 | 20 |
| HAM/TSP#1 | 2112 | chr13_59276184_- |  |  |  |  | 0 | 2 |
| HAM/TSP#1 | 2113 | chr13_59386670_+ |  |  |  |  | 0 | 1 |
| HAM/TSP#1 | 2114 | chr13_59723331_- |  |  |  |  | 0 | 1 |
| HAM/TSP#1 | 2115 | chr13_60010380_- |  |  |  |  | 0 | 1 |
| HAM/TSP#1 | 2116 | chr13_60091750_- |  |  |  |  | 0 | 2 |
| HAM/TSP#1 | 2117 | chr13_60134263_+ |  |  |  |  | 0 | 21 |
| HAM/TSP#1 | 2118 | chr13_60426388_- |  |  |  |  | 0 | 1 |
| HAM/TSP#1 | 2119 | chr13_60590007_- |  |  |  |  | 0 | 4 |
| HAM/TSP#1 | 2120 | chr13_61782681_+ |  |  |  |  | 0 | 3 |
| HAM/TSP#1 | 2121 | chr13_62269281_- |  |  |  |  | 6 | 0 |
| HAM/TSP#1 | 2122 | chr13_63711826_- |  |  |  |  | 0 | 8 |
| HAM/TSP#1 | 2123 | chr13_65408880_+ |  |  |  |  | 0 | 2 |
| HAM/TSP#1 | 2124 | chr13_67415258_- |  |  |  |  | 0 | 2 |
| HAM/TSP#1 | 2125 | chr13_68005371_- |  |  |  |  | 0 | 1 |
| HAM/TSP#1 | 2126 | chr13_68237397_+ |  |  |  |  | 0 | 2 |
| HAM/TSP#1 | 2127 | chr13_68336849_- |  |  |  |  | 0 | 1 |
| HAM/TSP#1 | 2128 | chr13_70282746_+ |  |  |  |  | 0 | 1 |
| HAM/TSP#1 | 2129 | chr13_71113919_- |  |  |  |  | 0 | 2 |
| HAM/TSP#1 | 2130 | chr13_75427118_+ |  |  |  |  | 0 | 1 |
| HAM/TSP#1 | 2131 | chr13_75495854_- |  |  |  |  | 0 | 1 |
| HAM/TSP#1 | 2132 | chr13_76192618_+ |  |  |  |  | 0 | 1 |
| HAM/TSP#1 | 2133 | chr13_76480040_+ |  |  |  |  | 0 | 3 |
| HAM/TSP#1 | 2134 | chr13_76688973_- |  |  |  |  | 0 | 4 |
| HAM/TSP#1 | 2135 | chr13_78686969_+ |  |  |  |  | 0 | 4 |
| HAM/TSP#1 | 2136 | chr13_79137993_+ |  |  |  |  | 0 | 2 |
| HAM/TSP#1 | 2137 | chr13_82193958_+ |  |  |  |  | 0 | 1 |
| HAM/TSP#1 | 2138 | chr13_82836123_+ |  |  |  |  | 0 | 1 |
| HAM/TSP#1 | 2139 | chr13_84823714_+ |  |  |  |  | 0 | 4 |
| HAM/TSP#1 | 2140 | chr13_85158178_+ |  |  |  |  | 0 | 6 |
| HAM/TSP#1 | 2141 | chr13_85231955_- |  |  |  |  | 0 | 1 |
| HAM/TSP#1 | 2142 | chr13_85328036_+ |  |  |  |  | 0 | 6 |
| HAM/TSP#1 | 2143 | chr13_87879912_+ |  |  |  |  | 0 | 1 |
| HAM/TSP#1 | 2144 | chr13_89243166_+ |  |  |  |  | 0 | 1 |
| HAM/TSP#1 | 2145 | chr13_91483564_- |  |  |  |  | 0 | 1 |
| HAM/TSP#1 | 2146 | chr13_93392765_- |  |  |  |  | 0 | 1 |
| HAM/TSP#1 | 2147 | chr13_96187606_- |  |  |  |  | 0 | 5 |
| HAM/TSP#1 | 2148 | chr13_96510450_+ |  |  |  |  | 0 | 1 |
| HAM/TSP#1 | 2149 | chr13_97219251_- |  |  |  |  | 0 | 4 |
| HAM/TSP#1 | 2150 | chr13_97485399_- |  |  |  |  | 0 | 2 |
| HAM/TSP#1 | 2151 | chr13_99357952_- |  |  |  |  | 0 | 8 |
| HAM/TSP#1 | 2152 | chr13_99442180_+ |  |  |  |  | 0 | 2 |
| HAM/TSP#1 | 2153 | chr14_100841082_+ |  |  |  |  | 0 | 1 |
| HAM/TSP#1 | 2154 | chr14_101846748_- |  |  |  |  | 3 | 48 |
| HAM/TSP#1 | 2155 | chr14_104189339_+ |  |  |  |  | 0 | 1 |
| HAM/TSP#1 | 2156 | chr14_104402447_- |  |  |  |  | 0 | 1 |
| HAM/TSP#1 | 2157 | chr14_104592147_+ |  |  |  |  | 0 | 1 |
| HAM/TSP#1 | 2158 | chr14_104656781_+ |  |  |  |  | 0 | 4 |
| HAM/TSP#1 | 2159 | chr14_104929477_- |  |  |  |  | 0 | 2 |
| HAM/TSP#1 | 2160 | chr14_105079665_+ |  |  |  |  | 0 | 1 |
| HAM/TSP#1 | 2161 | chr14_105291234_+ |  |  |  |  | 0 | 1 |
| HAM/TSP#1 | 2162 | chr14_105946886_+ |  |  |  |  | 0 | 1 |
| HAM/TSP#1 | 2163 | chr14_106423838_+ |  |  |  |  | 0 | 1 |
| HAM/TSP#1 | 2164 | chr14_19745767_- |  |  |  |  | 0 | 2 |
| HAM/TSP#1 | 2165 | chr14_20383049_- |  |  |  |  | 0 | 2 |
| HAM/TSP#1 | 2166 | chr14_20632411_+ |  |  |  |  | 0 | 2 |
| HAM/TSP#1 | 2167 | chr14_20806759_+ |  |  |  |  | 0 | 1 |
| HAM/TSP#1 | 2168 | chr14_21319384_- |  |  |  |  | 0 | 1 |
| HAM/TSP#1 | 2169 | chr14_21616383_+ |  |  |  |  | 0 | 1 |
| HAM/TSP#1 | 2170 | chr14_23123175_- |  |  |  |  | 0 | 2 |
| HAM/TSP#1 | 2171 | chr14_24086517_+ |  |  |  |  | 0 | 1 |
| HAM/TSP#1 | 2172 | chr14_24454710_+ |  |  |  |  | 0 | 1 |
| HAM/TSP#1 | 2173 | chr14_25991417_- |  |  |  |  | 0 | 3 |
| HAM/TSP#1 | 2174 | chr14_27106979_+ |  |  |  |  | 0 | 2 |
| HAM/TSP#1 | 2175 | chr14_27402206_+ |  |  |  |  | 0 | 1 |
| HAM/TSP#1 | 2176 | chr14_29823606_+ |  |  |  |  | 0 | 1 |
| HAM/TSP#1 | 2177 | chr14_29854917_+ |  |  |  |  | 0 | 1 |
| HAM/TSP#1 | 2178 | chr14_33548877_- |  |  |  |  | 0 | 1 |
| HAM/TSP#1 | 2179 | chr14_34184828_+ |  |  |  |  | 0 | 4 |
| HAM/TSP#1 | 2180 | chr14_34189075_+ |  |  |  |  | 3 | 2 |
| HAM/TSP#1 | 2181 | chr14_36378304_- |  |  |  |  | 0 | 1 |
| HAM/TSP#1 | 2182 | chr14_36683510_- |  |  |  |  | 0 | 4 |
| HAM/TSP#1 | 2183 | chr14_37604261_+ |  |  |  |  | 0 | 3 |
| HAM/TSP#1 | 2184 | chr14_38084510_- |  |  |  |  | 0 | 1 |
| HAM/TSP#1 | 2185 | chr14_39139500_+ |  |  |  |  | 0 | 1 |
| HAM/TSP#1 | 2186 | chr14_39264857_+ |  |  |  |  | 0 | 3 |
| HAM/TSP#1 | 2187 | chr14_39293427_- |  |  |  |  | 0 | 2 |
| HAM/TSP#1 | 2188 | chr14_42735461_- |  |  |  |  | 0 | 1 |
| HAM/TSP#1 | 2189 | chr14_44368819_- |  |  |  |  | 0 | 1 |
| HAM/TSP#1 | 2190 | chr14_44973354_+ |  |  |  |  | 0 | 1 |
| HAM/TSP#1 | 2191 | chr14_45917371_+ |  |  |  |  | 0 | 1 |
| HAM/TSP#1 | 2192 | chr14_45995547_- |  |  |  |  | 0 | 1 |
| HAM/TSP#1 | 2193 | chr14_46165271_- |  |  |  |  | 0 | 1 |
| HAM/TSP#1 | 2194 | chr14_46361083_- |  |  |  |  | 0 | 1 |
| HAM/TSP#1 | 2195 | chr14_46889202_+ |  |  |  |  | 0 | 2 |
| HAM/TSP#1 | 2196 | chr14_47244953_- |  |  |  |  | 0 | 2 |
| HAM/TSP#1 | 2197 | chr14_48233436_- |  |  |  |  | 0 | 4 |
| HAM/TSP#1 | 2198 | chr14_48957739_- |  |  |  |  | 0 | 1 |
| HAM/TSP#1 | 2199 | chr14_48967864_+ |  |  |  |  | 0 | 1 |
| HAM/TSP#1 | 2200 | chr14_49109870_- |  |  |  |  | 0 | 1 |
| HAM/TSP#1 | 2201 | chr14_49318935_+ |  |  |  |  | 0 | 1 |
| HAM/TSP#1 | 2202 | chr14_49764508_- |  |  |  |  | 0 | 6 |
| HAM/TSP#1 | 2203 | chr14_49780191_+ |  |  |  |  | 0 | 4 |
| HAM/TSP#1 | 2204 | chr14_50036482_+ |  |  |  |  | 0 | 1 |
| HAM/TSP#1 | 2205 | chr14_53124401_- |  |  |  |  | 0 | 1 |
| HAM/TSP#1 | 2206 | chr14_54948930_+ |  |  |  |  | 0 | 1 |
| HAM/TSP#1 | 2207 | chr14_57684876_+ |  |  |  |  | 0 | 1 |
| HAM/TSP#1 | 2208 | chr14_57741021_- |  |  |  |  | 0 | 1 |
| HAM/TSP#1 | 2209 | chr14_58245705_+ |  |  |  |  | 0 | 2 |
| HAM/TSP#1 | 2210 | chr14_58675748_- |  |  |  |  | 0 | 4 |
| HAM/TSP#1 | 2211 | chr14_58705958_+ |  |  |  |  | 0 | 1 |
| HAM/TSP#1 | 2212 | chr14_59175979_- |  |  |  |  | 0 | 3 |
| HAM/TSP#1 | 2213 | chr14_59408933_+ |  |  |  |  | 0 | 2 |
| HAM/TSP#1 | 2214 | chr14_59827212_- |  |  |  |  | 0 | 1 |
| HAM/TSP#1 | 2215 | chr14_61274136_- |  |  |  |  | 0 | 1 |
| HAM/TSP#1 | 2216 | chr14_61678648_- |  |  |  |  | 0 | 3 |
| HAM/TSP#1 | 2217 | chr14_62005613_+ |  |  |  |  | 0 | 1 |
| HAM/TSP#1 | 2218 | chr14_63313778_- |  |  |  |  | 0 | 1 |
| HAM/TSP#1 | 2219 | chr14_64635071_+ |  |  |  |  | 0 | 1 |
| HAM/TSP#1 | 2220 | chr14_64716910_+ |  |  |  |  | 0 | 1 |
| HAM/TSP#1 | 2221 | chr14_64764652_+ |  |  |  |  | 0 | 1 |
| HAM/TSP#1 | 2222 | chr14_65026420_- |  |  |  |  | 0 | 1 |
| HAM/TSP#1 | 2223 | chr14_65152073_+ |  |  |  |  | 0 | 1 |
| HAM/TSP#1 | 2224 | chr14_67690350_- |  |  |  |  | 0 | 7 |
| HAM/TSP#1 | 2225 | chr14_69158892_+ |  |  |  |  | 0 | 1 |
| HAM/TSP#1 | 2226 | chr14_69768683_+ |  |  |  |  | 0 | 1 |
| HAM/TSP#1 | 2227 | chr14_70013413_+ |  |  |  |  | 0 | 1 |
| HAM/TSP#1 | 2228 | chr14_70816688_- |  |  |  |  | 0 | 2 |
| HAM/TSP#1 | 2229 | chr14_71779630_- |  |  |  |  | 0 | 1 |
| HAM/TSP#1 | 2230 | chr14_72327087_- |  |  |  |  | 0 | 1 |
| HAM/TSP#1 | 2231 | chr14_73057553_- |  |  |  |  | 0 | 1 |
| HAM/TSP#1 | 2232 | chr14_76538584_- |  |  |  |  | 0 | 1 |
| HAM/TSP#1 | 2233 | chr14_77638820_- |  |  |  |  | 0 | 15 |
| HAM/TSP#1 | 2234 | chr14_77638821_- |  |  |  |  | 0 | 1 |
| HAM/TSP#1 | 2235 | chr14_78282924_+ |  |  |  |  | 0 | 1 |
| HAM/TSP#1 | 2236 | chr14_78739945_+ |  |  |  |  | 0 | 1 |
| HAM/TSP#1 | 2237 | chr14_81844860_- |  |  |  |  | 0 | 1 |
| HAM/TSP#1 | 2238 | chr14_83790499_- |  |  |  |  | 0 | 1 |
| HAM/TSP#1 | 2239 | chr14_85935273_- |  |  |  |  | 0 | 3 |
| HAM/TSP#1 | 2240 | chr14_86278582_+ |  |  |  |  | 0 | 1 |
| HAM/TSP#1 | 2241 | chr14_87185135_+ |  |  |  |  | 0 | 1 |
| HAM/TSP#1 | 2242 | chr14_88508790_+ |  |  |  |  | 0 | 12 |
| HAM/TSP#1 | 2243 | chr14_88607471_+ |  |  |  |  | 0 | 5 |
| HAM/TSP#1 | 2244 | chr14_88833801_- |  |  |  |  | 0 | 1 |
| HAM/TSP#1 | 2245 | chr14_89006865_+ |  |  |  |  | 0 | 1 |
| HAM/TSP#1 | 2246 | chr14_89138933_+ |  |  |  |  | 0 | 1 |
| HAM/TSP#1 | 2247 | chr14_89266928_+ |  |  |  |  | 0 | 1 |
| HAM/TSP#1 | 2248 | chr14_89902430_+ |  |  |  |  | 0 | 1 |
| HAM/TSP#1 | 2249 | chr14_90030309_- |  |  |  |  | 0 | 12 |
| HAM/TSP#1 | 2250 | chr14_90397715_- |  |  |  |  | 0 | 2 |
| HAM/TSP#1 | 2251 | chr14_91356284_- |  |  |  |  | 0 | 1 |
| HAM/TSP#1 | 2252 | chr14_92100297_- |  |  |  |  | 0 | 17 |
| HAM/TSP#1 | 2253 | chr14_92724523_+ |  |  |  |  | 0 | 14 |
| HAM/TSP#1 | 2254 | chr14_93812602_+ |  |  |  |  | 0 | 3 |
| HAM/TSP#1 | 2255 | chr14_94307526_+ |  |  |  |  | 0 | 4 |
| HAM/TSP#1 | 2256 | chr14_95176011_- |  |  |  |  | 0 | 5 |
| HAM/TSP#1 | 2257 | chr14_95533633_+ |  |  |  |  | 0 | 1 |
| HAM/TSP#1 | 2258 | chr14_95536834_- |  |  |  |  | 0 | 2 |
| HAM/TSP#1 | 2259 | chr14_96686342_- |  |  |  |  | 0 | 1 |
| HAM/TSP#1 | 2260 | chr14_97967932_- |  |  |  |  | 0 | 1 |
| HAM/TSP#1 | 2261 | chr14_98504378_- |  |  |  |  | 0 | 3 |
| HAM/TSP#1 | 2262 | chr14_99987788_- |  |  |  |  | 0 | 1 |
| HAM/TSP#1 | 2263 | chr15_100050061_- |  |  |  |  | 0 | 4 |
| HAM/TSP#1 | 2264 | chr15_22932333_+ |  |  |  |  | 0 | 1 |
| HAM/TSP#1 | 2265 | chr15_22960206_- |  |  |  |  | 0 | 1 |
| HAM/TSP#1 | 2266 | chr15_23503525_- |  |  |  |  | 0 | 3 |
| HAM/TSP#1 | 2267 | chr15_23618061_+ |  |  |  |  | 0 | 1 |
| HAM/TSP#1 | 2268 | chr15_25069296_- |  |  |  |  | 0 | 1 |
| HAM/TSP#1 | 2269 | chr15_26078044_+ |  |  |  |  | 0 | 3 |
| HAM/TSP#1 | 2270 | chr15_26695396_- |  |  |  |  | 0 | 1 |
| HAM/TSP#1 | 2271 | chr15_26793931_+ |  |  |  |  | 0 | 1 |
| HAM/TSP#1 | 2272 | chr15_28284308_- |  |  |  |  | 0 | 1 |
| HAM/TSP#1 | 2273 | chr15_28818934_+ |  |  |  |  | 0 | 1 |
| HAM/TSP#1 | 2274 | chr15_29082139_- |  |  |  |  | 0 | 1 |
| HAM/TSP#1 | 2275 | chr15_29643031_+ |  |  |  |  | 0 | 1 |
| HAM/TSP#1 | 2276 | chr15_29661752_- |  |  |  |  | 0 | 1 |
| HAM/TSP#1 | 2277 | chr15_29825291_- |  |  |  |  | 0 | 2 |
| HAM/TSP#1 | 2278 | chr15_30896096_- |  |  |  |  | 0 | 1 |
| HAM/TSP#1 | 2279 | chr15_31041021_+ |  |  |  |  | 0 | 4 |
| HAM/TSP#1 | 2280 | chr15_35293446_+ |  |  |  |  | 0 | 3 |
| HAM/TSP#1 | 2281 | chr15_35493842_+ |  |  |  |  | 0 | 2 |
| HAM/TSP#1 | 2282 | chr15_35650879_+ |  |  |  |  | 0 | 1 |
| HAM/TSP#1 | 2283 | chr15_35913320_- |  |  |  |  | 0 | 1 |
| HAM/TSP#1 | 2284 | chr15_36047973_- |  |  |  |  | 0 | 1 |
| HAM/TSP#1 | 2285 | chr15_36742257_+ |  |  |  |  | 0 | 1 |
| HAM/TSP#1 | 2286 | chr15_39201552_+ |  |  |  |  | 0 | 2 |
| HAM/TSP#1 | 2287 | chr15_39459018_- |  |  |  |  | 0 | 2 |
| HAM/TSP#1 | 2288 | chr15_41343591_+ |  |  |  |  | 0 | 4 |
| HAM/TSP#1 | 2289 | chr15_42239496_+ |  |  |  |  | 0 | 2 |
| HAM/TSP#1 | 2290 | chr15_45976433_- |  |  |  |  | 0 | 5 |
| HAM/TSP#1 | 2291 | chr15_46849616_- |  |  |  |  | 0 | 3 |
| HAM/TSP#1 | 2292 | chr15_48045475_+ |  |  |  |  | 0 | 1 |
| HAM/TSP#1 | 2293 | chr15_48495964_+ |  |  |  |  | 2 | 7 |
| HAM/TSP#1 | 2294 | chr15_49520585_+ |  |  |  |  | 0 | 85 |
| HAM/TSP#1 | 2295 | chr15_49716187_- |  |  |  |  | 0 | 1 |
| HAM/TSP#1 | 2296 | chr15_50294162_- |  |  |  |  | 0 | 1 |
| HAM/TSP#1 | 2297 | chr15_50557732_- |  |  |  |  | 0 | 1 |
| HAM/TSP#1 | 2298 | chr15_52078584_+ |  |  |  |  | 0 | 1 |
| HAM/TSP#1 | 2299 | chr15_53253856_- |  |  |  |  | 0 | 2 |
| HAM/TSP#1 | 2300 | chr15_53680450_- |  |  |  |  | 0 | 25 |
| HAM/TSP#1 | 2301 | chr15_54430424_- |  |  |  |  | 0 | 5 |
| HAM/TSP#1 | 2302 | chr15_56141012_+ |  |  |  |  | 0 | 1 |
| HAM/TSP#1 | 2303 | chr15_56782173_+ |  |  |  |  | 0 | 1 |
| HAM/TSP#1 | 2304 | chr15_57003601_+ |  |  |  |  | 0 | 1 |
| HAM/TSP#1 | 2305 | chr15_59039114_- |  |  |  |  | 0 | 2 |
| HAM/TSP#1 | 2306 | chr15_59366056_- |  |  |  |  | 0 | 1 |
| HAM/TSP#1 | 2307 | chr15_60132315_+ |  |  |  |  | 0 | 3 |
| HAM/TSP#1 | 2308 | chr15_60351142_+ |  |  |  |  | 0 | 1 |
| HAM/TSP#1 | 2309 | chr15_60947808_- |  |  |  |  | 0 | 1 |
| HAM/TSP#1 | 2310 | chr15_61696027_+ |  |  |  |  | 0 | 1 |
| HAM/TSP#1 | 2311 | chr15_62605790_- |  |  |  |  | 0 | 1 |
| HAM/TSP#1 | 2312 | chr15_63183979_- |  |  |  |  | 0 | 2 |
| HAM/TSP#1 | 2313 | chr15_63781894_- |  |  |  |  | 0 | 3 |
| HAM/TSP#1 | 2314 | chr15_64566745_+ |  |  |  |  | 0 | 1 |
| HAM/TSP#1 | 2315 | chr15_64597620_+ |  |  |  |  | 0 | 1 |
| HAM/TSP#1 | 2316 | chr15_65182856_+ |  |  |  |  | 0 | 2 |
| HAM/TSP#1 | 2317 | chr15_65442344_- |  |  |  |  | 0 | 1 |
| HAM/TSP#1 | 2318 | chr15_67024739_+ |  |  |  |  | 0 | 1 |
| HAM/TSP#1 | 2319 | chr15_68052148_+ |  |  |  |  | 0 | 3 |
| HAM/TSP#1 | 2320 | chr15_68350673_- |  |  |  |  | 0 | 1 |
| HAM/TSP#1 | 2321 | chr15_69079301_- |  |  |  |  | 0 | 1 |
| HAM/TSP#1 | 2322 | chr15_69500900_- |  |  |  |  | 0 | 1 |
| HAM/TSP#1 | 2323 | chr15_69576421_+ |  |  |  |  | 0 | 1 |
| HAM/TSP#1 | 2324 | chr15_70049307_- |  |  |  |  | 0 | 1 |
| HAM/TSP#1 | 2325 | chr15_70419026_+ |  |  |  |  | 0 | 1 |
| HAM/TSP#1 | 2326 | chr15_70437878_+ |  |  |  |  | 0 | 1 |
| HAM/TSP#1 | 2327 | chr15_75368368_+ |  |  |  |  | 0 | 1 |
| HAM/TSP#1 | 2328 | chr15_75696903_- |  |  |  |  | 0 | 1 |
| HAM/TSP#1 | 2329 | chr15_77423431_- |  |  |  |  | 0 | 2 |
| HAM/TSP#1 | 2330 | chr15_77904981_+ |  |  |  |  | 0 | 1 |
| HAM/TSP#1 | 2331 | chr15_78179156_- |  |  |  |  | 0 | 1 |
| HAM/TSP#1 | 2332 | chr15_80152349_+ |  |  |  |  | 0 | 1 |
| HAM/TSP#1 | 2333 | chr15_80451606_- |  |  |  |  | 0 | 1 |
| HAM/TSP#1 | 2334 | chr15_80781020_- |  |  |  |  | 0 | 1 |
| HAM/TSP#1 | 2335 | chr15_81445780_+ |  |  |  |  | 0 | 1 |
| HAM/TSP#1 | 2336 | chr15_82151389_- |  |  |  |  | 0 | 1 |
| HAM/TSP#1 | 2337 | chr15_83186953_+ |  |  |  |  | 0 | 8 |
| HAM/TSP#1 | 2338 | chr15_83303991_+ |  |  |  |  | 0 | 1 |
| HAM/TSP#1 | 2339 | chr15_86980127_- |  |  |  |  | 5 | 0 |
| HAM/TSP#1 | 2340 | chr15_87833502_+ |  |  |  |  | 0 | 1 |
| HAM/TSP#1 | 2341 | chr15_88933902_- |  |  |  |  | 0 | 1 |
| HAM/TSP#1 | 2342 | chr15_89578323_+ |  |  |  |  | 0 | 1 |
| HAM/TSP#1 | 2343 | chr15_90280072_+ |  |  |  |  | 0 | 1 |
| HAM/TSP#1 | 2344 | chr15_96082882_+ |  |  |  |  | 0 | 2 |
| HAM/TSP#1 | 2345 | chr15_96816717_+ |  |  |  |  | 0 | 1 |
| HAM/TSP#1 | 2346 | chr15_99733215_- |  |  |  |  | 0 | 1 |
| HAM/TSP#1 | 2347 | chr16_10174392_- |  |  |  |  | 0 | 1 |
| HAM/TSP#1 | 2348 | chr16_10248997_+ |  |  |  |  | 0 | 1 |
| HAM/TSP#1 | 2349 | chr16_10578047_+ |  |  |  |  | 0 | 4 |
| HAM/TSP#1 | 2350 | chr16_12541980_- |  |  |  |  | 0 | 1 |
| HAM/TSP#1 | 2351 | chr16_14845198_+ |  |  |  |  | 0 | 1 |
| HAM/TSP#1 | 2352 | chr16_1537966_- |  |  |  |  | 0 | 1 |
| HAM/TSP#1 | 2353 | chr16_16144116_+ |  |  |  |  | 0 | 1 |
| HAM/TSP#1 | 2354 | chr16_16149070_+ |  |  |  |  | 0 | 1 |
| HAM/TSP#1 | 2355 | chr16_18650571_+ |  |  |  |  | 0 | 1 |
| HAM/TSP#1 | 2356 | chr16_20355560_- |  |  |  |  | 0 | 1 |
| HAM/TSP#1 | 2357 | chr16_21994390_+ |  |  |  |  | 0 | 1 |
| HAM/TSP#1 | 2358 | chr16_22382263_+ |  |  |  |  | 0 | 5 |
| HAM/TSP#1 | 2359 | chr16_22724034_- |  |  |  |  | 0 | 1 |
| HAM/TSP#1 | 2360 | chr16_23386832_- |  |  |  |  | 0 | 1 |
| HAM/TSP#1 | 2361 | chr16_23697600_- |  |  |  |  | 0 | 1 |
| HAM/TSP#1 | 2362 | chr16_25171432_- |  |  |  |  | 0 | 2 |
| HAM/TSP#1 | 2363 | chr16_25844623_- |  |  |  |  | 0 | 1 |
| HAM/TSP#1 | 2364 | chr16_25869139_+ |  |  |  |  | 0 | 1 |
| HAM/TSP#1 | 2365 | chr16_27012083_- |  |  |  |  | 0 | 1 |
| HAM/TSP#1 | 2366 | chr16_27330515_- |  |  |  |  | 0 | 1 |
| HAM/TSP#1 | 2367 | chr16_27426965_- |  |  |  |  | 0 | 1 |
| HAM/TSP#1 | 2368 | chr16_28571144_+ |  |  |  |  | 0 | 1 |
| HAM/TSP#1 | 2369 | chr16_29004718_- |  |  |  |  | 0 | 1 |
| HAM/TSP#1 | 2370 | chr16_29309192_- |  |  |  |  | 0 | 1 |
| HAM/TSP#1 | 2371 | chr16_29592380_+ |  |  |  |  | 0 | 4 |
| HAM/TSP#1 | 2372 | chr16_30068828_- |  |  |  |  | 0 | 1 |
| HAM/TSP#1 | 2373 | chr16_3019897_+ |  |  |  |  | 0 | 1 |
| HAM/TSP#1 | 2374 | chr16_30368358_- |  |  |  |  | 0 | 2 |
| HAM/TSP#1 | 2375 | chr16_3090366_+ |  |  |  |  | 0 | 9 |
| HAM/TSP#1 | 2376 | chr16_3090375_+ |  |  |  |  | 0 | 2 |
| HAM/TSP#1 | 2377 | chr16_31028239_+ |  |  |  |  | 0 | 6 |
| HAM/TSP#1 | 2378 | chr16_31038331_+ |  |  |  |  | 0 | 1 |
| HAM/TSP#1 | 2379 | chr16_31857314_- |  |  |  |  | 0 | 2 |
| HAM/TSP#1 | 2380 | chr16_31918749_+ |  |  |  |  | 0 | 1 |
| HAM/TSP#1 | 2381 | chr16_33604335_+ |  |  |  |  | 0 | 1 |
| HAM/TSP#1 | 2382 | chr16_34713508_- |  |  |  |  | 0 | 3 |
| HAM/TSP#1 | 2383 | chr16_35303350_+ |  |  |  |  | 0 | 1 |
| HAM/TSP#1 | 2384 | chr16_35449750_- |  |  |  |  | 0 | 1 |
| HAM/TSP#1 | 2385 | chr16_35513163_- |  |  |  |  | 0 | 5 |
| HAM/TSP#1 | 2386 | chr16_46394458_+ |  |  |  |  | 0 | 1 |
| HAM/TSP#1 | 2387 | chr16_46491441_- |  |  |  |  | 0 | 2 |
| HAM/TSP#1 | 2388 | chr16_46590812_+ |  |  |  |  | 0 | 2 |
| HAM/TSP#1 | 2389 | chr16_47074667_- |  |  |  |  | 0 | 1 |
| HAM/TSP#1 | 2390 | chr16_48492945_+ |  |  |  |  | 0 | 1 |
| HAM/TSP#1 | 2391 | chr16_4997638_- |  |  |  |  | 0 | 1 |
| HAM/TSP#1 | 2392 | chr16_50319822_+ |  |  |  |  | 0 | 1 |
| HAM/TSP#1 | 2393 | chr16_50423504_- |  |  |  |  | 0 | 1 |
| HAM/TSP#1 | 2394 | chr16_51922738_+ |  |  |  |  | 0 | 1 |
| HAM/TSP#1 | 2395 | chr16_52809388_+ |  |  |  |  | 0 | 3 |
| HAM/TSP#1 | 2396 | chr16_53256521_- |  |  |  |  | 0 | 1 |
| HAM/TSP#1 | 2397 | chr16_5345265_+ |  |  |  |  | 0 | 6 |
| HAM/TSP#1 | 2398 | chr16_53475181_+ |  |  |  |  | 0 | 2 |
| HAM/TSP#1 | 2399 | chr16_54004748_+ |  |  |  |  | 0 | 1 |
| HAM/TSP#1 | 2400 | chr16_55400296_+ |  |  |  |  | 0 | 1 |
| HAM/TSP#1 | 2401 | chr16_55755509_+ |  |  |  |  | 0 | 1 |
| HAM/TSP#1 | 2402 | chr16_55842621_- |  |  |  |  | 0 | 1 |
| HAM/TSP#1 | 2403 | chr16_56119072_+ |  |  |  |  | 0 | 1 |
| HAM/TSP#1 | 2404 | chr16_56430444_- |  |  |  |  | 0 | 13 |
| HAM/TSP#1 | 2405 | chr16_57461219_- |  |  |  |  | 0 | 1 |
| HAM/TSP#1 | 2406 | chr16_57810809_- |  |  |  |  | 0 | 1 |
| HAM/TSP#1 | 2407 | chr16_58136584_- |  |  |  |  | 0 | 12 |
| HAM/TSP#1 | 2408 | chr16_59113345_+ |  |  |  |  | 0 | 1 |
| HAM/TSP#1 | 2409 | chr16_59976429_- |  |  |  |  | 0 | 3 |
| HAM/TSP#1 | 2410 | chr16_60549532_- |  |  |  |  | 0 | 1 |
| HAM/TSP#1 | 2411 | chr16_61129937_+ |  |  |  |  | 0 | 1 |
| HAM/TSP#1 | 2412 | chr16_61858840_- |  |  |  |  | 0 | 1 |
| HAM/TSP#1 | 2413 | chr16_63447606_+ |  |  |  |  | 0 | 1 |
| HAM/TSP#1 | 2414 | chr16_63789988_+ |  |  |  |  | 0 | 1 |
| HAM/TSP#1 | 2415 | chr16_6392694_- |  |  |  |  | 0 | 1 |
| HAM/TSP#1 | 2416 | chr16_64187597_+ |  |  |  |  | 0 | 5 |
| HAM/TSP#1 | 2417 | chr16_6607263_+ |  |  |  |  | 0 | 1 |
| HAM/TSP#1 | 2418 | chr16_664085_- |  |  |  |  | 0 | 1 |
| HAM/TSP#1 | 2419 | chr16_66492829_+ |  |  |  |  | 0 | 1 |
| HAM/TSP#1 | 2420 | chr16_67006843_+ |  |  |  |  | 0 | 2 |
| HAM/TSP#1 | 2421 | chr16_67776255_- |  |  |  |  | 0 | 15 |
| HAM/TSP#1 | 2422 | chr16_69369274_+ |  |  |  |  | 0 | 1 |
| HAM/TSP#1 | 2423 | chr16_70214133_- |  |  |  |  | 0 | 1 |
| HAM/TSP#1 | 2424 | chr16_70312671_- |  |  |  |  | 0 | 1 |
| HAM/TSP#1 | 2425 | chr16_70481096_- |  |  |  |  | 0 | 1 |
| HAM/TSP#1 | 2426 | chr16_72518011_+ |  |  |  |  | 0 | 6 |
| HAM/TSP#1 | 2427 | chr16_72843079_- |  |  |  |  | 0 | 6 |
| HAM/TSP#1 | 2428 | chr16_72955423_+ |  |  |  |  | 0 | 1 |
| HAM/TSP#1 | 2429 | chr16_73854923_+ |  |  |  |  | 3 | 0 |
| HAM/TSP#1 | 2430 | chr16_74401580_- |  |  |  |  | 0 | 1 |
| HAM/TSP#1 | 2431 | chr16_74445153_- |  |  |  |  | 0 | 1 |
| HAM/TSP#1 | 2432 | chr16_75607241_- |  |  |  |  | 1 | 29 |
| HAM/TSP#1 | 2433 | chr16_7638728_- |  |  |  |  | 0 | 1 |
| HAM/TSP#1 | 2434 | chr16_76587363_- |  |  |  |  | 0 | 1 |
| HAM/TSP#1 | 2435 | chr16_7702775_- |  |  |  |  | 0 | 1 |
| HAM/TSP#1 | 2436 | chr16_78383612_+ |  |  |  |  | 0 | 9 |
| HAM/TSP#1 | 2437 | chr16_79005670_- |  |  |  |  | 0 | 1 |
| HAM/TSP#1 | 2438 | chr16_81322425_+ |  |  |  |  | 0 | 2 |
| HAM/TSP#1 | 2439 | chr16_81765317_- |  |  |  |  | 0 | 1 |
| HAM/TSP#1 | 2440 | chr16_83511077_+ |  |  |  |  | 0 | 1 |
| HAM/TSP#1 | 2441 | chr16_83863496_- |  |  |  |  | 0 | 1 |
| HAM/TSP#1 | 2442 | chr16_84507428_+ |  |  |  |  | 0 | 5 |
| HAM/TSP#1 | 2443 | chr16_85525502_+ |  |  |  |  | 0 | 1 |
| HAM/TSP#1 | 2444 | chr16_85604206_- |  |  |  |  | 0 | 1 |
| HAM/TSP#1 | 2445 | chr16_85604208_- |  |  |  |  | 0 | 6 |
| HAM/TSP#1 | 2446 | chr16_85624266_+ |  |  |  |  | 0 | 1 |
| HAM/TSP#1 | 2447 | chr16_85637259_- |  |  |  |  | 0 | 1 |
| HAM/TSP#1 | 2448 | chr16_86601279_+ |  |  |  |  | 0 | 2 |
| HAM/TSP#1 | 2449 | chr16_87195754_- |  |  |  |  | 0 | 1 |
| HAM/TSP#1 | 2450 | chr16_87382614_- |  |  |  |  | 0 | 7 |
| HAM/TSP#1 | 2451 | chr16_87817890_+ |  |  |  |  | 0 | 1 |
| HAM/TSP#1 | 2452 | chr16_87964229_+ |  |  |  |  | 0 | 1 |
| HAM/TSP#1 | 2453 | chr16_8869642_+ |  |  |  |  | 0 | 1 |
| HAM/TSP#1 | 2454 | chr16_89000072_+ |  |  |  |  | 0 | 2 |
| HAM/TSP#1 | 2455 | chr16_89024331_- |  |  |  |  | 0 | 1 |
| HAM/TSP#1 | 2456 | chr16_89075718_- |  |  |  |  | 0 | 1 |
| HAM/TSP#1 | 2457 | chr16_89434667_+ |  |  |  |  | 0 | 1 |
| HAM/TSP#1 | 2458 | chr17_10196970_+ |  |  |  |  | 0 | 1 |
| HAM/TSP#1 | 2459 | chr17_11068902_- |  |  |  |  | 0 | 2 |
| HAM/TSP#1 | 2460 | chr17_11216478_- |  |  |  |  | 0 | 4 |
| HAM/TSP#1 | 2461 | chr17_11993683_- |  |  |  |  | 0 | 1 |
| HAM/TSP#1 | 2462 | chr17_1283802_+ |  |  |  |  | 2 | 1 |
| HAM/TSP#1 | 2463 | chr17_13333732_- |  |  |  |  | 0 | 1 |
| HAM/TSP#1 | 2464 | chr17_13344161_+ |  |  |  |  | 0 | 2 |
| HAM/TSP#1 | 2465 | chr17_14933653_+ |  |  |  |  | 0 | 1 |
| HAM/TSP#1 | 2466 | chr17_15575491_+ |  |  |  |  | 0 | 1 |
| HAM/TSP#1 | 2467 | chr17_1634656_+ |  |  |  |  | 0 | 6 |
| HAM/TSP#1 | 2468 | chr17_17262402_- |  |  |  |  | 0 | 1 |
| HAM/TSP#1 | 2469 | chr17_17517130_+ |  |  |  |  | 0 | 2 |
| HAM/TSP#1 | 2470 | chr17_17588136_+ |  |  |  |  | 0 | 1 |
| HAM/TSP#1 | 2471 | chr17_18303306_+ |  |  |  |  | 0 | 11 |
| HAM/TSP#1 | 2472 | chr17_18303315_+ |  |  |  |  | 0 | 1 |
| HAM/TSP#1 | 2473 | chr17_20976152_- |  |  |  |  | 0 | 1 |
| HAM/TSP#1 | 2474 | chr17_20999691_+ |  |  |  |  | 0 | 3 |
| HAM/TSP#1 | 2475 | chr17_21291983_- |  |  |  |  | 0 | 1 |
| HAM/TSP#1 | 2476 | chr17_21462462_+ |  |  |  |  | 0 | 1 |
| HAM/TSP#1 | 2477 | chr17_21772319_+ |  |  |  |  | 0 | 1 |
| HAM/TSP#1 | 2478 | chr17_226716_+ |  |  |  |  | 0 | 2 |
| HAM/TSP#1 | 2479 | chr17_23177049_+ |  |  |  |  | 0 | 1 |
| HAM/TSP#1 | 2480 | chr17_25443016_- |  |  |  |  | 0 | 1 |
| HAM/TSP#1 | 2481 | chr17_26603721_+ |  |  |  |  | 0 | 1 |
| HAM/TSP#1 | 2482 | chr17_26619637_- |  |  |  |  | 0 | 7 |
| HAM/TSP#1 | 2483 | chr17_27453769_+ |  |  |  |  | 0 | 1 |
| HAM/TSP#1 | 2484 | chr17_27501887_+ |  |  |  |  | 0 | 2 |
| HAM/TSP#1 | 2485 | chr17_28090911_+ |  |  |  |  | 0 | 1 |
| HAM/TSP#1 | 2486 | chr17_29032595_- |  |  |  |  | 0 | 1 |
| HAM/TSP#1 | 2487 | chr17_31188913_- |  |  |  |  | 0 | 1 |
| HAM/TSP#1 | 2488 | chr17_3146152_+ |  |  |  |  | 0 | 1 |
| HAM/TSP#1 | 2489 | chr17_32078719_- |  |  |  |  | 0 | 1 |
| HAM/TSP#1 | 2490 | chr17_32371906_+ |  |  |  |  | 0 | 7 |
| HAM/TSP#1 | 2491 | chr17_33933550_- |  |  |  |  | 0 | 1 |
| HAM/TSP#1 | 2492 | chr17_33939970_- |  |  |  |  | 0 | 1 |
| HAM/TSP#1 | 2493 | chr17_35170985_+ |  |  |  |  | 0 | 1 |
| HAM/TSP#1 | 2494 | chr17_36701921_+ |  |  |  |  | 0 | 1 |
| HAM/TSP#1 | 2495 | chr17_37009257_+ |  |  |  |  | 0 | 1 |
| HAM/TSP#1 | 2496 | chr17_38708004_- |  |  |  |  | 0 | 2 |
| HAM/TSP#1 | 2497 | chr17_38738226_- |  |  |  |  | 0 | 1 |
| HAM/TSP#1 | 2498 | chr17_39602982_+ |  |  |  |  | 0 | 1 |
| HAM/TSP#1 | 2499 | chr17_39906008_+ |  |  |  |  | 0 | 1 |
| HAM/TSP#1 | 2500 | chr17_41275941_+ |  |  |  |  | 0 | 1 |
| HAM/TSP#1 | 2501 | chr17_42170241_- |  |  |  |  | 0 | 1 |
| HAM/TSP#1 | 2502 | chr17_4249226_+ |  |  |  |  | 0 | 1 |
| HAM/TSP#1 | 2503 | chr17_42618148_+ |  |  |  |  | 0 | 4 |
| HAM/TSP#1 | 2504 | chr17_42654221_+ |  |  |  |  | 0 | 1 |
| HAM/TSP#1 | 2505 | chr17_44624423_- |  |  |  |  | 0 | 3 |
| HAM/TSP#1 | 2506 | chr17_45852735_- |  |  |  |  | 0 | 1 |
| HAM/TSP#1 | 2507 | chr17_48894488_- |  |  |  |  | 0 | 1 |
| HAM/TSP#1 | 2508 | chr17_49402762_- |  |  |  |  | 0 | 1 |
| HAM/TSP#1 | 2509 | chr17_50957377_+ |  |  |  |  | 0 | 1 |
| HAM/TSP#1 | 2510 | chr17_51821525_- |  |  |  |  | 0 | 3 |
| HAM/TSP#1 | 2511 | chr17_52883502_+ |  |  |  |  | 0 | 1 |
| HAM/TSP#1 | 2512 | chr17_53122349_+ |  |  |  |  | 0 | 8 |
| HAM/TSP#1 | 2513 | chr17_5320404_+ |  |  |  |  | 0 | 8 |
| HAM/TSP#1 | 2514 | chr17_53412584_+ |  |  |  |  | 0 | 1 |
| HAM/TSP#1 | 2515 | chr17_53766263_- |  |  |  |  | 0 | 5 |
| HAM/TSP#1 | 2516 | chr17_54187044_+ |  |  |  |  | 0 | 1 |
| HAM/TSP#1 | 2517 | chr17_5419411_+ |  |  |  |  | 0 | 1 |
| HAM/TSP#1 | 2518 | chr17_54394407_- |  |  |  |  | 0 | 1 |
| HAM/TSP#1 | 2519 | chr17_55159104_+ |  |  |  |  | 0 | 1 |
| HAM/TSP#1 | 2520 | chr17_55732234_+ |  |  |  |  | 0 | 2 |
| HAM/TSP#1 | 2521 | chr17_56183934_+ |  |  |  |  | 0 | 1 |
| HAM/TSP#1 | 2522 | chr17_5668983_- |  |  |  |  | 0 | 1 |
| HAM/TSP#1 | 2523 | chr17_56749612_+ |  |  |  |  | 0 | 1 |
| HAM/TSP#1 | 2524 | chr17_58906626_- |  |  |  |  | 0 | 2 |
| HAM/TSP#1 | 2525 | chr17_5900266_+ |  |  |  |  | 0 | 1 |
| HAM/TSP#1 | 2526 | chr17_60517410_+ |  |  |  |  | 0 | 2 |
| HAM/TSP#1 | 2527 | chr17_61208865_- |  |  |  |  | 0 | 1 |
| HAM/TSP#1 | 2528 | chr17_61424555_- |  |  |  |  | 0 | 1 |
| HAM/TSP#1 | 2529 | chr17_61743336_+ |  |  |  |  | 0 | 3 |
| HAM/TSP#1 | 2530 | chr17_64143964_+ |  |  |  |  | 0 | 1 |
| HAM/TSP#1 | 2531 | chr17_65015787_- |  |  |  |  | 0 | 8 |
| HAM/TSP#1 | 2532 | chr17_65279006_- |  |  |  |  | 0 | 1 |
| HAM/TSP#1 | 2533 | chr17_66093148_- |  |  |  |  | 0 | 1 |
| HAM/TSP#1 | 2534 | chr17_6776751_- |  |  |  |  | 0 | 10 |
| HAM/TSP#1 | 2535 | chr17_68406127_+ |  |  |  |  | 0 | 1 |
| HAM/TSP#1 | 2536 | chr17_68677049_- |  |  |  |  | 0 | 1 |
| HAM/TSP#1 | 2537 | chr17_7032071_- |  |  |  |  | 0 | 2 |
| HAM/TSP#1 | 2538 | chr17_7032080_- |  |  |  |  | 0 | 96 |
| HAM/TSP#1 | 2539 | chr17_72306496_- |  |  |  |  | 0 | 1 |
| HAM/TSP#1 | 2540 | chr17_72525444_- |  |  |  |  | 0 | 4 |
| HAM/TSP#1 | 2541 | chr17_72541676_+ |  |  |  |  | 0 | 1 |
| HAM/TSP#1 | 2542 | chr17_72768241_+ |  |  |  |  | 0 | 1 |
| HAM/TSP#1 | 2543 | chr17_73169704_+ |  |  |  |  | 0 | 1 |
| HAM/TSP#1 | 2544 | chr17_73560849_+ |  |  |  |  | 0 | 1 |
| HAM/TSP#1 | 2545 | chr17_7374296_+ |  |  |  |  | 0 | 1 |
| HAM/TSP#1 | 2546 | chr17_73935836_- |  |  |  |  | 0 | 7 |
| HAM/TSP#1 | 2547 | chr17_7438924_+ |  |  |  |  | 0 | 2 |
| HAM/TSP#1 | 2548 | chr17_74648268_+ |  |  |  |  | 0 | 1 |
| HAM/TSP#1 | 2549 | chr17_75093986_- |  |  |  |  | 0 | 4 |
| HAM/TSP#1 | 2550 | chr17_7604044_+ |  |  |  |  | 0 | 1 |
| HAM/TSP#1 | 2551 | chr17_76237277_- |  |  |  |  | 0 | 2 |
| HAM/TSP#1 | 2552 | chr17_7654478_+ |  |  |  |  | 0 | 1 |
| HAM/TSP#1 | 2553 | chr17_77266591_- |  |  |  |  | 0 | 1 |
| HAM/TSP#1 | 2554 | chr17_77280497_- |  |  |  |  | 0 | 1 |
| HAM/TSP#1 | 2555 | chr17_77322340_+ |  |  |  |  | 0 | 1 |
| HAM/TSP#1 | 2556 | chr17_77598105_+ |  |  |  |  | 0 | 1 |
| HAM/TSP#1 | 2557 | chr17_79609988_+ |  |  |  |  | 0 | 10 |
| HAM/TSP#1 | 2558 | chr17_81049937_- |  |  |  |  | 0 | 1 |
| HAM/TSP#1 | 2559 | chr17_8128604_- |  |  |  |  | 0 | 1 |
| HAM/TSP#1 | 2560 | chr17_82086114_- |  |  |  |  | 0 | 1 |
| HAM/TSP#1 | 2561 | chr17_82649634_+ |  |  |  |  | 0 | 1 |
| HAM/TSP#1 | 2562 | chr17_82906786_- |  |  |  |  | 0 | 1 |
| HAM/TSP#1 | 2563 | chr17_8373666_+ |  |  |  |  | 0 | 1 |
| HAM/TSP#1 | 2564 | chr17_8924480_+ |  |  |  |  | 0 | 1 |
| HAM/TSP#1 | 2565 | chr18_1005590_- |  |  |  |  | 0 | 1 |
| HAM/TSP#1 | 2566 | chr18_10972779_- |  |  |  |  | 0 | 34 |
| HAM/TSP#1 | 2567 | chr18_11369804_+ |  |  |  |  | 0 | 6 |
| HAM/TSP#1 | 2568 | chr18_12847336_+ |  |  |  |  | 0 | 2 |
| HAM/TSP#1 | 2569 | chr18_13266050_+ |  |  |  |  | 0 | 6 |
| HAM/TSP#1 | 2570 | chr18_13279966_- |  |  |  |  | 0 | 1 |
| HAM/TSP#1 | 2571 | chr18_13572158_- |  |  |  |  | 0 | 3 |
| HAM/TSP#1 | 2572 | chr18_13924860_+ |  |  |  |  | 0 | 1 |
| HAM/TSP#1 | 2573 | chr18_139972_- |  |  |  |  | 0 | 1 |
| HAM/TSP#1 | 2574 | chr18_14151765_+ |  |  |  |  | 0 | 1 |
| HAM/TSP#1 | 2575 | chr18_1430963_+ |  |  |  |  | 0 | 1 |
| HAM/TSP#1 | 2576 | chr18_165897_+ |  |  |  |  | 0 | 3 |
| HAM/TSP#1 | 2577 | chr18_21359067_+ |  |  |  |  | 0 | 1 |
| HAM/TSP#1 | 2578 | chr18_21567734_- |  |  |  |  | 0 | 1 |
| HAM/TSP#1 | 2579 | chr18_21754514_- |  |  |  |  | 0 | 1 |
| HAM/TSP#1 | 2580 | chr18_22097217_- |  |  |  |  | 0 | 2 |
| HAM/TSP#1 | 2581 | chr18_22935097_+ |  |  |  |  | 0 | 2 |
| HAM/TSP#1 | 2582 | chr18_25507239_- |  |  |  |  | 0 | 1 |
| HAM/TSP#1 | 2583 | chr18_26531089_+ |  |  |  |  | 0 | 1 |
| HAM/TSP#1 | 2584 | chr18_27980346_+ |  |  |  |  | 0 | 1 |
| HAM/TSP#1 | 2585 | chr18_30481837_+ |  |  |  |  | 0 | 6 |
| HAM/TSP#1 | 2586 | chr18_34718839_- |  |  |  |  | 0 | 43 |
| HAM/TSP#1 | 2587 | chr18_35136539_+ |  |  |  |  | 0 | 1 |
| HAM/TSP#1 | 2588 | chr18_36106565_- |  |  |  |  | 0 | 1 |
| HAM/TSP#1 | 2589 | chr18_36327356_+ |  |  |  |  | 0 | 1 |
| HAM/TSP#1 | 2590 | chr18_36884471_+ |  |  |  |  | 0 | 3 |
| HAM/TSP#1 | 2591 | chr18_37296347_- |  |  |  |  | 0 | 1 |
| HAM/TSP#1 | 2592 | chr18_38849533_+ |  |  |  |  | 0 | 6 |
| HAM/TSP#1 | 2593 | chr18_41553945_+ |  |  |  |  | 0 | 1 |
| HAM/TSP#1 | 2594 | chr18_4328312_+ |  |  |  |  | 0 | 7 |
| HAM/TSP#1 | 2595 | chr18_44672130_- |  |  |  |  | 0 | 1 |
| HAM/TSP#1 | 2596 | chr18_46889911_+ |  |  |  |  | 4 | 11 |
| HAM/TSP#1 | 2597 | chr18_47684278_- |  |  |  |  | 0 | 1 |
| HAM/TSP#1 | 2598 | chr18_47737902_- |  |  |  |  | 0 | 1 |
| HAM/TSP#1 | 2599 | chr18_48729095_+ |  |  |  |  | 0 | 1 |
| HAM/TSP#1 | 2600 | chr18_48770129_+ |  |  |  |  | 0 | 2 |
| HAM/TSP#1 | 2601 | chr18_49284184_- |  |  |  |  | 0 | 1 |
| HAM/TSP#1 | 2602 | chr18_50057219_- |  |  |  |  | 0 | 1 |
| HAM/TSP#1 | 2603 | chr18_50469619_+ |  |  |  |  | 0 | 1 |
| HAM/TSP#1 | 2604 | chr18_50586720_+ |  |  |  |  | 0 | 1 |
| HAM/TSP#1 | 2605 | chr18_51024979_- |  |  |  |  | 0 | 1 |
| HAM/TSP#1 | 2606 | chr18_54714481_- |  |  |  |  | 0 | 1 |
| HAM/TSP#1 | 2607 | chr18_54741477_- |  |  |  |  | 0 | 1 |
| HAM/TSP#1 | 2608 | chr18_55440007_- |  |  |  |  | 0 | 6 |
| HAM/TSP#1 | 2609 | chr18_56326582_+ |  |  |  |  | 0 | 1 |
| HAM/TSP#1 | 2610 | chr18_56473578_+ |  |  |  |  | 0 | 1 |
| HAM/TSP#1 | 2611 | chr18_56905996_- |  |  |  |  | 0 | 1 |
| HAM/TSP#1 | 2612 | chr18_57154293_- |  |  |  |  | 0 | 1 |
| HAM/TSP#1 | 2613 | chr18_57301095_+ |  |  |  |  | 0 | 13 |
| HAM/TSP#1 | 2614 | chr18_5879455_- |  |  |  |  | 0 | 14 |
| HAM/TSP#1 | 2615 | chr18_59051992_+ |  |  |  |  | 0 | 1 |
| HAM/TSP#1 | 2616 | chr18_60632938_- |  |  |  |  | 0 | 2 |
| HAM/TSP#1 | 2617 | chr18_63246888_- |  |  |  |  | 0 | 8 |
| HAM/TSP#1 | 2618 | chr18_64082608_+ |  |  |  |  | 0 | 1 |
| HAM/TSP#1 | 2619 | chr18_64566956_- |  |  |  |  | 0 | 1 |
| HAM/TSP#1 | 2620 | chr18_6469432_- |  |  |  |  | 0 | 1 |
| HAM/TSP#1 | 2621 | chr18_65162193_- |  |  |  |  | 0 | 3 |
| HAM/TSP#1 | 2622 | chr18_68122388_- |  |  |  |  | 0 | 1 |
| HAM/TSP#1 | 2623 | chr18_68588397_+ |  |  |  |  | 0 | 1 |
| HAM/TSP#1 | 2624 | chr18_69852656_+ |  |  |  |  | 0 | 5 |
| HAM/TSP#1 | 2625 | chr18_71197863_- |  |  |  |  | 0 | 2 |
| HAM/TSP#1 | 2626 | chr18_71662085_- |  |  |  |  | 0 | 2 |
| HAM/TSP#1 | 2627 | chr18_72819427_- |  |  |  |  | 0 | 1 |
| HAM/TSP#1 | 2628 | chr18_73099120_+ |  |  |  |  | 0 | 1 |
| HAM/TSP#1 | 2629 | chr18_74460858_- |  |  |  |  | 0 | 1 |
| HAM/TSP#1 | 2630 | chr18_74968132_- |  |  |  |  | 0 | 1 |
| HAM/TSP#1 | 2631 | chr18_75824881_+ |  |  |  |  | 0 | 1 |
| HAM/TSP#1 | 2632 | chr18_76140777_+ |  |  |  |  | 0 | 1 |
| HAM/TSP#1 | 2633 | chr18_76725626_- |  |  |  |  | 0 | 2 |
| HAM/TSP#1 | 2634 | chr18_7809124_+ |  |  |  |  | 0 | 1 |
| HAM/TSP#1 | 2635 | chr18_7868940_- |  |  |  |  | 0 | 1 |
| HAM/TSP#1 | 2636 | chr18_79403751_+ |  |  |  |  | 0 | 1 |
| HAM/TSP#1 | 2637 | chr18_79701106_- |  |  |  |  | 0 | 1 |
| HAM/TSP#1 | 2638 | chr18_816655_- |  |  |  |  | 0 | 1 |
| HAM/TSP#1 | 2639 | chr18_9292569_+ |  |  |  |  | 0 | 1 |
| HAM/TSP#1 | 2640 | chr18_9409782_+ |  |  |  |  | 0 | 4 |
| HAM/TSP#1 | 2641 | chr18_9534463_+ |  |  |  |  | 0 | 1 |
| HAM/TSP#1 | 2642 | chr19_10641257_- |  |  |  |  | 0 | 2 |
| HAM/TSP#1 | 2643 | chr19_11579084_+ |  |  |  |  | 0 | 1 |
| HAM/TSP#1 | 2644 | chr19_12788756_+ |  |  |  |  | 0 | 1 |
| HAM/TSP#1 | 2645 | chr19_12837582_- |  |  |  |  | 0 | 1 |
| HAM/TSP#1 | 2646 | chr19_13104451_- |  |  |  |  | 0 | 9 |
| HAM/TSP#1 | 2647 | chr19_1328443_- |  |  |  |  | 0 | 1 |
| HAM/TSP#1 | 2648 | chr19_13318528_- |  |  |  |  | 0 | 1 |
| HAM/TSP#1 | 2649 | chr19_13933057_+ |  |  |  |  | 0 | 1 |
| HAM/TSP#1 | 2650 | chr19_14415528_+ |  |  |  |  | 0 | 1 |
| HAM/TSP#1 | 2651 | chr19_1455784_- |  |  |  |  | 0 | 1 |
| HAM/TSP#1 | 2652 | chr19_1471973_- |  |  |  |  | 0 | 1 |
| HAM/TSP#1 | 2653 | chr19_1471974_- |  |  |  |  | 0 | 9 |
| HAM/TSP#1 | 2654 | chr19_16859648_- |  |  |  |  | 0 | 1 |
| HAM/TSP#1 | 2655 | chr19_16997754_+ |  |  |  |  | 0 | 1 |
| HAM/TSP#1 | 2656 | chr19_17632714_+ |  |  |  |  | 0 | 1 |
| HAM/TSP#1 | 2657 | chr19_17811544_- |  |  |  |  | 0 | 1 |
| HAM/TSP#1 | 2658 | chr19_18268553_+ |  |  |  |  | 0 | 1 |
| HAM/TSP#1 | 2659 | chr19_18790999_+ |  |  |  |  | 0 | 1 |
| HAM/TSP#1 | 2660 | chr19_19052643_- |  |  |  |  | 0 | 10 |
| HAM/TSP#1 | 2661 | chr19_19141354_+ |  |  |  |  | 0 | 2 |
| HAM/TSP#1 | 2662 | chr19_1967145_- |  |  |  |  | 0 | 1 |
| HAM/TSP#1 | 2663 | chr19_19904984_+ |  |  |  |  | 0 | 1 |
| HAM/TSP#1 | 2664 | chr19_20196615_- |  |  |  |  | 0 | 2 |
| HAM/TSP#1 | 2665 | chr19_21169010_- |  |  |  |  | 0 | 1 |
| HAM/TSP#1 | 2666 | chr19_21630857_+ |  |  |  |  | 0 | 1 |
| HAM/TSP#1 | 2667 | chr19_21630858_+ |  |  |  |  | 0 | 4 |
| HAM/TSP#1 | 2668 | chr19_22326508_- |  |  |  |  | 0 | 1 |
| HAM/TSP#1 | 2669 | chr19_24346227_- |  |  |  |  | 0 | 1 |
| HAM/TSP#1 | 2670 | chr19_2484351_+ |  |  |  |  | 0 | 1 |
| HAM/TSP#1 | 2671 | chr19_27785298_+ |  |  |  |  | 0 | 15 |
| HAM/TSP#1 | 2672 | chr19_29680395_+ |  |  |  |  | 0 | 1 |
| HAM/TSP#1 | 2673 | chr19_30562926_- |  |  |  |  | 0 | 4 |
| HAM/TSP#1 | 2674 | chr19_30717699_- |  |  |  |  | 0 | 1 |
| HAM/TSP#1 | 2675 | chr19_32924207_+ |  |  |  |  | 0 | 3 |
| HAM/TSP#1 | 2676 | chr19_3336113_+ |  |  |  |  | 0 | 1 |
| HAM/TSP#1 | 2677 | chr19_33689238_+ |  |  |  |  | 0 | 1 |
| HAM/TSP#1 | 2678 | chr19_34713308_+ |  |  |  |  | 0 | 1 |
| HAM/TSP#1 | 2679 | chr19_34963119_- |  |  |  |  | 0 | 6 |
| HAM/TSP#1 | 2680 | chr19_35333277_- |  |  |  |  | 0 | 2 |
| HAM/TSP#1 | 2681 | chr19_35383011_- |  |  |  |  | 0 | 1 |
| HAM/TSP#1 | 2682 | chr19_35784937_- |  |  |  |  | 0 | 1 |
| HAM/TSP#1 | 2683 | chr19_36143119_- |  |  |  |  | 0 | 14 |
| HAM/TSP#1 | 2684 | chr19_37403440_+ |  |  |  |  | 0 | 1 |
| HAM/TSP#1 | 2685 | chr19_37926838_- |  |  |  |  | 0 | 1 |
| HAM/TSP#1 | 2686 | chr19_38229983_- |  |  |  |  | 0 | 1 |
| HAM/TSP#1 | 2687 | chr19_38798708_+ |  |  |  |  | 0 | 1 |
| HAM/TSP#1 | 2688 | chr19_39304813_- |  |  |  |  | 0 | 1 |
| HAM/TSP#1 | 2689 | chr19_39754655_+ |  |  |  |  | 0 | 1 |
| HAM/TSP#1 | 2690 | chr19_4058917_+ |  |  |  |  | 0 | 1 |
| HAM/TSP#1 | 2691 | chr19_40768436_+ |  |  |  |  | 0 | 5 |
| HAM/TSP#1 | 2692 | chr19_43948633_+ |  |  |  |  | 0 | 1 |
| HAM/TSP#1 | 2693 | chr19_44048834_- |  |  |  |  | 0 | 1 |
| HAM/TSP#1 | 2694 | chr19_4406458_- |  |  |  |  | 0 | 4 |
| HAM/TSP#1 | 2695 | chr19_44287338_- |  |  |  |  | 0 | 1 |
| HAM/TSP#1 | 2696 | chr19_44528632_- |  |  |  |  | 0 | 1 |
| HAM/TSP#1 | 2697 | chr19_45345476_+ |  |  |  |  | 0 | 5 |
| HAM/TSP#1 | 2698 | chr19_46227814_- |  |  |  |  | 0 | 3 |
| HAM/TSP#1 | 2699 | chr19_46594288_+ |  |  |  |  | 0 | 4 |
| HAM/TSP#1 | 2700 | chr19_47469033_- |  |  |  |  | 0 | 1 |
| HAM/TSP#1 | 2701 | chr19_47627208_+ |  |  |  |  | 0 | 1 |
| HAM/TSP#1 | 2702 | chr19_48369229_+ |  |  |  |  | 0 | 1 |
| HAM/TSP#1 | 2703 | chr19_4939598_+ |  |  |  |  | 0 | 1 |
| HAM/TSP#1 | 2704 | chr19_49838399_- |  |  |  |  | 0 | 2 |
| HAM/TSP#1 | 2705 | chr19_50332588_+ |  |  |  |  | 0 | 1 |
| HAM/TSP#1 | 2706 | chr19_50688108_- |  |  |  |  | 0 | 1 |
| HAM/TSP#1 | 2707 | chr19_51101225_+ |  |  |  |  | 0 | 28 |
| HAM/TSP#1 | 2708 | chr19_51545963_- |  |  |  |  | 0 | 1 |
| HAM/TSP#1 | 2709 | chr19_53662810_+ |  |  |  |  | 0 | 1 |
| HAM/TSP#1 | 2710 | chr19_54726543_+ |  |  |  |  | 0 | 1 |
| HAM/TSP#1 | 2711 | chr19_56826131_- |  |  |  |  | 0 | 2 |
| HAM/TSP#1 | 2712 | chr19_56975468_+ |  |  |  |  | 0 | 1 |
| HAM/TSP#1 | 2713 | chr19_57525737_+ |  |  |  |  | 0 | 1 |
| HAM/TSP#1 | 2714 | chr19_5944847_- |  |  |  |  | 0 | 1 |
| HAM/TSP#1 | 2715 | chr19_7849984_- |  |  |  |  | 0 | 1 |
| HAM/TSP#1 | 2716 | chr19_8646312_+ |  |  |  |  | 0 | 1 |
| HAM/TSP#1 | 2717 | chr19_928513_- |  |  |  |  | 0 | 1 |
| HAM/TSP#1 | 2718 | chr19_9872249_- |  |  |  |  | 0 | 1 |
| HAM/TSP#1 | 2719 | chr20_10044547_+ |  |  |  |  | 0 | 2 |
| HAM/TSP#1 | 2720 | chr20_11573067_+ |  |  |  |  | 0 | 2 |
| HAM/TSP#1 | 2721 | chr20_11718996_+ |  |  |  |  | 0 | 1 |
| HAM/TSP#1 | 2722 | chr20_11941443_+ |  |  |  |  | 0 | 7 |
| HAM/TSP#1 | 2723 | chr20_1280757_- |  |  |  |  | 0 | 1 |
| HAM/TSP#1 | 2724 | chr20_12950822_- |  |  |  |  | 0 | 6 |
| HAM/TSP#1 | 2725 | chr20_13768294_- |  |  |  |  | 0 | 2 |
| HAM/TSP#1 | 2726 | chr20_15987639_+ |  |  |  |  | 0 | 1 |
| HAM/TSP#1 | 2727 | chr20_18146377_+ |  |  |  |  | 0 | 16 |
| HAM/TSP#1 | 2728 | chr20_18889417_+ |  |  |  |  | 0 | 1 |
| HAM/TSP#1 | 2729 | chr20_18895931_- |  |  |  |  | 0 | 1 |
| HAM/TSP#1 | 2730 | chr20_19466857_- |  |  |  |  | 0 | 1 |
| HAM/TSP#1 | 2731 | chr20_19768581_- |  |  |  |  | 0 | 1 |
| HAM/TSP#1 | 2732 | chr20_20353139_+ |  |  |  |  | 0 | 4 |
| HAM/TSP#1 | 2733 | chr20_20767178_+ |  |  |  |  | 0 | 2 |
| HAM/TSP#1 | 2734 | chr20_21926475_+ |  |  |  |  | 0 | 1 |
| HAM/TSP#1 | 2735 | chr20_22304173_+ |  |  |  |  | 0 | 16 |
| HAM/TSP#1 | 2736 | chr20_22816835_+ |  |  |  |  | 0 | 2 |
| HAM/TSP#1 | 2737 | chr20_2328290_- |  |  |  |  | 0 | 1 |
| HAM/TSP#1 | 2738 | chr20_23486416_- |  |  |  |  | 0 | 1 |
| HAM/TSP#1 | 2739 | chr20_271316_+ |  |  |  |  | 0 | 3 |
| HAM/TSP#1 | 2740 | chr20_2830347_- |  |  |  |  | 0 | 2 |
| HAM/TSP#1 | 2741 | chr20_29358939_+ |  |  |  |  | 0 | 1 |
| HAM/TSP#1 | 2742 | chr20_2979462_- |  |  |  |  | 0 | 1 |
| HAM/TSP#1 | 2743 | chr20_30324611_- |  |  |  |  | 0 | 3 |
| HAM/TSP#1 | 2744 | chr20_30386557_- |  |  |  |  | 0 | 3 |
| HAM/TSP#1 | 2745 | chr20_30725527_- |  |  |  |  | 0 | 1 |
| HAM/TSP#1 | 2746 | chr20_35733706_- |  |  |  |  | 0 | 3 |
| HAM/TSP#1 | 2747 | chr20_35943211_- |  |  |  |  | 0 | 1 |
| HAM/TSP#1 | 2748 | chr20_36816005_+ |  |  |  |  | 0 | 1 |
| HAM/TSP#1 | 2749 | chr20_37034908_+ |  |  |  |  | 0 | 6 |
| HAM/TSP#1 | 2750 | chr20_37143507_+ |  |  |  |  | 0 | 3 |
| HAM/TSP#1 | 2751 | chr20_37411691_- |  |  |  |  | 0 | 1 |
| HAM/TSP#1 | 2752 | chr20_37798218_+ |  |  |  |  | 0 | 1 |
| HAM/TSP#1 | 2753 | chr20_38136300_+ |  |  |  |  | 0 | 1 |
| HAM/TSP#1 | 2754 | chr20_44017340_- |  |  |  |  | 0 | 1 |
| HAM/TSP#1 | 2755 | chr20_44646865_+ |  |  |  |  | 0 | 7 |
| HAM/TSP#1 | 2756 | chr20_44831452_- |  |  |  |  | 0 | 1 |
| HAM/TSP#1 | 2757 | chr20_44961847_+ |  |  |  |  | 0 | 4 |
| HAM/TSP#1 | 2758 | chr20_45217042_- |  |  |  |  | 0 | 1 |
| HAM/TSP#1 | 2759 | chr20_47490709_+ |  |  |  |  | 0 | 25 |
| HAM/TSP#1 | 2760 | chr20_48167880_- |  |  |  |  | 0 | 1 |
| HAM/TSP#1 | 2761 | chr20_48324360_- |  |  |  |  | 0 | 1 |
| HAM/TSP#1 | 2762 | chr20_48597038_- |  |  |  |  | 0 | 1 |
| HAM/TSP#1 | 2763 | chr20_49874737_+ |  |  |  |  | 0 | 14 |
| HAM/TSP#1 | 2764 | chr20_54330155_- |  |  |  |  | 0 | 6 |
| HAM/TSP#1 | 2765 | chr20_55260980_+ |  |  |  |  | 0 | 1 |
| HAM/TSP#1 | 2766 | chr20_56616892_- |  |  |  |  | 0 | 1 |
| HAM/TSP#1 | 2767 | chr20_56761658_+ |  |  |  |  | 0 | 3 |
| HAM/TSP#1 | 2768 | chr20_5711936_- |  |  |  |  | 0 | 7 |
| HAM/TSP#1 | 2769 | chr20_58054810_- |  |  |  |  | 0 | 1 |
| HAM/TSP#1 | 2770 | chr20_58418937_- |  |  |  |  | 0 | 1 |
| HAM/TSP#1 | 2771 | chr20_58784412_- |  |  |  |  | 0 | 1 |
| HAM/TSP#1 | 2772 | chr20_58822867_+ |  |  |  |  | 0 | 1 |
| HAM/TSP#1 | 2773 | chr20_58850073_- |  |  |  |  | 0 | 21 |
| HAM/TSP#1 | 2774 | chr20_59441062_+ |  |  |  |  | 0 | 1 |
| HAM/TSP#1 | 2775 | chr20_5946294_- |  |  |  |  | 0 | 8 |
| HAM/TSP#1 | 2776 | chr20_61343718_+ |  |  |  |  | 0 | 1 |
| HAM/TSP#1 | 2777 | chr20_61467025_+ |  |  |  |  | 0 | 3 |
| HAM/TSP#1 | 2778 | chr20_6158970_- |  |  |  |  | 0 | 1 |
| HAM/TSP#1 | 2779 | chr20_61835671_- |  |  |  |  | 0 | 1 |
| HAM/TSP#1 | 2780 | chr20_61840240_- |  |  |  |  | 0 | 1 |
| HAM/TSP#1 | 2781 | chr20_62133510_+ |  |  |  |  | 0 | 12 |
| HAM/TSP#1 | 2782 | chr20_7159349_- |  |  |  |  | 0 | 1 |
| HAM/TSP#1 | 2783 | chr20_7168658_- |  |  |  |  | 0 | 2 |
| HAM/TSP#1 | 2784 | chr20_7429432_+ |  |  |  |  | 0 | 1 |
| HAM/TSP#1 | 2785 | chr20_8550117_+ |  |  |  |  | 0 | 1 |
| HAM/TSP#1 | 2786 | chr20_8701223_+ |  |  |  |  | 0 | 1 |
| HAM/TSP#1 | 2787 | chr21_10336741_+ |  |  |  |  | 0 | 5 |
| HAM/TSP#1 | 2788 | chr21_10402888_+ |  |  |  |  | 0 | 1 |
| HAM/TSP#1 | 2789 | chr21_13850522_+ |  |  |  |  | 0 | 1 |
| HAM/TSP#1 | 2790 | chr21_13936034_+ |  |  |  |  | 0 | 5 |
| HAM/TSP#1 | 2791 | chr21_14012883_- |  |  |  |  | 0 | 1 |
| HAM/TSP#1 | 2792 | chr21_14462489_+ |  |  |  |  | 0 | 1 |
| HAM/TSP#1 | 2793 | chr21_15714910_- |  |  |  |  | 0 | 2 |
| HAM/TSP#1 | 2794 | chr21_15886506_+ |  |  |  |  | 0 | 5 |
| HAM/TSP#1 | 2795 | chr21_17171731_- |  |  |  |  | 0 | 7 |
| HAM/TSP#1 | 2796 | chr21_17344523_+ |  |  |  |  | 0 | 1 |
| HAM/TSP#1 | 2797 | chr21_18061049_+ |  |  |  |  | 0 | 1 |
| HAM/TSP#1 | 2798 | chr21_19009847_+ |  |  |  |  | 0 | 1 |
| HAM/TSP#1 | 2799 | chr21_20590874_+ |  |  |  |  | 0 | 1 |
| HAM/TSP#1 | 2800 | chr21_21122810_- |  |  |  |  | 0 | 1 |
| HAM/TSP#1 | 2801 | chr21_22715950_- |  |  |  |  | 0 | 4 |
| HAM/TSP#1 | 2802 | chr21_23257621_- |  |  |  |  | 0 | 2 |
| HAM/TSP#1 | 2803 | chr21_23857275_+ |  |  |  |  | 0 | 3 |
| HAM/TSP#1 | 2804 | chr21_24162521_- |  |  |  |  | 0 | 2 |
| HAM/TSP#1 | 2805 | chr21_24747092_+ |  |  |  |  | 0 | 1 |
| HAM/TSP#1 | 2806 | chr21_26216809_- |  |  |  |  | 0 | 1 |
| HAM/TSP#1 | 2807 | chr21_26269403_- |  |  |  |  | 0 | 1 |
| HAM/TSP#1 | 2808 | chr21_27079329_+ |  |  |  |  | 0 | 1 |
| HAM/TSP#1 | 2809 | chr21_27339285_+ |  |  |  |  | 0 | 5 |
| HAM/TSP#1 | 2810 | chr21_28834784_- |  |  |  |  | 0 | 2 |
| HAM/TSP#1 | 2811 | chr21_29716978_+ |  |  |  |  | 0 | 1 |
| HAM/TSP#1 | 2812 | chr21_31919303_+ |  |  |  |  | 0 | 1 |
| HAM/TSP#1 | 2813 | chr21_32264851_+ |  |  |  |  | 0 | 2 |
| HAM/TSP#1 | 2814 | chr21_33372951_- |  |  |  |  | 0 | 1 |
| HAM/TSP#1 | 2815 | chr21_33951976_- |  |  |  |  | 0 | 3 |
| HAM/TSP#1 | 2816 | chr21_34882662_- |  |  |  |  | 0 | 1 |
| HAM/TSP#1 | 2817 | chr21_36485683_+ |  |  |  |  | 0 | 1 |
| HAM/TSP#1 | 2818 | chr21_36572945_- |  |  |  |  | 0 | 1 |
| HAM/TSP#1 | 2819 | chr21_36892750_+ |  |  |  |  | 0 | 9 |
| HAM/TSP#1 | 2820 | chr21_37357892_+ |  |  |  |  | 0 | 10 |
| HAM/TSP#1 | 2821 | chr21_37736877_- |  |  |  |  | 0 | 1 |
| HAM/TSP#1 | 2822 | chr21_38012710_+ |  |  |  |  | 0 | 2 |
| HAM/TSP#1 | 2823 | chr21_38824924_- |  |  |  |  | 0 | 3 |
| HAM/TSP#1 | 2824 | chr21_39622303_+ |  |  |  |  | 0 | 2 |
| HAM/TSP#1 | 2825 | chr21_41136147_+ |  |  |  |  | 0 | 6 |
| HAM/TSP#1 | 2826 | chr21_41640544_- |  |  |  |  | 0 | 1 |
| HAM/TSP#1 | 2827 | chr21_42102611_+ |  |  |  |  | 0 | 1 |
| HAM/TSP#1 | 2828 | chr21_45455743_+ |  |  |  |  | 0 | 1 |
| HAM/TSP#1 | 2829 | chr21_45620321_+ |  |  |  |  | 0 | 1 |
| HAM/TSP#1 | 2830 | chr21_46190149_- |  |  |  |  | 0 | 1 |
| HAM/TSP#1 | 2831 | chr21_5313117_- |  |  |  |  | 6 | 5 |
| HAM/TSP#1 | 2832 | chr21_6359547_+ |  |  |  |  | 0 | 2 |
| HAM/TSP#1 | 2833 | chr21_6371492_+ |  |  |  |  | 0 | 17 |
| HAM/TSP#1 | 2834 | chr21_7955475_+ |  |  |  |  | 0 | 1 |
| HAM/TSP#1 | 2835 | chr21_9010789_+ |  |  |  |  | 0 | 3 |
| HAM/TSP#1 | 2836 | chr21_9024641_- |  |  |  |  | 0 | 1 |
| HAM/TSP#1 | 2837 | chr21_9248585_+ |  |  |  |  | 0 | 5 |
| HAM/TSP#1 | 2838 | chr21_9549038_+ |  |  |  |  | 0 | 3 |
| HAM/TSP#1 | 2839 | chr21_9765832_+ |  |  |  |  | 0 | 5 |
| HAM/TSP#1 | 2840 | chr21_9778889_- |  |  |  |  | 0 | 1 |
| HAM/TSP#1 | 2841 | chr22_10693757_- |  |  |  |  | 0 | 1 |
| HAM/TSP#1 | 2842 | chr22_10696279_+ |  |  |  |  | 0 | 8 |
| HAM/TSP#1 | 2843 | chr22_11570550_+ |  |  |  |  | 0 | 1 |
| HAM/TSP#1 | 2844 | chr22_11587596_+ |  |  |  |  | 0 | 2 |
| HAM/TSP#1 | 2845 | chr22_12054832_+ |  |  |  |  | 0 | 1 |
| HAM/TSP#1 | 2846 | chr22_17169881_- |  |  |  |  | 0 | 5 |
| HAM/TSP#1 | 2847 | chr22_17731644_+ |  |  |  |  | 0 | 1 |
| HAM/TSP#1 | 2848 | chr22_17736013_+ |  |  |  |  | 0 | 5 |
| HAM/TSP#1 | 2849 | chr22_17923652_+ |  |  |  |  | 0 | 1 |
| HAM/TSP#1 | 2850 | chr22_19731784_+ |  |  |  |  | 0 | 4 |
| HAM/TSP#1 | 2851 | chr22_19950047_- |  |  |  |  | 0 | 2 |
| HAM/TSP#1 | 2852 | chr22_20140261_+ |  |  |  |  | 0 | 1 |
| HAM/TSP#1 | 2853 | chr22_20462681_- |  |  |  |  | 0 | 8 |
| HAM/TSP#1 | 2854 | chr22_22443054_+ |  |  |  |  | 0 | 19 |
| HAM/TSP#1 | 2855 | chr22_22646982_+ |  |  |  |  | 0 | 1 |
| HAM/TSP#1 | 2856 | chr22_22734221_+ |  |  |  |  | 0 | 1 |
| HAM/TSP#1 | 2857 | chr22_23033472_- |  |  |  |  | 0 | 1 |
| HAM/TSP#1 | 2858 | chr22_23789926_- |  |  |  |  | 0 | 6 |
| HAM/TSP#1 | 2859 | chr22_25022101_- |  |  |  |  | 0 | 1 |
| HAM/TSP#1 | 2860 | chr22_25594923_+ |  |  |  |  | 0 | 7 |
| HAM/TSP#1 | 2861 | chr22_25823389_+ |  |  |  |  | 0 | 1 |
| HAM/TSP#1 | 2862 | chr22_26808640_- |  |  |  |  | 0 | 1 |
| HAM/TSP#1 | 2863 | chr22_27242454_+ |  |  |  |  | 0 | 1 |
| HAM/TSP#1 | 2864 | chr22_27598848_- |  |  |  |  | 0 | 3 |
| HAM/TSP#1 | 2865 | chr22_30808980_- |  |  |  |  | 0 | 4 |
| HAM/TSP#1 | 2866 | chr22_30951660_+ |  |  |  |  | 0 | 2 |
| HAM/TSP#1 | 2867 | chr22_31588610_+ |  |  |  |  | 0 | 3 |
| HAM/TSP#1 | 2868 | chr22_31618365_+ |  |  |  |  | 0 | 1 |
| HAM/TSP#1 | 2869 | chr22_31825572_+ |  |  |  |  | 0 | 1 |
| HAM/TSP#1 | 2870 | chr22_32073326_+ |  |  |  |  | 0 | 1 |
| HAM/TSP#1 | 2871 | chr22_32790929_- |  |  |  |  | 0 | 1 |
| HAM/TSP#1 | 2872 | chr22_32838032_- |  |  |  |  | 0 | 1 |
| HAM/TSP#1 | 2873 | chr22_32863586_- |  |  |  |  | 0 | 1 |
| HAM/TSP#1 | 2874 | chr22_34639250_+ |  |  |  |  | 0 | 1 |
| HAM/TSP#1 | 2875 | chr22_34712046_+ |  |  |  |  | 0 | 1 |
| HAM/TSP#1 | 2876 | chr22_35316970_+ |  |  |  |  | 0 | 1 |
| HAM/TSP#1 | 2877 | chr22_35347502_+ |  |  |  |  | 0 | 4 |
| HAM/TSP#1 | 2878 | chr22_35666831_- |  |  |  |  | 0 | 1 |
| HAM/TSP#1 | 2879 | chr22_35970501_+ |  |  |  |  | 0 | 5 |
| HAM/TSP#1 | 2880 | chr22_37391837_+ |  |  |  |  | 0 | 1 |
| HAM/TSP#1 | 2881 | chr22_38164464_+ |  |  |  |  | 0 | 2 |
| HAM/TSP#1 | 2882 | chr22_39461547_- |  |  |  |  | 0 | 1 |
| HAM/TSP#1 | 2883 | chr22_39575113_- |  |  |  |  | 0 | 1 |
| HAM/TSP#1 | 2884 | chr22_40096444_+ |  |  |  |  | 0 | 9 |
| HAM/TSP#1 | 2885 | chr22_40096459_+ |  |  |  |  | 0 | 1 |
| HAM/TSP#1 | 2886 | chr22_41227786_+ |  |  |  |  | 0 | 1 |
| HAM/TSP#1 | 2887 | chr22_41361978_- |  |  |  |  | 0 | 1 |
| HAM/TSP#1 | 2888 | chr22_41809586_+ |  |  |  |  | 0 | 9 |
| HAM/TSP#1 | 2889 | chr22_42381844_- |  |  |  |  | 0 | 1 |
| HAM/TSP#1 | 2890 | chr22_43094194_+ |  |  |  |  | 0 | 11 |
| HAM/TSP#1 | 2891 | chr22_43278133_- |  |  |  |  | 0 | 3 |
| HAM/TSP#1 | 2892 | chr22_43473658_+ |  |  |  |  | 0 | 1 |
| HAM/TSP#1 | 2893 | chr22_43535873_+ |  |  |  |  | 0 | 1 |
| HAM/TSP#1 | 2894 | chr22_43946240_+ |  |  |  |  | 0 | 1 |
| HAM/TSP#1 | 2895 | chr22_44307145_- |  |  |  |  | 0 | 1 |
| HAM/TSP#1 | 2896 | chr22_44938900_- |  |  |  |  | 0 | 1 |
| HAM/TSP#1 | 2897 | chr22_45101083_+ |  |  |  |  | 0 | 1 |
| HAM/TSP#1 | 2898 | chr22_45329113_- |  |  |  |  | 0 | 1 |
| HAM/TSP#1 | 2899 | chr22_46032031_+ |  |  |  |  | 0 | 1 |
| HAM/TSP#1 | 2900 | chr22_46792392_+ |  |  |  |  | 0 | 1 |
| HAM/TSP#1 | 2901 | chr22_47195104_+ |  |  |  |  | 0 | 1 |
| HAM/TSP#1 | 2902 | chr22_48821279_+ |  |  |  |  | 0 | 1 |
| HAM/TSP#1 | 2903 | chr22_49102626_- |  |  |  |  | 0 | 1 |
| HAM/TSP#1 | 2904 | chr22_50290084_- |  |  |  |  | 0 | 1 |
| HAM/TSP#1 | 2905 | chrX_100711126_- |  |  |  |  | 0 | 2 |
| HAM/TSP#1 | 2906 | chrX_104196677_+ |  |  |  |  | 0 | 13 |
| HAM/TSP#1 | 2907 | chrX_106577517_+ |  |  |  |  | 0 | 1 |
| HAM/TSP#1 | 2908 | chrX_107141923_- |  |  |  |  | 0 | 4 |
| HAM/TSP#1 | 2909 | chrX_111649001_- |  |  |  |  | 0 | 1 |
| HAM/TSP#1 | 2910 | chrX_113691630_+ |  |  |  |  | 0 | 1 |
| HAM/TSP#1 | 2911 | chrX_114367453_- |  |  |  |  | 0 | 1 |
| HAM/TSP#1 | 2912 | chrX_117607343_- |  |  |  |  | 0 | 1 |
| HAM/TSP#1 | 2913 | chrX_118697435_- |  |  |  |  | 0 | 1 |
| HAM/TSP#1 | 2914 | chrX_12310049_+ |  |  |  |  | 0 | 1 |
| HAM/TSP#1 | 2915 | chrX_125817020_+ |  |  |  |  | 0 | 1 |
| HAM/TSP#1 | 2916 | chrX_129149972_+ |  |  |  |  | 0 | 1 |
| HAM/TSP#1 | 2917 | chrX_129842237_+ |  |  |  |  | 0 | 4 |
| HAM/TSP#1 | 2918 | chrX_130809224_- |  |  |  |  | 0 | 5 |
| HAM/TSP#1 | 2919 | chrX_130881876_- |  |  |  |  | 0 | 5 |
| HAM/TSP#1 | 2920 | chrX_13726877_- |  |  |  |  | 0 | 2 |
| HAM/TSP#1 | 2921 | chrX_13726879_- |  |  |  |  | 0 | 1 |
| HAM/TSP#1 | 2922 | chrX_137920649_- |  |  |  |  | 1 | 0 |
| HAM/TSP#1 | 2923 | chrX_139033605_+ |  |  |  |  | 0 | 1 |
| HAM/TSP#1 | 2924 | chrX_139913912_+ |  |  |  |  | 0 | 1 |
| HAM/TSP#1 | 2925 | chrX_140481505_+ |  |  |  |  | 0 | 1 |
| HAM/TSP#1 | 2926 | chrX_140871938_- |  |  |  |  | 0 | 3 |
| HAM/TSP#1 | 2927 | chrX_141289208_- |  |  |  |  | 0 | 1 |
| HAM/TSP#1 | 2928 | chrX_143705086_+ |  |  |  |  | 0 | 1 |
| HAM/TSP#1 | 2929 | chrX_144044843_- |  |  |  |  | 0 | 1 |
| HAM/TSP#1 | 2930 | chrX_147793632_+ |  |  |  |  | 0 | 1 |
| HAM/TSP#1 | 2931 | chrX_150479966_+ |  |  |  |  | 0 | 8 |
| HAM/TSP#1 | 2932 | chrX_150578055_- |  |  |  |  | 0 | 1 |
| HAM/TSP#1 | 2933 | chrX_153809682_+ |  |  |  |  | 0 | 49 |
| HAM/TSP#1 | 2934 | chrX_153953221_+ |  |  |  |  | 0 | 1 |
| HAM/TSP#1 | 2935 | chrX_16138509_+ |  |  |  |  | 0 | 1 |
| HAM/TSP#1 | 2936 | chrX_19805636_+ |  |  |  |  | 0 | 1 |
| HAM/TSP#1 | 2937 | chrX_20683482_+ |  |  |  |  | 0 | 1 |
| HAM/TSP#1 | 2938 | chrX_21146606_+ |  |  |  |  | 0 | 1 |
| HAM/TSP#1 | 2939 | chrX_21945265_+ |  |  |  |  | 0 | 5 |
| HAM/TSP#1 | 2940 | chrX_22107217_- |  |  |  |  | 0 | 1 |
| HAM/TSP#1 | 2941 | chrX_30185544_+ |  |  |  |  | 0 | 1 |
| HAM/TSP#1 | 2942 | chrX_30527193_+ |  |  |  |  | 0 | 1 |
| HAM/TSP#1 | 2943 | chrX_33525772_- |  |  |  |  | 0 | 1 |
| HAM/TSP#1 | 2944 | chrX_36132733_+ |  |  |  |  | 0 | 1 |
| HAM/TSP#1 | 2945 | chrX_37738192_+ |  |  |  |  | 0 | 1 |
| HAM/TSP#1 | 2946 | chrX_3790154_- |  |  |  |  | 0 | 1 |
| HAM/TSP#1 | 2947 | chrX_40103111_+ |  |  |  |  | 0 | 1 |
| HAM/TSP#1 | 2948 | chrX_4300390_+ |  |  |  |  | 0 | 6 |
| HAM/TSP#1 | 2949 | chrX_44929197_- |  |  |  |  | 0 | 1 |
| HAM/TSP#1 | 2950 | chrX_51133545_- |  |  |  |  | 0 | 1 |
| HAM/TSP#1 | 2951 | chrX_54097459_- |  |  |  |  | 0 | 1 |
| HAM/TSP#1 | 2952 | chrX_57873744_- |  |  |  |  | 0 | 1 |
| HAM/TSP#1 | 2953 | chrX_6276193_+ |  |  |  |  | 0 | 1 |
| HAM/TSP#1 | 2954 | chrX_64214614_- |  |  |  |  | 0 | 4 |
| HAM/TSP#1 | 2955 | chrX_65375372_+ |  |  |  |  | 0 | 2 |
| HAM/TSP#1 | 2956 | chrX_65782328_- |  |  |  |  | 0 | 5 |
| HAM/TSP#1 | 2957 | chrX_68647947_+ |  |  |  |  | 0 | 1 |
| HAM/TSP#1 | 2958 | chrX_68651885_+ |  |  |  |  | 0 | 1 |
| HAM/TSP#1 | 2959 | chrX_69089524_+ |  |  |  |  | 0 | 1 |
| HAM/TSP#1 | 2960 | chrX_70469742_+ |  |  |  |  | 0 | 1 |
| HAM/TSP#1 | 2961 | chrX_7355577_- |  |  |  |  | 0 | 4 |
| HAM/TSP#1 | 2962 | chrX_74288125_- |  |  |  |  | 0 | 1 |
| HAM/TSP#1 | 2963 | chrX_75637665_+ |  |  |  |  | 0 | 1 |
| HAM/TSP#1 | 2964 | chrX_7664603_+ |  |  |  |  | 0 | 1 |
| HAM/TSP#1 | 2965 | chrX_76952380_+ |  |  |  |  | 0 | 1 |
| HAM/TSP#1 | 2966 | chrX_78015886_- |  |  |  |  | 0 | 5 |
| HAM/TSP#1 | 2967 | chrX_82813442_+ |  |  |  |  | 0 | 6 |
| HAM/TSP#1 | 2968 | chrX_83488771_- |  |  |  |  | 0 | 1 |
| HAM/TSP#1 | 2969 | chrX_84187012_- |  |  |  |  | 0 | 1 |
| HAM/TSP#1 | 2970 | chrX_84752238_+ |  |  |  |  | 0 | 1 |
| HAM/TSP#1 | 2971 | chrX_85274271_- |  |  |  |  | 0 | 1 |
| HAM/TSP#1 | 2972 | chrX_85774432_- |  |  |  |  | 0 | 5 |
| HAM/TSP#1 | 2973 | chrX_86035102_+ |  |  |  |  | 0 | 1 |
| HAM/TSP#1 | 2974 | chrX_89976569_- |  |  |  |  | 0 | 1 |
| HAM/TSP#1 | 2975 | chrX_90895490_+ |  |  |  |  | 0 | 11 |
| HAM/TSP#1 | 2976 | chrX_94593127_+ |  |  |  |  | 0 | 1 |
| HAM/TSP#1 | 2977 | chrY_10850554_+ |  |  |  |  | 0 | 1 |
| HAM/TSP#1 | 2978 | chrY_11304949_- |  |  |  |  | 0 | 1 |
| HAM/TSP#1 | 2979 | chrY_11312491_- |  |  |  |  | 0 | 2 |
| HAM/TSP#1 | 2980 | chrY_11329912_+ |  |  |  |  | 0 | 1 |
| HAM/TSP#1 | 2981 | chrY_11334376_- |  |  |  |  | 0 | 1 |
| HAM/TSP#1 | 2982 | chrY_11923451_- |  |  |  |  | 0 | 1 |
| HAM/TSP#1 | 2983 | chrY_12739969_+ |  |  |  |  | 0 | 4 |
| HAM/TSP#1 | 2984 | chrY_13310947_+ |  |  |  |  | 0 | 7 |
| HAM/TSP#1 | 2985 | chrY_16923201_- |  |  |  |  | 0 | 1 |
| HAM/TSP#1 | 2986 | chrY_19515185_- |  |  |  |  | 0 | 1 |
| HAM/TSP#1 | 2987 | chrY_20593043_- |  |  |  |  | 0 | 1 |
| HAM/TSP#1 | 2988 | chrY_56848252_+ |  |  |  |  | 0 | 1 |
| HAM/TSP#1 | 2989 | chrY_8264525_+ |  |  |  |  | 0 | 1 |
| HAM/TSP#2 | 2990 | chr1_100577758_- | 1 | 0 | 0 | 0 | 0 |  |
| HAM/TSP#2 | 2991 | chr1_101422087_+ | 1 | 0 | 0 | 0 | 0 |  |
| HAM/TSP#2 | 2992 | chr1_101454339_- | 0 | 1 | 0 | 0 | 0 |  |
| HAM/TSP#2 | 2993 | chr1_102135788_+ | 1 | 0 | 0 | 0 | 0 |  |
| HAM/TSP#2 | 2994 | chr1_102735223_+ | 1 | 0 | 0 | 0 | 0 |  |
| HAM/TSP#2 | 2995 | chr1_103528389_+ | 1 | 0 | 0 | 0 | 0 |  |
| HAM/TSP#2 | 2996 | chr1_104028022_+ | 2 | 0 | 0 | 0 | 0 |  |
| HAM/TSP#2 | 2997 | chr1_104697878_+ | 1 | 0 | 0 | 0 | 0 |  |
| HAM/TSP#2 | 2998 | chr1_105336945_+ | 2 | 0 | 0 | 0 | 0 |  |
| HAM/TSP#2 | 2999 | chr1_105389165_+ | 2 | 0 | 0 | 0 | 0 |  |
| HAM/TSP#2 | 3000 | chr1_106567537_- | 0 | 0 | 0 | 0 | 1 |  |
| HAM/TSP#2 | 3001 | chr1_106812229_+ | 0 | 3 | 0 | 0 | 0 |  |
| HAM/TSP#2 | 3002 | chr1_107179702_- | 1 | 0 | 0 | 0 | 0 |  |
| HAM/TSP#2 | 3003 | chr1_107338619_- | 2 | 0 | 0 | 0 | 0 |  |
| HAM/TSP#2 | 3004 | chr1_108054932_+ | 1 | 0 | 0 | 0 | 0 |  |
| HAM/TSP#2 | 3005 | chr1_108586415_- | 10 | 0 | 0 | 0 | 0 |  |
| HAM/TSP#2 | 3006 | chr1_10869012_+ | 2 | 0 | 0 | 0 | 0 |  |
| HAM/TSP#2 | 3007 | chr1_108950520_- | 0 | 2 | 0 | 0 | 0 |  |
| HAM/TSP#2 | 3008 | chr1_109490430_+ | 1 | 0 | 0 | 0 | 0 |  |
| HAM/TSP#2 | 3009 | chr1_109826000_+ | 3 | 0 | 0 | 0 | 0 |  |
| HAM/TSP#2 | 3010 | chr1_110214808_+ | 2 | 0 | 0 | 0 | 0 |  |
| HAM/TSP#2 | 3011 | chr1_110735717_+ | 1 | 0 | 0 | 0 | 0 |  |
| HAM/TSP#2 | 3012 | chr1_111704830_- | 3 | 0 | 0 | 0 | 0 |  |
| HAM/TSP#2 | 3013 | chr1_111748291_+ | 1 | 0 | 0 | 0 | 0 |  |
| HAM/TSP#2 | 3014 | chr1_11264501_+ | 2 | 0 | 0 | 0 | 0 |  |
| HAM/TSP#2 | 3015 | chr1_112747853_+ | 1 | 0 | 0 | 0 | 0 |  |
| HAM/TSP#2 | 3016 | chr1_113266264_+ | 0 | 7 | 0 | 0 | 0 |  |
| HAM/TSP#2 | 3017 | chr1_113811542_+ | 9 | 0 | 0 | 0 | 0 |  |
| HAM/TSP#2 | 3018 | chr1_11383942_- | 0 | 2 | 0 | 0 | 0 |  |
| HAM/TSP#2 | 3019 | chr1_113858840_- | 1 | 0 | 0 | 0 | 0 |  |
| HAM/TSP#2 | 3020 | chr1_114108722_+ | 1 | 0 | 0 | 0 | 0 |  |
| HAM/TSP#2 | 3021 | chr1_114832549_- | 14 | 0 | 0 | 0 | 0 |  |
| HAM/TSP#2 | 3022 | chr1_115414914_+ | 4 | 0 | 0 | 0 | 0 |  |
| HAM/TSP#2 | 3023 | chr1_115724835_+ | 0 | 0 | 1 | 0 | 0 |  |
| HAM/TSP#2 | 3024 | chr1_116569789_+ | 1 | 0 | 0 | 0 | 0 |  |
| HAM/TSP#2 | 3025 | chr1_117961723_+ | 0 | 1 | 0 | 0 | 0 |  |
| HAM/TSP#2 | 3026 | chr1_118000735_- | 15 | 0 | 0 | 0 | 0 |  |
| HAM/TSP#2 | 3027 | chr1_118456251_+ | 1 | 0 | 0 | 0 | 0 |  |
| HAM/TSP#2 | 3028 | chr1_118824508_- | 3 | 0 | 0 | 0 | 0 |  |
| HAM/TSP#2 | 3029 | chr1_119358204_+ | 6 | 0 | 0 | 0 | 0 |  |
| HAM/TSP#2 | 3030 | chr1_119415427_- | 0 | 0 | 0 | 0 | 1 |  |
| HAM/TSP#2 | 3031 | chr1_119663577_+ | 10 | 0 | 0 | 0 | 0 |  |
| HAM/TSP#2 | 3032 | chr1_119765753_+ | 1 | 0 | 0 | 0 | 0 |  |
| HAM/TSP#2 | 3033 | chr1_1213742_+ | 1 | 0 | 0 | 0 | 0 |  |
| HAM/TSP#2 | 3034 | chr1_121410825_- | 0 | 0 | 0 | 0 | 1 |  |
| HAM/TSP#2 | 3035 | chr1_121591862_- | 1 | 0 | 0 | 0 | 0 |  |
| HAM/TSP#2 | 3036 | chr1_13959850_+ | 1 | 0 | 0 | 0 | 0 |  |
| HAM/TSP#2 | 3037 | chr1_143710583_+ | 0 | 20 | 0 | 0 | 0 |  |
| HAM/TSP#2 | 3038 | chr1_14518900_- | 1 | 0 | 0 | 0 | 0 |  |
| HAM/TSP#2 | 3039 | chr1_145824929_- | 11 | 0 | 0 | 0 | 0 |  |
| HAM/TSP#2 | 3040 | chr1_146377429_- | 1 | 0 | 0 | 0 | 0 |  |
| HAM/TSP#2 | 3041 | chr1_147063838_+ | 2 | 0 | 0 | 0 | 0 |  |
| HAM/TSP#2 | 3042 | chr1_147632659_+ | 0 | 1 | 0 | 0 | 0 |  |
| HAM/TSP#2 | 3043 | chr1_148494187_- | 8 | 0 | 0 | 0 | 0 |  |
| HAM/TSP#2 | 3044 | chr1_150727676_+ | 1 | 0 | 0 | 0 | 0 |  |
| HAM/TSP#2 | 3045 | chr1_15074087_- | 1 | 0 | 0 | 0 | 0 |  |
| HAM/TSP#2 | 3046 | chr1_151715017_- | 1 | 0 | 0 | 0 | 0 |  |
| HAM/TSP#2 | 3047 | chr1_152383566_+ | 1 | 0 | 0 | 0 | 0 |  |
| HAM/TSP#2 | 3048 | chr1_15279493_- | 0 | 0 | 0 | 0 | 1 |  |
| HAM/TSP#2 | 3049 | chr1_153139735_+ | 1 | 0 | 0 | 0 | 0 |  |
| HAM/TSP#2 | 3050 | chr1_153322064_+ | 2 | 0 | 0 | 0 | 0 |  |
| HAM/TSP#2 | 3051 | chr1_153533652_+ | 3 | 0 | 0 | 0 | 0 |  |
| HAM/TSP#2 | 3052 | chr1_154161438_- | 6 | 0 | 0 | 0 | 0 |  |
| HAM/TSP#2 | 3053 | chr1_154721053_+ | 0 | 0 | 1 | 0 | 0 |  |
| HAM/TSP#2 | 3054 | chr1_155015530_- | 1 | 0 | 0 | 0 | 0 |  |
| HAM/TSP#2 | 3055 | chr1_155077873_+ | 2 | 0 | 0 | 0 | 0 |  |
| HAM/TSP#2 | 3056 | chr1_155591163_+ | 0 | 1 | 0 | 0 | 0 |  |
| HAM/TSP#2 | 3057 | chr1_157835034_- | 2 | 0 | 0 | 0 | 0 |  |
| HAM/TSP#2 | 3058 | chr1_157862654_+ | 1 | 0 | 0 | 0 | 0 |  |
| HAM/TSP#2 | 3059 | chr1_159007631_+ | 1 | 0 | 0 | 0 | 0 |  |
| HAM/TSP#2 | 3060 | chr1_160793669_+ | 3 | 0 | 0 | 0 | 0 |  |
| HAM/TSP#2 | 3061 | chr1_161002671_- | 4 | 0 | 0 | 0 | 0 |  |
| HAM/TSP#2 | 3062 | chr1_161228232_- | 1 | 0 | 0 | 0 | 0 |  |
| HAM/TSP#2 | 3063 | chr1_163923114_+ | 5 | 0 | 0 | 0 | 4 |  |
| HAM/TSP#2 | 3064 | chr1_164442306_- | 1 | 0 | 0 | 0 | 0 |  |
| HAM/TSP#2 | 3065 | chr1_16560826_+ | 1 | 0 | 0 | 0 | 0 |  |
| HAM/TSP#2 | 3066 | chr1_166202684_+ | 1 | 0 | 0 | 0 | 0 |  |
| HAM/TSP#2 | 3067 | chr1_16631357_- | 1 | 0 | 0 | 0 | 0 |  |
| HAM/TSP#2 | 3068 | chr1_166841070_- | 1 | 0 | 0 | 0 | 0 |  |
| HAM/TSP#2 | 3069 | chr1_168197003_- | 1 | 0 | 0 | 0 | 0 |  |
| HAM/TSP#2 | 3070 | chr1_170127990_- | 1 | 0 | 0 | 0 | 0 |  |
| HAM/TSP#2 | 3071 | chr1_171221407_+ | 1 | 0 | 0 | 0 | 0 |  |
| HAM/TSP#2 | 3072 | chr1_171383239_- | 3 | 0 | 0 | 0 | 0 |  |
| HAM/TSP#2 | 3073 | chr1_171649236_+ | 4 | 0 | 0 | 0 | 0 |  |
| HAM/TSP#2 | 3074 | chr1_172840366_+ | 3 | 0 | 0 | 0 | 0 |  |
| HAM/TSP#2 | 3075 | chr1_174108930_+ | 1 | 0 | 0 | 0 | 0 |  |
| HAM/TSP#2 | 3076 | chr1_17505514_- | 0 | 3 | 0 | 0 | 0 |  |
| HAM/TSP#2 | 3077 | chr1_175827595_- | 2 | 0 | 0 | 0 | 0 |  |
| HAM/TSP#2 | 3078 | chr1_17747758_+ | 0 | 0 | 0 | 1 | 0 |  |
| HAM/TSP#2 | 3079 | chr1_179130537_- | 0 | 1 | 0 | 0 | 0 |  |
| HAM/TSP#2 | 3080 | chr1_179227247_- | 2 | 0 | 0 | 0 | 0 |  |
| HAM/TSP#2 | 3081 | chr1_179406342_- | 0 | 1 | 0 | 0 | 0 |  |
| HAM/TSP#2 | 3082 | chr1_179518550_+ | 1 | 0 | 0 | 0 | 0 |  |
| HAM/TSP#2 | 3083 | chr1_179878444_+ | 1 | 0 | 0 | 0 | 0 |  |
| HAM/TSP#2 | 3084 | chr1_180031964_+ | 2 | 0 | 0 | 0 | 0 |  |
| HAM/TSP#2 | 3085 | chr1_181256299_+ | 1 | 0 | 0 | 0 | 0 |  |
| HAM/TSP#2 | 3086 | chr1_182821967_+ | 0 | 0 | 0 | 0 | 1 |  |
| HAM/TSP#2 | 3087 | chr1_183574253_+ | 1 | 0 | 0 | 0 | 0 |  |
| HAM/TSP#2 | 3088 | chr1_183651821_+ | 1 | 0 | 0 | 0 | 0 |  |
| HAM/TSP#2 | 3089 | chr1_188085506_+ | 3 | 0 | 0 | 0 | 0 |  |
| HAM/TSP#2 | 3090 | chr1_188732005_+ | 0 | 0 | 0 | 0 | 1 |  |
| HAM/TSP#2 | 3091 | chr1_189482505_+ | 2 | 0 | 0 | 0 | 0 |  |
| HAM/TSP#2 | 3092 | chr1_189567182_+ | 1 | 0 | 0 | 0 | 0 |  |
| HAM/TSP#2 | 3093 | chr1_190383390_- | 1 | 0 | 0 | 0 | 0 |  |
| HAM/TSP#2 | 3094 | chr1_190416083_+ | 3 | 0 | 0 | 0 | 0 |  |
| HAM/TSP#2 | 3095 | chr1_193430209_- | 2 | 0 | 0 | 0 | 0 |  |
| HAM/TSP#2 | 3096 | chr1_194490160_+ | 1 | 0 | 0 | 0 | 0 |  |
| HAM/TSP#2 | 3097 | chr1_195643383_- | 1 | 0 | 0 | 0 | 0 |  |
| HAM/TSP#2 | 3098 | chr1_200394241_+ | 1 | 0 | 0 | 0 | 0 |  |
| HAM/TSP#2 | 3099 | chr1_200778325_- | 1 | 0 | 0 | 0 | 0 |  |
| HAM/TSP#2 | 3100 | chr1_20185300_- | 4 | 0 | 0 | 0 | 0 |  |
| HAM/TSP#2 | 3101 | chr1_203337971_+ | 1 | 0 | 0 | 0 | 0 |  |
| HAM/TSP#2 | 3102 | chr1_205259979_+ | 1 | 0 | 0 | 0 | 0 |  |
| HAM/TSP#2 | 3103 | chr1_20591213_+ | 1 | 0 | 0 | 0 | 0 |  |
| HAM/TSP#2 | 3104 | chr1_206721188_- | 0 | 9 | 0 | 0 | 0 |  |
| HAM/TSP#2 | 3105 | chr1_207769363_+ | 2 | 0 | 0 | 0 | 0 |  |
| HAM/TSP#2 | 3106 | chr1_20832118_- | 7 | 0 | 0 | 0 | 0 |  |
| HAM/TSP#2 | 3107 | chr1_208590749_- | 0 | 1 | 0 | 0 | 0 |  |
| HAM/TSP#2 | 3108 | chr1_209652315_+ | 1 | 0 | 0 | 0 | 0 |  |
| HAM/TSP#2 | 3109 | chr1_209676999_+ | 4 | 0 | 0 | 0 | 0 |  |
| HAM/TSP#2 | 3110 | chr1_212442705_- | 4 | 0 | 0 | 0 | 0 |  |
| HAM/TSP#2 | 3111 | chr1_212695690_+ | 1 | 0 | 0 | 0 | 0 |  |
| HAM/TSP#2 | 3112 | chr1_213799460_- | 1 | 0 | 0 | 0 | 0 |  |
| HAM/TSP#2 | 3113 | chr1_213810482_+ | 30 | 0 | 0 | 0 | 0 |  |
| HAM/TSP#2 | 3114 | chr1_215993509_- | 0 | 1 | 0 | 0 | 0 |  |
| HAM/TSP#2 | 3115 | chr1_217866996_- | 1 | 0 | 0 | 0 | 0 |  |
| HAM/TSP#2 | 3116 | chr1_217898021_+ | 0 | 7 | 0 | 0 | 0 |  |
| HAM/TSP#2 | 3117 | chr1_218096969_- | 1 | 0 | 0 | 0 | 0 |  |
| HAM/TSP#2 | 3118 | chr1_218220432_+ | 2 | 0 | 0 | 0 | 0 |  |
| HAM/TSP#2 | 3119 | chr1_21911788_+ | 0 | 1 | 0 | 0 | 0 |  |
| HAM/TSP#2 | 3120 | chr1_219177070_+ | 1 | 0 | 0 | 0 | 0 |  |
| HAM/TSP#2 | 3121 | chr1_219808949_+ | 1 | 0 | 0 | 0 | 0 |  |
| HAM/TSP#2 | 3122 | chr1_220222397_+ | 5 | 0 | 0 | 0 | 0 |  |
| HAM/TSP#2 | 3123 | chr1_220728708_+ | 1 | 0 | 0 | 0 | 0 |  |
| HAM/TSP#2 | 3124 | chr1_223243550_- | 1 | 0 | 0 | 0 | 0 |  |
| HAM/TSP#2 | 3125 | chr1_223756419_- | 1 | 0 | 0 | 0 | 0 |  |
| HAM/TSP#2 | 3126 | chr1_224279443_+ | 6 | 2 | 0 | 0 | 1 |  |
| HAM/TSP#2 | 3127 | chr1_224573937_+ | 2 | 0 | 0 | 0 | 0 |  |
| HAM/TSP#2 | 3128 | chr1_225678818_- | 4 | 0 | 0 | 0 | 0 |  |
| HAM/TSP#2 | 3129 | chr1_22608187_- | 0 | 0 | 0 | 0 | 1 |  |
| HAM/TSP#2 | 3130 | chr1_227778957_- | 0 | 24 | 0 | 0 | 0 |  |
| HAM/TSP#2 | 3131 | chr1_229084248_- | 2 | 0 | 0 | 0 | 0 |  |
| HAM/TSP#2 | 3132 | chr1_230477019_+ | 2 | 0 | 0 | 0 | 0 |  |
| HAM/TSP#2 | 3133 | chr1_23097509_- | 1 | 0 | 0 | 0 | 0 |  |
| HAM/TSP#2 | 3134 | chr1_231988309_- | 6 | 0 | 0 | 0 | 0 |  |
| HAM/TSP#2 | 3135 | chr1_233637840_- | 1 | 0 | 0 | 0 | 0 |  |
| HAM/TSP#2 | 3136 | chr1_233718036_+ | 2 | 0 | 0 | 0 | 0 |  |
| HAM/TSP#2 | 3137 | chr1_234700171_- | 0 | 1 | 0 | 0 | 0 |  |
| HAM/TSP#2 | 3138 | chr1_23531834_- | 4 | 0 | 0 | 0 | 0 |  |
| HAM/TSP#2 | 3139 | chr1_235617548_+ | 1 | 0 | 0 | 0 | 0 |  |
| HAM/TSP#2 | 3140 | chr1_235918452_+ | 2 | 0 | 0 | 0 | 0 |  |
| HAM/TSP#2 | 3141 | chr1_235945373_+ | 8 | 0 | 0 | 0 | 0 |  |
| HAM/TSP#2 | 3142 | chr1_236655249_+ | 4 | 0 | 0 | 0 | 0 |  |
| HAM/TSP#2 | 3143 | chr1_239478993_- | 1 | 0 | 0 | 0 | 0 |  |
| HAM/TSP#2 | 3144 | chr1_242227077_- | 1 | 0 | 0 | 0 | 0 |  |
| HAM/TSP#2 | 3145 | chr1_245372469_- | 1 | 0 | 0 | 0 | 0 |  |
| HAM/TSP#2 | 3146 | chr1_246497841_- | 1 | 0 | 0 | 0 | 0 |  |
| HAM/TSP#2 | 3147 | chr1_246914743_- | 27 | 0 | 0 | 0 | 0 |  |
| HAM/TSP#2 | 3148 | chr1_248787368_- | 2 | 0 | 0 | 0 | 0 |  |
| HAM/TSP#2 | 3149 | chr1_248863602_+ | 1 | 0 | 0 | 0 | 0 |  |
| HAM/TSP#2 | 3150 | chr1_2605904_+ | 1 | 0 | 0 | 0 | 0 |  |
| HAM/TSP#2 | 3151 | chr1_27408297_+ | 1 | 0 | 0 | 0 | 0 |  |
| HAM/TSP#2 | 3152 | chr1_27868583_+ | 0 | 1 | 0 | 0 | 0 |  |
| HAM/TSP#2 | 3153 | chr1_27997515_- | 0 | 1 | 0 | 0 | 0 |  |
| HAM/TSP#2 | 3154 | chr1_2857171_- | 0 | 0 | 0 | 1 | 0 |  |
| HAM/TSP#2 | 3155 | chr1_2865288_+ | 1 | 0 | 0 | 0 | 0 |  |
| HAM/TSP#2 | 3156 | chr1_29680775_- | 1 | 0 | 0 | 0 | 0 |  |
| HAM/TSP#2 | 3157 | chr1_29789689_+ | 1 | 0 | 0 | 0 | 0 |  |
| HAM/TSP#2 | 3158 | chr1_29834370_- | 1 | 0 | 0 | 0 | 0 |  |
| HAM/TSP#2 | 3159 | chr1_30325835_- | 1 | 0 | 0 | 0 | 0 |  |
| HAM/TSP#2 | 3160 | chr1_31325688_+ | 1 | 0 | 0 | 0 | 0 |  |
| HAM/TSP#2 | 3161 | chr1_32115742_- | 0 | 3 | 0 | 0 | 0 |  |
| HAM/TSP#2 | 3162 | chr1_32263523_- | 0 | 1 | 0 | 0 | 0 |  |
| HAM/TSP#2 | 3163 | chr1_3353868_- | 1 | 0 | 0 | 0 | 0 |  |
| HAM/TSP#2 | 3164 | chr1_34164472_+ | 1 | 0 | 0 | 0 | 0 |  |
| HAM/TSP#2 | 3165 | chr1_3497730_+ | 4 | 0 | 0 | 0 | 0 |  |
| HAM/TSP#2 | 3166 | chr1_35449685_+ | 1 | 0 | 0 | 0 | 0 |  |
| HAM/TSP#2 | 3167 | chr1_37535095_+ | 1 | 0 | 0 | 0 | 0 |  |
| HAM/TSP#2 | 3168 | chr1_37659891_+ | 0 | 0 | 0 | 0 | 1 |  |
| HAM/TSP#2 | 3169 | chr1_39540696_- | 0 | 50 | 0 | 0 | 0 |  |
| HAM/TSP#2 | 3170 | chr1_39574189_+ | 1 | 0 | 0 | 0 | 0 |  |
| HAM/TSP#2 | 3171 | chr1_39646003_- | 1 | 0 | 0 | 0 | 0 |  |
| HAM/TSP#2 | 3172 | chr1_41695112_+ | 1 | 0 | 0 | 0 | 0 |  |
| HAM/TSP#2 | 3173 | chr1_4255805_- | 1 | 0 | 0 | 0 | 0 |  |
| HAM/TSP#2 | 3174 | chr1_42588402_+ | 1 | 0 | 0 | 0 | 0 |  |
| HAM/TSP#2 | 3175 | chr1_42932371_+ | 1 | 0 | 0 | 0 | 0 |  |
| HAM/TSP#2 | 3176 | chr1_43223421_- | 1 | 0 | 0 | 0 | 0 |  |
| HAM/TSP#2 | 3177 | chr1_44813010_- | 7 | 0 | 0 | 0 | 0 |  |
| HAM/TSP#2 | 3178 | chr1_45533081_- | 2 | 0 | 0 | 0 | 0 |  |
| HAM/TSP#2 | 3179 | chr1_47428273_- | 1 | 0 | 0 | 0 | 0 |  |
| HAM/TSP#2 | 3180 | chr1_47901886_+ | 1 | 0 | 0 | 0 | 0 |  |
| HAM/TSP#2 | 3181 | chr1_49703295_+ | 1 | 0 | 0 | 0 | 0 |  |
| HAM/TSP#2 | 3182 | chr1_56664900_- | 0 | 9 | 0 | 0 | 0 |  |
| HAM/TSP#2 | 3183 | chr1_56792478_+ | 0 | 8 | 0 | 0 | 0 |  |
| HAM/TSP#2 | 3184 | chr1_59783239_+ | 5 | 0 | 0 | 0 | 0 |  |
| HAM/TSP#2 | 3185 | chr1_60648658_+ | 1 | 0 | 0 | 0 | 0 |  |
| HAM/TSP#2 | 3186 | chr1_60864511_- | 2 | 0 | 0 | 0 | 0 |  |
| HAM/TSP#2 | 3187 | chr1_61049000_+ | 1 | 0 | 0 | 0 | 0 |  |
| HAM/TSP#2 | 3188 | chr1_63165491_+ | 0 | 1 | 0 | 0 | 0 |  |
| HAM/TSP#2 | 3189 | chr1_632176_+ | 1 | 0 | 0 | 0 | 0 |  |
| HAM/TSP#2 | 3190 | chr1_63584429_- | 0 | 1 | 0 | 0 | 0 |  |
| HAM/TSP#2 | 3191 | chr1_66185188_+ | 2 | 0 | 0 | 0 | 0 |  |
| HAM/TSP#2 | 3192 | chr1_66212370_+ | 0 | 1 | 0 | 0 | 0 |  |
| HAM/TSP#2 | 3193 | chr1_66222016_+ | 2 | 0 | 0 | 0 | 0 |  |
| HAM/TSP#2 | 3194 | chr1_66760448_- | 1 | 0 | 0 | 0 | 0 |  |
| HAM/TSP#2 | 3195 | chr1_66769390_+ | 1 | 0 | 0 | 0 | 0 |  |
| HAM/TSP#2 | 3196 | chr1_68063309_+ | 1 | 0 | 0 | 0 | 0 |  |
| HAM/TSP#2 | 3197 | chr1_68848968_- | 2 | 0 | 0 | 0 | 0 |  |
| HAM/TSP#2 | 3198 | chr1_69073419_+ | 1 | 0 | 0 | 0 | 0 |  |
| HAM/TSP#2 | 3199 | chr1_69087132_- | 1 | 0 | 0 | 0 | 0 |  |
| HAM/TSP#2 | 3200 | chr1_69312180_- | 1 | 0 | 0 | 0 | 0 |  |
| HAM/TSP#2 | 3201 | chr1_7139959_+ | 0 | 2 | 0 | 0 | 0 |  |
| HAM/TSP#2 | 3202 | chr1_72764481_- | 3 | 0 | 0 | 0 | 0 |  |
| HAM/TSP#2 | 3203 | chr1_73062114_- | 1 | 0 | 0 | 0 | 0 |  |
| HAM/TSP#2 | 3204 | chr1_73561372_- | 1 | 0 | 0 | 0 | 0 |  |
| HAM/TSP#2 | 3205 | chr1_74541910_+ | 4 | 0 | 0 | 0 | 0 |  |
| HAM/TSP#2 | 3206 | chr1_75157608_- | 1 | 0 | 0 | 0 | 0 |  |
| HAM/TSP#2 | 3207 | chr1_75172734_- | 1 | 0 | 0 | 0 | 0 |  |
| HAM/TSP#2 | 3208 | chr1_7621635_- | 1 | 0 | 0 | 0 | 0 |  |
| HAM/TSP#2 | 3209 | chr1_76944462_+ | 3 | 0 | 0 | 0 | 0 |  |
| HAM/TSP#2 | 3210 | chr1_7883625_- | 0 | 0 | 0 | 8 | 0 |  |
| HAM/TSP#2 | 3211 | chr1_80085212_+ | 2 | 0 | 0 | 0 | 0 |  |
| HAM/TSP#2 | 3212 | chr1_80676826_- | 1 | 0 | 0 | 0 | 0 |  |
| HAM/TSP#2 | 3213 | chr1_81153528_- | 1 | 0 | 0 | 0 | 0 |  |
| HAM/TSP#2 | 3214 | chr1_81432914_+ | 4 | 0 | 0 | 0 | 1 |  |
| HAM/TSP#2 | 3215 | chr1_81875752_+ | 0 | 1 | 0 | 0 | 0 |  |
| HAM/TSP#2 | 3216 | chr1_8387788_+ | 1 | 0 | 0 | 0 | 0 |  |
| HAM/TSP#2 | 3217 | chr1_84018661_+ | 4 | 0 | 0 | 0 | 0 |  |
| HAM/TSP#2 | 3218 | chr1_85232281_- | 4 | 0 | 0 | 0 | 0 |  |
| HAM/TSP#2 | 3219 | chr1_86774267_+ | 1 | 0 | 0 | 0 | 0 |  |
| HAM/TSP#2 | 3220 | chr1_87279102_+ | 2 | 0 | 0 | 0 | 0 |  |
| HAM/TSP#2 | 3221 | chr1_88276738_+ | 1 | 0 | 0 | 0 | 0 |  |
| HAM/TSP#2 | 3222 | chr1_90228300_- | 0 | 0 | 0 | 0 | 1 |  |
| HAM/TSP#2 | 3223 | chr1_90529551_- | 0 | 1 | 0 | 0 | 0 |  |
| HAM/TSP#2 | 3224 | chr1_906951_- | 1 | 0 | 0 | 0 | 0 |  |
| HAM/TSP#2 | 3225 | chr1_91547790_+ | 1 | 0 | 0 | 0 | 0 |  |
| HAM/TSP#2 | 3226 | chr1_92585487_- | 0 | 1 | 0 | 0 | 0 |  |
| HAM/TSP#2 | 3227 | chr1_9331601_+ | 3 | 0 | 0 | 0 | 0 |  |
| HAM/TSP#2 | 3228 | chr1_9363751_+ | 5 | 4 | 0 | 0 | 0 |  |
| HAM/TSP#2 | 3229 | chr1_9385408_- | 2 | 0 | 0 | 0 | 0 |  |
| HAM/TSP#2 | 3230 | chr1_93874735_+ | 1 | 0 | 0 | 0 | 0 |  |
| HAM/TSP#2 | 3231 | chr1_9461951_- | 1 | 0 | 0 | 0 | 0 |  |
| HAM/TSP#2 | 3232 | chr1_95089855_- | 1 | 0 | 0 | 0 | 0 |  |
| HAM/TSP#2 | 3233 | chr1_95209358_+ | 1 | 0 | 0 | 0 | 0 |  |
| HAM/TSP#2 | 3234 | chr1_96692854_- | 4 | 0 | 0 | 0 | 0 |  |
| HAM/TSP#2 | 3235 | chr1_9789755_+ | 1 | 0 | 0 | 0 | 0 |  |
| HAM/TSP#2 | 3236 | chr1_99053921_+ | 3 | 0 | 0 | 0 | 0 |  |
| HAM/TSP#2 | 3237 | chr2_100817232_+ | 0 | 0 | 0 | 0 | 4 |  |
| HAM/TSP#2 | 3238 | chr2_100920935_- | 6 | 0 | 0 | 0 | 0 |  |
| HAM/TSP#2 | 3239 | chr2_101060585_- | 2 | 0 | 0 | 0 | 0 |  |
| HAM/TSP#2 | 3240 | chr2_101981020_+ | 1 | 0 | 0 | 0 | 0 |  |
| HAM/TSP#2 | 3241 | chr2_10275042_+ | 1 | 0 | 0 | 0 | 0 |  |
| HAM/TSP#2 | 3242 | chr2_10407873_- | 0 | 8 | 0 | 0 | 0 |  |
| HAM/TSP#2 | 3243 | chr2_10551697_+ | 1 | 0 | 0 | 0 | 0 |  |
| HAM/TSP#2 | 3244 | chr2_106353081_+ | 1 | 0 | 0 | 0 | 0 |  |
| HAM/TSP#2 | 3245 | chr2_107637155_- | 0 | 3 | 0 | 0 | 0 |  |
| HAM/TSP#2 | 3246 | chr2_107750906_- | 1 | 0 | 0 | 0 | 0 |  |
| HAM/TSP#2 | 3247 | chr2_109199416_+ | 1 | 0 | 0 | 0 | 0 |  |
| HAM/TSP#2 | 3248 | chr2_111265153_+ | 0 | 0 | 0 | 0 | 1 |  |
| HAM/TSP#2 | 3249 | chr2_113151166_+ | 0 | 1 | 0 | 0 | 0 |  |
| HAM/TSP#2 | 3250 | chr2_113154593_+ | 13 | 1 | 0 | 0 | 0 |  |
| HAM/TSP#2 | 3251 | chr2_113986367_+ | 0 | 1 | 0 | 0 | 0 |  |
| HAM/TSP#2 | 3252 | chr2_114889023_+ | 4 | 0 | 0 | 0 | 0 |  |
| HAM/TSP#2 | 3253 | chr2_116016709_+ | 3 | 0 | 0 | 0 | 0 |  |
| HAM/TSP#2 | 3254 | chr2_116528186_- | 0 | 1 | 0 | 0 | 0 |  |
| HAM/TSP#2 | 3255 | chr2_11660910_- | 0 | 3 | 0 | 0 | 0 |  |
| HAM/TSP#2 | 3256 | chr2_117680173_- | 1 | 0 | 0 | 0 | 0 |  |
| HAM/TSP#2 | 3257 | chr2_11862761_+ | 2 | 0 | 0 | 0 | 0 |  |
| HAM/TSP#2 | 3258 | chr2_118844368_+ | 1 | 0 | 0 | 0 | 0 |  |
| HAM/TSP#2 | 3259 | chr2_119230761_+ | 1 | 0 | 0 | 0 | 0 |  |
| HAM/TSP#2 | 3260 | chr2_119284249_+ | 1 | 0 | 0 | 0 | 0 |  |
| HAM/TSP#2 | 3261 | chr2_119400799_- | 1 | 0 | 0 | 0 | 0 |  |
| HAM/TSP#2 | 3262 | chr2_1194462_- | 1 | 0 | 0 | 0 | 0 |  |
| HAM/TSP#2 | 3263 | chr2_119821974_- | 1 | 0 | 0 | 0 | 0 |  |
| HAM/TSP#2 | 3264 | chr2_120008087_- | 0 | 1 | 0 | 0 | 0 |  |
| HAM/TSP#2 | 3265 | chr2_124196684_+ | 4 | 0 | 0 | 0 | 0 |  |
| HAM/TSP#2 | 3266 | chr2_124434842_- | 1 | 0 | 0 | 0 | 0 |  |
| HAM/TSP#2 | 3267 | chr2_124989965_+ | 0 | 0 | 6 | 0 | 0 |  |
| HAM/TSP#2 | 3268 | chr2_126826170_+ | 2 | 0 | 0 | 0 | 0 |  |
| HAM/TSP#2 | 3269 | chr2_127202893_+ | 1 | 0 | 0 | 0 | 0 |  |
| HAM/TSP#2 | 3270 | chr2_127859016_- | 1 | 0 | 0 | 0 | 0 |  |
| HAM/TSP#2 | 3271 | chr2_128346063_- | 1 | 0 | 0 | 0 | 0 |  |
| HAM/TSP#2 | 3272 | chr2_128726552_- | 12 | 0 | 0 | 0 | 0 |  |
| HAM/TSP#2 | 3273 | chr2_129130187_+ | 3 | 0 | 0 | 0 | 0 |  |
| HAM/TSP#2 | 3274 | chr2_129964336_- | 1 | 0 | 0 | 0 | 0 |  |
| HAM/TSP#2 | 3275 | chr2_130455_+ | 2 | 0 | 0 | 0 | 0 |  |
| HAM/TSP#2 | 3276 | chr2_130728564_- | 0 | 1 | 0 | 0 | 0 |  |
| HAM/TSP#2 | 3277 | chr2_133017502_- | 2 | 0 | 0 | 0 | 0 |  |
| HAM/TSP#2 | 3278 | chr2_133120382_- | 2 | 0 | 0 | 0 | 0 |  |
| HAM/TSP#2 | 3279 | chr2_13324694_+ | 1 | 0 | 0 | 0 | 0 |  |
| HAM/TSP#2 | 3280 | chr2_133551128_+ | 1 | 0 | 0 | 0 | 0 |  |
| HAM/TSP#2 | 3281 | chr2_134126286_+ | 6 | 0 | 0 | 0 | 0 |  |
| HAM/TSP#2 | 3282 | chr2_136041126_+ | 5 | 0 | 0 | 0 | 0 |  |
| HAM/TSP#2 | 3283 | chr2_136068743_+ | 0 | 1 | 0 | 0 | 0 |  |
| HAM/TSP#2 | 3284 | chr2_139024933_+ | 11 | 0 | 0 | 0 | 0 |  |
| HAM/TSP#2 | 3285 | chr2_139351211_- | 1 | 0 | 0 | 0 | 0 |  |
| HAM/TSP#2 | 3286 | chr2_142841594_- | 1 | 0 | 0 | 0 | 0 |  |
| HAM/TSP#2 | 3287 | chr2_143641093_- | 18 | 1 | 0 | 0 | 0 |  |
| HAM/TSP#2 | 3288 | chr2_145599222_- | 0 | 1 | 0 | 0 | 0 |  |
| HAM/TSP#2 | 3289 | chr2_148390651_- | 1 | 0 | 0 | 0 | 0 |  |
| HAM/TSP#2 | 3290 | chr2_155916203_- | 8 | 0 | 0 | 3 | 0 |  |
| HAM/TSP#2 | 3291 | chr2_155916204_- | 0 | 0 | 0 | 1 | 0 |  |
| HAM/TSP#2 | 3292 | chr2_156933943_+ | 0 | 1 | 0 | 0 | 0 |  |
| HAM/TSP#2 | 3293 | chr2_157058124_+ | 1 | 0 | 0 | 0 | 0 |  |
| HAM/TSP#2 | 3294 | chr2_157268328_+ | 3 | 0 | 0 | 0 | 0 |  |
| HAM/TSP#2 | 3295 | chr2_158476035_+ | 2 | 0 | 0 | 0 | 0 |  |
| HAM/TSP#2 | 3296 | chr2_159609910_+ | 1 | 0 | 0 | 0 | 0 |  |
| HAM/TSP#2 | 3297 | chr2_159796867_+ | 1 | 0 | 0 | 0 | 0 |  |
| HAM/TSP#2 | 3298 | chr2_160405064_- | 1 | 0 | 0 | 0 | 0 |  |
| HAM/TSP#2 | 3299 | chr2_16142888_+ | 1 | 0 | 0 | 0 | 0 |  |
| HAM/TSP#2 | 3300 | chr2_162015104_- | 1 | 0 | 0 | 0 | 0 |  |
| HAM/TSP#2 | 3301 | chr2_164994440_- | 1 | 0 | 0 | 0 | 0 |  |
| HAM/TSP#2 | 3302 | chr2_165510229_+ | 10 | 0 | 0 | 0 | 0 |  |
| HAM/TSP#2 | 3303 | chr2_166628462_+ | 0 | 0 | 0 | 0 | 1 |  |
| HAM/TSP#2 | 3304 | chr2_166846247_- | 3 | 0 | 0 | 0 | 0 |  |
| HAM/TSP#2 | 3305 | chr2_168226144_- | 1 | 0 | 0 | 0 | 0 |  |
| HAM/TSP#2 | 3306 | chr2_168309884_+ | 16 | 0 | 7 | 0 | 5 |  |
| HAM/TSP#2 | 3307 | chr2_174532632_+ | 0 | 0 | 0 | 1 | 0 |  |
| HAM/TSP#2 | 3308 | chr2_175958189_- | 4 | 0 | 0 | 0 | 0 |  |
| HAM/TSP#2 | 3309 | chr2_176462566_- | 1 | 0 | 0 | 0 | 0 |  |
| HAM/TSP#2 | 3310 | chr2_17649794_+ | 12 | 0 | 0 | 0 | 0 |  |
| HAM/TSP#2 | 3311 | chr2_176754918_+ | 1 | 0 | 0 | 0 | 0 |  |
| HAM/TSP#2 | 3312 | chr2_178663704_+ | 25 | 0 | 0 | 0 | 0 |  |
| HAM/TSP#2 | 3313 | chr2_179044800_- | 0 | 3 | 0 | 0 | 0 |  |
| HAM/TSP#2 | 3314 | chr2_180369192_+ | 2 | 0 | 0 | 0 | 0 |  |
| HAM/TSP#2 | 3315 | chr2_184592412_+ | 3 | 0 | 0 | 0 | 0 |  |
| HAM/TSP#2 | 3316 | chr2_187107169_+ | 1 | 0 | 0 | 0 | 0 |  |
| HAM/TSP#2 | 3317 | chr2_18715192_- | 1 | 0 | 0 | 0 | 0 |  |
| HAM/TSP#2 | 3318 | chr2_188744172_- | 2 | 0 | 0 | 0 | 0 |  |
| HAM/TSP#2 | 3319 | chr2_189820623_- | 1 | 0 | 0 | 0 | 0 |  |
| HAM/TSP#2 | 3320 | chr2_191328861_+ | 1 | 0 | 0 | 0 | 0 |  |
| HAM/TSP#2 | 3321 | chr2_192514277_- | 1 | 0 | 0 | 0 | 0 |  |
| HAM/TSP#2 | 3322 | chr2_193201897_- | 1 | 0 | 0 | 0 | 0 |  |
| HAM/TSP#2 | 3323 | chr2_197075926_+ | 1 | 0 | 0 | 0 | 0 |  |
| HAM/TSP#2 | 3324 | chr2_198014994_+ | 0 | 1 | 0 | 0 | 0 |  |
| HAM/TSP#2 | 3325 | chr2_198961621_+ | 7 | 0 | 0 | 0 | 0 |  |
| HAM/TSP#2 | 3326 | chr2_202337317_+ | 0 | 1 | 0 | 0 | 0 |  |
| HAM/TSP#2 | 3327 | chr2_202414027_+ | 2 | 0 | 0 | 0 | 0 |  |
| HAM/TSP#2 | 3328 | chr2_205020516_+ | 3 | 0 | 0 | 0 | 0 |  |
| HAM/TSP#2 | 3329 | chr2_207887782_+ | 0 | 0 | 0 | 2 | 0 |  |
| HAM/TSP#2 | 3330 | chr2_210080004_- | 0 | 1 | 0 | 0 | 0 |  |
| HAM/TSP#2 | 3331 | chr2_212033324_+ | 2 | 0 | 0 | 0 | 0 |  |
| HAM/TSP#2 | 3332 | chr2_212706061_- | 1 | 0 | 0 | 0 | 0 |  |
| HAM/TSP#2 | 3333 | chr2_215211956_- | 1 | 0 | 0 | 0 | 0 |  |
| HAM/TSP#2 | 3334 | chr2_21705390_- | 2 | 0 | 0 | 0 | 0 |  |
| HAM/TSP#2 | 3335 | chr2_217435200_+ | 0 | 1 | 0 | 0 | 0 |  |
| HAM/TSP#2 | 3336 | chr2_217610882_- | 1 | 0 | 0 | 0 | 0 |  |
| HAM/TSP#2 | 3337 | chr2_217865725_- | 1 | 0 | 0 | 0 | 0 |  |
| HAM/TSP#2 | 3338 | chr2_219371226_- | 1 | 0 | 0 | 0 | 0 |  |
| HAM/TSP#2 | 3339 | chr2_220444422_- | 0 | 1 | 0 | 0 | 0 |  |
| HAM/TSP#2 | 3340 | chr2_226334163_- | 3 | 0 | 0 | 0 | 0 |  |
| HAM/TSP#2 | 3341 | chr2_227463057_+ | 1 | 0 | 0 | 0 | 0 |  |
| HAM/TSP#2 | 3342 | chr2_22890268_+ | 1 | 0 | 0 | 0 | 0 |  |
| HAM/TSP#2 | 3343 | chr2_230325219_+ | 0 | 1 | 0 | 0 | 0 |  |
| HAM/TSP#2 | 3344 | chr2_231468573_- | 2 | 0 | 0 | 0 | 0 |  |
| HAM/TSP#2 | 3345 | chr2_232882901_- | 1 | 0 | 0 | 0 | 0 |  |
| HAM/TSP#2 | 3346 | chr2_236025307_- | 1 | 0 | 0 | 0 | 0 |  |
| HAM/TSP#2 | 3347 | chr2_238367235_+ | 0 | 1 | 0 | 0 | 0 |  |
| HAM/TSP#2 | 3348 | chr2_239115093_- | 1 | 0 | 0 | 0 | 0 |  |
| HAM/TSP#2 | 3349 | chr2_239366032_- | 0 | 0 | 0 | 0 | 1 |  |
| HAM/TSP#2 | 3350 | chr2_239659949_- | 0 | 0 | 0 | 1 | 0 |  |
| HAM/TSP#2 | 3351 | chr2_239912022_+ | 1 | 0 | 0 | 0 | 0 |  |
| HAM/TSP#2 | 3352 | chr2_241693032_+ | 1 | 0 | 0 | 0 | 0 |  |
| HAM/TSP#2 | 3353 | chr2_25329746_+ | 1 | 0 | 0 | 0 | 0 |  |
| HAM/TSP#2 | 3354 | chr2_26584110_- | 0 | 1 | 0 | 0 | 0 |  |
| HAM/TSP#2 | 3355 | chr2_29468530_- | 1 | 0 | 0 | 0 | 0 |  |
| HAM/TSP#2 | 3356 | chr2_29737992_+ | 0 | 1 | 0 | 0 | 0 |  |
| HAM/TSP#2 | 3357 | chr2_31603922_- | 0 | 1 | 0 | 0 | 0 |  |
| HAM/TSP#2 | 3358 | chr2_31879689_- | 1 | 0 | 0 | 0 | 0 |  |
| HAM/TSP#2 | 3359 | chr2_32065680_- | 1 | 0 | 0 | 0 | 0 |  |
| HAM/TSP#2 | 3360 | chr2_34685397_- | 1 | 0 | 0 | 0 | 0 |  |
| HAM/TSP#2 | 3361 | chr2_35167395_- | 2 | 0 | 0 | 0 | 0 |  |
| HAM/TSP#2 | 3362 | chr2_35618981_- | 1 | 0 | 0 | 0 | 0 |  |
| HAM/TSP#2 | 3363 | chr2_36279879_- | 3 | 0 | 0 | 0 | 0 |  |
| HAM/TSP#2 | 3364 | chr2_3676379_+ | 1 | 0 | 0 | 0 | 0 |  |
| HAM/TSP#2 | 3365 | chr2_3681812_+ | 1 | 0 | 0 | 0 | 0 |  |
| HAM/TSP#2 | 3366 | chr2_37607681_- | 1 | 0 | 0 | 0 | 0 |  |
| HAM/TSP#2 | 3367 | chr2_37668746_+ | 2 | 0 | 0 | 0 | 0 |  |
| HAM/TSP#2 | 3368 | chr2_37933159_+ | 1 | 0 | 0 | 0 | 0 |  |
| HAM/TSP#2 | 3369 | chr2_39760300_+ | 2 | 0 | 0 | 0 | 0 |  |
| HAM/TSP#2 | 3370 | chr2_42118160_+ | 0 | 3 | 0 | 0 | 0 |  |
| HAM/TSP#2 | 3371 | chr2_42198172_- | 2 | 0 | 0 | 0 | 0 |  |
| HAM/TSP#2 | 3372 | chr2_42664826_- | 1 | 0 | 0 | 0 | 0 |  |
| HAM/TSP#2 | 3373 | chr2_44269945_+ | 3 | 0 | 0 | 0 | 0 |  |
| HAM/TSP#2 | 3374 | chr2_46017913_- | 2 | 0 | 0 | 0 | 0 |  |
| HAM/TSP#2 | 3375 | chr2_47332532_+ | 0 | 1 | 0 | 0 | 0 |  |
| HAM/TSP#2 | 3376 | chr2_4767582_- | 1 | 0 | 0 | 0 | 0 |  |
| HAM/TSP#2 | 3377 | chr2_48315656_- | 0 | 0 | 0 | 0 | 1 |  |
| HAM/TSP#2 | 3378 | chr2_48948139_+ | 1 | 0 | 0 | 0 | 0 |  |
| HAM/TSP#2 | 3379 | chr2_49359376_+ | 0 | 1 | 0 | 0 | 0 |  |
| HAM/TSP#2 | 3380 | chr2_49466194_- | 2 | 0 | 0 | 0 | 0 |  |
| HAM/TSP#2 | 3381 | chr2_49515130_- | 6 | 0 | 0 | 0 | 0 |  |
| HAM/TSP#2 | 3382 | chr2_50705608_- | 2 | 0 | 0 | 0 | 0 |  |
| HAM/TSP#2 | 3383 | chr2_52877609_- | 9 | 0 | 0 | 0 | 0 |  |
| HAM/TSP#2 | 3384 | chr2_53174521_- | 0 | 1 | 0 | 0 | 0 |  |
| HAM/TSP#2 | 3385 | chr2_53842279_- | 1 | 0 | 0 | 0 | 0 |  |
| HAM/TSP#2 | 3386 | chr2_54090990_- | 1 | 0 | 0 | 0 | 0 |  |
| HAM/TSP#2 | 3387 | chr2_54566052_+ | 4 | 0 | 0 | 0 | 0 |  |
| HAM/TSP#2 | 3388 | chr2_54634118_+ | 1 | 0 | 0 | 0 | 0 |  |
| HAM/TSP#2 | 3389 | chr2_55838970_- | 1 | 0 | 0 | 0 | 0 |  |
| HAM/TSP#2 | 3390 | chr2_56448379_- | 3 | 0 | 0 | 0 | 0 |  |
| HAM/TSP#2 | 3391 | chr2_57304769_- | 0 | 1 | 0 | 0 | 0 |  |
| HAM/TSP#2 | 3392 | chr2_57434693_+ | 1 | 0 | 0 | 0 | 0 |  |
| HAM/TSP#2 | 3393 | chr2_59869128_+ | 3 | 0 | 0 | 0 | 0 |  |
| HAM/TSP#2 | 3394 | chr2_59906245_- | 5 | 0 | 0 | 0 | 0 |  |
| HAM/TSP#2 | 3395 | chr2_60631799_+ | 1 | 0 | 0 | 0 | 0 |  |
| HAM/TSP#2 | 3396 | chr2_61295737_- | 1 | 0 | 0 | 0 | 0 |  |
| HAM/TSP#2 | 3397 | chr2_61985675_+ | 0 | 1 | 0 | 0 | 0 |  |
| HAM/TSP#2 | 3398 | chr2_65757975_+ | 2 | 0 | 0 | 0 | 0 |  |
| HAM/TSP#2 | 3399 | chr2_67343726_- | 1 | 0 | 0 | 0 | 0 |  |
| HAM/TSP#2 | 3400 | chr2_6763176_+ | 1 | 0 | 0 | 0 | 0 |  |
| HAM/TSP#2 | 3401 | chr2_67672174_- | 3 | 0 | 0 | 0 | 0 |  |
| HAM/TSP#2 | 3402 | chr2_68386923_- | 5 | 0 | 0 | 0 | 0 |  |
| HAM/TSP#2 | 3403 | chr2_6907635_+ | 1 | 0 | 0 | 0 | 0 |  |
| HAM/TSP#2 | 3404 | chr2_74007882_+ | 1 | 0 | 0 | 0 | 0 |  |
| HAM/TSP#2 | 3405 | chr2_74015186_+ | 1 | 0 | 0 | 0 | 0 |  |
| HAM/TSP#2 | 3406 | chr2_74513737_- | 3 | 0 | 0 | 0 | 0 |  |
| HAM/TSP#2 | 3407 | chr2_74548116_+ | 3 | 0 | 0 | 0 | 0 |  |
| HAM/TSP#2 | 3408 | chr2_75666591_- | 1 | 0 | 0 | 0 | 0 |  |
| HAM/TSP#2 | 3409 | chr2_76138541_+ | 2 | 0 | 0 | 0 | 0 |  |
| HAM/TSP#2 | 3410 | chr2_76914289_+ | 1 | 0 | 0 | 0 | 0 |  |
| HAM/TSP#2 | 3411 | chr2_79373125_+ | 1 | 0 | 0 | 0 | 0 |  |
| HAM/TSP#2 | 3412 | chr2_79868391_+ | 1 | 0 | 0 | 0 | 0 |  |
| HAM/TSP#2 | 3413 | chr2_80112275_- | 1 | 0 | 0 | 0 | 0 |  |
| HAM/TSP#2 | 3414 | chr2_80182524_+ | 2 | 0 | 0 | 0 | 0 |  |
| HAM/TSP#2 | 3415 | chr2_80471984_+ | 25 | 0 | 0 | 0 | 0 |  |
| HAM/TSP#2 | 3416 | chr2_80488246_+ | 1 | 0 | 0 | 0 | 0 |  |
| HAM/TSP#2 | 3417 | chr2_80576167_+ | 2 | 0 | 0 | 0 | 0 |  |
| HAM/TSP#2 | 3418 | chr2_80746420_+ | 1 | 0 | 0 | 0 | 0 |  |
| HAM/TSP#2 | 3419 | chr2_80838850_+ | 2 | 0 | 0 | 0 | 0 |  |
| HAM/TSP#2 | 3420 | chr2_80989317_- | 1 | 0 | 0 | 0 | 0 |  |
| HAM/TSP#2 | 3421 | chr2_81044527_+ | 18 | 0 | 0 | 0 | 0 |  |
| HAM/TSP#2 | 3422 | chr2_81248823_- | 1 | 0 | 0 | 0 | 0 |  |
| HAM/TSP#2 | 3423 | chr2_81604169_+ | 2 | 0 | 0 | 0 | 0 |  |
| HAM/TSP#2 | 3424 | chr2_82496648_- | 3 | 0 | 0 | 0 | 0 |  |
| HAM/TSP#2 | 3425 | chr2_82574911_+ | 4 | 0 | 0 | 0 | 0 |  |
| HAM/TSP#2 | 3426 | chr2_82843720_+ | 5 | 0 | 0 | 0 | 0 |  |
| HAM/TSP#2 | 3427 | chr2_83301041_- | 0 | 1 | 0 | 0 | 0 |  |
| HAM/TSP#2 | 3428 | chr2_84680667_+ | 1 | 0 | 0 | 0 | 0 |  |
| HAM/TSP#2 | 3429 | chr2_84844412_+ | 3 | 0 | 0 | 0 | 0 |  |
| HAM/TSP#2 | 3430 | chr2_8529074_- | 1 | 0 | 0 | 0 | 0 |  |
| HAM/TSP#2 | 3431 | chr2_85916812_+ | 0 | 1 | 0 | 0 | 0 |  |
| HAM/TSP#2 | 3432 | chr2_87283771_- | 12 | 0 | 7 | 0 | 0 |  |
| HAM/TSP#2 | 3433 | chr2_88597697_- | 1 | 0 | 0 | 0 | 0 |  |
| HAM/TSP#2 | 3434 | chr2_88638286_- | 3 | 0 | 0 | 0 | 0 |  |
| HAM/TSP#2 | 3435 | chr2_89609580_- | 1 | 0 | 0 | 0 | 0 |  |
| HAM/TSP#2 | 3436 | chr2_94547857_- | 0 | 0 | 0 | 1 | 0 |  |
| HAM/TSP#2 | 3437 | chr2_94879237_- | 6 | 0 | 0 | 0 | 0 |  |
| HAM/TSP#2 | 3438 | chr2_95089697_+ | 1 | 0 | 0 | 0 | 0 |  |
| HAM/TSP#2 | 3439 | chr2_97682482_- | 8 | 0 | 0 | 0 | 0 |  |
| HAM/TSP#2 | 3440 | chr2_97860541_+ | 1 | 0 | 0 | 0 | 0 |  |
| HAM/TSP#2 | 3441 | chr2_9851190_- | 0 | 15 | 0 | 0 | 0 |  |
| HAM/TSP#2 | 3442 | chr2_9878930_- | 1 | 0 | 0 | 0 | 0 |  |
| HAM/TSP#2 | 3443 | chr2_99218478_- | 2 | 0 | 0 | 0 | 0 |  |
| HAM/TSP#2 | 3444 | chr3_10136343_+ | 1 | 0 | 0 | 0 | 0 |  |
| HAM/TSP#2 | 3445 | chr3_10230465_+ | 2 | 0 | 0 | 0 | 0 |  |
| HAM/TSP#2 | 3446 | chr3_103065727_- | 1 | 0 | 0 | 0 | 0 |  |
| HAM/TSP#2 | 3447 | chr3_103542664_- | 0 | 1 | 0 | 0 | 0 |  |
| HAM/TSP#2 | 3448 | chr3_106162383_+ | 3 | 0 | 0 | 0 | 0 |  |
| HAM/TSP#2 | 3449 | chr3_107130175_+ | 1 | 0 | 0 | 0 | 0 |  |
| HAM/TSP#2 | 3450 | chr3_108100961_- | 7 | 0 | 0 | 0 | 0 |  |
| HAM/TSP#2 | 3451 | chr3_10860526_- | 1 | 0 | 0 | 0 | 0 |  |
| HAM/TSP#2 | 3452 | chr3_108876043_+ | 0 | 1 | 0 | 0 | 0 |  |
| HAM/TSP#2 | 3453 | chr3_110239201_+ | 8 | 0 | 0 | 0 | 0 |  |
| HAM/TSP#2 | 3454 | chr3_110291249_- | 1 | 0 | 0 | 0 | 0 |  |
| HAM/TSP#2 | 3455 | chr3_110355438_+ | 2 | 0 | 0 | 0 | 0 |  |
| HAM/TSP#2 | 3456 | chr3_11163559_- | 0 | 1 | 0 | 0 | 0 |  |
| HAM/TSP#2 | 3457 | chr3_111770345_+ | 1 | 0 | 0 | 0 | 0 |  |
| HAM/TSP#2 | 3458 | chr3_111862111_+ | 1 | 0 | 0 | 0 | 0 |  |
| HAM/TSP#2 | 3459 | chr3_111957820_- | 2 | 1 | 0 | 0 | 0 |  |
| HAM/TSP#2 | 3460 | chr3_111996697_- | 0 | 1 | 0 | 0 | 0 |  |
| HAM/TSP#2 | 3461 | chr3_113130374_- | 3 | 0 | 0 | 0 | 0 |  |
| HAM/TSP#2 | 3462 | chr3_113409501_+ | 1 | 0 | 0 | 0 | 0 |  |
| HAM/TSP#2 | 3463 | chr3_114411397_+ | 1 | 0 | 0 | 0 | 0 |  |
| HAM/TSP#2 | 3464 | chr3_114942506_+ | 1 | 0 | 0 | 0 | 0 |  |
| HAM/TSP#2 | 3465 | chr3_11570367_- | 1 | 0 | 0 | 0 | 0 |  |
| HAM/TSP#2 | 3466 | chr3_116120154_- | 13 | 1 | 0 | 0 | 0 |  |
| HAM/TSP#2 | 3467 | chr3_116350996_- | 0 | 2 | 0 | 0 | 0 |  |
| HAM/TSP#2 | 3468 | chr3_117264325_- | 0 | 14 | 0 | 0 | 0 |  |
| HAM/TSP#2 | 3469 | chr3_117695600_+ | 2 | 0 | 0 | 0 | 0 |  |
| HAM/TSP#2 | 3470 | chr3_11791111_+ | 1 | 0 | 0 | 0 | 0 |  |
| HAM/TSP#2 | 3471 | chr3_118624586_+ | 3 | 0 | 0 | 0 | 0 |  |
| HAM/TSP#2 | 3472 | chr3_119200992_+ | 12 | 0 | 0 | 0 | 0 |  |
| HAM/TSP#2 | 3473 | chr3_119208635_- | 0 | 1 | 0 | 0 | 0 |  |
| HAM/TSP#2 | 3474 | chr3_122539263_+ | 1 | 0 | 0 | 0 | 0 |  |
| HAM/TSP#2 | 3475 | chr3_122881782_- | 10 | 2 | 0 | 0 | 0 |  |
| HAM/TSP#2 | 3476 | chr3_123969823_- | 0 | 1 | 0 | 0 | 0 |  |
| HAM/TSP#2 | 3477 | chr3_124278214_- | 6 | 0 | 0 | 0 | 0 |  |
| HAM/TSP#2 | 3478 | chr3_126081984_+ | 0 | 1 | 0 | 0 | 0 |  |
| HAM/TSP#2 | 3479 | chr3_126717487_+ | 1 | 0 | 0 | 0 | 0 |  |
| HAM/TSP#2 | 3480 | chr3_126941022_+ | 2 | 0 | 0 | 0 | 0 |  |
| HAM/TSP#2 | 3481 | chr3_127027336_- | 1 | 0 | 0 | 0 | 0 |  |
| HAM/TSP#2 | 3482 | chr3_127072720_+ | 1 | 0 | 0 | 0 | 0 |  |
| HAM/TSP#2 | 3483 | chr3_127677443_+ | 4 | 0 | 0 | 0 | 0 |  |
| HAM/TSP#2 | 3484 | chr3_128138577_- | 1 | 0 | 0 | 0 | 0 |  |
| HAM/TSP#2 | 3485 | chr3_128541820_- | 0 | 1 | 0 | 0 | 0 |  |
| HAM/TSP#2 | 3486 | chr3_128782339_+ | 1 | 0 | 0 | 0 | 0 |  |
| HAM/TSP#2 | 3487 | chr3_130306393_- | 1 | 0 | 0 | 0 | 0 |  |
| HAM/TSP#2 | 3488 | chr3_131774427_- | 1 | 0 | 0 | 0 | 0 |  |
| HAM/TSP#2 | 3489 | chr3_133212709_- | 0 | 0 | 0 | 3 | 0 |  |
| HAM/TSP#2 | 3490 | chr3_13407080_- | 3 | 0 | 0 | 0 | 0 |  |
| HAM/TSP#2 | 3491 | chr3_134246632_- | 4 | 0 | 0 | 0 | 0 |  |
| HAM/TSP#2 | 3492 | chr3_135065190_- | 141 | 23 | 19 | 7 | 6 |  |
| HAM/TSP#2 | 3493 | chr3_13679523_+ | 1 | 0 | 0 | 0 | 0 |  |
| HAM/TSP#2 | 3494 | chr3_137536181_- | 3 | 0 | 0 | 0 | 0 |  |
| HAM/TSP#2 | 3495 | chr3_137769008_- | 0 | 1 | 0 | 0 | 0 |  |
| HAM/TSP#2 | 3496 | chr3_139385963_+ | 1 | 0 | 0 | 0 | 0 |  |
| HAM/TSP#2 | 3497 | chr3_141411213_+ | 1 | 0 | 0 | 0 | 0 |  |
| HAM/TSP#2 | 3498 | chr3_141411992_- | 2 | 0 | 0 | 0 | 0 |  |
| HAM/TSP#2 | 3499 | chr3_142905304_+ | 1 | 0 | 0 | 0 | 0 |  |
| HAM/TSP#2 | 3500 | chr3_143009439_+ | 1 | 0 | 0 | 0 | 0 |  |
| HAM/TSP#2 | 3501 | chr3_143301034_- | 1 | 0 | 0 | 0 | 0 |  |
| HAM/TSP#2 | 3502 | chr3_143695986_- | 0 | 1 | 0 | 0 | 0 |  |
| HAM/TSP#2 | 3503 | chr3_144077327_+ | 0 | 4 | 4 | 6 | 7 |  |
| HAM/TSP#2 | 3504 | chr3_14571695_- | 1 | 0 | 0 | 0 | 0 |  |
| HAM/TSP#2 | 3505 | chr3_147364617_- | 4 | 0 | 0 | 0 | 0 |  |
| HAM/TSP#2 | 3506 | chr3_148696661_- | 1 | 0 | 0 | 0 | 0 |  |
| HAM/TSP#2 | 3507 | chr3_149262463_- | 2 | 0 | 0 | 0 | 0 |  |
| HAM/TSP#2 | 3508 | chr3_150285644_- | 1 | 0 | 0 | 0 | 0 |  |
| HAM/TSP#2 | 3509 | chr3_153625633_+ | 1 | 0 | 0 | 0 | 0 |  |
| HAM/TSP#2 | 3510 | chr3_154222449_- | 1 | 0 | 0 | 0 | 0 |  |
| HAM/TSP#2 | 3511 | chr3_155486870_- | 0 | 11 | 0 | 0 | 0 |  |
| HAM/TSP#2 | 3512 | chr3_155894786_+ | 5 | 0 | 0 | 0 | 0 |  |
| HAM/TSP#2 | 3513 | chr3_156237790_+ | 4 | 0 | 0 | 0 | 0 |  |
| HAM/TSP#2 | 3514 | chr3_15641815_- | 1 | 0 | 0 | 0 | 0 |  |
| HAM/TSP#2 | 3515 | chr3_157501801_- | 1 | 0 | 0 | 0 | 0 |  |
| HAM/TSP#2 | 3516 | chr3_158533247_- | 2 | 0 | 0 | 0 | 0 |  |
| HAM/TSP#2 | 3517 | chr3_159837958_+ | 4 | 0 | 0 | 0 | 3 |  |
| HAM/TSP#2 | 3518 | chr3_159930493_+ | 4 | 0 | 0 | 0 | 0 |  |
| HAM/TSP#2 | 3519 | chr3_161129825_- | 7 | 0 | 0 | 0 | 0 |  |
| HAM/TSP#2 | 3520 | chr3_162433192_+ | 1 | 0 | 0 | 0 | 0 |  |
| HAM/TSP#2 | 3521 | chr3_163128944_- | 1 | 0 | 0 | 0 | 0 |  |
| HAM/TSP#2 | 3522 | chr3_163436806_- | 2 | 0 | 0 | 0 | 0 |  |
| HAM/TSP#2 | 3523 | chr3_165203184_+ | 0 | 1 | 0 | 0 | 0 |  |
| HAM/TSP#2 | 3524 | chr3_165473396_+ | 2 | 0 | 0 | 0 | 0 |  |
| HAM/TSP#2 | 3525 | chr3_166550189_+ | 1 | 0 | 0 | 0 | 0 |  |
| HAM/TSP#2 | 3526 | chr3_166849531_- | 2 | 1 | 0 | 0 | 0 |  |
| HAM/TSP#2 | 3527 | chr3_169785309_- | 5 | 0 | 0 | 0 | 0 |  |
| HAM/TSP#2 | 3528 | chr3_171057448_+ | 0 | 0 | 0 | 2 | 0 |  |
| HAM/TSP#2 | 3529 | chr3_17137004_- | 1 | 0 | 0 | 0 | 0 |  |
| HAM/TSP#2 | 3530 | chr3_17302471_- | 2 | 0 | 0 | 0 | 0 |  |
| HAM/TSP#2 | 3531 | chr3_173559294_+ | 4 | 0 | 0 | 0 | 0 |  |
| HAM/TSP#2 | 3532 | chr3_174308224_+ | 1 | 0 | 0 | 0 | 0 |  |
| HAM/TSP#2 | 3533 | chr3_176588020_- | 1 | 0 | 0 | 0 | 0 |  |
| HAM/TSP#2 | 3534 | chr3_177150360_+ | 5 | 0 | 0 | 0 | 0 |  |
| HAM/TSP#2 | 3535 | chr3_177431193_+ | 1 | 0 | 0 | 0 | 0 |  |
| HAM/TSP#2 | 3536 | chr3_178061393_+ | 3 | 0 | 0 | 0 | 0 |  |
| HAM/TSP#2 | 3537 | chr3_178267794_- | 1 | 0 | 0 | 0 | 0 |  |
| HAM/TSP#2 | 3538 | chr3_178267813_- | 1 | 0 | 0 | 0 | 0 |  |
| HAM/TSP#2 | 3539 | chr3_179016812_- | 6 | 0 | 0 | 0 | 0 |  |
| HAM/TSP#2 | 3540 | chr3_180142838_- | 1 | 0 | 0 | 0 | 0 |  |
| HAM/TSP#2 | 3541 | chr3_181589408_+ | 1 | 0 | 0 | 0 | 0 |  |
| HAM/TSP#2 | 3542 | chr3_182475590_+ | 9 | 0 | 0 | 0 | 0 |  |
| HAM/TSP#2 | 3543 | chr3_18273009_+ | 1 | 0 | 0 | 0 | 0 |  |
| HAM/TSP#2 | 3544 | chr3_183973795_- | 1 | 0 | 0 | 0 | 0 |  |
| HAM/TSP#2 | 3545 | chr3_184054579_- | 1 | 0 | 0 | 0 | 0 |  |
| HAM/TSP#2 | 3546 | chr3_184621800_- | 0 | 5 | 0 | 0 | 0 |  |
| HAM/TSP#2 | 3547 | chr3_185072140_- | 1 | 0 | 0 | 0 | 0 |  |
| HAM/TSP#2 | 3548 | chr3_186134516_+ | 0 | 8 | 0 | 0 | 0 |  |
| HAM/TSP#2 | 3549 | chr3_186789017_+ | 1 | 0 | 0 | 0 | 0 |  |
| HAM/TSP#2 | 3550 | chr3_18684802_+ | 2 | 1 | 0 | 0 | 0 |  |
| HAM/TSP#2 | 3551 | chr3_187993323_+ | 1 | 0 | 0 | 0 | 0 |  |
| HAM/TSP#2 | 3552 | chr3_189700445_+ | 2 | 0 | 0 | 0 | 0 |  |
| HAM/TSP#2 | 3553 | chr3_190617700_- | 1 | 0 | 0 | 0 | 0 |  |
| HAM/TSP#2 | 3554 | chr3_191369384_+ | 0 | 1 | 0 | 0 | 0 |  |
| HAM/TSP#2 | 3555 | chr3_192672896_- | 3 | 0 | 0 | 0 | 0 |  |
| HAM/TSP#2 | 3556 | chr3_194071432_+ | 1 | 0 | 0 | 0 | 0 |  |
| HAM/TSP#2 | 3557 | chr3_194819492_- | 1 | 0 | 0 | 0 | 0 |  |
| HAM/TSP#2 | 3558 | chr3_195167580_+ | 2 | 0 | 0 | 0 | 0 |  |
| HAM/TSP#2 | 3559 | chr3_196563906_- | 0 | 1 | 0 | 0 | 0 |  |
| HAM/TSP#2 | 3560 | chr3_196819502_+ | 2 | 0 | 0 | 0 | 0 |  |
| HAM/TSP#2 | 3561 | chr3_197557664_- | 1 | 0 | 0 | 0 | 0 |  |
| HAM/TSP#2 | 3562 | chr3_198109120_+ | 1 | 0 | 0 | 0 | 0 |  |
| HAM/TSP#2 | 3563 | chr3_2070965_- | 2 | 0 | 0 | 0 | 0 |  |
| HAM/TSP#2 | 3564 | chr3_21538348_- | 1 | 0 | 0 | 0 | 0 |  |
| HAM/TSP#2 | 3565 | chr3_23126411_- | 0 | 1 | 0 | 0 | 0 |  |
| HAM/TSP#2 | 3566 | chr3_23595203_- | 5 | 0 | 0 | 0 | 0 |  |
| HAM/TSP#2 | 3567 | chr3_25510059_+ | 0 | 2 | 0 | 0 | 0 |  |
| HAM/TSP#2 | 3568 | chr3_25606581_+ | 3 | 0 | 0 | 0 | 0 |  |
| HAM/TSP#2 | 3569 | chr3_27899027_+ | 0 | 1 | 0 | 0 | 0 |  |
| HAM/TSP#2 | 3570 | chr3_28646489_+ | 4 | 0 | 0 | 0 | 0 |  |
| HAM/TSP#2 | 3571 | chr3_31989990_+ | 1 | 0 | 0 | 0 | 0 |  |
| HAM/TSP#2 | 3572 | chr3_35890648_- | 1 | 0 | 0 | 0 | 0 |  |
| HAM/TSP#2 | 3573 | chr3_36123761_+ | 1 | 0 | 0 | 0 | 0 |  |
| HAM/TSP#2 | 3574 | chr3_36949608_+ | 1 | 0 | 0 | 0 | 0 |  |
| HAM/TSP#2 | 3575 | chr3_3702341_- | 4 | 0 | 0 | 0 | 0 |  |
| HAM/TSP#2 | 3576 | chr3_37138616_- | 1 | 0 | 0 | 0 | 0 |  |
| HAM/TSP#2 | 3577 | chr3_37406426_+ | 2 | 0 | 0 | 0 | 0 |  |
| HAM/TSP#2 | 3578 | chr3_38162578_+ | 1 | 0 | 0 | 0 | 0 |  |
| HAM/TSP#2 | 3579 | chr3_38720858_- | 0 | 0 | 1 | 0 | 0 |  |
| HAM/TSP#2 | 3580 | chr3_39329641_- | 1 | 0 | 0 | 0 | 0 |  |
| HAM/TSP#2 | 3581 | chr3_39781163_- | 4 | 0 | 0 | 0 | 0 |  |
| HAM/TSP#2 | 3582 | chr3_40835291_+ | 1 | 0 | 0 | 0 | 0 |  |
| HAM/TSP#2 | 3583 | chr3_41331069_- | 3 | 0 | 0 | 0 | 0 |  |
| HAM/TSP#2 | 3584 | chr3_42154533_+ | 1 | 0 | 0 | 0 | 0 |  |
| HAM/TSP#2 | 3585 | chr3_43004883_+ | 2 | 0 | 0 | 0 | 0 |  |
| HAM/TSP#2 | 3586 | chr3_44290572_+ | 1 | 0 | 0 | 0 | 0 |  |
| HAM/TSP#2 | 3587 | chr3_45314770_+ | 1 | 0 | 0 | 0 | 0 |  |
| HAM/TSP#2 | 3588 | chr3_45386726_- | 5 | 0 | 0 | 0 | 0 |  |
| HAM/TSP#2 | 3589 | chr3_46406863_+ | 0 | 1 | 0 | 0 | 0 |  |
| HAM/TSP#2 | 3590 | chr3_47138970_+ | 1 | 0 | 0 | 0 | 0 |  |
| HAM/TSP#2 | 3591 | chr3_4748622_+ | 1 | 0 | 0 | 0 | 0 |  |
| HAM/TSP#2 | 3592 | chr3_49813049_+ | 1 | 0 | 0 | 0 | 0 |  |
| HAM/TSP#2 | 3593 | chr3_50073611_- | 1 | 0 | 0 | 0 | 0 |  |
| HAM/TSP#2 | 3594 | chr3_50212580_+ | 1 | 0 | 0 | 0 | 0 |  |
| HAM/TSP#2 | 3595 | chr3_5037414_+ | 1 | 0 | 0 | 0 | 0 |  |
| HAM/TSP#2 | 3596 | chr3_50474994_+ | 1 | 0 | 0 | 0 | 0 |  |
| HAM/TSP#2 | 3597 | chr3_5085253_+ | 3 | 0 | 0 | 0 | 0 |  |
| HAM/TSP#2 | 3598 | chr3_5133657_+ | 0 | 19 | 0 | 0 | 0 |  |
| HAM/TSP#2 | 3599 | chr3_52121265_- | 1 | 0 | 0 | 0 | 0 |  |
| HAM/TSP#2 | 3600 | chr3_52817448_+ | 1 | 0 | 0 | 0 | 0 |  |
| HAM/TSP#2 | 3601 | chr3_5339732_- | 1 | 0 | 0 | 0 | 0 |  |
| HAM/TSP#2 | 3602 | chr3_54490900_+ | 1 | 0 | 0 | 0 | 0 |  |
| HAM/TSP#2 | 3603 | chr3_54564287_+ | 5 | 0 | 0 | 0 | 0 |  |
| HAM/TSP#2 | 3604 | chr3_54575065_- | 1 | 0 | 0 | 0 | 0 |  |
| HAM/TSP#2 | 3605 | chr3_54625691_- | 1 | 0 | 0 | 0 | 0 |  |
| HAM/TSP#2 | 3606 | chr3_55132613_- | 1 | 0 | 0 | 0 | 0 |  |
| HAM/TSP#2 | 3607 | chr3_56630708_- | 1 | 0 | 0 | 0 | 0 |  |
| HAM/TSP#2 | 3608 | chr3_58947185_+ | 3 | 0 | 0 | 0 | 0 |  |
| HAM/TSP#2 | 3609 | chr3_59313767_+ | 0 | 1 | 0 | 0 | 0 |  |
| HAM/TSP#2 | 3610 | chr3_59934091_+ | 2 | 0 | 0 | 0 | 0 |  |
| HAM/TSP#2 | 3611 | chr3_60009478_- | 1 | 0 | 0 | 0 | 0 |  |
| HAM/TSP#2 | 3612 | chr3_61437374_- | 1 | 0 | 0 | 0 | 0 |  |
| HAM/TSP#2 | 3613 | chr3_61733857_- | 1 | 0 | 0 | 0 | 0 |  |
| HAM/TSP#2 | 3614 | chr3_63223528_- | 2 | 0 | 0 | 0 | 0 |  |
| HAM/TSP#2 | 3615 | chr3_63322269_+ | 11 | 0 | 0 | 0 | 0 |  |
| HAM/TSP#2 | 3616 | chr3_63435217_- | 3 | 0 | 0 | 0 | 0 |  |
| HAM/TSP#2 | 3617 | chr3_63950370_- | 1 | 0 | 0 | 0 | 0 |  |
| HAM/TSP#2 | 3618 | chr3_66485458_+ | 13 | 0 | 0 | 0 | 0 |  |
| HAM/TSP#2 | 3619 | chr3_67130231_+ | 8 | 0 | 0 | 0 | 0 |  |
| HAM/TSP#2 | 3620 | chr3_67639420_- | 10 | 0 | 0 | 0 | 0 |  |
| HAM/TSP#2 | 3621 | chr3_67700737_+ | 5 | 0 | 0 | 0 | 0 |  |
| HAM/TSP#2 | 3622 | chr3_68167659_+ | 8 | 0 | 0 | 0 | 0 |  |
| HAM/TSP#2 | 3623 | chr3_68805463_- | 1 | 0 | 0 | 0 | 0 |  |
| HAM/TSP#2 | 3624 | chr3_69437281_+ | 1 | 0 | 0 | 0 | 0 |  |
| HAM/TSP#2 | 3625 | chr3_6957848_- | 5 | 0 | 0 | 0 | 0 |  |
| HAM/TSP#2 | 3626 | chr3_69798145_- | 1 | 0 | 0 | 0 | 0 |  |
| HAM/TSP#2 | 3627 | chr3_69952045_- | 2 | 0 | 0 | 0 | 0 |  |
| HAM/TSP#2 | 3628 | chr3_73159304_- | 1 | 0 | 0 | 0 | 0 |  |
| HAM/TSP#2 | 3629 | chr3_74019316_+ | 1 | 0 | 0 | 0 | 0 |  |
| HAM/TSP#2 | 3630 | chr3_7459210_- | 1 | 0 | 0 | 0 | 0 |  |
| HAM/TSP#2 | 3631 | chr3_74738723_+ | 8 | 0 | 0 | 0 | 0 |  |
| HAM/TSP#2 | 3632 | chr3_74883653_+ | 1 | 0 | 0 | 0 | 0 |  |
| HAM/TSP#2 | 3633 | chr3_75395451_- | 1 | 0 | 0 | 0 | 0 |  |
| HAM/TSP#2 | 3634 | chr3_75406549_+ | 1 | 0 | 0 | 0 | 0 |  |
| HAM/TSP#2 | 3635 | chr3_75755090_- | 0 | 0 | 7 | 0 | 0 |  |
| HAM/TSP#2 | 3636 | chr3_76180440_+ | 4 | 0 | 0 | 0 | 0 |  |
| HAM/TSP#2 | 3637 | chr3_76500491_+ | 1 | 0 | 0 | 0 | 0 |  |
| HAM/TSP#2 | 3638 | chr3_76703170_- | 0 | 1 | 0 | 0 | 0 |  |
| HAM/TSP#2 | 3639 | chr3_77184234_- | 3 | 0 | 0 | 0 | 0 |  |
| HAM/TSP#2 | 3640 | chr3_77280015_- | 2 | 0 | 0 | 0 | 0 |  |
| HAM/TSP#2 | 3641 | chr3_77495596_+ | 2 | 0 | 0 | 0 | 0 |  |
| HAM/TSP#2 | 3642 | chr3_77674499_+ | 1 | 0 | 0 | 0 | 0 |  |
| HAM/TSP#2 | 3643 | chr3_78291580_+ | 2 | 0 | 0 | 0 | 0 |  |
| HAM/TSP#2 | 3644 | chr3_78541660_+ | 1 | 0 | 0 | 0 | 0 |  |
| HAM/TSP#2 | 3645 | chr3_81044304_+ | 1 | 0 | 0 | 0 | 0 |  |
| HAM/TSP#2 | 3646 | chr3_81138825_+ | 2 | 0 | 0 | 0 | 0 |  |
| HAM/TSP#2 | 3647 | chr3_81252002_+ | 10 | 0 | 0 | 0 | 0 |  |
| HAM/TSP#2 | 3648 | chr3_81604124_- | 2 | 0 | 0 | 0 | 0 |  |
| HAM/TSP#2 | 3649 | chr3_81800937_- | 1 | 0 | 0 | 0 | 0 |  |
| HAM/TSP#2 | 3650 | chr3_81890340_- | 3 | 0 | 0 | 0 | 0 |  |
| HAM/TSP#2 | 3651 | chr3_83124196_+ | 3 | 0 | 0 | 0 | 0 |  |
| HAM/TSP#2 | 3652 | chr3_84602877_- | 1 | 0 | 0 | 0 | 0 |  |
| HAM/TSP#2 | 3653 | chr3_8499418_- | 1 | 0 | 0 | 0 | 0 |  |
| HAM/TSP#2 | 3654 | chr3_85694806_- | 2 | 0 | 0 | 0 | 0 |  |
| HAM/TSP#2 | 3655 | chr3_86381741_+ | 1 | 0 | 0 | 0 | 0 |  |
| HAM/TSP#2 | 3656 | chr3_86632787_+ | 2 | 0 | 0 | 0 | 0 |  |
| HAM/TSP#2 | 3657 | chr3_87533441_+ | 1 | 0 | 0 | 0 | 0 |  |
| HAM/TSP#2 | 3658 | chr3_87908275_- | 1 | 0 | 0 | 0 | 0 |  |
| HAM/TSP#2 | 3659 | chr3_88427263_+ | 5 | 0 | 0 | 0 | 0 |  |
| HAM/TSP#2 | 3660 | chr3_88778367_- | 1 | 0 | 0 | 0 | 0 |  |
| HAM/TSP#2 | 3661 | chr3_89285973_- | 1 | 0 | 0 | 0 | 0 |  |
| HAM/TSP#2 | 3662 | chr3_89768939_- | 1 | 0 | 0 | 0 | 0 |  |
| HAM/TSP#2 | 3663 | chr3_90465488_+ | 1 | 0 | 0 | 0 | 0 |  |
| HAM/TSP#2 | 3664 | chr3_90482571_- | 1 | 0 | 0 | 0 | 0 |  |
| HAM/TSP#2 | 3665 | chr3_91529980_+ | 0 | 2 | 0 | 0 | 0 |  |
| HAM/TSP#2 | 3666 | chr3_93470489_+ | 4 | 0 | 0 | 0 | 0 |  |
| HAM/TSP#2 | 3667 | chr3_93470732_- | 2 | 0 | 0 | 0 | 0 |  |
| HAM/TSP#2 | 3668 | chr3_93470760_- | 0 | 1 | 0 | 0 | 0 |  |
| HAM/TSP#2 | 3669 | chr3_93970028_+ | 27 | 0 | 1 | 0 | 2 |  |
| HAM/TSP#2 | 3670 | chr3_93996835_+ | 2 | 0 | 0 | 0 | 0 |  |
| HAM/TSP#2 | 3671 | chr3_94107264_+ | 9 | 2 | 0 | 0 | 0 |  |
| HAM/TSP#2 | 3672 | chr3_94306908_+ | 1 | 0 | 0 | 0 | 0 |  |
| HAM/TSP#2 | 3673 | chr3_94539145_+ | 1 | 0 | 0 | 0 | 0 |  |
| HAM/TSP#2 | 3674 | chr3_94773078_- | 0 | 1 | 0 | 0 | 0 |  |
| HAM/TSP#2 | 3675 | chr3_94814256_+ | 6 | 0 | 0 | 0 | 0 |  |
| HAM/TSP#2 | 3676 | chr3_95158990_- | 0 | 1 | 0 | 0 | 0 |  |
| HAM/TSP#2 | 3677 | chr3_95679343_- | 2 | 0 | 0 | 0 | 0 |  |
| HAM/TSP#2 | 3678 | chr3_95944705_- | 1 | 0 | 0 | 0 | 0 |  |
| HAM/TSP#2 | 3679 | chr3_96798403_+ | 1 | 0 | 0 | 0 | 0 |  |
| HAM/TSP#2 | 3680 | chr3_98515084_- | 2 | 0 | 0 | 4 | 0 |  |
| HAM/TSP#2 | 3681 | chr3_98710027_- | 5 | 2 | 0 | 0 | 0 |  |
| HAM/TSP#2 | 3682 | chr3_987858_+ | 0 | 2 | 0 | 0 | 0 |  |
| HAM/TSP#2 | 3683 | chr3_9903159_- | 12 | 0 | 0 | 0 | 0 |  |
| HAM/TSP#2 | 3684 | chr3_99972616_- | 1 | 0 | 0 | 0 | 0 |  |
| HAM/TSP#2 | 3685 | chr4_10119290_+ | 1 | 0 | 0 | 0 | 0 |  |
| HAM/TSP#2 | 3686 | chr4_101817325_- | 2 | 0 | 0 | 0 | 0 |  |
| HAM/TSP#2 | 3687 | chr4_102251181_- | 15 | 0 | 0 | 0 | 0 |  |
| HAM/TSP#2 | 3688 | chr4_102769426_- | 2 | 0 | 0 | 0 | 0 |  |
| HAM/TSP#2 | 3689 | chr4_1036520_- | 1 | 0 | 0 | 0 | 0 |  |
| HAM/TSP#2 | 3690 | chr4_105265480_- | 3 | 0 | 0 | 0 | 0 |  |
| HAM/TSP#2 | 3691 | chr4_10671686_+ | 4 | 0 | 0 | 0 | 0 |  |
| HAM/TSP#2 | 3692 | chr4_107791663_- | 1 | 0 | 0 | 0 | 0 |  |
| HAM/TSP#2 | 3693 | chr4_10930119_+ | 1 | 0 | 0 | 0 | 0 |  |
| HAM/TSP#2 | 3694 | chr4_1105443_- | 1 | 0 | 0 | 0 | 0 |  |
| HAM/TSP#2 | 3695 | chr4_110708350_- | 1 | 0 | 0 | 0 | 0 |  |
| HAM/TSP#2 | 3696 | chr4_112310283_+ | 3 | 0 | 0 | 0 | 0 |  |
| HAM/TSP#2 | 3697 | chr4_113471744_+ | 2 | 0 | 0 | 0 | 0 |  |
| HAM/TSP#2 | 3698 | chr4_114302676_+ | 0 | 13 | 0 | 0 | 0 |  |
| HAM/TSP#2 | 3699 | chr4_11450823_+ | 7 | 0 | 0 | 0 | 0 |  |
| HAM/TSP#2 | 3700 | chr4_115466435_- | 2 | 0 | 0 | 0 | 0 |  |
| HAM/TSP#2 | 3701 | chr4_118320659_+ | 0 | 2 | 0 | 0 | 0 |  |
| HAM/TSP#2 | 3702 | chr4_118849416_+ | 1 | 0 | 0 | 0 | 0 |  |
| HAM/TSP#2 | 3703 | chr4_119091237_+ | 1 | 0 | 0 | 0 | 0 |  |
| HAM/TSP#2 | 3704 | chr4_119292414_- | 3 | 0 | 0 | 0 | 0 |  |
| HAM/TSP#2 | 3705 | chr4_121223590_+ | 5 | 0 | 0 | 0 | 0 |  |
| HAM/TSP#2 | 3706 | chr4_122689864_- | 2 | 0 | 0 | 0 | 0 |  |
| HAM/TSP#2 | 3707 | chr4_122763930_- | 1 | 0 | 0 | 0 | 0 |  |
| HAM/TSP#2 | 3708 | chr4_122932190_+ | 2 | 1 | 0 | 0 | 0 |  |
| HAM/TSP#2 | 3709 | chr4_123462160_- | 1 | 0 | 0 | 0 | 0 |  |
| HAM/TSP#2 | 3710 | chr4_125244633_+ | 1 | 0 | 0 | 0 | 0 |  |
| HAM/TSP#2 | 3711 | chr4_125265254_+ | 1 | 0 | 0 | 0 | 0 |  |
| HAM/TSP#2 | 3712 | chr4_125468096_+ | 1 | 0 | 0 | 0 | 0 |  |
| HAM/TSP#2 | 3713 | chr4_126177815_- | 3 | 0 | 0 | 0 | 0 |  |
| HAM/TSP#2 | 3714 | chr4_127838925_- | 2 | 0 | 0 | 0 | 0 |  |
| HAM/TSP#2 | 3715 | chr4_133645640_+ | 1 | 0 | 0 | 0 | 0 |  |
| HAM/TSP#2 | 3716 | chr4_1349637_- | 0 | 1 | 0 | 0 | 0 |  |
| HAM/TSP#2 | 3717 | chr4_135307450_+ | 2 | 0 | 0 | 0 | 0 |  |
| HAM/TSP#2 | 3718 | chr4_135545385_+ | 4 | 0 | 0 | 0 | 0 |  |
| HAM/TSP#2 | 3719 | chr4_135687431_+ | 0 | 1 | 0 | 0 | 0 |  |
| HAM/TSP#2 | 3720 | chr4_136104957_- | 1 | 0 | 0 | 0 | 0 |  |
| HAM/TSP#2 | 3721 | chr4_136434855_- | 2 | 0 | 0 | 0 | 0 |  |
| HAM/TSP#2 | 3722 | chr4_137493489_+ | 1 | 0 | 0 | 0 | 0 |  |
| HAM/TSP#2 | 3723 | chr4_143405520_- | 1 | 0 | 0 | 0 | 0 |  |
| HAM/TSP#2 | 3724 | chr4_145145059_- | 1 | 0 | 0 | 0 | 0 |  |
| HAM/TSP#2 | 3725 | chr4_145164234_- | 1 | 0 | 0 | 0 | 0 |  |
| HAM/TSP#2 | 3726 | chr4_146627694_- | 2 | 2 | 0 | 0 | 1 |  |
| HAM/TSP#2 | 3727 | chr4_147466221_- | 2 | 0 | 0 | 0 | 0 |  |
| HAM/TSP#2 | 3728 | chr4_1478136_+ | 1 | 0 | 0 | 0 | 0 |  |
| HAM/TSP#2 | 3729 | chr4_148825448_- | 1 | 0 | 0 | 0 | 0 |  |
| HAM/TSP#2 | 3730 | chr4_150356239_+ | 1 | 0 | 0 | 0 | 0 |  |
| HAM/TSP#2 | 3731 | chr4_150637710_- | 1 | 0 | 0 | 0 | 0 |  |
| HAM/TSP#2 | 3732 | chr4_152841421_+ | 1 | 0 | 0 | 0 | 0 |  |
| HAM/TSP#2 | 3733 | chr4_153159451_+ | 1 | 0 | 0 | 0 | 0 |  |
| HAM/TSP#2 | 3734 | chr4_153247057_- | 5 | 0 | 0 | 0 | 0 |  |
| HAM/TSP#2 | 3735 | chr4_154014000_+ | 1 | 0 | 0 | 0 | 0 |  |
| HAM/TSP#2 | 3736 | chr4_154118562_- | 1 | 0 | 0 | 0 | 0 |  |
| HAM/TSP#2 | 3737 | chr4_15418427_- | 1 | 0 | 0 | 0 | 0 |  |
| HAM/TSP#2 | 3738 | chr4_155212306_- | 4 | 0 | 0 | 0 | 0 |  |
| HAM/TSP#2 | 3739 | chr4_155614755_+ | 1 | 0 | 0 | 0 | 0 |  |
| HAM/TSP#2 | 3740 | chr4_157710403_- | 1 | 0 | 0 | 0 | 0 |  |
| HAM/TSP#2 | 3741 | chr4_157800660_- | 1 | 1 | 0 | 0 | 0 |  |
| HAM/TSP#2 | 3742 | chr4_158904152_- | 1 | 0 | 0 | 0 | 0 |  |
| HAM/TSP#2 | 3743 | chr4_159101268_- | 13 | 2 | 0 | 0 | 0 |  |
| HAM/TSP#2 | 3744 | chr4_159129563_+ | 2 | 0 | 0 | 0 | 0 |  |
| HAM/TSP#2 | 3745 | chr4_159736776_+ | 1 | 0 | 0 | 0 | 0 |  |
| HAM/TSP#2 | 3746 | chr4_15993928_+ | 2 | 0 | 0 | 0 | 0 |  |
| HAM/TSP#2 | 3747 | chr4_160310132_- | 12 | 0 | 0 | 0 | 0 |  |
| HAM/TSP#2 | 3748 | chr4_160830946_- | 9 | 0 | 0 | 0 | 0 |  |
| HAM/TSP#2 | 3749 | chr4_161865357_- | 2 | 0 | 0 | 0 | 0 |  |
| HAM/TSP#2 | 3750 | chr4_161913319_+ | 3 | 0 | 0 | 0 | 0 |  |
| HAM/TSP#2 | 3751 | chr4_163070483_- | 0 | 0 | 0 | 3 | 0 |  |
| HAM/TSP#2 | 3752 | chr4_163155415_+ | 0 | 1 | 0 | 0 | 0 |  |
| HAM/TSP#2 | 3753 | chr4_165031850_+ | 1 | 0 | 0 | 0 | 0 |  |
| HAM/TSP#2 | 3754 | chr4_165718038_- | 1 | 3 | 2 | 1 | 3 |  |
| HAM/TSP#2 | 3755 | chr4_165799668_- | 2 | 0 | 0 | 0 | 1 |  |
| HAM/TSP#2 | 3756 | chr4_166211857_+ | 11 | 0 | 0 | 0 | 0 |  |
| HAM/TSP#2 | 3757 | chr4_166309740_- | 1 | 0 | 0 | 0 | 0 |  |
| HAM/TSP#2 | 3758 | chr4_166522222_- | 1 | 0 | 0 | 0 | 0 |  |
| HAM/TSP#2 | 3759 | chr4_166594024_- | 1 | 0 | 0 | 0 | 0 |  |
| HAM/TSP#2 | 3760 | chr4_168145333_- | 0 | 1 | 0 | 0 | 0 |  |
| HAM/TSP#2 | 3761 | chr4_168513161_- | 3 | 0 | 0 | 0 | 0 |  |
| HAM/TSP#2 | 3762 | chr4_168632340_+ | 1 | 0 | 0 | 0 | 0 |  |
| HAM/TSP#2 | 3763 | chr4_169209384_- | 11 | 0 | 0 | 0 | 0 |  |
| HAM/TSP#2 | 3764 | chr4_169809223_- | 0 | 3 | 0 | 0 | 0 |  |
| HAM/TSP#2 | 3765 | chr4_17141445_- | 1 | 0 | 0 | 0 | 0 |  |
| HAM/TSP#2 | 3766 | chr4_171708033_+ | 1 | 0 | 0 | 0 | 0 |  |
| HAM/TSP#2 | 3767 | chr4_17275190_+ | 1 | 0 | 0 | 0 | 0 |  |
| HAM/TSP#2 | 3768 | chr4_173319162_- | 1 | 0 | 0 | 0 | 0 |  |
| HAM/TSP#2 | 3769 | chr4_173335804_- | 7 | 0 | 0 | 0 | 0 |  |
| HAM/TSP#2 | 3770 | chr4_174499416_+ | 1 | 0 | 0 | 0 | 0 |  |
| HAM/TSP#2 | 3771 | chr4_177279319_- | 1 | 0 | 0 | 0 | 0 |  |
| HAM/TSP#2 | 3772 | chr4_17827955_- | 2 | 0 | 0 | 0 | 0 |  |
| HAM/TSP#2 | 3773 | chr4_17827959_- | 22 | 1 | 0 | 0 | 0 |  |
| HAM/TSP#2 | 3774 | chr4_178630767_+ | 3 | 0 | 0 | 0 | 0 |  |
| HAM/TSP#2 | 3775 | chr4_182339265_+ | 1 | 0 | 0 | 0 | 0 |  |
| HAM/TSP#2 | 3776 | chr4_182921191_+ | 3 | 0 | 0 | 0 | 0 |  |
| HAM/TSP#2 | 3777 | chr4_183118224_+ | 9 | 0 | 0 | 0 | 0 |  |
| HAM/TSP#2 | 3778 | chr4_183822608_+ | 1 | 0 | 0 | 0 | 0 |  |
| HAM/TSP#2 | 3779 | chr4_184546504_- | 6 | 0 | 0 | 0 | 0 |  |
| HAM/TSP#2 | 3780 | chr4_184629347_- | 1 | 0 | 0 | 0 | 0 |  |
| HAM/TSP#2 | 3781 | chr4_184629359_- | 5 | 0 | 0 | 0 | 0 |  |
| HAM/TSP#2 | 3782 | chr4_185046342_- | 0 | 2 | 0 | 0 | 0 |  |
| HAM/TSP#2 | 3783 | chr4_185077355_+ | 1 | 0 | 0 | 0 | 0 |  |
| HAM/TSP#2 | 3784 | chr4_185723722_+ | 0 | 2 | 0 | 0 | 0 |  |
| HAM/TSP#2 | 3785 | chr4_185939538_+ | 1 | 0 | 0 | 0 | 0 |  |
| HAM/TSP#2 | 3786 | chr4_186187543_+ | 1 | 0 | 0 | 0 | 0 |  |
| HAM/TSP#2 | 3787 | chr4_186758777_- | 9 | 0 | 0 | 0 | 0 |  |
| HAM/TSP#2 | 3788 | chr4_186784438_+ | 3 | 0 | 0 | 0 | 0 |  |
| HAM/TSP#2 | 3789 | chr4_188178528_+ | 2 | 0 | 0 | 0 | 0 |  |
| HAM/TSP#2 | 3790 | chr4_189246988_- | 1 | 0 | 0 | 0 | 0 |  |
| HAM/TSP#2 | 3791 | chr4_19342650_- | 0 | 1 | 0 | 0 | 0 |  |
| HAM/TSP#2 | 3792 | chr4_19677244_- | 6 | 0 | 0 | 0 | 0 |  |
| HAM/TSP#2 | 3793 | chr4_2107781_- | 1 | 0 | 0 | 0 | 0 |  |
| HAM/TSP#2 | 3794 | chr4_21885573_- | 2 | 0 | 0 | 0 | 0 |  |
| HAM/TSP#2 | 3795 | chr4_21885574_- | 3 | 1 | 0 | 0 | 0 |  |
| HAM/TSP#2 | 3796 | chr4_22440404_+ | 2 | 0 | 0 | 0 | 0 |  |
| HAM/TSP#2 | 3797 | chr4_22737212_+ | 2 | 0 | 0 | 0 | 0 |  |
| HAM/TSP#2 | 3798 | chr4_2275267_+ | 3 | 0 | 0 | 0 | 0 |  |
| HAM/TSP#2 | 3799 | chr4_23605820_- | 4 | 0 | 0 | 0 | 0 |  |
| HAM/TSP#2 | 3800 | chr4_24244060_+ | 1 | 0 | 0 | 0 | 0 |  |
| HAM/TSP#2 | 3801 | chr4_25895898_+ | 1 | 0 | 0 | 0 | 0 |  |
| HAM/TSP#2 | 3802 | chr4_26307796_- | 0 | 2 | 0 | 0 | 0 |  |
| HAM/TSP#2 | 3803 | chr4_26657080_+ | 1 | 0 | 0 | 0 | 0 |  |
| HAM/TSP#2 | 3804 | chr4_26885475_+ | 6 | 0 | 0 | 0 | 0 |  |
| HAM/TSP#2 | 3805 | chr4_26916013_+ | 3 | 0 | 0 | 0 | 0 |  |
| HAM/TSP#2 | 3806 | chr4_27046777_+ | 4 | 0 | 0 | 0 | 0 |  |
| HAM/TSP#2 | 3807 | chr4_27161174_- | 1 | 0 | 0 | 0 | 0 |  |
| HAM/TSP#2 | 3808 | chr4_28883246_- | 1 | 0 | 0 | 0 | 0 |  |
| HAM/TSP#2 | 3809 | chr4_29439846_- | 7 | 0 | 0 | 0 | 0 |  |
| HAM/TSP#2 | 3810 | chr4_29619002_+ | 4 | 0 | 0 | 0 | 0 |  |
| HAM/TSP#2 | 3811 | chr4_29660948_- | 1 | 0 | 0 | 0 | 0 |  |
| HAM/TSP#2 | 3812 | chr4_30737146_- | 7 | 0 | 0 | 0 | 0 |  |
| HAM/TSP#2 | 3813 | chr4_32532501_+ | 2 | 0 | 0 | 0 | 0 |  |
| HAM/TSP#2 | 3814 | chr4_32577581_+ | 1 | 0 | 0 | 0 | 0 |  |
| HAM/TSP#2 | 3815 | chr4_32879498_+ | 1 | 0 | 0 | 0 | 0 |  |
| HAM/TSP#2 | 3816 | chr4_32950635_- | 1 | 0 | 0 | 0 | 0 |  |
| HAM/TSP#2 | 3817 | chr4_32987222_+ | 0 | 0 | 0 | 0 | 1 |  |
| HAM/TSP#2 | 3818 | chr4_33243123_- | 1 | 0 | 0 | 0 | 0 |  |
| HAM/TSP#2 | 3819 | chr4_35158078_+ | 1 | 0 | 0 | 0 | 0 |  |
| HAM/TSP#2 | 3820 | chr4_36295413_+ | 0 | 1 | 0 | 0 | 0 |  |
| HAM/TSP#2 | 3821 | chr4_3678558_+ | 1 | 0 | 0 | 0 | 0 |  |
| HAM/TSP#2 | 3822 | chr4_38329168_- | 2 | 0 | 0 | 0 | 0 |  |
| HAM/TSP#2 | 3823 | chr4_38806235_+ | 3 | 0 | 0 | 0 | 0 |  |
| HAM/TSP#2 | 3824 | chr4_40452025_+ | 1 | 0 | 0 | 0 | 0 |  |
| HAM/TSP#2 | 3825 | chr4_41299136_- | 1 | 0 | 0 | 0 | 0 |  |
| HAM/TSP#2 | 3826 | chr4_43678073_+ | 1 | 0 | 0 | 0 | 0 |  |
| HAM/TSP#2 | 3827 | chr4_44499757_+ | 2 | 0 | 0 | 0 | 0 |  |
| HAM/TSP#2 | 3828 | chr4_44785010_+ | 1 | 0 | 0 | 0 | 0 |  |
| HAM/TSP#2 | 3829 | chr4_45425270_+ | 1 | 0 | 0 | 0 | 0 |  |
| HAM/TSP#2 | 3830 | chr4_46320881_- | 2 | 0 | 0 | 0 | 0 |  |
| HAM/TSP#2 | 3831 | chr4_46658461_- | 1 | 0 | 0 | 0 | 0 |  |
| HAM/TSP#2 | 3832 | chr4_48332376_+ | 0 | 0 | 0 | 0 | 1 |  |
| HAM/TSP#2 | 3833 | chr4_48801399_+ | 0 | 1 | 0 | 0 | 0 |  |
| HAM/TSP#2 | 3834 | chr4_48914307_+ | 0 | 8 | 0 | 0 | 0 |  |
| HAM/TSP#2 | 3835 | chr4_49097681_+ | 1 | 0 | 0 | 0 | 0 |  |
| HAM/TSP#2 | 3836 | chr4_49137155_+ | 0 | 9 | 0 | 0 | 0 |  |
| HAM/TSP#2 | 3837 | chr4_49145710_- | 0 | 0 | 0 | 0 | 2 |  |
| HAM/TSP#2 | 3838 | chr4_49309235_- | 1 | 0 | 0 | 0 | 0 |  |
| HAM/TSP#2 | 3839 | chr4_49562115_- | 0 | 1 | 0 | 0 | 0 |  |
| HAM/TSP#2 | 3840 | chr4_49632610_+ | 0 | 0 | 0 | 1 | 0 |  |
| HAM/TSP#2 | 3841 | chr4_49709789_- | 2 | 0 | 0 | 0 | 0 |  |
| HAM/TSP#2 | 3842 | chr4_49711244_- | 1 | 0 | 0 | 0 | 0 |  |
| HAM/TSP#2 | 3843 | chr4_50325230_+ | 1 | 0 | 0 | 0 | 0 |  |
| HAM/TSP#2 | 3844 | chr4_50332000_- | 0 | 0 | 0 | 0 | 1 |  |
| HAM/TSP#2 | 3845 | chr4_51418854_+ | 0 | 0 | 1 | 0 | 0 |  |
| HAM/TSP#2 | 3846 | chr4_51894303_+ | 0 | 1 | 0 | 0 | 0 |  |
| HAM/TSP#2 | 3847 | chr4_53451632_+ | 2 | 0 | 0 | 0 | 0 |  |
| HAM/TSP#2 | 3848 | chr4_54218554_+ | 1 | 0 | 0 | 0 | 0 |  |
| HAM/TSP#2 | 3849 | chr4_5447032_- | 0 | 2 | 0 | 0 | 0 |  |
| HAM/TSP#2 | 3850 | chr4_55296274_+ | 3 | 0 | 0 | 0 | 0 |  |
| HAM/TSP#2 | 3851 | chr4_55311033_- | 4 | 0 | 0 | 0 | 0 |  |
| HAM/TSP#2 | 3852 | chr4_55702286_- | 1 | 0 | 0 | 0 | 0 |  |
| HAM/TSP#2 | 3853 | chr4_56719236_- | 1 | 0 | 0 | 0 | 0 |  |
| HAM/TSP#2 | 3854 | chr4_57193408_+ | 3 | 0 | 0 | 0 | 0 |  |
| HAM/TSP#2 | 3855 | chr4_57691829_+ | 2 | 0 | 0 | 0 | 0 |  |
| HAM/TSP#2 | 3856 | chr4_59404704_+ | 0 | 0 | 0 | 1 | 0 |  |
| HAM/TSP#2 | 3857 | chr4_61214423_+ | 4 | 0 | 0 | 0 | 0 |  |
| HAM/TSP#2 | 3858 | chr4_61331667_+ | 1 | 0 | 0 | 0 | 0 |  |
| HAM/TSP#2 | 3859 | chr4_62487637_- | 1 | 0 | 0 | 0 | 0 |  |
| HAM/TSP#2 | 3860 | chr4_63417090_+ | 2 | 0 | 0 | 0 | 0 |  |
| HAM/TSP#2 | 3861 | chr4_63630276_- | 3 | 0 | 0 | 0 | 0 |  |
| HAM/TSP#2 | 3862 | chr4_64590214_+ | 1 | 0 | 0 | 0 | 0 |  |
| HAM/TSP#2 | 3863 | chr4_64718206_- | 1 | 0 | 0 | 0 | 0 |  |
| HAM/TSP#2 | 3864 | chr4_65725764_+ | 2 | 0 | 0 | 0 | 0 |  |
| HAM/TSP#2 | 3865 | chr4_66656815_- | 4 | 0 | 0 | 0 | 0 |  |
| HAM/TSP#2 | 3866 | chr4_6707828_+ | 0 | 10 | 0 | 0 | 0 |  |
| HAM/TSP#2 | 3867 | chr4_67302527_- | 3 | 0 | 0 | 0 | 0 |  |
| HAM/TSP#2 | 3868 | chr4_67728873_- | 0 | 11 | 0 | 0 | 0 |  |
| HAM/TSP#2 | 3869 | chr4_68969795_- | 1 | 0 | 0 | 0 | 0 |  |
| HAM/TSP#2 | 3870 | chr4_69175453_- | 1 | 0 | 0 | 0 | 0 |  |
| HAM/TSP#2 | 3871 | chr4_69924803_+ | 0 | 1 | 0 | 0 | 0 |  |
| HAM/TSP#2 | 3872 | chr4_70735472_- | 3 | 1 | 0 | 0 | 0 |  |
| HAM/TSP#2 | 3873 | chr4_71192378_+ | 1 | 0 | 0 | 0 | 0 |  |
| HAM/TSP#2 | 3874 | chr4_72582435_- | 0 | 0 | 0 | 0 | 1 |  |
| HAM/TSP#2 | 3875 | chr4_73087982_- | 3 | 0 | 0 | 0 | 0 |  |
| HAM/TSP#2 | 3876 | chr4_73141770_+ | 2 | 1 | 0 | 0 | 0 |  |
| HAM/TSP#2 | 3877 | chr4_73612798_+ | 3 | 0 | 0 | 0 | 0 |  |
| HAM/TSP#2 | 3878 | chr4_76158722_- | 3 | 0 | 0 | 0 | 0 |  |
| HAM/TSP#2 | 3879 | chr4_76201217_- | 0 | 0 | 0 | 0 | 1 |  |
| HAM/TSP#2 | 3880 | chr4_76282075_+ | 1 | 0 | 0 | 0 | 0 |  |
| HAM/TSP#2 | 3881 | chr4_76891330_+ | 0 | 1 | 0 | 0 | 0 |  |
| HAM/TSP#2 | 3882 | chr4_77431556_+ | 1 | 0 | 0 | 0 | 0 |  |
| HAM/TSP#2 | 3883 | chr4_77586173_- | 2 | 0 | 0 | 0 | 0 |  |
| HAM/TSP#2 | 3884 | chr4_78454947_- | 1 | 0 | 0 | 0 | 0 |  |
| HAM/TSP#2 | 3885 | chr4_78780563_+ | 1 | 0 | 0 | 0 | 0 |  |
| HAM/TSP#2 | 3886 | chr4_7895857_- | 1 | 0 | 0 | 0 | 0 |  |
| HAM/TSP#2 | 3887 | chr4_79129674_+ | 0 | 0 | 0 | 0 | 1 |  |
| HAM/TSP#2 | 3888 | chr4_79668905_- | 4 | 0 | 0 | 0 | 0 |  |
| HAM/TSP#2 | 3889 | chr4_80585737_- | 2 | 0 | 0 | 0 | 0 |  |
| HAM/TSP#2 | 3890 | chr4_80694946_+ | 1 | 0 | 0 | 0 | 0 |  |
| HAM/TSP#2 | 3891 | chr4_81517095_- | 2 | 0 | 0 | 0 | 0 |  |
| HAM/TSP#2 | 3892 | chr4_82294096_+ | 3 | 0 | 0 | 0 | 0 |  |
| HAM/TSP#2 | 3893 | chr4_82429904_+ | 2 | 0 | 0 | 0 | 0 |  |
| HAM/TSP#2 | 3894 | chr4_82593584_+ | 1 | 0 | 0 | 0 | 0 |  |
| HAM/TSP#2 | 3895 | chr4_83010791_- | 1 | 0 | 0 | 0 | 0 |  |
| HAM/TSP#2 | 3896 | chr4_83595863_+ | 1 | 0 | 0 | 0 | 0 |  |
| HAM/TSP#2 | 3897 | chr4_86628452_+ | 1 | 0 | 0 | 0 | 0 |  |
| HAM/TSP#2 | 3898 | chr4_87055494_+ | 1 | 0 | 0 | 0 | 0 |  |
| HAM/TSP#2 | 3899 | chr4_87133761_- | 1 | 0 | 0 | 0 | 0 |  |
| HAM/TSP#2 | 3900 | chr4_87282595_+ | 5 | 0 | 0 | 0 | 0 |  |
| HAM/TSP#2 | 3901 | chr4_8737108_+ | 1 | 0 | 0 | 0 | 0 |  |
| HAM/TSP#2 | 3902 | chr4_88317266_- | 1 | 0 | 0 | 0 | 0 |  |
| HAM/TSP#2 | 3903 | chr4_88807269_- | 0 | 0 | 0 | 1 | 0 |  |
| HAM/TSP#2 | 3904 | chr4_9000397_+ | 5 | 0 | 0 | 0 | 0 |  |
| HAM/TSP#2 | 3905 | chr4_91254067_- | 3 | 0 | 0 | 0 | 0 |  |
| HAM/TSP#2 | 3906 | chr4_91485099_- | 4 | 0 | 0 | 0 | 0 |  |
| HAM/TSP#2 | 3907 | chr4_91618985_- | 3 | 0 | 0 | 0 | 0 |  |
| HAM/TSP#2 | 3908 | chr4_92069690_- | 1 | 0 | 0 | 0 | 0 |  |
| HAM/TSP#2 | 3909 | chr4_94570944_- | 1 | 0 | 0 | 0 | 0 |  |
| HAM/TSP#2 | 3910 | chr4_94752991_+ | 0 | 1 | 0 | 0 | 0 |  |
| HAM/TSP#2 | 3911 | chr4_94951191_- | 1 | 0 | 0 | 0 | 0 |  |
| HAM/TSP#2 | 3912 | chr4_97183605_- | 1 | 0 | 0 | 0 | 0 |  |
| HAM/TSP#2 | 3913 | chr4_9844787_- | 1 | 0 | 0 | 0 | 0 |  |
| HAM/TSP#2 | 3914 | chr4_99834306_+ | 5 | 1 | 0 | 0 | 0 |  |
| HAM/TSP#2 | 3915 | chr5_100445360_+ | 1 | 0 | 0 | 0 | 0 |  |
| HAM/TSP#2 | 3916 | chr5_101284075_- | 1 | 0 | 0 | 0 | 0 |  |
| HAM/TSP#2 | 3917 | chr5_10268939_- | 1 | 0 | 0 | 0 | 0 |  |
| HAM/TSP#2 | 3918 | chr5_102782992_- | 1 | 0 | 0 | 0 | 0 |  |
| HAM/TSP#2 | 3919 | chr5_103637404_+ | 1 | 0 | 0 | 0 | 0 |  |
| HAM/TSP#2 | 3920 | chr5_107716294_- | 2 | 0 | 0 | 0 | 0 |  |
| HAM/TSP#2 | 3921 | chr5_108185360_- | 1 | 0 | 0 | 0 | 0 |  |
| HAM/TSP#2 | 3922 | chr5_108378309_- | 1 | 0 | 0 | 0 | 0 |  |
| HAM/TSP#2 | 3923 | chr5_108689639_- | 4 | 0 | 0 | 0 | 0 |  |
| HAM/TSP#2 | 3924 | chr5_110333157_- | 0 | 2 | 0 | 0 | 0 |  |
| HAM/TSP#2 | 3925 | chr5_112527671_+ | 1 | 0 | 0 | 0 | 0 |  |
| HAM/TSP#2 | 3926 | chr5_113781733_- | 0 | 1 | 0 | 0 | 0 |  |
| HAM/TSP#2 | 3927 | chr5_115619773_+ | 1 | 0 | 0 | 0 | 0 |  |
| HAM/TSP#2 | 3928 | chr5_115753339_+ | 1 | 0 | 0 | 0 | 0 |  |
| HAM/TSP#2 | 3929 | chr5_11590536_- | 1 | 0 | 0 | 0 | 0 |  |
| HAM/TSP#2 | 3930 | chr5_116953048_+ | 2 | 0 | 0 | 0 | 0 |  |
| HAM/TSP#2 | 3931 | chr5_11937033_+ | 1 | 0 | 0 | 0 | 0 |  |
| HAM/TSP#2 | 3932 | chr5_120414558_- | 1 | 0 | 0 | 0 | 0 |  |
| HAM/TSP#2 | 3933 | chr5_12279799_- | 1 | 0 | 0 | 0 | 0 |  |
| HAM/TSP#2 | 3934 | chr5_122945678_- | 0 | 1 | 0 | 0 | 0 |  |
| HAM/TSP#2 | 3935 | chr5_12333569_- | 0 | 1 | 0 | 0 | 0 |  |
| HAM/TSP#2 | 3936 | chr5_124587457_- | 2 | 0 | 0 | 0 | 0 |  |
| HAM/TSP#2 | 3937 | chr5_12467131_- | 1 | 0 | 0 | 0 | 0 |  |
| HAM/TSP#2 | 3938 | chr5_124844057_- | 4 | 0 | 0 | 0 | 0 |  |
| HAM/TSP#2 | 3939 | chr5_126777188_- | 1 | 0 | 0 | 0 | 0 |  |
| HAM/TSP#2 | 3940 | chr5_127297555_- | 1 | 0 | 0 | 0 | 0 |  |
| HAM/TSP#2 | 3941 | chr5_131381023_- | 1 | 0 | 0 | 0 | 0 |  |
| HAM/TSP#2 | 3942 | chr5_131862950_+ | 0 | 1 | 0 | 0 | 0 |  |
| HAM/TSP#2 | 3943 | chr5_133062359_- | 1 | 0 | 0 | 0 | 0 |  |
| HAM/TSP#2 | 3944 | chr5_133355431_- | 1 | 0 | 0 | 0 | 0 |  |
| HAM/TSP#2 | 3945 | chr5_133674467_+ | 1 | 0 | 0 | 0 | 0 |  |
| HAM/TSP#2 | 3946 | chr5_135022494_- | 1 | 0 | 0 | 3 | 4 |  |
| HAM/TSP#2 | 3947 | chr5_135550630_- | 1 | 0 | 0 | 0 | 0 |  |
| HAM/TSP#2 | 3948 | chr5_135656278_+ | 4 | 0 | 0 | 0 | 0 |  |
| HAM/TSP#2 | 3949 | chr5_136262393_+ | 1 | 0 | 0 | 0 | 0 |  |
| HAM/TSP#2 | 3950 | chr5_136311377_+ | 5 | 0 | 0 | 0 | 0 |  |
| HAM/TSP#2 | 3951 | chr5_136311382_- | 8 | 0 | 0 | 0 | 0 |  |
| HAM/TSP#2 | 3952 | chr5_136415216_- | 0 | 1 | 2 | 0 | 0 |  |
| HAM/TSP#2 | 3953 | chr5_138019008_- | 1 | 0 | 0 | 0 | 0 |  |
| HAM/TSP#2 | 3954 | chr5_13805602_+ | 13 | 1 | 0 | 0 | 0 |  |
| HAM/TSP#2 | 3955 | chr5_1390180_- | 1 | 0 | 0 | 0 | 0 |  |
| HAM/TSP#2 | 3956 | chr5_139518239_- | 2 | 0 | 0 | 0 | 0 |  |
| HAM/TSP#2 | 3957 | chr5_139784414_- | 1 | 0 | 0 | 0 | 0 |  |
| HAM/TSP#2 | 3958 | chr5_139939423_- | 1 | 0 | 0 | 0 | 0 |  |
| HAM/TSP#2 | 3959 | chr5_142498479_- | 1 | 0 | 0 | 0 | 0 |  |
| HAM/TSP#2 | 3960 | chr5_143897714_+ | 1 | 0 | 0 | 0 | 0 |  |
| HAM/TSP#2 | 3961 | chr5_145552666_- | 19 | 0 | 0 | 0 | 0 |  |
| HAM/TSP#2 | 3962 | chr5_146573892_+ | 1 | 0 | 0 | 0 | 0 |  |
| HAM/TSP#2 | 3963 | chr5_14692786_+ | 7 | 0 | 0 | 0 | 0 |  |
| HAM/TSP#2 | 3964 | chr5_14737441_+ | 0 | 1 | 0 | 0 | 0 |  |
| HAM/TSP#2 | 3965 | chr5_150118584_+ | 1 | 0 | 0 | 0 | 0 |  |
| HAM/TSP#2 | 3966 | chr5_150618402_+ | 1 | 0 | 0 | 0 | 0 |  |
| HAM/TSP#2 | 3967 | chr5_153162190_- | 1 | 0 | 0 | 0 | 0 |  |
| HAM/TSP#2 | 3968 | chr5_153973649_+ | 2 | 0 | 0 | 0 | 0 |  |
| HAM/TSP#2 | 3969 | chr5_154201234_+ | 1 | 0 | 0 | 0 | 0 |  |
| HAM/TSP#2 | 3970 | chr5_154272328_+ | 3 | 0 | 0 | 0 | 0 |  |
| HAM/TSP#2 | 3971 | chr5_154391070_+ | 2 | 0 | 0 | 0 | 0 |  |
| HAM/TSP#2 | 3972 | chr5_154411328_+ | 0 | 1 | 0 | 0 | 0 |  |
| HAM/TSP#2 | 3973 | chr5_154825813_- | 4 | 0 | 0 | 0 | 0 |  |
| HAM/TSP#2 | 3974 | chr5_156214195_- | 12 | 0 | 0 | 0 | 0 |  |
| HAM/TSP#2 | 3975 | chr5_156903891_- | 0 | 0 | 0 | 1 | 0 |  |
| HAM/TSP#2 | 3976 | chr5_157668454_- | 1 | 0 | 0 | 0 | 0 |  |
| HAM/TSP#2 | 3977 | chr5_15768694_+ | 0 | 2 | 0 | 0 | 0 |  |
| HAM/TSP#2 | 3978 | chr5_158320707_+ | 1 | 0 | 0 | 0 | 0 |  |
| HAM/TSP#2 | 3979 | chr5_15867907_+ | 1 | 0 | 0 | 0 | 0 |  |
| HAM/TSP#2 | 3980 | chr5_163080748_- | 1 | 0 | 0 | 0 | 0 |  |
| HAM/TSP#2 | 3981 | chr5_163635272_- | 0 | 1 | 0 | 0 | 0 |  |
| HAM/TSP#2 | 3982 | chr5_164408272_- | 7 | 0 | 0 | 0 | 0 |  |
| HAM/TSP#2 | 3983 | chr5_16462049_- | 1 | 0 | 0 | 0 | 0 |  |
| HAM/TSP#2 | 3984 | chr5_16582637_- | 1 | 0 | 0 | 0 | 0 |  |
| HAM/TSP#2 | 3985 | chr5_167228571_- | 1 | 0 | 0 | 0 | 0 |  |
| HAM/TSP#2 | 3986 | chr5_167789145_- | 3 | 0 | 0 | 0 | 0 |  |
| HAM/TSP#2 | 3987 | chr5_168012844_- | 5 | 0 | 0 | 0 | 0 |  |
| HAM/TSP#2 | 3988 | chr5_171145967_+ | 2 | 0 | 0 | 0 | 0 |  |
| HAM/TSP#2 | 3989 | chr5_172187487_+ | 7 | 1 | 0 | 0 | 0 |  |
| HAM/TSP#2 | 3990 | chr5_1745594_- | 1 | 0 | 0 | 0 | 0 |  |
| HAM/TSP#2 | 3991 | chr5_175468414_- | 0 | 13 | 0 | 0 | 0 |  |
| HAM/TSP#2 | 3992 | chr5_175586403_+ | 1 | 0 | 0 | 0 | 0 |  |
| HAM/TSP#2 | 3993 | chr5_178147791_- | 1 | 0 | 0 | 0 | 0 |  |
| HAM/TSP#2 | 3994 | chr5_180462864_- | 1 | 0 | 0 | 0 | 0 |  |
| HAM/TSP#2 | 3995 | chr5_180810725_- | 2 | 0 | 0 | 0 | 0 |  |
| HAM/TSP#2 | 3996 | chr5_180871886_- | 31 | 0 | 0 | 0 | 0 |  |
| HAM/TSP#2 | 3997 | chr5_18218432_+ | 4 | 0 | 0 | 0 | 0 |  |
| HAM/TSP#2 | 3998 | chr5_19793742_- | 1 | 0 | 0 | 0 | 0 |  |
| HAM/TSP#2 | 3999 | chr5_20007574_+ | 3 | 1 | 0 | 0 | 0 |  |
| HAM/TSP#2 | 4000 | chr5_20345382_- | 3 | 0 | 0 | 0 | 0 |  |
| HAM/TSP#2 | 4001 | chr5_20543346_+ | 0 | 2 | 0 | 0 | 0 |  |
| HAM/TSP#2 | 4002 | chr5_21786635_- | 1 | 0 | 0 | 0 | 0 |  |
| HAM/TSP#2 | 4003 | chr5_22054435_- | 1 | 0 | 0 | 0 | 0 |  |
| HAM/TSP#2 | 4004 | chr5_22058444_+ | 4 | 0 | 0 | 0 | 0 |  |
| HAM/TSP#2 | 4005 | chr5_23106481_+ | 1 | 0 | 0 | 0 | 0 |  |
| HAM/TSP#2 | 4006 | chr5_24071228_+ | 10 | 0 | 0 | 0 | 0 |  |
| HAM/TSP#2 | 4007 | chr5_24812636_+ | 4 | 0 | 0 | 0 | 0 |  |
| HAM/TSP#2 | 4008 | chr5_2527108_- | 2 | 0 | 0 | 0 | 0 |  |
| HAM/TSP#2 | 4009 | chr5_30121155_- | 1 | 0 | 0 | 0 | 0 |  |
| HAM/TSP#2 | 4010 | chr5_30883223_- | 2 | 0 | 0 | 0 | 0 |  |
| HAM/TSP#2 | 4011 | chr5_32571227_- | 3 | 0 | 0 | 0 | 0 |  |
| HAM/TSP#2 | 4012 | chr5_34005908_+ | 2 | 0 | 0 | 0 | 0 |  |
| HAM/TSP#2 | 4013 | chr5_34021916_- | 3 | 0 | 0 | 0 | 0 |  |
| HAM/TSP#2 | 4014 | chr5_34332690_+ | 1 | 0 | 0 | 0 | 0 |  |
| HAM/TSP#2 | 4015 | chr5_34684547_- | 5 | 0 | 0 | 0 | 0 |  |
| HAM/TSP#2 | 4016 | chr5_34702589_- | 6 | 0 | 0 | 0 | 0 |  |
| HAM/TSP#2 | 4017 | chr5_34756112_- | 2 | 0 | 0 | 0 | 0 |  |
| HAM/TSP#2 | 4018 | chr5_35854031_+ | 2 | 0 | 0 | 0 | 0 |  |
| HAM/TSP#2 | 4019 | chr5_37433140_- | 1 | 0 | 0 | 0 | 0 |  |
| HAM/TSP#2 | 4020 | chr5_37572618_- | 6 | 0 | 0 | 0 | 0 |  |
| HAM/TSP#2 | 4021 | chr5_37834883_+ | 1 | 0 | 0 | 0 | 0 |  |
| HAM/TSP#2 | 4022 | chr5_40591640_+ | 0 | 1 | 0 | 0 | 0 |  |
| HAM/TSP#2 | 4023 | chr5_41550998_+ | 0 | 9 | 0 | 0 | 0 |  |
| HAM/TSP#2 | 4024 | chr5_41707929_+ | 3 | 0 | 0 | 0 | 0 |  |
| HAM/TSP#2 | 4025 | chr5_42553398_- | 46 | 0 | 0 | 0 | 2 |  |
| HAM/TSP#2 | 4026 | chr5_4380644_- | 1 | 0 | 0 | 0 | 0 |  |
| HAM/TSP#2 | 4027 | chr5_44278866_- | 1 | 0 | 0 | 0 | 0 |  |
| HAM/TSP#2 | 4028 | chr5_45391397_- | 1 | 0 | 0 | 0 | 0 |  |
| HAM/TSP#2 | 4029 | chr5_45518060_+ | 1 | 0 | 0 | 0 | 0 |  |
| HAM/TSP#2 | 4030 | chr5_46338550_- | 2 | 0 | 0 | 0 | 0 |  |
| HAM/TSP#2 | 4031 | chr5_46375810_+ | 2 | 0 | 0 | 0 | 0 |  |
| HAM/TSP#2 | 4032 | chr5_4759389_- | 1 | 0 | 0 | 0 | 0 |  |
| HAM/TSP#2 | 4033 | chr5_4953743_- | 1 | 0 | 0 | 0 | 0 |  |
| HAM/TSP#2 | 4034 | chr5_49599676_- | 1 | 0 | 0 | 0 | 0 |  |
| HAM/TSP#2 | 4035 | chr5_50465826_+ | 1 | 0 | 0 | 0 | 0 |  |
| HAM/TSP#2 | 4036 | chr5_5048304_- | 2 | 0 | 0 | 0 | 0 |  |
| HAM/TSP#2 | 4037 | chr5_5057617_- | 1 | 0 | 0 | 0 | 0 |  |
| HAM/TSP#2 | 4038 | chr5_50831377_- | 0 | 0 | 0 | 0 | 1 |  |
| HAM/TSP#2 | 4039 | chr5_5088511_+ | 1 | 0 | 0 | 0 | 0 |  |
| HAM/TSP#2 | 4040 | chr5_51504079_+ | 0 | 6 | 0 | 0 | 0 |  |
| HAM/TSP#2 | 4041 | chr5_5183357_- | 4 | 2 | 0 | 0 | 0 |  |
| HAM/TSP#2 | 4042 | chr5_5259611_+ | 9 | 0 | 0 | 0 | 0 |  |
| HAM/TSP#2 | 4043 | chr5_53573376_+ | 1 | 0 | 0 | 0 | 0 |  |
| HAM/TSP#2 | 4044 | chr5_54991162_- | 0 | 1 | 0 | 0 | 0 |  |
| HAM/TSP#2 | 4045 | chr5_56630673_+ | 1 | 0 | 0 | 0 | 1 |  |
| HAM/TSP#2 | 4046 | chr5_5712715_- | 1 | 0 | 0 | 0 | 0 |  |
| HAM/TSP#2 | 4047 | chr5_57842063_+ | 1 | 0 | 0 | 0 | 0 |  |
| HAM/TSP#2 | 4048 | chr5_59047003_+ | 6 | 0 | 0 | 0 | 0 |  |
| HAM/TSP#2 | 4049 | chr5_59803257_- | 0 | 1 | 0 | 0 | 0 |  |
| HAM/TSP#2 | 4050 | chr5_60001129_+ | 2 | 1 | 0 | 0 | 0 |  |
| HAM/TSP#2 | 4051 | chr5_60429391_- | 1 | 0 | 0 | 0 | 0 |  |
| HAM/TSP#2 | 4052 | chr5_60762302_- | 1 | 0 | 0 | 0 | 0 |  |
| HAM/TSP#2 | 4053 | chr5_61312595_+ | 5 | 0 | 0 | 0 | 0 |  |
| HAM/TSP#2 | 4054 | chr5_61976038_- | 1 | 0 | 0 | 0 | 0 |  |
| HAM/TSP#2 | 4055 | chr5_63182721_+ | 1 | 0 | 0 | 0 | 0 |  |
| HAM/TSP#2 | 4056 | chr5_64364968_+ | 1 | 0 | 0 | 0 | 0 |  |
| HAM/TSP#2 | 4057 | chr5_66122472_+ | 0 | 3 | 0 | 0 | 0 |  |
| HAM/TSP#2 | 4058 | chr5_68303966_- | 1 | 0 | 0 | 0 | 0 |  |
| HAM/TSP#2 | 4059 | chr5_68491192_- | 3 | 0 | 0 | 0 | 0 |  |
| HAM/TSP#2 | 4060 | chr5_71785012_- | 1 | 0 | 0 | 0 | 0 |  |
| HAM/TSP#2 | 4061 | chr5_71807551_+ | 1 | 0 | 0 | 0 | 0 |  |
| HAM/TSP#2 | 4062 | chr5_73729241_+ | 1 | 0 | 0 | 0 | 0 |  |
| HAM/TSP#2 | 4063 | chr5_75639542_- | 1 | 0 | 0 | 0 | 0 |  |
| HAM/TSP#2 | 4064 | chr5_76081704_+ | 1 | 0 | 0 | 0 | 0 |  |
| HAM/TSP#2 | 4065 | chr5_76736419_- | 2 | 0 | 0 | 0 | 0 |  |
| HAM/TSP#2 | 4066 | chr5_77003318_- | 1 | 0 | 0 | 0 | 0 |  |
| HAM/TSP#2 | 4067 | chr5_77364908_- | 2 | 0 | 0 | 0 | 0 |  |
| HAM/TSP#2 | 4068 | chr5_78559132_- | 1 | 0 | 0 | 0 | 0 |  |
| HAM/TSP#2 | 4069 | chr5_79664778_+ | 8 | 1 | 0 | 0 | 0 |  |
| HAM/TSP#2 | 4070 | chr5_80713217_+ | 1 | 0 | 0 | 0 | 0 |  |
| HAM/TSP#2 | 4071 | chr5_8082711_- | 2 | 0 | 0 | 0 | 0 |  |
| HAM/TSP#2 | 4072 | chr5_81863588_+ | 14 | 0 | 0 | 0 | 0 |  |
| HAM/TSP#2 | 4073 | chr5_83705177_+ | 1 | 0 | 0 | 0 | 0 |  |
| HAM/TSP#2 | 4074 | chr5_84320481_+ | 1 | 0 | 0 | 0 | 0 |  |
| HAM/TSP#2 | 4075 | chr5_84372168_+ | 1 | 0 | 0 | 0 | 0 |  |
| HAM/TSP#2 | 4076 | chr5_8461735_- | 1 | 0 | 0 | 0 | 0 |  |
| HAM/TSP#2 | 4077 | chr5_85098527_- | 1 | 0 | 0 | 0 | 0 |  |
| HAM/TSP#2 | 4078 | chr5_87820728_- | 1 | 0 | 0 | 0 | 0 |  |
| HAM/TSP#2 | 4079 | chr5_89614227_+ | 16 | 1 | 0 | 2 | 0 |  |
| HAM/TSP#2 | 4080 | chr5_90131975_+ | 1 | 0 | 0 | 0 | 0 |  |
| HAM/TSP#2 | 4081 | chr5_9025683_+ | 1 | 0 | 0 | 0 | 0 |  |
| HAM/TSP#2 | 4082 | chr5_90331203_+ | 0 | 0 | 0 | 3 | 0 |  |
| HAM/TSP#2 | 4083 | chr5_91078709_- | 1 | 0 | 0 | 0 | 0 |  |
| HAM/TSP#2 | 4084 | chr5_93469693_- | 1 | 0 | 0 | 0 | 0 |  |
| HAM/TSP#2 | 4085 | chr5_9378777_+ | 3 | 0 | 0 | 0 | 0 |  |
| HAM/TSP#2 | 4086 | chr5_94473_- | 1 | 0 | 0 | 0 | 0 |  |
| HAM/TSP#2 | 4087 | chr5_94825692_+ | 2 | 0 | 0 | 0 | 0 |  |
| HAM/TSP#2 | 4088 | chr5_94832450_- | 1 | 0 | 0 | 0 | 0 |  |
| HAM/TSP#2 | 4089 | chr5_95526345_- | 4 | 0 | 0 | 0 | 0 |  |
| HAM/TSP#2 | 4090 | chr5_96071640_+ | 1 | 0 | 0 | 0 | 0 |  |
| HAM/TSP#2 | 4091 | chr5_96208107_- | 1 | 0 | 0 | 0 | 0 |  |
| HAM/TSP#2 | 4092 | chr5_96669924_- | 1 | 1 | 0 | 0 | 0 |  |
| HAM/TSP#2 | 4093 | chr5_96696492_- | 8 | 1 | 0 | 0 | 0 |  |
| HAM/TSP#2 | 4094 | chr5_97183370_- | 3 | 0 | 0 | 0 | 0 |  |
| HAM/TSP#2 | 4095 | chr5_98393451_- | 1 | 0 | 0 | 0 | 0 |  |
| HAM/TSP#2 | 4096 | chr5_99217744_+ | 3 | 0 | 0 | 0 | 0 |  |
| HAM/TSP#2 | 4097 | chr6_102796509_- | 1 | 0 | 0 | 0 | 0 |  |
| HAM/TSP#2 | 4098 | chr6_10649640_+ | 14 | 0 | 0 | 0 | 0 |  |
| HAM/TSP#2 | 4099 | chr6_106666073_- | 0 | 1 | 0 | 0 | 0 |  |
| HAM/TSP#2 | 4100 | chr6_107652996_+ | 0 | 2 | 0 | 0 | 0 |  |
| HAM/TSP#2 | 4101 | chr6_108299574_+ | 4 | 0 | 0 | 0 | 0 |  |
| HAM/TSP#2 | 4102 | chr6_108945516_+ | 1 | 0 | 0 | 0 | 0 |  |
| HAM/TSP#2 | 4103 | chr6_109920484_- | 1 | 0 | 0 | 0 | 0 |  |
| HAM/TSP#2 | 4104 | chr6_111553922_- | 0 | 2 | 0 | 0 | 0 |  |
| HAM/TSP#2 | 4105 | chr6_11295488_- | 1 | 0 | 0 | 0 | 0 |  |
| HAM/TSP#2 | 4106 | chr6_11347527_+ | 1 | 0 | 0 | 0 | 0 |  |
| HAM/TSP#2 | 4107 | chr6_114529963_- | 0 | 1 | 0 | 0 | 0 |  |
| HAM/TSP#2 | 4108 | chr6_115401153_+ | 1 | 0 | 0 | 0 | 0 |  |
| HAM/TSP#2 | 4109 | chr6_116573015_- | 1 | 0 | 0 | 0 | 0 |  |
| HAM/TSP#2 | 4110 | chr6_11743578_- | 0 | 6 | 0 | 0 | 0 |  |
| HAM/TSP#2 | 4111 | chr6_11797790_- | 5 | 0 | 0 | 0 | 0 |  |
| HAM/TSP#2 | 4112 | chr6_119080190_+ | 4 | 0 | 0 | 0 | 0 |  |
| HAM/TSP#2 | 4113 | chr6_120176184_- | 1 | 0 | 0 | 0 | 0 |  |
| HAM/TSP#2 | 4114 | chr6_122598571_- | 2 | 0 | 0 | 0 | 0 |  |
| HAM/TSP#2 | 4115 | chr6_123655246_+ | 0 | 2 | 0 | 0 | 0 |  |
| HAM/TSP#2 | 4116 | chr6_125131565_- | 0 | 1 | 0 | 0 | 0 |  |
| HAM/TSP#2 | 4117 | chr6_128980234_+ | 3 | 0 | 0 | 0 | 0 |  |
| HAM/TSP#2 | 4118 | chr6_131948899_- | 1 | 0 | 0 | 0 | 0 |  |
| HAM/TSP#2 | 4119 | chr6_135355794_+ | 0 | 0 | 0 | 0 | 1 |  |
| HAM/TSP#2 | 4120 | chr6_137493646_- | 1 | 0 | 0 | 0 | 0 |  |
| HAM/TSP#2 | 4121 | chr6_138773810_- | 1 | 0 | 0 | 0 | 0 |  |
| HAM/TSP#2 | 4122 | chr6_143001597_- | 0 | 1 | 0 | 0 | 0 |  |
| HAM/TSP#2 | 4123 | chr6_143594532_+ | 0 | 3 | 0 | 0 | 0 |  |
| HAM/TSP#2 | 4124 | chr6_144998632_- | 1 | 0 | 0 | 0 | 0 |  |
| HAM/TSP#2 | 4125 | chr6_145631796_+ | 2 | 0 | 0 | 0 | 0 |  |
| HAM/TSP#2 | 4126 | chr6_148759357_- | 4 | 0 | 0 | 0 | 0 |  |
| HAM/TSP#2 | 4127 | chr6_149355344_- | 1 | 0 | 0 | 0 | 0 |  |
| HAM/TSP#2 | 4128 | chr6_151430303_+ | 1 | 0 | 0 | 0 | 0 |  |
| HAM/TSP#2 | 4129 | chr6_152772470_- | 1 | 0 | 0 | 0 | 0 |  |
| HAM/TSP#2 | 4130 | chr6_155214278_- | 1 | 0 | 0 | 0 | 0 |  |
| HAM/TSP#2 | 4131 | chr6_155284144_- | 1 | 0 | 0 | 0 | 0 |  |
| HAM/TSP#2 | 4132 | chr6_158486466_- | 1 | 0 | 0 | 0 | 0 |  |
| HAM/TSP#2 | 4133 | chr6_159723319_- | 1 | 0 | 0 | 0 | 0 |  |
| HAM/TSP#2 | 4134 | chr6_161830038_- | 6 | 0 | 0 | 0 | 0 |  |
| HAM/TSP#2 | 4135 | chr6_163492874_- | 2 | 0 | 0 | 0 | 0 |  |
| HAM/TSP#2 | 4136 | chr6_166696405_- | 6 | 0 | 0 | 0 | 0 |  |
| HAM/TSP#2 | 4137 | chr6_167415946_- | 1 | 0 | 0 | 0 | 0 |  |
| HAM/TSP#2 | 4138 | chr6_167791627_+ | 1 | 0 | 0 | 0 | 0 |  |
| HAM/TSP#2 | 4139 | chr6_168643421_- | 1 | 0 | 0 | 0 | 0 |  |
| HAM/TSP#2 | 4140 | chr6_170523770_- | 0 | 1 | 0 | 0 | 0 |  |
| HAM/TSP#2 | 4141 | chr6_19183660_- | 11 | 0 | 0 | 0 | 0 |  |
| HAM/TSP#2 | 4142 | chr6_20219236_+ | 4 | 0 | 0 | 0 | 0 |  |
| HAM/TSP#2 | 4143 | chr6_21542773_- | 1 | 0 | 0 | 0 | 0 |  |
| HAM/TSP#2 | 4144 | chr6_24224502_+ | 1 | 0 | 0 | 0 | 0 |  |
| HAM/TSP#2 | 4145 | chr6_25042533_- | 1 | 0 | 0 | 0 | 0 |  |
| HAM/TSP#2 | 4146 | chr6_27634053_- | 1 | 0 | 0 | 0 | 0 |  |
| HAM/TSP#2 | 4147 | chr6_2779135_- | 0 | 17 | 0 | 0 | 0 |  |
| HAM/TSP#2 | 4148 | chr6_27838814_- | 3 | 0 | 0 | 0 | 0 |  |
| HAM/TSP#2 | 4149 | chr6_28092296_+ | 0 | 2 | 0 | 0 | 0 |  |
| HAM/TSP#2 | 4150 | chr6_2861581_- | 1 | 0 | 0 | 0 | 0 |  |
| HAM/TSP#2 | 4151 | chr6_29316979_- | 1 | 0 | 0 | 0 | 0 |  |
| HAM/TSP#2 | 4152 | chr6_30516972_+ | 1 | 0 | 0 | 0 | 0 |  |
| HAM/TSP#2 | 4153 | chr6_32038879_+ | 0 | 0 | 0 | 1 | 0 |  |
| HAM/TSP#2 | 4154 | chr6_3251010_+ | 1 | 0 | 0 | 0 | 0 |  |
| HAM/TSP#2 | 4155 | chr6_33774038_- | 1 | 0 | 0 | 0 | 0 |  |
| HAM/TSP#2 | 4156 | chr6_3454797_+ | 3 | 0 | 0 | 0 | 0 |  |
| HAM/TSP#2 | 4157 | chr6_35516638_- | 1 | 0 | 0 | 0 | 0 |  |
| HAM/TSP#2 | 4158 | chr6_3603446_+ | 1 | 0 | 0 | 0 | 0 |  |
| HAM/TSP#2 | 4159 | chr6_36186805_- | 2 | 0 | 0 | 0 | 0 |  |
| HAM/TSP#2 | 4160 | chr6_36671229_- | 0 | 0 | 0 | 1 | 0 |  |
| HAM/TSP#2 | 4161 | chr6_36722355_+ | 1 | 0 | 0 | 0 | 0 |  |
| HAM/TSP#2 | 4162 | chr6_36867638_- | 0 | 2 | 0 | 0 | 0 |  |
| HAM/TSP#2 | 4163 | chr6_37046059_+ | 2 | 0 | 0 | 0 | 0 |  |
| HAM/TSP#2 | 4164 | chr6_3724742_+ | 1 | 0 | 0 | 0 | 0 |  |
| HAM/TSP#2 | 4165 | chr6_38069318_+ | 1 | 0 | 0 | 0 | 0 |  |
| HAM/TSP#2 | 4166 | chr6_3886537_+ | 1 | 0 | 0 | 0 | 0 |  |
| HAM/TSP#2 | 4167 | chr6_40378822_+ | 5 | 0 | 0 | 0 | 1 |  |
| HAM/TSP#2 | 4168 | chr6_40884106_+ | 1 | 0 | 0 | 0 | 0 |  |
| HAM/TSP#2 | 4169 | chr6_40954279_+ | 1 | 0 | 0 | 0 | 0 |  |
| HAM/TSP#2 | 4170 | chr6_41345285_- | 3 | 0 | 0 | 0 | 0 |  |
| HAM/TSP#2 | 4171 | chr6_43020392_+ | 0 | 0 | 0 | 1 | 0 |  |
| HAM/TSP#2 | 4172 | chr6_43133170_- | 1 | 0 | 0 | 0 | 0 |  |
| HAM/TSP#2 | 4173 | chr6_45785196_+ | 0 | 4 | 0 | 0 | 0 |  |
| HAM/TSP#2 | 4174 | chr6_46176525_- | 1 | 0 | 0 | 0 | 0 |  |
| HAM/TSP#2 | 4175 | chr6_46502610_- | 1 | 0 | 0 | 0 | 0 |  |
| HAM/TSP#2 | 4176 | chr6_48551492_+ | 0 | 1 | 0 | 0 | 0 |  |
| HAM/TSP#2 | 4177 | chr6_48786066_+ | 3 | 0 | 0 | 0 | 0 |  |
| HAM/TSP#2 | 4178 | chr6_52257281_+ | 1 | 0 | 0 | 0 | 0 |  |
| HAM/TSP#2 | 4179 | chr6_52628385_- | 1 | 0 | 0 | 0 | 0 |  |
| HAM/TSP#2 | 4180 | chr6_52824939_+ | 2 | 0 | 0 | 0 | 0 |  |
| HAM/TSP#2 | 4181 | chr6_53310408_- | 2 | 0 | 0 | 0 | 0 |  |
| HAM/TSP#2 | 4182 | chr6_54384622_- | 1 | 0 | 0 | 0 | 0 |  |
| HAM/TSP#2 | 4183 | chr6_56419047_+ | 2 | 0 | 0 | 0 | 0 |  |
| HAM/TSP#2 | 4184 | chr6_56717338_+ | 1 | 0 | 0 | 0 | 0 |  |
| HAM/TSP#2 | 4185 | chr6_57189139_+ | 5 | 0 | 0 | 0 | 0 |  |
| HAM/TSP#2 | 4186 | chr6_57517111_+ | 1 | 0 | 0 | 0 | 0 |  |
| HAM/TSP#2 | 4187 | chr6_58401307_- | 1 | 0 | 0 | 0 | 0 |  |
| HAM/TSP#2 | 4188 | chr6_61956226_- | 2 | 0 | 0 | 0 | 0 |  |
| HAM/TSP#2 | 4189 | chr6_63338811_+ | 1 | 0 | 0 | 0 | 0 |  |
| HAM/TSP#2 | 4190 | chr6_64933549_+ | 0 | 2 | 0 | 0 | 0 |  |
| HAM/TSP#2 | 4191 | chr6_65627421_+ | 0 | 1 | 0 | 0 | 0 |  |
| HAM/TSP#2 | 4192 | chr6_65730393_+ | 1 | 0 | 0 | 0 | 0 |  |
| HAM/TSP#2 | 4193 | chr6_66341011_- | 1 | 0 | 0 | 0 | 0 |  |
| HAM/TSP#2 | 4194 | chr6_67723249_+ | 2 | 0 | 0 | 0 | 0 |  |
| HAM/TSP#2 | 4195 | chr6_67869952_+ | 5 | 0 | 0 | 0 | 0 |  |
| HAM/TSP#2 | 4196 | chr6_69269159_+ | 1 | 0 | 0 | 0 | 0 |  |
| HAM/TSP#2 | 4197 | chr6_69788326_- | 0 | 1 | 0 | 0 | 0 |  |
| HAM/TSP#2 | 4198 | chr6_70393662_+ | 1 | 0 | 0 | 0 | 0 |  |
| HAM/TSP#2 | 4199 | chr6_70470653_- | 1 | 0 | 0 | 0 | 0 |  |
| HAM/TSP#2 | 4200 | chr6_705318_+ | 1 | 1 | 0 | 0 | 0 |  |
| HAM/TSP#2 | 4201 | chr6_70534606_- | 1 | 0 | 0 | 0 | 0 |  |
| HAM/TSP#2 | 4202 | chr6_71066754_+ | 1 | 0 | 0 | 0 | 0 |  |
| HAM/TSP#2 | 4203 | chr6_7251935_+ | 1 | 0 | 0 | 0 | 0 |  |
| HAM/TSP#2 | 4204 | chr6_72620647_- | 4 | 0 | 0 | 0 | 0 |  |
| HAM/TSP#2 | 4205 | chr6_73309989_- | 17 | 0 | 0 | 0 | 0 |  |
| HAM/TSP#2 | 4206 | chr6_73434928_+ | 2 | 0 | 0 | 0 | 0 |  |
| HAM/TSP#2 | 4207 | chr6_7395782_+ | 1 | 0 | 0 | 0 | 0 |  |
| HAM/TSP#2 | 4208 | chr6_75210874_- | 5 | 0 | 0 | 0 | 0 |  |
| HAM/TSP#2 | 4209 | chr6_75603303_+ | 3 | 0 | 0 | 0 | 0 |  |
| HAM/TSP#2 | 4210 | chr6_75710638_- | 1 | 0 | 0 | 0 | 0 |  |
| HAM/TSP#2 | 4211 | chr6_75956798_- | 1 | 0 | 0 | 0 | 0 |  |
| HAM/TSP#2 | 4212 | chr6_76463501_+ | 2 | 0 | 0 | 0 | 0 |  |
| HAM/TSP#2 | 4213 | chr6_77410511_- | 1 | 0 | 0 | 0 | 0 |  |
| HAM/TSP#2 | 4214 | chr6_77566557_- | 4 | 0 | 0 | 0 | 0 |  |
| HAM/TSP#2 | 4215 | chr6_80135839_+ | 1 | 0 | 0 | 0 | 0 |  |
| HAM/TSP#2 | 4216 | chr6_80240141_+ | 0 | 1 | 0 | 0 | 0 |  |
| HAM/TSP#2 | 4217 | chr6_80278357_- | 1 | 0 | 0 | 0 | 0 |  |
| HAM/TSP#2 | 4218 | chr6_83935262_- | 1 | 0 | 0 | 0 | 0 |  |
| HAM/TSP#2 | 4219 | chr6_84903900_- | 1 | 0 | 0 | 0 | 0 |  |
| HAM/TSP#2 | 4220 | chr6_85428272_+ | 3 | 0 | 0 | 0 | 0 |  |
| HAM/TSP#2 | 4221 | chr6_86399899_- | 3 | 0 | 0 | 0 | 0 |  |
| HAM/TSP#2 | 4222 | chr6_87099457_+ | 1 | 0 | 0 | 0 | 0 |  |
| HAM/TSP#2 | 4223 | chr6_87176003_- | 1 | 0 | 0 | 0 | 0 |  |
| HAM/TSP#2 | 4224 | chr6_89897679_- | 1 | 0 | 0 | 0 | 0 |  |
| HAM/TSP#2 | 4225 | chr6_90077982_+ | 1 | 0 | 0 | 0 | 0 |  |
| HAM/TSP#2 | 4226 | chr6_91296487_- | 4 | 0 | 0 | 0 | 0 |  |
| HAM/TSP#2 | 4227 | chr6_94677388_- | 8 | 0 | 0 | 0 | 0 |  |
| HAM/TSP#2 | 4228 | chr6_96038099_- | 0 | 0 | 0 | 0 | 1 |  |
| HAM/TSP#2 | 4229 | chr6_97352607_+ | 1 | 0 | 0 | 0 | 0 |  |
| HAM/TSP#2 | 4230 | chr7_102802215_+ | 0 | 0 | 0 | 1 | 0 |  |
| HAM/TSP#2 | 4231 | chr7_102930501_+ | 0 | 2 | 0 | 0 | 0 |  |
| HAM/TSP#2 | 4232 | chr7_103439827_+ | 0 | 1 | 0 | 0 | 0 |  |
| HAM/TSP#2 | 4233 | chr7_105115927_+ | 1 | 0 | 0 | 0 | 0 |  |
| HAM/TSP#2 | 4234 | chr7_111892413_+ | 0 | 1 | 0 | 0 | 0 |  |
| HAM/TSP#2 | 4235 | chr7_113785381_- | 2 | 0 | 0 | 0 | 0 |  |
| HAM/TSP#2 | 4236 | chr7_11891769_+ | 1 | 0 | 0 | 0 | 0 |  |
| HAM/TSP#2 | 4237 | chr7_120069928_- | 1 | 0 | 0 | 0 | 0 |  |
| HAM/TSP#2 | 4238 | chr7_121492218_+ | 1 | 0 | 0 | 0 | 0 |  |
| HAM/TSP#2 | 4239 | chr7_124186880_+ | 1 | 0 | 0 | 0 | 0 |  |
| HAM/TSP#2 | 4240 | chr7_124377547_+ | 1 | 0 | 0 | 0 | 0 |  |
| HAM/TSP#2 | 4241 | chr7_124587587_+ | 5 | 0 | 0 | 0 | 0 |  |
| HAM/TSP#2 | 4242 | chr7_125210997_- | 1 | 0 | 0 | 0 | 0 |  |
| HAM/TSP#2 | 4243 | chr7_125864039_- | 1 | 0 | 0 | 0 | 0 |  |
| HAM/TSP#2 | 4244 | chr7_126156436_- | 1 | 0 | 0 | 0 | 0 |  |
| HAM/TSP#2 | 4245 | chr7_127256406_- | 1 | 0 | 0 | 0 | 0 |  |
| HAM/TSP#2 | 4246 | chr7_131146007_+ | 1 | 0 | 0 | 0 | 0 |  |
| HAM/TSP#2 | 4247 | chr7_136078963_+ | 2 | 0 | 0 | 0 | 0 |  |
| HAM/TSP#2 | 4248 | chr7_136079003_+ | 1 | 0 | 0 | 0 | 0 |  |
| HAM/TSP#2 | 4249 | chr7_13693636_+ | 1 | 0 | 0 | 0 | 0 |  |
| HAM/TSP#2 | 4250 | chr7_140566370_+ | 0 | 0 | 0 | 0 | 1 |  |
| HAM/TSP#2 | 4251 | chr7_142131196_- | 1 | 0 | 0 | 0 | 0 |  |
| HAM/TSP#2 | 4252 | chr7_143321358_- | 1 | 0 | 0 | 0 | 0 |  |
| HAM/TSP#2 | 4253 | chr7_144426127_- | 1 | 0 | 0 | 0 | 0 |  |
| HAM/TSP#2 | 4254 | chr7_147014000_- | 8 | 0 | 0 | 0 | 0 |  |
| HAM/TSP#2 | 4255 | chr7_147806940_- | 0 | 1 | 0 | 0 | 0 |  |
| HAM/TSP#2 | 4256 | chr7_149045748_- | 1 | 0 | 0 | 0 | 0 |  |
| HAM/TSP#2 | 4257 | chr7_149088162_- | 5 | 0 | 0 | 0 | 0 |  |
| HAM/TSP#2 | 4258 | chr7_14916711_+ | 5 | 0 | 0 | 0 | 0 |  |
| HAM/TSP#2 | 4259 | chr7_149828800_+ | 4 | 0 | 0 | 0 | 0 |  |
| HAM/TSP#2 | 4260 | chr7_150044020_+ | 1 | 0 | 0 | 0 | 0 |  |
| HAM/TSP#2 | 4261 | chr7_150944133_+ | 1 | 0 | 0 | 0 | 0 |  |
| HAM/TSP#2 | 4262 | chr7_151870521_+ | 1 | 0 | 0 | 0 | 0 |  |
| HAM/TSP#2 | 4263 | chr7_154022712_- | 2 | 0 | 0 | 0 | 0 |  |
| HAM/TSP#2 | 4264 | chr7_157571362_+ | 2 | 0 | 0 | 0 | 0 |  |
| HAM/TSP#2 | 4265 | chr7_158343622_- | 3 | 0 | 0 | 0 | 0 |  |
| HAM/TSP#2 | 4266 | chr7_158843013_- | 6 | 1 | 0 | 0 | 0 |  |
| HAM/TSP#2 | 4267 | chr7_159222639_+ | 0 | 1 | 0 | 0 | 0 |  |
| HAM/TSP#2 | 4268 | chr7_17054437_- | 1 | 0 | 0 | 0 | 0 |  |
| HAM/TSP#2 | 4269 | chr7_17268553_- | 9 | 0 | 0 | 0 | 0 |  |
| HAM/TSP#2 | 4270 | chr7_18613946_+ | 0 | 8 | 0 | 0 | 0 |  |
| HAM/TSP#2 | 4271 | chr7_19530169_+ | 1 | 0 | 0 | 0 | 0 |  |
| HAM/TSP#2 | 4272 | chr7_19715889_+ | 2 | 0 | 0 | 0 | 0 |  |
| HAM/TSP#2 | 4273 | chr7_2034404_+ | 3 | 0 | 0 | 0 | 0 |  |
| HAM/TSP#2 | 4274 | chr7_23481436_- | 3 | 0 | 0 | 0 | 0 |  |
| HAM/TSP#2 | 4275 | chr7_23542388_+ | 1 | 0 | 0 | 0 | 0 |  |
| HAM/TSP#2 | 4276 | chr7_26068731_+ | 1 | 0 | 0 | 0 | 0 |  |
| HAM/TSP#2 | 4277 | chr7_26087554_+ | 1 | 0 | 0 | 0 | 0 |  |
| HAM/TSP#2 | 4278 | chr7_26579062_- | 1 | 0 | 0 | 0 | 0 |  |
| HAM/TSP#2 | 4279 | chr7_2731377_- | 1 | 0 | 0 | 0 | 0 |  |
| HAM/TSP#2 | 4280 | chr7_27668195_- | 2 | 0 | 0 | 0 | 0 |  |
| HAM/TSP#2 | 4281 | chr7_30438182_+ | 1 | 0 | 0 | 0 | 0 |  |
| HAM/TSP#2 | 4282 | chr7_30860709_- | 1 | 0 | 0 | 0 | 0 |  |
| HAM/TSP#2 | 4283 | chr7_31478054_+ | 3 | 0 | 0 | 0 | 0 |  |
| HAM/TSP#2 | 4284 | chr7_32638698_- | 0 | 2 | 0 | 0 | 0 |  |
| HAM/TSP#2 | 4285 | chr7_32947774_+ | 0 | 25 | 0 | 0 | 0 |  |
| HAM/TSP#2 | 4286 | chr7_33834079_+ | 2 | 0 | 0 | 0 | 0 |  |
| HAM/TSP#2 | 4287 | chr7_3572449_- | 4 | 0 | 0 | 0 | 0 |  |
| HAM/TSP#2 | 4288 | chr7_35879016_+ | 1 | 0 | 0 | 0 | 0 |  |
| HAM/TSP#2 | 4289 | chr7_3633848_- | 1 | 0 | 0 | 0 | 0 |  |
| HAM/TSP#2 | 4290 | chr7_36723196_- | 5 | 0 | 0 | 0 | 0 |  |
| HAM/TSP#2 | 4291 | chr7_37717589_- | 0 | 0 | 0 | 1 | 0 |  |
| HAM/TSP#2 | 4292 | chr7_37965041_+ | 7 | 0 | 0 | 0 | 0 |  |
| HAM/TSP#2 | 4293 | chr7_38668357_+ | 0 | 1 | 0 | 0 | 0 |  |
| HAM/TSP#2 | 4294 | chr7_39608822_- | 6 | 0 | 0 | 0 | 0 |  |
| HAM/TSP#2 | 4295 | chr7_40193004_- | 1 | 0 | 0 | 0 | 0 |  |
| HAM/TSP#2 | 4296 | chr7_41034089_+ | 0 | 1 | 0 | 0 | 0 |  |
| HAM/TSP#2 | 4297 | chr7_41198153_+ | 15 | 2 | 0 | 0 | 0 |  |
| HAM/TSP#2 | 4298 | chr7_42296504_- | 5 | 1 | 0 | 0 | 0 |  |
| HAM/TSP#2 | 4299 | chr7_4357969_- | 7 | 0 | 0 | 0 | 0 |  |
| HAM/TSP#2 | 4300 | chr7_43644745_+ | 0 | 0 | 0 | 14 | 0 |  |
| HAM/TSP#2 | 4301 | chr7_45328438_+ | 1 | 0 | 0 | 0 | 0 |  |
| HAM/TSP#2 | 4302 | chr7_45889143_+ | 1 | 0 | 0 | 0 | 0 |  |
| HAM/TSP#2 | 4303 | chr7_46196140_+ | 1 | 0 | 0 | 0 | 0 |  |
| HAM/TSP#2 | 4304 | chr7_46344010_+ | 7 | 0 | 0 | 0 | 0 |  |
| HAM/TSP#2 | 4305 | chr7_46954782_+ | 1 | 0 | 0 | 0 | 0 |  |
| HAM/TSP#2 | 4306 | chr7_47237095_- | 4 | 0 | 0 | 0 | 0 |  |
| HAM/TSP#2 | 4307 | chr7_4765927_- | 1 | 0 | 0 | 0 | 0 |  |
| HAM/TSP#2 | 4308 | chr7_47833801_+ | 1 | 0 | 0 | 0 | 0 |  |
| HAM/TSP#2 | 4309 | chr7_4805272_- | 5 | 0 | 0 | 0 | 0 |  |
| HAM/TSP#2 | 4310 | chr7_48078915_+ | 1 | 0 | 0 | 0 | 0 |  |
| HAM/TSP#2 | 4311 | chr7_48279834_- | 1 | 0 | 0 | 0 | 0 |  |
| HAM/TSP#2 | 4312 | chr7_48665365_+ | 1 | 0 | 0 | 0 | 0 |  |
| HAM/TSP#2 | 4313 | chr7_50870375_- | 1 | 0 | 0 | 0 | 0 |  |
| HAM/TSP#2 | 4314 | chr7_51031735_+ | 1 | 0 | 0 | 0 | 0 |  |
| HAM/TSP#2 | 4315 | chr7_51389384_- | 2 | 0 | 0 | 0 | 0 |  |
| HAM/TSP#2 | 4316 | chr7_51763951_+ | 2 | 0 | 0 | 0 | 0 |  |
| HAM/TSP#2 | 4317 | chr7_52540993_- | 1 | 0 | 0 | 0 | 0 |  |
| HAM/TSP#2 | 4318 | chr7_53708816_+ | 2 | 0 | 0 | 0 | 0 |  |
| HAM/TSP#2 | 4319 | chr7_53749296_- | 4 | 0 | 0 | 0 | 0 |  |
| HAM/TSP#2 | 4320 | chr7_54057599_+ | 1 | 0 | 0 | 0 | 0 |  |
| HAM/TSP#2 | 4321 | chr7_54101723_- | 2 | 0 | 0 | 0 | 0 |  |
| HAM/TSP#2 | 4322 | chr7_54186188_- | 1 | 0 | 0 | 0 | 0 |  |
| HAM/TSP#2 | 4323 | chr7_55310397_- | 1 | 0 | 0 | 0 | 0 |  |
| HAM/TSP#2 | 4324 | chr7_55507161_+ | 1 | 0 | 0 | 0 | 0 |  |
| HAM/TSP#2 | 4325 | chr7_55567654_+ | 5 | 0 | 0 | 0 | 0 |  |
| HAM/TSP#2 | 4326 | chr7_56093564_+ | 3 | 0 | 0 | 0 | 0 |  |
| HAM/TSP#2 | 4327 | chr7_57719913_+ | 7 | 0 | 0 | 0 | 0 |  |
| HAM/TSP#2 | 4328 | chr7_60929243_+ | 4 | 0 | 0 | 0 | 0 |  |
| HAM/TSP#2 | 4329 | chr7_61046840_+ | 1 | 0 | 0 | 0 | 0 |  |
| HAM/TSP#2 | 4330 | chr7_62164827_- | 1 | 0 | 0 | 0 | 0 |  |
| HAM/TSP#2 | 4331 | chr7_62269264_+ | 0 | 0 | 0 | 0 | 1 |  |
| HAM/TSP#2 | 4332 | chr7_66702906_+ | 1 | 0 | 0 | 0 | 0 |  |
| HAM/TSP#2 | 4333 | chr7_69060820_+ | 1 | 0 | 0 | 0 | 0 |  |
| HAM/TSP#2 | 4334 | chr7_69507915_- | 1 | 0 | 0 | 0 | 0 |  |
| HAM/TSP#2 | 4335 | chr7_69865208_+ | 2 | 0 | 0 | 0 | 0 |  |
| HAM/TSP#2 | 4336 | chr7_70125033_- | 0 | 0 | 0 | 5 | 0 |  |
| HAM/TSP#2 | 4337 | chr7_70400560_+ | 5 | 0 | 0 | 0 | 0 |  |
| HAM/TSP#2 | 4338 | chr7_70552375_- | 1 | 0 | 0 | 0 | 0 |  |
| HAM/TSP#2 | 4339 | chr7_71494775_+ | 1 | 0 | 0 | 0 | 0 |  |
| HAM/TSP#2 | 4340 | chr7_72232039_- | 0 | 2 | 0 | 0 | 0 |  |
| HAM/TSP#2 | 4341 | chr7_72366844_- | 1 | 0 | 0 | 0 | 0 |  |
| HAM/TSP#2 | 4342 | chr7_72447500_- | 1 | 0 | 0 | 0 | 0 |  |
| HAM/TSP#2 | 4343 | chr7_73425304_- | 1 | 0 | 0 | 0 | 0 |  |
| HAM/TSP#2 | 4344 | chr7_73721226_+ | 4 | 0 | 0 | 0 | 0 |  |
| HAM/TSP#2 | 4345 | chr7_74061158_+ | 1 | 0 | 0 | 0 | 0 |  |
| HAM/TSP#2 | 4346 | chr7_74436612_+ | 1 | 0 | 0 | 0 | 0 |  |
| HAM/TSP#2 | 4347 | chr7_74622666_+ | 0 | 0 | 0 | 1 | 0 |  |
| HAM/TSP#2 | 4348 | chr7_78416965_- | 1 | 0 | 0 | 0 | 0 |  |
| HAM/TSP#2 | 4349 | chr7_80233442_+ | 1 | 0 | 0 | 0 | 0 |  |
| HAM/TSP#2 | 4350 | chr7_80280757_+ | 1 | 0 | 0 | 0 | 0 |  |
| HAM/TSP#2 | 4351 | chr7_80765582_- | 1 | 0 | 0 | 0 | 0 |  |
| HAM/TSP#2 | 4352 | chr7_80778701_+ | 1 | 0 | 0 | 0 | 0 |  |
| HAM/TSP#2 | 4353 | chr7_80927602_+ | 0 | 7 | 0 | 0 | 0 |  |
| HAM/TSP#2 | 4354 | chr7_81383298_- | 2 | 0 | 0 | 0 | 0 |  |
| HAM/TSP#2 | 4355 | chr7_82973203_- | 1 | 0 | 0 | 0 | 1 |  |
| HAM/TSP#2 | 4356 | chr7_84647913_- | 4 | 0 | 0 | 0 | 0 |  |
| HAM/TSP#2 | 4357 | chr7_86055293_+ | 1 | 0 | 0 | 0 | 0 |  |
| HAM/TSP#2 | 4358 | chr7_86657341_+ | 1 | 0 | 0 | 0 | 0 |  |
| HAM/TSP#2 | 4359 | chr7_8747660_- | 0 | 1 | 0 | 0 | 0 |  |
| HAM/TSP#2 | 4360 | chr7_87501569_- | 0 | 0 | 0 | 0 | 6 |  |
| HAM/TSP#2 | 4361 | chr7_9019350_+ | 1 | 0 | 0 | 0 | 0 |  |
| HAM/TSP#2 | 4362 | chr7_90735934_+ | 1 | 0 | 0 | 0 | 0 |  |
| HAM/TSP#2 | 4363 | chr7_91267422_+ | 1 | 0 | 0 | 0 | 0 |  |
| HAM/TSP#2 | 4364 | chr7_92052653_- | 2 | 0 | 0 | 0 | 0 |  |
| HAM/TSP#2 | 4365 | chr7_9208642_- | 3 | 0 | 0 | 0 | 0 |  |
| HAM/TSP#2 | 4366 | chr7_98250093_- | 3 | 0 | 0 | 0 | 0 |  |
| HAM/TSP#2 | 4367 | chr7_99458780_- | 1 | 0 | 0 | 0 | 0 |  |
| HAM/TSP#2 | 4368 | chr7_99698201_+ | 1 | 0 | 0 | 0 | 0 |  |
| HAM/TSP#2 | 4369 | chr8_100413383_+ | 3 | 0 | 0 | 0 | 0 |  |
| HAM/TSP#2 | 4370 | chr8_101985045_- | 1 | 0 | 0 | 0 | 0 |  |
| HAM/TSP#2 | 4371 | chr8_102735048_- | 0 | 2 | 0 | 0 | 0 |  |
| HAM/TSP#2 | 4372 | chr8_103459839_+ | 2 | 0 | 0 | 0 | 0 |  |
| HAM/TSP#2 | 4373 | chr8_105344351_- | 1 | 0 | 0 | 0 | 0 |  |
| HAM/TSP#2 | 4374 | chr8_107445539_- | 1 | 0 | 0 | 0 | 0 |  |
| HAM/TSP#2 | 4375 | chr8_107456232_+ | 36 | 0 | 0 | 0 | 0 |  |
| HAM/TSP#2 | 4376 | chr8_109317598_- | 1 | 0 | 0 | 0 | 0 |  |
| HAM/TSP#2 | 4377 | chr8_110624354_- | 1 | 0 | 0 | 0 | 0 |  |
| HAM/TSP#2 | 4378 | chr8_11219083_+ | 2 | 0 | 0 | 0 | 0 |  |
| HAM/TSP#2 | 4379 | chr8_116373569_- | 2 | 0 | 0 | 0 | 0 |  |
| HAM/TSP#2 | 4380 | chr8_11679181_+ | 1 | 0 | 0 | 0 | 0 |  |
| HAM/TSP#2 | 4381 | chr8_11684946_+ | 0 | 17 | 0 | 0 | 0 |  |
| HAM/TSP#2 | 4382 | chr8_117637658_- | 4 | 0 | 0 | 0 | 0 |  |
| HAM/TSP#2 | 4383 | chr8_117937281_- | 10 | 2 | 0 | 0 | 0 |  |
| HAM/TSP#2 | 4384 | chr8_118794104_- | 3 | 0 | 0 | 0 | 0 |  |
| HAM/TSP#2 | 4385 | chr8_119763070_- | 12 | 0 | 0 | 0 | 0 |  |
| HAM/TSP#2 | 4386 | chr8_120307761_+ | 1 | 0 | 0 | 0 | 0 |  |
| HAM/TSP#2 | 4387 | chr8_1229302_- | 2 | 0 | 0 | 0 | 0 |  |
| HAM/TSP#2 | 4388 | chr8_124917408_+ | 1 | 0 | 0 | 0 | 0 |  |
| HAM/TSP#2 | 4389 | chr8_129101071_- | 1 | 0 | 0 | 0 | 0 |  |
| HAM/TSP#2 | 4390 | chr8_129558820_- | 3 | 0 | 0 | 0 | 0 |  |
| HAM/TSP#2 | 4391 | chr8_13015739_- | 1 | 0 | 0 | 0 | 0 |  |
| HAM/TSP#2 | 4392 | chr8_130983238_- | 1 | 0 | 0 | 0 | 0 |  |
| HAM/TSP#2 | 4393 | chr8_131883481_+ | 0 | 0 | 0 | 0 | 1 |  |
| HAM/TSP#2 | 4394 | chr8_132968751_+ | 1 | 0 | 0 | 0 | 0 |  |
| HAM/TSP#2 | 4395 | chr8_134328209_+ | 0 | 9 | 0 | 0 | 0 |  |
| HAM/TSP#2 | 4396 | chr8_13749585_- | 0 | 1 | 0 | 0 | 0 |  |
| HAM/TSP#2 | 4397 | chr8_137749143_- | 1 | 0 | 0 | 0 | 0 |  |
| HAM/TSP#2 | 4398 | chr8_13794908_- | 3 | 0 | 0 | 0 | 0 |  |
| HAM/TSP#2 | 4399 | chr8_138901636_+ | 1 | 0 | 0 | 0 | 0 |  |
| HAM/TSP#2 | 4400 | chr8_139464599_+ | 1 | 0 | 0 | 0 | 0 |  |
| HAM/TSP#2 | 4401 | chr8_140496666_- | 1 | 0 | 0 | 0 | 0 |  |
| HAM/TSP#2 | 4402 | chr8_141253411_- | 1 | 0 | 0 | 0 | 0 |  |
| HAM/TSP#2 | 4403 | chr8_141828502_- | 2 | 0 | 0 | 0 | 0 |  |
| HAM/TSP#2 | 4404 | chr8_142849282_+ | 5 | 0 | 0 | 0 | 0 |  |
| HAM/TSP#2 | 4405 | chr8_143425200_- | 1 | 0 | 0 | 0 | 0 |  |
| HAM/TSP#2 | 4406 | chr8_145068098_- | 1 | 0 | 0 | 0 | 0 |  |
| HAM/TSP#2 | 4407 | chr8_1451490_+ | 3 | 0 | 0 | 0 | 0 |  |
| HAM/TSP#2 | 4408 | chr8_16821192_- | 1 | 0 | 0 | 0 | 0 |  |
| HAM/TSP#2 | 4409 | chr8_18119455_+ | 4 | 0 | 0 | 0 | 0 |  |
| HAM/TSP#2 | 4410 | chr8_19160944_+ | 1 | 0 | 0 | 0 | 0 |  |
| HAM/TSP#2 | 4411 | chr8_19341697_+ | 1 | 0 | 0 | 0 | 0 |  |
| HAM/TSP#2 | 4412 | chr8_20435393_+ | 1 | 0 | 0 | 0 | 0 |  |
| HAM/TSP#2 | 4413 | chr8_2082948_+ | 2 | 0 | 0 | 0 | 0 |  |
| HAM/TSP#2 | 4414 | chr8_22728520_+ | 0 | 2 | 0 | 0 | 0 |  |
| HAM/TSP#2 | 4415 | chr8_22761153_- | 1 | 0 | 0 | 0 | 0 |  |
| HAM/TSP#2 | 4416 | chr8_23243884_+ | 1 | 0 | 0 | 0 | 0 |  |
| HAM/TSP#2 | 4417 | chr8_23466876_+ | 8 | 0 | 0 | 0 | 0 |  |
| HAM/TSP#2 | 4418 | chr8_25231326_+ | 1 | 0 | 0 | 0 | 0 |  |
| HAM/TSP#2 | 4419 | chr8_27376065_- | 0 | 2 | 0 | 0 | 0 |  |
| HAM/TSP#2 | 4420 | chr8_28187480_+ | 0 | 1 | 0 | 0 | 0 |  |
| HAM/TSP#2 | 4421 | chr8_32965538_+ | 3 | 0 | 0 | 0 | 0 |  |
| HAM/TSP#2 | 4422 | chr8_33277948_- | 4 | 1 | 0 | 0 | 5 |  |
| HAM/TSP#2 | 4423 | chr8_33372903_- | 2 | 0 | 0 | 0 | 0 |  |
| HAM/TSP#2 | 4424 | chr8_34123928_+ | 1 | 0 | 0 | 0 | 0 |  |
| HAM/TSP#2 | 4425 | chr8_34622266_+ | 2 | 0 | 0 | 0 | 0 |  |
| HAM/TSP#2 | 4426 | chr8_35336959_- | 4 | 0 | 0 | 0 | 0 |  |
| HAM/TSP#2 | 4427 | chr8_35373152_+ | 25 | 0 | 0 | 0 | 0 |  |
| HAM/TSP#2 | 4428 | chr8_35757534_- | 4 | 0 | 0 | 0 | 0 |  |
| HAM/TSP#2 | 4429 | chr8_3683405_+ | 1 | 0 | 0 | 0 | 0 |  |
| HAM/TSP#2 | 4430 | chr8_37078508_+ | 5 | 0 | 0 | 0 | 0 |  |
| HAM/TSP#2 | 4431 | chr8_37891777_+ | 1 | 0 | 0 | 0 | 0 |  |
| HAM/TSP#2 | 4432 | chr8_38952749_+ | 1 | 0 | 0 | 0 | 0 |  |
| HAM/TSP#2 | 4433 | chr8_40407964_+ | 2 | 70 | 0 | 0 | 0 |  |
| HAM/TSP#2 | 4434 | chr8_41105927_+ | 4 | 0 | 0 | 0 | 0 |  |
| HAM/TSP#2 | 4435 | chr8_41770136_+ | 3 | 0 | 0 | 0 | 0 |  |
| HAM/TSP#2 | 4436 | chr8_41935135_- | 1 | 0 | 0 | 0 | 0 |  |
| HAM/TSP#2 | 4437 | chr8_42191088_+ | 5 | 0 | 0 | 0 | 0 |  |
| HAM/TSP#2 | 4438 | chr8_43080995_+ | 5 | 0 | 0 | 0 | 0 |  |
| HAM/TSP#2 | 4439 | chr8_43255398_+ | 1 | 0 | 0 | 0 | 0 |  |
| HAM/TSP#2 | 4440 | chr8_43268723_- | 0 | 1 | 0 | 0 | 0 |  |
| HAM/TSP#2 | 4441 | chr8_43364894_+ | 1 | 0 | 0 | 0 | 0 |  |
| HAM/TSP#2 | 4442 | chr8_43462672_+ | 1 | 0 | 0 | 0 | 0 |  |
| HAM/TSP#2 | 4443 | chr8_43930580_- | 2 | 0 | 0 | 0 | 0 |  |
| HAM/TSP#2 | 4444 | chr8_46225504_+ | 2 | 0 | 0 | 0 | 0 |  |
| HAM/TSP#2 | 4445 | chr8_46493949_- | 1 | 0 | 0 | 0 | 0 |  |
| HAM/TSP#2 | 4446 | chr8_46965017_- | 1 | 0 | 0 | 0 | 0 |  |
| HAM/TSP#2 | 4447 | chr8_47001015_+ | 3 | 0 | 0 | 0 | 0 |  |
| HAM/TSP#2 | 4448 | chr8_47101762_+ | 8 | 1 | 0 | 0 | 0 |  |
| HAM/TSP#2 | 4449 | chr8_47139613_+ | 1 | 0 | 0 | 0 | 0 |  |
| HAM/TSP#2 | 4450 | chr8_51612942_- | 5 | 0 | 0 | 0 | 1 |  |
| HAM/TSP#2 | 4451 | chr8_52071355_- | 25 | 0 | 0 | 0 | 0 |  |
| HAM/TSP#2 | 4452 | chr8_52421878_+ | 1 | 0 | 0 | 0 | 0 |  |
| HAM/TSP#2 | 4453 | chr8_53227890_- | 21 | 0 | 0 | 0 | 0 |  |
| HAM/TSP#2 | 4454 | chr8_53983903_- | 1 | 0 | 0 | 0 | 0 |  |
| HAM/TSP#2 | 4455 | chr8_55119151_- | 5 | 0 | 0 | 0 | 0 |  |
| HAM/TSP#2 | 4456 | chr8_55137023_+ | 0 | 1 | 0 | 0 | 0 |  |
| HAM/TSP#2 | 4457 | chr8_55632611_+ | 1 | 0 | 0 | 0 | 0 |  |
| HAM/TSP#2 | 4458 | chr8_556815_- | 1 | 0 | 0 | 0 | 0 |  |
| HAM/TSP#2 | 4459 | chr8_55956592_+ | 4 | 0 | 0 | 0 | 0 |  |
| HAM/TSP#2 | 4460 | chr8_56144629_+ | 3 | 0 | 0 | 0 | 9 |  |
| HAM/TSP#2 | 4461 | chr8_56847113_- | 1 | 0 | 0 | 0 | 0 |  |
| HAM/TSP#2 | 4462 | chr8_5779139_- | 1 | 0 | 0 | 0 | 0 |  |
| HAM/TSP#2 | 4463 | chr8_57873531_- | 2 | 0 | 0 | 0 | 0 |  |
| HAM/TSP#2 | 4464 | chr8_59006473_- | 7 | 0 | 0 | 0 | 0 |  |
| HAM/TSP#2 | 4465 | chr8_59078840_+ | 3 | 0 | 0 | 0 | 0 |  |
| HAM/TSP#2 | 4466 | chr8_59110088_+ | 3 | 1 | 0 | 0 | 0 |  |
| HAM/TSP#2 | 4467 | chr8_6009622_+ | 3 | 0 | 0 | 0 | 0 |  |
| HAM/TSP#2 | 4468 | chr8_6029589_- | 1 | 0 | 0 | 0 | 0 |  |
| HAM/TSP#2 | 4469 | chr8_61936291_+ | 1 | 0 | 0 | 0 | 0 |  |
| HAM/TSP#2 | 4470 | chr8_62272581_+ | 3 | 0 | 0 | 0 | 0 |  |
| HAM/TSP#2 | 4471 | chr8_62743126_- | 0 | 1 | 0 | 0 | 0 |  |
| HAM/TSP#2 | 4472 | chr8_64163773_- | 5 | 0 | 0 | 0 | 0 |  |
| HAM/TSP#2 | 4473 | chr8_64209671_+ | 1 | 0 | 0 | 0 | 0 |  |
| HAM/TSP#2 | 4474 | chr8_6437956_+ | 2 | 0 | 0 | 0 | 0 |  |
| HAM/TSP#2 | 4475 | chr8_6479123_+ | 5 | 0 | 0 | 0 | 0 |  |
| HAM/TSP#2 | 4476 | chr8_67314580_+ | 1 | 0 | 0 | 0 | 0 |  |
| HAM/TSP#2 | 4477 | chr8_67578625_- | 1 | 0 | 0 | 0 | 0 |  |
| HAM/TSP#2 | 4478 | chr8_6834382_+ | 1 | 0 | 0 | 0 | 0 |  |
| HAM/TSP#2 | 4479 | chr8_70559341_- | 0 | 0 | 0 | 0 | 1 |  |
| HAM/TSP#2 | 4480 | chr8_70562026_+ | 1 | 1 | 0 | 0 | 0 |  |
| HAM/TSP#2 | 4481 | chr8_73399658_- | 1 | 0 | 0 | 0 | 0 |  |
| HAM/TSP#2 | 4482 | chr8_75109734_+ | 2 | 0 | 0 | 0 | 0 |  |
| HAM/TSP#2 | 4483 | chr8_76086598_+ | 0 | 5 | 0 | 0 | 0 |  |
| HAM/TSP#2 | 4484 | chr8_77222706_- | 20 | 0 | 0 | 0 | 0 |  |
| HAM/TSP#2 | 4485 | chr8_78357258_- | 1 | 0 | 0 | 0 | 0 |  |
| HAM/TSP#2 | 4486 | chr8_80685458_- | 1 | 0 | 0 | 0 | 0 |  |
| HAM/TSP#2 | 4487 | chr8_81104750_+ | 0 | 16 | 0 | 0 | 0 |  |
| HAM/TSP#2 | 4488 | chr8_82145003_- | 2 | 0 | 0 | 0 | 0 |  |
| HAM/TSP#2 | 4489 | chr8_82784962_- | 4 | 0 | 0 | 0 | 0 |  |
| HAM/TSP#2 | 4490 | chr8_83711774_- | 1 | 0 | 0 | 0 | 0 |  |
| HAM/TSP#2 | 4491 | chr8_8377310_+ | 1 | 0 | 0 | 0 | 0 |  |
| HAM/TSP#2 | 4492 | chr8_84183792_+ | 0 | 1 | 0 | 0 | 0 |  |
| HAM/TSP#2 | 4493 | chr8_84549709_- | 0 | 1 | 0 | 0 | 0 |  |
| HAM/TSP#2 | 4494 | chr8_84647468_- | 1 | 0 | 0 | 0 | 0 |  |
| HAM/TSP#2 | 4495 | chr8_85125331_+ | 3 | 0 | 0 | 0 | 0 |  |
| HAM/TSP#2 | 4496 | chr8_88215045_- | 1 | 0 | 0 | 0 | 0 |  |
| HAM/TSP#2 | 4497 | chr8_88265102_- | 47 | 1 | 0 | 0 | 5 |  |
| HAM/TSP#2 | 4498 | chr8_88819170_+ | 1 | 0 | 0 | 0 | 0 |  |
| HAM/TSP#2 | 4499 | chr8_89824646_+ | 1 | 0 | 0 | 0 | 0 |  |
| HAM/TSP#2 | 4500 | chr8_90100975_+ | 1 | 0 | 0 | 0 | 0 |  |
| HAM/TSP#2 | 4501 | chr8_90225525_- | 4 | 0 | 0 | 0 | 0 |  |
| HAM/TSP#2 | 4502 | chr8_91998947_- | 0 | 0 | 0 | 0 | 1 |  |
| HAM/TSP#2 | 4503 | chr8_93270740_- | 1 | 0 | 0 | 0 | 0 |  |
| HAM/TSP#2 | 4504 | chr8_93740203_+ | 4 | 0 | 0 | 0 | 0 |  |
| HAM/TSP#2 | 4505 | chr8_94856460_- | 1 | 0 | 0 | 0 | 0 |  |
| HAM/TSP#2 | 4506 | chr8_96248279_+ | 6 | 0 | 0 | 0 | 0 |  |
| HAM/TSP#2 | 4507 | chr8_96340615_+ | 1 | 0 | 0 | 0 | 0 |  |
| HAM/TSP#2 | 4508 | chr8_98439278_+ | 1 | 0 | 0 | 0 | 0 |  |
| HAM/TSP#2 | 4509 | chr8_98518237_+ | 1 | 0 | 0 | 0 | 0 |  |
| HAM/TSP#2 | 4510 | chr9_100133814_- | 0 | 1 | 0 | 0 | 0 |  |
| HAM/TSP#2 | 4511 | chr9_100332945_- | 4 | 0 | 0 | 0 | 0 |  |
| HAM/TSP#2 | 4512 | chr9_104393779_- | 1 | 0 | 0 | 0 | 0 |  |
| HAM/TSP#2 | 4513 | chr9_104538412_- | 6 | 0 | 0 | 0 | 0 |  |
| HAM/TSP#2 | 4514 | chr9_105113743_+ | 3 | 0 | 0 | 0 | 0 |  |
| HAM/TSP#2 | 4515 | chr9_106065653_- | 19 | 0 | 0 | 0 | 0 |  |
| HAM/TSP#2 | 4516 | chr9_10797162_+ | 4 | 0 | 0 | 0 | 0 |  |
| HAM/TSP#2 | 4517 | chr9_108806436_+ | 1 | 0 | 0 | 0 | 0 |  |
| HAM/TSP#2 | 4518 | chr9_108930905_- | 1 | 0 | 0 | 0 | 0 |  |
| HAM/TSP#2 | 4519 | chr9_109648415_+ | 1 | 0 | 0 | 0 | 0 |  |
| HAM/TSP#2 | 4520 | chr9_110639060_+ | 1 | 0 | 0 | 0 | 0 |  |
| HAM/TSP#2 | 4521 | chr9_110812710_- | 2 | 0 | 0 | 0 | 0 |  |
| HAM/TSP#2 | 4522 | chr9_111735010_- | 1 | 0 | 0 | 0 | 0 |  |
| HAM/TSP#2 | 4523 | chr9_113147176_- | 1 | 0 | 0 | 0 | 0 |  |
| HAM/TSP#2 | 4524 | chr9_11540429_- | 1 | 0 | 0 | 0 | 0 |  |
| HAM/TSP#2 | 4525 | chr9_119179851_- | 1 | 0 | 0 | 0 | 0 |  |
| HAM/TSP#2 | 4526 | chr9_120110708_- | 1 | 0 | 0 | 0 | 0 |  |
| HAM/TSP#2 | 4527 | chr9_120893721_+ | 4 | 0 | 0 | 0 | 0 |  |
| HAM/TSP#2 | 4528 | chr9_12152098_+ | 4 | 2 | 0 | 0 | 0 |  |
| HAM/TSP#2 | 4529 | chr9_121751742_- | 2 | 0 | 0 | 0 | 0 |  |
| HAM/TSP#2 | 4530 | chr9_123854914_+ | 0 | 1 | 0 | 0 | 0 |  |
| HAM/TSP#2 | 4531 | chr9_124433872_- | 1 | 0 | 0 | 0 | 0 |  |
| HAM/TSP#2 | 4532 | chr9_125141225_+ | 6 | 0 | 0 | 0 | 0 |  |
| HAM/TSP#2 | 4533 | chr9_125658686_+ | 2 | 0 | 0 | 0 | 0 |  |
| HAM/TSP#2 | 4534 | chr9_125667434_- | 1 | 0 | 0 | 0 | 0 |  |
| HAM/TSP#2 | 4535 | chr9_126527328_- | 0 | 0 | 0 | 0 | 1 |  |
| HAM/TSP#2 | 4536 | chr9_130254361_+ | 6 | 0 | 0 | 0 | 0 |  |
| HAM/TSP#2 | 4537 | chr9_130908336_- | 1 | 0 | 0 | 0 | 0 |  |
| HAM/TSP#2 | 4538 | chr9_131969369_+ | 1 | 0 | 0 | 0 | 0 |  |
| HAM/TSP#2 | 4539 | chr9_133141637_- | 1 | 0 | 0 | 0 | 0 |  |
| HAM/TSP#2 | 4540 | chr9_133583833_+ | 1 | 0 | 0 | 0 | 0 |  |
| HAM/TSP#2 | 4541 | chr9_134378361_- | 1 | 0 | 0 | 0 | 0 |  |
| HAM/TSP#2 | 4542 | chr9_134818936_- | 1 | 0 | 0 | 0 | 0 |  |
| HAM/TSP#2 | 4543 | chr9_135474489_- | 1 | 0 | 0 | 0 | 0 |  |
| HAM/TSP#2 | 4544 | chr9_135606683_- | 0 | 0 | 0 | 1 | 0 |  |
| HAM/TSP#2 | 4545 | chr9_135606694_- | 8 | 0 | 0 | 0 | 0 |  |
| HAM/TSP#2 | 4546 | chr9_136833379_- | 0 | 0 | 0 | 0 | 1 |  |
| HAM/TSP#2 | 4547 | chr9_137548232_+ | 1 | 0 | 0 | 0 | 0 |  |
| HAM/TSP#2 | 4548 | chr9_137680570_- | 15 | 0 | 0 | 0 | 0 |  |
| HAM/TSP#2 | 4549 | chr9_14043300_- | 0 | 2 | 0 | 0 | 0 |  |
| HAM/TSP#2 | 4550 | chr9_15297425_- | 1 | 0 | 0 | 0 | 0 |  |
| HAM/TSP#2 | 4551 | chr9_15470071_+ | 1 | 0 | 0 | 0 | 0 |  |
| HAM/TSP#2 | 4552 | chr9_15578812_- | 1 | 0 | 0 | 0 | 0 |  |
| HAM/TSP#2 | 4553 | chr9_16585029_+ | 1 | 0 | 0 | 0 | 0 |  |
| HAM/TSP#2 | 4554 | chr9_17169812_+ | 1 | 0 | 0 | 0 | 0 |  |
| HAM/TSP#2 | 4555 | chr9_1795019_+ | 1 | 0 | 0 | 0 | 0 |  |
| HAM/TSP#2 | 4556 | chr9_18307683_- | 18 | 0 | 0 | 0 | 0 |  |
| HAM/TSP#2 | 4557 | chr9_18574820_+ | 1 | 0 | 0 | 0 | 0 |  |
| HAM/TSP#2 | 4558 | chr9_19067277_+ | 1 | 0 | 0 | 0 | 0 |  |
| HAM/TSP#2 | 4559 | chr9_19448961_- | 1 | 0 | 0 | 0 | 0 |  |
| HAM/TSP#2 | 4560 | chr9_20819246_+ | 9 | 0 | 0 | 0 | 0 |  |
| HAM/TSP#2 | 4561 | chr9_2136804_- | 1 | 0 | 0 | 0 | 0 |  |
| HAM/TSP#2 | 4562 | chr9_2156095_+ | 2 | 0 | 0 | 0 | 0 |  |
| HAM/TSP#2 | 4563 | chr9_21745151_- | 3 | 0 | 0 | 0 | 0 |  |
| HAM/TSP#2 | 4564 | chr9_22215151_- | 3 | 0 | 0 | 0 | 0 |  |
| HAM/TSP#2 | 4565 | chr9_23211447_+ | 4 | 0 | 0 | 0 | 0 |  |
| HAM/TSP#2 | 4566 | chr9_23386410_+ | 3 | 0 | 0 | 0 | 0 |  |
| HAM/TSP#2 | 4567 | chr9_23387491_+ | 1 | 0 | 0 | 0 | 0 |  |
| HAM/TSP#2 | 4568 | chr9_23683819_+ | 3 | 0 | 0 | 0 | 0 |  |
| HAM/TSP#2 | 4569 | chr9_24462437_+ | 17 | 0 | 0 | 0 | 0 |  |
| HAM/TSP#2 | 4570 | chr9_26832229_- | 0 | 0 | 4 | 0 | 0 |  |
| HAM/TSP#2 | 4571 | chr9_2691482_+ | 1 | 0 | 0 | 0 | 0 |  |
| HAM/TSP#2 | 4572 | chr9_28399367_+ | 2 | 0 | 0 | 0 | 0 |  |
| HAM/TSP#2 | 4573 | chr9_28963994_+ | 1 | 0 | 0 | 0 | 0 |  |
| HAM/TSP#2 | 4574 | chr9_29414489_+ | 1 | 0 | 0 | 0 | 0 |  |
| HAM/TSP#2 | 4575 | chr9_31289373_- | 1 | 0 | 0 | 0 | 0 |  |
| HAM/TSP#2 | 4576 | chr9_35698019_+ | 2 | 0 | 0 | 0 | 0 |  |
| HAM/TSP#2 | 4577 | chr9_35827152_- | 2 | 0 | 0 | 0 | 0 |  |
| HAM/TSP#2 | 4578 | chr9_36575898_- | 2 | 0 | 0 | 0 | 0 |  |
| HAM/TSP#2 | 4579 | chr9_36744792_- | 1 | 0 | 0 | 0 | 0 |  |
| HAM/TSP#2 | 4580 | chr9_37847565_- | 5 | 0 | 0 | 0 | 0 |  |
| HAM/TSP#2 | 4581 | chr9_41037631_- | 10 | 0 | 0 | 0 | 0 |  |
| HAM/TSP#2 | 4582 | chr9_41240530_- | 1 | 0 | 0 | 0 | 0 |  |
| HAM/TSP#2 | 4583 | chr9_41263395_- | 4 | 0 | 0 | 0 | 0 |  |
| HAM/TSP#2 | 4584 | chr9_42364971_- | 1 | 0 | 0 | 0 | 0 |  |
| HAM/TSP#2 | 4585 | chr9_42952934_- | 1 | 0 | 0 | 0 | 0 |  |
| HAM/TSP#2 | 4586 | chr9_44875735_+ | 15 | 0 | 0 | 0 | 0 |  |
| HAM/TSP#2 | 4587 | chr9_45322854_+ | 0 | 0 | 0 | 0 | 1 |  |
| HAM/TSP#2 | 4588 | chr9_4621156_- | 1 | 0 | 0 | 0 | 0 |  |
| HAM/TSP#2 | 4589 | chr9_5505112_- | 2 | 0 | 0 | 0 | 0 |  |
| HAM/TSP#2 | 4590 | chr9_6107025_- | 0 | 2 | 0 | 0 | 0 |  |
| HAM/TSP#2 | 4591 | chr9_61380106_+ | 1 | 0 | 0 | 0 | 0 |  |
| HAM/TSP#2 | 4592 | chr9_63898636_- | 1 | 0 | 0 | 0 | 0 |  |
| HAM/TSP#2 | 4593 | chr9_64031763_+ | 1 | 0 | 0 | 0 | 0 |  |
| HAM/TSP#2 | 4594 | chr9_64077317_- | 4 | 0 | 0 | 0 | 0 |  |
| HAM/TSP#2 | 4595 | chr9_64952447_+ | 0 | 0 | 1 | 0 | 0 |  |
| HAM/TSP#2 | 4596 | chr9_6682373_- | 3 | 0 | 0 | 0 | 0 |  |
| HAM/TSP#2 | 4597 | chr9_6802346_- | 2 | 0 | 0 | 0 | 0 |  |
| HAM/TSP#2 | 4598 | chr9_68395127_- | 3 | 0 | 0 | 0 | 0 |  |
| HAM/TSP#2 | 4599 | chr9_68471589_- | 1 | 0 | 0 | 0 | 0 |  |
| HAM/TSP#2 | 4600 | chr9_69187500_+ | 5 | 0 | 0 | 0 | 0 |  |
| HAM/TSP#2 | 4601 | chr9_69466567_- | 1 | 0 | 0 | 0 | 0 |  |
| HAM/TSP#2 | 4602 | chr9_70382874_- | 0 | 1 | 0 | 0 | 0 |  |
| HAM/TSP#2 | 4603 | chr9_7317761_- | 0 | 0 | 0 | 1 | 0 |  |
| HAM/TSP#2 | 4604 | chr9_75105932_+ | 1 | 0 | 0 | 0 | 0 |  |
| HAM/TSP#2 | 4605 | chr9_76704497_- | 1 | 0 | 0 | 0 | 0 |  |
| HAM/TSP#2 | 4606 | chr9_77673886_- | 1 | 0 | 0 | 0 | 0 |  |
| HAM/TSP#2 | 4607 | chr9_78086235_- | 1 | 0 | 0 | 0 | 0 |  |
| HAM/TSP#2 | 4608 | chr9_78276093_- | 3 | 0 | 0 | 0 | 0 |  |
| HAM/TSP#2 | 4609 | chr9_79241992_- | 1 | 0 | 0 | 0 | 0 |  |
| HAM/TSP#2 | 4610 | chr9_80911773_- | 1 | 0 | 0 | 0 | 0 |  |
| HAM/TSP#2 | 4611 | chr9_81661790_+ | 1 | 0 | 0 | 0 | 0 |  |
| HAM/TSP#2 | 4612 | chr9_81856080_- | 1 | 0 | 0 | 0 | 0 |  |
| HAM/TSP#2 | 4613 | chr9_82084358_+ | 1 | 0 | 0 | 0 | 0 |  |
| HAM/TSP#2 | 4614 | chr9_82173148_- | 1 | 0 | 0 | 0 | 0 |  |
| HAM/TSP#2 | 4615 | chr9_82797846_+ | 4 | 0 | 0 | 0 | 0 |  |
| HAM/TSP#2 | 4616 | chr9_84866904_- | 1 | 0 | 0 | 0 | 0 |  |
| HAM/TSP#2 | 4617 | chr9_88926631_+ | 4 | 0 | 0 | 0 | 0 |  |
| HAM/TSP#2 | 4618 | chr9_89634472_- | 1 | 0 | 0 | 0 | 0 |  |
| HAM/TSP#2 | 4619 | chr9_89901576_- | 0 | 0 | 0 | 3 | 0 |  |
| HAM/TSP#2 | 4620 | chr9_90338143_- | 0 | 1 | 0 | 0 | 0 |  |
| HAM/TSP#2 | 4621 | chr9_91013912_- | 1 | 0 | 0 | 0 | 0 |  |
| HAM/TSP#2 | 4622 | chr9_91541916_+ | 2 | 0 | 0 | 0 | 0 |  |
| HAM/TSP#2 | 4623 | chr9_91816064_- | 1 | 0 | 0 | 0 | 0 |  |
| HAM/TSP#2 | 4624 | chr9_92613289_+ | 1 | 0 | 0 | 0 | 0 |  |
| HAM/TSP#2 | 4625 | chr9_92690024_- | 1 | 0 | 0 | 0 | 0 |  |
| HAM/TSP#2 | 4626 | chr9_93400387_- | 1 | 0 | 0 | 0 | 0 |  |
| HAM/TSP#2 | 4627 | chr9_93912503_- | 10 | 0 | 0 | 0 | 0 |  |
| HAM/TSP#2 | 4628 | chr9_94021154_- | 1 | 0 | 0 | 0 | 0 |  |
| HAM/TSP#2 | 4629 | chr9_94686255_- | 1 | 0 | 0 | 0 | 0 |  |
| HAM/TSP#2 | 4630 | chr9_94956200_- | 1 | 2 | 0 | 0 | 0 |  |
| HAM/TSP#2 | 4631 | chr9_97013946_- | 2 | 0 | 0 | 0 | 0 |  |
| HAM/TSP#2 | 4632 | chr9_97292778_- | 4 | 2 | 0 | 0 | 0 |  |
| HAM/TSP#2 | 4633 | chr9_97770784_+ | 1 | 0 | 0 | 0 | 0 |  |
| HAM/TSP#2 | 4634 | chr9_98248935_+ | 1 | 0 | 0 | 0 | 0 |  |
| HAM/TSP#2 | 4635 | chr10_100104416_+ | 3 | 0 | 0 | 0 | 0 |  |
| HAM/TSP#2 | 4636 | chr10_102610387_- | 1 | 0 | 0 | 0 | 0 |  |
| HAM/TSP#2 | 4637 | chr10_102991073_- | 1 | 0 | 0 | 0 | 0 |  |
| HAM/TSP#2 | 4638 | chr10_103072371_+ | 1 | 0 | 0 | 0 | 0 |  |
| HAM/TSP#2 | 4639 | chr10_103469414_+ | 1 | 1 | 0 | 0 | 0 |  |
| HAM/TSP#2 | 4640 | chr10_104170004_+ | 0 | 6 | 0 | 0 | 0 |  |
| HAM/TSP#2 | 4641 | chr10_104349203_- | 1 | 0 | 0 | 0 | 0 |  |
| HAM/TSP#2 | 4642 | chr10_105163686_- | 1 | 0 | 0 | 0 | 0 |  |
| HAM/TSP#2 | 4643 | chr10_106351262_- | 1 | 0 | 0 | 0 | 0 |  |
| HAM/TSP#2 | 4644 | chr10_106402476_+ | 3 | 0 | 0 | 0 | 0 |  |
| HAM/TSP#2 | 4645 | chr10_106780515_+ | 1 | 0 | 0 | 0 | 0 |  |
| HAM/TSP#2 | 4646 | chr10_10686912_- | 1 | 0 | 0 | 0 | 0 |  |
| HAM/TSP#2 | 4647 | chr10_108544368_- | 5 | 0 | 0 | 0 | 0 |  |
| HAM/TSP#2 | 4648 | chr10_1087270_+ | 1 | 0 | 0 | 0 | 0 |  |
| HAM/TSP#2 | 4649 | chr10_109116863_+ | 1 | 0 | 0 | 0 | 0 |  |
| HAM/TSP#2 | 4650 | chr10_110782173_- | 1 | 0 | 0 | 0 | 0 |  |
| HAM/TSP#2 | 4651 | chr10_110955492_+ | 1 | 0 | 0 | 0 | 0 |  |
| HAM/TSP#2 | 4652 | chr10_11218097_- | 0 | 0 | 0 | 0 | 4 |  |
| HAM/TSP#2 | 4653 | chr10_114581445_- | 1 | 0 | 0 | 0 | 0 |  |
| HAM/TSP#2 | 4654 | chr10_115047710_- | 1 | 0 | 0 | 0 | 0 |  |
| HAM/TSP#2 | 4655 | chr10_115047711_- | 1 | 0 | 0 | 0 | 0 |  |
| HAM/TSP#2 | 4656 | chr10_116146351_+ | 0 | 1 | 0 | 0 | 0 |  |
| HAM/TSP#2 | 4657 | chr10_118304067_+ | 0 | 2 | 0 | 0 | 0 |  |
| HAM/TSP#2 | 4658 | chr10_119572826_+ | 5 | 0 | 0 | 0 | 0 |  |
| HAM/TSP#2 | 4659 | chr10_121272621_- | 1 | 0 | 0 | 0 | 0 |  |
| HAM/TSP#2 | 4660 | chr10_122142892_+ | 1 | 0 | 0 | 0 | 0 |  |
| HAM/TSP#2 | 4661 | chr10_122486570_+ | 1 | 1 | 0 | 0 | 0 |  |
| HAM/TSP#2 | 4662 | chr10_124691766_+ | 1 | 0 | 0 | 0 | 0 |  |
| HAM/TSP#2 | 4663 | chr10_124912910_+ | 1 | 0 | 0 | 0 | 0 |  |
| HAM/TSP#2 | 4664 | chr10_126844288_- | 0 | 1 | 0 | 0 | 0 |  |
| HAM/TSP#2 | 4665 | chr10_126855738_+ | 1 | 0 | 0 | 0 | 0 |  |
| HAM/TSP#2 | 4666 | chr10_127379623_+ | 6 | 0 | 0 | 0 | 0 |  |
| HAM/TSP#2 | 4667 | chr10_127568583_+ | 1 | 0 | 0 | 0 | 0 |  |
| HAM/TSP#2 | 4668 | chr10_127757678_- | 1 | 0 | 0 | 0 | 0 |  |
| HAM/TSP#2 | 4669 | chr10_129003033_+ | 0 | 1 | 0 | 0 | 0 |  |
| HAM/TSP#2 | 4670 | chr10_129963353_- | 1 | 0 | 0 | 0 | 0 |  |
| HAM/TSP#2 | 4671 | chr10_130106965_+ | 1 | 0 | 0 | 0 | 0 |  |
| HAM/TSP#2 | 4672 | chr10_130180530_- | 0 | 1 | 0 | 0 | 0 |  |
| HAM/TSP#2 | 4673 | chr10_131864039_- | 1 | 0 | 0 | 0 | 0 |  |
| HAM/TSP#2 | 4674 | chr10_132169006_+ | 0 | 0 | 0 | 0 | 2 |  |
| HAM/TSP#2 | 4675 | chr10_132578217_+ | 0 | 0 | 0 | 0 | 7 |  |
| HAM/TSP#2 | 4676 | chr10_132837932_+ | 0 | 0 | 0 | 1 | 0 |  |
| HAM/TSP#2 | 4677 | chr10_133309598_- | 0 | 0 | 0 | 0 | 1 |  |
| HAM/TSP#2 | 4678 | chr10_13529832_- | 3 | 0 | 0 | 0 | 0 |  |
| HAM/TSP#2 | 4679 | chr10_14443505_- | 1 | 0 | 0 | 0 | 0 |  |
| HAM/TSP#2 | 4680 | chr10_14572471_- | 1 | 0 | 0 | 0 | 0 |  |
| HAM/TSP#2 | 4681 | chr10_15170191_- | 10 | 0 | 0 | 0 | 0 |  |
| HAM/TSP#2 | 4682 | chr10_15820251_- | 0 | 1 | 0 | 0 | 0 |  |
| HAM/TSP#2 | 4683 | chr10_16484090_+ | 3 | 0 | 0 | 0 | 0 |  |
| HAM/TSP#2 | 4684 | chr10_17371093_+ | 1 | 0 | 0 | 0 | 0 |  |
| HAM/TSP#2 | 4685 | chr10_17392266_+ | 1 | 0 | 0 | 0 | 0 |  |
| HAM/TSP#2 | 4686 | chr10_17444516_+ | 10 | 1 | 0 | 0 | 1 |  |
| HAM/TSP#2 | 4687 | chr10_17629822_+ | 0 | 0 | 0 | 0 | 1 |  |
| HAM/TSP#2 | 4688 | chr10_17825417_- | 1 | 0 | 0 | 0 | 0 |  |
| HAM/TSP#2 | 4689 | chr10_18109756_- | 1 | 0 | 0 | 0 | 0 |  |
| HAM/TSP#2 | 4690 | chr10_21378318_+ | 1 | 0 | 0 | 0 | 0 |  |
| HAM/TSP#2 | 4691 | chr10_21509207_- | 1 | 0 | 0 | 0 | 0 |  |
| HAM/TSP#2 | 4692 | chr10_21731334_+ | 19 | 0 | 0 | 0 | 0 |  |
| HAM/TSP#2 | 4693 | chr10_22306279_- | 1 | 0 | 0 | 0 | 0 |  |
| HAM/TSP#2 | 4694 | chr10_22454233_- | 2 | 0 | 0 | 0 | 0 |  |
| HAM/TSP#2 | 4695 | chr10_23118364_+ | 0 | 1 | 0 | 0 | 0 |  |
| HAM/TSP#2 | 4696 | chr10_23930421_- | 0 | 1 | 0 | 0 | 0 |  |
| HAM/TSP#2 | 4697 | chr10_25446757_- | 2 | 0 | 0 | 0 | 0 |  |
| HAM/TSP#2 | 4698 | chr10_26673835_+ | 3 | 0 | 0 | 0 | 0 |  |
| HAM/TSP#2 | 4699 | chr10_27847034_- | 1 | 0 | 0 | 0 | 0 |  |
| HAM/TSP#2 | 4700 | chr10_30466784_- | 7 | 0 | 0 | 0 | 0 |  |
| HAM/TSP#2 | 4701 | chr10_30833068_- | 1 | 0 | 0 | 0 | 0 |  |
| HAM/TSP#2 | 4702 | chr10_31876156_+ | 1 | 0 | 0 | 0 | 0 |  |
| HAM/TSP#2 | 4703 | chr10_32017081_- | 0 | 40 | 0 | 0 | 0 |  |
| HAM/TSP#2 | 4704 | chr10_32243053_- | 0 | 0 | 0 | 1 | 0 |  |
| HAM/TSP#2 | 4705 | chr10_32460845_- | 17 | 0 | 0 | 0 | 0 |  |
| HAM/TSP#2 | 4706 | chr10_32489773_+ | 1 | 0 | 0 | 0 | 0 |  |
| HAM/TSP#2 | 4707 | chr10_32506181_+ | 0 | 0 | 0 | 1 | 0 |  |
| HAM/TSP#2 | 4708 | chr10_32544434_- | 2 | 9 | 11 | 11 | 14 |  |
| HAM/TSP#2 | 4709 | chr10_34078700_+ | 0 | 1 | 0 | 0 | 0 |  |
| HAM/TSP#2 | 4710 | chr10_34333042_+ | 4 | 0 | 0 | 0 | 0 |  |
| HAM/TSP#2 | 4711 | chr10_34389285_- | 0 | 5 | 0 | 0 | 0 |  |
| HAM/TSP#2 | 4712 | chr10_35068286_- | 1 | 0 | 0 | 0 | 0 |  |
| HAM/TSP#2 | 4713 | chr10_37721650_+ | 1 | 0 | 0 | 0 | 0 |  |
| HAM/TSP#2 | 4714 | chr10_38014393_- | 4 | 0 | 0 | 0 | 0 |  |
| HAM/TSP#2 | 4715 | chr10_38048107_- | 2 | 0 | 0 | 0 | 0 |  |
| HAM/TSP#2 | 4716 | chr10_38677837_+ | 2 | 0 | 0 | 0 | 0 |  |
| HAM/TSP#2 | 4717 | chr10_38902422_+ | 1 | 0 | 0 | 0 | 0 |  |
| HAM/TSP#2 | 4718 | chr10_42073031_+ | 1 | 0 | 0 | 0 | 0 |  |
| HAM/TSP#2 | 4719 | chr10_43447533_- | 1 | 0 | 0 | 0 | 0 |  |
| HAM/TSP#2 | 4720 | chr10_43648192_+ | 2 | 0 | 0 | 0 | 0 |  |
| HAM/TSP#2 | 4721 | chr10_43897622_- | 1 | 0 | 0 | 0 | 0 |  |
| HAM/TSP#2 | 4722 | chr10_44964530_+ | 2 | 0 | 0 | 0 | 0 |  |
| HAM/TSP#2 | 4723 | chr10_45559095_- | 1 | 0 | 0 | 0 | 0 |  |
| HAM/TSP#2 | 4724 | chr10_46091180_+ | 1 | 0 | 0 | 0 | 0 |  |
| HAM/TSP#2 | 4725 | chr10_48187113_+ | 1 | 0 | 0 | 0 | 0 |  |
| HAM/TSP#2 | 4726 | chr10_48204555_- | 1 | 0 | 0 | 0 | 0 |  |
| HAM/TSP#2 | 4727 | chr10_48265640_- | 8 | 0 | 0 | 0 | 0 |  |
| HAM/TSP#2 | 4728 | chr10_48630505_+ | 1 | 0 | 0 | 0 | 0 |  |
| HAM/TSP#2 | 4729 | chr10_48704061_+ | 1 | 0 | 0 | 0 | 0 |  |
| HAM/TSP#2 | 4730 | chr10_49600214_+ | 0 | 5 | 0 | 0 | 0 |  |
| HAM/TSP#2 | 4731 | chr10_50642468_+ | 1 | 0 | 0 | 0 | 0 |  |
| HAM/TSP#2 | 4732 | chr10_52687826_- | 1 | 0 | 0 | 0 | 0 |  |
| HAM/TSP#2 | 4733 | chr10_52916386_- | 9 | 0 | 0 | 0 | 0 |  |
| HAM/TSP#2 | 4734 | chr10_53133195_+ | 2 | 0 | 0 | 0 | 0 |  |
| HAM/TSP#2 | 4735 | chr10_54026689_+ | 5 | 0 | 0 | 0 | 0 |  |
| HAM/TSP#2 | 4736 | chr10_54612386_- | 1 | 0 | 0 | 0 | 0 |  |
| HAM/TSP#2 | 4737 | chr10_54799618_+ | 1 | 0 | 0 | 0 | 0 |  |
| HAM/TSP#2 | 4738 | chr10_55053400_- | 1 | 0 | 0 | 0 | 0 |  |
| HAM/TSP#2 | 4739 | chr10_55181918_- | 1 | 1 | 0 | 0 | 0 |  |
| HAM/TSP#2 | 4740 | chr10_55356281_+ | 7 | 0 | 0 | 0 | 0 |  |
| HAM/TSP#2 | 4741 | chr10_55635979_+ | 1 | 0 | 0 | 0 | 0 |  |
| HAM/TSP#2 | 4742 | chr10_55742971_- | 1 | 0 | 0 | 0 | 0 |  |
| HAM/TSP#2 | 4743 | chr10_55742972_- | 2 | 0 | 0 | 0 | 0 |  |
| HAM/TSP#2 | 4744 | chr10_57481862_- | 4 | 0 | 0 | 0 | 0 |  |
| HAM/TSP#2 | 4745 | chr10_58858252_- | 4 | 0 | 0 | 0 | 0 |  |
| HAM/TSP#2 | 4746 | chr10_59149897_- | 2 | 0 | 0 | 0 | 0 |  |
| HAM/TSP#2 | 4747 | chr10_59818688_+ | 3 | 0 | 0 | 0 | 0 |  |
| HAM/TSP#2 | 4748 | chr10_61105614_+ | 3 | 0 | 0 | 0 | 0 |  |
| HAM/TSP#2 | 4749 | chr10_6137045_+ | 1 | 0 | 0 | 0 | 0 |  |
| HAM/TSP#2 | 4750 | chr10_62150732_+ | 6 | 0 | 0 | 0 | 0 |  |
| HAM/TSP#2 | 4751 | chr10_63817132_+ | 4 | 0 | 0 | 0 | 0 |  |
| HAM/TSP#2 | 4752 | chr10_64196891_- | 1 | 0 | 0 | 0 | 0 |  |
| HAM/TSP#2 | 4753 | chr10_66117942_- | 1 | 0 | 0 | 0 | 0 |  |
| HAM/TSP#2 | 4754 | chr10_66704514_+ | 1 | 0 | 0 | 0 | 0 |  |
| HAM/TSP#2 | 4755 | chr10_66811159_- | 4 | 0 | 0 | 0 | 0 |  |
| HAM/TSP#2 | 4756 | chr10_66895293_- | 1 | 0 | 0 | 0 | 0 |  |
| HAM/TSP#2 | 4757 | chr10_67290588_+ | 1 | 0 | 0 | 0 | 0 |  |
| HAM/TSP#2 | 4758 | chr10_68257886_- | 1 | 0 | 0 | 0 | 0 |  |
| HAM/TSP#2 | 4759 | chr10_6930125_+ | 2 | 0 | 0 | 0 | 0 |  |
| HAM/TSP#2 | 4760 | chr10_69395379_+ | 2 | 0 | 0 | 0 | 0 |  |
| HAM/TSP#2 | 4761 | chr10_69469388_+ | 3 | 0 | 0 | 0 | 0 |  |
| HAM/TSP#2 | 4762 | chr10_7015185_- | 0 | 13 | 0 | 0 | 0 |  |
| HAM/TSP#2 | 4763 | chr10_70363882_+ | 1 | 0 | 0 | 0 | 0 |  |
| HAM/TSP#2 | 4764 | chr10_70476362_- | 0 | 5 | 0 | 0 | 0 |  |
| HAM/TSP#2 | 4765 | chr10_70584265_- | 2 | 0 | 0 | 0 | 0 |  |
| HAM/TSP#2 | 4766 | chr10_70677991_+ | 1 | 0 | 0 | 0 | 0 |  |
| HAM/TSP#2 | 4767 | chr10_71000013_- | 1 | 0 | 0 | 0 | 0 |  |
| HAM/TSP#2 | 4768 | chr10_71806552_- | 0 | 1 | 0 | 0 | 0 |  |
| HAM/TSP#2 | 4769 | chr10_77261394_- | 3 | 0 | 0 | 0 | 0 |  |
| HAM/TSP#2 | 4770 | chr10_77408495_- | 0 | 2 | 0 | 0 | 0 |  |
| HAM/TSP#2 | 4771 | chr10_77576021_- | 0 | 2 | 0 | 0 | 0 |  |
| HAM/TSP#2 | 4772 | chr10_77857166_+ | 1 | 0 | 0 | 0 | 0 |  |
| HAM/TSP#2 | 4773 | chr10_79215153_+ | 1 | 0 | 0 | 0 | 0 |  |
| HAM/TSP#2 | 4774 | chr10_7945586_- | 0 | 0 | 0 | 0 | 1 |  |
| HAM/TSP#2 | 4775 | chr10_81118548_- | 1 | 0 | 0 | 0 | 0 |  |
| HAM/TSP#2 | 4776 | chr10_82355295_+ | 9 | 0 | 0 | 0 | 0 |  |
| HAM/TSP#2 | 4777 | chr10_8314930_+ | 13 | 0 | 0 | 0 | 0 |  |
| HAM/TSP#2 | 4778 | chr10_83692881_- | 3 | 0 | 0 | 0 | 0 |  |
| HAM/TSP#2 | 4779 | chr10_88098763_+ | 1 | 0 | 0 | 0 | 0 |  |
| HAM/TSP#2 | 4780 | chr10_88735482_+ | 3 | 0 | 0 | 0 | 0 |  |
| HAM/TSP#2 | 4781 | chr10_88965298_- | 4 | 0 | 0 | 0 | 0 |  |
| HAM/TSP#2 | 4782 | chr10_89206022_+ | 1 | 0 | 0 | 0 | 0 |  |
| HAM/TSP#2 | 4783 | chr10_89494034_+ | 1 | 0 | 0 | 0 | 0 |  |
| HAM/TSP#2 | 4784 | chr10_92776861_+ | 1 | 0 | 0 | 0 | 0 |  |
| HAM/TSP#2 | 4785 | chr10_93713168_+ | 2 | 0 | 0 | 0 | 1 |  |
| HAM/TSP#2 | 4786 | chr10_95654777_+ | 1 | 0 | 0 | 0 | 0 |  |
| HAM/TSP#2 | 4787 | chr10_96469482_+ | 1 | 0 | 0 | 0 | 0 |  |
| HAM/TSP#2 | 4788 | chr10_97551506_+ | 1 | 0 | 0 | 0 | 0 |  |
| HAM/TSP#2 | 4789 | chr10_99388882_- | 2 | 0 | 0 | 0 | 0 |  |
| HAM/TSP#2 | 4790 | chr11_101086288_- | 1 | 0 | 0 | 0 | 0 |  |
| HAM/TSP#2 | 4791 | chr11_101947838_+ | 1 | 0 | 0 | 0 | 0 |  |
| HAM/TSP#2 | 4792 | chr11_104322893_+ | 1 | 0 | 0 | 0 | 0 |  |
| HAM/TSP#2 | 4793 | chr11_105058464_- | 2 | 0 | 0 | 0 | 0 |  |
| HAM/TSP#2 | 4794 | chr11_106758849_- | 1 | 0 | 0 | 0 | 0 |  |
| HAM/TSP#2 | 4795 | chr11_107862909_- | 1 | 0 | 0 | 0 | 0 |  |
| HAM/TSP#2 | 4796 | chr11_108466251_+ | 2 | 0 | 0 | 0 | 0 |  |
| HAM/TSP#2 | 4797 | chr11_108578931_- | 1 | 0 | 0 | 0 | 0 |  |
| HAM/TSP#2 | 4798 | chr11_108753065_- | 1 | 0 | 0 | 0 | 0 |  |
| HAM/TSP#2 | 4799 | chr11_11058804_+ | 41 | 2 | 0 | 0 | 0 |  |
| HAM/TSP#2 | 4800 | chr11_110891482_- | 0 | 3 | 0 | 0 | 0 |  |
| HAM/TSP#2 | 4801 | chr11_113159998_+ | 1 | 0 | 0 | 0 | 0 |  |
| HAM/TSP#2 | 4802 | chr11_113873706_+ | 1 | 0 | 0 | 0 | 0 |  |
| HAM/TSP#2 | 4803 | chr11_114186589_+ | 2 | 0 | 0 | 0 | 0 |  |
| HAM/TSP#2 | 4804 | chr11_114360587_- | 2 | 0 | 0 | 0 | 0 |  |
| HAM/TSP#2 | 4805 | chr11_11524032_- | 1 | 0 | 0 | 0 | 0 |  |
| HAM/TSP#2 | 4806 | chr11_115447789_- | 1 | 0 | 0 | 0 | 0 |  |
| HAM/TSP#2 | 4807 | chr11_116031133_+ | 3 | 0 | 0 | 0 | 0 |  |
| HAM/TSP#2 | 4808 | chr11_11617245_+ | 1 | 0 | 0 | 0 | 0 |  |
| HAM/TSP#2 | 4809 | chr11_116727981_+ | 2 | 0 | 0 | 0 | 0 |  |
| HAM/TSP#2 | 4810 | chr11_119297338_- | 0 | 1 | 0 | 0 | 0 |  |
| HAM/TSP#2 | 4811 | chr11_120060102_+ | 3 | 0 | 0 | 0 | 0 |  |
| HAM/TSP#2 | 4812 | chr11_120299510_+ | 0 | 1 | 0 | 0 | 0 |  |
| HAM/TSP#2 | 4813 | chr11_12182817_- | 1 | 0 | 0 | 0 | 0 |  |
| HAM/TSP#2 | 4814 | chr11_12225963_+ | 1 | 0 | 0 | 0 | 0 |  |
| HAM/TSP#2 | 4815 | chr11_122607785_- | 0 | 0 | 0 | 0 | 1 |  |
| HAM/TSP#2 | 4816 | chr11_123381675_+ | 1 | 0 | 0 | 0 | 0 |  |
| HAM/TSP#2 | 4817 | chr11_123617957_- | 1 | 0 | 0 | 0 | 0 |  |
| HAM/TSP#2 | 4818 | chr11_123732057_+ | 1 | 0 | 0 | 0 | 0 |  |
| HAM/TSP#2 | 4819 | chr11_123959999_+ | 1 | 0 | 0 | 0 | 0 |  |
| HAM/TSP#2 | 4820 | chr11_123960002_+ | 1 | 0 | 0 | 0 | 0 |  |
| HAM/TSP#2 | 4821 | chr11_125351552_+ | 1 | 0 | 0 | 0 | 0 |  |
| HAM/TSP#2 | 4822 | chr11_127873856_- | 6 | 0 | 0 | 0 | 0 |  |
| HAM/TSP#2 | 4823 | chr11_130005214_+ | 1 | 0 | 0 | 0 | 0 |  |
| HAM/TSP#2 | 4824 | chr11_130309464_+ | 5 | 0 | 0 | 0 | 1 |  |
| HAM/TSP#2 | 4825 | chr11_130474829_+ | 1 | 0 | 0 | 0 | 0 |  |
| HAM/TSP#2 | 4826 | chr11_131606159_+ | 1 | 0 | 0 | 0 | 0 |  |
| HAM/TSP#2 | 4827 | chr11_132547134_+ | 1 | 0 | 0 | 0 | 0 |  |
| HAM/TSP#2 | 4828 | chr11_133263616_- | 2 | 0 | 0 | 0 | 0 |  |
| HAM/TSP#2 | 4829 | chr11_133399986_- | 1 | 0 | 0 | 0 | 0 |  |
| HAM/TSP#2 | 4830 | chr11_133922693_+ | 1 | 0 | 0 | 0 | 0 |  |
| HAM/TSP#2 | 4831 | chr11_134034517_+ | 1 | 0 | 0 | 0 | 0 |  |
| HAM/TSP#2 | 4832 | chr11_14342531_- | 3 | 0 | 0 | 0 | 0 |  |
| HAM/TSP#2 | 4833 | chr11_15418093_+ | 2 | 0 | 0 | 0 | 0 |  |
| HAM/TSP#2 | 4834 | chr11_16114356_- | 2 | 0 | 0 | 0 | 0 |  |
| HAM/TSP#2 | 4835 | chr11_16567403_+ | 1 | 0 | 0 | 0 | 0 |  |
| HAM/TSP#2 | 4836 | chr11_16607194_- | 16 | 0 | 0 | 0 | 0 |  |
| HAM/TSP#2 | 4837 | chr11_17467452_- | 1 | 0 | 0 | 0 | 0 |  |
| HAM/TSP#2 | 4838 | chr11_17543691_- | 1 | 0 | 0 | 0 | 0 |  |
| HAM/TSP#2 | 4839 | chr11_18116484_- | 2 | 0 | 0 | 0 | 0 |  |
| HAM/TSP#2 | 4840 | chr11_19791046_+ | 1 | 1 | 0 | 0 | 0 |  |
| HAM/TSP#2 | 4841 | chr11_21274812_- | 1 | 0 | 0 | 0 | 0 |  |
| HAM/TSP#2 | 4842 | chr11_21345209_+ | 2 | 0 | 0 | 0 | 0 |  |
| HAM/TSP#2 | 4843 | chr11_21712325_- | 2 | 0 | 0 | 0 | 0 |  |
| HAM/TSP#2 | 4844 | chr11_22688692_+ | 0 | 1 | 0 | 0 | 0 |  |
| HAM/TSP#2 | 4845 | chr11_23702238_+ | 1 | 0 | 0 | 0 | 0 |  |
| HAM/TSP#2 | 4846 | chr11_23709371_- | 0 | 1 | 0 | 0 | 0 |  |
| HAM/TSP#2 | 4847 | chr11_24894022_- | 2 | 0 | 0 | 0 | 0 |  |
| HAM/TSP#2 | 4848 | chr11_2582422_+ | 1 | 0 | 0 | 0 | 0 |  |
| HAM/TSP#2 | 4849 | chr11_27206959_- | 3 | 0 | 0 | 0 | 0 |  |
| HAM/TSP#2 | 4850 | chr11_28029326_- | 1 | 0 | 0 | 0 | 0 |  |
| HAM/TSP#2 | 4851 | chr11_29595796_- | 6 | 0 | 0 | 0 | 0 |  |
| HAM/TSP#2 | 4852 | chr11_30339991_+ | 1 | 0 | 0 | 0 | 0 |  |
| HAM/TSP#2 | 4853 | chr11_3049498_- | 1 | 0 | 0 | 0 | 0 |  |
| HAM/TSP#2 | 4854 | chr11_30595872_- | 2 | 0 | 0 | 0 | 0 |  |
| HAM/TSP#2 | 4855 | chr11_31100557_- | 4 | 0 | 0 | 0 | 0 |  |
| HAM/TSP#2 | 4856 | chr11_31724664_- | 2 | 0 | 0 | 0 | 0 |  |
| HAM/TSP#2 | 4857 | chr11_32315500_+ | 1 | 0 | 0 | 0 | 0 |  |
| HAM/TSP#2 | 4858 | chr11_33628058_- | 1 | 0 | 0 | 0 | 0 |  |
| HAM/TSP#2 | 4859 | chr11_34243941_- | 1 | 0 | 0 | 0 | 0 |  |
| HAM/TSP#2 | 4860 | chr11_35574952_+ | 0 | 1 | 0 | 0 | 0 |  |
| HAM/TSP#2 | 4861 | chr11_36528195_+ | 25 | 1 | 6 | 0 | 0 |  |
| HAM/TSP#2 | 4862 | chr11_38605242_+ | 1 | 0 | 0 | 0 | 0 |  |
| HAM/TSP#2 | 4863 | chr11_38838223_- | 3 | 0 | 0 | 0 | 0 |  |
| HAM/TSP#2 | 4864 | chr11_39110069_- | 2 | 0 | 0 | 0 | 0 |  |
| HAM/TSP#2 | 4865 | chr11_40475618_+ | 1 | 0 | 0 | 0 | 0 |  |
| HAM/TSP#2 | 4866 | chr11_40475619_+ | 2 | 0 | 0 | 0 | 0 |  |
| HAM/TSP#2 | 4867 | chr11_40905336_- | 7 | 0 | 0 | 0 | 0 |  |
| HAM/TSP#2 | 4868 | chr11_41867081_+ | 1 | 0 | 0 | 0 | 0 |  |
| HAM/TSP#2 | 4869 | chr11_42613595_- | 4 | 0 | 0 | 0 | 0 |  |
| HAM/TSP#2 | 4870 | chr11_43189822_- | 2 | 0 | 0 | 0 | 0 |  |
| HAM/TSP#2 | 4871 | chr11_44788580_+ | 1 | 0 | 0 | 0 | 0 |  |
| HAM/TSP#2 | 4872 | chr11_45921279_- | 1 | 0 | 0 | 0 | 0 |  |
| HAM/TSP#2 | 4873 | chr11_45921285_- | 8 | 0 | 0 | 0 | 0 |  |
| HAM/TSP#2 | 4874 | chr11_47217504_+ | 1 | 0 | 0 | 0 | 0 |  |
| HAM/TSP#2 | 4875 | chr11_48612174_+ | 2 | 0 | 0 | 0 | 0 |  |
| HAM/TSP#2 | 4876 | chr11_49716093_- | 0 | 4 | 0 | 0 | 0 |  |
| HAM/TSP#2 | 4877 | chr11_50204695_+ | 1 | 0 | 0 | 0 | 0 |  |
| HAM/TSP#2 | 4878 | chr11_51972044_+ | 0 | 1 | 0 | 0 | 0 |  |
| HAM/TSP#2 | 4879 | chr11_53512705_+ | 2 | 0 | 0 | 0 | 0 |  |
| HAM/TSP#2 | 4880 | chr11_55424548_+ | 6 | 0 | 0 | 0 | 0 |  |
| HAM/TSP#2 | 4881 | chr11_56394407_- | 1 | 0 | 0 | 0 | 0 |  |
| HAM/TSP#2 | 4882 | chr11_57755069_- | 1 | 0 | 0 | 0 | 0 |  |
| HAM/TSP#2 | 4883 | chr11_58077104_+ | 1 | 0 | 0 | 0 | 0 |  |
| HAM/TSP#2 | 4884 | chr11_59148235_- | 1 | 0 | 0 | 0 | 0 |  |
| HAM/TSP#2 | 4885 | chr11_61039329_+ | 11 | 1 | 0 | 0 | 0 |  |
| HAM/TSP#2 | 4886 | chr11_61863706_+ | 0 | 0 | 1 | 0 | 0 |  |
| HAM/TSP#2 | 4887 | chr11_63705098_+ | 1 | 0 | 0 | 0 | 0 |  |
| HAM/TSP#2 | 4888 | chr11_63933030_+ | 0 | 0 | 0 | 1 | 0 |  |
| HAM/TSP#2 | 4889 | chr11_64254847_- | 1 | 0 | 0 | 0 | 0 |  |
| HAM/TSP#2 | 4890 | chr11_64655052_- | 1 | 0 | 0 | 0 | 0 |  |
| HAM/TSP#2 | 4891 | chr11_65052735_+ | 1 | 0 | 0 | 0 | 0 |  |
| HAM/TSP#2 | 4892 | chr11_65283853_- | 0 | 1 | 0 | 0 | 0 |  |
| HAM/TSP#2 | 4893 | chr11_67112616_+ | 1 | 0 | 0 | 0 | 0 |  |
| HAM/TSP#2 | 4894 | chr11_68511234_+ | 1 | 0 | 0 | 0 | 0 |  |
| HAM/TSP#2 | 4895 | chr11_68993659_- | 1 | 0 | 0 | 0 | 0 |  |
| HAM/TSP#2 | 4896 | chr11_69337784_+ | 1 | 0 | 0 | 0 | 0 |  |
| HAM/TSP#2 | 4897 | chr11_7000457_- | 0 | 4 | 0 | 0 | 0 |  |
| HAM/TSP#2 | 4898 | chr11_70278708_+ | 0 | 1 | 0 | 0 | 0 |  |
| HAM/TSP#2 | 4899 | chr11_70773666_+ | 1 | 0 | 0 | 0 | 0 |  |
| HAM/TSP#2 | 4900 | chr11_75009046_+ | 1 | 0 | 0 | 0 | 0 |  |
| HAM/TSP#2 | 4901 | chr11_77192482_- | 0 | 1 | 0 | 0 | 0 |  |
| HAM/TSP#2 | 4902 | chr11_77400688_- | 0 | 2 | 0 | 0 | 0 |  |
| HAM/TSP#2 | 4903 | chr11_79271081_+ | 3 | 0 | 0 | 0 | 0 |  |
| HAM/TSP#2 | 4904 | chr11_80319065_- | 1 | 0 | 0 | 0 | 0 |  |
| HAM/TSP#2 | 4905 | chr11_80345590_- | 1 | 0 | 0 | 0 | 0 |  |
| HAM/TSP#2 | 4906 | chr11_80392762_+ | 2 | 0 | 0 | 0 | 0 |  |
| HAM/TSP#2 | 4907 | chr11_81145271_- | 5 | 0 | 0 | 0 | 0 |  |
| HAM/TSP#2 | 4908 | chr11_81596650_+ | 17 | 0 | 0 | 0 | 0 |  |
| HAM/TSP#2 | 4909 | chr11_82180754_- | 1 | 0 | 0 | 0 | 0 |  |
| HAM/TSP#2 | 4910 | chr11_82979894_+ | 2 | 0 | 0 | 0 | 0 |  |
| HAM/TSP#2 | 4911 | chr11_83020059_- | 1 | 0 | 0 | 0 | 0 |  |
| HAM/TSP#2 | 4912 | chr11_84390797_+ | 0 | 1 | 0 | 0 | 0 |  |
| HAM/TSP#2 | 4913 | chr11_84640553_- | 2 | 0 | 0 | 0 | 0 |  |
| HAM/TSP#2 | 4914 | chr11_84897324_- | 1 | 0 | 0 | 0 | 0 |  |
| HAM/TSP#2 | 4915 | chr11_85682099_+ | 2 | 0 | 0 | 0 | 0 |  |
| HAM/TSP#2 | 4916 | chr11_85710749_+ | 1 | 0 | 0 | 0 | 0 |  |
| HAM/TSP#2 | 4917 | chr11_86183140_- | 4 | 0 | 0 | 0 | 0 |  |
| HAM/TSP#2 | 4918 | chr11_8814916_+ | 3 | 0 | 0 | 0 | 0 |  |
| HAM/TSP#2 | 4919 | chr11_8822692_- | 1 | 0 | 0 | 0 | 0 |  |
| HAM/TSP#2 | 4920 | chr11_8862474_- | 1 | 0 | 0 | 0 | 0 |  |
| HAM/TSP#2 | 4921 | chr11_90148687_+ | 1 | 0 | 0 | 0 | 0 |  |
| HAM/TSP#2 | 4922 | chr11_90752879_- | 1 | 0 | 0 | 0 | 0 |  |
| HAM/TSP#2 | 4923 | chr11_91176930_+ | 0 | 1 | 0 | 0 | 0 |  |
| HAM/TSP#2 | 4924 | chr11_9156042_+ | 0 | 0 | 1 | 0 | 0 |  |
| HAM/TSP#2 | 4925 | chr11_94423745_+ | 1 | 0 | 0 | 0 | 0 |  |
| HAM/TSP#2 | 4926 | chr11_96529177_- | 2 | 0 | 0 | 0 | 0 |  |
| HAM/TSP#2 | 4927 | chr11_9949210_- | 1 | 0 | 0 | 0 | 0 |  |
| HAM/TSP#2 | 4928 | chr11_99525000_+ | 1 | 0 | 0 | 0 | 0 |  |
| HAM/TSP#2 | 4929 | chr12_100984003_+ | 2 | 0 | 0 | 0 | 0 |  |
| HAM/TSP#2 | 4930 | chr12_101116527_- | 1 | 0 | 0 | 0 | 0 |  |
| HAM/TSP#2 | 4931 | chr12_101744172_- | 2 | 0 | 0 | 0 | 0 |  |
| HAM/TSP#2 | 4932 | chr12_101761419_- | 1 | 0 | 0 | 0 | 0 |  |
| HAM/TSP#2 | 4933 | chr12_103719205_- | 1 | 0 | 0 | 0 | 0 |  |
| HAM/TSP#2 | 4934 | chr12_10652146_- | 1 | 0 | 0 | 0 | 0 |  |
| HAM/TSP#2 | 4935 | chr12_107374937_+ | 1 | 0 | 0 | 0 | 0 |  |
| HAM/TSP#2 | 4936 | chr12_107755948_+ | 1 | 0 | 0 | 0 | 0 |  |
| HAM/TSP#2 | 4937 | chr12_107844204_- | 1 | 0 | 0 | 0 | 0 |  |
| HAM/TSP#2 | 4938 | chr12_108690102_- | 6 | 0 | 0 | 0 | 0 |  |
| HAM/TSP#2 | 4939 | chr12_110845006_- | 1 | 0 | 0 | 0 | 0 |  |
| HAM/TSP#2 | 4940 | chr12_110849499_- | 1 | 0 | 0 | 0 | 0 |  |
| HAM/TSP#2 | 4941 | chr12_110855390_- | 0 | 0 | 0 | 0 | 1 |  |
| HAM/TSP#2 | 4942 | chr12_111615324_+ | 1 | 0 | 0 | 0 | 0 |  |
| HAM/TSP#2 | 4943 | chr12_112939447_- | 3 | 0 | 0 | 0 | 0 |  |
| HAM/TSP#2 | 4944 | chr12_113347019_- | 4 | 0 | 0 | 0 | 0 |  |
| HAM/TSP#2 | 4945 | chr12_114984401_- | 1 | 0 | 0 | 0 | 0 |  |
| HAM/TSP#2 | 4946 | chr12_115882136_- | 1 | 0 | 0 | 0 | 0 |  |
| HAM/TSP#2 | 4947 | chr12_117148184_+ | 1 | 0 | 0 | 0 | 0 |  |
| HAM/TSP#2 | 4948 | chr12_119509598_+ | 1 | 0 | 0 | 0 | 0 |  |
| HAM/TSP#2 | 4949 | chr12_119701939_+ | 5 | 0 | 0 | 0 | 0 |  |
| HAM/TSP#2 | 4950 | chr12_120139653_+ | 6 | 0 | 0 | 0 | 0 |  |
| HAM/TSP#2 | 4951 | chr12_123059035_- | 1 | 0 | 0 | 0 | 0 |  |
| HAM/TSP#2 | 4952 | chr12_123109365_+ | 1 | 0 | 0 | 0 | 0 |  |
| HAM/TSP#2 | 4953 | chr12_123675020_+ | 3 | 0 | 0 | 0 | 0 |  |
| HAM/TSP#2 | 4954 | chr12_123713225_+ | 1 | 0 | 0 | 0 | 0 |  |
| HAM/TSP#2 | 4955 | chr12_124751269_- | 2 | 0 | 0 | 0 | 0 |  |
| HAM/TSP#2 | 4956 | chr12_124774026_+ | 1 | 0 | 0 | 0 | 0 |  |
| HAM/TSP#2 | 4957 | chr12_124779401_+ | 1 | 0 | 0 | 0 | 0 |  |
| HAM/TSP#2 | 4958 | chr12_12486273_+ | 1 | 0 | 0 | 0 | 0 |  |
| HAM/TSP#2 | 4959 | chr12_125216775_- | 1 | 0 | 0 | 0 | 0 |  |
| HAM/TSP#2 | 4960 | chr12_12645206_- | 2 | 0 | 0 | 0 | 0 |  |
| HAM/TSP#2 | 4961 | chr12_12803781_- | 1 | 0 | 0 | 0 | 0 |  |
| HAM/TSP#2 | 4962 | chr12_130749219_- | 1 | 0 | 0 | 0 | 0 |  |
| HAM/TSP#2 | 4963 | chr12_131037203_- | 1 | 0 | 0 | 0 | 0 |  |
| HAM/TSP#2 | 4964 | chr12_131124204_+ | 1 | 0 | 0 | 0 | 0 |  |
| HAM/TSP#2 | 4965 | chr12_132739294_- | 0 | 9 | 0 | 0 | 0 |  |
| HAM/TSP#2 | 4966 | chr12_132955077_- | 1 | 0 | 0 | 0 | 0 |  |
| HAM/TSP#2 | 4967 | chr12_13920408_+ | 14 | 0 | 0 | 0 | 0 |  |
| HAM/TSP#2 | 4968 | chr12_14336754_+ | 0 | 1 | 0 | 0 | 0 |  |
| HAM/TSP#2 | 4969 | chr12_1526896_+ | 1 | 0 | 0 | 0 | 0 |  |
| HAM/TSP#2 | 4970 | chr12_15789322_+ | 1 | 0 | 0 | 0 | 0 |  |
| HAM/TSP#2 | 4971 | chr12_1687934_- | 2 | 0 | 0 | 0 | 0 |  |
| HAM/TSP#2 | 4972 | chr12_17094337_+ | 1 | 0 | 0 | 0 | 0 |  |
| HAM/TSP#2 | 4973 | chr12_17646972_+ | 0 | 1 | 0 | 0 | 0 |  |
| HAM/TSP#2 | 4974 | chr12_1803680_+ | 2 | 0 | 0 | 0 | 0 |  |
| HAM/TSP#2 | 4975 | chr12_18066585_- | 1 | 0 | 0 | 0 | 0 |  |
| HAM/TSP#2 | 4976 | chr12_18419962_- | 1 | 0 | 0 | 0 | 0 |  |
| HAM/TSP#2 | 4977 | chr12_18620201_+ | 1 | 0 | 0 | 0 | 0 |  |
| HAM/TSP#2 | 4978 | chr12_18699142_- | 3 | 0 | 0 | 0 | 0 |  |
| HAM/TSP#2 | 4979 | chr12_18957638_+ | 1 | 0 | 0 | 0 | 0 |  |
| HAM/TSP#2 | 4980 | chr12_20103432_- | 1 | 0 | 0 | 0 | 0 |  |
| HAM/TSP#2 | 4981 | chr12_21090456_+ | 0 | 3 | 0 | 0 | 0 |  |
| HAM/TSP#2 | 4982 | chr12_2115469_- | 1 | 0 | 0 | 0 | 0 |  |
| HAM/TSP#2 | 4983 | chr12_24043586_+ | 1 | 0 | 0 | 0 | 0 |  |
| HAM/TSP#2 | 4984 | chr12_26319738_- | 1 | 0 | 0 | 0 | 0 |  |
| HAM/TSP#2 | 4985 | chr12_26555877_+ | 6 | 0 | 0 | 0 | 0 |  |
| HAM/TSP#2 | 4986 | chr12_26726640_- | 5 | 0 | 0 | 0 | 0 |  |
| HAM/TSP#2 | 4987 | chr12_29456198_+ | 2 | 0 | 0 | 0 | 0 |  |
| HAM/TSP#2 | 4988 | chr12_3012504_+ | 1 | 0 | 0 | 0 | 0 |  |
| HAM/TSP#2 | 4989 | chr12_31344623_- | 2 | 0 | 0 | 0 | 0 |  |
| HAM/TSP#2 | 4990 | chr12_31441320_- | 1 | 0 | 0 | 0 | 0 |  |
| HAM/TSP#2 | 4991 | chr12_33037457_- | 1 | 0 | 0 | 0 | 1 |  |
| HAM/TSP#2 | 4992 | chr12_34301092_+ | 1 | 0 | 0 | 0 | 0 |  |
| HAM/TSP#2 | 4993 | chr12_34690984_+ | 1 | 0 | 0 | 0 | 0 |  |
| HAM/TSP#2 | 4994 | chr12_35476498_+ | 0 | 1 | 0 | 0 | 0 |  |
| HAM/TSP#2 | 4995 | chr12_3714212_+ | 8 | 0 | 0 | 0 | 0 |  |
| HAM/TSP#2 | 4996 | chr12_3753763_- | 3 | 0 | 0 | 0 | 0 |  |
| HAM/TSP#2 | 4997 | chr12_38943626_- | 1 | 0 | 0 | 0 | 0 |  |
| HAM/TSP#2 | 4998 | chr12_39116091_- | 0 | 0 | 0 | 0 | 2 |  |
| HAM/TSP#2 | 4999 | chr12_39448188_- | 2 | 0 | 0 | 0 | 0 |  |
| HAM/TSP#2 | 5000 | chr12_39943393_- | 1 | 0 | 0 | 0 | 0 |  |
| HAM/TSP#2 | 5001 | chr12_40638951_- | 3 | 0 | 0 | 0 | 0 |  |
| HAM/TSP#2 | 5002 | chr12_40767844_+ | 1 | 0 | 0 | 0 | 0 |  |
| HAM/TSP#2 | 5003 | chr12_40780083_- | 4 | 0 | 0 | 0 | 0 |  |
| HAM/TSP#2 | 5004 | chr12_41155241_+ | 1 | 0 | 0 | 0 | 0 |  |
| HAM/TSP#2 | 5005 | chr12_42327588_- | 13 | 0 | 0 | 0 | 0 |  |
| HAM/TSP#2 | 5006 | chr12_43590557_+ | 0 | 0 | 0 | 0 | 1 |  |
| HAM/TSP#2 | 5007 | chr12_44147577_- | 1 | 0 | 0 | 0 | 0 |  |
| HAM/TSP#2 | 5008 | chr12_45320775_- | 1 | 0 | 0 | 0 | 0 |  |
| HAM/TSP#2 | 5009 | chr12_4708662_+ | 1 | 0 | 0 | 0 | 0 |  |
| HAM/TSP#2 | 5010 | chr12_4734898_+ | 1 | 0 | 0 | 0 | 0 |  |
| HAM/TSP#2 | 5011 | chr12_47486496_+ | 1 | 0 | 0 | 0 | 0 |  |
| HAM/TSP#2 | 5012 | chr12_49086589_+ | 1 | 0 | 0 | 0 | 0 |  |
| HAM/TSP#2 | 5013 | chr12_49285064_+ | 0 | 1 | 0 | 0 | 0 |  |
| HAM/TSP#2 | 5014 | chr12_51938942_- | 0 | 1 | 0 | 0 | 0 |  |
| HAM/TSP#2 | 5015 | chr12_52077053_+ | 0 | 7 | 0 | 0 | 0 |  |
| HAM/TSP#2 | 5016 | chr12_52496771_+ | 1 | 0 | 0 | 0 | 0 |  |
| HAM/TSP#2 | 5017 | chr12_52573645_+ | 1 | 0 | 0 | 0 | 0 |  |
| HAM/TSP#2 | 5018 | chr12_54571938_+ | 2 | 0 | 0 | 0 | 0 |  |
| HAM/TSP#2 | 5019 | chr12_54823285_- | 0 | 1 | 0 | 0 | 0 |  |
| HAM/TSP#2 | 5020 | chr12_55012024_+ | 2 | 0 | 0 | 0 | 0 |  |
| HAM/TSP#2 | 5021 | chr12_55540444_- | 1 | 0 | 0 | 0 | 0 |  |
| HAM/TSP#2 | 5022 | chr12_55827995_+ | 18 | 1 | 0 | 0 | 0 |  |
| HAM/TSP#2 | 5023 | chr12_55827996_+ | 1 | 0 | 0 | 0 | 0 |  |
| HAM/TSP#2 | 5024 | chr12_5633280_- | 0 | 2 | 0 | 0 | 0 |  |
| HAM/TSP#2 | 5025 | chr12_57245986_+ | 0 | 1 | 0 | 0 | 0 |  |
| HAM/TSP#2 | 5026 | chr12_57487191_+ | 0 | 0 | 0 | 0 | 2 |  |
| HAM/TSP#2 | 5027 | chr12_58943282_+ | 1 | 0 | 0 | 0 | 0 |  |
| HAM/TSP#2 | 5028 | chr12_61461498_- | 3 | 0 | 15 | 0 | 0 |  |
| HAM/TSP#2 | 5029 | chr12_6212465_- | 1 | 0 | 0 | 0 | 0 |  |
| HAM/TSP#2 | 5030 | chr12_63994749_- | 1 | 0 | 0 | 0 | 0 |  |
| HAM/TSP#2 | 5031 | chr12_64378902_- | 5 | 0 | 0 | 0 | 0 |  |
| HAM/TSP#2 | 5032 | chr12_64868912_+ | 3 | 0 | 0 | 0 | 0 |  |
| HAM/TSP#2 | 5033 | chr12_65009133_+ | 3 | 0 | 0 | 0 | 7 |  |
| HAM/TSP#2 | 5034 | chr12_6729353_- | 1 | 0 | 0 | 0 | 0 |  |
| HAM/TSP#2 | 5035 | chr12_6923366_+ | 9 | 0 | 0 | 0 | 0 |  |
| HAM/TSP#2 | 5036 | chr12_70186618_+ | 2 | 0 | 0 | 0 | 0 |  |
| HAM/TSP#2 | 5037 | chr12_70305150_+ | 1 | 0 | 0 | 0 | 0 |  |
| HAM/TSP#2 | 5038 | chr12_73936181_+ | 2 | 0 | 0 | 0 | 0 |  |
| HAM/TSP#2 | 5039 | chr12_74138225_+ | 1 | 0 | 0 | 0 | 0 |  |
| HAM/TSP#2 | 5040 | chr12_74236529_+ | 4 | 0 | 0 | 0 | 0 |  |
| HAM/TSP#2 | 5041 | chr12_74674108_+ | 14 | 0 | 0 | 0 | 0 |  |
| HAM/TSP#2 | 5042 | chr12_75588379_- | 1 | 0 | 0 | 0 | 0 |  |
| HAM/TSP#2 | 5043 | chr12_7684217_- | 1 | 0 | 0 | 0 | 0 |  |
| HAM/TSP#2 | 5044 | chr12_77370721_+ | 0 | 3 | 0 | 0 | 0 |  |
| HAM/TSP#2 | 5045 | chr12_78061002_- | 0 | 11 | 0 | 0 | 0 |  |
| HAM/TSP#2 | 5046 | chr12_78215141_+ | 1 | 0 | 0 | 0 | 0 |  |
| HAM/TSP#2 | 5047 | chr12_78726403_- | 1 | 0 | 0 | 0 | 0 |  |
| HAM/TSP#2 | 5048 | chr12_78837471_- | 3 | 0 | 0 | 0 | 0 |  |
| HAM/TSP#2 | 5049 | chr12_79402018_- | 8 | 0 | 0 | 0 | 0 |  |
| HAM/TSP#2 | 5050 | chr12_80819689_- | 0 | 2 | 0 | 0 | 0 |  |
| HAM/TSP#2 | 5051 | chr12_82278522_- | 2 | 0 | 0 | 0 | 0 |  |
| HAM/TSP#2 | 5052 | chr12_8259153_- | 1 | 0 | 0 | 0 | 0 |  |
| HAM/TSP#2 | 5053 | chr12_83657839_- | 4 | 0 | 0 | 0 | 0 |  |
| HAM/TSP#2 | 5054 | chr12_84336098_- | 2 | 0 | 0 | 0 | 0 |  |
| HAM/TSP#2 | 5055 | chr12_84357994_- | 0 | 2 | 0 | 0 | 0 |  |
| HAM/TSP#2 | 5056 | chr12_84727822_+ | 0 | 2 | 0 | 0 | 0 |  |
| HAM/TSP#2 | 5057 | chr12_84848746_- | 1 | 0 | 0 | 0 | 0 |  |
| HAM/TSP#2 | 5058 | chr12_85952623_+ | 2 | 0 | 0 | 0 | 0 |  |
| HAM/TSP#2 | 5059 | chr12_87469234_- | 1 | 0 | 0 | 0 | 0 |  |
| HAM/TSP#2 | 5060 | chr12_88076810_+ | 3 | 1 | 0 | 0 | 0 |  |
| HAM/TSP#2 | 5061 | chr12_89245027_- | 1 | 0 | 0 | 0 | 0 |  |
| HAM/TSP#2 | 5062 | chr12_89353014_- | 1 | 0 | 0 | 0 | 0 |  |
| HAM/TSP#2 | 5063 | chr12_91013504_+ | 1 | 0 | 0 | 0 | 0 |  |
| HAM/TSP#2 | 5064 | chr12_92504643_- | 4 | 0 | 0 | 0 | 0 |  |
| HAM/TSP#2 | 5065 | chr12_93259868_+ | 3 | 0 | 0 | 0 | 0 |  |
| HAM/TSP#2 | 5066 | chr12_93769355_- | 1 | 0 | 0 | 0 | 0 |  |
| HAM/TSP#2 | 5067 | chr12_94734320_- | 0 | 8 | 0 | 0 | 0 |  |
| HAM/TSP#2 | 5068 | chr12_94760591_- | 1 | 0 | 0 | 0 | 0 |  |
| HAM/TSP#2 | 5069 | chr12_94963697_- | 1 | 0 | 0 | 0 | 0 |  |
| HAM/TSP#2 | 5070 | chr12_95594863_- | 0 | 1 | 0 | 0 | 0 |  |
| HAM/TSP#2 | 5071 | chr12_96770811_- | 0 | 0 | 0 | 2 | 0 |  |
| HAM/TSP#2 | 5072 | chr12_97928199_- | 2 | 0 | 0 | 0 | 0 |  |
| HAM/TSP#2 | 5073 | chr13_101473323_- | 1 | 0 | 0 | 0 | 0 |  |
| HAM/TSP#2 | 5074 | chr13_101480480_- | 4 | 0 | 0 | 0 | 0 |  |
| HAM/TSP#2 | 5075 | chr13_102164684_- | 2 | 0 | 0 | 0 | 0 |  |
| HAM/TSP#2 | 5076 | chr13_102629725_+ | 1 | 0 | 0 | 0 | 0 |  |
| HAM/TSP#2 | 5077 | chr13_103913430_+ | 2 | 0 | 0 | 0 | 0 |  |
| HAM/TSP#2 | 5078 | chr13_105818035_+ | 1 | 0 | 0 | 0 | 0 |  |
| HAM/TSP#2 | 5079 | chr13_106224235_- | 1 | 0 | 0 | 0 | 0 |  |
| HAM/TSP#2 | 5080 | chr13_106981894_- | 2 | 0 | 0 | 0 | 0 |  |
| HAM/TSP#2 | 5081 | chr13_107245524_+ | 2 | 0 | 0 | 0 | 0 |  |
| HAM/TSP#2 | 5082 | chr13_107705987_- | 1 | 0 | 0 | 0 | 0 |  |
| HAM/TSP#2 | 5083 | chr13_108074833_+ | 1 | 0 | 0 | 0 | 0 |  |
| HAM/TSP#2 | 5084 | chr13_109692030_- | 6 | 0 | 0 | 0 | 0 |  |
| HAM/TSP#2 | 5085 | chr13_110289122_+ | 1 | 0 | 0 | 0 | 0 |  |
| HAM/TSP#2 | 5086 | chr13_110812968_+ | 1 | 0 | 0 | 0 | 0 |  |
| HAM/TSP#2 | 5087 | chr13_112368729_+ | 8 | 0 | 0 | 0 | 0 |  |
| HAM/TSP#2 | 5088 | chr13_18878252_- | 1 | 0 | 0 | 0 | 0 |  |
| HAM/TSP#2 | 5089 | chr13_19017007_- | 17 | 0 | 0 | 0 | 0 |  |
| HAM/TSP#2 | 5090 | chr13_19194345_+ | 1 | 0 | 0 | 0 | 0 |  |
| HAM/TSP#2 | 5091 | chr13_20121733_+ | 0 | 1 | 0 | 0 | 0 |  |
| HAM/TSP#2 | 5092 | chr13_21210281_- | 1 | 0 | 0 | 0 | 0 |  |
| HAM/TSP#2 | 5093 | chr13_21421916_+ | 1 | 1 | 0 | 0 | 0 |  |
| HAM/TSP#2 | 5094 | chr13_23742606_- | 1 | 0 | 0 | 0 | 0 |  |
| HAM/TSP#2 | 5095 | chr13_23794277_- | 1 | 0 | 0 | 0 | 0 |  |
| HAM/TSP#2 | 5096 | chr13_25237497_- | 0 | 1 | 0 | 0 | 0 |  |
| HAM/TSP#2 | 5097 | chr13_25298801_- | 2 | 0 | 0 | 0 | 0 |  |
| HAM/TSP#2 | 5098 | chr13_26140742_- | 1 | 0 | 0 | 0 | 0 |  |
| HAM/TSP#2 | 5099 | chr13_26419906_+ | 1 | 0 | 0 | 0 | 0 |  |
| HAM/TSP#2 | 5100 | chr13_26771775_- | 0 | 1 | 0 | 0 | 0 |  |
| HAM/TSP#2 | 5101 | chr13_27741471_- | 1 | 0 | 0 | 0 | 0 |  |
| HAM/TSP#2 | 5102 | chr13_28080711_- | 1 | 0 | 0 | 0 | 0 |  |
| HAM/TSP#2 | 5103 | chr13_28378477_+ | 2 | 0 | 0 | 0 | 0 |  |
| HAM/TSP#2 | 5104 | chr13_28822950_- | 2 | 0 | 0 | 0 | 0 |  |
| HAM/TSP#2 | 5105 | chr13_28866971_- | 1 | 0 | 0 | 0 | 0 |  |
| HAM/TSP#2 | 5106 | chr13_28890378_+ | 1 | 0 | 0 | 0 | 0 |  |
| HAM/TSP#2 | 5107 | chr13_29278473_+ | 0 | 5 | 0 | 0 | 0 |  |
| HAM/TSP#2 | 5108 | chr13_29546368_+ | 2 | 0 | 0 | 0 | 0 |  |
| HAM/TSP#2 | 5109 | chr13_30201345_- | 3 | 0 | 0 | 0 | 0 |  |
| HAM/TSP#2 | 5110 | chr13_31244783_+ | 4 | 0 | 0 | 0 | 0 |  |
| HAM/TSP#2 | 5111 | chr13_32213313_+ | 0 | 1 | 0 | 0 | 0 |  |
| HAM/TSP#2 | 5112 | chr13_32335228_- | 1 | 0 | 0 | 0 | 0 |  |
| HAM/TSP#2 | 5113 | chr13_32426377_- | 0 | 12 | 0 | 0 | 0 |  |
| HAM/TSP#2 | 5114 | chr13_33612278_+ | 3 | 0 | 0 | 0 | 0 |  |
| HAM/TSP#2 | 5115 | chr13_33655785_- | 1 | 0 | 0 | 0 | 0 |  |
| HAM/TSP#2 | 5116 | chr13_35311849_+ | 5 | 0 | 0 | 0 | 0 |  |
| HAM/TSP#2 | 5117 | chr13_36293543_+ | 5 | 1 | 0 | 0 | 0 |  |
| HAM/TSP#2 | 5118 | chr13_36826050_+ | 1 | 0 | 0 | 0 | 0 |  |
| HAM/TSP#2 | 5119 | chr13_36826051_+ | 4 | 0 | 0 | 0 | 0 |  |
| HAM/TSP#2 | 5120 | chr13_36917380_- | 2 | 0 | 0 | 0 | 0 |  |
| HAM/TSP#2 | 5121 | chr13_37566447_+ | 0 | 2 | 0 | 0 | 0 |  |
| HAM/TSP#2 | 5122 | chr13_37776960_+ | 6 | 0 | 0 | 0 | 0 |  |
| HAM/TSP#2 | 5123 | chr13_38100468_+ | 1 | 0 | 0 | 0 | 0 |  |
| HAM/TSP#2 | 5124 | chr13_39951960_- | 1 | 0 | 0 | 0 | 0 |  |
| HAM/TSP#2 | 5125 | chr13_40032360_+ | 2 | 0 | 0 | 0 | 0 |  |
| HAM/TSP#2 | 5126 | chr13_41062448_+ | 0 | 1 | 0 | 0 | 0 |  |
| HAM/TSP#2 | 5127 | chr13_41463058_- | 1 | 0 | 0 | 0 | 0 |  |
| HAM/TSP#2 | 5128 | chr13_42338522_- | 0 | 1 | 0 | 0 | 0 |  |
| HAM/TSP#2 | 5129 | chr13_42626363_- | 1 | 0 | 0 | 0 | 0 |  |
| HAM/TSP#2 | 5130 | chr13_42699193_+ | 7 | 0 | 0 | 0 | 0 |  |
| HAM/TSP#2 | 5131 | chr13_42808852_- | 8 | 0 | 0 | 0 | 0 |  |
| HAM/TSP#2 | 5132 | chr13_43699139_+ | 1 | 0 | 0 | 0 | 0 |  |
| HAM/TSP#2 | 5133 | chr13_44620925_- | 40 | 1 | 0 | 0 | 0 |  |
| HAM/TSP#2 | 5134 | chr13_45587238_+ | 6 | 0 | 0 | 0 | 0 |  |
| HAM/TSP#2 | 5135 | chr13_46543214_- | 2 | 0 | 0 | 0 | 0 |  |
| HAM/TSP#2 | 5136 | chr13_48225186_- | 1 | 0 | 0 | 0 | 0 |  |
| HAM/TSP#2 | 5137 | chr13_48895872_+ | 1 | 0 | 0 | 0 | 0 |  |
| HAM/TSP#2 | 5138 | chr13_49505473_- | 1 | 0 | 0 | 0 | 0 |  |
| HAM/TSP#2 | 5139 | chr13_50592974_- | 1 | 0 | 0 | 0 | 0 |  |
| HAM/TSP#2 | 5140 | chr13_50981382_- | 1 | 0 | 0 | 0 | 0 |  |
| HAM/TSP#2 | 5141 | chr13_51999185_+ | 0 | 1 | 0 | 0 | 0 |  |
| HAM/TSP#2 | 5142 | chr13_53631948_- | 2 | 0 | 0 | 0 | 0 |  |
| HAM/TSP#2 | 5143 | chr13_54417902_- | 4 | 0 | 8 | 1 | 0 |  |
| HAM/TSP#2 | 5144 | chr13_55926797_- | 0 | 12 | 0 | 0 | 0 |  |
| HAM/TSP#2 | 5145 | chr13_56224502_+ | 3 | 0 | 0 | 0 | 0 |  |
| HAM/TSP#2 | 5146 | chr13_56474234_+ | 2 | 0 | 0 | 0 | 0 |  |
| HAM/TSP#2 | 5147 | chr13_57488116_- | 2 | 0 | 0 | 0 | 0 |  |
| HAM/TSP#2 | 5148 | chr13_58280491_- | 2 | 0 | 0 | 0 | 0 |  |
| HAM/TSP#2 | 5149 | chr13_58572563_+ | 2 | 0 | 0 | 0 | 0 |  |
| HAM/TSP#2 | 5150 | chr13_58638103_+ | 0 | 2 | 0 | 0 | 0 |  |
| HAM/TSP#2 | 5151 | chr13_58973552_+ | 2 | 0 | 0 | 0 | 0 |  |
| HAM/TSP#2 | 5152 | chr13_59950582_+ | 1 | 0 | 0 | 0 | 0 |  |
| HAM/TSP#2 | 5153 | chr13_60520684_- | 0 | 2 | 0 | 0 | 0 |  |
| HAM/TSP#2 | 5154 | chr13_61195789_- | 1 | 0 | 0 | 0 | 0 |  |
| HAM/TSP#2 | 5155 | chr13_61766621_+ | 0 | 0 | 0 | 1 | 0 |  |
| HAM/TSP#2 | 5156 | chr13_62651098_+ | 2 | 0 | 0 | 0 | 0 |  |
| HAM/TSP#2 | 5157 | chr13_62897372_- | 1 | 0 | 0 | 0 | 0 |  |
| HAM/TSP#2 | 5158 | chr13_63579477_- | 1 | 0 | 0 | 0 | 0 |  |
| HAM/TSP#2 | 5159 | chr13_64789517_- | 0 | 2 | 0 | 0 | 0 |  |
| HAM/TSP#2 | 5160 | chr13_65332429_+ | 0 | 28 | 6 | 0 | 0 |  |
| HAM/TSP#2 | 5161 | chr13_65758952_- | 1 | 0 | 0 | 0 | 0 |  |
| HAM/TSP#2 | 5162 | chr13_66495547_+ | 1 | 0 | 0 | 0 | 0 |  |
| HAM/TSP#2 | 5163 | chr13_66652735_+ | 3 | 0 | 0 | 0 | 0 |  |
| HAM/TSP#2 | 5164 | chr13_66704912_+ | 1 | 0 | 0 | 0 | 0 |  |
| HAM/TSP#2 | 5165 | chr13_67700273_- | 39 | 1 | 0 | 0 | 0 |  |
| HAM/TSP#2 | 5166 | chr13_67803744_- | 9 | 2 | 0 | 0 | 0 |  |
| HAM/TSP#2 | 5167 | chr13_68243931_- | 0 | 1 | 0 | 0 | 0 |  |
| HAM/TSP#2 | 5168 | chr13_68680260_+ | 2 | 0 | 0 | 0 | 0 |  |
| HAM/TSP#2 | 5169 | chr13_68928993_+ | 1 | 0 | 0 | 0 | 0 |  |
| HAM/TSP#2 | 5170 | chr13_69045016_- | 1 | 0 | 0 | 0 | 0 |  |
| HAM/TSP#2 | 5171 | chr13_71321087_- | 0 | 0 | 0 | 1 | 0 |  |
| HAM/TSP#2 | 5172 | chr13_74988472_+ | 1 | 0 | 0 | 0 | 0 |  |
| HAM/TSP#2 | 5173 | chr13_75281651_+ | 2 | 0 | 0 | 0 | 0 |  |
| HAM/TSP#2 | 5174 | chr13_76569632_- | 1 | 0 | 0 | 0 | 0 |  |
| HAM/TSP#2 | 5175 | chr13_77147785_+ | 2 | 0 | 0 | 0 | 0 |  |
| HAM/TSP#2 | 5176 | chr13_77364196_- | 2 | 0 | 0 | 0 | 0 |  |
| HAM/TSP#2 | 5177 | chr13_77377365_- | 1 | 0 | 0 | 0 | 0 |  |
| HAM/TSP#2 | 5178 | chr13_78103873_+ | 0 | 2 | 0 | 0 | 0 |  |
| HAM/TSP#2 | 5179 | chr13_78182541_+ | 1 | 0 | 0 | 0 | 0 |  |
| HAM/TSP#2 | 5180 | chr13_79601491_+ | 5 | 0 | 0 | 0 | 0 |  |
| HAM/TSP#2 | 5181 | chr13_79604329_- | 3 | 0 | 0 | 0 | 0 |  |
| HAM/TSP#2 | 5182 | chr13_80487499_+ | 0 | 10 | 0 | 0 | 0 |  |
| HAM/TSP#2 | 5183 | chr13_81453832_- | 3 | 0 | 0 | 0 | 0 |  |
| HAM/TSP#2 | 5184 | chr13_81781379_+ | 0 | 1 | 0 | 0 | 0 |  |
| HAM/TSP#2 | 5185 | chr13_82255933_+ | 2 | 0 | 0 | 0 | 0 |  |
| HAM/TSP#2 | 5186 | chr13_83235609_- | 6 | 0 | 0 | 0 | 0 |  |
| HAM/TSP#2 | 5187 | chr13_84986426_+ | 0 | 10 | 0 | 0 | 0 |  |
| HAM/TSP#2 | 5188 | chr13_85142038_+ | 5 | 0 | 0 | 0 | 0 |  |
| HAM/TSP#2 | 5189 | chr13_85223263_- | 1 | 0 | 0 | 0 | 0 |  |
| HAM/TSP#2 | 5190 | chr13_86062216_+ | 0 | 6 | 0 | 0 | 0 |  |
| HAM/TSP#2 | 5191 | chr13_86764158_+ | 5 | 0 | 0 | 0 | 0 |  |
| HAM/TSP#2 | 5192 | chr13_86916487_+ | 2 | 0 | 0 | 0 | 0 |  |
| HAM/TSP#2 | 5193 | chr13_88905537_+ | 1 | 0 | 0 | 0 | 0 |  |
| HAM/TSP#2 | 5194 | chr13_89075626_+ | 3 | 0 | 0 | 0 | 0 |  |
| HAM/TSP#2 | 5195 | chr13_90125782_- | 1 | 0 | 0 | 0 | 0 |  |
| HAM/TSP#2 | 5196 | chr13_90297388_+ | 1 | 0 | 0 | 0 | 0 |  |
| HAM/TSP#2 | 5197 | chr13_90549305_+ | 2 | 0 | 0 | 0 | 0 |  |
| HAM/TSP#2 | 5198 | chr13_90832369_+ | 1 | 0 | 0 | 0 | 0 |  |
| HAM/TSP#2 | 5199 | chr13_91246933_- | 5 | 0 | 0 | 0 | 0 |  |
| HAM/TSP#2 | 5200 | chr13_92291928_+ | 1 | 0 | 0 | 0 | 0 |  |
| HAM/TSP#2 | 5201 | chr13_93219334_+ | 1 | 0 | 0 | 0 | 0 |  |
| HAM/TSP#2 | 5202 | chr13_93256584_+ | 1 | 14 | 11 | 30 | 12 |  |
| HAM/TSP#2 | 5203 | chr13_94548579_- | 3 | 0 | 0 | 0 | 0 |  |
| HAM/TSP#2 | 5204 | chr13_95158018_- | 1 | 0 | 0 | 0 | 0 |  |
| HAM/TSP#2 | 5205 | chr13_95282317_+ | 1 | 0 | 0 | 0 | 0 |  |
| HAM/TSP#2 | 5206 | chr13_95733698_- | 6 | 0 | 0 | 0 | 0 |  |
| HAM/TSP#2 | 5207 | chr13_96554318_+ | 1 | 0 | 0 | 0 | 0 |  |
| HAM/TSP#2 | 5208 | chr13_97321094_+ | 6 | 0 | 0 | 0 | 0 |  |
| HAM/TSP#2 | 5209 | chr13_97436758_+ | 1 | 0 | 0 | 0 | 0 |  |
| HAM/TSP#2 | 5210 | chr13_97504880_+ | 12 | 1 | 0 | 0 | 0 |  |
| HAM/TSP#2 | 5211 | chr13_97848587_- | 1 | 0 | 0 | 0 | 0 |  |
| HAM/TSP#2 | 5212 | chr13_98413478_- | 1 | 0 | 0 | 0 | 0 |  |
| HAM/TSP#2 | 5213 | chr13_98413523_- | 3 | 0 | 0 | 0 | 0 |  |
| HAM/TSP#2 | 5214 | chr14_100127881_- | 1 | 0 | 0 | 0 | 0 |  |
| HAM/TSP#2 | 5215 | chr14_103164960_+ | 1 | 0 | 0 | 0 | 0 |  |
| HAM/TSP#2 | 5216 | chr14_104162243_- | 1 | 0 | 0 | 0 | 0 |  |
| HAM/TSP#2 | 5217 | chr14_104288081_- | 5 | 0 | 0 | 0 | 0 |  |
| HAM/TSP#2 | 5218 | chr14_104483884_- | 0 | 1 | 0 | 0 | 0 |  |
| HAM/TSP#2 | 5219 | chr14_104991859_+ | 1 | 0 | 0 | 0 | 0 |  |
| HAM/TSP#2 | 5220 | chr14_105224295_- | 1 | 0 | 0 | 0 | 0 |  |
| HAM/TSP#2 | 5221 | chr14_106354174_- | 2 | 0 | 0 | 0 | 0 |  |
| HAM/TSP#2 | 5222 | chr14_19927443_+ | 1 | 0 | 0 | 0 | 0 |  |
| HAM/TSP#2 | 5223 | chr14_19946569_+ | 1 | 0 | 0 | 0 | 0 |  |
| HAM/TSP#2 | 5224 | chr14_20063318_- | 2 | 0 | 0 | 0 | 0 |  |
| HAM/TSP#2 | 5225 | chr14_20457990_+ | 2 | 0 | 0 | 0 | 0 |  |
| HAM/TSP#2 | 5226 | chr14_20608163_+ | 4 | 0 | 0 | 0 | 0 |  |
| HAM/TSP#2 | 5227 | chr14_21264841_- | 1 | 0 | 0 | 0 | 0 |  |
| HAM/TSP#2 | 5228 | chr14_21312975_+ | 3 | 0 | 0 | 0 | 0 |  |
| HAM/TSP#2 | 5229 | chr14_21385920_- | 5 | 3 | 0 | 0 | 0 |  |
| HAM/TSP#2 | 5230 | chr14_23885283_- | 3 | 1 | 0 | 0 | 0 |  |
| HAM/TSP#2 | 5231 | chr14_24282689_+ | 1 | 0 | 0 | 0 | 0 |  |
| HAM/TSP#2 | 5232 | chr14_24909422_- | 4 | 0 | 0 | 0 | 0 |  |
| HAM/TSP#2 | 5233 | chr14_25612760_+ | 5 | 0 | 1 | 0 | 0 |  |
| HAM/TSP#2 | 5234 | chr14_25612764_+ | 1 | 0 | 0 | 0 | 0 |  |
| HAM/TSP#2 | 5235 | chr14_26490502_- | 1 | 0 | 0 | 0 | 0 |  |
| HAM/TSP#2 | 5236 | chr14_26504674_- | 3 | 0 | 0 | 0 | 0 |  |
| HAM/TSP#2 | 5237 | chr14_27164445_- | 1 | 0 | 0 | 0 | 0 |  |
| HAM/TSP#2 | 5238 | chr14_27407779_+ | 6 | 0 | 0 | 0 | 0 |  |
| HAM/TSP#2 | 5239 | chr14_28835135_- | 3 | 0 | 0 | 0 | 0 |  |
| HAM/TSP#2 | 5240 | chr14_29083869_+ | 1 | 0 | 0 | 0 | 0 |  |
| HAM/TSP#2 | 5241 | chr14_29908991_+ | 1 | 0 | 0 | 0 | 0 |  |
| HAM/TSP#2 | 5242 | chr14_30328991_- | 5 | 0 | 0 | 0 | 0 |  |
| HAM/TSP#2 | 5243 | chr14_31585400_+ | 12 | 0 | 0 | 0 | 0 |  |
| HAM/TSP#2 | 5244 | chr14_31969529_- | 5 | 0 | 0 | 0 | 0 |  |
| HAM/TSP#2 | 5245 | chr14_32145575_+ | 0 | 0 | 0 | 0 | 4 |  |
| HAM/TSP#2 | 5246 | chr14_32171667_+ | 1 | 0 | 0 | 0 | 0 |  |
| HAM/TSP#2 | 5247 | chr14_32599603_- | 3 | 0 | 0 | 0 | 0 |  |
| HAM/TSP#2 | 5248 | chr14_33439044_- | 0 | 1 | 0 | 0 | 0 |  |
| HAM/TSP#2 | 5249 | chr14_33620055_- | 2 | 0 | 0 | 0 | 0 |  |
| HAM/TSP#2 | 5250 | chr14_33706071_+ | 2 | 0 | 0 | 0 | 0 |  |
| HAM/TSP#2 | 5251 | chr14_35286355_- | 1 | 0 | 0 | 0 | 0 |  |
| HAM/TSP#2 | 5252 | chr14_35896067_+ | 1 | 0 | 0 | 0 | 0 |  |
| HAM/TSP#2 | 5253 | chr14_36285708_+ | 3 | 0 | 0 | 0 | 0 |  |
| HAM/TSP#2 | 5254 | chr14_36860554_+ | 2 | 0 | 0 | 0 | 0 |  |
| HAM/TSP#2 | 5255 | chr14_38652811_+ | 2 | 0 | 0 | 0 | 0 |  |
| HAM/TSP#2 | 5256 | chr14_39019650_- | 0 | 1 | 0 | 0 | 0 |  |
| HAM/TSP#2 | 5257 | chr14_39928704_+ | 16 | 0 | 0 | 0 | 0 |  |
| HAM/TSP#2 | 5258 | chr14_40466834_+ | 2 | 0 | 0 | 0 | 0 |  |
| HAM/TSP#2 | 5259 | chr14_42000819_+ | 10 | 0 | 0 | 0 | 0 |  |
| HAM/TSP#2 | 5260 | chr14_42025636_+ | 1 | 0 | 0 | 0 | 0 |  |
| HAM/TSP#2 | 5261 | chr14_43001842_- | 22 | 2 | 0 | 0 | 0 |  |
| HAM/TSP#2 | 5262 | chr14_43146909_- | 1 | 0 | 0 | 0 | 0 |  |
| HAM/TSP#2 | 5263 | chr14_44338549_- | 2 | 0 | 0 | 0 | 0 |  |
| HAM/TSP#2 | 5264 | chr14_45206280_- | 3 | 0 | 0 | 0 | 0 |  |
| HAM/TSP#2 | 5265 | chr14_45735494_+ | 1 | 0 | 0 | 0 | 0 |  |
| HAM/TSP#2 | 5266 | chr14_45969343_+ | 7 | 0 | 0 | 0 | 0 |  |
| HAM/TSP#2 | 5267 | chr14_46541087_+ | 0 | 1 | 0 | 0 | 0 |  |
| HAM/TSP#2 | 5268 | chr14_46726345_- | 0 | 2 | 0 | 0 | 0 |  |
| HAM/TSP#2 | 5269 | chr14_46741803_+ | 1 | 0 | 0 | 0 | 0 |  |
| HAM/TSP#2 | 5270 | chr14_47022302_+ | 7 | 0 | 0 | 0 | 0 |  |
| HAM/TSP#2 | 5271 | chr14_47045829_+ | 2 | 0 | 0 | 0 | 0 |  |
| HAM/TSP#2 | 5272 | chr14_47059998_+ | 2 | 0 | 0 | 0 | 0 |  |
| HAM/TSP#2 | 5273 | chr14_47676440_- | 3 | 0 | 0 | 0 | 0 |  |
| HAM/TSP#2 | 5274 | chr14_47787762_- | 1 | 0 | 0 | 0 | 0 |  |
| HAM/TSP#2 | 5275 | chr14_50981860_- | 1 | 0 | 0 | 0 | 0 |  |
| HAM/TSP#2 | 5276 | chr14_51212346_+ | 2 | 0 | 0 | 0 | 0 |  |
| HAM/TSP#2 | 5277 | chr14_51248781_+ | 1 | 0 | 0 | 0 | 0 |  |
| HAM/TSP#2 | 5278 | chr14_51641612_+ | 5 | 0 | 0 | 0 | 0 |  |
| HAM/TSP#2 | 5279 | chr14_52007172_- | 0 | 4 | 0 | 0 | 0 |  |
| HAM/TSP#2 | 5280 | chr14_52800555_+ | 16 | 0 | 0 | 0 | 2 |  |
| HAM/TSP#2 | 5281 | chr14_54628576_+ | 1 | 0 | 0 | 0 | 0 |  |
| HAM/TSP#2 | 5282 | chr14_54802468_+ | 2 | 0 | 0 | 0 | 0 |  |
| HAM/TSP#2 | 5283 | chr14_56159852_- | 7 | 0 | 0 | 0 | 0 |  |
| HAM/TSP#2 | 5284 | chr14_56869164_+ | 0 | 1 | 0 | 0 | 0 |  |
| HAM/TSP#2 | 5285 | chr14_56973042_- | 2 | 0 | 0 | 0 | 0 |  |
| HAM/TSP#2 | 5286 | chr14_57394234_+ | 1 | 0 | 0 | 0 | 0 |  |
| HAM/TSP#2 | 5287 | chr14_58446738_- | 1 | 0 | 0 | 0 | 0 |  |
| HAM/TSP#2 | 5288 | chr14_61038405_+ | 3 | 0 | 0 | 0 | 0 |  |
| HAM/TSP#2 | 5289 | chr14_61867520_- | 2 | 0 | 0 | 0 | 0 |  |
| HAM/TSP#2 | 5290 | chr14_63148300_- | 1 | 0 | 0 | 0 | 0 |  |
| HAM/TSP#2 | 5291 | chr14_64956301_- | 1 | 0 | 0 | 0 | 0 |  |
| HAM/TSP#2 | 5292 | chr14_65208820_- | 2 | 0 | 0 | 0 | 0 |  |
| HAM/TSP#2 | 5293 | chr14_65343497_+ | 13 | 2 | 0 | 0 | 0 |  |
| HAM/TSP#2 | 5294 | chr14_65451117_+ | 1 | 0 | 0 | 0 | 0 |  |
| HAM/TSP#2 | 5295 | chr14_65737700_- | 3 | 0 | 0 | 0 | 0 |  |
| HAM/TSP#2 | 5296 | chr14_67368058_+ | 3 | 0 | 0 | 0 | 0 |  |
| HAM/TSP#2 | 5297 | chr14_67464123_+ | 0 | 16 | 0 | 0 | 0 |  |
| HAM/TSP#2 | 5298 | chr14_68920685_+ | 1 | 0 | 0 | 0 | 0 |  |
| HAM/TSP#2 | 5299 | chr14_69781487_+ | 1 | 0 | 0 | 0 | 0 |  |
| HAM/TSP#2 | 5300 | chr14_70650866_+ | 4 | 0 | 0 | 0 | 0 |  |
| HAM/TSP#2 | 5301 | chr14_73024856_+ | 1 | 0 | 0 | 0 | 0 |  |
| HAM/TSP#2 | 5302 | chr14_73379505_- | 0 | 5 | 0 | 0 | 0 |  |
| HAM/TSP#2 | 5303 | chr14_75292322_+ | 3 | 0 | 0 | 0 | 0 |  |
| HAM/TSP#2 | 5304 | chr14_75749336_+ | 1 | 0 | 0 | 0 | 0 |  |
| HAM/TSP#2 | 5305 | chr14_75832269_- | 3 | 0 | 0 | 0 | 0 |  |
| HAM/TSP#2 | 5306 | chr14_76957452_+ | 3 | 0 | 0 | 0 | 0 |  |
| HAM/TSP#2 | 5307 | chr14_77206359_+ | 1 | 0 | 0 | 0 | 0 |  |
| HAM/TSP#2 | 5308 | chr14_78282762_+ | 0 | 1 | 0 | 0 | 0 |  |
| HAM/TSP#2 | 5309 | chr14_78936260_- | 3 | 0 | 0 | 0 | 0 |  |
| HAM/TSP#2 | 5310 | chr14_79786063_- | 1 | 0 | 0 | 0 | 0 |  |
| HAM/TSP#2 | 5311 | chr14_80069147_- | 3 | 0 | 0 | 0 | 0 |  |
| HAM/TSP#2 | 5312 | chr14_80299935_- | 2 | 0 | 0 | 0 | 0 |  |
| HAM/TSP#2 | 5313 | chr14_81086406_+ | 1 | 0 | 0 | 0 | 0 |  |
| HAM/TSP#2 | 5314 | chr14_81613630_+ | 4 | 0 | 0 | 0 | 0 |  |
| HAM/TSP#2 | 5315 | chr14_83964507_+ | 0 | 1 | 0 | 0 | 0 |  |
| HAM/TSP#2 | 5316 | chr14_84268471_- | 7 | 0 | 0 | 0 | 0 |  |
| HAM/TSP#2 | 5317 | chr14_84906306_- | 0 | 4 | 0 | 0 | 0 |  |
| HAM/TSP#2 | 5318 | chr14_85935867_+ | 2 | 0 | 0 | 0 | 0 |  |
| HAM/TSP#2 | 5319 | chr14_86865289_- | 1 | 0 | 0 | 0 | 0 |  |
| HAM/TSP#2 | 5320 | chr14_90058908_- | 2 | 0 | 0 | 0 | 0 |  |
| HAM/TSP#2 | 5321 | chr14_90248005_- | 3 | 0 | 0 | 0 | 0 |  |
| HAM/TSP#2 | 5322 | chr14_90676777_+ | 1 | 0 | 0 | 0 | 0 |  |
| HAM/TSP#2 | 5323 | chr14_91268262_- | 1 | 0 | 0 | 0 | 0 |  |
| HAM/TSP#2 | 5324 | chr14_92040302_+ | 1 | 0 | 0 | 0 | 0 |  |
| HAM/TSP#2 | 5325 | chr14_93012584_+ | 1 | 0 | 0 | 0 | 0 |  |
| HAM/TSP#2 | 5326 | chr14_94534571_+ | 4 | 0 | 0 | 0 | 0 |  |
| HAM/TSP#2 | 5327 | chr14_94724674_- | 1 | 0 | 0 | 0 | 0 |  |
| HAM/TSP#2 | 5328 | chr14_95464047_- | 0 | 0 | 0 | 47 | 0 |  |
| HAM/TSP#2 | 5329 | chr14_96112832_+ | 1 | 0 | 0 | 0 | 0 |  |
| HAM/TSP#2 | 5330 | chr14_96947342_- | 1 | 0 | 0 | 0 | 0 |  |
| HAM/TSP#2 | 5331 | chr14_98123053_+ | 1 | 0 | 0 | 0 | 0 |  |
| HAM/TSP#2 | 5332 | chr14_99014515_+ | 1 | 0 | 0 | 0 | 0 |  |
| HAM/TSP#2 | 5333 | chr15_100707135_- | 0 | 3 | 0 | 0 | 0 |  |
| HAM/TSP#2 | 5334 | chr15_101134850_+ | 1 | 0 | 0 | 0 | 0 |  |
| HAM/TSP#2 | 5335 | chr15_101742561_+ | 3 | 0 | 0 | 0 | 0 |  |
| HAM/TSP#2 | 5336 | chr15_18400762_+ | 1 | 0 | 0 | 0 | 0 |  |
| HAM/TSP#2 | 5337 | chr15_20268959_- | 1 | 0 | 0 | 0 | 0 |  |
| HAM/TSP#2 | 5338 | chr15_20390065_- | 5 | 0 | 0 | 0 | 0 |  |
| HAM/TSP#2 | 5339 | chr15_23909425_+ | 1 | 0 | 0 | 0 | 0 |  |
| HAM/TSP#2 | 5340 | chr15_24145901_+ | 2 | 0 | 0 | 0 | 0 |  |
| HAM/TSP#2 | 5341 | chr15_25638887_+ | 1 | 0 | 0 | 0 | 0 |  |
| HAM/TSP#2 | 5342 | chr15_25857980_- | 0 | 3 | 0 | 0 | 0 |  |
| HAM/TSP#2 | 5343 | chr15_25862011_+ | 0 | 0 | 0 | 1 | 0 |  |
| HAM/TSP#2 | 5344 | chr15_26816282_- | 3 | 0 | 0 | 0 | 0 |  |
| HAM/TSP#2 | 5345 | chr15_26831775_- | 1 | 0 | 0 | 0 | 0 |  |
| HAM/TSP#2 | 5346 | chr15_27237998_+ | 1 | 0 | 0 | 0 | 0 |  |
| HAM/TSP#2 | 5347 | chr15_28887651_- | 0 | 1 | 0 | 0 | 0 |  |
| HAM/TSP#2 | 5348 | chr15_31552928_+ | 1 | 0 | 0 | 0 | 0 |  |
| HAM/TSP#2 | 5349 | chr15_31560365_- | 1 | 0 | 0 | 0 | 0 |  |
| HAM/TSP#2 | 5350 | chr15_31784247_+ | 2 | 0 | 0 | 0 | 0 |  |
| HAM/TSP#2 | 5351 | chr15_33203909_+ | 1 | 0 | 0 | 0 | 0 |  |
| HAM/TSP#2 | 5352 | chr15_36557515_+ | 1 | 0 | 0 | 0 | 0 |  |
| HAM/TSP#2 | 5353 | chr15_36834430_+ | 1 | 0 | 0 | 0 | 0 |  |
| HAM/TSP#2 | 5354 | chr15_37597419_- | 1 | 0 | 0 | 0 | 0 |  |
| HAM/TSP#2 | 5355 | chr15_38085057_+ | 12 | 1 | 0 | 0 | 0 |  |
| HAM/TSP#2 | 5356 | chr15_38697695_- | 1 | 0 | 0 | 0 | 0 |  |
| HAM/TSP#2 | 5357 | chr15_38966325_- | 4 | 0 | 0 | 0 | 0 |  |
| HAM/TSP#2 | 5358 | chr15_39123844_+ | 1 | 0 | 0 | 0 | 0 |  |
| HAM/TSP#2 | 5359 | chr15_39210462_- | 2 | 0 | 0 | 0 | 0 |  |
| HAM/TSP#2 | 5360 | chr15_39430348_- | 3 | 0 | 0 | 0 | 0 |  |
| HAM/TSP#2 | 5361 | chr15_40545170_- | 0 | 1 | 0 | 0 | 0 |  |
| HAM/TSP#2 | 5362 | chr15_41309145_+ | 1 | 0 | 0 | 0 | 0 |  |
| HAM/TSP#2 | 5363 | chr15_41571843_+ | 0 | 1 | 0 | 0 | 0 |  |
| HAM/TSP#2 | 5364 | chr15_44456602_+ | 3 | 0 | 0 | 0 | 0 |  |
| HAM/TSP#2 | 5365 | chr15_44499379_- | 1 | 0 | 0 | 0 | 0 |  |
| HAM/TSP#2 | 5366 | chr15_46632959_- | 2 | 0 | 0 | 0 | 0 |  |
| HAM/TSP#2 | 5367 | chr15_46690381_+ | 1 | 0 | 0 | 0 | 0 |  |
| HAM/TSP#2 | 5368 | chr15_47196961_+ | 1 | 0 | 0 | 0 | 0 |  |
| HAM/TSP#2 | 5369 | chr15_47388516_- | 2 | 0 | 0 | 0 | 0 |  |
| HAM/TSP#2 | 5370 | chr15_47725972_- | 4 | 0 | 0 | 0 | 0 |  |
| HAM/TSP#2 | 5371 | chr15_48668697_+ | 8 | 0 | 0 | 0 | 0 |  |
| HAM/TSP#2 | 5372 | chr15_48806133_- | 1 | 0 | 0 | 0 | 0 |  |
| HAM/TSP#2 | 5373 | chr15_51551898_- | 2 | 0 | 0 | 0 | 0 |  |
| HAM/TSP#2 | 5374 | chr15_53243284_- | 6 | 0 | 0 | 0 | 0 |  |
| HAM/TSP#2 | 5375 | chr15_54034836_- | 5 | 0 | 0 | 0 | 0 |  |
| HAM/TSP#2 | 5376 | chr15_56022556_+ | 12 | 0 | 0 | 0 | 0 |  |
| HAM/TSP#2 | 5377 | chr15_56747827_- | 3 | 0 | 0 | 0 | 0 |  |
| HAM/TSP#2 | 5378 | chr15_58303157_- | 2 | 0 | 0 | 0 | 0 |  |
| HAM/TSP#2 | 5379 | chr15_59557880_- | 0 | 1 | 0 | 0 | 0 |  |
| HAM/TSP#2 | 5380 | chr15_62367208_+ | 17 | 0 | 0 | 0 | 0 |  |
| HAM/TSP#2 | 5381 | chr15_64352134_+ | 2 | 0 | 0 | 0 | 0 |  |
| HAM/TSP#2 | 5382 | chr15_64473502_+ | 0 | 1 | 0 | 0 | 0 |  |
| HAM/TSP#2 | 5383 | chr15_64528294_+ | 1 | 0 | 0 | 0 | 0 |  |
| HAM/TSP#2 | 5384 | chr15_65778794_- | 1 | 0 | 0 | 0 | 0 |  |
| HAM/TSP#2 | 5385 | chr15_65917753_- | 1 | 0 | 0 | 0 | 0 |  |
| HAM/TSP#2 | 5386 | chr15_66181848_- | 1 | 0 | 0 | 0 | 0 |  |
| HAM/TSP#2 | 5387 | chr15_66634845_+ | 1 | 0 | 0 | 0 | 0 |  |
| HAM/TSP#2 | 5388 | chr15_66840104_- | 1 | 0 | 0 | 0 | 0 |  |
| HAM/TSP#2 | 5389 | chr15_69184082_+ | 1 | 0 | 0 | 0 | 0 |  |
| HAM/TSP#2 | 5390 | chr15_69752261_- | 1 | 0 | 0 | 0 | 0 |  |
| HAM/TSP#2 | 5391 | chr15_69874969_+ | 1 | 0 | 0 | 0 | 0 |  |
| HAM/TSP#2 | 5392 | chr15_69903376_- | 1 | 0 | 0 | 0 | 0 |  |
| HAM/TSP#2 | 5393 | chr15_70064153_+ | 0 | 1 | 0 | 0 | 0 |  |
| HAM/TSP#2 | 5394 | chr15_71148288_+ | 1 | 0 | 0 | 0 | 0 |  |
| HAM/TSP#2 | 5395 | chr15_71297428_- | 1 | 0 | 0 | 0 | 0 |  |
| HAM/TSP#2 | 5396 | chr15_71860216_- | 5 | 0 | 0 | 0 | 0 |  |
| HAM/TSP#2 | 5397 | chr15_72318215_+ | 1 | 0 | 0 | 0 | 0 |  |
| HAM/TSP#2 | 5398 | chr15_75300671_- | 1 | 0 | 0 | 0 | 0 |  |
| HAM/TSP#2 | 5399 | chr15_77813331_+ | 1 | 0 | 0 | 0 | 0 |  |
| HAM/TSP#2 | 5400 | chr15_81301265_- | 15 | 0 | 0 | 0 | 1 |  |
| HAM/TSP#2 | 5401 | chr15_81562683_- | 1 | 0 | 0 | 0 | 0 |  |
| HAM/TSP#2 | 5402 | chr15_81611842_+ | 9 | 0 | 0 | 0 | 0 |  |
| HAM/TSP#2 | 5403 | chr15_82345993_- | 0 | 3 | 0 | 0 | 0 |  |
| HAM/TSP#2 | 5404 | chr15_83321460_+ | 1 | 0 | 0 | 0 | 0 |  |
| HAM/TSP#2 | 5405 | chr15_84770120_- | 1 | 0 | 0 | 0 | 0 |  |
| HAM/TSP#2 | 5406 | chr15_85590304_- | 2 | 0 | 0 | 0 | 0 |  |
| HAM/TSP#2 | 5407 | chr15_86344337_- | 13 | 0 | 0 | 0 | 0 |  |
| HAM/TSP#2 | 5408 | chr15_87801997_+ | 0 | 1 | 0 | 0 | 0 |  |
| HAM/TSP#2 | 5409 | chr15_89859088_- | 1 | 0 | 0 | 0 | 0 |  |
| HAM/TSP#2 | 5410 | chr15_91056442_+ | 1 | 0 | 0 | 0 | 0 |  |
| HAM/TSP#2 | 5411 | chr15_92103713_- | 1 | 1 | 0 | 0 | 0 |  |
| HAM/TSP#2 | 5412 | chr15_93262211_- | 2 | 0 | 0 | 0 | 0 |  |
| HAM/TSP#2 | 5413 | chr15_93418358_- | 0 | 1 | 0 | 0 | 0 |  |
| HAM/TSP#2 | 5414 | chr15_97326671_+ | 1 | 0 | 0 | 0 | 0 |  |
| HAM/TSP#2 | 5415 | chr15_99015112_+ | 3 | 0 | 0 | 0 | 0 |  |
| HAM/TSP#2 | 5416 | chr16_10380073_+ | 1 | 0 | 0 | 0 | 0 |  |
| HAM/TSP#2 | 5417 | chr16_1042461_- | 1 | 0 | 0 | 0 | 0 |  |
| HAM/TSP#2 | 5418 | chr16_14359038_- | 0 | 0 | 0 | 1 | 0 |  |
| HAM/TSP#2 | 5419 | chr16_16619148_- | 1 | 0 | 0 | 0 | 0 |  |
| HAM/TSP#2 | 5420 | chr16_1763386_- | 1 | 0 | 0 | 0 | 0 |  |
| HAM/TSP#2 | 5421 | chr16_19403918_+ | 1 | 0 | 0 | 0 | 0 |  |
| HAM/TSP#2 | 5422 | chr16_19625360_- | 1 | 0 | 0 | 0 | 0 |  |
| HAM/TSP#2 | 5423 | chr16_22690992_- | 1 | 0 | 0 | 0 | 0 |  |
| HAM/TSP#2 | 5424 | chr16_23917422_- | 1 | 0 | 0 | 0 | 0 |  |
| HAM/TSP#2 | 5425 | chr16_24586410_+ | 1 | 0 | 0 | 0 | 0 |  |
| HAM/TSP#2 | 5426 | chr16_24884509_- | 0 | 13 | 0 | 0 | 0 |  |
| HAM/TSP#2 | 5427 | chr16_25010199_+ | 4 | 0 | 0 | 0 | 0 |  |
| HAM/TSP#2 | 5428 | chr16_27142360_- | 1 | 0 | 0 | 0 | 0 |  |
| HAM/TSP#2 | 5429 | chr16_27859370_+ | 1 | 0 | 0 | 0 | 0 |  |
| HAM/TSP#2 | 5430 | chr16_28957915_- | 1 | 0 | 0 | 0 | 0 |  |
| HAM/TSP#2 | 5431 | chr16_31474977_- | 5 | 0 | 0 | 0 | 0 |  |
| HAM/TSP#2 | 5432 | chr16_31712807_+ | 0 | 1 | 0 | 0 | 0 |  |
| HAM/TSP#2 | 5433 | chr16_34572888_- | 2 | 0 | 0 | 0 | 0 |  |
| HAM/TSP#2 | 5434 | chr16_34671635_+ | 1 | 0 | 0 | 0 | 0 |  |
| HAM/TSP#2 | 5435 | chr16_35326968_+ | 1 | 0 | 0 | 0 | 0 |  |
| HAM/TSP#2 | 5436 | chr16_36563345_- | 1 | 0 | 0 | 0 | 0 |  |
| HAM/TSP#2 | 5437 | chr16_38029832_- | 1 | 0 | 0 | 0 | 0 |  |
| HAM/TSP#2 | 5438 | chr16_4479889_- | 2 | 0 | 0 | 0 | 0 |  |
| HAM/TSP#2 | 5439 | chr16_46387835_+ | 3 | 0 | 0 | 0 | 0 |  |
| HAM/TSP#2 | 5440 | chr16_46390790_- | 2 | 0 | 0 | 0 | 0 |  |
| HAM/TSP#2 | 5441 | chr16_46399033_- | 1 | 0 | 0 | 0 | 0 |  |
| HAM/TSP#2 | 5442 | chr16_46400954_- | 1 | 0 | 0 | 0 | 0 |  |
| HAM/TSP#2 | 5443 | chr16_46697368_- | 3 | 0 | 0 | 0 | 0 |  |
| HAM/TSP#2 | 5444 | chr16_46924561_- | 0 | 0 | 0 | 0 | 1 |  |
| HAM/TSP#2 | 5445 | chr16_47744238_- | 1 | 0 | 0 | 0 | 0 |  |
| HAM/TSP#2 | 5446 | chr16_4775456_+ | 0 | 1 | 0 | 0 | 0 |  |
| HAM/TSP#2 | 5447 | chr16_47911516_- | 8 | 0 | 0 | 0 | 0 |  |
| HAM/TSP#2 | 5448 | chr16_49903351_- | 2 | 0 | 0 | 0 | 0 |  |
| HAM/TSP#2 | 5449 | chr16_51187043_+ | 1 | 0 | 0 | 0 | 0 |  |
| HAM/TSP#2 | 5450 | chr16_51531161_+ | 1 | 0 | 0 | 0 | 0 |  |
| HAM/TSP#2 | 5451 | chr16_52257044_- | 0 | 3 | 0 | 0 | 0 |  |
| HAM/TSP#2 | 5452 | chr16_52529719_- | 2 | 0 | 0 | 0 | 0 |  |
| HAM/TSP#2 | 5453 | chr16_53142027_- | 2 | 0 | 0 | 0 | 0 |  |
| HAM/TSP#2 | 5454 | chr16_54105677_- | 1 | 0 | 0 | 0 | 0 |  |
| HAM/TSP#2 | 5455 | chr16_54391682_+ | 1 | 0 | 0 | 0 | 0 |  |
| HAM/TSP#2 | 5456 | chr16_55507338_+ | 1 | 0 | 0 | 0 | 0 |  |
| HAM/TSP#2 | 5457 | chr16_55529558_- | 1 | 0 | 0 | 0 | 0 |  |
| HAM/TSP#2 | 5458 | chr16_5577296_- | 2 | 0 | 0 | 0 | 0 |  |
| HAM/TSP#2 | 5459 | chr16_56196766_- | 0 | 10 | 0 | 0 | 0 |  |
| HAM/TSP#2 | 5460 | chr16_56848159_- | 2 | 0 | 0 | 0 | 0 |  |
| HAM/TSP#2 | 5461 | chr16_58454064_- | 13 | 0 | 0 | 0 | 0 |  |
| HAM/TSP#2 | 5462 | chr16_59143670_+ | 1 | 0 | 0 | 0 | 0 |  |
| HAM/TSP#2 | 5463 | chr16_59438650_- | 5 | 0 | 0 | 0 | 0 |  |
| HAM/TSP#2 | 5464 | chr16_59524451_+ | 0 | 1 | 0 | 0 | 0 |  |
| HAM/TSP#2 | 5465 | chr16_59545919_- | 1 | 0 | 0 | 0 | 0 |  |
| HAM/TSP#2 | 5466 | chr16_60116899_+ | 4 | 0 | 0 | 0 | 0 |  |
| HAM/TSP#2 | 5467 | chr16_60400219_+ | 8 | 0 | 0 | 0 | 0 |  |
| HAM/TSP#2 | 5468 | chr16_60704281_+ | 2 | 0 | 0 | 0 | 0 |  |
| HAM/TSP#2 | 5469 | chr16_62736986_- | 4 | 0 | 0 | 0 | 0 |  |
| HAM/TSP#2 | 5470 | chr16_63549321_- | 1 | 0 | 0 | 0 | 0 |  |
| HAM/TSP#2 | 5471 | chr16_63918957_+ | 2 | 0 | 0 | 0 | 0 |  |
| HAM/TSP#2 | 5472 | chr16_64087902_- | 3 | 0 | 0 | 0 | 0 |  |
| HAM/TSP#2 | 5473 | chr16_64207819_+ | 3 | 0 | 0 | 0 | 0 |  |
| HAM/TSP#2 | 5474 | chr16_64242773_- | 1 | 0 | 0 | 0 | 0 |  |
| HAM/TSP#2 | 5475 | chr16_64329864_+ | 1 | 0 | 0 | 0 | 0 |  |
| HAM/TSP#2 | 5476 | chr16_65122425_+ | 0 | 1 | 0 | 0 | 0 |  |
| HAM/TSP#2 | 5477 | chr16_65182904_+ | 1 | 0 | 0 | 0 | 0 |  |
| HAM/TSP#2 | 5478 | chr16_65927598_- | 3 | 0 | 0 | 0 | 0 |  |
| HAM/TSP#2 | 5479 | chr16_66035474_- | 4 | 0 | 0 | 0 | 0 |  |
| HAM/TSP#2 | 5480 | chr16_6697845_- | 8 | 0 | 0 | 0 | 0 |  |
| HAM/TSP#2 | 5481 | chr16_67951120_+ | 6 | 0 | 0 | 0 | 0 |  |
| HAM/TSP#2 | 5482 | chr16_681884_- | 1 | 0 | 0 | 0 | 0 |  |
| HAM/TSP#2 | 5483 | chr16_68359444_- | 16 | 0 | 0 | 0 | 0 |  |
| HAM/TSP#2 | 5484 | chr16_71898556_- | 1 | 0 | 0 | 0 | 0 |  |
| HAM/TSP#2 | 5485 | chr16_72766816_- | 3 | 0 | 0 | 0 | 0 |  |
| HAM/TSP#2 | 5486 | chr16_72997396_+ | 1 | 0 | 0 | 0 | 0 |  |
| HAM/TSP#2 | 5487 | chr16_73695730_+ | 1 | 0 | 0 | 0 | 0 |  |
| HAM/TSP#2 | 5488 | chr16_75459519_- | 1 | 0 | 0 | 0 | 0 |  |
| HAM/TSP#2 | 5489 | chr16_76091708_- | 3 | 0 | 0 | 0 | 0 |  |
| HAM/TSP#2 | 5490 | chr16_76181532_+ | 1 | 0 | 0 | 0 | 0 |  |
| HAM/TSP#2 | 5491 | chr16_76801509_- | 3 | 0 | 0 | 0 | 0 |  |
| HAM/TSP#2 | 5492 | chr16_77196035_- | 1 | 0 | 0 | 0 | 0 |  |
| HAM/TSP#2 | 5493 | chr16_78183541_- | 1 | 0 | 0 | 0 | 0 |  |
| HAM/TSP#2 | 5494 | chr16_79466345_- | 5 | 0 | 0 | 0 | 0 |  |
| HAM/TSP#2 | 5495 | chr16_79708987_+ | 1 | 0 | 0 | 0 | 0 |  |
| HAM/TSP#2 | 5496 | chr16_8000839_+ | 10 | 0 | 0 | 0 | 0 |  |
| HAM/TSP#2 | 5497 | chr16_80085399_- | 2 | 0 | 0 | 0 | 0 |  |
| HAM/TSP#2 | 5498 | chr16_80733549_- | 5 | 0 | 0 | 0 | 0 |  |
| HAM/TSP#2 | 5499 | chr16_80983006_- | 2 | 0 | 0 | 0 | 0 |  |
| HAM/TSP#2 | 5500 | chr16_81893786_- | 1 | 0 | 0 | 0 | 0 |  |
| HAM/TSP#2 | 5501 | chr16_82128989_+ | 3 | 0 | 0 | 0 | 0 |  |
| HAM/TSP#2 | 5502 | chr16_82212148_+ | 1 | 0 | 0 | 0 | 0 |  |
| HAM/TSP#2 | 5503 | chr16_82223179_- | 1 | 0 | 0 | 0 | 0 |  |
| HAM/TSP#2 | 5504 | chr16_82655187_+ | 2 | 0 | 0 | 0 | 0 |  |
| HAM/TSP#2 | 5505 | chr16_82837142_- | 2 | 0 | 0 | 0 | 0 |  |
| HAM/TSP#2 | 5506 | chr16_82883625_- | 4 | 0 | 0 | 0 | 0 |  |
| HAM/TSP#2 | 5507 | chr16_82894855_- | 2 | 0 | 0 | 0 | 0 |  |
| HAM/TSP#2 | 5508 | chr16_82976090_+ | 1 | 0 | 0 | 0 | 0 |  |
| HAM/TSP#2 | 5509 | chr16_8365951_+ | 1 | 0 | 0 | 0 | 0 |  |
| HAM/TSP#2 | 5510 | chr16_84950019_- | 1 | 0 | 0 | 0 | 0 |  |
| HAM/TSP#2 | 5511 | chr16_85225354_- | 1 | 0 | 0 | 0 | 0 |  |
| HAM/TSP#2 | 5512 | chr16_85445558_- | 1 | 0 | 0 | 0 | 0 |  |
| HAM/TSP#2 | 5513 | chr16_85840171_+ | 1 | 0 | 0 | 0 | 0 |  |
| HAM/TSP#2 | 5514 | chr16_85890158_- | 1 | 0 | 0 | 0 | 0 |  |
| HAM/TSP#2 | 5515 | chr16_86718200_+ | 1 | 0 | 0 | 0 | 0 |  |
| HAM/TSP#2 | 5516 | chr16_87648354_+ | 1 | 0 | 0 | 0 | 0 |  |
| HAM/TSP#2 | 5517 | chr16_87703758_+ | 1 | 0 | 0 | 0 | 0 |  |
| HAM/TSP#2 | 5518 | chr16_88538932_+ | 1 | 0 | 0 | 0 | 0 |  |
| HAM/TSP#2 | 5519 | chr16_88736194_- | 0 | 6 | 0 | 0 | 0 |  |
| HAM/TSP#2 | 5520 | chr16_89230371_+ | 6 | 0 | 0 | 0 | 0 |  |
| HAM/TSP#2 | 5521 | chr17_10071350_+ | 1 | 0 | 0 | 0 | 0 |  |
| HAM/TSP#2 | 5522 | chr17_10189729_+ | 1 | 0 | 0 | 0 | 0 |  |
| HAM/TSP#2 | 5523 | chr17_10323569_- | 1 | 0 | 0 | 0 | 0 |  |
| HAM/TSP#2 | 5524 | chr17_10733757_- | 1 | 0 | 0 | 0 | 0 |  |
| HAM/TSP#2 | 5525 | chr17_12196712_+ | 2 | 0 | 0 | 0 | 0 |  |
| HAM/TSP#2 | 5526 | chr17_12545413_- | 0 | 1 | 0 | 0 | 0 |  |
| HAM/TSP#2 | 5527 | chr17_13273188_+ | 10 | 0 | 0 | 0 | 0 |  |
| HAM/TSP#2 | 5528 | chr17_1464195_+ | 18 | 0 | 0 | 0 | 0 |  |
| HAM/TSP#2 | 5529 | chr17_15393997_+ | 1 | 0 | 0 | 0 | 0 |  |
| HAM/TSP#2 | 5530 | chr17_16521072_- | 0 | 1 | 0 | 0 | 0 |  |
| HAM/TSP#2 | 5531 | chr17_17857764_+ | 1 | 0 | 0 | 0 | 0 |  |
| HAM/TSP#2 | 5532 | chr17_20675121_- | 1 | 0 | 0 | 0 | 0 |  |
| HAM/TSP#2 | 5533 | chr17_21320024_- | 1 | 0 | 0 | 0 | 0 |  |
| HAM/TSP#2 | 5534 | chr17_21514798_+ | 1 | 0 | 0 | 0 | 0 |  |
| HAM/TSP#2 | 5535 | chr17_22501892_- | 9 | 0 | 0 | 0 | 0 |  |
| HAM/TSP#2 | 5536 | chr17_2381559_- | 1 | 0 | 0 | 0 | 0 |  |
| HAM/TSP#2 | 5537 | chr17_2387367_- | 1 | 0 | 0 | 0 | 0 |  |
| HAM/TSP#2 | 5538 | chr17_26603715_+ | 0 | 1 | 0 | 0 | 0 |  |
| HAM/TSP#2 | 5539 | chr17_26855001_- | 3 | 0 | 0 | 0 | 0 |  |
| HAM/TSP#2 | 5540 | chr17_27003732_+ | 1 | 0 | 0 | 0 | 0 |  |
| HAM/TSP#2 | 5541 | chr17_2721454_+ | 1 | 0 | 0 | 0 | 0 |  |
| HAM/TSP#2 | 5542 | chr17_28806395_+ | 10 | 0 | 0 | 13 | 1 |  |
| HAM/TSP#2 | 5543 | chr17_30635121_- | 0 | 0 | 0 | 0 | 1 |  |
| HAM/TSP#2 | 5544 | chr17_31385888_+ | 1 | 0 | 0 | 0 | 0 |  |
| HAM/TSP#2 | 5545 | chr17_3169969_+ | 2 | 0 | 0 | 0 | 0 |  |
| HAM/TSP#2 | 5546 | chr17_33161263_+ | 1 | 0 | 0 | 0 | 0 |  |
| HAM/TSP#2 | 5547 | chr17_33800189_- | 1 | 0 | 0 | 0 | 0 |  |
| HAM/TSP#2 | 5548 | chr17_33874752_+ | 3 | 0 | 0 | 0 | 0 |  |
| HAM/TSP#2 | 5549 | chr17_34751241_- | 1 | 0 | 0 | 0 | 0 |  |
| HAM/TSP#2 | 5550 | chr17_38537575_+ | 0 | 1 | 0 | 0 | 0 |  |
| HAM/TSP#2 | 5551 | chr17_40590294_+ | 1 | 0 | 0 | 0 | 0 |  |
| HAM/TSP#2 | 5552 | chr17_40818314_+ | 2 | 0 | 0 | 0 | 0 |  |
| HAM/TSP#2 | 5553 | chr17_4152124_- | 1 | 0 | 0 | 0 | 0 |  |
| HAM/TSP#2 | 5554 | chr17_42390282_- | 0 | 1 | 0 | 0 | 0 |  |
| HAM/TSP#2 | 5555 | chr17_441287_+ | 1 | 0 | 0 | 0 | 0 |  |
| HAM/TSP#2 | 5556 | chr17_45221242_+ | 1 | 0 | 0 | 0 | 0 |  |
| HAM/TSP#2 | 5557 | chr17_4523905_+ | 0 | 2 | 0 | 0 | 0 |  |
| HAM/TSP#2 | 5558 | chr17_45851926_+ | 0 | 1 | 0 | 0 | 0 |  |
| HAM/TSP#2 | 5559 | chr17_4675867_+ | 1 | 0 | 0 | 0 | 0 |  |
| HAM/TSP#2 | 5560 | chr17_47105865_+ | 1 | 0 | 0 | 0 | 0 |  |
| HAM/TSP#2 | 5561 | chr17_47955508_+ | 0 | 1 | 0 | 0 | 0 |  |
| HAM/TSP#2 | 5562 | chr17_50025165_- | 0 | 2 | 0 | 0 | 0 |  |
| HAM/TSP#2 | 5563 | chr17_52466471_- | 0 | 1 | 0 | 0 | 0 |  |
| HAM/TSP#2 | 5564 | chr17_54648346_- | 2 | 0 | 0 | 0 | 0 |  |
| HAM/TSP#2 | 5565 | chr17_55245716_+ | 2 | 0 | 0 | 0 | 0 |  |
| HAM/TSP#2 | 5566 | chr17_5591103_+ | 7 | 0 | 0 | 0 | 0 |  |
| HAM/TSP#2 | 5567 | chr17_59571326_+ | 1 | 0 | 0 | 0 | 0 |  |
| HAM/TSP#2 | 5568 | chr17_6037552_+ | 1 | 0 | 0 | 0 | 0 |  |
| HAM/TSP#2 | 5569 | chr17_61163491_- | 2 | 0 | 0 | 0 | 0 |  |
| HAM/TSP#2 | 5570 | chr17_63956755_- | 2 | 1 | 0 | 0 | 0 |  |
| HAM/TSP#2 | 5571 | chr17_64170006_- | 0 | 1 | 0 | 0 | 0 |  |
| HAM/TSP#2 | 5572 | chr17_6456346_- | 0 | 3 | 0 | 0 | 0 |  |
| HAM/TSP#2 | 5573 | chr17_65311693_+ | 1 | 0 | 0 | 0 | 0 |  |
| HAM/TSP#2 | 5574 | chr17_65434760_+ | 8 | 0 | 0 | 0 | 0 |  |
| HAM/TSP#2 | 5575 | chr17_65882428_- | 10 | 0 | 0 | 0 | 0 |  |
| HAM/TSP#2 | 5576 | chr17_6740966_+ | 2 | 0 | 0 | 0 | 0 |  |
| HAM/TSP#2 | 5577 | chr17_67621733_- | 1 | 0 | 0 | 0 | 0 |  |
| HAM/TSP#2 | 5578 | chr17_68049364_- | 2 | 0 | 0 | 0 | 0 |  |
| HAM/TSP#2 | 5579 | chr17_69526668_+ | 1 | 0 | 0 | 0 | 0 |  |
| HAM/TSP#2 | 5580 | chr17_69984332_- | 1 | 0 | 0 | 0 | 0 |  |
| HAM/TSP#2 | 5581 | chr17_7011248_+ | 2 | 0 | 0 | 0 | 0 |  |
| HAM/TSP#2 | 5582 | chr17_71990993_+ | 1 | 0 | 0 | 0 | 0 |  |
| HAM/TSP#2 | 5583 | chr17_72987222_+ | 3 | 0 | 0 | 0 | 0 |  |
| HAM/TSP#2 | 5584 | chr17_7361943_+ | 11 | 0 | 0 | 0 | 0 |  |
| HAM/TSP#2 | 5585 | chr17_74041325_- | 2 | 0 | 0 | 0 | 0 |  |
| HAM/TSP#2 | 5586 | chr17_74321848_- | 1 | 0 | 0 | 0 | 0 |  |
| HAM/TSP#2 | 5587 | chr17_74984223_- | 1 | 0 | 0 | 0 | 0 |  |
| HAM/TSP#2 | 5588 | chr17_75504541_+ | 0 | 0 | 0 | 1 | 0 |  |
| HAM/TSP#2 | 5589 | chr17_75521972_+ | 1 | 0 | 0 | 0 | 0 |  |
| HAM/TSP#2 | 5590 | chr17_76002236_- | 1 | 0 | 0 | 0 | 0 |  |
| HAM/TSP#2 | 5591 | chr17_76260173_- | 0 | 2 | 0 | 0 | 0 |  |
| HAM/TSP#2 | 5592 | chr17_76799275_- | 1 | 0 | 0 | 0 | 0 |  |
| HAM/TSP#2 | 5593 | chr17_7688713_+ | 1 | 0 | 0 | 0 | 0 |  |
| HAM/TSP#2 | 5594 | chr17_77490425_+ | 1 | 0 | 0 | 0 | 0 |  |
| HAM/TSP#2 | 5595 | chr17_78983325_- | 26 | 0 | 0 | 0 | 0 |  |
| HAM/TSP#2 | 5596 | chr17_79917062_+ | 1 | 0 | 0 | 0 | 0 |  |
| HAM/TSP#2 | 5597 | chr17_82863917_+ | 1 | 0 | 0 | 0 | 0 |  |
| HAM/TSP#2 | 5598 | chr17_83141478_- | 0 | 2 | 0 | 0 | 0 |  |
| HAM/TSP#2 | 5599 | chr17_8452633_- | 3 | 0 | 0 | 0 | 0 |  |
| HAM/TSP#2 | 5600 | chr17_8960827_- | 3 | 0 | 0 | 0 | 0 |  |
| HAM/TSP#2 | 5601 | chr17_9205807_+ | 0 | 1 | 0 | 0 | 0 |  |
| HAM/TSP#2 | 5602 | chr17_9570361_+ | 6 | 0 | 0 | 0 | 0 |  |
| HAM/TSP#2 | 5603 | chr18_11470356_+ | 2 | 0 | 0 | 0 | 0 |  |
| HAM/TSP#2 | 5604 | chr18_12167417_+ | 1 | 0 | 0 | 0 | 0 |  |
| HAM/TSP#2 | 5605 | chr18_1309864_+ | 2 | 0 | 0 | 0 | 0 |  |
| HAM/TSP#2 | 5606 | chr18_14685810_+ | 2 | 0 | 0 | 0 | 0 |  |
| HAM/TSP#2 | 5607 | chr18_22334050_- | 3 | 1 | 0 | 0 | 0 |  |
| HAM/TSP#2 | 5608 | chr18_22885933_+ | 1 | 0 | 0 | 0 | 0 |  |
| HAM/TSP#2 | 5609 | chr18_22974789_+ | 0 | 3 | 0 | 0 | 0 |  |
| HAM/TSP#2 | 5610 | chr18_23072576_- | 0 | 1 | 0 | 0 | 0 |  |
| HAM/TSP#2 | 5611 | chr18_24004668_- | 1 | 0 | 0 | 0 | 0 |  |
| HAM/TSP#2 | 5612 | chr18_24936671_- | 1 | 0 | 0 | 0 | 0 |  |
| HAM/TSP#2 | 5613 | chr18_25018176_+ | 1 | 0 | 0 | 0 | 0 |  |
| HAM/TSP#2 | 5614 | chr18_25440051_- | 0 | 1 | 0 | 0 | 0 |  |
| HAM/TSP#2 | 5615 | chr18_26419821_- | 1 | 0 | 0 | 0 | 0 |  |
| HAM/TSP#2 | 5616 | chr18_27191559_+ | 2 | 0 | 0 | 0 | 0 |  |
| HAM/TSP#2 | 5617 | chr18_27707178_- | 11 | 1 | 0 | 0 | 0 |  |
| HAM/TSP#2 | 5618 | chr18_29843889_- | 0 | 1 | 0 | 0 | 0 |  |
| HAM/TSP#2 | 5619 | chr18_29992009_+ | 1 | 0 | 0 | 0 | 0 |  |
| HAM/TSP#2 | 5620 | chr18_31579867_+ | 3 | 0 | 0 | 0 | 0 |  |
| HAM/TSP#2 | 5621 | chr18_3173085_- | 1 | 0 | 0 | 0 | 0 |  |
| HAM/TSP#2 | 5622 | chr18_32013506_+ | 15 | 1 | 0 | 0 | 0 |  |
| HAM/TSP#2 | 5623 | chr18_32124816_+ | 1 | 0 | 0 | 0 | 0 |  |
| HAM/TSP#2 | 5624 | chr18_3244468_+ | 2 | 0 | 0 | 0 | 0 |  |
| HAM/TSP#2 | 5625 | chr18_32551375_- | 2 | 0 | 0 | 0 | 0 |  |
| HAM/TSP#2 | 5626 | chr18_33137536_- | 1 | 0 | 0 | 0 | 0 |  |
| HAM/TSP#2 | 5627 | chr18_34076248_- | 1 | 0 | 0 | 0 | 0 |  |
| HAM/TSP#2 | 5628 | chr18_35266543_+ | 2 | 2 | 0 | 0 | 0 |  |
| HAM/TSP#2 | 5629 | chr18_36226820_+ | 1 | 0 | 0 | 0 | 0 |  |
| HAM/TSP#2 | 5630 | chr18_37473564_+ | 2 | 0 | 0 | 0 | 0 |  |
| HAM/TSP#2 | 5631 | chr18_37567213_- | 5 | 0 | 0 | 0 | 0 |  |
| HAM/TSP#2 | 5632 | chr18_38268264_+ | 1 | 0 | 0 | 0 | 0 |  |
| HAM/TSP#2 | 5633 | chr18_3914467_+ | 1 | 0 | 0 | 0 | 0 |  |
| HAM/TSP#2 | 5634 | chr18_3925601_+ | 1 | 0 | 0 | 0 | 0 |  |
| HAM/TSP#2 | 5635 | chr18_40020676_- | 0 | 1 | 0 | 0 | 0 |  |
| HAM/TSP#2 | 5636 | chr18_40412864_+ | 4 | 0 | 0 | 0 | 0 |  |
| HAM/TSP#2 | 5637 | chr18_41530267_+ | 1 | 0 | 0 | 0 | 0 |  |
| HAM/TSP#2 | 5638 | chr18_41841376_- | 0 | 2 | 0 | 0 | 1 |  |
| HAM/TSP#2 | 5639 | chr18_43900418_+ | 1 | 0 | 0 | 0 | 0 |  |
| HAM/TSP#2 | 5640 | chr18_45598249_- | 1 | 0 | 0 | 0 | 0 |  |
| HAM/TSP#2 | 5641 | chr18_45800747_+ | 1 | 0 | 0 | 0 | 0 |  |
| HAM/TSP#2 | 5642 | chr18_46058766_- | 1 | 0 | 0 | 0 | 0 |  |
| HAM/TSP#2 | 5643 | chr18_4615250_- | 1 | 0 | 0 | 0 | 0 |  |
| HAM/TSP#2 | 5644 | chr18_48816754_- | 2 | 0 | 0 | 0 | 0 |  |
| HAM/TSP#2 | 5645 | chr18_48974081_+ | 2 | 0 | 0 | 0 | 0 |  |
| HAM/TSP#2 | 5646 | chr18_49186762_- | 0 | 1 | 0 | 0 | 0 |  |
| HAM/TSP#2 | 5647 | chr18_49795589_- | 2 | 0 | 0 | 0 | 0 |  |
| HAM/TSP#2 | 5648 | chr18_50908002_+ | 2 | 0 | 0 | 0 | 0 |  |
| HAM/TSP#2 | 5649 | chr18_51067559_+ | 0 | 1 | 0 | 0 | 0 |  |
| HAM/TSP#2 | 5650 | chr18_51579173_+ | 2 | 0 | 0 | 0 | 0 |  |
| HAM/TSP#2 | 5651 | chr18_51935450_+ | 2 | 0 | 0 | 0 | 0 |  |
| HAM/TSP#2 | 5652 | chr18_52877675_- | 1 | 0 | 0 | 0 | 0 |  |
| HAM/TSP#2 | 5653 | chr18_54021714_+ | 3 | 0 | 0 | 0 | 0 |  |
| HAM/TSP#2 | 5654 | chr18_55175236_- | 0 | 1 | 0 | 0 | 0 |  |
| HAM/TSP#2 | 5655 | chr18_55299535_- | 1 | 0 | 0 | 0 | 0 |  |
| HAM/TSP#2 | 5656 | chr18_55500001_- | 0 | 0 | 0 | 0 | 1 |  |
| HAM/TSP#2 | 5657 | chr18_56110748_+ | 6 | 0 | 0 | 0 | 0 |  |
| HAM/TSP#2 | 5658 | chr18_56751229_- | 0 | 0 | 0 | 0 | 1 |  |
| HAM/TSP#2 | 5659 | chr18_56840679_+ | 9 | 1 | 0 | 0 | 0 |  |
| HAM/TSP#2 | 5660 | chr18_57019333_- | 1 | 0 | 0 | 0 | 0 |  |
| HAM/TSP#2 | 5661 | chr18_57047758_- | 1 | 0 | 0 | 0 | 0 |  |
| HAM/TSP#2 | 5662 | chr18_5716939_- | 3 | 0 | 0 | 0 | 0 |  |
| HAM/TSP#2 | 5663 | chr18_57386089_- | 2 | 0 | 0 | 0 | 0 |  |
| HAM/TSP#2 | 5664 | chr18_57575840_- | 0 | 3 | 0 | 0 | 0 |  |
| HAM/TSP#2 | 5665 | chr18_5896762_+ | 1 | 0 | 0 | 0 | 0 |  |
| HAM/TSP#2 | 5666 | chr18_59201916_- | 1 | 0 | 0 | 0 | 0 |  |
| HAM/TSP#2 | 5667 | chr18_59532334_+ | 1 | 0 | 0 | 0 | 0 |  |
| HAM/TSP#2 | 5668 | chr18_5985907_+ | 1 | 0 | 0 | 0 | 0 |  |
| HAM/TSP#2 | 5669 | chr18_60185553_+ | 1 | 0 | 0 | 0 | 0 |  |
| HAM/TSP#2 | 5670 | chr18_62242549_- | 1 | 0 | 0 | 0 | 0 |  |
| HAM/TSP#2 | 5671 | chr18_63368910_+ | 1 | 0 | 0 | 0 | 0 |  |
| HAM/TSP#2 | 5672 | chr18_6487442_+ | 0 | 0 | 2 | 0 | 0 |  |
| HAM/TSP#2 | 5673 | chr18_65985339_+ | 0 | 14 | 0 | 0 | 0 |  |
| HAM/TSP#2 | 5674 | chr18_67212273_+ | 0 | 0 | 0 | 0 | 1 |  |
| HAM/TSP#2 | 5675 | chr18_67658516_- | 0 | 17 | 0 | 0 | 0 |  |
| HAM/TSP#2 | 5676 | chr18_69086989_+ | 1 | 0 | 0 | 0 | 0 |  |
| HAM/TSP#2 | 5677 | chr18_70019451_+ | 4 | 0 | 0 | 0 | 0 |  |
| HAM/TSP#2 | 5678 | chr18_71357598_- | 4 | 0 | 0 | 0 | 0 |  |
| HAM/TSP#2 | 5679 | chr18_7149862_+ | 0 | 1 | 0 | 0 | 0 |  |
| HAM/TSP#2 | 5680 | chr18_71810895_- | 1 | 0 | 0 | 0 | 0 |  |
| HAM/TSP#2 | 5681 | chr18_73868396_- | 1 | 0 | 0 | 0 | 0 |  |
| HAM/TSP#2 | 5682 | chr18_74571115_+ | 2 | 0 | 0 | 0 | 0 |  |
| HAM/TSP#2 | 5683 | chr18_75022809_+ | 3 | 0 | 0 | 0 | 0 |  |
| HAM/TSP#2 | 5684 | chr18_76411899_- | 1 | 0 | 0 | 0 | 0 |  |
| HAM/TSP#2 | 5685 | chr18_78131238_- | 1 | 0 | 0 | 0 | 0 |  |
| HAM/TSP#2 | 5686 | chr18_78809351_+ | 5 | 0 | 0 | 0 | 9 |  |
| HAM/TSP#2 | 5687 | chr18_79056668_- | 1 | 0 | 0 | 0 | 0 |  |
| HAM/TSP#2 | 5688 | chr18_79399211_- | 1 | 0 | 0 | 0 | 0 |  |
| HAM/TSP#2 | 5689 | chr18_8117553_+ | 1 | 0 | 0 | 0 | 0 |  |
| HAM/TSP#2 | 5690 | chr18_8370283_+ | 2 | 0 | 0 | 0 | 0 |  |
| HAM/TSP#2 | 5691 | chr18_8754665_+ | 3 | 0 | 0 | 0 | 0 |  |
| HAM/TSP#2 | 5692 | chr19_1071052_- | 1 | 0 | 0 | 0 | 0 |  |
| HAM/TSP#2 | 5693 | chr19_10879604_- | 3 | 0 | 0 | 0 | 0 |  |
| HAM/TSP#2 | 5694 | chr19_11419458_+ | 0 | 0 | 0 | 0 | 1 |  |
| HAM/TSP#2 | 5695 | chr19_11764818_+ | 1 | 0 | 0 | 0 | 0 |  |
| HAM/TSP#2 | 5696 | chr19_11812277_+ | 1 | 0 | 0 | 0 | 0 |  |
| HAM/TSP#2 | 5697 | chr19_12002657_- | 1 | 0 | 0 | 0 | 0 |  |
| HAM/TSP#2 | 5698 | chr19_12456227_+ | 1 | 0 | 0 | 0 | 0 |  |
| HAM/TSP#2 | 5699 | chr19_13008900_- | 1 | 0 | 0 | 0 | 0 |  |
| HAM/TSP#2 | 5700 | chr19_16889760_- | 1 | 0 | 0 | 0 | 0 |  |
| HAM/TSP#2 | 5701 | chr19_18369414_- | 1 | 0 | 0 | 0 | 0 |  |
| HAM/TSP#2 | 5702 | chr19_18432074_+ | 1 | 0 | 0 | 0 | 0 |  |
| HAM/TSP#2 | 5703 | chr19_18928112_+ | 1 | 0 | 0 | 0 | 0 |  |
| HAM/TSP#2 | 5704 | chr19_19873951_+ | 1 | 0 | 0 | 0 | 0 |  |
| HAM/TSP#2 | 5705 | chr19_20797908_+ | 1 | 0 | 0 | 0 | 0 |  |
| HAM/TSP#2 | 5706 | chr19_20820687_- | 1 | 0 | 0 | 0 | 0 |  |
| HAM/TSP#2 | 5707 | chr19_21755705_- | 5 | 0 | 0 | 0 | 0 |  |
| HAM/TSP#2 | 5708 | chr19_22311679_- | 0 | 0 | 1 | 0 | 0 |  |
| HAM/TSP#2 | 5709 | chr19_24030264_- | 1 | 0 | 0 | 0 | 0 |  |
| HAM/TSP#2 | 5710 | chr19_24895769_- | 1 | 0 | 0 | 0 | 0 |  |
| HAM/TSP#2 | 5711 | chr19_2635220_- | 0 | 0 | 0 | 1 | 0 |  |
| HAM/TSP#2 | 5712 | chr19_27422830_+ | 1 | 0 | 0 | 0 | 0 |  |
| HAM/TSP#2 | 5713 | chr19_27510158_- | 1 | 0 | 0 | 0 | 0 |  |
| HAM/TSP#2 | 5714 | chr19_27682937_- | 4 | 0 | 0 | 0 | 0 |  |
| HAM/TSP#2 | 5715 | chr19_28633290_- | 5 | 0 | 0 | 0 | 0 |  |
| HAM/TSP#2 | 5716 | chr19_29012095_- | 1 | 0 | 0 | 0 | 0 |  |
| HAM/TSP#2 | 5717 | chr19_29029794_+ | 0 | 3 | 0 | 0 | 0 |  |
| HAM/TSP#2 | 5718 | chr19_29142127_- | 1 | 0 | 0 | 0 | 0 |  |
| HAM/TSP#2 | 5719 | chr19_29416343_- | 1 | 0 | 0 | 0 | 0 |  |
| HAM/TSP#2 | 5720 | chr19_29429315_+ | 0 | 0 | 0 | 1 | 0 |  |
| HAM/TSP#2 | 5721 | chr19_30167415_- | 1 | 0 | 0 | 0 | 0 |  |
| HAM/TSP#2 | 5722 | chr19_30833221_+ | 1 | 0 | 0 | 0 | 0 |  |
| HAM/TSP#2 | 5723 | chr19_34138890_+ | 1 | 0 | 0 | 0 | 0 |  |
| HAM/TSP#2 | 5724 | chr19_34270966_- | 1 | 0 | 0 | 0 | 0 |  |
| HAM/TSP#2 | 5725 | chr19_34339107_+ | 1 | 0 | 0 | 0 | 0 |  |
| HAM/TSP#2 | 5726 | chr19_34554771_+ | 1 | 0 | 0 | 0 | 0 |  |
| HAM/TSP#2 | 5727 | chr19_36665867_- | 1 | 0 | 0 | 0 | 0 |  |
| HAM/TSP#2 | 5728 | chr19_37364611_- | 1 | 0 | 0 | 0 | 0 |  |
| HAM/TSP#2 | 5729 | chr19_37929106_- | 0 | 1 | 0 | 0 | 0 |  |
| HAM/TSP#2 | 5730 | chr19_38356654_+ | 1 | 0 | 0 | 0 | 0 |  |
| HAM/TSP#2 | 5731 | chr19_39952938_+ | 1 | 0 | 0 | 0 | 0 |  |
| HAM/TSP#2 | 5732 | chr19_39991110_+ | 0 | 0 | 0 | 0 | 1 |  |
| HAM/TSP#2 | 5733 | chr19_41406915_+ | 7 | 0 | 0 | 0 | 0 |  |
| HAM/TSP#2 | 5734 | chr19_42204307_- | 1 | 0 | 0 | 0 | 0 |  |
| HAM/TSP#2 | 5735 | chr19_43411137_+ | 1 | 0 | 0 | 0 | 0 |  |
| HAM/TSP#2 | 5736 | chr19_44355897_- | 1 | 0 | 0 | 0 | 0 |  |
| HAM/TSP#2 | 5737 | chr19_44443086_- | 1 | 0 | 0 | 0 | 0 |  |
| HAM/TSP#2 | 5738 | chr19_45040719_+ | 1 | 0 | 0 | 0 | 0 |  |
| HAM/TSP#2 | 5739 | chr19_48246213_+ | 1 | 0 | 0 | 0 | 0 |  |
| HAM/TSP#2 | 5740 | chr19_49015082_+ | 1 | 0 | 0 | 0 | 0 |  |
| HAM/TSP#2 | 5741 | chr19_49524625_- | 1 | 0 | 0 | 0 | 0 |  |
| HAM/TSP#2 | 5742 | chr19_50296816_- | 1 | 0 | 0 | 0 | 0 |  |
| HAM/TSP#2 | 5743 | chr19_50337756_- | 3 | 0 | 0 | 0 | 0 |  |
| HAM/TSP#2 | 5744 | chr19_55883137_- | 1 | 0 | 0 | 0 | 0 |  |
| HAM/TSP#2 | 5745 | chr19_56314824_- | 1 | 0 | 0 | 0 | 0 |  |
| HAM/TSP#2 | 5746 | chr19_56903744_- | 2 | 0 | 0 | 0 | 0 |  |
| HAM/TSP#2 | 5747 | chr19_58130054_+ | 1 | 0 | 0 | 0 | 0 |  |
| HAM/TSP#2 | 5748 | chr19_7701860_- | 1 | 0 | 0 | 0 | 0 |  |
| HAM/TSP#2 | 5749 | chr19_8090971_+ | 1 | 0 | 0 | 0 | 0 |  |
| HAM/TSP#2 | 5750 | chr19_8805034_+ | 1 | 0 | 0 | 0 | 0 |  |
| HAM/TSP#2 | 5751 | chr19_893391_+ | 2 | 0 | 0 | 0 | 0 |  |
| HAM/TSP#2 | 5752 | chr19_9906598_- | 1 | 0 | 0 | 0 | 0 |  |
| HAM/TSP#2 | 5753 | chr20_10330516_- | 1 | 0 | 0 | 0 | 0 |  |
| HAM/TSP#2 | 5754 | chr20_12243930_+ | 5 | 0 | 0 | 0 | 0 |  |
| HAM/TSP#2 | 5755 | chr20_12953237_+ | 1 | 0 | 0 | 0 | 0 |  |
| HAM/TSP#2 | 5756 | chr20_13231378_+ | 1 | 0 | 0 | 0 | 0 |  |
| HAM/TSP#2 | 5757 | chr20_13265317_- | 7 | 0 | 0 | 0 | 0 |  |
| HAM/TSP#2 | 5758 | chr20_13337881_+ | 0 | 6 | 1 | 0 | 0 |  |
| HAM/TSP#2 | 5759 | chr20_13699301_+ | 1 | 0 | 0 | 0 | 0 |  |
| HAM/TSP#2 | 5760 | chr20_14058562_+ | 0 | 1 | 0 | 0 | 0 |  |
| HAM/TSP#2 | 5761 | chr20_14106645_+ | 5 | 0 | 0 | 0 | 0 |  |
| HAM/TSP#2 | 5762 | chr20_1460894_+ | 1 | 0 | 0 | 0 | 0 |  |
| HAM/TSP#2 | 5763 | chr20_15733191_+ | 7 | 0 | 0 | 0 | 0 |  |
| HAM/TSP#2 | 5764 | chr20_16129238_+ | 2 | 0 | 0 | 0 | 0 |  |
| HAM/TSP#2 | 5765 | chr20_16377119_- | 0 | 10 | 0 | 0 | 0 |  |
| HAM/TSP#2 | 5766 | chr20_1702373_- | 1 | 0 | 0 | 0 | 0 |  |
| HAM/TSP#2 | 5767 | chr20_17147989_+ | 1 | 0 | 0 | 0 | 0 |  |
| HAM/TSP#2 | 5768 | chr20_19932344_+ | 1 | 0 | 0 | 0 | 0 |  |
| HAM/TSP#2 | 5769 | chr20_20089778_+ | 1 | 0 | 0 | 0 | 0 |  |
| HAM/TSP#2 | 5770 | chr20_20438489_- | 5 | 0 | 0 | 0 | 0 |  |
| HAM/TSP#2 | 5771 | chr20_21705176_- | 1 | 0 | 0 | 0 | 0 |  |
| HAM/TSP#2 | 5772 | chr20_22008370_+ | 1 | 0 | 0 | 0 | 0 |  |
| HAM/TSP#2 | 5773 | chr20_23037318_+ | 2 | 0 | 0 | 0 | 0 |  |
| HAM/TSP#2 | 5774 | chr20_23391239_- | 1 | 0 | 0 | 0 | 0 |  |
| HAM/TSP#2 | 5775 | chr20_23859606_+ | 5 | 0 | 0 | 0 | 0 |  |
| HAM/TSP#2 | 5776 | chr20_25374618_- | 1 | 0 | 0 | 0 | 0 |  |
| HAM/TSP#2 | 5777 | chr20_25748539_+ | 1 | 0 | 0 | 0 | 0 |  |
| HAM/TSP#2 | 5778 | chr20_2724442_+ | 3 | 0 | 0 | 0 | 0 |  |
| HAM/TSP#2 | 5779 | chr20_28513645_+ | 8 | 1 | 0 | 0 | 0 |  |
| HAM/TSP#2 | 5780 | chr20_30330628_- | 5 | 0 | 0 | 0 | 0 |  |
| HAM/TSP#2 | 5781 | chr20_30346060_- | 0 | 1 | 0 | 0 | 0 |  |
| HAM/TSP#2 | 5782 | chr20_30494741_+ | 1 | 0 | 0 | 0 | 0 |  |
| HAM/TSP#2 | 5783 | chr20_30724968_+ | 1 | 0 | 0 | 0 | 0 |  |
| HAM/TSP#2 | 5784 | chr20_31058586_+ | 0 | 0 | 0 | 0 | 1 |  |
| HAM/TSP#2 | 5785 | chr20_3210519_+ | 1 | 0 | 0 | 0 | 0 |  |
| HAM/TSP#2 | 5786 | chr20_32738119_- | 10 | 0 | 0 | 0 | 5 |  |
| HAM/TSP#2 | 5787 | chr20_32952680_+ | 22 | 0 | 0 | 0 | 0 |  |
| HAM/TSP#2 | 5788 | chr20_33311431_+ | 5 | 1 | 0 | 0 | 0 |  |
| HAM/TSP#2 | 5789 | chr20_33337629_+ | 2 | 0 | 0 | 0 | 0 |  |
| HAM/TSP#2 | 5790 | chr20_33750659_+ | 1 | 0 | 0 | 0 | 0 |  |
| HAM/TSP#2 | 5791 | chr20_34552465_+ | 1 | 0 | 0 | 0 | 0 |  |
| HAM/TSP#2 | 5792 | chr20_34919791_- | 1 | 0 | 0 | 0 | 0 |  |
| HAM/TSP#2 | 5793 | chr20_35565004_+ | 1 | 0 | 0 | 0 | 0 |  |
| HAM/TSP#2 | 5794 | chr20_37340673_+ | 2 | 0 | 0 | 0 | 0 |  |
| HAM/TSP#2 | 5795 | chr20_37518249_- | 0 | 1 | 0 | 0 | 0 |  |
| HAM/TSP#2 | 5796 | chr20_38433016_- | 1 | 0 | 0 | 0 | 0 |  |
| HAM/TSP#2 | 5797 | chr20_39865649_- | 1 | 0 | 0 | 0 | 0 |  |
| HAM/TSP#2 | 5798 | chr20_40533419_+ | 2 | 0 | 0 | 0 | 0 |  |
| HAM/TSP#2 | 5799 | chr20_40760897_- | 1 | 0 | 0 | 0 | 0 |  |
| HAM/TSP#2 | 5800 | chr20_41163998_- | 3 | 0 | 0 | 0 | 0 |  |
| HAM/TSP#2 | 5801 | chr20_43223329_- | 6 | 0 | 0 | 0 | 0 |  |
| HAM/TSP#2 | 5802 | chr20_43802306_- | 1 | 0 | 0 | 0 | 0 |  |
| HAM/TSP#2 | 5803 | chr20_44143622_- | 1 | 0 | 0 | 0 | 0 |  |
| HAM/TSP#2 | 5804 | chr20_44750724_- | 1 | 0 | 0 | 0 | 0 |  |
| HAM/TSP#2 | 5805 | chr20_47492668_+ | 0 | 1 | 0 | 0 | 0 |  |
| HAM/TSP#2 | 5806 | chr20_48600127_- | 0 | 0 | 0 | 0 | 1 |  |
| HAM/TSP#2 | 5807 | chr20_48708879_- | 1 | 0 | 0 | 0 | 0 |  |
| HAM/TSP#2 | 5808 | chr20_49542804_- | 1 | 0 | 0 | 0 | 0 |  |
| HAM/TSP#2 | 5809 | chr20_50247730_- | 1 | 0 | 0 | 0 | 0 |  |
| HAM/TSP#2 | 5810 | chr20_50387538_+ | 2 | 0 | 0 | 0 | 0 |  |
| HAM/TSP#2 | 5811 | chr20_50803967_+ | 1 | 0 | 0 | 0 | 0 |  |
| HAM/TSP#2 | 5812 | chr20_52469058_- | 2 | 0 | 0 | 0 | 0 |  |
| HAM/TSP#2 | 5813 | chr20_54825331_- | 2 | 0 | 0 | 0 | 0 |  |
| HAM/TSP#2 | 5814 | chr20_56418741_+ | 4 | 0 | 0 | 0 | 0 |  |
| HAM/TSP#2 | 5815 | chr20_56513661_- | 1 | 0 | 0 | 0 | 0 |  |
| HAM/TSP#2 | 5816 | chr20_56632511_+ | 6 | 0 | 0 | 0 | 0 |  |
| HAM/TSP#2 | 5817 | chr20_56960057_- | 1 | 0 | 0 | 0 | 0 |  |
| HAM/TSP#2 | 5818 | chr20_57578249_+ | 0 | 73 | 1 | 0 | 0 |  |
| HAM/TSP#2 | 5819 | chr20_57578342_+ | 0 | 1 | 0 | 0 | 0 |  |
| HAM/TSP#2 | 5820 | chr20_6052189_- | 1 | 0 | 0 | 0 | 0 |  |
| HAM/TSP#2 | 5821 | chr20_61129409_- | 1 | 0 | 0 | 0 | 0 |  |
| HAM/TSP#2 | 5822 | chr20_61544563_+ | 1 | 0 | 0 | 0 | 0 |  |
| HAM/TSP#2 | 5823 | chr20_61932730_+ | 1 | 0 | 0 | 0 | 0 |  |
| HAM/TSP#2 | 5824 | chr20_62351549_+ | 1 | 0 | 0 | 0 | 0 |  |
| HAM/TSP#2 | 5825 | chr20_62610442_- | 1 | 0 | 0 | 0 | 0 |  |
| HAM/TSP#2 | 5826 | chr20_62714131_+ | 1 | 0 | 0 | 0 | 0 |  |
| HAM/TSP#2 | 5827 | chr20_6315187_- | 4 | 0 | 0 | 0 | 0 |  |
| HAM/TSP#2 | 5828 | chr20_63285840_- | 1 | 0 | 0 | 0 | 0 |  |
| HAM/TSP#2 | 5829 | chr20_63409036_+ | 1 | 0 | 0 | 0 | 0 |  |
| HAM/TSP#2 | 5830 | chr20_7146255_- | 2 | 0 | 0 | 0 | 0 |  |
| HAM/TSP#2 | 5831 | chr20_7379256_- | 39 | 1 | 0 | 0 | 3 |  |
| HAM/TSP#2 | 5832 | chr20_9849525_- | 11 | 0 | 0 | 0 | 0 |  |
| HAM/TSP#2 | 5833 | chr20_987643_+ | 1 | 0 | 0 | 0 | 0 |  |
| HAM/TSP#2 | 5834 | chr21_10356837_- | 1 | 0 | 0 | 0 | 0 |  |
| HAM/TSP#2 | 5835 | chr21_10414652_+ | 1 | 0 | 0 | 0 | 0 |  |
| HAM/TSP#2 | 5836 | chr21_13170802_- | 1 | 0 | 0 | 0 | 0 |  |
| HAM/TSP#2 | 5837 | chr21_14280545_+ | 1 | 0 | 0 | 0 | 0 |  |
| HAM/TSP#2 | 5838 | chr21_14495474_- | 2 | 0 | 0 | 0 | 0 |  |
| HAM/TSP#2 | 5839 | chr21_14703414_- | 1 | 0 | 0 | 0 | 0 |  |
| HAM/TSP#2 | 5840 | chr21_15975281_- | 1 | 0 | 0 | 0 | 0 |  |
| HAM/TSP#2 | 5841 | chr21_16468659_+ | 1 | 0 | 0 | 0 | 0 |  |
| HAM/TSP#2 | 5842 | chr21_17028980_- | 2 | 0 | 0 | 0 | 0 |  |
| HAM/TSP#2 | 5843 | chr21_17491125_+ | 1 | 0 | 0 | 0 | 0 |  |
| HAM/TSP#2 | 5844 | chr21_18074297_- | 2 | 0 | 0 | 0 | 0 |  |
| HAM/TSP#2 | 5845 | chr21_18207716_+ | 1 | 0 | 0 | 0 | 0 |  |
| HAM/TSP#2 | 5846 | chr21_19195077_- | 1 | 0 | 0 | 0 | 0 |  |
| HAM/TSP#2 | 5847 | chr21_19829537_+ | 1 | 0 | 0 | 0 | 0 |  |
| HAM/TSP#2 | 5848 | chr21_21884235_+ | 8 | 0 | 0 | 0 | 0 |  |
| HAM/TSP#2 | 5849 | chr21_22649750_+ | 0 | 2 | 0 | 0 | 0 |  |
| HAM/TSP#2 | 5850 | chr21_23256491_+ | 1 | 0 | 0 | 0 | 0 |  |
| HAM/TSP#2 | 5851 | chr21_23728598_+ | 3 | 0 | 0 | 0 | 0 |  |
| HAM/TSP#2 | 5852 | chr21_23841471_- | 3 | 0 | 0 | 0 | 0 |  |
| HAM/TSP#2 | 5853 | chr21_25274685_- | 1 | 0 | 0 | 0 | 0 |  |
| HAM/TSP#2 | 5854 | chr21_25786493_- | 3 | 0 | 0 | 0 | 0 |  |
| HAM/TSP#2 | 5855 | chr21_26086247_+ | 1 | 0 | 0 | 0 | 0 |  |
| HAM/TSP#2 | 5856 | chr21_29173534_- | 1 | 0 | 0 | 0 | 0 |  |
| HAM/TSP#2 | 5857 | chr21_29426262_- | 2 | 0 | 0 | 0 | 0 |  |
| HAM/TSP#2 | 5858 | chr21_30691966_+ | 1 | 0 | 0 | 0 | 0 |  |
| HAM/TSP#2 | 5859 | chr21_32142401_- | 0 | 1 | 0 | 0 | 0 |  |
| HAM/TSP#2 | 5860 | chr21_32623219_+ | 1 | 0 | 0 | 0 | 0 |  |
| HAM/TSP#2 | 5861 | chr21_33058066_- | 1 | 0 | 0 | 0 | 0 |  |
| HAM/TSP#2 | 5862 | chr21_33125106_+ | 0 | 1 | 0 | 0 | 0 |  |
| HAM/TSP#2 | 5863 | chr21_33853598_+ | 0 | 0 | 0 | 1 | 0 |  |
| HAM/TSP#2 | 5864 | chr21_34189910_- | 1 | 0 | 0 | 0 | 0 |  |
| HAM/TSP#2 | 5865 | chr21_34210020_- | 6 | 0 | 0 | 0 | 0 |  |
| HAM/TSP#2 | 5866 | chr21_35777522_- | 3 | 0 | 0 | 0 | 0 |  |
| HAM/TSP#2 | 5867 | chr21_36068019_- | 2 | 0 | 0 | 0 | 0 |  |
| HAM/TSP#2 | 5868 | chr21_37142117_+ | 1 | 0 | 0 | 0 | 0 |  |
| HAM/TSP#2 | 5869 | chr21_37871121_+ | 1 | 0 | 0 | 0 | 0 |  |
| HAM/TSP#2 | 5870 | chr21_38001743_- | 1 | 0 | 0 | 0 | 0 |  |
| HAM/TSP#2 | 5871 | chr21_38101200_- | 5 | 0 | 0 | 0 | 0 |  |
| HAM/TSP#2 | 5872 | chr21_38635838_- | 2 | 0 | 0 | 0 | 0 |  |
| HAM/TSP#2 | 5873 | chr21_39253374_- | 1 | 0 | 0 | 0 | 0 |  |
| HAM/TSP#2 | 5874 | chr21_40757250_+ | 1 | 0 | 0 | 0 | 0 |  |
| HAM/TSP#2 | 5875 | chr21_41449690_- | 2 | 0 | 0 | 0 | 0 |  |
| HAM/TSP#2 | 5876 | chr21_42000961_- | 1 | 0 | 0 | 0 | 0 |  |
| HAM/TSP#2 | 5877 | chr21_42051192_+ | 2 | 0 | 0 | 0 | 0 |  |
| HAM/TSP#2 | 5878 | chr21_42437909_+ | 10 | 0 | 0 | 0 | 0 |  |
| HAM/TSP#2 | 5879 | chr21_43163408_+ | 2 | 0 | 0 | 0 | 0 |  |
| HAM/TSP#2 | 5880 | chr21_44033733_+ | 1 | 0 | 0 | 0 | 0 |  |
| HAM/TSP#2 | 5881 | chr21_44948322_- | 5 | 0 | 0 | 0 | 0 |  |
| HAM/TSP#2 | 5882 | chr21_45473646_+ | 2 | 0 | 0 | 0 | 0 |  |
| HAM/TSP#2 | 5883 | chr21_46270482_+ | 2 | 0 | 0 | 0 | 0 |  |
| HAM/TSP#2 | 5884 | chr21_6375352_- | 1 | 0 | 0 | 0 | 0 |  |
| HAM/TSP#2 | 5885 | chr21_7924106_- | 11 | 0 | 0 | 0 | 0 |  |
| HAM/TSP#2 | 5886 | chr21_7926401_- | 1 | 0 | 0 | 0 | 0 |  |
| HAM/TSP#2 | 5887 | chr21_7929625_+ | 2 | 1 | 0 | 0 | 0 |  |
| HAM/TSP#2 | 5888 | chr21_8234560_- | 0 | 0 | 0 | 1 | 0 |  |
| HAM/TSP#2 | 5889 | chr21_9008643_+ | 3 | 0 | 0 | 0 | 0 |  |
| HAM/TSP#2 | 5890 | chr21_9081493_- | 3 | 0 | 0 | 0 | 0 |  |
| HAM/TSP#2 | 5891 | chr21_9359272_+ | 0 | 119 | 1 | 2 | 0 |  |
| HAM/TSP#2 | 5892 | chr22_10687483_- | 2 | 0 | 0 | 0 | 0 |  |
| HAM/TSP#2 | 5893 | chr22_11452616_+ | 0 | 0 | 1 | 0 | 0 |  |
| HAM/TSP#2 | 5894 | chr22_11929996_- | 2 | 0 | 0 | 0 | 0 |  |
| HAM/TSP#2 | 5895 | chr22_12781464_- | 0 | 0 | 5 | 0 | 0 |  |
| HAM/TSP#2 | 5896 | chr22_16016114_- | 1 | 0 | 0 | 0 | 0 |  |
| HAM/TSP#2 | 5897 | chr22_16019347_- | 0 | 12 | 0 | 1 | 0 |  |
| HAM/TSP#2 | 5898 | chr22_16554393_+ | 3 | 0 | 0 | 0 | 0 |  |
| HAM/TSP#2 | 5899 | chr22_16767926_+ | 3 | 0 | 0 | 0 | 0 |  |
| HAM/TSP#2 | 5900 | chr22_16960547_+ | 2 | 1 | 0 | 0 | 0 |  |
| HAM/TSP#2 | 5901 | chr22_16997152_+ | 0 | 12 | 0 | 0 | 0 |  |
| HAM/TSP#2 | 5902 | chr22_17172935_+ | 1 | 0 | 0 | 0 | 0 |  |
| HAM/TSP#2 | 5903 | chr22_17499568_+ | 4 | 0 | 0 | 0 | 0 |  |
| HAM/TSP#2 | 5904 | chr22_19287028_- | 8 | 0 | 0 | 0 | 0 |  |
| HAM/TSP#2 | 5905 | chr22_19418903_- | 2 | 0 | 0 | 0 | 0 |  |
| HAM/TSP#2 | 5906 | chr22_20449461_+ | 1 | 0 | 0 | 0 | 0 |  |
| HAM/TSP#2 | 5907 | chr22_22072490_+ | 3 | 0 | 0 | 0 | 0 |  |
| HAM/TSP#2 | 5908 | chr22_22729355_+ | 1 | 0 | 0 | 0 | 0 |  |
| HAM/TSP#2 | 5909 | chr22_23381984_+ | 1 | 0 | 0 | 0 | 0 |  |
| HAM/TSP#2 | 5910 | chr22_24146479_+ | 1 | 0 | 0 | 0 | 0 |  |
| HAM/TSP#2 | 5911 | chr22_24630348_+ | 0 | 1 | 0 | 0 | 0 |  |
| HAM/TSP#2 | 5912 | chr22_24788196_- | 1 | 0 | 0 | 0 | 0 |  |
| HAM/TSP#2 | 5913 | chr22_25625464_+ | 3 | 0 | 0 | 0 | 0 |  |
| HAM/TSP#2 | 5914 | chr22_26098457_+ | 4 | 0 | 0 | 0 | 0 |  |
| HAM/TSP#2 | 5915 | chr22_26916975_+ | 1 | 0 | 0 | 0 | 0 |  |
| HAM/TSP#2 | 5916 | chr22_27404020_- | 9 | 0 | 0 | 0 | 0 |  |
| HAM/TSP#2 | 5917 | chr22_27677930_- | 0 | 25 | 0 | 0 | 0 |  |
| HAM/TSP#2 | 5918 | chr22_27813374_+ | 1 | 0 | 0 | 0 | 0 |  |
| HAM/TSP#2 | 5919 | chr22_28114902_- | 1 | 0 | 0 | 0 | 0 |  |
| HAM/TSP#2 | 5920 | chr22_28896666_+ | 5 | 0 | 0 | 0 | 0 |  |
| HAM/TSP#2 | 5921 | chr22_29606771_- | 1 | 0 | 0 | 0 | 0 |  |
| HAM/TSP#2 | 5922 | chr22_30324060_- | 1 | 0 | 0 | 0 | 0 |  |
| HAM/TSP#2 | 5923 | chr22_31959516_- | 0 | 20 | 0 | 0 | 0 |  |
| HAM/TSP#2 | 5924 | chr22_31974938_+ | 1 | 0 | 0 | 0 | 0 |  |
| HAM/TSP#2 | 5925 | chr22_34362710_- | 1 | 0 | 0 | 0 | 0 |  |
| HAM/TSP#2 | 5926 | chr22_34477642_+ | 0 | 1 | 0 | 0 | 0 |  |
| HAM/TSP#2 | 5927 | chr22_35458661_- | 2 | 0 | 0 | 0 | 0 |  |
| HAM/TSP#2 | 5928 | chr22_37318556_+ | 0 | 1 | 0 | 0 | 0 |  |
| HAM/TSP#2 | 5929 | chr22_39678519_+ | 0 | 0 | 0 | 0 | 1 |  |
| HAM/TSP#2 | 5930 | chr22_41446704_- | 1 | 0 | 0 | 0 | 0 |  |
| HAM/TSP#2 | 5931 | chr22_41946431_- | 0 | 0 | 0 | 1 | 0 |  |
| HAM/TSP#2 | 5932 | chr22_42215714_+ | 1 | 0 | 0 | 0 | 0 |  |
| HAM/TSP#2 | 5933 | chr22_43000725_+ | 1 | 0 | 0 | 0 | 0 |  |
| HAM/TSP#2 | 5934 | chr22_43122746_+ | 6 | 0 | 0 | 0 | 0 |  |
| HAM/TSP#2 | 5935 | chr22_43645607_+ | 1 | 0 | 0 | 0 | 0 |  |
| HAM/TSP#2 | 5936 | chr22_44326742_- | 1 | 0 | 0 | 0 | 0 |  |
| HAM/TSP#2 | 5937 | chr22_45023970_- | 1 | 0 | 0 | 0 | 0 |  |
| HAM/TSP#2 | 5938 | chr22_46119400_+ | 1 | 0 | 0 | 0 | 0 |  |
| HAM/TSP#2 | 5939 | chr22_46469315_- | 1 | 0 | 0 | 0 | 0 |  |
| HAM/TSP#2 | 5940 | chr22_46792389_+ | 2 | 0 | 0 | 0 | 0 |  |
| HAM/TSP#2 | 5941 | chr22_47026571_+ | 1 | 0 | 0 | 0 | 0 |  |
| HAM/TSP#2 | 5942 | chr22_47146018_+ | 12 | 0 | 0 | 0 | 0 |  |
| HAM/TSP#2 | 5943 | chr22_47555325_+ | 17 | 0 | 0 | 0 | 0 |  |
| HAM/TSP#2 | 5944 | chr22_47871460_- | 2 | 0 | 0 | 0 | 0 |  |
| HAM/TSP#2 | 5945 | chr22_47914163_- | 0 | 3 | 0 | 0 | 0 |  |
| HAM/TSP#2 | 5946 | chr22_47971985_- | 6 | 0 | 0 | 0 | 0 |  |
| HAM/TSP#2 | 5947 | chr22_48140879_- | 1 | 0 | 0 | 0 | 0 |  |
| HAM/TSP#2 | 5948 | chr22_48451787_+ | 2 | 0 | 0 | 0 | 0 |  |
| HAM/TSP#2 | 5949 | chr22_48728262_- | 0 | 3 | 0 | 0 | 0 |  |
| HAM/TSP#2 | 5950 | chr22_50262978_- | 4 | 0 | 0 | 0 | 0 |  |
| HAM/TSP#2 | 5951 | chrX_100233963_- | 6 | 0 | 0 | 0 | 0 |  |
| HAM/TSP#2 | 5952 | chrX_101416581_- | 3 | 1 | 0 | 0 | 0 |  |
| HAM/TSP#2 | 5953 | chrX_102724188_+ | 1 | 0 | 0 | 0 | 0 |  |
| HAM/TSP#2 | 5954 | chrX_103796844_- | 1 | 0 | 0 | 0 | 0 |  |
| HAM/TSP#2 | 5955 | chrX_104841649_+ | 3 | 0 | 0 | 0 | 0 |  |
| HAM/TSP#2 | 5956 | chrX_106039143_- | 2 | 0 | 0 | 0 | 0 |  |
| HAM/TSP#2 | 5957 | chrX_107733908_- | 1 | 0 | 0 | 0 | 0 |  |
| HAM/TSP#2 | 5958 | chrX_107742958_- | 2 | 0 | 0 | 0 | 0 |  |
| HAM/TSP#2 | 5959 | chrX_110294212_- | 0 | 1 | 0 | 0 | 0 |  |
| HAM/TSP#2 | 5960 | chrX_111230434_- | 1 | 0 | 0 | 0 | 0 |  |
| HAM/TSP#2 | 5961 | chrX_111534284_+ | 5 | 0 | 0 | 0 | 0 |  |
| HAM/TSP#2 | 5962 | chrX_111752428_+ | 1 | 0 | 0 | 0 | 0 |  |
| HAM/TSP#2 | 5963 | chrX_117998566_- | 0 | 1 | 0 | 0 | 0 |  |
| HAM/TSP#2 | 5964 | chrX_118083627_+ | 8 | 0 | 0 | 0 | 0 |  |
| HAM/TSP#2 | 5965 | chrX_118305133_- | 1 | 0 | 0 | 0 | 0 |  |
| HAM/TSP#2 | 5966 | chrX_118428737_+ | 3 | 0 | 0 | 0 | 0 |  |
| HAM/TSP#2 | 5967 | chrX_121313744_+ | 2 | 0 | 0 | 0 | 0 |  |
| HAM/TSP#2 | 5968 | chrX_124320158_+ | 11 | 0 | 1 | 0 | 0 |  |
| HAM/TSP#2 | 5969 | chrX_126910339_+ | 1 | 0 | 0 | 0 | 0 |  |
| HAM/TSP#2 | 5970 | chrX_127114985_+ | 0 | 1 | 0 | 0 | 0 |  |
| HAM/TSP#2 | 5971 | chrX_128184020_+ | 1 | 0 | 0 | 0 | 0 |  |
| HAM/TSP#2 | 5972 | chrX_130495003_- | 1 | 0 | 0 | 0 | 0 |  |
| HAM/TSP#2 | 5973 | chrX_131041399_+ | 1 | 0 | 0 | 0 | 0 |  |
| HAM/TSP#2 | 5974 | chrX_131804935_- | 26 | 0 | 0 | 0 | 1 |  |
| HAM/TSP#2 | 5975 | chrX_133610673_- | 1 | 0 | 0 | 0 | 0 |  |
| HAM/TSP#2 | 5976 | chrX_133794431_- | 1 | 0 | 0 | 0 | 0 |  |
| HAM/TSP#2 | 5977 | chrX_136175216_+ | 1 | 0 | 0 | 0 | 0 |  |
| HAM/TSP#2 | 5978 | chrX_136774339_- | 0 | 17 | 0 | 0 | 0 |  |
| HAM/TSP#2 | 5979 | chrX_137163603_+ | 1 | 1 | 0 | 0 | 0 |  |
| HAM/TSP#2 | 5980 | chrX_137186275_- | 1 | 0 | 0 | 0 | 0 |  |
| HAM/TSP#2 | 5981 | chrX_138589702_+ | 0 | 2 | 0 | 0 | 0 |  |
| HAM/TSP#2 | 5982 | chrX_139154606_- | 2 | 0 | 0 | 0 | 0 |  |
| HAM/TSP#2 | 5983 | chrX_140710594_+ | 1 | 0 | 0 | 0 | 0 |  |
| HAM/TSP#2 | 5984 | chrX_140832519_- | 1 | 0 | 0 | 0 | 0 |  |
| HAM/TSP#2 | 5985 | chrX_142248976_- | 1 | 0 | 0 | 0 | 0 |  |
| HAM/TSP#2 | 5986 | chrX_144655260_+ | 1 | 0 | 0 | 0 | 0 |  |
| HAM/TSP#2 | 5987 | chrX_146756535_+ | 4 | 0 | 0 | 0 | 0 |  |
| HAM/TSP#2 | 5988 | chrX_148189671_+ | 2 | 0 | 0 | 0 | 0 |  |
| HAM/TSP#2 | 5989 | chrX_150528994_- | 0 | 1 | 0 | 0 | 0 |  |
| HAM/TSP#2 | 5990 | chrX_152172079_+ | 1 | 0 | 0 | 0 | 0 |  |
| HAM/TSP#2 | 5991 | chrX_153813295_- | 1 | 0 | 0 | 0 | 0 |  |
| HAM/TSP#2 | 5992 | chrX_155181338_- | 1 | 0 | 0 | 0 | 0 |  |
| HAM/TSP#2 | 5993 | chrX_19837345_+ | 1 | 0 | 0 | 0 | 0 |  |
| HAM/TSP#2 | 5994 | chrX_25509631_+ | 1 | 0 | 0 | 0 | 0 |  |
| HAM/TSP#2 | 5995 | chrX_26129913_+ | 2 | 0 | 0 | 0 | 0 |  |
| HAM/TSP#2 | 5996 | chrX_27984919_+ | 1 | 0 | 0 | 0 | 0 |  |
| HAM/TSP#2 | 5997 | chrX_28222011_+ | 6 | 0 | 0 | 0 | 0 |  |
| HAM/TSP#2 | 5998 | chrX_35823258_- | 5 | 1 | 0 | 0 | 0 |  |
| HAM/TSP#2 | 5999 | chrX_36611610_- | 0 | 1 | 0 | 0 | 0 |  |
| HAM/TSP#2 | 6000 | chrX_39172261_+ | 2 | 0 | 0 | 0 | 0 |  |
| HAM/TSP#2 | 6001 | chrX_39868932_+ | 0 | 15 | 0 | 0 | 0 |  |
| HAM/TSP#2 | 6002 | chrX_40192413_+ | 1 | 0 | 0 | 0 | 0 |  |
| HAM/TSP#2 | 6003 | chrX_41614703_+ | 1 | 0 | 0 | 0 | 0 |  |
| HAM/TSP#2 | 6004 | chrX_41665781_- | 1 | 0 | 0 | 0 | 0 |  |
| HAM/TSP#2 | 6005 | chrX_42429186_+ | 5 | 0 | 0 | 0 | 0 |  |
| HAM/TSP#2 | 6006 | chrX_47620546_- | 1 | 0 | 0 | 0 | 0 |  |
| HAM/TSP#2 | 6007 | chrX_51590214_+ | 1 | 0 | 0 | 0 | 0 |  |
| HAM/TSP#2 | 6008 | chrX_52026537_+ | 1 | 0 | 0 | 0 | 0 |  |
| HAM/TSP#2 | 6009 | chrX_52436448_- | 1 | 0 | 0 | 0 | 0 |  |
| HAM/TSP#2 | 6010 | chrX_53751508_- | 2 | 0 | 0 | 0 | 0 |  |
| HAM/TSP#2 | 6011 | chrX_57225623_- | 1 | 0 | 0 | 0 | 0 |  |
| HAM/TSP#2 | 6012 | chrX_57918710_+ | 1 | 0 | 0 | 0 | 0 |  |
| HAM/TSP#2 | 6013 | chrX_63790456_- | 1 | 0 | 0 | 0 | 0 |  |
| HAM/TSP#2 | 6014 | chrX_65672459_- | 3 | 0 | 0 | 0 | 0 |  |
| HAM/TSP#2 | 6015 | chrX_65715772_+ | 3 | 0 | 0 | 0 | 0 |  |
| HAM/TSP#2 | 6016 | chrX_66826509_+ | 3 | 0 | 0 | 0 | 0 |  |
| HAM/TSP#2 | 6017 | chrX_68862038_- | 1 | 0 | 0 | 0 | 0 |  |
| HAM/TSP#2 | 6018 | chrX_68917917_+ | 0 | 1 | 0 | 0 | 0 |  |
| HAM/TSP#2 | 6019 | chrX_69149137_- | 2 | 0 | 0 | 0 | 0 |  |
| HAM/TSP#2 | 6020 | chrX_72302963_+ | 0 | 1 | 0 | 0 | 0 |  |
| HAM/TSP#2 | 6021 | chrX_78067388_+ | 1 | 0 | 0 | 0 | 0 |  |
| HAM/TSP#2 | 6022 | chrX_80806055_+ | 2 | 0 | 0 | 0 | 0 |  |
| HAM/TSP#2 | 6023 | chrX_82073242_+ | 1 | 0 | 0 | 0 | 0 |  |
| HAM/TSP#2 | 6024 | chrX_85216536_- | 3 | 0 | 0 | 0 | 0 |  |
| HAM/TSP#2 | 6025 | chrX_89136408_- | 1 | 0 | 0 | 0 | 0 |  |
| HAM/TSP#2 | 6026 | chrX_92372096_+ | 1 | 0 | 0 | 0 | 0 |  |
| HAM/TSP#2 | 6027 | chrX_93429849_+ | 1 | 0 | 0 | 0 | 0 |  |
| HAM/TSP#2 | 6028 | chrX_96679271_- | 4 | 0 | 0 | 0 | 0 |  |
| HAM/TSP#2 | 6029 | chrX_96862415_+ | 1 | 0 | 0 | 0 | 0 |  |
| HAM/TSP#2 | 6030 | chrX_97132306_- | 0 | 0 | 0 | 0 | 1 |  |
| HAM/TSP#2 | 6031 | chrX_97366091_- | 1 | 0 | 0 | 0 | 0 |  |
| HAM/TSP#2 | 6032 | chrX_97366092_- | 5 | 0 | 0 | 0 | 0 |  |
| HAM/TSP#2 | 6033 | chrX_99521707_- | 1 | 0 | 0 | 0 | 0 |  |
| HAM/TSP#2 | 6034 | chrY_11282186_+ | 0 | 0 | 0 | 0 | 1 |  |
| HAM/TSP#2 | 6035 | chrY_11305546_+ | 1 | 0 | 0 | 0 | 0 |  |
| HAM/TSP#2 | 6036 | chrY_11309809_- | 1 | 0 | 0 | 0 | 0 |  |
| HAM/TSP#2 | 6037 | chrY_11333255_- | 1 | 0 | 0 | 0 | 0 |  |
| HAM/TSP#2 | 6038 | chrY_11721940_+ | 1 | 0 | 0 | 0 | 0 |  |
| HAM/TSP#2 | 6039 | chrY_12375670_- | 2 | 0 | 0 | 0 | 0 |  |
| HAM/TSP#2 | 6040 | chrY_12866505_- | 2 | 0 | 0 | 0 | 0 |  |
| HAM/TSP#2 | 6041 | chrY_15844117_+ | 1 | 0 | 0 | 0 | 0 |  |
| HAM/TSP#2 | 6042 | chrY_19226903_- | 1 | 0 | 0 | 0 | 0 |  |
| HAM/TSP#2 | 6043 | chrY_19466682_+ | 1 | 0 | 0 | 0 | 0 |  |
| HAM/TSP#2 | 6044 | chrY_20053792_+ | 6 | 0 | 0 | 0 | 0 |  |
| HAM/TSP#2 | 6045 | chrY_9608775_- | 7 | 0 | 0 | 0 | 0 |  |
| HAM/TSP#3 | 6046 | chr1_102720051_+ | 1 | 0 | 0 | 0 | 0 |  |
| HAM/TSP#3 | 6047 | chr1_102862268_- | 13 | 0 | 0 | 0 | 0 |  |
| HAM/TSP#3 | 6048 | chr1_104207701_- | 1 | 0 | 0 | 0 | 0 |  |
| HAM/TSP#3 | 6049 | chr1_104905366_+ | 7 | 0 | 0 | 0 | 0 |  |
| HAM/TSP#3 | 6050 | chr1_105788713_- | 2 | 0 | 0 | 0 | 0 |  |
| HAM/TSP#3 | 6051 | chr1_106651071_- | 7 | 0 | 0 | 0 | 0 |  |
| HAM/TSP#3 | 6052 | chr1_108203079_+ | 11 | 0 | 0 | 13 | 0 |  |
| HAM/TSP#3 | 6053 | chr1_108879292_+ | 11 | 0 | 0 | 0 | 0 |  |
| HAM/TSP#3 | 6054 | chr1_108928987_- | 1 | 0 | 0 | 0 | 0 |  |
| HAM/TSP#3 | 6055 | chr1_10893571_+ | 0 | 1 | 0 | 0 | 0 |  |
| HAM/TSP#3 | 6056 | chr1_109654759_- | 0 | 1 | 0 | 0 | 0 |  |
| HAM/TSP#3 | 6057 | chr1_110162884_+ | 0 | 1 | 0 | 0 | 0 |  |
| HAM/TSP#3 | 6058 | chr1_110226410_+ | 0 | 1 | 0 | 0 | 0 |  |
| HAM/TSP#3 | 6059 | chr1_112615678_- | 5 | 0 | 0 | 0 | 0 |  |
| HAM/TSP#3 | 6060 | chr1_11345238_+ | 0 | 1 | 0 | 0 | 0 |  |
| HAM/TSP#3 | 6061 | chr1_113905074_+ | 0 | 1 | 0 | 0 | 0 |  |
| HAM/TSP#3 | 6062 | chr1_115477615_- | 8 | 0 | 0 | 0 | 0 |  |
| HAM/TSP#3 | 6063 | chr1_1163361_- | 0 | 0 | 1 | 0 | 0 |  |
| HAM/TSP#3 | 6064 | chr1_116735349_+ | 1 | 0 | 0 | 0 | 0 |  |
| HAM/TSP#3 | 6065 | chr1_116863594_+ | 4 | 0 | 0 | 0 | 0 |  |
| HAM/TSP#3 | 6066 | chr1_11749766_+ | 0 | 1 | 0 | 0 | 0 |  |
| HAM/TSP#3 | 6067 | chr1_117635225_- | 10 | 0 | 0 | 0 | 0 |  |
| HAM/TSP#3 | 6068 | chr1_118465536_+ | 3 | 0 | 0 | 0 | 0 |  |
| HAM/TSP#3 | 6069 | chr1_118477863_- | 15 | 0 | 0 | 0 | 0 |  |
| HAM/TSP#3 | 6070 | chr1_118600200_- | 8 | 0 | 0 | 0 | 0 |  |
| HAM/TSP#3 | 6071 | chr1_119368079_- | 5 | 0 | 0 | 0 | 0 |  |
| HAM/TSP#3 | 6072 | chr1_119368088_- | 9 | 0 | 0 | 0 | 0 |  |
| HAM/TSP#3 | 6073 | chr1_119467231_- | 0 | 1 | 0 | 0 | 0 |  |
| HAM/TSP#3 | 6074 | chr1_11966312_+ | 0 | 1 | 0 | 0 | 0 |  |
| HAM/TSP#3 | 6075 | chr1_121510845_- | 10 | 0 | 0 | 0 | 0 |  |
| HAM/TSP#3 | 6076 | chr1_12166821_- | 7 | 0 | 0 | 0 | 0 |  |
| HAM/TSP#3 | 6077 | chr1_121967816_+ | 1 | 0 | 0 | 0 | 0 |  |
| HAM/TSP#3 | 6078 | chr1_125111907_- | 1 | 0 | 0 | 0 | 0 |  |
| HAM/TSP#3 | 6079 | chr1_143439009_- | 0 | 2 | 0 | 0 | 0 |  |
| HAM/TSP#3 | 6080 | chr1_143588928_+ | 5 | 0 | 0 | 0 | 0 |  |
| HAM/TSP#3 | 6081 | chr1_14516461_- | 0 | 1 | 0 | 0 | 0 |  |
| HAM/TSP#3 | 6082 | chr1_147579458_+ | 0 | 1 | 0 | 0 | 0 |  |
| HAM/TSP#3 | 6083 | chr1_148356421_+ | 1 | 0 | 0 | 0 | 0 |  |
| HAM/TSP#3 | 6084 | chr1_150522161_- | 1 | 0 | 0 | 0 | 0 |  |
| HAM/TSP#3 | 6085 | chr1_152106328_- | 1 | 0 | 0 | 0 | 0 |  |
| HAM/TSP#3 | 6086 | chr1_152923682_+ | 1 | 0 | 0 | 0 | 0 |  |
| HAM/TSP#3 | 6087 | chr1_154577527_- | 0 | 1 | 0 | 0 | 0 |  |
| HAM/TSP#3 | 6088 | chr1_155453535_- | 0 | 1 | 0 | 0 | 0 |  |
| HAM/TSP#3 | 6089 | chr1_156140346_+ | 0 | 1 | 0 | 0 | 0 |  |
| HAM/TSP#3 | 6090 | chr1_156215209_- | 6 | 0 | 0 | 0 | 0 |  |
| HAM/TSP#3 | 6091 | chr1_157437553_- | 0 | 1 | 0 | 0 | 0 |  |
| HAM/TSP#3 | 6092 | chr1_157680920_- | 0 | 0 | 0 | 1 | 0 |  |
| HAM/TSP#3 | 6093 | chr1_158591813_+ | 3 | 0 | 0 | 0 | 0 |  |
| HAM/TSP#3 | 6094 | chr1_159573060_+ | 4 | 0 | 0 | 0 | 0 |  |
| HAM/TSP#3 | 6095 | chr1_162574500_+ | 8 | 0 | 0 | 0 | 0 |  |
| HAM/TSP#3 | 6096 | chr1_163057501_+ | 1 | 0 | 0 | 0 | 0 |  |
| HAM/TSP#3 | 6097 | chr1_165166427_- | 4 | 0 | 0 | 0 | 0 |  |
| HAM/TSP#3 | 6098 | chr1_166604097_- | 3 | 0 | 0 | 0 | 0 |  |
| HAM/TSP#3 | 6099 | chr1_167519332_+ | 4 | 0 | 0 | 0 | 0 |  |
| HAM/TSP#3 | 6100 | chr1_169578948_+ | 1 | 0 | 0 | 0 | 0 |  |
| HAM/TSP#3 | 6101 | chr1_169923830_- | 0 | 1 | 0 | 0 | 0 |  |
| HAM/TSP#3 | 6102 | chr1_170788030_- | 3 | 0 | 0 | 0 | 0 |  |
| HAM/TSP#3 | 6103 | chr1_171717310_+ | 17 | 0 | 0 | 0 | 0 |  |
| HAM/TSP#3 | 6104 | chr1_173456462_- | 2 | 0 | 0 | 0 | 0 |  |
| HAM/TSP#3 | 6105 | chr1_175450615_+ | 4 | 0 | 0 | 0 | 0 |  |
| HAM/TSP#3 | 6106 | chr1_177039978_+ | 0 | 1 | 0 | 0 | 0 |  |
| HAM/TSP#3 | 6107 | chr1_180065242_- | 0 | 1 | 0 | 0 | 0 |  |
| HAM/TSP#3 | 6108 | chr1_180156611_- | 3 | 0 | 0 | 0 | 0 |  |
| HAM/TSP#3 | 6109 | chr1_180187188_+ | 0 | 1 | 0 | 0 | 0 |  |
| HAM/TSP#3 | 6110 | chr1_18383965_- | 1 | 0 | 0 | 0 | 0 |  |
| HAM/TSP#3 | 6111 | chr1_185173035_- | 0 | 1 | 0 | 0 | 0 |  |
| HAM/TSP#3 | 6112 | chr1_186621186_+ | 0 | 1 | 0 | 0 | 0 |  |
| HAM/TSP#3 | 6113 | chr1_189512269_- | 0 | 0 | 0 | 1 | 0 |  |
| HAM/TSP#3 | 6114 | chr1_190354906_+ | 1 | 0 | 0 | 0 | 0 |  |
| HAM/TSP#3 | 6115 | chr1_19260343_+ | 0 | 1 | 0 | 0 | 0 |  |
| HAM/TSP#3 | 6116 | chr1_19477573_+ | 0 | 1 | 0 | 0 | 0 |  |
| HAM/TSP#3 | 6117 | chr1_195848618_- | 1 | 0 | 0 | 0 | 0 |  |
| HAM/TSP#3 | 6118 | chr1_196021703_- | 29 | 0 | 0 | 0 | 0 |  |
| HAM/TSP#3 | 6119 | chr1_197139622_+ | 1 | 0 | 0 | 0 | 0 |  |
| HAM/TSP#3 | 6120 | chr1_197880057_- | 0 | 0 | 0 | 1 | 0 |  |
| HAM/TSP#3 | 6121 | chr1_201510224_+ | 2 | 0 | 0 | 0 | 0 |  |
| HAM/TSP#3 | 6122 | chr1_202275727_- | 0 | 1 | 0 | 0 | 0 |  |
| HAM/TSP#3 | 6123 | chr1_203008884_+ | 1 | 0 | 0 | 0 | 0 |  |
| HAM/TSP#3 | 6124 | chr1_203795130_- | 1 | 0 | 0 | 0 | 0 |  |
| HAM/TSP#3 | 6125 | chr1_204210695_+ | 0 | 1 | 0 | 0 | 0 |  |
| HAM/TSP#3 | 6126 | chr1_204358198_+ | 0 | 1 | 0 | 0 | 0 |  |
| HAM/TSP#3 | 6127 | chr1_204567564_+ | 3 | 0 | 0 | 0 | 0 |  |
| HAM/TSP#3 | 6128 | chr1_205873124_+ | 0 | 1 | 0 | 0 | 0 |  |
| HAM/TSP#3 | 6129 | chr1_205947295_+ | 1 | 0 | 0 | 0 | 0 |  |
| HAM/TSP#3 | 6130 | chr1_210204569_- | 1 | 0 | 0 | 0 | 0 |  |
| HAM/TSP#3 | 6131 | chr1_212368650_+ | 9 | 0 | 0 | 0 | 0 |  |
| HAM/TSP#3 | 6132 | chr1_215165050_+ | 0 | 1 | 0 | 0 | 0 |  |
| HAM/TSP#3 | 6133 | chr1_216517381_+ | 6 | 0 | 0 | 0 | 0 |  |
| HAM/TSP#3 | 6134 | chr1_216777350_+ | 0 | 1 | 0 | 0 | 0 |  |
| HAM/TSP#3 | 6135 | chr1_219485399_- | 3 | 0 | 0 | 0 | 0 |  |
| HAM/TSP#3 | 6136 | chr1_220214907_- | 16 | 0 | 0 | 0 | 0 |  |
| HAM/TSP#3 | 6137 | chr1_22026296_+ | 2 | 0 | 0 | 0 | 0 |  |
| HAM/TSP#3 | 6138 | chr1_221609288_- | 1 | 0 | 0 | 0 | 0 |  |
| HAM/TSP#3 | 6139 | chr1_223327919_+ | 0 | 1 | 0 | 0 | 0 |  |
| HAM/TSP#3 | 6140 | chr1_223539228_- | 0 | 3 | 0 | 0 | 0 |  |
| HAM/TSP#3 | 6141 | chr1_223756418_- | 2 | 17 | 0 | 0 | 0 |  |
| HAM/TSP#3 | 6142 | chr1_224642849_- | 2 | 0 | 0 | 0 | 0 |  |
| HAM/TSP#3 | 6143 | chr1_225918356_+ | 0 | 15 | 0 | 0 | 0 |  |
| HAM/TSP#3 | 6144 | chr1_22641562_- | 0 | 1 | 0 | 0 | 0 |  |
| HAM/TSP#3 | 6145 | chr1_226682898_+ | 0 | 1 | 0 | 0 | 0 |  |
| HAM/TSP#3 | 6146 | chr1_226708421_- | 5 | 0 | 0 | 0 | 0 |  |
| HAM/TSP#3 | 6147 | chr1_231127802_- | 1 | 0 | 0 | 0 | 0 |  |
| HAM/TSP#3 | 6148 | chr1_232022160_+ | 2 | 0 | 0 | 0 | 0 |  |
| HAM/TSP#3 | 6149 | chr1_233306757_+ | 1 | 0 | 0 | 0 | 0 |  |
| HAM/TSP#3 | 6150 | chr1_233359619_+ | 0 | 1 | 0 | 0 | 0 |  |
| HAM/TSP#3 | 6151 | chr1_234165528_- | 1 | 0 | 0 | 0 | 0 |  |
| HAM/TSP#3 | 6152 | chr1_234206014_+ | 1 | 0 | 0 | 0 | 0 |  |
| HAM/TSP#3 | 6153 | chr1_234487613_+ | 0 | 1 | 0 | 0 | 0 |  |
| HAM/TSP#3 | 6154 | chr1_234780534_+ | 0 | 0 | 0 | 0 | 1 |  |
| HAM/TSP#3 | 6155 | chr1_239498761_+ | 3 | 0 | 0 | 0 | 0 |  |
| HAM/TSP#3 | 6156 | chr1_239617049_+ | 4 | 0 | 0 | 0 | 0 |  |
| HAM/TSP#3 | 6157 | chr1_239790145_+ | 1 | 0 | 0 | 0 | 0 |  |
| HAM/TSP#3 | 6158 | chr1_240824873_+ | 0 | 0 | 0 | 1 | 0 |  |
| HAM/TSP#3 | 6159 | chr1_241560827_+ | 1 | 0 | 0 | 0 | 0 |  |
| HAM/TSP#3 | 6160 | chr1_24159357_- | 0 | 1 | 0 | 0 | 0 |  |
| HAM/TSP#3 | 6161 | chr1_241768941_- | 3 | 0 | 0 | 0 | 0 |  |
| HAM/TSP#3 | 6162 | chr1_241966541_+ | 0 | 28 | 0 | 0 | 0 |  |
| HAM/TSP#3 | 6163 | chr1_242408720_+ | 4 | 0 | 0 | 0 | 0 |  |
| HAM/TSP#3 | 6164 | chr1_245095163_- | 0 | 1 | 0 | 0 | 0 |  |
| HAM/TSP#3 | 6165 | chr1_245247603_- | 1 | 0 | 0 | 0 | 0 |  |
| HAM/TSP#3 | 6166 | chr1_245649873_- | 12 | 0 | 0 | 0 | 0 |  |
| HAM/TSP#3 | 6167 | chr1_2461568_+ | 0 | 1 | 0 | 0 | 0 |  |
| HAM/TSP#3 | 6168 | chr1_246590108_+ | 0 | 1 | 0 | 0 | 0 |  |
| HAM/TSP#3 | 6169 | chr1_248224177_+ | 1 | 0 | 0 | 0 | 0 |  |
| HAM/TSP#3 | 6170 | chr1_25622348_+ | 2 | 0 | 0 | 0 | 0 |  |
| HAM/TSP#3 | 6171 | chr1_25873324_- | 0 | 1 | 0 | 0 | 0 |  |
| HAM/TSP#3 | 6172 | chr1_27006202_+ | 0 | 1 | 0 | 0 | 0 |  |
| HAM/TSP#3 | 6173 | chr1_27577932_- | 1 | 0 | 0 | 0 | 0 |  |
| HAM/TSP#3 | 6174 | chr1_27606089_- | 0 | 1 | 0 | 0 | 0 |  |
| HAM/TSP#3 | 6175 | chr1_29192835_+ | 1 | 0 | 0 | 0 | 0 |  |
| HAM/TSP#3 | 6176 | chr1_30761591_+ | 0 | 1 | 0 | 0 | 0 |  |
| HAM/TSP#3 | 6177 | chr1_31415914_- | 0 | 1 | 0 | 0 | 0 |  |
| HAM/TSP#3 | 6178 | chr1_31447366_+ | 1 | 0 | 0 | 0 | 0 |  |
| HAM/TSP#3 | 6179 | chr1_31766445_+ | 0 | 1 | 0 | 0 | 0 |  |
| HAM/TSP#3 | 6180 | chr1_32287716_- | 23 | 0 | 0 | 0 | 0 |  |
| HAM/TSP#3 | 6181 | chr1_32708327_+ | 1 | 0 | 0 | 0 | 0 |  |
| HAM/TSP#3 | 6182 | chr1_32741258_- | 0 | 1 | 0 | 0 | 0 |  |
| HAM/TSP#3 | 6183 | chr1_32804553_- | 0 | 1 | 0 | 0 | 0 |  |
| HAM/TSP#3 | 6184 | chr1_33045318_+ | 0 | 1 | 0 | 0 | 0 |  |
| HAM/TSP#3 | 6185 | chr1_33221365_+ | 0 | 1 | 0 | 0 | 0 |  |
| HAM/TSP#3 | 6186 | chr1_33474638_+ | 1 | 1 | 0 | 0 | 0 |  |
| HAM/TSP#3 | 6187 | chr1_3356578_+ | 0 | 1 | 0 | 0 | 0 |  |
| HAM/TSP#3 | 6188 | chr1_34216568_+ | 0 | 1 | 0 | 0 | 0 |  |
| HAM/TSP#3 | 6189 | chr1_34864088_- | 0 | 1 | 0 | 0 | 0 |  |
| HAM/TSP#3 | 6190 | chr1_3497775_+ | 0 | 0 | 0 | 1 | 0 |  |
| HAM/TSP#3 | 6191 | chr1_3542484_- | 0 | 1 | 0 | 0 | 0 |  |
| HAM/TSP#3 | 6192 | chr1_35769425_+ | 0 | 1 | 0 | 0 | 0 |  |
| HAM/TSP#3 | 6193 | chr1_35854675_+ | 1 | 0 | 0 | 0 | 0 |  |
| HAM/TSP#3 | 6194 | chr1_38833793_- | 2 | 0 | 0 | 0 | 0 |  |
| HAM/TSP#3 | 6195 | chr1_39548893_- | 2 | 0 | 0 | 0 | 0 |  |
| HAM/TSP#3 | 6196 | chr1_4076973_- | 0 | 1 | 0 | 0 | 0 |  |
| HAM/TSP#3 | 6197 | chr1_40983978_+ | 10 | 0 | 0 | 7 | 0 |  |
| HAM/TSP#3 | 6198 | chr1_41177054_- | 0 | 1 | 0 | 0 | 0 |  |
| HAM/TSP#3 | 6199 | chr1_41427150_- | 2 | 0 | 0 | 0 | 0 |  |
| HAM/TSP#3 | 6200 | chr1_4271800_- | 0 | 1 | 0 | 0 | 0 |  |
| HAM/TSP#3 | 6201 | chr1_4377907_- | 1 | 0 | 0 | 0 | 0 |  |
| HAM/TSP#3 | 6202 | chr1_4511760_+ | 3 | 0 | 0 | 0 | 0 |  |
| HAM/TSP#3 | 6203 | chr1_4513934_+ | 0 | 1 | 0 | 0 | 0 |  |
| HAM/TSP#3 | 6204 | chr1_46617892_- | 0 | 1 | 0 | 0 | 0 |  |
| HAM/TSP#3 | 6205 | chr1_4768065_+ | 0 | 1 | 0 | 0 | 0 |  |
| HAM/TSP#3 | 6206 | chr1_47723858_- | 10 | 0 | 0 | 0 | 0 |  |
| HAM/TSP#3 | 6207 | chr1_47923550_- | 5 | 0 | 0 | 0 | 0 |  |
| HAM/TSP#3 | 6208 | chr1_49243466_- | 0 | 22 | 0 | 0 | 0 |  |
| HAM/TSP#3 | 6209 | chr1_50970936_- | 2 | 0 | 0 | 0 | 0 |  |
| HAM/TSP#3 | 6210 | chr1_51617070_+ | 0 | 1 | 0 | 0 | 0 |  |
| HAM/TSP#3 | 6211 | chr1_52013771_- | 23 | 0 | 0 | 0 | 0 |  |
| HAM/TSP#3 | 6212 | chr1_53006731_+ | 1 | 0 | 0 | 0 | 0 |  |
| HAM/TSP#3 | 6213 | chr1_53689237_+ | 0 | 1 | 0 | 0 | 0 |  |
| HAM/TSP#3 | 6214 | chr1_56983616_+ | 0 | 1 | 0 | 0 | 0 |  |
| HAM/TSP#3 | 6215 | chr1_5816338_+ | 0 | 1 | 0 | 0 | 0 |  |
| HAM/TSP#3 | 6216 | chr1_58368393_+ | 0 | 1 | 0 | 0 | 0 |  |
| HAM/TSP#3 | 6217 | chr1_58622827_- | 0 | 1 | 0 | 0 | 0 |  |
| HAM/TSP#3 | 6218 | chr1_58957162_+ | 4 | 0 | 0 | 0 | 0 |  |
| HAM/TSP#3 | 6219 | chr1_58957173_+ | 1 | 0 | 0 | 0 | 0 |  |
| HAM/TSP#3 | 6220 | chr1_59653007_+ | 0 | 1 | 0 | 0 | 0 |  |
| HAM/TSP#3 | 6221 | chr1_61152580_+ | 5 | 0 | 0 | 0 | 0 |  |
| HAM/TSP#3 | 6222 | chr1_64699429_+ | 6 | 0 | 0 | 0 | 0 |  |
| HAM/TSP#3 | 6223 | chr1_6677149_- | 1 | 0 | 0 | 0 | 0 |  |
| HAM/TSP#3 | 6224 | chr1_67172573_- | 2 | 0 | 0 | 0 | 0 |  |
| HAM/TSP#3 | 6225 | chr1_68494054_- | 4 | 0 | 0 | 0 | 0 |  |
| HAM/TSP#3 | 6226 | chr1_69256691_+ | 28 | 0 | 0 | 0 | 0 |  |
| HAM/TSP#3 | 6227 | chr1_6975837_+ | 0 | 1 | 0 | 0 | 0 |  |
| HAM/TSP#3 | 6228 | chr1_71463890_- | 19 | 0 | 1 | 0 | 0 |  |
| HAM/TSP#3 | 6229 | chr1_72713195_+ | 20 | 0 | 0 | 0 | 0 |  |
| HAM/TSP#3 | 6230 | chr1_73679808_+ | 1 | 0 | 0 | 0 | 0 |  |
| HAM/TSP#3 | 6231 | chr1_75719228_+ | 22 | 0 | 0 | 0 | 0 |  |
| HAM/TSP#3 | 6232 | chr1_77530916_+ | 0 | 1 | 0 | 0 | 0 |  |
| HAM/TSP#3 | 6233 | chr1_78697074_+ | 2 | 0 | 0 | 0 | 0 |  |
| HAM/TSP#3 | 6234 | chr1_79140835_+ | 14 | 0 | 0 | 0 | 0 |  |
| HAM/TSP#3 | 6235 | chr1_79401132_+ | 9 | 0 | 0 | 0 | 0 |  |
| HAM/TSP#3 | 6236 | chr1_80692263_+ | 1 | 0 | 0 | 0 | 0 |  |
| HAM/TSP#3 | 6237 | chr1_81335660_- | 2 | 0 | 0 | 0 | 0 |  |
| HAM/TSP#3 | 6238 | chr1_81576388_- | 3 | 0 | 0 | 0 | 0 |  |
| HAM/TSP#3 | 6239 | chr1_8222918_+ | 0 | 1 | 0 | 0 | 0 |  |
| HAM/TSP#3 | 6240 | chr1_8577595_- | 2 | 0 | 0 | 0 | 0 |  |
| HAM/TSP#3 | 6241 | chr1_86716585_- | 3 | 0 | 0 | 0 | 0 |  |
| HAM/TSP#3 | 6242 | chr1_87983263_+ | 2 | 0 | 0 | 0 | 0 |  |
| HAM/TSP#3 | 6243 | chr1_88300810_- | 3 | 0 | 0 | 0 | 0 |  |
| HAM/TSP#3 | 6244 | chr1_906943_- | 0 | 0 | 1 | 0 | 0 |  |
| HAM/TSP#3 | 6245 | chr1_9146417_- | 0 | 1 | 0 | 0 | 0 |  |
| HAM/TSP#3 | 6246 | chr1_92916516_+ | 5 | 0 | 0 | 0 | 0 |  |
| HAM/TSP#3 | 6247 | chr1_9354873_+ | 5 | 0 | 0 | 0 | 0 |  |
| HAM/TSP#3 | 6248 | chr1_94767381_+ | 2 | 0 | 0 | 0 | 0 |  |
| HAM/TSP#3 | 6249 | chr1_95221461_+ | 1 | 0 | 0 | 0 | 0 |  |
| HAM/TSP#3 | 6250 | chr1_96775715_+ | 22 | 0 | 0 | 0 | 1 |  |
| HAM/TSP#3 | 6251 | chr1_96820917_- | 3 | 0 | 0 | 0 | 0 |  |
| HAM/TSP#3 | 6252 | chr1_98916728_+ | 2 | 0 | 0 | 0 | 0 |  |
| HAM/TSP#3 | 6253 | chr1_99714617_- | 0 | 1 | 0 | 0 | 0 |  |
| HAM/TSP#3 | 6254 | chr2_100271215_- | 0 | 1 | 0 | 0 | 0 |  |
| HAM/TSP#3 | 6255 | chr2_101663112_- | 7 | 0 | 0 | 0 | 0 |  |
| HAM/TSP#3 | 6256 | chr2_104439876_- | 1 | 0 | 0 | 0 | 0 |  |
| HAM/TSP#3 | 6257 | chr2_105071697_+ | 6 | 0 | 0 | 0 | 0 |  |
| HAM/TSP#3 | 6258 | chr2_106865387_- | 11 | 0 | 0 | 5 | 0 |  |
| HAM/TSP#3 | 6259 | chr2_109196750_- | 1 | 0 | 0 | 0 | 0 |  |
| HAM/TSP#3 | 6260 | chr2_109344860_+ | 0 | 1 | 0 | 0 | 0 |  |
| HAM/TSP#3 | 6261 | chr2_111922746_+ | 0 | 1 | 0 | 0 | 0 |  |
| HAM/TSP#3 | 6262 | chr2_114235174_+ | 0 | 1 | 0 | 0 | 0 |  |
| HAM/TSP#3 | 6263 | chr2_114497402_+ | 0 | 3 | 0 | 0 | 0 |  |
| HAM/TSP#3 | 6264 | chr2_11463409_- | 1 | 0 | 0 | 0 | 0 |  |
| HAM/TSP#3 | 6265 | chr2_116118234_+ | 2 | 0 | 0 | 0 | 0 |  |
| HAM/TSP#3 | 6266 | chr2_116609716_- | 2 | 0 | 0 | 0 | 0 |  |
| HAM/TSP#3 | 6267 | chr2_116609721_- | 18 | 0 | 0 | 0 | 0 |  |
| HAM/TSP#3 | 6268 | chr2_116759267_- | 1 | 0 | 0 | 0 | 0 |  |
| HAM/TSP#3 | 6269 | chr2_116759268_- | 3 | 0 | 0 | 0 | 0 |  |
| HAM/TSP#3 | 6270 | chr2_11707706_- | 0 | 1 | 0 | 0 | 0 |  |
| HAM/TSP#3 | 6271 | chr2_117227874_+ | 1 | 0 | 0 | 0 | 0 |  |
| HAM/TSP#3 | 6272 | chr2_118478443_+ | 1 | 0 | 0 | 0 | 0 |  |
| HAM/TSP#3 | 6273 | chr2_118551353_+ | 0 | 1 | 0 | 0 | 0 |  |
| HAM/TSP#3 | 6274 | chr2_120758512_- | 0 | 1 | 0 | 0 | 0 |  |
| HAM/TSP#3 | 6275 | chr2_120989348_+ | 0 | 0 | 0 | 1 | 0 |  |
| HAM/TSP#3 | 6276 | chr2_121738543_+ | 0 | 1 | 0 | 0 | 0 |  |
| HAM/TSP#3 | 6277 | chr2_122126809_+ | 1 | 0 | 0 | 0 | 0 |  |
| HAM/TSP#3 | 6278 | chr2_124500643_- | 4 | 0 | 0 | 0 | 0 |  |
| HAM/TSP#3 | 6279 | chr2_124812550_- | 1 | 0 | 0 | 0 | 0 |  |
| HAM/TSP#3 | 6280 | chr2_126943403_+ | 0 | 1 | 0 | 0 | 0 |  |
| HAM/TSP#3 | 6281 | chr2_127456804_+ | 1 | 0 | 0 | 0 | 0 |  |
| HAM/TSP#3 | 6282 | chr2_127750250_+ | 1 | 0 | 0 | 0 | 0 |  |
| HAM/TSP#3 | 6283 | chr2_128068182_+ | 1 | 0 | 0 | 0 | 0 |  |
| HAM/TSP#3 | 6284 | chr2_129296938_- | 0 | 1 | 0 | 0 | 0 |  |
| HAM/TSP#3 | 6285 | chr2_130391426_+ | 2 | 0 | 0 | 0 | 0 |  |
| HAM/TSP#3 | 6286 | chr2_132431204_+ | 1 | 0 | 0 | 0 | 0 |  |
| HAM/TSP#3 | 6287 | chr2_132514005_+ | 0 | 10 | 0 | 0 | 0 |  |
| HAM/TSP#3 | 6288 | chr2_134152226_- | 7 | 0 | 0 | 0 | 0 |  |
| HAM/TSP#3 | 6289 | chr2_134198992_- | 1 | 0 | 0 | 0 | 0 |  |
| HAM/TSP#3 | 6290 | chr2_134448026_- | 0 | 1 | 0 | 0 | 0 |  |
| HAM/TSP#3 | 6291 | chr2_136097414_- | 3 | 0 | 0 | 0 | 0 |  |
| HAM/TSP#3 | 6292 | chr2_136115399_- | 16 | 0 | 0 | 0 | 0 |  |
| HAM/TSP#3 | 6293 | chr2_13908743_- | 0 | 1 | 0 | 0 | 0 |  |
| HAM/TSP#3 | 6294 | chr2_139558429_+ | 7 | 0 | 0 | 1 | 0 |  |
| HAM/TSP#3 | 6295 | chr2_140750450_+ | 33 | 0 | 0 | 0 | 0 |  |
| HAM/TSP#3 | 6296 | chr2_141288346_- | 2 | 0 | 0 | 0 | 0 |  |
| HAM/TSP#3 | 6297 | chr2_143633555_- | 9 | 0 | 0 | 0 | 0 |  |
| HAM/TSP#3 | 6298 | chr2_143696226_- | 1 | 0 | 0 | 0 | 0 |  |
| HAM/TSP#3 | 6299 | chr2_146261147_- | 0 | 26 | 0 | 0 | 0 |  |
| HAM/TSP#3 | 6300 | chr2_146498698_+ | 6 | 0 | 0 | 0 | 0 |  |
| HAM/TSP#3 | 6301 | chr2_147526743_- | 5 | 0 | 0 | 0 | 0 |  |
| HAM/TSP#3 | 6302 | chr2_14805759_- | 1 | 0 | 0 | 0 | 0 |  |
| HAM/TSP#3 | 6303 | chr2_15158425_+ | 7 | 0 | 0 | 12 | 0 |  |
| HAM/TSP#3 | 6304 | chr2_152183077_- | 2 | 0 | 0 | 0 | 0 |  |
| HAM/TSP#3 | 6305 | chr2_152269816_- | 8 | 0 | 0 | 0 | 0 |  |
| HAM/TSP#3 | 6306 | chr2_152729354_- | 8 | 0 | 0 | 0 | 0 |  |
| HAM/TSP#3 | 6307 | chr2_153724049_+ | 5 | 0 | 0 | 0 | 0 |  |
| HAM/TSP#3 | 6308 | chr2_156356590_- | 1 | 0 | 0 | 0 | 0 |  |
| HAM/TSP#3 | 6309 | chr2_158447471_- | 0 | 1 | 0 | 0 | 0 |  |
| HAM/TSP#3 | 6310 | chr2_15882049_- | 13 | 0 | 0 | 0 | 0 |  |
| HAM/TSP#3 | 6311 | chr2_159050455_+ | 0 | 1 | 0 | 0 | 0 |  |
| HAM/TSP#3 | 6312 | chr2_159848563_+ | 7 | 0 | 0 | 0 | 0 |  |
| HAM/TSP#3 | 6313 | chr2_160420122_+ | 1 | 0 | 0 | 0 | 0 |  |
| HAM/TSP#3 | 6314 | chr2_160652163_- | 1 | 0 | 0 | 0 | 0 |  |
| HAM/TSP#3 | 6315 | chr2_161280200_+ | 10 | 0 | 0 | 0 | 0 |  |
| HAM/TSP#3 | 6316 | chr2_161281668_+ | 1 | 0 | 0 | 0 | 0 |  |
| HAM/TSP#3 | 6317 | chr2_168702213_- | 5 | 0 | 0 | 0 | 0 |  |
| HAM/TSP#3 | 6318 | chr2_16912778_- | 0 | 1 | 0 | 0 | 0 |  |
| HAM/TSP#3 | 6319 | chr2_170389374_+ | 2 | 0 | 0 | 0 | 0 |  |
| HAM/TSP#3 | 6320 | chr2_171114493_- | 0 | 1 | 0 | 0 | 0 |  |
| HAM/TSP#3 | 6321 | chr2_171318254_- | 3 | 0 | 0 | 0 | 0 |  |
| HAM/TSP#3 | 6322 | chr2_171487738_- | 12 | 0 | 0 | 0 | 0 |  |
| HAM/TSP#3 | 6323 | chr2_174683188_- | 0 | 1 | 0 | 0 | 0 |  |
| HAM/TSP#3 | 6324 | chr2_176534577_+ | 0 | 1 | 0 | 0 | 0 |  |
| HAM/TSP#3 | 6325 | chr2_1809245_- | 0 | 1 | 0 | 0 | 0 |  |
| HAM/TSP#3 | 6326 | chr2_183133462_+ | 22 | 0 | 0 | 0 | 0 |  |
| HAM/TSP#3 | 6327 | chr2_18328580_+ | 5 | 0 | 0 | 0 | 0 |  |
| HAM/TSP#3 | 6328 | chr2_183512666_+ | 1 | 0 | 0 | 0 | 0 |  |
| HAM/TSP#3 | 6329 | chr2_18638379_+ | 9 | 0 | 0 | 0 | 0 |  |
| HAM/TSP#3 | 6330 | chr2_18955884_- | 22 | 0 | 4 | 0 | 0 |  |
| HAM/TSP#3 | 6331 | chr2_191248108_+ | 8 | 0 | 0 | 0 | 0 |  |
| HAM/TSP#3 | 6332 | chr2_195792522_- | 1 | 0 | 0 | 0 | 0 |  |
| HAM/TSP#3 | 6333 | chr2_196225787_+ | 0 | 1 | 0 | 0 | 0 |  |
| HAM/TSP#3 | 6334 | chr2_198483227_+ | 0 | 1 | 0 | 0 | 0 |  |
| HAM/TSP#3 | 6335 | chr2_199819610_- | 5 | 0 | 0 | 0 | 0 |  |
| HAM/TSP#3 | 6336 | chr2_206004045_+ | 1 | 0 | 0 | 0 | 0 |  |
| HAM/TSP#3 | 6337 | chr2_207527887_+ | 1 | 0 | 0 | 0 | 0 |  |
| HAM/TSP#3 | 6338 | chr2_208693505_+ | 9 | 0 | 0 | 0 | 0 |  |
| HAM/TSP#3 | 6339 | chr2_20990577_- | 1 | 0 | 0 | 0 | 0 |  |
| HAM/TSP#3 | 6340 | chr2_20990601_- | 31 | 0 | 0 | 0 | 0 |  |
| HAM/TSP#3 | 6341 | chr2_210029253_- | 3 | 0 | 0 | 0 | 0 |  |
| HAM/TSP#3 | 6342 | chr2_211589895_- | 1 | 0 | 0 | 0 | 0 |  |
| HAM/TSP#3 | 6343 | chr2_214890336_- | 13 | 0 | 0 | 2 | 0 |  |
| HAM/TSP#3 | 6344 | chr2_217823703_- | 1 | 0 | 0 | 0 | 0 |  |
| HAM/TSP#3 | 6345 | chr2_219282019_- | 0 | 1 | 0 | 0 | 0 |  |
| HAM/TSP#3 | 6346 | chr2_219449920_- | 0 | 1 | 0 | 0 | 0 |  |
| HAM/TSP#3 | 6347 | chr2_222418991_+ | 2 | 0 | 0 | 0 | 0 |  |
| HAM/TSP#3 | 6348 | chr2_222925219_- | 3 | 0 | 0 | 0 | 0 |  |
| HAM/TSP#3 | 6349 | chr2_225031887_- | 1 | 0 | 5 | 0 | 0 |  |
| HAM/TSP#3 | 6350 | chr2_227433057_- | 1 | 0 | 0 | 0 | 0 |  |
| HAM/TSP#3 | 6351 | chr2_228562344_+ | 0 | 1 | 0 | 0 | 0 |  |
| HAM/TSP#3 | 6352 | chr2_230372873_- | 3 | 0 | 0 | 0 | 0 |  |
| HAM/TSP#3 | 6353 | chr2_23064804_- | 0 | 9 | 0 | 0 | 0 |  |
| HAM/TSP#3 | 6354 | chr2_231732833_- | 0 | 1 | 0 | 0 | 0 |  |
| HAM/TSP#3 | 6355 | chr2_234925399_- | 3 | 0 | 0 | 0 | 0 |  |
| HAM/TSP#3 | 6356 | chr2_235722734_+ | 5 | 0 | 0 | 0 | 0 |  |
| HAM/TSP#3 | 6357 | chr2_238124623_+ | 0 | 1 | 0 | 0 | 0 |  |
| HAM/TSP#3 | 6358 | chr2_238701768_- | 0 | 1 | 0 | 0 | 0 |  |
| HAM/TSP#3 | 6359 | chr2_239137106_- | 2 | 0 | 0 | 0 | 0 |  |
| HAM/TSP#3 | 6360 | chr2_239231352_+ | 1 | 0 | 0 | 0 | 0 |  |
| HAM/TSP#3 | 6361 | chr2_239944921_- | 9 | 0 | 0 | 0 | 0 |  |
| HAM/TSP#3 | 6362 | chr2_240298326_- | 0 | 1 | 0 | 0 | 0 |  |
| HAM/TSP#3 | 6363 | chr2_241864573_+ | 2 | 0 | 0 | 0 | 0 |  |
| HAM/TSP#3 | 6364 | chr2_242019848_+ | 3 | 0 | 0 | 0 | 0 |  |
| HAM/TSP#3 | 6365 | chr2_24207338_+ | 1 | 0 | 0 | 0 | 0 |  |
| HAM/TSP#3 | 6366 | chr2_24822374_+ | 0 | 1 | 0 | 0 | 0 |  |
| HAM/TSP#3 | 6367 | chr2_24840081_+ | 0 | 1 | 0 | 0 | 0 |  |
| HAM/TSP#3 | 6368 | chr2_25284338_+ | 1 | 0 | 0 | 0 | 0 |  |
| HAM/TSP#3 | 6369 | chr2_28205097_- | 1 | 0 | 0 | 0 | 0 |  |
| HAM/TSP#3 | 6370 | chr2_28988023_- | 0 | 6 | 0 | 0 | 0 |  |
| HAM/TSP#3 | 6371 | chr2_34200403_+ | 2 | 0 | 0 | 0 | 0 |  |
| HAM/TSP#3 | 6372 | chr2_34620854_+ | 2 | 0 | 0 | 0 | 0 |  |
| HAM/TSP#3 | 6373 | chr2_35356480_+ | 0 | 1 | 0 | 0 | 0 |  |
| HAM/TSP#3 | 6374 | chr2_36129015_- | 2 | 0 | 0 | 0 | 0 |  |
| HAM/TSP#3 | 6375 | chr2_37207898_- | 1 | 0 | 0 | 0 | 0 |  |
| HAM/TSP#3 | 6376 | chr2_37303078_+ | 0 | 1 | 0 | 0 | 0 |  |
| HAM/TSP#3 | 6377 | chr2_37317402_+ | 1 | 0 | 0 | 0 | 0 |  |
| HAM/TSP#3 | 6378 | chr2_3758161_- | 0 | 1 | 0 | 0 | 0 |  |
| HAM/TSP#3 | 6379 | chr2_41637010_+ | 0 | 1 | 0 | 0 | 0 |  |
| HAM/TSP#3 | 6380 | chr2_43380773_- | 1 | 0 | 0 | 0 | 0 |  |
| HAM/TSP#3 | 6381 | chr2_45252061_+ | 1 | 0 | 0 | 0 | 0 |  |
| HAM/TSP#3 | 6382 | chr2_46171202_- | 0 | 1 | 0 | 0 | 0 |  |
| HAM/TSP#3 | 6383 | chr2_46847983_+ | 0 | 1 | 0 | 0 | 0 |  |
| HAM/TSP#3 | 6384 | chr2_49384949_+ | 14 | 0 | 0 | 0 | 0 |  |
| HAM/TSP#3 | 6385 | chr2_50094900_+ | 10 | 0 | 0 | 0 | 0 |  |
| HAM/TSP#3 | 6386 | chr2_50558826_+ | 1 | 0 | 0 | 0 | 0 |  |
| HAM/TSP#3 | 6387 | chr2_52480945_+ | 4 | 0 | 0 | 0 | 0 |  |
| HAM/TSP#3 | 6388 | chr2_53257038_- | 0 | 1 | 0 | 0 | 0 |  |
| HAM/TSP#3 | 6389 | chr2_54023240_- | 1 | 0 | 0 | 0 | 0 |  |
| HAM/TSP#3 | 6390 | chr2_56033755_+ | 2 | 0 | 0 | 0 | 0 |  |
| HAM/TSP#3 | 6391 | chr2_57988355_+ | 0 | 1 | 0 | 0 | 0 |  |
| HAM/TSP#3 | 6392 | chr2_597353_- | 0 | 1 | 0 | 0 | 0 |  |
| HAM/TSP#3 | 6393 | chr2_60631805_+ | 1 | 0 | 0 | 0 | 0 |  |
| HAM/TSP#3 | 6394 | chr2_61064688_+ | 21 | 0 | 0 | 0 | 0 |  |
| HAM/TSP#3 | 6395 | chr2_62471028_+ | 42 | 0 | 1 | 0 | 0 |  |
| HAM/TSP#3 | 6396 | chr2_63733725_+ | 4 | 0 | 0 | 0 | 0 |  |
| HAM/TSP#3 | 6397 | chr2_65024849_- | 0 | 1 | 0 | 0 | 0 |  |
| HAM/TSP#3 | 6398 | chr2_6536756_+ | 0 | 1 | 0 | 0 | 0 |  |
| HAM/TSP#3 | 6399 | chr2_66350954_- | 12 | 0 | 0 | 0 | 0 |  |
| HAM/TSP#3 | 6400 | chr2_67976881_+ | 1 | 0 | 0 | 0 | 0 |  |
| HAM/TSP#3 | 6401 | chr2_71351064_+ | 4 | 0 | 0 | 0 | 0 |  |
| HAM/TSP#3 | 6402 | chr2_71610407_+ | 9 | 0 | 0 | 0 | 0 |  |
| HAM/TSP#3 | 6403 | chr2_72580425_- | 9 | 0 | 0 | 0 | 0 |  |
| HAM/TSP#3 | 6404 | chr2_73089105_+ | 2 | 0 | 0 | 0 | 0 |  |
| HAM/TSP#3 | 6405 | chr2_74288058_- | 0 | 1 | 0 | 0 | 0 |  |
| HAM/TSP#3 | 6406 | chr2_7430103_+ | 1 | 0 | 0 | 0 | 0 |  |
| HAM/TSP#3 | 6407 | chr2_74421505_+ | 0 | 1 | 0 | 0 | 0 |  |
| HAM/TSP#3 | 6408 | chr2_74546888_- | 8 | 0 | 0 | 0 | 0 |  |
| HAM/TSP#3 | 6409 | chr2_74548213_- | 0 | 36 | 0 | 0 | 0 |  |
| HAM/TSP#3 | 6410 | chr2_76860152_- | 12 | 0 | 0 | 0 | 0 |  |
| HAM/TSP#3 | 6411 | chr2_77503_+ | 1 | 0 | 0 | 0 | 0 |  |
| HAM/TSP#3 | 6412 | chr2_78194609_- | 5 | 0 | 0 | 0 | 0 |  |
| HAM/TSP#3 | 6413 | chr2_79406508_+ | 2 | 0 | 0 | 0 | 0 |  |
| HAM/TSP#3 | 6414 | chr2_79560915_+ | 1 | 0 | 0 | 0 | 0 |  |
| HAM/TSP#3 | 6415 | chr2_81008034_+ | 4 | 0 | 0 | 0 | 0 |  |
| HAM/TSP#3 | 6416 | chr2_8119124_+ | 1 | 0 | 0 | 0 | 0 |  |
| HAM/TSP#3 | 6417 | chr2_82304795_+ | 3 | 0 | 0 | 0 | 0 |  |
| HAM/TSP#3 | 6418 | chr2_82331425_- | 0 | 1 | 0 | 0 | 0 |  |
| HAM/TSP#3 | 6419 | chr2_84675522_- | 3 | 0 | 0 | 0 | 0 |  |
| HAM/TSP#3 | 6420 | chr2_88626566_+ | 0 | 0 | 0 | 13 | 0 |  |
| HAM/TSP#3 | 6421 | chr2_88741315_+ | 3 | 0 | 0 | 0 | 0 |  |
| HAM/TSP#3 | 6422 | chr2_89034081_+ | 0 | 1 | 0 | 0 | 0 |  |
| HAM/TSP#3 | 6423 | chr2_9083842_- | 9 | 0 | 0 | 0 | 0 |  |
| HAM/TSP#3 | 6424 | chr2_9094693_+ | 1 | 0 | 0 | 0 | 0 |  |
| HAM/TSP#3 | 6425 | chr2_91597177_- | 1 | 0 | 0 | 0 | 0 |  |
| HAM/TSP#3 | 6426 | chr2_9393040_+ | 3 | 0 | 0 | 0 | 0 |  |
| HAM/TSP#3 | 6427 | chr2_94821017_- | 2 | 0 | 0 | 0 | 0 |  |
| HAM/TSP#3 | 6428 | chr2_94908334_+ | 6 | 0 | 0 | 0 | 0 |  |
| HAM/TSP#3 | 6429 | chr2_9519409_- | 0 | 1 | 0 | 0 | 0 |  |
| HAM/TSP#3 | 6430 | chr2_96525991_+ | 1 | 0 | 0 | 0 | 0 |  |
| HAM/TSP#3 | 6431 | chr2_96568635_- | 0 | 1 | 0 | 0 | 0 |  |
| HAM/TSP#3 | 6432 | chr2_98281080_- | 0 | 1 | 0 | 0 | 0 |  |
| HAM/TSP#3 | 6433 | chr2_98710832_+ | 3 | 0 | 0 | 0 | 0 |  |
| HAM/TSP#3 | 6434 | chr3_100682034_+ | 0 | 1 | 0 | 0 | 0 |  |
| HAM/TSP#3 | 6435 | chr3_101177769_+ | 23 | 0 | 0 | 0 | 0 |  |
| HAM/TSP#3 | 6436 | chr3_102106813_+ | 7 | 0 | 0 | 0 | 0 |  |
| HAM/TSP#3 | 6437 | chr3_102893428_+ | 1 | 0 | 0 | 0 | 0 |  |
| HAM/TSP#3 | 6438 | chr3_10487466_+ | 0 | 1 | 0 | 0 | 0 |  |
| HAM/TSP#3 | 6439 | chr3_105339114_+ | 1 | 0 | 0 | 0 | 0 |  |
| HAM/TSP#3 | 6440 | chr3_10747367_+ | 0 | 1 | 0 | 0 | 0 |  |
| HAM/TSP#3 | 6441 | chr3_107747515_+ | 0 | 1 | 0 | 0 | 0 |  |
| HAM/TSP#3 | 6442 | chr3_111439089_+ | 0 | 2 | 0 | 0 | 0 |  |
| HAM/TSP#3 | 6443 | chr3_112134839_- | 7 | 0 | 0 | 0 | 0 |  |
| HAM/TSP#3 | 6444 | chr3_112438793_- | 16 | 0 | 0 | 0 | 0 |  |
| HAM/TSP#3 | 6445 | chr3_117707781_- | 1 | 0 | 0 | 0 | 0 |  |
| HAM/TSP#3 | 6446 | chr3_11837123_+ | 1 | 0 | 0 | 0 | 0 |  |
| HAM/TSP#3 | 6447 | chr3_11855437_- | 0 | 1 | 0 | 0 | 0 |  |
| HAM/TSP#3 | 6448 | chr3_119609473_+ | 0 | 1 | 0 | 0 | 0 |  |
| HAM/TSP#3 | 6449 | chr3_120043074_+ | 0 | 1 | 0 | 0 | 0 |  |
| HAM/TSP#3 | 6450 | chr3_120726699_- | 1 | 0 | 0 | 0 | 0 |  |
| HAM/TSP#3 | 6451 | chr3_122418891_- | 1 | 0 | 0 | 0 | 0 |  |
| HAM/TSP#3 | 6452 | chr3_123015937_+ | 2 | 0 | 0 | 0 | 0 |  |
| HAM/TSP#3 | 6453 | chr3_12310322_+ | 11 | 0 | 0 | 0 | 0 |  |
| HAM/TSP#3 | 6454 | chr3_123680233_- | 0 | 0 | 0 | 1 | 0 |  |
| HAM/TSP#3 | 6455 | chr3_12403296_- | 2 | 0 | 0 | 0 | 0 |  |
| HAM/TSP#3 | 6456 | chr3_124091671_- | 1 | 4 | 0 | 0 | 0 |  |
| HAM/TSP#3 | 6457 | chr3_125308215_- | 1 | 0 | 0 | 0 | 0 |  |
| HAM/TSP#3 | 6458 | chr3_127158199_- | 0 | 1 | 0 | 0 | 0 |  |
| HAM/TSP#3 | 6459 | chr3_127733642_- | 0 | 1 | 0 | 0 | 0 |  |
| HAM/TSP#3 | 6460 | chr3_12804458_- | 4 | 0 | 0 | 0 | 0 |  |
| HAM/TSP#3 | 6461 | chr3_128212888_- | 0 | 3 | 0 | 0 | 0 |  |
| HAM/TSP#3 | 6462 | chr3_128496153_- | 0 | 1 | 0 | 0 | 0 |  |
| HAM/TSP#3 | 6463 | chr3_128912869_+ | 0 | 1 | 0 | 0 | 0 |  |
| HAM/TSP#3 | 6464 | chr3_129368196_- | 0 | 1 | 0 | 0 | 0 |  |
| HAM/TSP#3 | 6465 | chr3_130547121_+ | 0 | 1 | 0 | 0 | 0 |  |
| HAM/TSP#3 | 6466 | chr3_131973160_+ | 4 | 0 | 0 | 0 | 0 |  |
| HAM/TSP#3 | 6467 | chr3_133391683_- | 2 | 4 | 0 | 1 | 0 |  |
| HAM/TSP#3 | 6468 | chr3_134970233_+ | 12 | 0 | 0 | 0 | 0 |  |
| HAM/TSP#3 | 6469 | chr3_136198398_+ | 7 | 0 | 0 | 0 | 0 |  |
| HAM/TSP#3 | 6470 | chr3_13749088_+ | 0 | 1 | 0 | 0 | 0 |  |
| HAM/TSP#3 | 6471 | chr3_138258676_+ | 0 | 1 | 0 | 0 | 0 |  |
| HAM/TSP#3 | 6472 | chr3_141258304_- | 0 | 0 | 0 | 0 | 1 |  |
| HAM/TSP#3 | 6473 | chr3_14283812_- | 0 | 1 | 0 | 0 | 0 |  |
| HAM/TSP#3 | 6474 | chr3_143534616_+ | 19 | 0 | 0 | 0 | 0 |  |
| HAM/TSP#3 | 6475 | chr3_143833871_- | 3 | 0 | 0 | 0 | 0 |  |
| HAM/TSP#3 | 6476 | chr3_145134404_- | 2 | 0 | 0 | 0 | 0 |  |
| HAM/TSP#3 | 6477 | chr3_147191707_+ | 4 | 0 | 0 | 0 | 0 |  |
| HAM/TSP#3 | 6478 | chr3_149223478_+ | 4 | 0 | 0 | 0 | 0 |  |
| HAM/TSP#3 | 6479 | chr3_14952945_+ | 14 | 0 | 0 | 0 | 0 |  |
| HAM/TSP#3 | 6480 | chr3_149734987_+ | 0 | 0 | 0 | 1 | 0 |  |
| HAM/TSP#3 | 6481 | chr3_149766361_- | 3 | 0 | 0 | 0 | 0 |  |
| HAM/TSP#3 | 6482 | chr3_151883699_- | 3 | 0 | 0 | 0 | 0 |  |
| HAM/TSP#3 | 6483 | chr3_152684986_- | 0 | 1 | 0 | 0 | 0 |  |
| HAM/TSP#3 | 6484 | chr3_15341254_- | 3 | 5 | 0 | 0 | 0 |  |
| HAM/TSP#3 | 6485 | chr3_15341652_+ | 1 | 0 | 0 | 0 | 0 |  |
| HAM/TSP#3 | 6486 | chr3_153564537_- | 0 | 1 | 0 | 0 | 0 |  |
| HAM/TSP#3 | 6487 | chr3_15393220_- | 18 | 0 | 0 | 0 | 0 |  |
| HAM/TSP#3 | 6488 | chr3_155374295_- | 0 | 1 | 0 | 0 | 0 |  |
| HAM/TSP#3 | 6489 | chr3_156289183_- | 0 | 1 | 0 | 0 | 0 |  |
| HAM/TSP#3 | 6490 | chr3_156775202_+ | 1 | 0 | 0 | 0 | 0 |  |
| HAM/TSP#3 | 6491 | chr3_157872866_- | 0 | 1 | 0 | 0 | 0 |  |
| HAM/TSP#3 | 6492 | chr3_158515487_- | 6 | 0 | 0 | 0 | 0 |  |
| HAM/TSP#3 | 6493 | chr3_159863611_+ | 3 | 0 | 0 | 0 | 0 |  |
| HAM/TSP#3 | 6494 | chr3_160230657_- | 5 | 0 | 0 | 0 | 0 |  |
| HAM/TSP#3 | 6495 | chr3_160287136_- | 0 | 4 | 0 | 0 | 0 |  |
| HAM/TSP#3 | 6496 | chr3_161837993_+ | 0 | 1 | 0 | 0 | 0 |  |
| HAM/TSP#3 | 6497 | chr3_16188652_+ | 5 | 0 | 0 | 0 | 0 |  |
| HAM/TSP#3 | 6498 | chr3_162047547_- | 0 | 1 | 0 | 0 | 0 |  |
| HAM/TSP#3 | 6499 | chr3_164120835_+ | 0 | 1 | 0 | 0 | 0 |  |
| HAM/TSP#3 | 6500 | chr3_165675287_+ | 0 | 83 | 0 | 0 | 0 |  |
| HAM/TSP#3 | 6501 | chr3_166516299_+ | 3 | 0 | 0 | 0 | 0 |  |
| HAM/TSP#3 | 6502 | chr3_166668976_- | 1 | 0 | 0 | 0 | 0 |  |
| HAM/TSP#3 | 6503 | chr3_167128240_- | 1 | 0 | 0 | 0 | 0 |  |
| HAM/TSP#3 | 6504 | chr3_171057449_+ | 0 | 1 | 0 | 0 | 0 |  |
| HAM/TSP#3 | 6505 | chr3_171459903_- | 0 | 1 | 0 | 0 | 0 |  |
| HAM/TSP#3 | 6506 | chr3_172491305_+ | 1 | 0 | 0 | 0 | 0 |  |
| HAM/TSP#3 | 6507 | chr3_177555187_- | 0 | 1 | 0 | 0 | 0 |  |
| HAM/TSP#3 | 6508 | chr3_177586882_- | 0 | 1 | 0 | 0 | 0 |  |
| HAM/TSP#3 | 6509 | chr3_177744942_+ | 23 | 0 | 0 | 0 | 0 |  |
| HAM/TSP#3 | 6510 | chr3_17818197_- | 0 | 1 | 0 | 0 | 0 |  |
| HAM/TSP#3 | 6511 | chr3_178705689_+ | 19 | 0 | 0 | 0 | 0 |  |
| HAM/TSP#3 | 6512 | chr3_179441287_+ | 0 | 1 | 0 | 0 | 0 |  |
| HAM/TSP#3 | 6513 | chr3_182777782_+ | 2 | 0 | 0 | 0 | 0 |  |
| HAM/TSP#3 | 6514 | chr3_184810962_+ | 0 | 9 | 0 | 0 | 0 |  |
| HAM/TSP#3 | 6515 | chr3_185260923_- | 1 | 0 | 0 | 0 | 0 |  |
| HAM/TSP#3 | 6516 | chr3_18634627_- | 0 | 2 | 0 | 0 | 0 |  |
| HAM/TSP#3 | 6517 | chr3_187398039_+ | 6 | 0 | 0 | 0 | 0 |  |
| HAM/TSP#3 | 6518 | chr3_188208704_+ | 6 | 0 | 0 | 0 | 0 |  |
| HAM/TSP#3 | 6519 | chr3_193052997_- | 0 | 1 | 0 | 0 | 0 |  |
| HAM/TSP#3 | 6520 | chr3_193962549_- | 1 | 0 | 0 | 0 | 0 |  |
| HAM/TSP#3 | 6521 | chr3_195070790_+ | 1 | 0 | 0 | 0 | 0 |  |
| HAM/TSP#3 | 6522 | chr3_195784215_- | 0 | 1 | 0 | 0 | 0 |  |
| HAM/TSP#3 | 6523 | chr3_196809536_- | 2 | 0 | 0 | 0 | 0 |  |
| HAM/TSP#3 | 6524 | chr3_197881428_- | 1 | 4 | 0 | 0 | 0 |  |
| HAM/TSP#3 | 6525 | chr3_198130232_+ | 14 | 0 | 0 | 0 | 0 |  |
| HAM/TSP#3 | 6526 | chr3_1990484_+ | 2 | 0 | 0 | 0 | 0 |  |
| HAM/TSP#3 | 6527 | chr3_21621329_- | 0 | 2 | 0 | 0 | 0 |  |
| HAM/TSP#3 | 6528 | chr3_21826657_+ | 0 | 1 | 0 | 0 | 0 |  |
| HAM/TSP#3 | 6529 | chr3_23283338_- | 9 | 0 | 0 | 0 | 0 |  |
| HAM/TSP#3 | 6530 | chr3_23486943_- | 0 | 1 | 0 | 0 | 0 |  |
| HAM/TSP#3 | 6531 | chr3_26053241_- | 0 | 1 | 0 | 0 | 0 |  |
| HAM/TSP#3 | 6532 | chr3_27399982_- | 9 | 0 | 0 | 0 | 0 |  |
| HAM/TSP#3 | 6533 | chr3_27498166_+ | 83 | 4 | 0 | 0 | 0 |  |
| HAM/TSP#3 | 6534 | chr3_29073272_+ | 1 | 0 | 0 | 0 | 0 |  |
| HAM/TSP#3 | 6535 | chr3_29073273_+ | 8 | 0 | 0 | 0 | 0 |  |
| HAM/TSP#3 | 6536 | chr3_31358699_- | 7 | 0 | 0 | 0 | 0 |  |
| HAM/TSP#3 | 6537 | chr3_31444126_+ | 4 | 0 | 0 | 0 | 0 |  |
| HAM/TSP#3 | 6538 | chr3_31518745_- | 1 | 0 | 0 | 0 | 0 |  |
| HAM/TSP#3 | 6539 | chr3_32156658_+ | 3 | 0 | 0 | 0 | 0 |  |
| HAM/TSP#3 | 6540 | chr3_33558279_+ | 6 | 0 | 0 | 0 | 0 |  |
[truncated: 357,129 more chars]
