# Supplementary material for: Human T-cell leukemia virus type 1 infects multiple lineage hematopoietic cells in vivo
Source: PLoS Pathog. 2017 Nov 29;13(11):e1006722. doi: 10.1371/journal.ppat.1006722 (PMC5724899; doi:10.1371/journal.ppat.1006722)
Supplement: S6 Table — Integration sites of HTLV-1 provirus in various hematopoietic cells and neutrophils at different time point were shown. (DOCX) [file ppat.1006722.s009.docx]

**Table S6. Clonality of HTLV-1 infected cells at different time point**

| Patient | ID | Integration sites | CD4 | CD8 | B | Mono | Neut | Neut  (1year) |
| --- | --- | --- | --- | --- | --- | --- | --- | --- |
| HAM/TSP#2 | 1 | chr3_135065190_- | 141 | 23 | 19 | 7 | 6 | 49 |
| HAM/TSP#2 | 2 | chr11_11058804_+ | 41 | 2 | 0 | 0 | 0 | 35 |
| HAM/TSP#2 | 3 | chr17_78983325_- | 26 | 0 | 0 | 0 | 0 | 23 |
| HAM/TSP#2 | 4 | chr17_21320041_- | 0 | 0 | 0 | 0 | 0 | 20 |
| HAM/TSP#2 | 5 | chr12_74674108_+ | 14 | 0 | 0 | 0 | 0 | 15 |
| HAM/TSP#2 | 6 | chr14_43001842_- | 22 | 2 | 0 | 0 | 0 | 15 |
| HAM/TSP#2 | 7 | chr19_50721954_- | 0 | 0 | 0 | 0 | 0 | 15 |
| HAM/TSP#2 | 8 | chr8_88265102_- | 47 | 1 | 0 | 0 | 5 | 14 |
| HAM/TSP#2 | 9 | chr6_27838814_- | 3 | 0 | 0 | 0 | 0 | 13 |
| HAM/TSP#2 | 10 | chr3_113130374_- | 3 | 0 | 0 | 0 | 0 | 12 |
| HAM/TSP#2 | 11 | chr4_159101268_- | 13 | 2 | 0 | 0 | 0 | 11 |
| HAM/TSP#2 | 12 | chr10_52916386_- | 9 | 0 | 0 | 0 | 0 | 11 |
| HAM/TSP#2 | 13 | chr10_66811159_- | 4 | 0 | 0 | 0 | 0 | 11 |
| HAM/TSP#2 | 14 | chr13_68123438_+ | 0 | 0 | 0 | 0 | 0 | 11 |
| HAM/TSP#2 | 15 | chr2_54634118_+ | 1 | 0 | 0 | 0 | 0 | 10 |
| HAM/TSP#2 | 16 | chr4_25423836_+ | 0 | 0 | 0 | 0 | 0 | 10 |
| HAM/TSP#2 | 17 | chr6_122598571_- | 2 | 0 | 0 | 0 | 0 | 10 |
| HAM/TSP#2 | 18 | chr14_31585400_+ | 12 | 0 | 0 | 0 | 0 | 10 |
| HAM/TSP#2 | 19 | chrX_118428738_+ | 0 | 0 | 0 | 0 | 0 | 10 |
| HAM/TSP#2 | 20 | chr11_110928548_- | 0 | 0 | 0 | 0 | 0 | 9 |
| HAM/TSP#2 | 21 | chr11_75762578_+ | 0 | 0 | 0 | 0 | 0 | 9 |
| HAM/TSP#2 | 22 | chr17_28806395_+ | 10 | 0 | 0 | 13 | 1 | 9 |
| HAM/TSP#2 | 23 | chr20_16377119_- | 0 | 10 | 0 | 0 | 0 | 9 |
| HAM/TSP#2 | 24 | chr6_10649640_+ | 14 | 0 | 0 | 0 | 0 | 8 |
| HAM/TSP#2 | 25 | chr8_107456232_+ | 36 | 0 | 0 | 0 | 0 | 8 |
| HAM/TSP#2 | 26 | chr11_86183140_- | 4 | 0 | 0 | 0 | 0 | 8 |
| HAM/TSP#2 | 27 | chr13_54417902_- | 4 | 0 | 8 | 1 | 0 | 8 |
| HAM/TSP#2 | 28 | chr1_114832549_- | 14 | 0 | 0 | 0 | 0 | 7 |
| HAM/TSP#2 | 29 | chr1_20185300_- | 4 | 0 | 0 | 0 | 0 | 7 |
| HAM/TSP#2 | 30 | chr8_77222706_- | 20 | 0 | 0 | 0 | 0 | 7 |
| HAM/TSP#2 | 31 | chr8_98013473_- | 0 | 0 | 0 | 0 | 0 | 7 |
| HAM/TSP#2 | 32 | chr13_44620925_- | 40 | 1 | 0 | 0 | 0 | 7 |
| HAM/TSP#2 | 33 | chr13_61057374_- | 0 | 0 | 0 | 0 | 0 | 7 |
| HAM/TSP#2 | 34 | chr21_41207246_+ | 0 | 0 | 0 | 0 | 0 | 7 |
| HAM/TSP#2 | 35 | chrX_125443284_+ | 0 | 0 | 0 | 0 | 0 | 7 |
| HAM/TSP#2 | 36 | chrX_131804935_- | 26 | 0 | 0 | 0 | 1 | 7 |
| HAM/TSP#2 | 37 | chr6_149953520_- | 0 | 0 | 0 | 0 | 0 | 6 |
| HAM/TSP#2 | 38 | chr7_42296504_- | 5 | 1 | 0 | 0 | 0 | 6 |
| HAM/TSP#2 | 39 | chr9_69187500_+ | 5 | 0 | 0 | 0 | 0 | 6 |
| HAM/TSP#2 | 40 | chr10_60513442_+ | 0 | 0 | 0 | 0 | 0 | 6 |
| HAM/TSP#2 | 41 | chr10_8314930_+ | 13 | 0 | 0 | 0 | 0 | 6 |
| HAM/TSP#2 | 42 | chr15_38085057_+ | 12 | 1 | 0 | 0 | 0 | 6 |
| HAM/TSP#2 | 43 | chrX_39172261_+ | 2 | 0 | 0 | 0 | 0 | 6 |
| HAM/TSP#2 | 44 | chr1_175827595_- | 2 | 0 | 0 | 0 | 0 | 5 |
| HAM/TSP#2 | 45 | chr1_213810482_+ | 30 | 0 | 0 | 0 | 0 | 5 |
| HAM/TSP#2 | 46 | chr1_80085212_+ | 2 | 0 | 0 | 0 | 0 | 5 |
| HAM/TSP#2 | 47 | chr1_84018661_+ | 4 | 0 | 0 | 0 | 0 | 5 |
| HAM/TSP#2 | 48 | chr4_121223590_+ | 5 | 0 | 0 | 0 | 0 | 5 |
| HAM/TSP#2 | 49 | chr5_155386718_- | 0 | 0 | 0 | 0 | 0 | 5 |
| HAM/TSP#2 | 50 | chr5_180871886_- | 31 | 0 | 0 | 0 | 0 | 5 |
| HAM/TSP#2 | 51 | chr11_24894022_- | 2 | 0 | 0 | 0 | 0 | 5 |
| HAM/TSP#2 | 52 | chr11_81596650_+ | 17 | 0 | 0 | 0 | 0 | 5 |
| HAM/TSP#2 | 53 | chr16_58454064_- | 13 | 0 | 0 | 0 | 0 | 5 |
| HAM/TSP#2 | 54 | chr20_56419024_- | 0 | 0 | 0 | 0 | 0 | 5 |
| HAM/TSP#2 | 55 | chr21_14854173_- | 0 | 0 | 0 | 0 | 0 | 5 |
| HAM/TSP#2 | 56 | chr21_22374789_+ | 0 | 0 | 0 | 0 | 0 | 5 |
| HAM/TSP#2 | 57 | chr21_23841471_- | 3 | 0 | 0 | 0 | 0 | 5 |
| HAM/TSP#2 | 58 | chr22_47871460_- | 2 | 0 | 0 | 0 | 0 | 5 |
| HAM/TSP#2 | 59 | chrX_56169257_- | 0 | 0 | 0 | 0 | 0 | 5 |
| HAM/TSP#2 | 60 | chrY_12866505_- | 2 | 0 | 0 | 0 | 0 | 5 |
| HAM/TSP#2 | 61 | chr1_214053082_- | 0 | 0 | 0 | 0 | 0 | 4 |
| HAM/TSP#2 | 62 | chr1_223756419_- | 1 | 0 | 0 | 0 | 0 | 4 |
| HAM/TSP#2 | 63 | chr1_45852145_+ | 0 | 0 | 0 | 0 | 0 | 4 |
| HAM/TSP#2 | 64 | chr1_53441789_+ | 0 | 0 | 0 | 0 | 0 | 4 |
| HAM/TSP#2 | 65 | chr1_81153528_- | 1 | 0 | 0 | 0 | 0 | 4 |
| HAM/TSP#2 | 66 | chr3_163436806_- | 2 | 0 | 0 | 0 | 0 | 4 |
| HAM/TSP#2 | 67 | chr4_67302527_- | 3 | 0 | 0 | 0 | 0 | 4 |
| HAM/TSP#2 | 68 | chr4_87282595_+ | 5 | 0 | 0 | 0 | 0 | 4 |
| HAM/TSP#2 | 69 | chr7_48279834_- | 1 | 0 | 0 | 0 | 0 | 4 |
| HAM/TSP#2 | 70 | chr9_12152098_+ | 4 | 2 | 0 | 0 | 0 | 4 |
| HAM/TSP#2 | 71 | chr11_106758849_- | 1 | 0 | 0 | 0 | 0 | 4 |
| HAM/TSP#2 | 72 | chr11_24737889_- | 0 | 0 | 0 | 0 | 0 | 4 |
| HAM/TSP#2 | 73 | chr13_42699193_+ | 7 | 0 | 0 | 0 | 0 | 4 |
| HAM/TSP#2 | 74 | chr13_62651098_+ | 2 | 0 | 0 | 0 | 0 | 4 |
| HAM/TSP#2 | 75 | chr13_70800458_+ | 0 | 0 | 0 | 0 | 0 | 4 |
| HAM/TSP#2 | 76 | chr14_106354174_- | 2 | 0 | 0 | 0 | 0 | 4 |
| HAM/TSP#2 | 77 | chr15_77813334_+ | 0 | 0 | 0 | 0 | 0 | 4 |
| HAM/TSP#2 | 78 | chr22_47698578_+ | 0 | 0 | 0 | 0 | 0 | 4 |
| HAM/TSP#2 | 79 | chrX_109558669_+ | 0 | 0 | 0 | 0 | 0 | 4 |
| HAM/TSP#2 | 80 | chrX_140710594_+ | 1 | 0 | 0 | 0 | 0 | 4 |
| HAM/TSP#2 | 81 | chr2_76332237_+ | 0 | 0 | 0 | 0 | 0 | 3 |
| HAM/TSP#2 | 82 | chr2_80471984_+ | 25 | 0 | 0 | 0 | 0 | 3 |
| HAM/TSP#2 | 83 | chr3_110239201_+ | 8 | 0 | 0 | 0 | 0 | 3 |
| HAM/TSP#2 | 84 | chr3_63322269_+ | 11 | 0 | 0 | 0 | 0 | 3 |
| HAM/TSP#2 | 85 | chr4_17141445_- | 1 | 0 | 0 | 0 | 0 | 3 |
| HAM/TSP#2 | 86 | chr4_32879498_+ | 1 | 0 | 0 | 0 | 0 | 3 |
| HAM/TSP#2 | 87 | chr5_40062957_+ | 0 | 0 | 0 | 0 | 0 | 3 |
| HAM/TSP#2 | 88 | chr6_124439268_+ | 0 | 0 | 0 | 0 | 0 | 3 |
| HAM/TSP#2 | 89 | chr9_120898014_+ | 0 | 0 | 0 | 0 | 0 | 3 |
| HAM/TSP#2 | 90 | chr9_41037631_- | 10 | 0 | 0 | 0 | 0 | 3 |
| HAM/TSP#2 | 91 | chr9_45322854_+ | 0 | 0 | 0 | 0 | 1 | 3 |
| HAM/TSP#2 | 92 | chr9_93912503_- | 10 | 0 | 0 | 0 | 0 | 3 |
| HAM/TSP#2 | 93 | chr13_97321094_+ | 6 | 0 | 0 | 0 | 0 | 3 |
| HAM/TSP#2 | 94 | chr14_103005898_+ | 0 | 0 | 0 | 0 | 0 | 3 |
| HAM/TSP#2 | 95 | chr15_47725972_- | 4 | 0 | 0 | 0 | 0 | 3 |
| HAM/TSP#2 | 96 | chr16_60400219_+ | 8 | 0 | 0 | 0 | 0 | 3 |
| HAM/TSP#2 | 97 | chr17_5106162_- | 0 | 0 | 0 | 0 | 0 | 3 |
| HAM/TSP#2 | 98 | chr18_26798408_- | 0 | 0 | 0 | 0 | 0 | 3 |
| HAM/TSP#2 | 99 | chr22_47971985_- | 6 | 0 | 0 | 0 | 0 | 3 |
| HAM/TSP#2 | 100 | chrX_145857134_- | 0 | 0 | 0 | 0 | 0 | 3 |
| HAM/TSP#2 | 101 | chr1_246914743_- | 27 | 0 | 0 | 0 | 0 | 2 |
| HAM/TSP#2 | 102 | chr2_168309884_+ | 16 | 0 | 7 | 0 | 5 | 2 |
| HAM/TSP#2 | 103 | chr3_165473396_+ | 2 | 0 | 0 | 0 | 0 | 2 |
| HAM/TSP#2 | 104 | chr3_174832382_- | 0 | 0 | 0 | 0 | 0 | 2 |
| HAM/TSP#2 | 105 | chr3_195167580_+ | 2 | 0 | 0 | 0 | 0 | 2 |
| HAM/TSP#2 | 106 | chr4_102251181_- | 15 | 0 | 0 | 0 | 0 | 2 |
| HAM/TSP#2 | 107 | chr4_121052340_+ | 0 | 0 | 0 | 0 | 0 | 2 |
| HAM/TSP#2 | 108 | chr4_186758777_- | 9 | 0 | 0 | 0 | 0 | 2 |
| HAM/TSP#2 | 109 | chr5_34702589_- | 6 | 0 | 0 | 0 | 0 | 2 |
| HAM/TSP#2 | 110 | chr5_75639542_- | 1 | 0 | 0 | 0 | 0 | 2 |
| HAM/TSP#2 | 111 | chr6_73518629_- | 0 | 0 | 0 | 0 | 0 | 2 |
| HAM/TSP#2 | 112 | chr6_82668565_+ | 0 | 0 | 0 | 0 | 0 | 2 |
| HAM/TSP#2 | 113 | chr7_53749296_- | 4 | 0 | 0 | 0 | 0 | 2 |
| HAM/TSP#2 | 114 | chr8_33277948_- | 4 | 1 | 0 | 0 | 5 | 2 |
| HAM/TSP#2 | 115 | chr8_99851848_+ | 0 | 0 | 0 | 0 | 0 | 2 |
| HAM/TSP#2 | 116 | chr11_115447789_- | 1 | 0 | 0 | 0 | 0 | 2 |
| HAM/TSP#2 | 117 | chr13_54486230_- | 0 | 0 | 0 | 0 | 0 | 2 |
| HAM/TSP#2 | 118 | chr13_97504880_+ | 12 | 1 | 0 | 0 | 0 | 2 |
| HAM/TSP#2 | 119 | chr14_39928704_+ | 16 | 0 | 0 | 0 | 0 | 2 |
| HAM/TSP#2 | 120 | chr14_85935867_+ | 2 | 0 | 0 | 0 | 0 | 2 |
| HAM/TSP#2 | 121 | chr14_87050616_+ | 0 | 0 | 0 | 0 | 0 | 2 |
| HAM/TSP#2 | 122 | chr15_45893830_+ | 0 | 0 | 0 | 0 | 0 | 2 |
| HAM/TSP#2 | 123 | chr16_59438651_- | 0 | 0 | 0 | 0 | 0 | 2 |
| HAM/TSP#2 | 124 | chr17_21514789_+ | 0 | 0 | 0 | 0 | 0 | 2 |
| HAM/TSP#2 | 125 | chr18_3925578_+ | 0 | 0 | 0 | 0 | 0 | 2 |
| HAM/TSP#2 | 126 | chr18_54828035_+ | 0 | 0 | 0 | 0 | 0 | 2 |
| HAM/TSP#2 | 127 | chr18_78571092_- | 0 | 0 | 0 | 0 | 0 | 2 |
| HAM/TSP#2 | 128 | chr19_578941_+ | 0 | 0 | 0 | 0 | 0 | 2 |
| HAM/TSP#2 | 129 | chr20_217991_- | 0 | 0 | 0 | 0 | 0 | 2 |
| HAM/TSP#2 | 130 | chr22_39402513_+ | 0 | 0 | 0 | 0 | 0 | 2 |
| HAM/TSP#2 | 131 | chr1_154161438_- | 6 | 0 | 0 | 0 | 0 | 1 |
| HAM/TSP#2 | 132 | chr1_35962350_- | 0 | 0 | 0 | 0 | 0 | 1 |
| HAM/TSP#2 | 133 | chr2_136041126_+ | 5 | 0 | 0 | 0 | 0 | 1 |
| HAM/TSP#2 | 134 | chr2_168477553_- | 0 | 0 | 0 | 0 | 0 | 1 |
| HAM/TSP#2 | 135 | chr2_170547066_- | 0 | 0 | 0 | 0 | 0 | 1 |
| HAM/TSP#2 | 136 | chr2_175958189_- | 4 | 0 | 0 | 0 | 0 | 1 |
| HAM/TSP#2 | 137 | chr2_217524316_- | 0 | 0 | 0 | 0 | 0 | 1 |
| HAM/TSP#2 | 138 | chr2_241194529_+ | 0 | 0 | 0 | 0 | 0 | 1 |
| HAM/TSP#2 | 139 | chr2_35142030_- | 0 | 0 | 0 | 0 | 0 | 1 |
| HAM/TSP#2 | 140 | chr2_85284281_+ | 0 | 0 | 0 | 0 | 0 | 1 |
| HAM/TSP#2 | 141 | chr3_159412803_+ | 0 | 0 | 0 | 0 | 0 | 1 |
| HAM/TSP#2 | 142 | chr4_186817805_+ | 0 | 0 | 0 | 0 | 0 | 1 |
| HAM/TSP#2 | 143 | chr4_40550690_+ | 0 | 0 | 0 | 0 | 0 | 1 |
| HAM/TSP#2 | 144 | chr4_65613059_+ | 0 | 0 | 0 | 0 | 0 | 1 |
| HAM/TSP#2 | 145 | chr4_67728873_- | 0 | 11 | 0 | 0 | 0 | 1 |
| HAM/TSP#2 | 146 | chr5_140131644_+ | 0 | 0 | 0 | 0 | 0 | 1 |
| HAM/TSP#2 | 147 | chr5_34002140_- | 0 | 0 | 0 | 0 | 0 | 1 |
| HAM/TSP#2 | 148 | chr6_135204854_- | 0 | 0 | 0 | 0 | 0 | 1 |
| HAM/TSP#2 | 149 | chr6_85736307_+ | 0 | 0 | 0 | 0 | 0 | 1 |
| HAM/TSP#2 | 150 | chr7_57719913_+ | 7 | 0 | 0 | 0 | 0 | 1 |
| HAM/TSP#2 | 151 | chr7_67976008_- | 0 | 0 | 0 | 0 | 0 | 1 |
| HAM/TSP#2 | 152 | chr8_109071227_+ | 0 | 0 | 0 | 0 | 0 | 1 |
| HAM/TSP#2 | 153 | chr8_128209578_+ | 0 | 0 | 0 | 0 | 0 | 1 |
| HAM/TSP#2 | 154 | chr8_142849282_+ | 5 | 0 | 0 | 0 | 0 | 1 |
| HAM/TSP#2 | 155 | chr8_37889008_+ | 0 | 0 | 0 | 0 | 0 | 1 |
| HAM/TSP#2 | 156 | chr8_53227890_- | 21 | 0 | 0 | 0 | 0 | 1 |
| HAM/TSP#2 | 157 | chr9_112347945_- | 0 | 0 | 0 | 0 | 0 | 1 |
| HAM/TSP#2 | 158 | chr9_21809386_- | 0 | 0 | 0 | 0 | 0 | 1 |
| HAM/TSP#2 | 159 | chr10_117150398_- | 0 | 0 | 0 | 0 | 0 | 1 |
| HAM/TSP#2 | 160 | chr11_108466251_+ | 2 | 0 | 0 | 0 | 0 | 1 |
| HAM/TSP#2 | 161 | chr12_4708662_+ | 1 | 0 | 0 | 0 | 0 | 1 |
| HAM/TSP#2 | 162 | chr13_21421916_+ | 1 | 1 | 0 | 0 | 0 | 1 |
| HAM/TSP#2 | 163 | chr14_19692823_+ | 0 | 0 | 0 | 0 | 0 | 1 |
| HAM/TSP#2 | 164 | chr14_68659681_+ | 0 | 0 | 0 | 0 | 0 | 1 |
| HAM/TSP#2 | 165 | chr15_101742561_+ | 3 | 0 | 0 | 0 | 0 | 1 |
| HAM/TSP#2 | 166 | chr15_24913524_- | 0 | 0 | 0 | 0 | 0 | 1 |
| HAM/TSP#2 | 167 | chr16_12012678_+ | 0 | 0 | 0 | 0 | 0 | 1 |
| HAM/TSP#2 | 168 | chr16_15649535_+ | 0 | 0 | 0 | 0 | 0 | 1 |
| HAM/TSP#2 | 169 | chr16_8000839_+ | 10 | 0 | 0 | 0 | 0 | 1 |
| HAM/TSP#2 | 170 | chr17_74998257_- | 0 | 0 | 0 | 0 | 0 | 1 |
| HAM/TSP#2 | 171 | chr17_80297544_- | 0 | 0 | 0 | 0 | 0 | 1 |
| HAM/TSP#2 | 172 | chr18_32013506_+ | 15 | 1 | 0 | 0 | 0 | 1 |
| HAM/TSP#2 | 173 | chr19_17876176_- | 0 | 0 | 0 | 0 | 0 | 1 |
| HAM/TSP#2 | 174 | chr20_32064265_+ | 0 | 0 | 0 | 0 | 0 | 1 |
| HAM/TSP#2 | 175 | chr21_9359272_+ | 0 | 119 | 1 | 2 | 0 | 1 |
| HAM/TSP#2 | 176 | chr22_19828856_- | 0 | 0 | 0 | 0 | 0 | 1 |
| HAM/TSP#2 | 177 | chr22_47555325_+ | 17 | 0 | 0 | 0 | 0 | 1 |
| HAM/TSP#2 | 178 | chrX_111230434_- | 1 | 0 | 0 | 0 | 0 | 1 |
| HAM/TSP#2 | 179 | chrX_24630506_+ | 0 | 0 | 0 | 0 | 0 | 1 |
| HAM/TSP#3 | 1 | chr7_94809435_- | 28 | 0 | 0 | 0 | 0 | 4 |
| HAM/TSP#3 | 2 | chr1_183358897_- | 0 | 0 | 0 | 0 | 0 | 2 |
| HAM/TSP#3 | 3 | chr13_20302065_- | 11 | 0 | 0 | 0 | 0 | 2 |
| HAM/TSP#3 | 4 | chr10_115443574_- | 0 | 0 | 0 | 0 | 0 | 1 |
| HAM/TSP#3 | 5 | chr14_69568423_- | 18 | 0 | 0 | 0 | 0 | 1 |
| HAM/TSP#3 | 6 | chr17_76377315_- | 45 | 0 | 0 | 0 | 0 | 1 |
| HAM/TSP#3 | 7 | chr19_53295299_- | 0 | 0 | 0 | 0 | 0 | 1 |
| HAM/TSP#3 | 8 | chr2_129319523_+ | 0 | 0 | 0 | 0 | 0 | 1 |
| HAM/TSP#3 | 9 | chr2_152269816_- | 8 | 0 | 0 | 0 | 0 | 1 |
| HAM/TSP#3 | 10 | chr21_41823298_- | 0 | 0 | 0 | 0 | 0 | 1 |
| HAM/TSP#3 | 11 | chr21_45485709_+ | 0 | 0 | 0 | 0 | 0 | 1 |
| HAM/TSP#3 | 12 | chr22_35171055_+ | 0 | 0 | 0 | 0 | 0 | 1 |
| HAM/TSP#3 | 13 | chr3_163378618_+ | 0 | 0 | 0 | 0 | 0 | 1 |
| HAM/TSP#3 | 14 | chr3_98938842_- | 0 | 0 | 0 | 0 | 0 | 1 |
| HAM/TSP#3 | 15 | chr5_171693329_- | 0 | 0 | 0 | 0 | 0 | 1 |
| HAM/TSP#3 | 16 | chr5_44149298_- | 5 | 0 | 0 | 0 | 0 | 1 |
| HAM/TSP#3 | 17 | chr7_28671277_- | 0 | 0 | 0 | 0 | 0 | 1 |
| HAM/TSP#3 | 18 | chr7_56268804_+ | 24 | 0 | 0 | 0 | 0 | 1 |
| HAM/TSP#3 | 19 | chr7_91699980_- | 17 | 0 | 0 | 0 | 0 | 1 |
| HAM/TSP#3 | 20 | chr9_22422521_- | 78 | 0 | 0 | 1 | 1 | 1 |

CD4 : CD4 T cells, CD8 : CD8 T cells, B : B cells, Mono : Monocytes, Neut : Neutrophils, Neut(1year) : Neutrophils 1 year later
